# Novel phosphate-activated macrophages prevent ectopic calcification by increasing extracellular ATP and pyrophosphate

**DOI:** 10.1371/journal.pone.0174998

**Published:** 2017-03-31

**Authors:** Ricardo Villa-Bellosta, Magda R. Hamczyk, Vicente Andrés

**Affiliations:** 1 Centro Nacional de Investigaciones CardiovascularesCarlos III (CNIC), Madrid, Spain; 2 Centro de Investigación Biomédica en Red de Enfermedades Cardiovasculares (CIBERCV), Madrid, Spain; 3 Fundación Instituto de Investigación Sanitaria Fundación Jiménez Díaz (FIIS-FJD), Madrid, Spain; Brigham and Women's Hospital, Harvard Medical School, UNITED STATES

## Abstract

**Purpose:**

Phosphorus is an essential nutrient involved in many pathobiological processes. Less than 1% of phosphorus is found in extracellular fluids as inorganic phosphate ion (Pi) in solution. High serum Pi level promotes ectopic calcification in many tissues, including blood vessels. Here, we studied the effect of elevated Pi concentration on macrophage polarization and calcification. Macrophages, present in virtually all tissues, play key roles in health and disease and display remarkable plasticity, being able to change their physiology in response to environmental cues.

**Methods and results:**

High-throughput transcriptomic analysis and functional studies demonstrated that Pi induces unpolarized macrophages to adopt a phenotype closely resembling that of alternatively-activated M2 macrophages, as revealed by arginine hydrolysis and energetic and antioxidant profiles. Pi-induced macrophages showed an anti-calcifying action mediated by increased availability of extracellular ATP and pyrophosphate.

**Conclusion:**

We conclude that the ability of Pi-activated macrophages to prevent calcium-phosphate deposition is a compensatory mechanism protecting tissues from hyperphosphatemia-induced pathologic calcification.

## Introduction

Phosphorus is an essential nutrient involved in many biological processes, including cell signaling, nucleic acid synthesis, energy metabolism, membrane function, and bone mineralization. Phosphorus is found in hard tissues (~85%), soft tissues (~14%), and extracellular fluids (~1%)[1]. The majority of phosphorus present in extracellular fluids exists in solution as a free phosphate ion, called inorganic phosphate (Pi). Plasma Pi has a concentration of 0.8–1.5 mmol/L, and exists in three forms: bound to proteins, complexed to cations, and ionized. Serum phosphate concentration is the outcome of the balance among phosphate absorption from the intestine, mobilization from bone, and renal excretion in urine [2]. Phosphate homeostasis is mainly regulated by phosphatonins, including vitamin D, parathyroid hormone (PTH), and fibroblast growth factor 23 (FGF23)[3].

Abnormally high serum phosphate is a pathologic condition called hyperphosphatemia. Hyperphospatemia can result from: 1) increased phosphate intake (eg, enema or vitamin D intoxication), 2) decreased phosphate excretion (associated with renal failure and hypoparathyroidism), 3) disorders that shifts intracellular phosphate to the extracellular space (including massive hemolysis), or genetic causes[4]. Acute hyperphosphatemia can cause calcification in soft tissues such as the myocardium, lung, and occasionally the liver[5]. In contrast, chronic hyperphosphatemia promotes calcification in many tissues, particularly blood vessels, kidney, bone, skin, joints, and the heart[6]. Elevated phosphate has long been recognized as an major risk factor for all-cause and cardiovascular mortality in patients with chronic kidney disease (CKD)[7]. Moreover, in recent years, this observation has been extended to the general population, where serum phosphate has been linked to the severity of coronary atherosclerosis, increased carotid intima thickness and left ventricle hypertrophy[8,9].

Macrophages are present in virtually all tissues and have immune functions as well as vital homeostatic roles, such as erythrocyte clearance [10]. Tissue-resident macrophages are collectively known as the mononuclear phagocyte system. All these myeloid linage cells have specific names depending on their location, for example Kupffer cell in liver, Langerhans cell in skin and mucosa, histiocyte in connective tissue, microglia in the central nervous system, and osteoclasts in bone[11–14]. Macrophages display remarkable plasticity and can change their physiology in response to environmental cues[15], giving rise to different cell populations with distinct functions[16]. For example, tissue-resident monocytes are activated by T_H_1 and T_H_2 cytokines to respectively form M1φs and M2φs, considered to represent the extremes of the heterogeneous spectrum of macrophage subsets[17]. Other macrophages subtypes include oxidized phospholipid-activated Mox, CXCL4-activated M4, and hemoglobin-activated M(Hb) macrophages[18,19].

Age-dependent vascular calcification is a major independent predictor of morbimortality associated with cardiovascular disease [8,9]. Intimal calcification, a frequent process in atherosclerosis, is associated with the presence of macrophages in lipid-rich areas of atherosclerotic plaques [20]; moreover, different stages in the atherosclerosis progression are associated with the presence of distinct macrophage subtypes [21]. The prevailing view is that macrophages promote intimal calcification in part by releasing substances that induce osteogenic differentiation and mineralization of vascular smooth muscle cells (VSMCs) [22,23]. However, a recent report demonstrated that alternatively-activated macrophages (M2φs) have an anti-calcifying activity not elicited by classically-activated macrophages (M1φs)[24]. Since hyperphosphatemia is a common condition that predisposes to calcification, in this study we analyzed whether high Pi concentration stimulates macrophage activation and the effects of Pi-induced macrophages on ectopic calcification.

## Materials and methods

### Mice

Male mice aged 8 to 9 weeks were used in this study (C57BL/6 background, Charles River Laboratories). Animal studies were approved by the Centro Nacional de Investigaciones Cardiovasculares (CNIC) ethics committee and conformed to directive 2010/63EU and recommendation 2007/526/EC regarding the protection of animals used for experimental and other scientific purposes, enforced in Spanish law under RD1201/2005.

### Isolation and polarization of macrophages from mouse bone marrow

Mice were euthanized by carbon dioxide inhalation to obtain tibiae and femurs. Bone-marrow-derived macrophages (BMDMs) were obtained by flushing mouse tibiae and femurs with ice-cold PBS and passing the suspension through a cell strainer with a 70-μm cut-off[25]. Cells were seeded on non-treated cell culture plates containing10 mL complete DMEM (DMEM, supplemented with 1 mmol/L L-glutamine, 100 IU/mL penicillin, 100 μg/mL streptomycin, and 10% (v/v) FBS) supplemented with 15% L929-cell-conditioned medium as a source of macrophage colony-stimulating factor (M-CSF) (L929 cells from American Type Culture Collection, Ref. CCL-1^™^). Cultures were incubated at 37°C, 5% CO_2_ for 7 days, at which time ∼95% were CD11b+ BMDMs (unpolarized macrophages: M0φs). To obtain MPiφs, M0φs were washed and cultured over the indicated period in complete DMEM supplemented with 1.6mmol/LPi (final Pi concentration, 2.5mmol/L). Control M0φs were incubated with complete DMEM. In both cases, the source of M-CSF was maintained.

### Quantitative real-time RT-PCR

Total RNA was isolated from macrophages with QIAzol lysis reagent (Qiagen, Madrid, Spain). After DNase treatment, 2μg RNA was reverse transcribed with the High Capacity cDNA Reverse Transcription Kit (Applied Biosystems, Madrid, Spain). Quantitative real-time polymerase chain reaction was performed using Power SYBR Green Mix on 384-well clear optical reaction plates, using the ABI PRISM 7900HT sequence detection system according to the manufacturer´s instruction for calibrator normalization (Applied Biosystems). All reactions were performed in triplicate. The primers used for amplification were as follows: **(1) iNOS** (NM_010927.3): 5′-GGA TCT TCC CAG GCA ACC A-3′ (forward), 5′-TCC ACA ACT CGC TCC AAG ATT-3′ (reverse); **(2) ArgI** (NM_007482.3): 5′-CAG AAG AAT GGA AGA GTC AG-3′ (forward), 5′-CAG ATA TGC AGG GAG TCA CC-3′ (reverse); **(3) eNPP1** (NM_008813): 5′-GGATTGTGCCAATAAGGACT-3′ (forward), 5′-CAAGAACTGTTGCTGCTGGAG-3′ (reverse); **(4) TNAP** (NM_007431): 5′-CTATGTCTGGAACCGCACTGA-3′ (forward), 5′- AGCCTTTGAGGTTTTTGGTCA-3′ (reverse); **(5) eNTPD1** (NM_009848): 5′-AGCCTCCACACAGATCACCTT-3′ (forward), 5′- GCCACCACTTGAAACCTGAAT-3′ (reverse); **(6) HIF-1α** (NM_010431.2): 5'-CAA GTT GGAACTGGTGGAAAA-3' (forward), 5'-GATTCATCA GTGGTGGCAGTT-3' (reverse); **(7) PGC-1β** (NM_133249.2): 5'-CCT GGA TAC AGA GACCCA CAA-3' (forward), 5'-CCA AGA GAG TCG CTT TGT GAC-3' (reverse). Expression was quantified by the comparative C_t_ method, with correction for the expression of the endogenous control gene acidic ribosomal phosphoprotein P0 (RPLP0, accession number NM_007475).

### RNAseq library preparation, sequencing, and FastQ file generation

Total RNA (500 ng) was used to generate barcoded RNA-seq libraries using the TruSeq RNA Sample Preparation Kit v2 (Illumina). Briefly, poly A+ RNA was prepared by two rounds of purification with poly-T oligo-attached magnetic beads, followed by fragmentation and first and second cDNA strand synthesis. Next, cDNA 3’ ends were adenylated and the adapters ligated, followed by PCR library amplification. Finally, the library size was checked using the Agilent 2100 Bioanalyzer DNA 1000 chip and RNA concentration was determined using the Qubit^®^ fluorometer (Life Technologies). Libraries were sequenced on a HiSeq2500 (Illumina) to generate 60-base single reads. FastQ files for each sample were obtained using CASAVA v1.8 (Illumina).

### Immunnobloting

Macrophage lysates were prepared in lysis buffer (0.1% SDS, 25 mmol/L Tris-HCl pH 7.4, and protease inhibitors) and 40 μg of protein was separated by SDS-PAGE and blotted to polyvinylidenedifluoride membrane. Rabbit monoclonal anti-eNTPD1 (1/5000, ab108248, Abcam, Cambridge, United Kindom) and rabbit polyclonal anti-TNAP (1μg/mL, ab65834, Abcam) were used according to manufacturers’ instructions and as described[26].

### Flow cytometry

Macrophages were trypsinized and Fc blocking (eBioscience 14–0161) was performed before staining with PE-Cy7-conjugatedanti-F4/80 (0.01 g/L, BioLegend 123114) or FITC-conjugated anti-CD11b (0.01 g/L, BD Pharmingen 553310). For negative control, cells were stained with the corresponding isotype control antibodies (BioLegend 400522,0.01 g/L and BD Pharmingen 553988, 0.01 g/L, respectively). Cells were analyzed using a LSRII Fortessa flow cytometer. Results were analyzed using BD Diva and FlowJo software.

### ATP, ADP and PPi quantification

PPi and ATP were quantified as described [24,26]. PPi was measured with an enzyme-linked bioluminescence assay in which PPi reacts with adenosine 5-phosphosulfate (A5508, Sigma-Aldrich) in the presence of ATP sulfurylase (A8957, Sigma-Aldrich) to generate ATP. For each sample, the amount of PPi was obtained by subtracting the blank reading (reaction without ATP-sulfurylase). ATP was measured by a coupled luciferin/luciferase reaction with an ATP determination kit (Invitrogen, Paisley, United Kingdom). ADP/ATP ratio was determined with the EnzyLight^™^ ADP/ATP ratio assay kit (BioAssay Systems).

### Total antioxidant capacity and GSH and GSSG quantification

Total antioxidant capacity was quantified with the Quantichrom Antioxidant Assay Kit (Bioassay Systems). Total, oxidized, and reduced glutathione was quantified with the EnzyChrom^™^ GSH/GSSG Assay Kit (BioAssay Systems).

### Arginase and alkaline phosphatase activities

Arginase activity was quantified in lysed macrophages as described[27,28]. Macrophages were lysed in 25 mmol/LTris-Cl buffer, pH 8 containing 0.1% Triton X-100 and protease inhibitor cocktail (Roche; Cat. Num. 11836145001). For each 100 μL of lysis buffer containing 15 μg protein, 10 μL of 10 mmol/L MnCl_2_ were added, and samples were then heated at 55°C in a water bath for 10 minutes. L-arginine (100 μL, 0.5 mol/L) was added to each sample (total sample volume 210 μL) and incubated at 37° for the indicated time. The reaction was stopped by adding 800 μL of acid solution (7:3:1 H_2_O: H_2_PO_4_ 44.6N: H_2_SO_4_ 36N). After adding 40 μL of 9% (w/v) α-isonitrosopropiophenone (dissolved in absolute ethanol), samples were heated at 100°C for 30 minutes. Finally, the samples were cooled on ice in the dark for 10 minutes and absorbance was read at 550 nm. For the standard curve, serial two-fold dilutions of 50 mmol/L urea were used. Protein was quantified with the Pierce^™^ BCA^™^ Protein Assay Kit (Thermo Scientific).

Phosphatase activity was measured with the p-nitrophenylphosphate (pNPP) Phosphatase Assay Kit (BioAssay Systems) as described[29].

### Calcification assays and CPD quantification

Mouse primary VSMCs were obtained and grown on sterile polystyrene 6-well culture plates as described[30]. To obtain a passive model of calcification, confluent VSMCs were fixed as previously described[31,32]. VSMCs were then co-cultured at 37°C and 5% CO_2_ with sterile 0.4 μm polycarbonate transwell membranes (Costar) containing M0φs or MPiφs. This co-culture system allows the effect of macrophages on calcification to be studied without interference from cell-cell interactions and VSMC activity. Macrophages and fixed VSMCs were co-incubated (after washing three times) in pro-calcifying medium: DMEM supplemented with 1 mmol/L L-glutamine, 100 IU/mL penicillin, 100 μg/mL streptomycin, 0.1% FBS and sodium phosphate (NaH_2_PO_4_^-^/Na_2_HPO_4_^2-^, pH 7.4) to a final concentration of 2.5 mmol/L. The transwell containing previously polarized macrophages and the pro-calcifying medium were both replaced daily over a 5-day period.

Calcium deposits were quantified as described[30]. Briefly, cells were treated overnight at 4°C with 0.6 mol/L HCl and analyzed with the colorimetric Calcium Quantification Assay Kit (BioAssay System, Hayward, CA).

### Statistical analysis

Results are presented as means ±SE of 3–4 independent experiments using 2–3 mice per experiment. Statistical significance was evaluated with GraphPad Prism 5 by means of unpaired Student’s t-test with Welch correction or ANOVA with Tukey’s multicomparison, as indicated in figure legends. Statistical significance was assigned at *P<0*.*05*.

## Results

### Macrophages are activated by high Pi concentration

**Fig 1:**
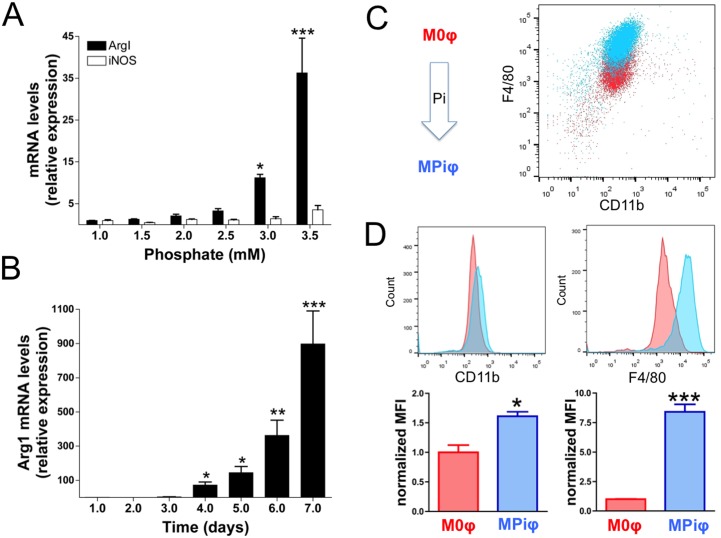
Macrophages are activated by high Pi concentration. Unpolarized M0φs were washed three times and incubated with the indicated concentration of Pi for 3 days (**A**) or incubated with 2.5 mmol/L Pi for the indicated time (**B**). Statistical significance was determined by one-way ANOVA analysis of variance followed by Tukey´s multicomparison test. (**C**) Flow cytometry studies showing activation of M0φs after exposure to high [Pi] (2.5 mmol/L) for 7 days. (**D**) Quantification of CD11b and F4/80, revealing significantly higher levels of macrophage-specific surface markers on MPiφs than on M0φs. Statistical significance was determined by Student’s *t*-test. Results are presented as mean ± SE of 3 independent experiments with 3 mice per experiment.*, *P*<0.05; **,*P*<0.01; ***,*P*<0.001.

To investigate the effect of Pi on macrophage polarization and activation during Pi-induced calcification, we performed *in vitro* assays in which unpolarized macrophages (M0φs) were incubated for 3 days in medium supplemented with a range of Pi concentrations. Pi dose-dependently induced the mRNA expression of the M2φ marker arginase 1 (*ArgI*) without significantly changing the expression of inducible nitric oxide synthase (*iNos*), a M1φ marker (Fig 1A). Time course experiments with macrophages exposed to 2.5 mmol/L Pi over 7 days revealed a progressive increase in *ArgI* mRNA starting after 4 days (Fig 1B). Moreover, flow cytometry analysis revealed significantly higher levels of the macrophage-specific surface markers F4/80 and CD11b on the surface of macrophages incubated with Pi (MPiφs) than on the surface of M0φs (Fig 1C and 1D). These results indicate that high Pi concentration activates unpolarized macrophages.

### MPiφs degrade arginine via Arg1

**Fig 2:**
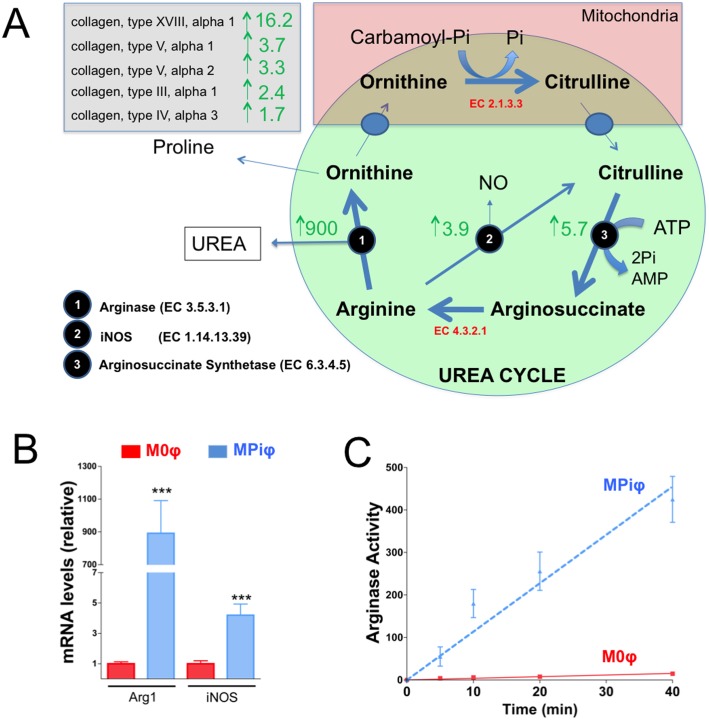
MPiφs degrade arginine via Arg1. (**A**) RNAseq data summarizing fold mRNA upregulation in MPiφs of collagen types and enzymes involved in the urea cycle. (**B**) *Arg1* and *iNOS* mRNA levels. (**C**) Arginase activity. Statistical significance was determined by Student’s *t*-test. Results are presented as mean ± SE of 4 independent experiments with 3 mice per experiment. ***, *P*<0.001.

To investigate the metabolic changes provoked by Pi in macrophages, we analyzed gene expression profiles of MPiφs and control M0φs. RNAseq analysis of macrophages derived from 5 mice in 3 independent experiments revealed 3,270 mRNAs differentially expressed in MPiφs versus M0φs. Of these mRNAs, 1,707 were upregulated and 1,563 downregulated in MPiφs (S1 Table). The most stongly up-regulated mRNA in MPiφs was *ArgI* (900-fold), whereas mRNA levels of *iNos* and arginosuccinate synthetase (an important regulatory enzyme in the urea cycle) were increased by 3.9-fold and 5.7-fold, respectively (Fig 2A). Hydrolysis of arginine by Arginase 1 produces urea and ornithine, the ornithine being transformed to proline, an important amino acid present in collagen fibers. Consistent with this, MPiφs showed elevated mRNA levels of five collagen genes and 4.8-fold downregulation of the urea transporter SLC14A1. Higher *Arg1* mRNA expression in MPiφs than in M0φs was confirmed by qPCR (Fig 2B) and was paralleled by augmented ArgI activity, quantified as the amount of urea produced from arginine (Fig 2C).

### MPiφs display an increased energetic profile

**Fig 3:**
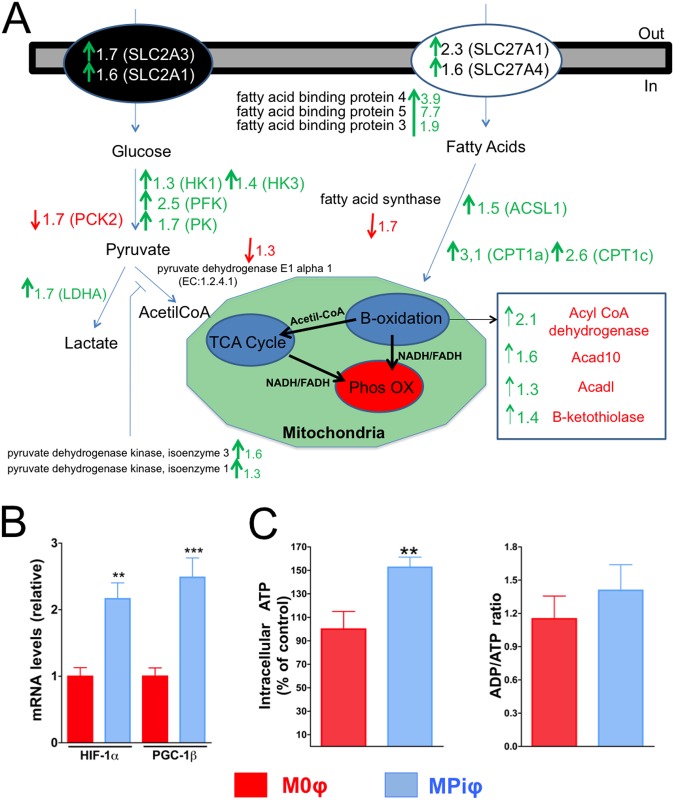
MPiφs have an enhanced energetic profile. (**A**) RNAseq data summarizing fold mRNA downregulation or upregulation in MPiφs of enzymes in the main pathways involved in glucose and fatty acid catabolism. (**B**) mRNA levels for *HIF-1α* and *PGC-1β* mRNA levels. (**C**) Intracellular ATP and ADP/ATP ratio. Statistical significance was determined by Student’s *t*-test. Results are presented as mean ± SE of 4 independent experiments with 3 mice per experiment. **, *P*<0.01; ***, *P*<0.001.

We next analyzed the energetic profile of MPiφs. Previous studies showed that M1φs synthesize ATP mainly via glycolysis, whereas M2φs primarily synthesize ATP via β-oxidation of fatty acids[33,34]. Compared with M0φs, MPiφs expressed higher mRNA transcript levels of both HIF-1α and PGC-1β, known markers of glycolysis and β-oxidation, respectively (S1 Table and Fig 3A and 3B). This was associated with a ~1.5-fold higher amount of intracellular ATP in MPiφs, with no significant difference in ADP/ATP ratio (Fig 3C). Moreover, RNAseq revealed upregulation in MPiφs of hexokinase, phosphofructokinase, and pyruvate kinase, key enzymes in glycolysis regulation (Fig 3A). MPiφs also showed upregulated mRNA expression of several transporters and enzymes involved in the transport and degradation of fatty acids via β-oxidation, including carnitine palmitoyltransferase I, acyl CoA dehydrogenase, and two fatty acid transporters (Fig 3A and S1 Table).

### MPiφs show increased antioxidant synthesis

**Fig 4:**
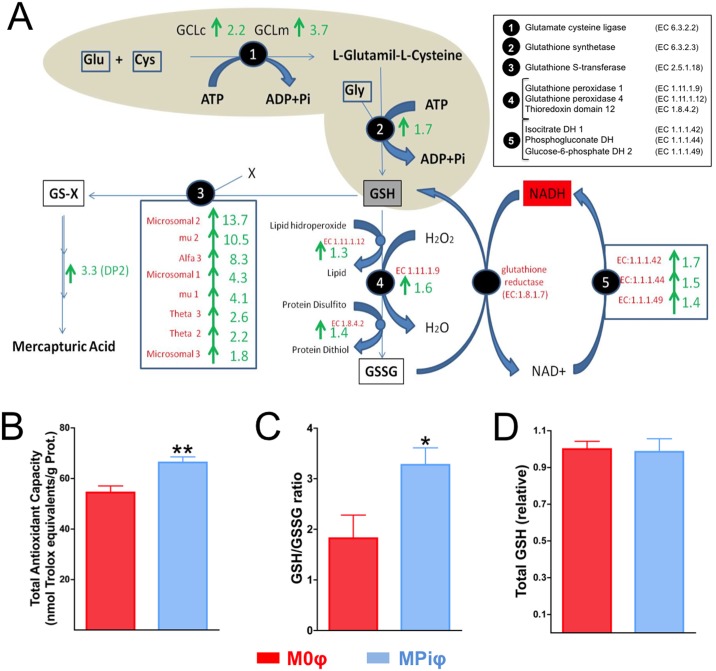
MPiφs show elevated antioxidant synthesis. (**A**) RNAseq data summarizing fold mRNA downregulation or upregulation in MPiφs of enzymes involved in glutathione metabolism. (**B**) Total antioxidant capacity. (**C**) GSH/GSSG ratio. (**D**) Total GSH. Statistical significance was determined Student’s *t*-test. Results are presented as mean ± SE of 4 independent experiments with 3 mice per experiment. *, *P*<0.05; **, *P*<0.01.

Mitochondria and NADPH oxidase are two major sources of reactive oxygen species (ROS) in macrophages. ROS include superoxide anion (O_2_^-^) and hydrogen peroxide (H_2_0_2_), and the oxidative stress they produce causes significant damage to cell structures. Cells are protected against oxidative stress by an interacting network of antioxidant enzymes that convert superoxide anion into hydrogen peroxide, which is then further reduced to water. RNAseq in MPiφs showed a 3.4-fold downregulation of *Nox1*, a gene that encodes NADPH oxidase 1, as well as upregulation of antioxidant enzymes, including superoxide dismutase 3 (9.3-fold), peroxiredoxine 1 (3.8-fold), glutathione peroxidase 1 (1.6-fold), catalase (1.4-fold), and superoxide dismutase 2 (1.3-fold) (Fig 4A and S1 Table).

In addition to upregulating antioxidant enzymes, cells respond to oxidative stress by synthesizing antioxidant metabolites, including uric acid and glutathione. Uric acid is an antioxidant produced from xanthine by the enzyme xanthine dehydrogenase. Glutathione exists in reduced (GSH) and oxidized (GSSG) states. GSH is a substrate in conjugation and reduction reactions catalyzed by glutathione S-transferase enzymes located in cytosol, microsomes, and mitochondria. Interestingly, MPiφs showed a 3.4-fold upregulation of xanthine dehydrogenase mRNA expression (S1 Table) and increased expression of 8 types of glutathione S-transferase (Fig 4A). MPiφs also showed upregulated mRNA levels of two enzymes involved in GSH synthesis and several enzymes involved in the GSH/GSSG cycle (Fig 4A). Consistent with these observations, MPiφs had a significantly higher total antioxidant capacity (Fig 4B) and a higher GSH/GSSG ratio (Fig 4C), whereas total GSH levels were not significantly altered (Fig 4D).

### MPiφs prevent calcium-phosphate deposition

Calcium-phosphate deposition (CPD) is inhibited both *in vitro* and *in vivo* by extracellular ATP[35] and pyrophosphate (PPi)[26], which is produced mainly from the hydrolysis of extracellular ATP[36]. We recently demonstrated that M2φs, but not M1φs, have an anti-calcifying activity dependent on increased availability of extracellular ATP and PPi[24].

**Fig 5:**
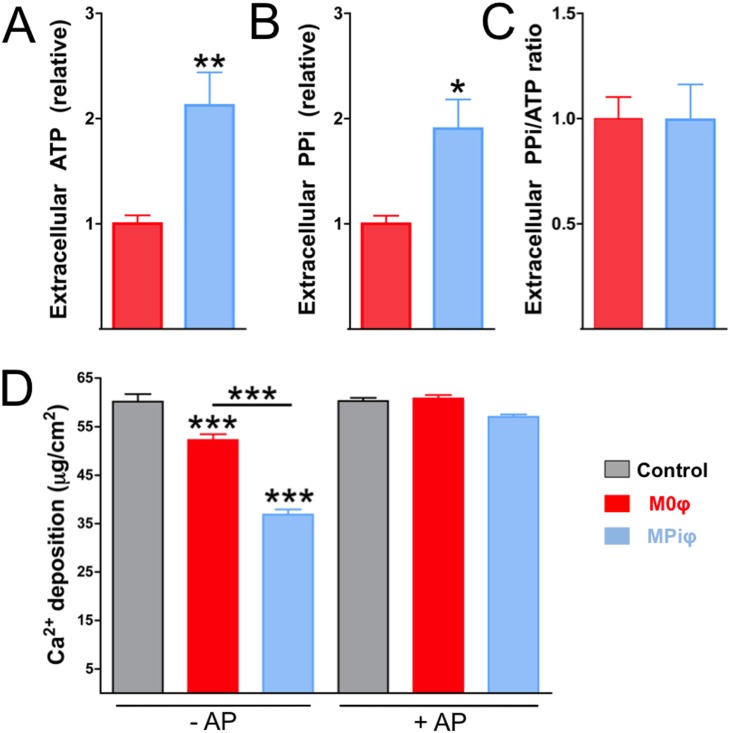
MPiφs prevent calcium-phosphate deposition by increasing extracellular ATP and PPi. Fixed VSMCs were incubated in pro-calcifying medium over 5 days with 0.4 μm polycarbonate transwells containing unpolarized M0φsor Pi-activated MPiφs. (**A, B**) Extracellular ATP and PPi released by the indicated macrophage types. (**C**) PPi/ATP ratio. Statistical significance was determined by unpaired Student’s *t*-test. Results are presented as mean ± SE of 3 independent experiments with 3 mice per experiment. (**D**) Calcium deposition on fixed VSMCs in absence (Control) or presence of M0φs or MPiφs. Experiments were performed in the presence or absence of exogenous alkaline phosphatase (-AP and +AP). Statistical significance was determined by one-way ANOVA analysis of variance followed by Tukey’s multicomparison test. Results are presented as mean ± SE of 4 independent experiments with 3 mice per experiment. *, *P*<0.05; **, *P*<0.01; ***, *P*<0.001.

The arginine degradation and the energetic and antioxidant profiles revealed by the RNAseq data and functional studies indicate that MPiφs have more in common with M2φs than with M1φs. This suggested that MPiφs might prevent CPD by increasing the availability of extracellular ATP and PPi. Supporting this idea, MPiφs produced higher levels of extracellular ATP and PPi than M0φs, with no significant differences in extracellular PPi/ATP ratio (Fig 5A–5C). Moreover, in a co-culture model of passive CPD with fixed VSMCs, MPiφs reduced the amount of deposited calcium to a significantly greater extent than M0φs (Fig 5D, -AP). Hydrolysis of extracellular polyphosphates by treatment of co-cultures with alkaline phosphatase significantly increased CPD to the level seen in the absence of macrophages (Fig 5D, +AP).

**Fig 6:**
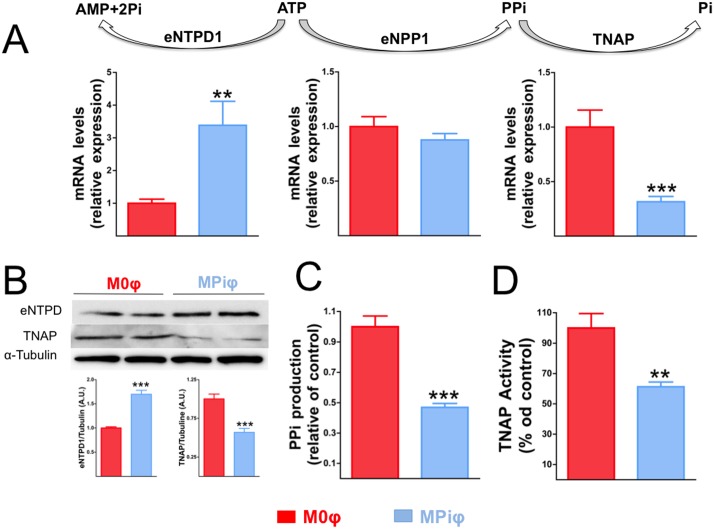
Extracellular PPi metabolism in MPiφs. **(A)** mRNA levels for the indicated ectoenzymes in unpolarized M0φs and Pi-activated MPiφs. **(B)** Immunoblot analysis of the indicated ectoenzymes. Relative levels were quantified by densitometry and were normalized to α–tubulin. **(C)** eNTPD activity, quantified as PPi produced from hydrolysis of 1 μmol/L ATP in 1h. (**D**) Alkaline phosphatase activity. Statistical significance was determined by unpaired Student’s *t*-test. Results are presented as mean ± SE of 4 independent experiments with 3 mice per experiment. **ATP**, adenosine triphosphate; **AMP**, adenosine monophosphate; **PPi**, pyrophosphate; **Pi**, inorganic phosphate; **eNPP1**, ectoenzyme nucleotide pyrophosphatase/phosphodiesterase-1; **eNTPD1**, ectonucleoside triphosphate diphosphohydrolase 1; **TNAP**, tissue non-specific alkaline phosphatase. **, *P*<0.01; ***, *P*<0,001.

These results suggested that MPiφ-mediated protection against CPD is due to the increased availability of extracellular ATP and PPi. The main ectoenzymes involved in the regulation of extracellular ATP and PPi metabolism include 1) ectonucleotide pyrophosphatase/phosphodiesterase 1 (eNPP1), which produces PPi from ATP hydrolysis; 2) tissue non-specific alkaline phosphatase (TNAP), which produces Pi from PPi hydrolysis; and 3) ectonucleoside triphosphate diphosphohydrolase 1 (eNTPD1), which produces Pi from ATP hydrolysis. Analysis by qPCR revealed similar levels of *eNPP1* mRNA in M0φs and MPiφs (Fig 6A). In contrast, compared with M0φs, MPiφs expressed ∼3.4-fold higher levels of *eNTPD1* and ∼3.2-fold lower levels of *TNAP* (Fig 6A). These differences were confirmed at the protein level by immunoblot, showing a ∼1.7-fold higher expression ofeNTPD1 and a ∼1.9-fold lower amount of TNAP in MPiφs (Fig 6B). We also measured the accumulation of extracellular PPi 1h after incubating M0φs or MPiφs with 1μmol/L ATP. In agreement with the qPCR and immunoblot data, MPiφs showed higher eNTPD activity (revealed by ∼2-fold lower production of extracellular PPi)(Fig 6C), and∼1.8-fold lower alkaline phosphatase activity (Fig 6D).

## Discussion

CPD, mainly in the form of hydroxyapatite or whitlockite, is the hallmark of calcification. Physiological CPD occurs in specific sites of hard tissues such as bone, antlers, and dentine. Soft tissues are not normally mineralized, but aging and several pathological conditions are associated with soft tissue calcification, which increases morbidity and mortality[2]. Elevated serum Pi is a key risk factor for pathological and ectopic soft tissue calcification[6]. Calcification is associated with the presence of macrophages, but our recent work showed that alternatively activated macrophages have an anti-calcifying effect[24]. We hypothesized that macrophages activated by high Pi concentration might influence ectopic calcification. In this study, we show for the first time that mouse macrophages are activated by Pi in a dose-dependent manner (Fig 1). Remarkably, this activation takes at least four days to become manifest, thereby simulating a subacute hyperphosphatemia. Pi-dependent activation of M0φs is mainly characterized by the elevated expression and activity of Arginase 1, a marker of mouse M2φs that is not expressed in M1φs[18]. Moreover, MPiφs showed higher mRNA expression of *HIF-1α* and *PGC-1β*, known markers of glycolysis and β-oxidation, respectively[33,34], and this was associated with increased amounts of intracellular and extracellular ATP (Figs 3C and 5B), as also occurs in M2φs[24].

Three subclasses of M2φs have been identified, M2a, M2b, and M2c, which differ in their mode of stimulation and the subsequent expression of surface molecules and cytokines[17,19]. For example, M2a (induced by IL-4 or IL-13) express Arginase 1, whereas M2b (induced by immune complexes in combination with IL-1β or LPS) express the anti-inflammatory IL-10, and M2c (induced by IL-10, TGFβ, or glucocorticoids) express CD163. In contrast, classically-activated M1φs release pro-inflammatory cytokines, including IL-1, IL-6, IL-12, and TNFα. Macrophages can also be classified into three groups according to their homeostatic activities: 1) M2a subclass (wound-healing macrophages), with tissue repair activity; 2) M2b/c subclass (regulatory macrophages), with anti-inflammatory activity; and 3) M1φs, (pro-inflammatory macrophage) with inflammatory and microbicide activity. Interestingly, MPiφs increase the expression of IL-1α (20.7-fold) and its natural antagonist IL1-Ra (15.7-fold; S1 Table). Moreover, MPiφs express high levels of two interleukin receptors: 1) IL-7R (∼18-fold), which is associated with several diseases including multiple sclerosis, rheumatoid arthritis and juvenile idiopathic arthritis; and 2) interleukin-1 receptor-like 2 (10.8-fold), which is unable to bind IL-1α with high affinity. Our data thus show that MPiφs, like wound-healing macrophages, have an inflammatory-neutral cytokine profile. MPiφs also have a high capacity to repair tissues due to the high expression of several collagen types (VIIIα1, 16.2-fold; Vα1, 3.7-fold; Vα2, 3.3-fold; IIIα1, 2.4-fold; and IVα3, 1.7-fold), as well as several matrix metalloproteinases (MMPs) (MMP8, 17.2-fold; MMP12, 11.8-fold; MMP13, 10.5-fold; MMP3, 6.6-fold; and MMP27, 5.6-fold).

From a functional perspective, M1φs participate in the removal of pathogens during infection through the activation of the NADPH oxidase (NOX) system and the subsequent generation of ROS, which cause significant damage to cellular structures. Our results show that MPiφs down regulate *Nox1* (3.4-fold) and upregulate several antioxidant enzymes that protect against oxidative stress, including catalase, xanthine dehydrogenase, two superoxide dismutases, two glutathione peroxidases, and eight glutathione S-transferases (see S1 Table). Consistent with these observations, MPiφs have an elevated total antioxidant capacity and a high GSH/GSSG ratio (Fig 4). We therefore conclude that MPiφs have a role unlike that of M1φs in terms of the ROS generation, and have an antioxidant gene expression profile similar to that of oxidized LDL-stimulated macrophages (Mox).

We recently reported that IL-4-activated M2φs (wound-healing macrophages) have an anti-calcifying activity that protects against CPD during ectopic calcification[24]. M2φ-dependent prevention of CPD is due to elevated amounts of extracellular PPi and ATP, resulting respectively from increased eNPP1 activity and of fatty acid β-oxidation. The present results show that MPiφs, likeIL-4-activated M2φs, prevent CPD *in vitro* via an increased availability of extracellular ATP and PPi. However, unlike M2φs, MPiφs increase extracellular PPi (Fig 5B) without significantly increasing its synthesis (Fig 5C), operating instead by decreasing expression and activity of TNAP (Fig 6), the enzyme that degrades PPi to form Pi. This reduction in TNAP activity in MPiφs may compensate the increased expression and activity of eNTPD1, the enzyme that directly competes with eNPP1 for extracellular ATP and releases Pi (not PPi) from its hydrolysis.

**Fig 7:**
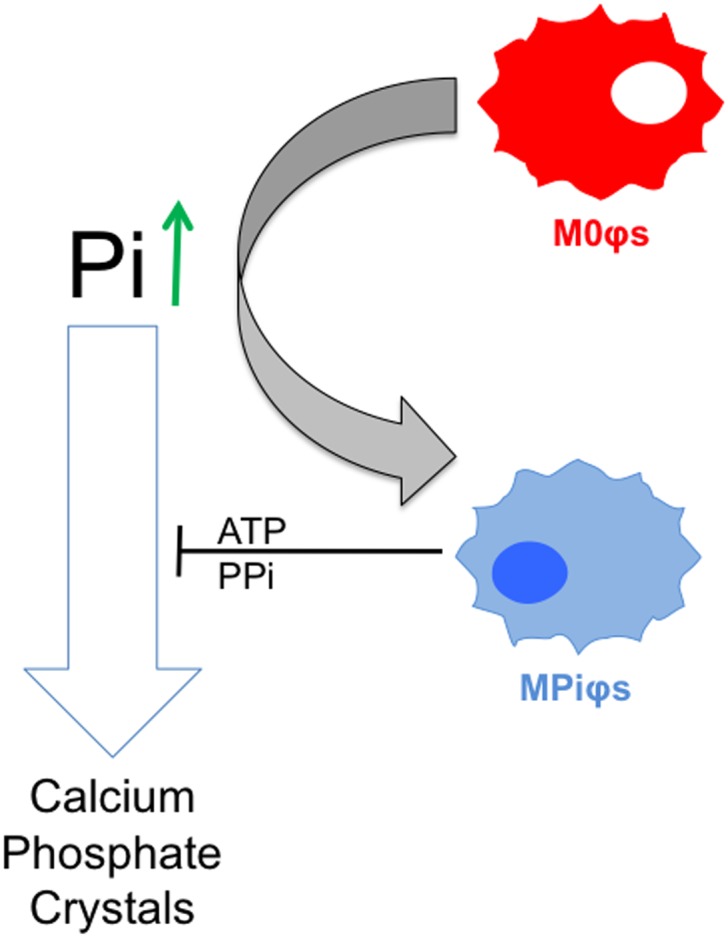
Increased release of ATP and PPi to the extracellular environment by Pi-activated macrophages protects tissues against Pi-induced calcification. Unpolarized M0φs exposed to high Pi concentrations (hyperphosphatemia) are polarized to Pi-activated macrophages (MPiφs), which release ATP and pyrophosphate (PPi), known endogenous inhibitors of calcium-phosphate crystal formation.

In conclusion, we have identified a phenotypic change in macrophages that is induced by hyperphosphatemia (MPiφs). The enhanced arginine hydrolysis in MPiφs and their energetic and antioxidant profiles together suggest classification as a new subclass of M2φs, with tissue repair and anti-calcifying activity elicited by increased accumulation of extracellular ATP/PPi. This capacity to prevent CPD could be a compensatory mechanism to protect tissues against Pi-induced calcification (Fig 7).

## Supporting information

S1 Table RNAseq analysis. Gene expression profiles of MPiφs and M0φs revealed 3,270 mRNAs differentially expressed in MPiφs versus M0φs.
(PDF)

## Abbreviations

AP: Alkaline phosphatase
ArgI: Arginase 1
CKD: Chronic kidney disease
CPD: Calcium-phosphate deposition
eNPP1: Ectoenzyme nucleotide pyrophosphatase/phosphodiesterase-1
eNTPD1: Ectonucleosidetriphosphatediphosphohydrolase 1
GSH: Reduced glutathione
GSSG: Oxidized glutathione
iNOS: Inducible NO synthetase
M0φ(s): Unpolarized macrophage(s)
M1φ(s): Classically-activated macrophage(s)
M2φ(s): Alternatively-activated macrophage(s)
MMP: matrix metalloproteinase
MPiφ(s): Phosphate-activated macrophage(s)
Pi: Inorganic phosphate
PPi: Pyrophosphate
ROS: Reactive oxygen species
TNAP: Tissue non-specific alkaline phosphatase
