# Supplementary material for: Novel phosphate-activated macrophages prevent ectopic calcification by increasing extracellular ATP and pyrophosphate
Source: PLoS One. 2017 Mar 31;12(3):e0174998. doi: 10.1371/journal.pone.0174998 (PMC5376322; doi:10.1371/journal.pone.0174998)
Supplement: S1 Table — Gene expression profiles of MPiφs and M0φs revealed 3,270 mRNAs differentially expressed in MPiφs versus M0φs. (PDF) [file pone.0174998.s001.pdf]

**Title** **Novel phosphate-activated macrophages prevent ectopic calcification by increasing extracellular ATP and pyrophosphate**

**Authors** **Ricardo Villa-Bellosta, Magda R. Hamczyk, Vicente Andrés**

## S1 Table. RNAseq analysis.

**Gene expression profiles of MPiφs and M0φs revealed 3,270 mRNAs differentially expressed in MPiφs versus M0φs.**

| ID | Name | Description | AvrExp | logFC | FC | logCPM | LR | PValue | FDR | Biotype |
|---|---|---|---|---|---|---|---|---|---|---|
| ENSMUSG00000019987 | Arg1 | arginase, liver [Source:MGI Symbol;Acc:MGI:88070] | 3,18635723 | 9,82699419 | **908,28056** | 1,73463245 | 127,906282 | 1,1767E-29 | 2,11941E-27 | protein_coding |
| ENSMUSG00000092232 | Gm20521 | predicted gene 20521 [Source:MGI Symbol;Acc:MGI:5141986] | 1,894828691 | 8,76899614 | **436,245408** | 0,90861575 | 81,205036 | 2,03473E-19 | 1,42392E-17 | protein_coding |
| ENSMUSG00000029372 | Ppbp | pro-platelet basic protein [Source:MGI Symbol;Acc:MGI:1888712] | 10,27183938 | 7,49406047 | **180,275617** | 3,38805538 | 316,252803 | 9,48717E-71 | 7,4454E-68 | protein_coding |
| ENSMUSG00000027562 | Car2 | carbonic anhydrase 2 [Source:MGI Symbol;Acc:MGI:88269] | 1,614128295 | 6,22956427 | **75,0387697** | 0,71441254 | 65,1141033 | 7,06844E-16 | 3,36194E-14 | protein_coding |
| ENSMUSG00000024245 | Tmem178 | transmembrane protein 178 [Source:MGI Symbol;Acc:MGI:1915277] | 2,643875279 | 6,04055238 | **65,8244831** | 1,42329564 | 93,4306829 | 4,20678E-22 | 4,05438E-20 | protein_coding |
| ENSMUSG00000029373 | Pf4 | platelet factor 4 [Source:MGI Symbol;Acc:MGI:1888711] | 238,5255262 | 5,92200193 | **60,6317658** | 7,89896858 | 598,58446 | 3,4017E-132 | 3,7374E-128 | protein_coding |
| ENSMUSG00000030162 | Olr1 | oxidized low density lipoprotein (lectin-like) receptor 1 [Source:MGI Symbol;Acc:MGI:1261434] | 11,25854041 | 5,60138256 | **48,5494336** | 3,51649579 | 294,633806 | 4,86317E-66 | 2,81219E-63 | protein_coding |
| ENSMUSG00000004814 | Ccl24 | chemokine (C-C motif) ligand 24 [Source:MGI Symbol;Acc:MGI:1928953] | 2,640019743 | 5,57670333 | **47,7259937** | 1,4434368 | 83,1393307 | 7,6469E-20 | 5,564E-18 | protein_coding |
| ENSMUSG00000007655 | Cav1 | caveolin 1, caveolae protein [Source:MGI Symbol;Acc:MGI:102709] | 15,63788095 | 5,53884435 | **46,4898657** | 3,97337774 | 322,715392 | 3,71054E-72 | 3,13598E-69 | protein_coding |
| ENSMUSG00000025804 | Ccr1 | chemokine (C-C motif) receptor 1 [Source:MGI Symbol;Acc:MGI:104618] | 21,10954132 | 5,52979988 | **46,1993255** | 4,42093095 | 484,152721 | 2,6677E-107 | 7,3274E-104 | protein_coding |
| ENSMUSG00000001281 | Itgb7 | integrin beta 7 [Source:MGI Symbol;Acc:MGI:96616] | 18,50262478 | 5,2730177 | **38,6666455** | 4,21429548 | 389,917751 | 8,62547E-87 | 1,57947E-83 | protein_coding |
| ENSMUSG00000028583 | Pdpn | podoplanin [Source:MGI Symbol;Acc:MGI:103098] | 18,94297446 | 5,23039541 | **37,5410062** | 4,2545855 | 347,376899 | 1,57893E-77 | 1,73477E-74 | protein_coding |
| ENSMUSG00000096951 | | Podoplanin [Source:UniProtKB/TrEMBL;Acc:A8Y5F6] | 18,94297446 | 5,23038855 | **37,5408277** | 4,25458796 | 347,446799 | 1,52455E-77 | 1,73477E-74 | protein_coding |
| ENSMUSG00000027315 | Spint1 | serine protease inhibitor, Kunitz type 1 [Source:MGI Symbol;Acc:MGI:1338033] | 6,074542701 | 5,01593113 | **32,3553219** | 2,60890259 | 128,274506 | 9,91416E-30 | 1,78987E-27 | protein_coding |
| ENSMUSG00000029819 | Npy | neuropeptide Y [Source:MGI Symbol;Acc:MGI:97374] | 31,78050909 | 4,80717685 | **27,9965441** | 4,99458787 | 350,670888 | 3,02722E-78 | 4,15751E-75 | protein_coding |
| ENSMUSG00000051379 | Flrt3 | fibronectin leucine rich transmembrane protein 3 [Source:MGI Symbol;Acc:MGI:1918686] | 6,716004208 | 4,61347282 | **24,4790017** | 2,76788845 | 150,970836 | 1,06356E-34 | 2,59674E-32 | protein_coding |
| ENSMUSG00000027399 | Il1a | interleukin 1 alpha [Source:MGI Symbol;Acc:MGI:96542] | 6,078538089 | 4,3766897 | **20,7737418** | 2,63649758 | 129,677332 | 4,8211E-30 | 9,3598E-28 | protein_coding |
| ENSMUSG00000053338 | Tarm1 | T cell-interacting, activating receptor on myeloid cells 1 [Source:MGI Symbol;Acc:MGI:2442280] | 3,556705006 | 4,36852176 | **20,656469** | 1,8668365 | 87,6867325 | 7,66871E-21 | 6,38304E-19 | protein_coding |
| ENSMUSG00000029379 | Cxcl3 | chemokine (C-X-C motif) ligand 3 [Source:MGI Symbol;Acc:MGI:3037818] | 32,78140215 | 4,35173814 | **20,417554** | 5,05752802 | 503,639229 | 1,5352E-111 | 5,6223E-108 | protein_coding |
| ENSMUSG00000028111 | Ctsk | cathepsin K [Source:MGI Symbol;Acc:MGI:107823] | 30,91543326 | 4,33428138 | **20,1719882** | 4,95145961 | 100,516982 | 1,17386E-23 | 1,25216E-21 | protein_coding |
| ENSMUSG00000004105 | Angptl2 | angiopoietin-like 2 [Source:MGI Symbol;Acc:MGI:1347002] | 38,02418501 | 4,3218223 | **19,9985334** | 5,25082965 | 119,745775 | 7,19103E-28 | 1,16188E-25 | protein_coding |
| ENSMUSG00000030208 | Emp1 | epithelial membrane protein 1 [Source:MGI Symbol;Acc:MGI:107941] | 125,4311247 | 4,29376953 | **19,6134243** | 6,97266152 | 312,484802 | 6,27963E-70 | 4,59962E-67 | protein_coding |
| ENSMUSG00000033213 | AA467197 | expressed sequence AA467197 [Source:MGI Symbol;Acc:MGI:3034182] | 2,108745164 | 4,26016689 | **19,1618757** | 1,13339309 | 61,0414341 | 5,58861E-15 | 2,2997E-13 | protein_coding |
| ENSMUSG00000040026 | Saa3 | serum amyloid A 3 [Source:MGI Symbol;Acc:MGI:98223] | 726,1513243 | 4,22604591 | **18,7139981** | 9,50521395 | 433,925372 | 2,27269E-96 | 4,99401E-93 | protein_coding |
| ENSMUSG00000003882 | Il7r | interleukin 7 receptor [Source:MGI Symbol;Acc:MGI:96562] | 85,13069302 | 4,16546171 | **17,9443991** | 6,41241668 | 132,898281 | 9,51649E-31 | 2,01072E-28 | protein_coding |
| ENSMUSG00000071714 | Csf2rb2 | colony stimulating factor 2 receptor, beta 2, low-affinity (granulocyte-macrophage) [Source:MGI Symbol;Acc:MGI:133976 | 151,784956 | 4,15579024 | **17,8245066** | 7,2469988 | 293,726184 | 7,66782E-66 | 4,21232E-63 | protein_coding |
| ENSMUSG00000061878 | Sphk1 | sphingosine kinase 1 [Source:MGI Symbol;Acc:MGI:1316649] | 6,486873259 | 4,15316721 | **17,7921285** | 2,69969877 | 101,281795 | 7,97857E-24 | 8,59417E-22 | protein_coding |
| ENSMUSG00000027068 | Dhrs9 | dehydrogenase/reductase (SDR family) member 9 [Source:MGI Symbol;Acc:MGI:2442798] | 6,020706741 | 4,14786271 | **17,7268305** | 2,60398815 | 106,216031 | 6,61196E-25 | 8,16243E-23 | protein_coding |
| ENSMUSG00000021403 | Serpinb9b | serine (or cysteine) peptidase inhibitor, clade B, member 9b [Source:MGI Symbol;Acc:MGI:894668] | 31,74720454 | 4,14223656 | **17,657835** | 4,99311294 | 182,846733 | 1,15848E-41 | 4,10588E-39 | protein_coding |
| ENSMUSG00000005800 | Mmp8 | matrix metallopeptidase 8 [Source:MGI Symbol;Acc:MGI:1202395] | 65,80831607 | 4,10565541 | **17,2157294** | 6,0452183 | 334,384299 | 1,06635E-74 | 1,06509E-71 | protein_coding |
| ENSMUSG00000001435 | Col18a1 | collagen, type XVIII, alpha 1 [Source:MGI Symbol;Acc:MGI:88451] | 38,57225199 | 4,02175937 | **16,2431482** | 5,26991752 | 68,3724093 | 1,35358E-16 | 6,98207E-15 | protein_coding |
| ENSMUSG00000032530 | Lyzl4 | lysozyme-like 4 [Source:MGI Symbol;Acc:MGI:1916282] | 2,170312386 | 4,01023106 | **16,1138694** | 1,13466923 | 54,277907 | 1,74048E-13 | 5,79473E-12 | protein_coding |
| ENSMUSG00000026981 | Il1rn | interleukin 1 receptor antagonist [Source:MGI Symbol;Acc:MGI:96547] | 285,1757045 | 3,97468773 | **15,7217263** | 8,15661071 | 310,775016 | 1,48045E-69 | 9,56805E-67 | protein_coding |
| ENSMUSG00000041324 | Inhba | inhibin beta-A [Source:MGI Symbol;Acc:MGI:96570] | 3,451797082 | 3,96554619 | **15,6224216** | 1,80408733 | 48,1408042 | 3,96685E-12 | 1,07881E-10 | protein_coding |
| ENSMUSG00000062345 | Serpinb2 | serine (or cysteine) peptidase inhibitor, clade B, member 2 [Source:MGI Symbol;Acc:MGI:97609] | 8,302799823 | 3,95566324 | **15,5157684** | 3,11215121 | 117,330086 | 2,43055E-27 | 3,70896E-25 | protein_coding |
| ENSMUSG00000027276 | Jag1 | jagged 1 [Source:MGI Symbol;Acc:MGI:1095416] | 24,57152223 | 3,93958704 | **15,3438333** | 4,62269175 | 84,1160777 | 4,66561E-20 | 3,51103E-18 | protein_coding |
| ENSMUSG00000027611 | Procr | protein C receptor, endothelial [Source:MGI Symbol;Acc:MGI:104596] | 80,33326123 | 3,93489744 | **15,2940378** | 6,32944949 | 172,734099 | 1,87085E-39 | 6,0456E-37 | protein_coding |
| ENSMUSG00000034226 | Rhov | ras homolog gene family, member V [Source:MGI Symbol;Acc:MGI:2444227] | 7,144914353 | 3,83301108 | **14,2511958** | 2,83244247 | 74,9905134 | 4,72981E-18 | 2,7939E-16 | protein_coding |
| ENSMUSG00000037406 | Htra4 | HtrA serine peptidase 4 [Source:MGI Symbol;Acc:MGI:3036260] | 2,51459752 | 3,79990541 | **13,9278958** | 1,34038594 | 52,9372242 | 3,44381E-13 | 1,09041E-11 | protein_coding |
| ENSMUSG00000074604 | Mgst2 | microsomal glutathione S-transferase 2 [Source:MGI Symbol;Acc:MGI:2448481] | 2,273277907 | 3,77574262 | **13,6965688** | 1,19073717 | 47,9116468 | 4,45865E-12 | 1,19773E-10 | protein_coding |
| ENSMUSG00000029304 | Spp1 | secreted phosphoprotein 1 [Source:MGI Symbol;Acc:MGI:98389] | 999,3238232 | 3,75941456 | **13,5424285** | 9,96487279 | 85,9272694 | 1,86677E-20 | 1,45462E-18 | protein_coding |
| ENSMUSG00000071064 | Zfp827 | zinc finger protein 827 [Source:MGI Symbol;Acc:MGI:2444807] | 2,888074892 | 3,71854692 | **13,1641906** | 1,56030583 | 56,067012 | 7,00425E-14 | 2,45865E-12 | protein_coding |
| ENSMUSG00000021508 | Cxcl14 | chemokine (C-X-C motif) ligand 14 [Source:MGI Symbol;Acc:MGI:1888514] | 1,731009265 | 3,69525159 | **12,9533342** | 0,79665362 | 43,8632314 | 3,52142E-11 | 8,35635E-10 | protein_coding |
| ENSMUSG00000038059 | 2010002N04Rik | RIKEN cDNA 2010002N04 gene [Source:MGI Symbol;Acc:MGI:1917088] | 10,85640086 | 3,63517203 | **12,4249835** | 3,45009383 | 162,008037 | 4,12034E-37 | 1,16077E-34 | protein_coding |
| ENSMUSG00000028327 | 1300002K09Rik | RIKEN cDNA 1300002K09 gene [Source:MGI Symbol;Acc:MGI:1921402] | 10,15069841 | 3,61785881 | **12,2767673** | 3,34920653 | 62,904251 | 2,17003E-15 | 9,69193E-14 | protein_coding |
| ENSMUSG00000031444 | F10 | coagulation factor X [Source:MGI Symbol;Acc:MGI:103107] | 6,367979468 | 3,61140298 | **12,2219534** | 2,70470014 | 120,024345 | 6,24891E-28 | 1,02473E-25 | protein_coding |
| ENSMUSG00000003849 | Nqo1 | NAD(P)H dehydrogenase, quinone 1 [Source:MGI Symbol;Acc:MGI:103187] | 47,79298872 | 3,60885497 | **12,2003867** | 5,58313938 | 267,482646 | 4,01388E-60 | 2,00457E-57 | protein_coding |
| ENSMUSG00000049723 | Mmp12 | matrix metallopeptidase 12 [Source:MGI Symbol;Acc:MGI:97005] | 541,4455138 | 3,5596592 | **11,791368** | 9,08080494 | 76,4426769 | 2,26699E-18 | 1,39147E-16 | protein_coding |
| ENSMUSG00000074634 | Gm7120 | predicted gene 7120 [Source:MGI Symbol;Acc:MGI:3648543] | 3,119222907 | 3,55560422 | **11,7582726** | 1,65630035 | 52,7331056 | 3,82096E-13 | 1,19945E-11 | protein_coding |
| ENSMUSG00000047414 | Flrt2 | fibronectin leucine rich transmembrane protein 2 [Source:MGI Symbol;Acc:MGI:3603594] | 26,35033824 | 3,55256898 | **11,7335607** | 4,72631304 | 151,696461 | 7,38188E-35 | 1,88616E-32 | protein_coding |
| ENSMUSG00000045092 | S1pr1 | sphingosine-1-phosphate receptor 1 [Source:MGI Symbol;Acc:MGI:1096355] | 15,76880043 | 3,54452246 | **11,6682998** | 3,98290239 | 103,69939 | 2,35464E-24 | 2,66705E-22 | protein_coding |
| ENSMUSG00000030142 | Clec4e | C-type lectin domain family 4, member e [Source:MGI Symbol;Acc:MGI:1861232] | 122,9732319 | 3,54215574 | **11,6491739** | 6,95038428 | 508,522762 | 1,3293E-112 | 7,3025E-109 | protein_coding |
| ENSMUSG00000026832 | Cytip | cytohesin 1 interacting protein [Source:MGI Symbol;Acc:MGI:2183535] | 5,578102351 | 3,48250725 | **11,1773575** | 2,48946438 | 77,9823729 | 1,03964E-18 | 6,7589E-17 | protein_coding |
| ENSMUSG00000045636 | Mtus1 | mitochondrial tumor suppressor 1 [Source:MGI Symbol;Acc:MGI:2142572] | 2,954999524 | 3,47513395 | **11,1203782** | 1,57896284 | 62,0937449 | 3,2749E-15 | 1,42111E-13 | protein_coding |
| ENSMUSG00000090145 | Ugt1a6b | UDP glucuronosyltransferase 1 family, polypeptide A6B [Source:MGI Symbol;Acc:MGI:3580629] | 10,93774141 | 3,46108036 | **11,0125782** | 3,46254161 | 104,667698 | 1,44437E-24 | 1,68822E-22 | protein_coding |
| ENSMUSG00000050860 | Phospho1 | phosphatase, orphan 1 [Source:MGI Symbol;Acc:MGI:2447348] | 11,61462687 | 3,45844196 | **10,9924568** | 3,53871831 | 108,309903 | 2,29873E-25 | 3,00669E-23 | protein_coding |
| ENSMUSG00000070942 | Il1rl2 | interleukin 1 receptor-like 2 [Source:MGI Symbol;Acc:MGI:1913107] | 2,261186585 | 3,43458286 | **10,81216** | 1,19266426 | 43,9308051 | 3,40192E-11 | 8,10594E-10 | protein_coding |
| ENSMUSG00000037759 | Ptger2 | prostaglandin E receptor 2 (subtype EP2) [Source:MGI Symbol;Acc:MGI:97794] | 10,09530626 | 3,42769577 | **10,7606683** | 3,36660947 | 162,288564 | 3,57805E-37 | 1,03453E-34 | protein_coding |
| ENSMUSG00000034220 | Gpc1 | glypican 1 [Source:MGI Symbol;Acc:MGI:1194891] | 20,80965916 | 3,40220425 | **10,5722039** | 4,38325809 | 103,38017 | 2,7663E-24 | 3,07003E-22 | protein_coding |
| ENSMUSG00000050578 | Mmp13 | matrix metallopeptidase 13 [Source:MGI Symbol;Acc:MGI:1340026] | 65,32048684 | 3,39707735 | **10,5347002** | 6,03193008 | 158,188833 | 2,81436E-36 | 7,54181E-34 | protein_coding |
| ENSMUSG00000040562 | Gstm2 | glutathione S-transferase, mu 2 [Source:MGI Symbol;Acc:MGI:95861] | 4,680217607 | 3,38979498 | **10,4816576** | 2,24271003 | 52,0267265 | 5,47503E-13 | 1,68499E-11 | protein_coding |

| | | | | | | | | | | |
|---|---|---|---|---|---|---|---|---|---|---|
| ENSMUSG00000050335 | Lgals3 | lectin, galactose binding, soluble 3 [Source:MGI Symbol;Acc:MGI:96778] | 1417,037389 | 3,38348777 | **10,4359336** | 10,4686983 | 86,890081 | 1,14723E-20 | 9,1338E-19 | protein_coding |
| ENSMUSG00000030342 | Cd9 | CD9 antigen [Source:MGI Symbol;Acc:MGI:88348] | 118,9544418 | 3,38123251 | **10,4196327** | 6,89538037 | 152,707791 | 4,43747E-35 | 1,16082E-32 | protein_coding |
| ENSMUSG00000059412 | Fxyd2 | FXYD domain-containing ion transport regulator 2 [Source:MGI Symbol;Acc:MGI:1195260] | 23,74713166 | 3,37266259 | **10,3579212** | 4,57087535 | 244,429018 | 4,25616E-55 | 1,8705E-52 | protein_coding |
| ENSMUSG00000029816 | Gpnmb | glycoprotein (transmembrane) nmb [Source:MGI Symbol;Acc:MGI:1934765] | 3903,546345 | 3,370878 | **10,3451166** | 11,9305821 | 60,2505036 | 8,35215E-15 | 3,37371E-13 | protein_coding |
| ENSMUSG00000045312 | Lhfpl2 | lipoma HMGIC fusion partner-like 2 [Source:MGI Symbol;Acc:MGI:2145236] | 163,154242 | 3,35067012 | **10,2012223** | 7,3505658 | 133,699089 | 6,35767E-31 | 1,36964E-28 | protein_coding |
| ENSMUSG00000023349 | Clec4n | C-type lectin domain family 4, member n [Source:MGI Symbol;Acc:MGI:1861231] | 35,29995447 | 3,33053149 | **10,0598124** | 5,158963 | 311,820145 | 8,7644E-70 | 6,0184E-67 | protein_coding |
| ENSMUSG00000045382 | Cxcr4 | chemokine (C-X-C motif) receptor 4 [Source:MGI Symbol;Acc:MGI:109563] | 33,49674366 | 3,29999648 | **9,84913131** | 5,06781758 | 92,1613553 | 7,98897E-22 | 7,43854E-20 | protein_coding |
| ENSMUSG00000038729 | Akap2 | A kinase (PRKA) anchor protein 2 [Source:MGI Symbol;Acc:MGI:1306795] | 7,62468515 | 3,29382242 | **9,80707169** | 2,93497781 | 22,3133425 | 2,31592E-06 | 2,32374E-05 | protein_coding |
| ENSMUSG00000037994 | Nhedc2 | Na+/H+ exchanger domain containing 2 [Source:MGI Symbol;Acc:MGI:2140077] | 4,813367023 | 3,28202797 | **9,72722286** | 2,28158799 | 72,0751683 | 2,07154E-17 | 1,1495E-15 | protein_coding |
| ENSMUSG00000072941 | Sod3 | superoxide dismutase 3, extracellular [Source:MGI Symbol;Acc:MGI:103181] | 2,860598695 | 3,2164524 | **9,2949841** | 1,53999686 | 25,2700795 | 4,9838E-07 | 5,71576E-06 | protein_coding |
| ENSMUSG00000028341 | Nr4a3 | nuclear receptor subfamily 4, group A, member 3 [Source:MGI Symbol;Acc:MGI:1352457] | 13,06451201 | 3,20963651 | **9,25117431** | 3,72778622 | 169,939597 | 7,62709E-39 | 2,32775E-36 | protein_coding |
| ENSMUSG00000030657 | Xylt1 | xylosyltransferase 1 [Source:MGI Symbol;Acc:MGI:2451073] | 3,544831942 | 3,19565264 | **9,161937** | 1,84720632 | 54,0589458 | 1,94564E-13 | 6,41945E-12 | protein_coding |
| ENSMUSG00000015094 | Npdc1 | neural proliferation, differentiation and control gene 1 [Source:MGI Symbol;Acc:MGI:1099802] | 4,023096384 | 3,17405692 | **9,02581319** | 2,01075786 | 21,4158726 | 3,69698E-06 | 3,56619E-05 | protein_coding |
| ENSMUSG00000028238 | Atp6v0d2 | ATPase, H+ transporting, lysosomal V0 subunit D2 [Source:MGI Symbol;Acc:MGI:1924415] | 49,12694519 | 3,16255001 | **8,95410979** | 5,61986427 | 88,1153343 | 6,17471E-21 | 5,30012E-19 | protein_coding |
| ENSMUSG00000014599 | Csf1 | colony stimulating factor 1 (macrophage) [Source:MGI Symbol;Acc:MGI:1339753] | 31,29511396 | 3,16204194 | **8,95095699** | 4,97111589 | 46,441294 | 9,44049E-12 | 2,4348E-10 | protein_coding |
| ENSMUSG00000025856 | Pdgfa | platelet derived growth factor, alpha [Source:MGI Symbol;Acc:MGI:97527] | 10,11056327 | 3,15467949 | **8,90539429** | 3,3427683 | 99,3791383 | 2,08507E-23 | 2,20276E-21 | protein_coding |
| ENSMUSG00000054545 | Ugt1a6a | UDP glucuronosyltransferase 1 family, polypeptide A6A [Source:MGI Symbol;Acc:MGI:2137698] | 18,68304191 | 3,14954638 | **8,87376519** | 4,2314369 | 128,79189 | 7,53153E-30 | 1,40252E-27 | protein_coding |
| ENSMUSG00000026822 | Lcn2 | lipocalin 2 [Source:MGI Symbol;Acc:MGI:96757] | 18,65933809 | 3,11397955 | **8,65767442** | 4,26967061 | 185,377659 | 3,24603E-42 | 1,1888E-39 | protein_coding |
| ENSMUSG00000064080 | Fbln2 | fibulin 2 [Source:MGI Symbol;Acc:MGI:95488] | 4,397877747 | 3,11222182 | **8,64713267** | 2,15683102 | 21,8550016 | 2,94049E-06 | 2,88973E-05 | protein_coding |
| ENSMUSG00000019256 | Ahr | aryl-hydrocarbon receptor [Source:MGI Symbol;Acc:MGI:105043] | 7,378515153 | 3,09142474 | **8,52337462** | 2,90791326 | 97,2587859 | 6,08335E-23 | 6,1319E-21 | protein_coding |
| ENSMUSG00000020399 | Havcr2 | hepatitis A virus cellular receptor 2 [Source:MGI Symbol;Acc:MGI:2159682] | 10,51627942 | 3,07867195 | **8,44836375** | 3,39781247 | 108,066753 | 2,59876E-25 | 3,32007E-23 | protein_coding |
| ENSMUSG00000021477 | Ctsl | cathepsin L [Source:MGI Symbol;Acc:MGI:88564] | 1548,358992 | 3,07070523 | **8,40183954** | 10,596579 | 102,367593 | 4,61185E-24 | 5,01687E-22 | protein_coding |
| ENSMUSG00000047415 | Gpr68 | G protein-coupled receptor 68 [Source:MGI Symbol;Acc:MGI:2441763] | 5,562629747 | 3,06338096 | **8,35929319** | 2,47746567 | 77,9370348 | 1,06378E-18 | 6,87515E-17 | protein_coding |
| ENSMUSG00000031530 | Dusp4 | dual specificity phosphatase 4 [Source:MGI Symbol;Acc:MGI:2442191] | 38,57088817 | 3,05813662 | **8,32896151** | 5,27108109 | 62,385728 | 2,82362E-15 | 1,24591E-13 | protein_coding |
| ENSMUSG00000046223 | Plaur | plasminogen activator, urokinase receptor [Source:MGI Symbol;Acc:MGI:97612] | 26,05651385 | 3,05788439 | **8,32750541** | 4,70632718 | 135,780462 | 2,22857E-31 | 4,89707E-29 | protein_coding |
| ENSMUSG00000050390 | C77080 | expressed sequence C77080 [Source:MGI Symbol;Acc:MGI:2140651] | 30,41720238 | 3,05644365 | **8,31919339** | 4,9262876 | 73,093633 | 1,23643E-17 | 7,00239E-16 | protein_coding |
| ENSMUSG00000029762 | Akr1b8 | aldo-keto reductase family 1, member B8 [Source:MGI Symbol;Acc:MGI:107673] | 53,13673239 | 3,05364371 | **8,30306335** | 5,73423972 | 89,590343 | 2,92951E-21 | 2,63824E-19 | protein_coding |
| ENSMUSG00000032561 | Acpp | acid phosphatase, prostate [Source:MGI Symbol;Acc:MGI:1928480] | 17,64708722 | 3,05305402 | **8,29967028** | 4,15147298 | 128,816316 | 7,43941E-30 | 1,40252E-27 | protein_coding |
| ENSMUSG00000025934 | Gsta3 | glutathione S-transferase, alpha 3 [Source:MGI Symbol;Acc:MGI:95856] | 14,51075494 | 3,04872535 | **8,27480523** | 3,86560415 | 53,5503077 | 2,52055E-13 | 8,19328E-12 | protein_coding |
| ENSMUSG00000010461 | Eya4 | eyes absent 4 homolog (Drosophila) [Source:MGI Symbol;Acc:MGI:1337104] | 8,655133746 | 3,04850453 | **8,27353875** | 3,12146786 | 68,5874891 | 1,21372E-16 | 6,29014E-15 | protein_coding |
| ENSMUSG00000021822 | Plau | plasminogen activator, urokinase [Source:MGI Symbol;Acc:MGI:97611] | 65,75156569 | 3,03704411 | **8,2080761** | 6,04034236 | 84,0386839 | 4,85188E-20 | 3,62636E-18 | protein_coding |
| ENSMUSG00000079227 | Ccr5 | chemokine (C-C motif) receptor 5 [Source:MGI Symbol;Acc:MGI:107182] | 23,91624581 | 3,03658122 | **8,20544296** | 4,60958606 | 253,429071 | 4,64399E-57 | 2,12598E-54 | protein_coding |
| ENSMUSG00000030109 | Slc6a12 | solute carrier family 6 (neurotransmitter transporter, betaine/GABA), member 12 [Source:MGI Symbol;Acc:MGI:95628] | 60,19991221 | 3,03592462 | **8,20170935** | 5,91572887 | 378,566685 | 2,55281E-84 | 4,00681E-81 | protein_coding |
| ENSMUSG00000063506 | Arhgap22 | Rho GTPase activating protein 22 [Source:MGI Symbol;Acc:MGI:2443418] | 16,37112505 | 3,03139732 | **8,17601204** | 4,03721882 | 127,853694 | 1,2083E-29 | 2,14122E-27 | protein_coding |
| ENSMUSG00000027737 | Slc7a11 | solute carrier family 7 (cationic amino acid transporter, y+ system), member 11 [Source:MGI Symbol;Acc:MGI:1347355] | 121,6362898 | 3,02984973 | **8,16724625** | 6,92901682 | 171,737164 | 3,08861E-39 | 9,6956E-37 | protein_coding |
| ENSMUSG00000026463 | Atp2b4 | ATPase, Ca++ transporting, plasma membrane 4 [Source:MGI Symbol;Acc:MGI:88111] | 4,678952054 | 3,01000449 | **8,05566949** | 2,24513237 | 48,9076063 | 2,68308E-12 | 7,57816E-11 | protein_coding |
| ENSMUSG00000018500 | Adora2b | adenosine A2b receptor [Source:MGI Symbol;Acc:MGI:99403] | 2,712094921 | 3,00251053 | **8,01393343** | 1,47661718 | 40,0051797 | 2,5329E-10 | 5,33123E-09 | protein_coding |
| ENSMUSG00000023992 | Trem2 | triggering receptor expressed on myeloid cells 2 [Source:MGI Symbol;Acc:MGI:1913150] | 153,9850446 | 3,00079031 | **8,00438364** | 7,266862 | 52,9899898 | 3,35253E-13 | 1,06766E-11 | protein_coding |
| ENSMUSG00000020689 | Itgb3 | integrin beta 3 [Source:MGI Symbol;Acc:MGI:96612] | 7,466099955 | 2,99449588 | **7,96953685** | 2,9013282 | 29,3869812 | 5,92745E-08 | 8,22284E-07 | protein_coding |
| ENSMUSG00000023232 | Serinc2 | serine incorporator 2 [Source:MGI Symbol;Acc:MGI:1919132] | 3,906476371 | 2,98903553 | **7,93943054** | 1,97393488 | 39,2437252 | 3,74069E-10 | 7,6392E-09 | protein_coding |
| ENSMUSG00000022246 | Rai14 | retinoic acid induced 14 [Source:MGI Symbol;Acc:MGI:1922896] | 16,56326466 | 2,98235188 | **7,90273416** | 4,05949714 | 105,472191 | 9,62391E-25 | 1,14932E-22 | protein_coding |
| ENSMUSG00000029189 | Sel1l3 | sel-1 suppressor of lin-12-like 3 (C. elegans) [Source:MGI Symbol;Acc:MGI:1916941] | 10,05425058 | 2,95690494 | **7,7645641** | 3,33822025 | 97,8827931 | 4,43894E-23 | 4,55801E-21 | protein_coding |
| ENSMUSG00000027765 | P2ry1 | purinergic receptor P2Y, G-protein coupled 1 [Source:MGI Symbol;Acc:MGI:105049] | 4,292988562 | 2,95313541 | **7,74430303** | 2,10847694 | 48,3815125 | 3,5086E-12 | 9,66141E-11 | protein_coding |
| ENSMUSG00000017417 | Plxdc1 | plexin domain containing 1 [Source:MGI Symbol;Acc:MGI:1919574] | 9,070485307 | 2,93869893 | **7,66719529** | 3,18594719 | 49,5155122 | 1,96809E-12 | 5,66059E-11 | protein_coding |
| ENSMUSG00000029207 | Apbb2 | amyloid beta (A4) precursor protein-binding, family B, member 2 [Source:MGI Symbol;Acc:MGI:108405] | 17,07157623 | 2,92818547 | **7,61152468** | 4,0970387 | 75,7732422 | 3,18184E-18 | 1,91109E-16 | protein_coding |
| ENSMUSG00000021338 | Lrrc16a | leucine rich repeat containing 16A [Source:MGI Symbol;Acc:MGI:1915982] | 7,448945074 | 2,92581001 | **7,59900227** | 2,89947384 | 61,8914309 | 3,62925E-15 | 1,55244E-13 | protein_coding |
| ENSMUSG00000071713 | Csf2rb | colony stimulating factor 2 receptor, beta, low-affinity (granulocyte-macrophage) [Source:MGI Symbol;Acc:MGI:1339759] | 321,064739 | 2,89710278 | **7,44928922** | 8,32746672 | 229,134945 | 9,20446E-52 | 3,88959E-49 | protein_coding |
| ENSMUSG00000051029 | Serpinb1b | serine (or cysteine) peptidase inhibitor, clade B, member 1b [Source:MGI Symbol;Acc:MGI:2445361] | 2,250176769 | 2,88004277 | **7,36171945** | 1,18413032 | 41,0183006 | 1,5081E-10 | 3,25531E-09 | protein_coding |
| ENSMUSG00000049866 | Arl4c | ADP-ribosylation factor-like 4C [Source:MGI Symbol;Acc:MGI:2445172] | 102,0635735 | 2,87594462 | **7,34083725** | 6,6752663 | 268,716807 | 2,16062E-60 | 1,13041E-57 | protein_coding |
| ENSMUSG00000035448 | Ccr3 | chemokine (C-C motif) receptor 3 [Source:MGI Symbol;Acc:MGI:104616] | 16,61635373 | 2,87165623 | **7,31904913** | 4,0759344 | 132,324482 | 1,27058E-30 | 2,63393E-28 | protein_coding |
| ENSMUSG00000017002 | Slpi | secretory leukocyte peptidase inhibitor [Source:MGI Symbol;Acc:MGI:109297] | 9,968839853 | 2,86874193 | **7,30427928** | 3,34332979 | 82,8838304 | 8,70201E-20 | 6,29007E-18 | protein_coding |
| ENSMUSG00000037868 | Egr2 | early growth response 2 [Source:MGI Symbol;Acc:MGI:95296] | 25,30721469 | 2,84708937 | **7,19547224** | 4,67111096 | 205,258221 | 1,48745E-46 | 5,63539E-44 | protein_coding |
| ENSMUSG00000040152 | Thbs1 | thrombospondin 1 [Source:MGI Symbol;Acc:MGI:98737] | 82,33471559 | 2,83149687 | **7,11812303** | 6,36714796 | 52,6673226 | 3,95111E-13 | 1,23326E-11 | protein_coding |
| ENSMUSG00000025473 | Adam8 | a disintegrin and metallopeptidase domain 8 [Source:MGI Symbol;Acc:MGI:107825] | 482,1253432 | 2,82658435 | **7,09392632** | 8,91341762 | 70,6597385 | 4,24467E-17 | 2,26389E-15 | protein_coding |
| ENSMUSG00000025044 | Msr1 | macrophage scavenger receptor 1 [Source:MGI Symbol;Acc:MGI:98257] | 235,3618343 | 2,82646088 | **7,09331925** | 7,8797973 | 97,9725889 | 4,24214E-23 | 4,39702E-21 | protein_coding |
| ENSMUSG00000023913 | Pla2g7 | phospholipase A2, group VII (platelet-activating factor acetylhydrolase, plasma) [Source:MGI Symbol;Acc:MGI:1351327] | 362,8218056 | 2,82295251 | **7,07609057** | 8,5038442 | 137,55959 | 9,09719E-32 | 2,03981E-29 | protein_coding |
| ENSMUSG00000039043 | 2610034B18Rik | RIKEN cDNA 2610034B18 gene [Source:MGI Symbol;Acc:MGI:1917670] | 8,302059388 | 2,81767395 | **7,05024772** | 3,07068891 | 57,2220882 | 3,89275E-14 | 1,43042E-12 | protein_coding |
| ENSMUSG00000075014 | Gm10800 | predicted gene 10800 [Source:MGI Symbol;Acc:MGI:3641657] | 9,390888642 | 2,81606872 | **7,04240753** | 3,24495722 | 52,2183949 | 4,96588E-13 | 1,5369E-11 | protein_coding |
| ENSMUSG00000040710 | St8sia4 | ST8 alpha-N-acetyl-neuraminide alpha-2,8-sialyltransferase 4 [Source:MGI Symbol;Acc:MGI:106018] | 10,08400288 | 2,81506428 | **7,03750614** | 3,34676052 | 95,8199095 | 1,25824E-22 | 1,24543E-20 | protein_coding |
| ENSMUSG00000037613 | Tnfrsf23 | tumor necrosis factor receptor superfamily, member 23 [Source:MGI Symbol;Acc:MGI:1930269] | 12,52783469 | 2,80947203 | **7,01027983** | 3,65791933 | 87,6967649 | 7,62991E-21 | 6,38304E-19 | protein_coding |
| ENSMUSG00000031209 | Heph | hephaestin [Source:MGI Symbol;Acc:MGI:1332240] | 1,757072512 | 2,80589205 | **6,99290572** | 0,85182816 | 23,221688 | 1,4436E-06 | 1,512E-05 | protein_coding |
| ENSMUSG00000056054 | S100a8 | S100 calcium binding protein A8 (calgranulin A) [Source:MGI Symbol;Acc:MGI:88244] | 3,712649527 | 2,79976224 | **6,96325687** | 1,90867715 | 55,010218 | 1,19905E-13 | 4,06603E-12 | protein_coding |
| ENSMUSG00000028364 | Tnc | tenascin C [Source:MGI Symbol;Acc:MGI:101922] | 4,862212215 | 2,79864263 | **6,95785508** | 2,29360602 | 22,4406693 | 2,16736E-06 | 2,18466E-05 | protein_coding |
| ENSMUSG00000052914 | Cyp2j6 | cytochrome P450, family 2, subfamily j, polypeptide 6 [Source:MGI Symbol;Acc:MGI:1270148] | 3,935518992 | 2,79747992 | **6,9522498** | 1,99322357 | 41,0510964 | 1,48301E-10 | 3,20745E-09 | protein_coding |
| ENSMUSG00000020592 | Sdc1 | syndecan 1 [Source:MGI Symbol;Acc:MGI:1349162] | 19,46795443 | 2,79638074 | **6,94695492** | 4,28599397 | 57,7591731 | 2,96255E-14 | 1,10338E-12 | protein_coding |
| ENSMUSG00000027864 | Ptgfrn | prostaglandin F2 receptor negative regulator [Source:MGI Symbol;Acc:MGI:1277114] | 2,949048366 | 2,79347683 | **6,93298593** | 1,58185064 | 22,6736557 | 1,91982E-06 | 1,96397E-05 | protein_coding |
| ENSMUSG00000020077 | Srgn | serglycin [Source:MGI Symbol;Acc:MGI:97756] | 48,00443097 | 2,7888957 | **6,91100585** | 5,59479531 | 328,667849 | 1,87471E-73 | 1,71645E-70 | protein_coding |
| ENSMUSG00000035373 | Ccl7 | chemokine (C-C motif) ligand 7 [Source:MGI Symbol;Acc:MGI:99512] | 71,02063929 | 2,77691159 | **6,85383562** | 6,16337903 | 258,203468 | 4,22788E-58 | 2,01964E-55 | protein_coding |
| ENSMUSG00000032578 | Cish | cytokine inducible SH2-containing protein [Source:MGI Symbol;Acc:MGI:103159] | 2,643856095 | 2,76444216 | **6,79485215** | 1,44279933 | 36,0999134 | 1,87455E-09 | 3,39303E-08 | protein_coding |
| ENSMUSG00000035385 | Ccl2 | chemokine (C-C motif) ligand 2 [Source:MGI Symbol;Acc:MGI:98259] | 114,8867319 | 2,75921795 | **6,77029149** | 6,84786189 | 160,11895 | 1,06576E-36 | 2,92737E-34 | protein_coding |
| ENSMUSG00000043613 | Mmp3 | matrix metallopeptidase 3 [Source:MGI Symbol;Acc:MGI:97010] | 1,97160671 | 2,73037848 | **6,63629712** | 1,0343963 | 32,9198392 | 9,60382E-09 | 1,53145E-07 | protein_coding |

| | | | | | | | | | | |
|---|---|---|---|---|---|---|---|---|---|---|
| ENSMUSG00000053965 | Pde5a | phosphodiesterase 5A, cGMP-specific [Source:MGI Symbol;Acc:MGI:2651499] | 1,465903725 | 2,71598729 | **6,57042772** | 0,59266165 | 24,7644409 | 6,47816E-07 | 7,27766E-06 | protein_coding |
| ENSMUSG00000028517 | Ppap2b | phosphatidic acid phosphatase type 2B [Source:MGI Symbol;Acc:MGI:1915166] | 9,140322901 | 2,70212415 | **6,50759358** | 3,20051868 | 42,0005648 | 9,12471E-11 | 2,03767E-09 | protein_coding |
| ENSMUSG00000030103 | Bhlhe40 | basic helix-loop-helix family, member e40 [Source:MGI Symbol;Acc:MGI:1097714] | 21,63436461 | 2,69805316 | **6,48925635** | 4,44129428 | 130,127719 | 3,84242E-30 | 7,67575E-28 | protein_coding |
| ENSMUSG00000050777 | Tmem37 | transmembrane protein 37 [Source:MGI Symbol;Acc:MGI:2157899] | 7,581972893 | 2,69778263 | **6,48803962** | 2,93499528 | 73,2547636 | 1,1395E-17 | 6,48687E-16 | protein_coding |
| ENSMUSG00000058624 | Gda | guanine deaminase [Source:MGI Symbol;Acc:MGI:95678] | 6,059555515 | 2,68423203 | **6,42738562** | 2,62949078 | 59,6112755 | 1,15571E-14 | 4,53493E-13 | protein_coding |
| ENSMUSG00000032487 | Ptgs2 | prostaglandin-endoperoxide synthase 2 [Source:MGI Symbol;Acc:MGI:97798] | 42,08504508 | 2,67136593 | **6,37032038** | 5,41803814 | 300,349782 | 2,76418E-67 | 1,68722E-64 | protein_coding |
| ENSMUSG00000079293 | Clec7a | C-type lectin domain family 7, member a [Source:MGI Symbol;Acc:MGI:1861431] | 33,32844724 | 2,65672296 | **6,30599036** | 5,06290534 | 140,514699 | 2,05429E-32 | 4,70219E-30 | ymorphic_pseudoge |
| ENSMUSG00000021250 | Fos | FBJ osteosarcoma oncogene [Source:MGI Symbol;Acc:MGI:95574] | 56,82152669 | 2,65388107 | **6,29358072** | 5,83095813 | 65,131327 | 7,00693E-16 | 3,36179E-14 | protein_coding |
| ENSMUSG00000049422 | Chchd10 | coiled-coil-helix-coiled-coil-helix domain containing 10 [Source:MGI Symbol;Acc:MGI:2143558] | 7,355813881 | 2,64670976 | **6,2623744** | 2,88658554 | 42,3328857 | 7,69871E-11 | 1,74404E-09 | protein_coding |
| ENSMUSG00000009185 | Ccl8 | chemokine (C-C motif) ligand 8 [Source:MGI Symbol;Acc:MGI:101878] | 4,839440896 | 2,637364 | **6,2219379** | 2,30646795 | 61,8044672 | 3,79313E-15 | 1,61531E-13 | protein_coding |
| ENSMUSG00000045362 | Tnfrsf26 | tumor necrosis factor receptor superfamily, member 26 [Source:MGI Symbol;Acc:MGI:2651928] | 23,1548043 | 2,61956376 | **6,14564215** | 4,54057403 | 90,7227298 | 1,65284E-21 | 1,51331E-19 | protein_coding |
| ENSMUSG00000037224 | Zfyve28 | zinc finger, FYVE domain containing 28 [Source:MGI Symbol;Acc:MGI:2684992] | 7,523582298 | 2,61351313 | **6,11992143** | 2,91541169 | 42,4771711 | 7,1512E-11 | 1,63348E-09 | protein_coding |
| ENSMUSG00000042190 | Cmklr1 | chemokine-like receptor 1 [Source:MGI Symbol;Acc:MGI:109603] | 21,17788911 | 2,60584926 | **6,08749745** | 4,41702557 | 151,106139 | 9,9355E-35 | 2,48094E-32 | protein_coding |
| ENSMUSG00000023905 | Tnfrsf12a | tumor necrosis factor receptor superfamily, member 12a [Source:MGI Symbol;Acc:MGI:1351484] | 3,600161945 | 2,60081309 | **6,06628421** | 1,86378312 | 23,5433721 | 1,22129E-06 | 1,29521E-05 | protein_coding |
| ENSMUSG00000001025 | S100a6 | S100 calcium binding protein A6 (calcyclin) [Source:MGI Symbol;Acc:MGI:1339467] | 53,85271274 | 2,59174044 | **6,02825497** | 5,7524923 | 90,30429 | 2,04209E-21 | 1,85425E-19 | protein_coding |
| ENSMUSG00000062515 | Fabp4 | fatty acid binding protein 4, adipocyte [Source:MGI Symbol;Acc:MGI:88038] | 10,37713854 | 2,58280128 | **5,99101847** | 3,38551155 | 54,831398 | 1,31326E-13 | 4,41247E-12 | protein_coding |
| ENSMUSG00000034612 | Chst11 | carbohydrate sulfotransferase 11 [Source:MGI Symbol;Acc:MGI:1927166] | 10,66213585 | 2,57304092 | **5,95062384** | 3,42182243 | 107,522168 | 3,42058E-25 | 4,31976E-23 | protein_coding |
| ENSMUSG00000001020 | S100a4 | S100 calcium binding protein A4 [Source:MGI Symbol;Acc:MGI:1330282] | 85,02744293 | 2,57018106 | **5,93883956** | 6,41042824 | 113,961051 | 1,32893E-26 | 1,9468E-24 | protein_coding |
| ENSMUSG00000097312 | | | 4,569929167 | 2,54652497 | **5,84225355** | 2,21745554 | 47,5811911 | 5,27724E-12 | 1,40731E-10 | lincRNA |
| ENSMUSG00000028494 | Plin2 | perilipin 2 [Source:MGI Symbol;Acc:MGI:87920] | 594,7453472 | 2,54007154 | **5,81617848** | 9,21628708 | 56,3103385 | 6,18892E-14 | 2,22214E-12 | protein_coding |
| ENSMUSG00000030605 | Mfge8 | milk fat globule-EGF factor 8 protein [Source:MGI Symbol;Acc:MGI:102768] | 59,12977126 | 2,53102005 | **5,7798019** | 5,88611039 | 37,6150503 | 8,6177E-10 | 1,64665E-08 | protein_coding |
| ENSMUSG00000026358 | Rgs1 | regulator of G-protein signaling 1 [Source:MGI Symbol;Acc:MGI:1354694] | 51,72872123 | 2,53021226 | **5,7765666** | 5,69559749 | 94,2301661 | 2,80887E-22 | 2,73107E-20 | protein_coding |
| ENSMUSG00000024087 | Cyp1b1 | cytochrome P450, family 1, subfamily b, polypeptide 1 [Source:MGI Symbol;Acc:MGI:88590] | 9,536125123 | 2,52427737 | **5,75285207** | 3,2676218 | 29,6976923 | 5,04949E-08 | 7,08702E-07 | protein_coding |
| ENSMUSG00000028542 | Slc6a9 | solute carrier family 6 (neurotransmitter transporter, glycine), member 9 [Source:MGI Symbol;Acc:MGI:95760] | 5,68609804 | 2,52246122 | **5,74561459** | 2,51582386 | 64,1259214 | 1,16716E-15 | 5,43371E-14 | protein_coding |
| ENSMUSG00000023034 | Nr4a1 | nuclear receptor subfamily 4, group A, member 1 [Source:MGI Symbol;Acc:MGI:1352454] | 9,806626045 | 2,5192741 | **5,73293569** | 3,30319868 | 82,5912364 | 1,00903E-19 | 7,19887E-18 | protein_coding |
| ENSMUSG00000076441 | Ass1 | argininosuccinate synthetase 1 [Source:MGI Symbol;Acc:MGI:88090] | 43,49543647 | 2,51661027 | **5,72236004** | 5,44622894 | 65,1158684 | 7,06211E-16 | 3,36194E-14 | protein_coding |
| ENSMUSG00000060550 | H2-Q7 | histocompatibility 2, Q region locus 7 [Source:MGI Symbol;Acc:MGI:95936] | 24,49624001 | 2,50713312 | **5,68489269** | 4,61987629 | 208,007997 | 3,73658E-47 | 1,46621E-44 | protein_coding |
| ENSMUSG00000039911 | Spsb1 | splA/ryanodine receptor domain and SOCS box containing 1 [Source:MGI Symbol;Acc:MGI:1921896] | 2,230571499 | 2,5070779 | **5,6846751** | 1,17395217 | 22,7432549 | 1,85153E-06 | 1,90475E-05 | protein_coding |
| ENSMUSG00000022636 | Alcam | activated leukocyte cell adhesion molecule [Source:MGI Symbol;Acc:MGI:1313266] | 82,3041186 | 2,50301147 | **5,66867464** | 6,36443321 | 85,2225615 | 2,66602E-20 | 2,03414E-18 | protein_coding |
| ENSMUSG00000029108 | Pcdh7 | protocadherin 7 [Source:MGI Symbol;Acc:MGI:1860487] | 24,46243419 | 2,49524474 | **5,63823942** | 4,61673674 | 97,0419809 | 6,78729E-23 | 6,77927E-21 | protein_coding |
| ENSMUSG00000027171 | Prrg4 | proline rich Gla (G-carboxyglutamic acid) 4 (transmembrane) [Source:MGI Symbol;Acc:MGI:2442211] | 3,676565079 | 2,49337792 | **5,63094835** | 1,89574948 | 31,9769026 | 1,56017E-08 | 2,40078E-07 | protein_coding |
| ENSMUSG00000032725 | Folr2 | folate receptor 2 (fetal) [Source:MGI Symbol;Acc:MGI:95569] | 64,17780759 | 2,49277731 | **5,62860461** | 6,00575613 | 80,6937319 | 2,63557E-19 | 1,83272E-17 | protein_coding |
| ENSMUSG00000070323 | Mmp27 | matrix metallopeptidase 27 [Source:MGI Symbol;Acc:MGI:3039232] | 19,02523755 | 2,47603895 | **5,56367809** | 4,26017953 | 131,800611 | 1,65427E-30 | 3,36584E-28 | protein_coding |
| ENSMUSG00000019970 | Sgk1 | serum/glucocorticoid regulated kinase 1 [Source:MGI Symbol;Acc:MGI:1340062] | 86,03368469 | 2,46090594 | **5,50562344** | 6,42803048 | 117,782406 | 1,93492E-27 | 2,99422E-25 | protein_coding |
| ENSMUSG00000032232 | Cgnl1 | cingulin-like 1 [Source:MGI Symbol;Acc:MGI:1915428] | 21,71436346 | 2,45253366 | **5,47376563** | 4,44331245 | 40,7121542 | 1,76387E-10 | 3,78508E-09 | protein_coding |
| ENSMUSG00000031565 | Fgfr1 | fibroblast growth factor receptor 1 [Source:MGI Symbol;Acc:MGI:95522] | 6,288504374 | 2,45191056 | **5,47140199** | 2,65200722 | 25,3144851 | 4,87038E-07 | 5,59152E-06 | protein_coding |
| ENSMUSG00000019122 | Ccl9 | chemokine (C-C motif) ligand 9 [Source:MGI Symbol;Acc:MGI:104533] | 289,6960125 | 2,44666175 | **5,45153214** | 8,17954359 | 146,387929 | 1,06791E-33 | 2,55068E-31 | protein_coding |
| ENSMUSG00000024912 | Fosl1 | fos-like antigen 1 [Source:MGI Symbol;Acc:MGI:107179] | 2,212899991 | 2,44459654 | **5,44373387** | 1,16356953 | 27,1690234 | 1,86422E-07 | 2,29878E-06 | protein_coding |
| ENSMUSG00000031520 | Vegfc | vascular endothelial growth factor C [Source:MGI Symbol;Acc:MGI:109124] | 3,410340408 | 2,43702643 | **5,41524432** | 1,79233341 | 33,2993405 | 7,90094E-09 | 1,27284E-07 | protein_coding |
| ENSMUSG00000028064 | Sema4a | sema domain, immunoglobulin domain (Ig), transmembrane domain (TM) and short cytoplasmic domain, (semaphorin) 4 | 28,87845227 | 2,43060954 | **5,39121161** | 4,85899366 | 169,608538 | 9,00879E-39 | 2,67512E-36 | protein_coding |
| ENSMUSG00000034471 | Caskin2 | CASK-interacting protein 2 [Source:MGI Symbol;Acc:MGI:2157062] | 6,651956545 | 2,4290735 | **5,38547463** | 2,74375081 | 46,4309685 | 9,49037E-12 | 2,44194E-10 | protein_coding |
| ENSMUSG00000043822 | Adamtsl5 | ADAMTS-like 5 [Source:MGI Symbol;Acc:MGI:1913798] | 22,02719701 | 2,42718354 | **5,37842417** | 4,46221867 | 88,7502843 | 4,47933E-21 | 3,9689E-19 | protein_coding |
| ENSMUSG00000022408 | Fam83f | family with sequence similarity 83, member F [Source:MGI Symbol;Acc:MGI:2146227] | 5,760920692 | 2,42478607 | **5,36949373** | 2,53115665 | 56,1275787 | 6,79177E-14 | 2,39171E-12 | protein_coding |
| ENSMUSG00000092534 | Gm20418 | predicted gene 20418 [Source:MGI Symbol;Acc:MGI:5141883] | 4,903893915 | 2,4230202 | **5,36292547** | 2,32191285 | 22,9138558 | 1,69426E-06 | 1,75446E-05 | protein_coding |
| ENSMUSG00000029082 | Bst1 | bone marrow stromal cell antigen 1 [Source:MGI Symbol;Acc:MGI:105370] | 15,3933369 | 2,41902307 | **5,3480875** | 3,96288224 | 108,510056 | 2,07793E-25 | 2,75063E-23 | protein_coding |
| ENSMUSG00000011148 | Adssl1 | adenylosuccinate synthetase like 1 [Source:MGI Symbol;Acc:MGI:87947] | 22,01101377 | 2,40878398 | **5,31026546** | 4,46141128 | 107,191681 | 4,0413E-25 | 5,04565E-23 | protein_coding |
| ENSMUSG00000031355 | Arhgap6 | Rho GTPase activating protein 6 [Source:MGI Symbol;Acc:MGI:1196332] | 6,047353002 | 2,40806224 | **5,30760952** | 2,60714301 | 52,6791831 | 3,92732E-13 | 1,22933E-11 | protein_coding |
| ENSMUSG00000042842 | Serpinb6b | serine (or cysteine) peptidase inhibitor, clade B, member 6b [Source:MGI Symbol;Acc:MGI:894688] | 9,641124829 | 2,4060028 | **5,30003836** | 3,28011456 | 63,1167876 | 1,94807E-15 | 8,80799E-14 | protein_coding |
| ENSMUSG00000036362 | P2ry13 | purinergic receptor P2Y, G-protein coupled 13 [Source:MGI Symbol;Acc:MGI:1921441] | 1,845784039 | 2,40322803 | **5,28985445** | 0,90982873 | 26,624843 | 2,47047E-07 | 2,98603E-06 | protein_coding |
| ENSMUSG00000024659 | Anxa1 | annexin A1 [Source:MGI Symbol;Acc:MGI:96819] | 440,9442673 | 2,39830558 | **5,27183634** | 8,78464906 | 79,6702709 | 4,42408E-19 | 3,01997E-17 | protein_coding |
| ENSMUSG00000031146 | Plp2 | proteolipid protein 2 [Source:MGI Symbol;Acc:MGI:1298382] | 75,64024748 | 2,39109998 | **5,24557156** | 6,2429848 | 92,11024 | 8,19801E-22 | 7,56903E-20 | protein_coding |
| ENSMUSG00000021614 | Vcan | versican [Source:MGI Symbol;Acc:MGI:102889] | 6,697141547 | 2,36214423 | **5,14133932** | 2,77466616 | 39,3302101 | 3,57862E-10 | 7,3492E-09 | protein_coding |
| ENSMUSG00000043939 | A530064D06Rik | RIKEN cDNA A530064D06 gene [Source:MGI Symbol;Acc:MGI:2443476] | 15,60499668 | 2,35657251 | **5,12152165** | 3,97632585 | 54,1239178 | 1,88236E-13 | 6,22936E-12 | protein_coding |
| ENSMUSG00000026271 | Gpr35 | G protein-coupled receptor 35 [Source:MGI Symbol;Acc:MGI:1929509] | 1,661916998 | 2,33895059 | **5,0593449** | 0,74507431 | 23,7840364 | 1,07773E-06 | 1,16202E-05 | protein_coding |
| ENSMUSG00000004267 | Eno2 | enolase 2, gamma neuronal [Source:MGI Symbol;Acc:MGI:95394] | 7,635576894 | 2,32852548 | **5,02291717** | 2,94709062 | 57,709651 | 3,03809E-14 | 1,12421E-12 | protein_coding |
| ENSMUSG00000028019 | Pdgfc | platelet-derived growth factor, C polypeptide [Source:MGI Symbol;Acc:MGI:1859631] | 10,09167114 | 2,32513374 | **5,01112229** | 3,34656319 | 47,3676702 | 5,88453E-12 | 1,56167E-10 | protein_coding |
| ENSMUSG00000028121 | Bcar3 | breast cancer anti-estrogen resistance 3 [Source:MGI Symbol;Acc:MGI:1352501] | 6,918009367 | 2,32084866 | **4,99626035** | 2,80122094 | 47,0800288 | 6,81465E-12 | 1,79551E-10 | protein_coding |
| ENSMUSG00000026012 | Cd28 | CD28 antigen [Source:MGI Symbol;Acc:MGI:88327] | 17,45689663 | 2,32036641 | **4,99459053** | 4,12684603 | 105,83819 | 8,00087E-25 | 9,76728E-23 | protein_coding |
| ENSMUSG00000026796 | Fam129b | family with sequence similarity 129, member B [Source:MGI Symbol;Acc:MGI:2442910] | 327,1522362 | 2,31204413 | **4,96586186** | 8,35400818 | 87,6493807 | 7,8149E-21 | 6,40764E-19 | protein_coding |
| ENSMUSG00000030144 | Clec4d | C-type lectin domain family 4, member d [Source:MGI Symbol;Acc:MGI:1298389] | 306,1582303 | 2,29946596 | **4,92275505** | 8,25891845 | 76,7530375 | 1,9373E-18 | 1,21629E-16 | protein_coding |
| ENSMUSG00000069833 | Ahnak | AHNAK nucleoprotein (desmoyokin) [Source:MGI Symbol;Acc:MGI:1316648] | 557,4277867 | 2,29025626 | **4,89142988** | 9,12296641 | 103,502389 | 2,6008E-24 | 2,91582E-22 | protein_coding |
| ENSMUSG00000019929 | Dcn | decorin [Source:MGI Symbol;Acc:MGI:94872] | 4,214026072 | 2,28276964 | **4,8661124** | 2,10264022 | 36,6803014 | 1,39179E-09 | 2,5657E-08 | protein_coding |
| ENSMUSG00000038776 | Ephx1 | epoxide hydrolase 1, microsomal [Source:MGI Symbol;Acc:MGI:95405] | 20,23944243 | 2,27843464 | **4,85151265** | 4,34574159 | 64,7040759 | 8,70346E-16 | 4,12177E-14 | protein_coding |
| ENSMUSG00000090307 | 1700071M16Rik | RIKEN cDNA 1700071M16 gene [Source:MGI Symbol;Acc:MGI:1920754] | 4,32374868 | 2,27772395 | **4,84912334** | 2,13886282 | 35,4287588 | 2,64552E-09 | 4,62839E-08 | antisense |
| ENSMUSG00000031438 | Rnf128 | ring finger protein 128 [Source:MGI Symbol;Acc:MGI:1914139] | 150,3792529 | 2,27425813 | **4,83748817** | 7,2331836 | 61,1684669 | 5,2394E-15 | 2,17228E-13 | protein_coding |
| ENSMUSG00000035473 | Galm | galactose mutarotase [Source:MGI Symbol;Acc:MGI:2442420] | 11,10628145 | 2,26714127 | **4,81368346** | 3,48529503 | 55,4671938 | 9,50318E-14 | 3,29374E-12 | protein_coding |
| ENSMUSG00000078486 | 2310042D19Rik | RIKEN cDNA 2310042D19 gene [Source:MGI Symbol;Acc:MGI:1921433] | 5,892940138 | 2,26048749 | **4,79153363** | 2,57579658 | 43,0839571 | 5,24405E-11 | 1,23112E-09 | protein_coding |
| ENSMUSG00000032802 | Srxn1 | sulfiredoxin 1 homolog (S. cerevisiae) [Source:MGI Symbol;Acc:MGI:104971] | 224,2290993 | 2,25691329 | **4,77967754** | 7,80961824 | 72,0601074 | 2,08741E-17 | 1,15248E-15 | protein_coding |
| ENSMUSG00000037679 | Inf2 | inverted formin, FH2 and WH2 domain containing [Source:MGI Symbol;Acc:MGI:1917685] | 30,58873991 | 2,25126531 | **4,76100226** | 4,93894154 | 69,1171475 | 9,27851E-17 | 4,85443E-15 | protein_coding |
| ENSMUSG00000028214 | Gem | GTP binding protein (gene overexpressed in skeletal muscle) [Source:MGI Symbol;Acc:MGI:99844] | 4,488592724 | 2,24685828 | **4,7464809** | 2,18060734 | 37,6401523 | 8,50751E-10 | 1,62843E-08 | protein_coding |
| ENSMUSG00000025854 | Fam20c | family with sequence similarity 20, member C [Source:MGI Symbol;Acc:MGI:2136853] | 72,50649251 | 2,24286821 | **4,73337167** | 6,1812808 | 103,118976 | 3,15614E-24 | 3,46765E-22 | protein_coding |

| | | | | | | | | | | |
|---|---|---|---|---|---|---|---|---|---|---|
| ENSMUSG00000031266 | Gla | galactosidase, alpha [Source:MGI Symbol;Acc:MGI:1347344] | 133,5161877 | 2,2352944 | **4,70858769** | 7,06183623 | 83,3390964 | 6,9119E-20 | 5,09671E-18 | protein_coding |
| ENSMUSG00000025372 | Baiap2 | brain-specific angiogenesis inhibitor 1-associated protein 2 [Source:MGI Symbol;Acc:MGI:2137336] | 27,77605844 | 2,2297771 | **4,69061504** | 4,7968476 | 97,2934368 | 5,97781E-23 | 6,08132E-21 | protein_coding |
| ENSMUSG00000023951 | Vegfa | vascular endothelial growth factor A [Source:MGI Symbol;Acc:MGI:103178] | 30,6366616 | 2,22518543 | **4,67570993** | 4,94187404 | 63,4160983 | 1,67345E-15 | 7,66091E-14 | protein_coding |
| ENSMUSG00000049409 | Prokr1 | prokineticin receptor 1 [Source:MGI Symbol;Acc:MGI:1929676] | 8,428132196 | 2,21996102 | **4,65880846** | 3,08937551 | 51,0710089 | 8,90843E-13 | 2,64532E-11 | protein_coding |
| ENSMUSG00000048965 | Mrgpre | MAS-related GPR, member E [Source:MGI Symbol;Acc:MGI:2441884] | 3,064805765 | 2,21785725 | **4,65201983** | 1,63425578 | 20,6119343 | 5,62444E-06 | 5,19292E-05 | protein_coding |
| ENSMUSG00000031714 | Gab1 | growth factor receptor bound protein 2-associated protein 1 [Source:MGI Symbol;Acc:MGI:108088] | 9,741295628 | 2,21539814 | **4,6440971** | 3,30654216 | 78,2937417 | 8,88028E-19 | 5,87757E-17 | protein_coding |
| ENSMUSG00000036853 | Mcoln3 | mucolipin 3 [Source:MGI Symbol;Acc:MGI:1890500] | 3,828925323 | 2,20855572 | **4,6221232** | 1,92465197 | 28,8867398 | 7,67364E-08 | 1,03703E-06 | protein_coding |
| ENSMUSG00000009654 | Oit3 | oncoprotein induced transcript 3 [Source:MGI Symbol;Acc:MGI:1201782] | 6,124003874 | 2,20775002 | **4,61954263** | 2,61861565 | 30,2326506 | 3,83204E-08 | 5,51081E-07 | protein_coding |
| ENSMUSG00000021701 | Plk2 | polo-like kinase 2 [Source:MGI Symbol;Acc:MGI:1099790] | 152,3163839 | 2,19753966 | **4,58696422** | 7,25237923 | 78,0488385 | 1,00524E-18 | 6,57416E-17 | protein_coding |
| ENSMUSG00000093765 | Gm20658 | predicted gene 20658 [Source:MGI Symbol;Acc:MGI:5313105] | 4,225566338 | 2,19183967 | **4,56887721** | 2,10103582 | 23,2763291 | 1,40316E-06 | 1,47386E-05 | rocessed_transcrip |
| ENSMUSG00000037907 | Ankrd13b | ankyrin repeat domain 13b [Source:MGI Symbol;Acc:MGI:2144501] | 9,240393643 | 2,18996423 | **4,56294172** | 3,20615308 | 44,3618778 | 2,72951E-11 | 6,64947E-10 | protein_coding |
| ENSMUSG00000031803 | B3gnt3 | UDP-GlcNAc:betaGal beta-1,3-N-acetylglucosaminyltransferase 3 [Source:MGI Symbol;Acc:MGI:2152535] | 7,05274229 | 2,18010677 | **4,53187092** | 2,82688944 | 41,32795 | 1,28715E-10 | 2,82274E-09 | protein_coding |
| ENSMUSG00000030789 | Itgax | integrin alpha X [Source:MGI Symbol;Acc:MGI:96609] | 9,030434998 | 2,17485692 | **4,51540977** | 3,18549405 | 37,0435667 | 1,15519E-09 | 2,14793E-08 | protein_coding |
| ENSMUSG00000021367 | Edn1 | endothelin 1 [Source:MGI Symbol;Acc:MGI:95283] | 4,999687093 | 2,16922521 | **4,49781777** | 2,36763511 | 35,9831525 | 1,99031E-09 | 3,59073E-08 | protein_coding |
| ENSMUSG00000032786 | Alas1 | aminolevulinic acid synthase 1 [Source:MGI Symbol;Acc:MGI:87989] | 338,8453217 | 2,16857028 | **4,49577639** | 8,40507901 | 122,195536 | 2,09173E-28 | 3,53566E-26 | protein_coding |
| ENSMUSG00000036611 | Eepd1 | endonuclease/exonuclease/phosphatase family domain containing 1 [Source:MGI Symbol;Acc:MGI:1914734] | 21,3241135 | 2,16270139 | **4,4775247** | 4,41933955 | 109,356811 | 1,35551E-25 | 1,83864E-23 | protein_coding |
| ENSMUSG00000032766 | Gng11 | guanine nucleotide binding protein (G protein), gamma 11 [Source:MGI Symbol;Acc:MGI:1913316] | 5,925904999 | 2,15608 | **4,45702175** | 2,57023246 | 37,8495192 | 7,64172E-10 | 1,4704E-08 | protein_coding |
| ENSMUSG00000028949 | Smarcd3 | SWI/SNF related, matrix associated, actin dependent regulator of chromatin, subfamily d, member 3 [Source:MGI Symbo | 1,513733572 | 2,15605735 | **4,45695176** | 0,65212971 | 13,0481756 | 0,00030358 | 0,001802937 | protein_coding |
| ENSMUSG00000043017 | Ptgir | prostaglandin I receptor (IP) [Source:MGI Symbol;Acc:MGI:99535] | 14,93804481 | 2,15031892 | **4,43925912** | 3,91530192 | 75,7616888 | 3,20051E-18 | 1,91109E-16 | protein_coding |
| ENSMUSG00000072621 | Slfn10-ps | schlafen 10, pseudogene [Source:MGI Symbol;Acc:MGI:3512288] | 20,2071341 | 2,145738 | **4,42518572** | 4,34259417 | 62,3643352 | 2,85446E-15 | 1,25448E-13 | protein_coding |
| ENSMUSG00000020937 | Plcd3 | phospholipase C, delta 3 [Source:MGI Symbol;Acc:MGI:107451] | 1,36211791 | 2,13313627 | **4,38670067** | 0,45377025 | 17,5706619 | 2,76826E-05 | 0,000216939 | protein_coding |
| ENSMUSG00000029084 | Cd38 | CD38 antigen [Source:MGI Symbol;Acc:MGI:107474] | 19,26038885 | 2,13221991 | **4,38391526** | 4,31896804 | 129,663092 | 4,85581E-30 | 9,3598E-28 | protein_coding |
| ENSMUSG00000052565 | Hist1h1d | histone cluster 1, H1d [Source:MGI Symbol;Acc:MGI:107502] | 1,624349121 | 2,11098308 | **4,31985559** | 0,7273535 | 19,5175006 | 9,96822E-06 | 8,65094E-05 | protein_coding |
| ENSMUSG00000045827 | Serpinb9 | serine (or cysteine) peptidase inhibitor, clade B, member 9 [Source:MGI Symbol;Acc:MGI:106603] | 28,47373476 | 2,11079672 | **4,31929759** | 4,83550455 | 82,3641891 | 1,13186E-19 | 8,02306E-18 | protein_coding |
| ENSMUSG00000057315 | Arhgap24 | Rho GTPase activating protein 24 [Source:MGI Symbol;Acc:MGI:1922647] | 5,057775651 | 2,10798087 | **4,31087542** | 2,34417252 | 38,1817057 | 6,44539E-10 | 1,25116E-08 | protein_coding |
| ENSMUSG00000055172 | C1ra | complement component 1, r subcomponent A [Source:MGI Symbol;Acc:MGI:1355313] | 7,350974806 | 2,10290481 | **4,29573444** | 2,89436001 | 29,5886335 | 5,34173E-08 | 7,46686E-07 | protein_coding |
| ENSMUSG00000008540 | Mgst1 | microsomal glutathione S-transferase 1 [Source:MGI Symbol;Acc:MGI:1913850] | 40,83604364 | 2,09695007 | **4,2780403** | 5,35688239 | 60,4307969 | 7,62112E-15 | 3,08979E-13 | protein_coding |
| ENSMUSG00000026748 | Plxdc2 | plexin domain containing 2 [Source:MGI Symbol;Acc:MGI:1914698] | 3,975340665 | 2,07151406 | **4,20327561** | 2,02219131 | 35,3895963 | 2,69925E-09 | 4,71489E-08 | protein_coding |
| ENSMUSG00000031596 | Slc7a2 | solute carrier family 7 (cationic amino acid transporter, y+ system), member 2 [Source:MGI Symbol;Acc:MGI:99828] | 2,118557133 | 2,0648771 | **4,18398331** | 1,12609942 | 13,291838 | 0,000266564 | 0,001611115 | protein_coding |
| ENSMUSG00000034765 | Dusp5 | dual specificity phosphatase 5 [Source:MGI Symbol;Acc:MGI:2685183] | 4,565251862 | 2,0614808 | **4,17414523** | 2,206062 | 23,8430975 | 1,04516E-06 | 1,13023E-05 | protein_coding |
| ENSMUSG00000006360 | Crip1 | cysteine-rich protein 1 (intestinal) [Source:MGI Symbol;Acc:MGI:88501] | 5,886061039 | 2,0610436 | **4,17288049** | 2,56562997 | 24,7264378 | 6,60716E-07 | 7,39235E-06 | protein_coding |
| ENSMUSG00000004044 | Ptrf | polymerase I and transcript release factor [Source:MGI Symbol;Acc:MGI:1277968] | 5,159115149 | 2,05929746 | **4,16783296** | 2,38141332 | 17,4430278 | 2,96048E-05 | 0,000229871 | protein_coding |
| ENSMUSG00000039062 | Anpep | alanyl (membrane) aminopeptidase [Source:MGI Symbol;Acc:MGI:5000466] | 197,266586 | 2,05078502 | **4,14331361** | 7,62430709 | 60,6806523 | 6,71267E-15 | 2,73156E-13 | protein_coding |
| ENSMUSG00000078485 | Plekhn1 | pleckstrin homology domain containing, family N member 1 [Source:MGI Symbol;Acc:MGI:2387630] | 12,71215799 | 2,05017756 | **4,14156939** | 3,67456659 | 47,9336758 | 4,40884E-12 | 1,19017E-10 | protein_coding |
| ENSMUSG00000031391 | L1cam | L1 cell adhesion molecule [Source:MGI Symbol;Acc:MGI:96721] | 8,428851222 | 2,04667271 | **4,13152016** | 3,08059371 | 38,519611 | 5,42063E-10 | 1,07116E-08 | protein_coding |
| ENSMUSG00000027955 | Fam198b | family with sequence similarity 198, member B [Source:MGI Symbol;Acc:MGI:1915909] | 14,36390303 | 2,04455295 | **4,12545416** | 3,84704179 | 43,9947551 | 3,29257E-11 | 7,86423E-10 | protein_coding |
| ENSMUSG00000001930 | Vwf | Von Willebrand factor homolog [Source:MGI Symbol;Acc:MGI:98941] | 24,20299151 | 2,0415022 | **4,11673961** | 4,59833343 | 88,4249765 | 5,27999E-21 | 4,60407E-19 | protein_coding |
| ENSMUSG00000024525 | Impa2 | inositol (myo)-1(or 4)-monophosphatase 2 [Source:MGI Symbol;Acc:MGI:2149728] | 3,039625346 | 2,0369033 | **4,10363751** | 1,6228644 | 27,6658625 | 1,44185E-07 | 1,82431E-06 | protein_coding |
| ENSMUSG00000058135 | Gstm1 | glutathione S-transferase, mu 1 [Source:MGI Symbol;Acc:MGI:95860] | 91,08221511 | 2,03502916 | **4,09831013** | 6,51097669 | 59,0749367 | 1,5178E-14 | 5,87186E-13 | protein_coding |
| ENSMUSG00000024812 | Tjp2 | tight junction protein 2 [Source:MGI Symbol;Acc:MGI:1341872] | 11,9735249 | 2,03200667 | **4,08973303** | 3,58950573 | 69,8467986 | 6,4094E-17 | 3,38558E-15 | protein_coding |
| ENSMUSG00000029380 | Cxcl1 | chemokine (C-X-C motif) ligand 1 [Source:MGI Symbol;Acc:MGI:108068] | 6,095300668 | 2,03065801 | **4,08591165** | 2,64457424 | 46,8231775 | 7,76891E-12 | 2,03231E-10 | protein_coding |
| ENSMUSG00000033777 | Tlr13 | toll-like receptor 13 [Source:MGI Symbol;Acc:MGI:3045213] | 151,643454 | 2,02925108 | **4,08192896** | 7,24558902 | 79,2410044 | 5,49771E-19 | 3,68313E-17 | protein_coding |
| ENSMUSG00000019852 | D10Bwg1379e | DNA segment, Chr 10, Brigham & Women's Genetics 1379 expressed [Source:MGI Symbol;Acc:MGI:106387] | 5,310315658 | 2,02867477 | **4,0802987** | 2,41373423 | 30,4362892 | 3,45013E-08 | 5,0274E-07 | protein_coding |
| ENSMUSG00000043740 | B430306N03Rik | RIKEN cDNA B430306N03 gene [Source:MGI Symbol;Acc:MGI:2443478] | 32,95480117 | 2,0245009 | **4,06851104** | 5,04877602 | 45,9411179 | 1,21861E-11 | 3,085E-10 | protein_coding |
| ENSMUSG00000037638 | Zbtb42 | zinc finger and BTB domain containing 42 [Source:MGI Symbol;Acc:MGI:3644133] | 3,514609109 | 2,02227729 | **4,0622451** | 1,81252702 | 25,505714 | 4,41075E-07 | 5,08509E-06 | protein_coding |
| ENSMUSG00000018217 | Pmp22 | peripheral myelin protein 22 [Source:MGI Symbol;Acc:MGI:97631] | 108,5531573 | 2,02092842 | **4,05844881** | 6,76345044 | 59,0970362 | 1,50085E-14 | 5,8268E-13 | protein_coding |
| ENSMUSG00000010051 | Hyal1 | hyaluronoglucosaminidase 1 [Source:MGI Symbol;Acc:MGI:96298] | 5,689389274 | 2,01371231 | **4,03819985** | 2,51847752 | 27,9328867 | 1,25597E-07 | 1,61964E-06 | protein_coding |
| ENSMUSG00000073409 | H2-Q6 | histocompatibility 2, Q region locus 6 [Source:MGI Symbol;Acc:MGI:95935] | 14,38178236 | 2,01047063 | **4,02913635** | 3,85015928 | 79,6474554 | 4,47546E-19 | 3,01997E-17 | protein_coding |
| ENSMUSG00000026980 | Ly75 | lymphocyte antigen 75 [Source:MGI Symbol;Acc:MGI:106662] | 2,215324467 | 1,99157157 | **3,97669957** | 1,17926402 | 13,2061375 | 0,000279034 | 0,00167986 | protein_coding |
| ENSMUSG00000060594 | Layn | layilin [Source:MGI Symbol;Acc:MGI:2685357] | 10,47516296 | 1,99000678 | **3,97238866** | 3,40418058 | 51,9545457 | 5,68004E-13 | 1,7432E-11 | protein_coding |
| ENSMUSG00000041959 | S100a10 | S100 calcium binding protein A10 (calpactin) [Source:MGI Symbol;Acc:MGI:1339468] | 47,43798581 | 1,98851856 | **3,96829302** | 5,57201021 | 71,5398174 | 2,71719E-17 | 1,48526E-15 | protein_coding |
| ENSMUSG00000020092 | Pald1 | phosphatase domain containing, paladin 1 [Source:MGI Symbol;Acc:MGI:1351623] | 9,161407442 | 1,97306954 | **3,92602549** | 3,20861458 | 62,0793869 | 3,29887E-15 | 1,42136E-13 | protein_coding |
| ENSMUSG00000035493 | Tgfbi | transforming growth factor, beta induced [Source:MGI Symbol;Acc:MGI:99959] | 99,23174753 | 1,96966203 | **3,91676354** | 6,6326766 | 208,789395 | 2,52342E-47 | 1,02684E-44 | protein_coding |
| ENSMUSG00000039813 | Tbc1d2 | TBC1 domain family, member 2 [Source:MGI Symbol;Acc:MGI:2652885] | 7,064528151 | 1,96822179 | **3,9128554** | 2,81668821 | 37,0674732 | 1,14111E-09 | 2,12859E-08 | protein_coding |
| ENSMUSG00000020826 | Nos2 | nitric oxide synthase 2, inducible [Source:MGI Symbol;Acc:MGI:97361] | 7,947165721 | 1,96678794 | **3,90896844** | 3,00374913 | 44,2405561 | 2,90402E-11 | 7,02786E-10 | protein_coding |
| ENSMUSG00000026749 | Nek6 | NIMA (never in mitosis gene a)-related expressed kinase 6 [Source:MGI Symbol;Acc:MGI:1891638] | 28,595093 | 1,96518997 | **3,90464115** | 4,840522 | 109,497962 | 1,26234E-25 | 1,73367E-23 | protein_coding |
| ENSMUSG00000072812 | Ahnak2 | AHNAK nucleoprotein 2 [Source:MGI Symbol;Acc:MGI:2144831] | 230,1022444 | 1,95786428 | **3,88486451** | 7,84613379 | 53,1971982 | 3,01689E-13 | 9,66372E-12 | protein_coding |
| ENSMUSG00000030084 | Plxna1 | plexin A1 [Source:MGI Symbol;Acc:MGI:107685] | 255,7138625 | 1,9517608 | **3,86846387** | 7,99865949 | 69,0597767 | 9,55238E-17 | 4,97403E-15 | protein_coding |
| ENSMUSG00000028780 | Sema3c | sema domain, immunoglobulin domain (Ig), short basic domain, secreted, (semaphorin) 3C [Source:MGI Symbol;Acc:MC | 2,654177304 | 1,95102484 | **3,86649096** | 1,44268182 | 16,8582418 | 4,0278E-05 | 0,000301866 | protein_coding |
| ENSMUSG00000008734 | Gprc5b | G protein-coupled receptor, family C, group 5, member B [Source:MGI Symbol;Acc:MGI:1927596] | 5,665382164 | 1,94555287 | **3,85185361** | 2,50430385 | 20,3938605 | 6,30317E-06 | 5,74713E-05 | protein_coding |
| ENSMUSG00000038963 | Slco4a1 | solute carrier organic anion transporter family, member 4a1 [Source:MGI Symbol;Acc:MGI:1351866] | 98,63270409 | 1,94367784 | **3,84685069** | 6,6227927 | 176,87993 | 2,32643E-40 | 7,98766E-38 | protein_coding |
| ENSMUSG00000046768 | Rhoj | ras homolog gene family, member J [Source:MGI Symbol;Acc:MGI:1931551] | 5,63979885 | 1,94172187 | **3,84163879** | 2,50346396 | 21,964213 | 2,77782E-06 | 2,74302E-05 | protein_coding |
| ENSMUSG00000045110 | Rassf8 | Ras association (RalGDS/AF-6) domain family (N-terminal) member 8 [Source:MGI Symbol;Acc:MGI:1918573] | 2,486812851 | 1,94095516 | **3,8395977** | 1,30493779 | 14,9449926 | 0,000110691 | 0,000745406 | protein_coding |
| ENSMUSG00000025429 | Pstpip2 | proline-serine-threonine phosphatase-interacting protein 2 [Source:MGI Symbol;Acc:MGI:1335088] | 17,54215221 | 1,94054626 | **3,8385096** | 4,14658179 | 108,598408 | 1,98734E-25 | 2,6628E-23 | protein_coding |
| ENSMUSG00000028927 | Padi2 | peptidyl arginine deiminase, type II [Source:MGI Symbol;Acc:MGI:1338892] | 4,860279534 | 1,94017784 | **3,83752949** | 2,28749154 | 32,4937664 | 1,19575E-08 | 1,87951E-07 | protein_coding |
| ENSMUSG00000031934 | Panx1 | pannexin 1 [Source:MGI Symbol;Acc:MGI:1860055] | 18,28150715 | 1,93759907 | **3,83067615** | 4,20209166 | 40,1655436 | 2,33328E-10 | 4,93944E-09 | protein_coding |
| ENSMUSG00000045455 | Gm9797 | predicted pseudogene 9797 [Source:MGI Symbol;Acc:MGI:3704349] | 4,950208678 | 1,9311895 | **3,81369508** | 2,32385652 | 23,623818 | 1,17129E-06 | 1,25428E-05 | protein_coding |
| ENSMUSG00000022106 | Rcbtb2 | regulator of chromosome condensation (RCC1) and BTB (POZ) domain containing protein 2 [Source:MGI Symbol;Acc:M | 65,46191142 | 1,92812636 | **3,8056064** | 6,03559699 | 123,871968 | 8,98586E-29 | 1,54262E-26 | protein_coding |
| ENSMUSG00000020334 | Slc22a4 | solute carrier family 22 (organic cation transporter), member 4 [Source:MGI Symbol;Acc:MGI:1353479] | 13,20894527 | 1,92678787 | **3,80207731** | 3,72825308 | 44,9779806 | 1,99263E-11 | 4,94198E-10 | protein_coding |
| ENSMUSG00000009376 | Met | met proto-oncogene [Source:MGI Symbol;Acc:MGI:96969] | 42,66128621 | 1,90767519 | **3,75203994** | 5,41786383 | 73,2623396 | 1,13513E-17 | 6,48687E-16 | protein_coding |
| ENSMUSG00000053101 | Gpr141 | G protein-coupled receptor 141 [Source:MGI Symbol;Acc:MGI:2672983] | 10,83692794 | 1,90179913 | **3,73678907** | 3,45129625 | 64,3442496 | 1,04473E-15 | 4,88444E-14 | protein_coding |

| | | | | | | | | | | |
|---|---|---|---|---|---|---|---|---|---|---|
| ENSMUSG00000027533 | Fabp5 | fatty acid binding protein 5, epidermal [Source:MGI Symbol;Acc:MGI:101790] | 239,4400251 | 1,8975681 | **3,72584615** | 7,90414229 | 89,1393211 | 3,67964E-21 | 3,28685E-19 | protein_coding |
| ENSMUSG00000049130 | C5ar1 | complement component 5a receptor 1 [Source:MGI Symbol;Acc:MGI:88232] | 241,5228224 | 1,89656715 | **3,72326203** | 7,91776496 | 104,744148 | 1,3897E-24 | 1,64179E-22 | protein_coding |
| ENSMUSG00000055413 | H2-Q5 | histocompatibility 2, Q region locus 5 [Source:MGI Symbol;Acc:MGI:95934] | 5,148759874 | 1,89160486 | **3,7104775** | 2,36867686 | 31,0273079 | 2,54424E-08 | 3,7724E-07 | pseudogene |
| ENSMUSG00000000058 | Cav2 | caveolin 2 [Source:MGI Symbol;Acc:MGI:107571] | 46,89944247 | 1,88972559 | **3,70564734** | 5,55368921 | 57,3935393 | 3,56777E-14 | 1,31541E-12 | protein_coding |
| ENSMUSG00000043421 | Hilpda | hypoxia inducible lipid droplet associated [Source:MGI Symbol;Acc:MGI:1916823] | 15,78574711 | 1,88954507 | **3,7051837** | 3,988042 | 35,3351958 | 2,7757E-09 | 4,82542E-08 | protein_coding |
| ENSMUSG00000048537 | Phldb1 | pleckstrin homology-like domain, family B, member 1 [Source:MGI Symbol;Acc:MGI:2143230] | 11,63776659 | 1,87916142 | **3,67861175** | 3,55424053 | 59,992857 | 9,52023E-15 | 3,81747E-13 | protein_coding |
| ENSMUSG00000026837 | Col5a1 | collagen, type V, alpha 1 [Source:MGI Symbol;Acc:MGI:88457] | 4,429082912 | 1,87800096 | **3,67565399** | 2,17389917 | 10,257909 | 0,001360999 | 0,006696503 | protein_coding |
| ENSMUSG00000030256 | Bhlhe41 | basic helix-loop-helix family, member e41 [Source:MGI Symbol;Acc:MGI:1930704] | 72,90618952 | 1,87365633 | **3,66460151** | 6,1897811 | 83,9440558 | 5,08976E-20 | 3,77846E-18 | protein_coding |
| ENSMUSG00000028124 | Gclm | glutamate-cysteine ligase, modifier subunit [Source:MGI Symbol;Acc:MGI:104995] | 211,0971202 | 1,86956605 | **3,65422648** | 7,72271337 | 46,6548605 | 8,46567E-12 | 2,20408E-10 | protein_coding |
| ENSMUSG00000028691 | Prdx1 | peroxiredoxin 1 [Source:MGI Symbol;Acc:MGI:99523] | 2395,16309 | 1,86849417 | **3,65151249** | 11,2259703 | 39,8643605 | 2,72225E-10 | 5,7079E-09 | protein_coding |
| ENSMUSG00000040624 | Plekhg1 | pleckstrin homology domain containing, family G (with RhoGef domain) member 1 [Source:MGI Symbol;Acc:MGI:26765 | 10,7954203 | 1,86763847 | **3,64934733** | 3,44602148 | 51,5917867 | 6,83268E-13 | 2,07378E-11 | protein_coding |
| ENSMUSG00000094114 | | Transmembrane protein C5orf28 homolog [Source:UniProtKB/Swiss-Prot;Acc:Q8VDR5] | 5,043355579 | 1,86483731 | **3,64226858** | 2,35938731 | 36,8279818 | 1,29025E-09 | 2,38252E-08 | protein_coding |
| ENSMUSG00000022505 | Emp2 | epithelial membrane protein 2 [Source:MGI Symbol;Acc:MGI:1098726] | 14,16390225 | 1,86457186 | **3,64159847** | 3,83834248 | 47,5155727 | 5,45687E-12 | 1,45169E-10 | protein_coding |
| ENSMUSG00000031838 | Ifi30 | interferon gamma inducible protein 30 [Source:MGI Symbol;Acc:MGI:2137648] | 51,51004396 | 1,86332612 | **3,63845538** | 5,69243278 | 80,548717 | 2,83626E-19 | 1,95987E-17 | protein_coding |
| ENSMUSG00000005413 | Hmox1 | heme oxygenase (decycling) 1 [Source:MGI Symbol;Acc:MGI:96163] | 973,4158477 | 1,85550379 | **3,61878098** | 9,92709604 | 35,5877252 | 2,43819E-09 | 4,31375E-08 | protein_coding |
| ENSMUSG00000026880 | Stom | stomatin [Source:MGI Symbol;Acc:MGI:95403] | 122,6978969 | 1,8484998 | **3,6012551** | 6,94091421 | 104,485897 | 1,58316E-24 | 1,83097E-22 | protein_coding |
| ENSMUSG00000017390 | Aldoc | aldolase C, fructose-bisphosphate [Source:MGI Symbol;Acc:MGI:101863] | 4,59197091 | 1,84583893 | **3,59461917** | 2,21351207 | 32,1737272 | 1,40984E-08 | 2,19094E-07 | protein_coding |
| ENSMUSG00000025757 | Hspa4l | heat shock protein 4 like [Source:MGI Symbol;Acc:MGI:107422] | 30,96414595 | 1,84453373 | **3,59136858** | 4,96148369 | 75,7628391 | 3,19865E-18 | 1,91109E-16 | protein_coding |
| ENSMUSG00000025586 | Cpeb1 | cytoplasmic polyadenylation element binding protein 1 [Source:MGI Symbol;Acc:MGI:108442] | 8,838287569 | 1,83938789 | **3,57858164** | 3,1462858 | 32,136199 | 1,43734E-08 | 2,22737E-07 | protein_coding |
| ENSMUSG00000053040 | Aph1c | anterior pharynx defective 1c homolog (C. elegans) [Source:MGI Symbol;Acc:MGI:1915568] | 46,10540502 | 1,83921771 | **3,57815952** | 5,52895613 | 55,2610709 | 1,05538E-13 | 3,63495E-12 | protein_coding |
| ENSMUSG00000029759 | Pon3 | paraoxonase 3 [Source:MGI Symbol;Acc:MGI:106686] | 28,56684444 | 1,83188001 | **3,56000684** | 4,84237934 | 53,0021345 | 3,33186E-13 | 1,06416E-11 | protein_coding |
| ENSMUSG00000073910 | Mob3b | MOB kinase activator 3B [Source:MGI Symbol;Acc:MGI:2664539] | 2,521927105 | 1,83044013 | **3,55645554** | 1,3524427 | 19,9165658 | 8,08963E-06 | 7,18519E-05 | protein_coding |
| ENSMUSG00000041695 | Kcnj2 | potassium inwardly-rectifying channel, subfamily J, member 2 [Source:MGI Symbol;Acc:MGI:104744] | 37,070892 | 1,82958282 | **3,55434276** | 5,21459276 | 70,0576144 | 5,75974E-17 | 3,05711E-15 | protein_coding |
| ENSMUSG00000034168 | Irf2bpl | interferon regulatory factor 2 binding protein-like [Source:MGI Symbol;Acc:MGI:2442463] | 53,3557618 | 1,82428045 | **3,54130339** | 5,74138051 | 111,39838 | 4,83989E-26 | 6,81741E-24 | protein_coding |
| ENSMUSG00000044103 | Il1f9 | interleukin 1 family, member 9 [Source:MGI Symbol;Acc:MGI:2449929] | 4,291901095 | 1,82387108 | **3,54029867** | 2,11645632 | 22,6034043 | 1,99131E-06 | 2,02955E-05 | protein_coding |
| ENSMUSG00000048807 | Slc35e4 | solute carrier family 35, member E4 [Source:MGI Symbol;Acc:MGI:2144150] | 29,45025947 | 1,82122712 | **3,53381649** | 4,88673417 | 87,1834799 | 9,89065E-21 | 8,04952E-19 | protein_coding |
| ENSMUSG00000018752 | BC096441 | cDNA sequence BC096441 [Source:MGI Symbol;Acc:MGI:3845075] | 3,466441531 | 1,81757176 | **3,52487417** | 1,81495381 | 16,2584615 | 5,52622E-05 | 0,000403433 | protein_coding |
| ENSMUSG00000048120 | Entpd1 | ectonucleoside triphosphate diphosphohydrolase 1 [Source:MGI Symbol;Acc:MGI:102805] | 25,52743394 | 1,81695379 | **3,52336465** | 4,67756521 | 105,667993 | 8,71843E-25 | 1,05263E-22 | protein_coding |
| ENSMUSG00000021483 | Cdk20 | cyclin-dependent kinase 20 [Source:MGI Symbol;Acc:MGI:2145349] | 19,80812061 | 1,81551964 | **3,51986388** | 4,30819289 | 85,4005491 | 2,43653E-20 | 1,87204E-18 | protein_coding |
| ENSMUSG00000033863 | Klf9 | Kruppel-like factor 9 [Source:MGI Symbol;Acc:MGI:1333856] | 5,966937791 | 1,80629024 | **3,49741801** | 2,5924263 | 37,5984713 | 8,69126E-10 | 1,65495E-08 | protein_coding |
| ENSMUSG00000050957 | Insl6 | insulin-like 6 [Source:MGI Symbol;Acc:MGI:1351595] | 4,147722252 | 1,80211527 | **3,48731157** | 2,06122686 | 20,7098183 | 5,34413E-06 | 4,95493E-05 | protein_coding |
| ENSMUSG00000069515 | Lyz1 | lysozyme 1 [Source:MGI Symbol;Acc:MGI:96902] | 3,628607283 | 1,79518993 | **3,47061162** | 1,88156781 | 25,2441714 | 5,0512E-07 | 5,78099E-06 | protein_coding |
| ENSMUSG00000032691 | Nlrp3 | NLR family, pyrin domain containing 3 [Source:MGI Symbol;Acc:MGI:2653833] | 15,62235633 | 1,7928265 | **3,46493071** | 3,99881777 | 69,6788116 | 6,97918E-17 | 3,66891E-15 | protein_coding |
| ENSMUSG00000016382 | Pls3 | plastin 3 (T-isoform) [Source:MGI Symbol;Acc:MGI:104807] | 26,23267654 | 1,79229022 | **3,46364295** | 4,71823169 | 51,1352405 | 8,62165E-13 | 2,58109E-11 | protein_coding |
| ENSMUSG00000021996 | Esd | esterase D/formylglutathione hydrolase [Source:MGI Symbol;Acc:MGI:95421] | 377,4837656 | 1,79026846 | **3,45879248** | 8,56087551 | 60,0741069 | 9,13522E-15 | 3,67651E-13 | protein_coding |
| ENSMUSG00000028378 | Ptgr1 | prostaglandin reductase 1 [Source:MGI Symbol;Acc:MGI:1914353] | 35,92896056 | 1,79007938 | **3,45833921** | 5,17368157 | 63,0583034 | 2,00678E-15 | 9,03625E-14 | protein_coding |
| ENSMUSG00000022951 | Rcan1 | regulator of calcineurin 1 [Source:MGI Symbol;Acc:MGI:1890564] | 51,82424597 | 1,78786714 | **3,45304023** | 5,70372455 | 144,147638 | 3,29847E-33 | 7,71069E-31 | protein_coding |
| ENSMUSG00000024066 | Xdh | xanthine dehydrogenase [Source:MGI Symbol;Acc:MGI:98973] | 165,2571599 | 1,78382804 | **3,4433863** | 7,36950809 | 58,983288 | 1,59017E-14 | 6,1088E-13 | protein_coding |
| ENSMUSG00000075359 | | Uncharacterized protein [Source:UniProtKB/TrEMBL;Acc:J3KMH2] | 2,130962515 | 1,77939779 | **3,43282852** | 1,14673433 | 9,8005766 | 0,001744572 | 0,008373791 | protein_coding |
| ENSMUSG00000089774 | Slc5a3 | solute carrier family 5 (inositol transporters), member 3 [Source:MGI Symbol;Acc:MGI:1858226] | 15,00096633 | 1,77767264 | **3,42872606** | 3,91421981 | 48,6451067 | 3,06733E-12 | 8,61911E-11 | protein_coding |
| ENSMUSG00000029915 | Clec5a | C-type lectin domain family 5, member a [Source:MGI Symbol;Acc:MGI:1345151] | 8,488567965 | 1,77620542 | **3,42524081** | 3,0943856 | 34,8033281 | 3,64749E-09 | 6,22283E-08 | protein_coding |
| ENSMUSG00000029371 | Cxcl5 | chemokine (C-X-C motif) ligand 5 [Source:MGI Symbol;Acc:MGI:1096868] | 7,728180414 | 1,7757749 | **3,42421883** | 2,99750303 | 38,3785648 | 5,82689E-10 | 1,13915E-08 | protein_coding |
| ENSMUSG00000026656 | Fcgr2b | Fc receptor, IgG, low affinity IIb [Source:MGI Symbol;Acc:MGI:95499] | 16,69103267 | 1,77512547 | **3,42267775** | 4,07757092 | 83,2198497 | 7,34168E-20 | 5,37754E-18 | protein_coding |
| ENSMUSG00000003363 | Pld3 | phospholipase D family, member 3 [Source:MGI Symbol;Acc:MGI:1333782] | 310,5752413 | 1,7716961 | **3,41455152** | 8,27911953 | 59,7228235 | 1,09203E-14 | 4,33145E-13 | protein_coding |
| ENSMUSG00000020099 | Unc5b | unc-5 homolog B (C. elegans) [Source:MGI Symbol;Acc:MGI:894703] | 7,211170134 | 1,76568715 | **3,40035919** | 2,86231709 | 25,0744163 | 5,51599E-07 | 6,24785E-06 | protein_coding |
| ENSMUSG00000048445 | Ccdc57 | coiled-coil domain containing 57 [Source:MGI Symbol;Acc:MGI:1918526] | 3,737535604 | 1,76141231 | **3,39029851** | 1,92127543 | 22,9069476 | 1,70036E-06 | 1,75912E-05 | protein_coding |
| ENSMUSG00000018800 | Abca5 | ATP-binding cassette, sub-family A (ABC1), member 5 [Source:MGI Symbol;Acc:MGI:2386607] | 3,889696944 | 1,75304246 | **3,37068652** | 1,96594163 | 21,1132469 | 4,32925E-06 | 4,11163E-05 | protein_coding |
| ENSMUSG00000011256 | Adam19 | a disintegrin and metallopeptidase domain 19 (meltrin beta) [Source:MGI Symbol;Acc:MGI:105377] | 3,570130151 | 1,75052627 | **3,36481286** | 1,8469602 | 21,3418895 | 3,84243E-06 | 3,68506E-05 | protein_coding |
| ENSMUSG00000022534 | Mefv | Mediterranean fever [Source:MGI Symbol;Acc:MGI:1859396] | 18,226388 | 1,74995329 | **3,36347677** | 4,20715604 | 104,165996 | 1,86056E-24 | 2,12938E-22 | protein_coding |
| ENSMUSG00000053687 | Dpep2 | dipeptidase 2 [Source:MGI Symbol;Acc:MGI:2442042] | 35,54021038 | 1,74266215 | **3,34652118** | 5,1577148 | 94,7622931 | 2,14676E-22 | 2,10593E-20 | protein_coding |
| ENSMUSG00000000730 | Dnmt3l | DNA (cytosine-5-)-methyltransferase 3-like [Source:MGI Symbol;Acc:MGI:1859287] | 2,606983571 | 1,73972709 | **3,33971985** | 1,40973469 | 11,2877708 | 0,000780193 | 0,004147064 | protein_coding |
| ENSMUSG00000035273 | Hpse | heparanase [Source:MGI Symbol;Acc:MGI:1343124] | 22,00120763 | 1,73753278 | **3,33464407** | 4,46785999 | 54,9534363 | 1,23419E-13 | 4,17234E-12 | protein_coding |
| ENSMUSG00000021280 | Exoc3l4 | exocyst complex component 3-like 4 [Source:MGI Symbol;Acc:MGI:1921363] | 4,791465608 | 1,73424304 | **3,32704883** | 2,27472529 | 30,1509859 | 3,99684E-08 | 5,71787E-07 | protein_coding |
| ENSMUSG00000026915 | Strbp | spermatid perinuclear RNA binding protein [Source:MGI Symbol;Acc:MGI:104626] | 2,390125687 | 1,72940862 | **3,31591867** | 1,28249498 | 18,4116765 | 1,77964E-05 | 0,000146574 | protein_coding |
| ENSMUSG00000029135 | Fosl2 | fos-like antigen 2 [Source:MGI Symbol;Acc:MGI:102858] | 42,13387543 | 1,72897436 | **3,3149207** | 5,40498148 | 77,424502 | 1,37894E-18 | 8,80838E-17 | protein_coding |
| ENSMUSG00000059498 | Fcgr3 | Fc receptor, IgG, low affinity III [Source:MGI Symbol;Acc:MGI:95500] | 107,5981252 | 1,72845948 | **3,31373785** | 6,75717292 | 174,678363 | 7,03792E-40 | 2,3432E-37 | protein_coding |
| ENSMUSG00000067719 | Gm10221 | predicted gene 10221 [Source:MGI Symbol;Acc:MGI:3708709] | 10,84251224 | 1,72746452 | **3,31145331** | 3,46606415 | 13,880511 | 0,000194808 | 0,001226565 | protein_coding |
| ENSMUSG00000042978 | Sbk1 | SH3-binding kinase 1 [Source:MGI Symbol;Acc:MGI:2135937] | 4,03896357 | 1,72241073 | **3,29987352** | 2,01260928 | 23,1516746 | 1,49713E-06 | 1,56359E-05 | protein_coding |
| ENSMUSG00000042476 | Abcb4 | ATP-binding cassette, sub-family B (MDR/TAP), member 4 [Source:MGI Symbol;Acc:MGI:97569] | 20,07350696 | 1,7204553 | **3,29540389** | 4,3319463 | 56,1369112 | 6,75961E-14 | 2,38803E-12 | protein_coding |
| ENSMUSG00000026712 | Mrc1 | mannose receptor, C type 1 [Source:MGI Symbol;Acc:MGI:97142] | 60,79309087 | 1,71831957 | **3,29052909** | 5,93187745 | 112,736385 | 2,46458E-26 | 3,56294E-24 | protein_coding |
| ENSMUSG00000026042 | Col5a2 | collagen, type V, alpha 2 [Source:MGI Symbol;Acc:MGI:88458] | 7,855650579 | 1,71559453 | **3,28431962** | 2,99571881 | 11,4786139 | 0,000704016 | 0,003786111 | protein_coding |
| ENSMUSG00000027956 | Tmem144 | transmembrane protein 144 [Source:MGI Symbol;Acc:MGI:1917902] | 4,106736199 | 1,71297633 | **3,27836464** | 2,0531098 | 23,852302 | 1,04017E-06 | 1,12706E-05 | protein_coding |
| ENSMUSG00000020658 | Efr3b | EFR3 homolog B (S. cerevisiae) [Source:MGI Symbol;Acc:MGI:2444851] | 3,318170488 | 1,71276217 | **3,27787801** | 1,72533963 | 20,4765604 | 6,0366E-06 | 5,52701E-05 | protein_coding |
| ENSMUSG00000016239 | Lonrf3 | LON peptidase N-terminal domain and ring finger 3 [Source:MGI Symbol;Acc:MGI:1921615] | 48,92300788 | 1,71135125 | **3,2746739** | 5,61552507 | 72,6173207 | 1,57392E-17 | 8,82277E-16 | protein_coding |
| ENSMUSG00000037788 | Vopp1 | vesicular, overexpressed in cancer, prosurvival protein 1 [Source:MGI Symbol;Acc:MGI:2141658] | 27,29715304 | 1,7096245 | **3,27075682** | 4,77841442 | 71,1439752 | 3,32085E-17 | 1,78854E-15 | protein_coding |
| ENSMUSG00000022972 | 1110004E09Rik | RIKEN cDNA 1110004E09 gene [Source:MGI Symbol;Acc:MGI:1915251] | 4,009931448 | 1,70948441 | **3,27043924** | 2,02227258 | 17,9871938 | 2,22396E-05 | 0,000178877 | protein_coding |
| ENSMUSG00000036564 | Ndrg4 | N-myc downstream regulated gene 4 [Source:MGI Symbol;Acc:MGI:2384590] | 6,046510844 | 1,70363597 | **3,25720827** | 2,59944851 | 18,2977707 | 1,88928E-05 | 0,000153988 | protein_coding |
| ENSMUSG00000047592 | Nxpe5 | neurexophilin and PC-esterase domain family, member 5 [Source:MGI Symbol;Acc:MGI:3584036] | 11,58699163 | 1,69940774 | **3,24767606** | 3,55549888 | 56,7808067 | 4,87198E-14 | 1,77247E-12 | protein_coding |
| ENSMUSG00000041488 | Stx3 | syntaxin 3 [Source:MGI Symbol;Acc:MGI:103077] | 22,78528249 | 1,6988986 | **3,24653013** | 4,51471271 | 62,1961816 | 3,1089E-15 | 1,36086E-13 | protein_coding |
| ENSMUSG00000003484 | Cyp4f18 | cytochrome P450, family 4, subfamily f, polypeptide 18 [Source:MGI Symbol;Acc:MGI:1919304] | 1,841674604 | 1,69364892 | **3,23473812** | 0,87225351 | 15,1648342 | 9,85212E-05 | 0,000671913 | protein_coding |
| ENSMUSG00000058755 | Osm | oncostatin M [Source:MGI Symbol;Acc:MGI:104749] | 9,379213249 | 1,68404933 | **3,21328587** | 3,23725104 | 44,6963485 | 2,30086E-11 | 5,63019E-10 | protein_coding |

| | | | | | | | | | | | |
|---|---|---|---|---|---|---|---|---|---|---|---|
| ENSMUSG00000028602 | Tnfrsf8 | tumor necrosis factor receptor superfamily, member 8 [Source:MGI Symbol;Acc:MGI:99908] | 1,562215092 | 1,68018286 | **3,20468568** | 0,6594817 | 13,0723423 | 0,000299688 | 0,001786584 | protein_coding |
| ENSMUSG00000056737 | Capg | capping protein (actin filament), gelsolin-like [Source:MGI Symbol;Acc:MGI:1098259] | 551,4059992 | 1,67615405 | **3,19574889** | 9,10729974 | 42,3109618 | 7,7855E-11 | 1,76007E-09 | protein_coding |
| ENSMUSG00000037411 | Serpine1 | serine (or cysteine) peptidase inhibitor, clade E, member 1 [Source:MGI Symbol;Acc:MGI:97608] | 9,453488684 | 1,67499387 | **3,19317997** | 3,26191769 | 15,8881751 | 6,71973E-05 | 0,000479413 | protein_coding |
| ENSMUSG00000047250 | Ptgs1 | prostaglandin-endoperoxide synthase 1 [Source:MGI Symbol;Acc:MGI:97797] | 13,27683317 | 1,67271562 | **3,1881414** | 3,73819109 | 51,1134776 | 8,71777E-13 | 2,60277E-11 | protein_coding |
| ENSMUSG00000074874 | Ctla2b | cytotoxic T lymphocyte-associated protein 2 beta [Source:MGI Symbol;Acc:MGI:88555] | 46,456143 | 1,67173901 | **3,18598398** | 5,53969316 | 76,4572065 | 2,25037E-18 | 1,38903E-16 | protein_coding |
| ENSMUSG00000060214 | Gm8203 | predicted pseudogene 8203 [Source:MGI Symbol;Acc:MGI:3646499] | 2,708024668 | 1,67072153 | **3,1837378** | 1,48818752 | 7,49343382 | 0,006192436 | 0,024264015 | pseudogene |
| ENSMUSG00000017057 | Il13ra1 | interleukin 13 receptor, alpha 1 [Source:MGI Symbol;Acc:MGI:105052] | 3,378459294 | 1,67027585 | **3,18275443** | 1,79787489 | 15,4373012 | 8,52881E-05 | 0,000593828 | protein_coding |
| ENSMUSG00000024900 | Cpt1a | carnitine palmitoyltransferase 1a, liver [Source:MGI Symbol;Acc:MGI:1098296] | 87,10565445 | 1,66795384 | **3,17763594** | 6,4467694 | 38,032942 | 6,95602E-10 | 1,3408E-08 | protein_coding |
| ENSMUSG00000002992 | Apoc2 | apolipoprotein C-II [Source:MGI Symbol;Acc:MGI:88054] | 4,158399306 | 1,66588288 | **3,17307776** | 2,06337345 | 25,7132337 | 3,96102E-07 | 4,59068E-06 | protein_coding |
| ENSMUSG00000026728 | Vim | vimentin [Source:MGI Symbol;Acc:MGI:98932] | 1111,286416 | 1,6639325 | **3,16879098** | 10,1181469 | 42,7039586 | 6,36833E-11 | 1,46993E-09 | protein_coding |
| ENSMUSG00000028278 | Rragd | Ras-related GTP binding D [Source:MGI Symbol;Acc:MGI:1098604] | 13,88139216 | 1,65803298 | **3,15585951** | 3,80114497 | 39,6958349 | 2,96758E-10 | 6,17515E-09 | protein_coding |
| ENSMUSG00000019558 | Slc6a8 | solute carrier family 6 (neurotransmitter transporter, creatine), member 8 [Source:MGI Symbol;Acc:MGI:2147834] | 144,8446925 | 1,65773553 | **3,1552089** | 7,17896943 | 47,8656 | 4,56461E-12 | 1,22023E-10 | protein_coding |
| ENSMUSG00000015947 | Fcgr1 | Fc receptor, IgG, high affinity I [Source:MGI Symbol;Acc:MGI:95498] | 150,2696856 | 1,65487791 | **3,14896541** | 7,23482201 | 110,814407 | 6,49778E-26 | 9,03684E-24 | protein_coding |
| ENSMUSG00000037664 | Cdkn1c | cyclin-dependent kinase inhibitor 1C (P57) [Source:MGI Symbol;Acc:MGI:104564] | 4,851079305 | 1,64781052 | **3,13357716** | 2,27373125 | 19,1756995 | 1,19221E-05 | 0,000101585 | protein_coding |
| ENSMUSG00000022565 | Plec | plectin [Source:MGI Symbol;Acc:MGI:1277961] | 522,4314524 | 1,64655015 | **3,1308408** | 9,02920338 | 54,1365811 | 1,87027E-13 | 6,20805E-12 | protein_coding |
| ENSMUSG00000029994 | Anxa4 | annexin A4 [Source:MGI Symbol;Acc:MGI:88030] | 203,5912624 | 1,64643878 | **3,13059912** | 7,67023014 | 56,2856708 | 6,26705E-14 | 2,24287E-12 | protein_coding |
| ENSMUSG00000027322 | Siglec1 | sialic acid binding Ig-like lectin 1, sialoadhesin [Source:MGI Symbol;Acc:MGI:99668] | 127,6979298 | 1,64366542 | **3,1245868** | 7,0007799 | 85,8655817 | 1,92592E-20 | 1,49015E-18 | protein_coding |
| ENSMUSG00000054675 | Tmem119 | transmembrane protein 119 [Source:MGI Symbol;Acc:MGI:2385228] | 5,335848509 | 1,64051542 | **3,11777199** | 2,43309926 | 23,5226066 | 1,23455E-06 | 1,30674E-05 | protein_coding |
| ENSMUSG00000050737 | Ptges | prostaglandin E synthase [Source:MGI Symbol;Acc:MGI:1927593] | 6,825056275 | 1,63728408 | **3,11079662** | 2,78438339 | 35,6073927 | 2,4137E-09 | 4,28422E-08 | protein_coding |
| ENSMUSG00000044864 | Ankrd50 | ankyrin repeat domain 50 [Source:MGI Symbol;Acc:MGI:2139777] | 49,8576082 | 1,63628399 | **3,10864093** | 5,64586871 | 88,1793721 | 5,97801E-21 | 5,17168E-19 | protein_coding |
| ENSMUSG00000026202 | Tuba4a | tubulin, alpha 4A [Source:MGI Symbol;Acc:MGI:1095410] | 52,98796295 | 1,63510574 | **3,10610315** | 5,7314497 | 42,1331185 | 8,52668E-11 | 1,91973E-09 | protein_coding |
| ENSMUSG00000070691 | Runx3 | runt related transcription factor 3 [Source:MGI Symbol;Acc:MGI:102672] | 2,007551671 | 1,63496617 | **3,10580268** | 1,04642006 | 10,8919595 | 0,000965826 | 0,004984279 | protein_coding |
| ENSMUSG00000058427 | Cxcl2 | chemokine (C-X-C motif) ligand 2 [Source:MGI Symbol;Acc:MGI:1340094] | 56,73235874 | 1,6280082 | **3,09085976** | 5,84166516 | 118,555291 | 1,3105E-27 | 2,08673E-25 | protein_coding |
| ENSMUSG00000033420 | Antxr1 | anthrax toxin receptor 1 [Source:MGI Symbol;Acc:MGI:1916788] | 2,858525716 | 1,62665931 | **3,08797124** | 1,54638977 | 9,2064997 | 0,002411574 | 0,011012452 | protein_coding |
| ENSMUSG00000038235 | F11r | F11 receptor [Source:MGI Symbol;Acc:MGI:1321398] | 3,51622626 | 1,62211708 | **3,07826424** | 1,8206396 | 18,0804689 | 2,11763E-05 | 0,000171454 | protein_coding |
| ENSMUSG00000028076 | Cd1d1 | CD1d1 antigen [Source:MGI Symbol;Acc:MGI:107674] | 2,873494546 | 1,620623 | **3,07507798** | 1,53795037 | 12,008377 | 0,00052962 | 0,002955272 | protein_coding |
| ENSMUSG00000040907 | Atp1a3 | ATPase, Na+/K+ transporting, alpha 3 polypeptide [Source:MGI Symbol;Acc:MGI:88107] | 95,5064395 | 1,62031928 | **3,07443068** | 6,57739422 | 65,7387179 | 5,14844E-16 | 2,51404E-14 | protein_coding |
| ENSMUSG00000018927 | Ccl6 | chemokine (C-C motif) ligand 6 [Source:MGI Symbol;Acc:MGI:98263] | 31,62991118 | 1,61995083 | **3,07364561** | 5,00039651 | 115,157976 | 7,26714E-27 | 1,09375E-24 | protein_coding |
| ENSMUSG00000036103 | Colec12 | collectin sub-family member 12 [Source:MGI Symbol;Acc:MGI:2152907] | 15,61823629 | 1,61978988 | **3,07330272** | 3,97263009 | 30,663925 | 3,06817E-08 | 4,51271E-07 | protein_coding |
| ENSMUSG00000020234 | 4930404N11Rik | RIKEN cDNA 4930404N11 gene [Source:MGI Symbol;Acc:MGI:1921072] | 8,060326497 | 1,61068687 | **3,05397208** | 3,00237638 | 32,6642986 | 1,09531E-08 | 1,73153E-07 | protein_coding |
| ENSMUSG00000037852 | Cpe | carboxypeptidase E [Source:MGI Symbol;Acc:MGI:101932] | 3,338569998 | 1,6058583 | **3,04376779** | 1,76327583 | 7,23657178 | 0,007143325 | 0,027365309 | protein_coding |
| ENSMUSG00000044468 | Fam46c | family with sequence similarity 46, member C [Source:MGI Symbol;Acc:MGI:1921895] | 14,21994648 | 1,60262292 | **3,03694949** | 3,83623669 | 36,4166114 | 1,59339E-09 | 2,90808E-08 | protein_coding |
| ENSMUSG00000048924 | Ccdc125 | coiled-coil domain containing 125 [Source:MGI Symbol;Acc:MGI:1923291] | 5,768882228 | 1,60191123 | **3,03545173** | 2,53963491 | 28,684709 | 8,51736E-08 | 1,13178E-06 | protein_coding |
| ENSMUSG00000021846 | Peli2 | pellino 2 [Source:MGI Symbol;Acc:MGI:1891445] | 3,587153757 | 1,60051672 | **3,03251907** | 1,8619679 | 20,101591 | 7,34356E-06 | 6,57033E-05 | protein_coding |
| ENSMUSG00000038147 | Cd84 | CD84 antigen [Source:MGI Symbol;Acc:MGI:1336885] | 223,5997657 | 1,60004416 | **3,03152593** | 7,80549016 | 66,0331398 | 4,43405E-16 | 2,19445E-14 | protein_coding |
| ENSMUSG00000075602 | Ly6a | lymphocyte antigen 6 complex, locus A [Source:MGI Symbol;Acc:MGI:107527] | 5,684332863 | 1,59931864 | **3,03000179** | 2,51866056 | 18,3818497 | 1,80772E-05 | 0,00014833 | protein_coding |
| ENSMUSG00000018774 | Cd68 | CD68 antigen [Source:MGI Symbol;Acc:MGI:88342] | 432,6864511 | 1,59591625 | **3,02286437** | 8,75747773 | 32,7956278 | 1,02375E-08 | 1,62777E-07 | protein_coding |
| ENSMUSG00000031488 | Rab11fip1 | RAB11 family interacting protein 1 (class I) [Source:MGI Symbol;Acc:MGI:1923017] | 22,46578284 | 1,59404051 | **3,0189367** | 4,50586685 | 74,9212672 | 4,89865E-18 | 2,87815E-16 | protein_coding |
| ENSMUSG00000024186 | Rgs11 | regulator of G-protein signaling 11 [Source:MGI Symbol;Acc:MGI:1354739] | 2,799414823 | 1,59276426 | **3,01626724** | 1,50313934 | 17,3139846 | 3,16846E-05 | 0,000244222 | protein_coding |
| ENSMUSG00000072825 | AW555464 | expressed sequence AW555464 [Source:MGI Symbol;Acc:MGI:2145043] | 26,23555426 | 1,59011008 | **3,01072322** | 4,71446802 | 38,3695758 | 5,85379E-10 | 1,14037E-08 | protein_coding |
| ENSMUSG00000037172 | E330009J07Rik | RIKEN cDNA E330009J07 gene [Source:MGI Symbol;Acc:MGI:2444256] | 4,234284124 | 1,59003408 | **3,01056462** | 2,10054531 | 20,5890263 | 5,69215E-06 | 5,24221E-05 | protein_coding |
| ENSMUSG00000038712 | Fam63a | family with sequence similarity 63, member A [Source:MGI Symbol;Acc:MGI:1922257] | 69,08549576 | 1,58808463 | **3,00649932** | 6,11285438 | 78,1488508 | 9,55612E-19 | 6,28701E-17 | protein_coding |
| ENSMUSG00000052397 | Ezr | ezrin [Source:MGI Symbol;Acc:MGI:98931] | 22,81512045 | 1,58577303 | **3,00168592** | 4,51860367 | 43,9270116 | 3,40852E-11 | 8,10594E-10 | protein_coding |
| ENSMUSG00000082691 | Dynlt1-ps1 | dynein light chain Tctex-type 1, pseudogene 1 [Source:MGI Symbol;Acc:MGI:3642625] | 3,991080553 | 1,57842804 | **2,98644271** | 1,97523353 | 9,095076 | 0,002562986 | 0,011550256 | pseudogene |
| ENSMUSG00000032491 | Nradd | neurotrophin receptor associated death domain [Source:MGI Symbol;Acc:MGI:1914419] | 3,196143111 | 1,5748954 | **2,97913891** | 1,6831417 | 14,6997215 | 0,000126065 | 0,000836399 | protein_coding |
| ENSMUSG00000085295 | 4930430E12Rik | RIKEN cDNA 4930430E12 gene [Source:MGI Symbol;Acc:MGI:1918889] | 11,7789291 | 1,57415155 | **2,97760327** | 3,56677656 | 45,2845933 | 1,70384E-11 | 4,28378E-10 | antisense |
| ENSMUSG00000022947 | Cbr3 | carbonyl reductase 3 [Source:MGI Symbol;Acc:MGI:1309992] | 17,68316824 | 1,5740333 | **2,97735923** | 4,15558705 | 61,733838 | 3,93165E-15 | 1,66143E-13 | protein_coding |
| ENSMUSG00000032554 | Trf | transferrin [Source:MGI Symbol;Acc:MGI:98821] | 19,7871103 | 1,57384181 | **2,97696408** | 4,30901272 | 71,4209072 | 2,88598E-17 | 1,56972E-15 | protein_coding |
| ENSMUSG00000001918 | Slc1a5 | solute carrier family 1 (neutral amino acid transporter), member 5 [Source:MGI Symbol;Acc:MGI:105305] | 17,3091856 | 1,5712408 | **2,97160179** | 4,11310105 | 79,6544213 | 4,45971E-19 | 3,01997E-17 | protein_coding |
| ENSMUSG00000089672 | Gp49a | glycoprotein 49 A [Source:MGI Symbol;Acc:MGI:102702] | 190,5957936 | 1,56833445 | **2,96562144** | 7,57538127 | 48,423245 | 3,43473E-12 | 9,50563E-11 | protein_coding |
| ENSMUSG00000022122 | Ednrb | endothelin receptor type B [Source:MGI Symbol;Acc:MGI:102720] | 10,5574148 | 1,5680441 | **2,96502465** | 3,43365236 | 36,6142649 | 1,43974E-09 | 2,64081E-08 | protein_coding |
| ENSMUSG00000034758 | Tle6 | transducin-like enhancer of split 6, homolog of Drosophila E(spl) [Source:MGI Symbol;Acc:MGI:2149593] | 5,613083543 | 1,56791278 | **2,96475477** | 2,50868096 | 26,6501609 | 2,4383E-07 | 2,9504E-06 | protein_coding |
| ENSMUSG00000028680 | Plk3 | polo-like kinase 3 [Source:MGI Symbol;Acc:MGI:109604] | 20,33941863 | 1,5650716 | **2,95892187** | 4,35276014 | 48,5576742 | 3,20717E-12 | 8,98907E-11 | protein_coding |
| ENSMUSG00000027646 | Src | Rous sarcoma oncogene [Source:MGI Symbol;Acc:MGI:98397] | 10,06842109 | 1,56483208 | **2,95843066** | 3,33978848 | 23,8941955 | 1,01778E-06 | 1,10931E-05 | protein_coding |
| ENSMUSG00000022756 | Slc7a4 | solute carrier family 7 (cationic amino acid transporter, y+ system), member 4 [Source:MGI Symbol;Acc:MGI:2146512] | 2,95116496 | 1,56190281 | **2,95242992** | 1,58085741 | 15,195612 | 9,69284E-05 | 0,000661872 | protein_coding |
| ENSMUSG00000021298 | Gpr132 | G protein-coupled receptor 132 [Source:MGI Symbol;Acc:MGI:1890220] | 8,775017383 | 1,55697213 | **2,94235665** | 3,13256816 | 43,2765898 | 4,75235E-11 | 1,12288E-09 | protein_coding |
| ENSMUSG00000054342 | Kcnn4 | potassium intermediate/small conductance calcium-activated channel, subfamily N, member 4 [Source:MGI Symbol;Acc: | 80,67446507 | 1,5546554 | **2,93763549** | 6,33451647 | 60,7167277 | 6,59077E-15 | 2,69193E-13 | protein_coding |
| ENSMUSG00000025880 | Smad7 | SMAD family member 7 [Source:MGI Symbol;Acc:MGI:1100518] | 2,171523538 | 1,55382658 | **2,93594834** | 1,17277351 | 13,378717 | 0,000254496 | 0,001549969 | protein_coding |
| ENSMUSG00000038463 | Olfml2b | olfactomedin-like 2B [Source:MGI Symbol;Acc:MGI:2443310] | 2,033897464 | 1,55344587 | **2,93517368** | 1,06950754 | 11,4353762 | 0,000720589 | 0,003867665 | protein_coding |
| ENSMUSG00000074657 | Kif5a | kinesin family member 5A [Source:MGI Symbol;Acc:MGI:109564] | 4,437318359 | 1,55177683 | **2,93177996** | 2,14667159 | 21,9001419 | 2,87213E-06 | 2,83014E-05 | protein_coding |
| ENSMUSG00000056145 | AI504432 | expressed sequence AI504432 [Source:MGI Symbol;Acc:MGI:2139742] | 4,124714333 | 1,54902132 | **2,92618569** | 2,07640771 | 18,0961331 | 2,10028E-05 | 0,000170301 | protein_coding |
| ENSMUSG00000026875 | Traf1 | TNF receptor-associated factor 1 [Source:MGI Symbol;Acc:MGI:101836] | 14,61897125 | 1,5472578 | **2,92261095** | 3,88712101 | 65,4189437 | 6,0554E-16 | 2,93087E-14 | protein_coding |
| ENSMUSG00000026600 | Soat1 | sterol O-acyltransferase 1 [Source:MGI Symbol;Acc:MGI:104665] | 224,6616941 | 1,54526739 | **2,91858157** | 7,8123119 | 56,3297751 | 6,12804E-14 | 2,2075E-12 | protein_coding |
| ENSMUSG00000074305 | C230081A13Rik | RIKEN cDNA C230081A13 gene [Source:MGI Symbol;Acc:MGI:2442366] | 77,68551429 | 1,53887614 | **2,90568062** | 6,28226389 | 64,014364 | 1,23515E-15 | 5,72601E-14 | protein_coding |
| ENSMUSG00000052713 | Zfp608 | zinc finger protein 608 [Source:MGI Symbol;Acc:MGI:2442338] | 3,692061798 | 1,53484178 | **2,89756651** | 1,90455365 | 19,086474 | 1,24927E-05 | 0,00010599 | protein_coding |
| ENSMUSG00000022415 | Syngr1 | synaptogyrin 1 [Source:MGI Symbol;Acc:MGI:1328323] | 25,25058137 | 1,53421684 | **2,89631164** | 4,65955639 | 28,3765131 | 9,98696E-08 | 1,31409E-06 | protein_coding |
| ENSMUSG00000095649 | | Uncharacterized protein [Source:UniProtKB/TrEMBL;Acc:J3QPZ6] | 1,751887725 | 1,53244101 | **2,89274872** | 0,8165183 | 9,58747127 | 0,001959096 | 0,009210349 | protein_coding |
| ENSMUSG00000071286 | Sowahc | sosondowah ankyrin repeat domain family member C [Source:MGI Symbol;Acc:MGI:3606051] | 10,80922955 | 1,5312858 | **2,89043333** | 3,44205009 | 38,8123307 | 4,66571E-10 | 9,37151E-09 | ocessed_pseudoge |
| ENSMUSG00000030577 | Cd22 | CD22 antigen [Source:MGI Symbol;Acc:MGI:88322] | 11,79301811 | 1,52896648 | **2,88579032** | 3,57045463 | 28,7046217 | 8,43023E-08 | 1,1227E-06 | protein_coding |
| ENSMUSG00000081999 | Gm13461 | predicted gene 13461 [Source:MGI Symbol;Acc:MGI:3650103] | 7,165760898 | 1,52414579 | **2,8761637** | 2,87581773 | 11,0244675 | 0,000899171 | 0,004686524 | pseudogene |
| ENSMUSG00000097415 | | | 4,115615639 | 1,52368965 | **2,87525448** | 2,04493471 | 21,731823 | 3,13547E-06 | 3,06762E-05 | lincRNA |

| | | | | | | | | | | |
|---|---|---|---|---|---|---|---|---|---|---|
| ENSMUSG00000025161 | Slc16a3 | solute carrier family 16 (monocarboxylic acid transporters), member 3 [Source:MGI Symbol;Acc:MGI:1933438] | 38,73802616 | 1,51942263 | **2,86676298** | 5,28821147 | 87,9793401 | 6,61417E-21 | 5,58999E-19 | protein_coding |
| ENSMUSG00000015305 | Sash1 | SAM and SH3 domain containing 1 [Source:MGI Symbol;Acc:MGI:1917347] | 212,8696388 | 1,51318117 | **2,85438744** | 7,73500495 | 59,878158 | 1,00915E-14 | 4,03184E-13 | protein_coding |
| ENSMUSG00000026249 | Serpine2 | serine (or cysteine) peptidase inhibitor, clade E, member 2 [Source:MGI Symbol;Acc:MGI:101780] | 3,061946519 | 1,50833094 | **2,84480732** | 1,65696183 | 9,30282856 | 0,002288003 | 0,010513715 | protein_coding |
| ENSMUSG00000021770 | Samd8 | sterile alpha motif domain containing 8 [Source:MGI Symbol;Acc:MGI:1914880] | 64,89327709 | 1,50783842 | **2,8438363** | 6,0223159 | 58,8120921 | 1,73472E-14 | 6,62923E-13 | protein_coding |
| ENSMUSG00000022236 | Ropn1l | ropporin 1-like [Source:MGI Symbol;Acc:MGI:2182357] | 2,408917699 | 1,50766372 | **2,84349195** | 1,27027036 | 14,2910554 | 0,000156607 | 0,001012695 | protein_coding |
| ENSMUSG00000033386 | Frrs1 | ferric-chelate reductase 1 [Source:MGI Symbol;Acc:MGI:108076] | 29,43278816 | 1,50676975 | **2,84173052** | 4,88560851 | 62,0874717 | 3,28535E-15 | 1,42111E-13 | protein_coding |
| ENSMUSG00000003573 | Homer3 | homer homolog 3 (Drosophila) [Source:MGI Symbol;Acc:MGI:1347359] | 30,66674086 | 1,5065639 | **2,84132508** | 4,94265322 | 52,091579 | 5,29716E-13 | 1,63483E-11 | protein_coding |
| ENSMUSG00000062585 | Cnr2 | cannabinoid receptor 2 (macrophage) [Source:MGI Symbol;Acc:MGI:104650] | 7,361083056 | 1,50570222 | **2,83962855** | 2,88163854 | 32,0994165 | 1,46481E-08 | 2,26356E-07 | protein_coding |
| ENSMUSG00000056749 | Nfil3 | nuclear factor, interleukin 3, regulated [Source:MGI Symbol;Acc:MGI:109495] | 11,54099306 | 1,50341808 | **2,83513627** | 3,5480163 | 48,938445 | 2,64123E-12 | 7,47916E-11 | protein_coding |
| ENSMUSG00000002289 | Angptl4 | angiopoietin-like 4 [Source:MGI Symbol;Acc:MGI:1888999] | 1,888841547 | 1,50059978 | **2,82960325** | 0,96049885 | 6,18752608 | 0,012865392 | 0,044520333 | protein_coding |
| ENSMUSG00000050967 | Creg2 | cellular repressor of E1A-stimulated genes 2 [Source:MGI Symbol;Acc:MGI:1928333] | 5,316481149 | 1,49772552 | **2,82397148** | 2,43982325 | 19,7235855 | 8,94901E-06 | 7,87822E-05 | protein_coding |
| ENSMUSG00000040133 | Gpr176 | G protein-coupled receptor 176 [Source:MGI Symbol;Acc:MGI:2685858] | 3,746478682 | 1,49742083 | **2,82337514** | 1,93065107 | 11,6259854 | 0,000650368 | 0,003532175 | protein_coding |
| ENSMUSG00000027398 | Il1b | interleukin 1 beta [Source:MGI Symbol;Acc:MGI:96543] | 14,77165668 | 1,49691427 | **2,82238397** | 3,91541649 | 28,9061002 | 7,59733E-08 | 1,02798E-06 | protein_coding |
| ENSMUSG00000005057 | Sh2b2 | SH2B adaptor protein 2 [Source:MGI Symbol;Acc:MGI:1345171] | 20,06366888 | 1,49341007 | **2,81553693** | 4,32664252 | 56,8771912 | 4,63895E-14 | 1,69329E-12 | protein_coding |
| ENSMUSG00000021575 | Ahrr | aryl-hydrocarbon receptor repressor [Source:MGI Symbol;Acc:MGI:1333776] | 23,20493619 | 1,49105224 | **2,81093919** | 4,5528355 | 82,6830233 | 9,6325E-20 | 6,91714E-18 | protein_coding |
| ENSMUSG00000025492 | Ifitm3 | interferon induced transmembrane protein 3 [Source:MGI Symbol;Acc:MGI:1913391] | 200,5567245 | 1,48863949 | **2,80624213** | 7,64941838 | 77,9197631 | 1,07312E-18 | 6,89497E-17 | protein_coding |
| ENSMUSG00000036478 | Btg1 | B cell translocation gene 1, anti-proliferative [Source:MGI Symbol;Acc:MGI:88215] | 93,54190727 | 1,48487448 | **2,7989282** | 6,551301 | 125,787473 | 3,42241E-29 | 5,96858E-27 | protein_coding |
| ENSMUSG00000006651 | Aplp1 | amyloid beta (A4) precursor-like protein 1 [Source:MGI Symbol;Acc:MGI:88046] | 2,7045129 | 1,47869678 | **2,78696866** | 1,45701577 | 13,6907794 | 0,00021551 | 0,001338501 | protein_coding |
| ENSMUSG00000020250 | Txnrd1 | thioredoxin reductase 1 [Source:MGI Symbol;Acc:MGI:1354175] | 311,3636241 | 1,46824499 | **2,76685108** | 8,2834286 | 51,5638492 | 6,93061E-13 | 2,0977E-11 | protein_coding |
| ENSMUSG00000054484 | Tmem62 | transmembrane protein 62 [Source:MGI Symbol;Acc:MGI:2139461] | 10,0808566 | 1,46741407 | **2,76525795** | 3,35443478 | 34,2028254 | 4,96568E-09 | 8,2789E-08 | protein_coding |
| ENSMUSG00000030745 | Il21r | interleukin 21 receptor [Source:MGI Symbol;Acc:MGI:1890475] | 57,95615914 | 1,4634218 | **2,75761644** | 5,86218802 | 112,024499 | 3,52922E-26 | 5,03579E-24 | protein_coding |
| ENSMUSG00000005447 | Pafah1b3 | platelet-activating factor acetylhydrolase, isoform 1b, subunit 3 [Source:MGI Symbol;Acc:MGI:108414] | 7,918512572 | 1,46298012 | **2,75677232** | 2,98690485 | 28,591408 | 8,93779E-08 | 1,18313E-06 | protein_coding |
| ENSMUSG00000018920 | Cxcl16 | chemokine (C-X-C motif) ligand 16 [Source:MGI Symbol;Acc:MGI:1932682] | 133,8441623 | 1,46216881 | **2,75522247** | 7,06678941 | 77,0519629 | 1,66519E-18 | 1,05754E-16 | protein_coding |
| ENSMUSG00000027848 | Olfml3 | olfactomedin-like 3 [Source:MGI Symbol;Acc:MGI:1914877] | 7,992444055 | 1,46189607 | **2,75470164** | 3,00146919 | 20,1325175 | 7,22577E-06 | 6,48607E-05 | protein_coding |
| ENSMUSG00000034472 | Rasd2 | RASD family, member 2 [Source:MGI Symbol;Acc:MGI:1922391] | 3,322717207 | 1,44969034 | **2,73149415** | 1,73433496 | 13,7415951 | 0,000209757 | 0,001305724 | protein_coding |
| ENSMUSG00000039693 | 5730528L13Rik | RIKEN cDNA 5730528L13 gene [Source:MGI Symbol;Acc:MGI:1913915] | 3,120986304 | 1,44558448 | **2,72373148** | 1,67742581 | 12,6845807 | 0,000368683 | 0,002151207 | protein_coding |
| ENSMUSG00000053318 | Slamf8 | SLAM family member 8 [Source:MGI Symbol;Acc:MGI:1921998] | 10,0959864 | 1,44448236 | **2,72165152** | 3,34516445 | 42,9653447 | 5,57182E-11 | 1,29698E-09 | protein_coding |
| ENSMUSG00000079625 | Tm4sf19 | transmembrane 4 L six family member 19 [Source:MGI Symbol;Acc:MGI:3645933] | 5,762005848 | 1,44112663 | **2,71532829** | 2,52318999 | 19,1466739 | 1,21048E-05 | 0,000103018 | protein_coding |
| ENSMUSG00000022901 | Cd86 | CD86 antigen [Source:MGI Symbol;Acc:MGI:101773] | 21,36254281 | 1,44103957 | **2,71516442** | 4,415332 | 66,9057413 | 2,84804E-16 | 1,4159E-14 | protein_coding |
| ENSMUSG00000033917 | Gde1 | glycerophosphodiester phosphodiesterase 1 [Source:MGI Symbol;Acc:MGI:1891827] | 56,60198587 | 1,43710193 | **2,70776386** | 5,82439042 | 64,5497771 | 9,41242E-16 | 4,43838E-14 | protein_coding |
| ENSMUSG00000041268 | Dmxl2 | Dmx-like 2 [Source:MGI Symbol;Acc:MGI:2444630] | 112,3508794 | 1,43520058 | **2,70419761** | 6,81440301 | 84,5209561 | 3,80164E-20 | 2,88059E-18 | protein_coding |
| ENSMUSG00000030259 | Rassf8 | Ras association (RalGDS/AF-6) domain family (N-terminal) member 8 [Source:MGI Symbol;Acc:MGI:1918573] | 27,14359303 | 1,42365788 | **2,68264821** | 4,76533358 | 48,0649903 | 4,12323E-12 | 1,11857E-10 | protein_coding |
| ENSMUSG00000083696 | Gm12563 | predicted gene 12563 [Source:MGI Symbol;Acc:MGI:3652205] | 5,480768245 | 1,42272194 | **2,68090842** | 2,48624534 | 13,9066082 | 0,000192122 | 0,00121104 | pseudogene |
| ENSMUSG00000040274 | Cdk6 | cyclin-dependent kinase 6 [Source:MGI Symbol;Acc:MGI:1277162] | 17,3110174 | 1,4185954 | **2,67325118** | 4,1166395 | 33,7295288 | 6,3332E-09 | 1,03392E-07 | protein_coding |
| ENSMUSG00000022707 | Gbe1 | glucan (1,4-alpha-), branching enzyme 1 [Source:MGI Symbol;Acc:MGI:1921435] | 45,24202165 | 1,41772849 | **2,67164531** | 5,50533425 | 55,7473107 | 8,24109E-14 | 2,86534E-12 | protein_coding |
| ENSMUSG00000023805 | Synj2 | synaptojanin 2 [Source:MGI Symbol;Acc:MGI:1201671] | 24,00393073 | 1,41710629 | **2,67049334** | 4,59627149 | 54,4919554 | 1,56085E-13 | 5,2125E-12 | protein_coding |
| ENSMUSG00000019806 | Aig1 | androgen-induced 1 [Source:MGI Symbol;Acc:MGI:1913503] | 6,120792455 | 1,41445951 | **2,66559854** | 2,61336692 | 23,6186145 | 1,17446E-06 | 1,25645E-05 | protein_coding |
| ENSMUSG00000035342 | Lzts2 | leucine zipper, putative tumor suppressor 2 [Source:MGI Symbol;Acc:MGI:2385095] | 4,98637444 | 1,41428836 | **2,66528233** | 2,32753902 | 14,5950776 | 0,000133262 | 0,000877789 | protein_coding |
| ENSMUSG00000026826 | Nr4a2 | nuclear receptor subfamily 4, group A, member 2 [Source:MGI Symbol;Acc:MGI:1352456] | 3,289963724 | 1,41160677 | **2,66033287** | 1,72569999 | 16,3304924 | 5,3201E-05 | 0,000389679 | protein_coding |
| ENSMUSG00000032374 | Plod2 | procollagen lysine, 2-oxoglutarate 5-dioxygenase 2 [Source:MGI Symbol;Acc:MGI:1347007] | 2,212001941 | 1,40953925 | **2,65652309** | 1,17983671 | 7,98505209 | 0,004716514 | 0,019264066 | protein_coding |
| ENSMUSG00000001036 | Epn2 | epsin 2 [Source:MGI Symbol;Acc:MGI:1333766] | 7,596568522 | 1,40742913 | **2,65264043** | 2,9402739 | 21,3572863 | 3,81169E-06 | 3,66338E-05 | protein_coding |
| ENSMUSG00000055013 | Agap1 | ArfGAP with GTPase domain, ankyrin repeat and PH domain 1 [Source:MGI Symbol;Acc:MGI:2653690] | 12,37448981 | 1,40325391 | **2,64497467** | 3,6303463 | 29,5968701 | 5,31908E-08 | 7,44467E-07 | protein_coding |
| ENSMUSG00000035640 | Dos | downstream of Stk11 [Source:MGI Symbol;Acc:MGI:1354170] | 1,856324457 | 1,40318431 | **2,64484707** | 0,89332622 | 10,8552906 | 0,000985139 | 0,005074413 | protein_coding |
| ENSMUSG00000014786 | Slc9a5 | solute carrier family 9 (sodium/hydrogen exchanger), member 5 [Source:MGI Symbol;Acc:MGI:2685542] | 2,523807415 | 1,40288417 | **2,64429689** | 1,35954251 | 10,6158918 | 0,001121199 | 0,005658607 | protein_coding |
| ENSMUSG00000031647 | Mfap3l | microfibrillar-associated protein 3-like [Source:MGI Symbol;Acc:MGI:1918556] | 26,80120617 | 1,40137905 | **2,64153963** | 4,74915428 | 33,7916722 | 6,13409E-09 | 1,0059E-07 | protein_coding |
| ENSMUSG00000039153 | Runx2 | runt related transcription factor 2 [Source:MGI Symbol;Acc:MGI:99829] | 5,064722269 | 1,40003766 | **2,63908472** | 2,36174785 | 19,0189453 | 1,29427E-05 | 0,000109723 | protein_coding |
| ENSMUSG00000073492 | Gm10521 | predicted gene 10521 [Source:MGI Symbol;Acc:MGI:3642358] | 12,49939144 | 1,39988101 | **2,63879818** | 3,64494746 | 38,6888727 | 4,97034E-10 | 9,92894E-09 | protein_coding |
| ENSMUSG00000038894 | Irs2 | insulin receptor substrate 2 [Source:MGI Symbol;Acc:MGI:109334] | 4,894620536 | 1,397606 | **2,63464029** | 2,29361899 | 20,0724815 | 7,45618E-06 | 6,66025E-05 | protein_coding |
| ENSMUSG00000033721 | Vav3 | vav 3 oncogene [Source:MGI Symbol;Acc:MGI:1888518] | 44,91707469 | 1,39457074 | **2,62910313** | 5,49303189 | 55,0298261 | 1,18715E-13 | 4,06329E-12 | protein_coding |
| ENSMUSG00000007783 | Cpt1c | carnitine palmitoyltransferase 1c [Source:MGI Symbol;Acc:MGI:2446526] | 1,605841008 | 1,39417037 | **2,62837363** | 0,73225737 | 6,56682204 | 0,010389731 | 0,037528406 | protein_coding |
| ENSMUSG00000010095 | Slc3a2 | solute carrier family 3 (activators of dibasic and neutral amino acid transport), member 2 [Source:MGI Symbol;Acc:MGI:9 | 288,7060407 | 1,39240117 | **2,62515239** | 8,17413638 | 62,434832 | 2,75409E-15 | 1,22013E-13 | protein_coding |
| ENSMUSG00000022126 | Irg1 | immunoresponsive gene 1 [Source:MGI Symbol;Acc:MGI:103206] | 238,9728411 | 1,38773543 | **2,61667624** | 7,90512354 | 92,7138486 | 6,04292E-22 | 5,72359E-20 | protein_coding |
| ENSMUSG00000024621 | Csf1r | colony stimulating factor 1 receptor [Source:MGI Symbol;Acc:MGI:1339758] | 664,6130553 | 1,38724722 | **2,61579091** | 9,37666368 | 53,2513672 | 2,93483E-13 | 9,42836E-12 | protein_coding |
| ENSMUSG00000040219 | Ttc12 | tetratricopeptide repeat domain 12 [Source:MGI Symbol;Acc:MGI:2444588] | 13,92138705 | 1,38622376 | **2,61393589** | 3,81136221 | 29,000519 | 7,23589E-08 | 9,8392E-07 | protein_coding |
| ENSMUSG00000002233 | Rhoc | ras homolog gene family, member C [Source:MGI Symbol;Acc:MGI:106028] | 34,1062959 | 1,38531978 | **2,61229854** | 5,09652066 | 58,0867519 | 2,5081E-14 | 9,43714E-13 | protein_coding |
| ENSMUSG00000021493 | Pdlim7 | PDZ and LIM domain 7 [Source:MGI Symbol;Acc:MGI:1914649] | 10,42318747 | 1,38231502 | **2,60686346** | 3,3931523 | 24,2835861 | 8,31446E-07 | 9,24605E-06 | protein_coding |
| ENSMUSG00000036086 | Zranb3 | zinc finger, RAN-binding domain containing 3 [Source:MGI Symbol;Acc:MGI:1918362] | 15,98234818 | 1,37985318 | **2,60241885** | 4,00703621 | 30,4289649 | 3,46318E-08 | 5,03973E-07 | protein_coding |
| ENSMUSG00000001131 | Timp1 | tissue inhibitor of metalloproteinase 1 [Source:MGI Symbol;Acc:MGI:98752] | 4,286622452 | 1,37873878 | **2,60040942** | 2,16190505 | 11,5324028 | 0,000683936 | 0,003694395 | protein_coding |
| ENSMUSG00000032280 | Tle3 | transducin-like enhancer of split 3, homolog of Drosophila E(spl) [Source:MGI Symbol;Acc:MGI:104634] | 32,04274372 | 1,37734221 | **2,59789337** | 5,00302254 | 87,9934494 | 6,56716E-21 | 5,58999E-19 | protein_coding |
| ENSMUSG00000018537 | Pcgf2 | polycomb group ring finger 2 [Source:MGI Symbol;Acc:MGI:99161] | 3,597062404 | 1,3722669 | **2,58877018** | 1,86968852 | 13,5593624 | 0,000231136 | 0,001420295 | protein_coding |
| ENSMUSG00000025025 | Mxi1 | Max interacting protein 1 [Source:MGI Symbol;Acc:MGI:97245] | 11,00307874 | 1,37056417 | **2,58571662** | 3,4708237 | 39,3928766 | 3,46559E-10 | 7,1438E-09 | protein_coding |
| ENSMUSG00000037814 | Larp1b | La ribonucleoprotein domain family, member 1B [Source:MGI Symbol;Acc:MGI:1914604] | 2,429237755 | 1,36965785 | **2,58409274** | 1,30804331 | 12,6621083 | 0,000373141 | 0,002172172 | protein_coding |
| ENSMUSG00000026473 | Glul | glutamate-ammonia ligase (glutamine synthetase) [Source:MGI Symbol;Acc:MGI:95739] | 150,8001545 | 1,36817092 | **2,58143079** | 7,23791235 | 65,7728399 | 5,06008E-16 | 2,48192E-14 | protein_coding |
| ENSMUSG00000072568 | Fam84b | family with sequence similarity 84, member B [Source:MGI Symbol;Acc:MGI:3026924] | 13,92604231 | 1,36583204 | **2,57724921** | 3,80561922 | 41,9202424 | 9,50733E-11 | 2,11451E-09 | protein_coding |
| ENSMUSG00000001665 | Gstt3 | glutathione S-transferase, theta 3 [Source:MGI Symbol;Acc:MGI:2143526] | 2,391661354 | 1,36560448 | **2,57684272** | 1,25614809 | 11,7113821 | 0,00062119 | 0,003393839 | protein_coding |
| ENSMUSG00000097081 | | | 1,738803654 | 1,35988227 | **2,56664234** | 0,8050104 | 8,97880174 | 0,002731297 | 0,012193726 | lincRNA |
| ENSMUSG00000037820 | Tgm2 | transglutaminase 2, C polypeptide [Source:MGI Symbol;Acc:MGI:98731] | 86,92773109 | 1,35952916 | **2,56601421** | 6,44483546 | 76,5208124 | 2,17904E-18 | 1,35261E-16 | protein_coding |
| ENSMUSG00000074934 | Grem1 | gremlin 1 [Source:MGI Symbol;Acc:MGI:1344337] | 5,751298424 | 1,35837175 | **2,56395644** | 2,54264422 | 7,94866855 | 0,004812277 | 0,019581555 | protein_coding |
| ENSMUSG00000045934 | Mtmr11 | myotubularin related protein 11 [Source:MGI Symbol;Acc:MGI:2652817] | 2,732018838 | 1,3564352 | **2,56051711** | 1,45672176 | 11,4042979 | 0,000732744 | 0,00392715 | protein_coding |
| ENSMUSG00000074622 | Mafb | v-maf musculoaponeurotic fibrosarcoma oncogene family, protein B (avian) [Source:MGI Symbol;Acc:MGI:104555] | 54,44314174 | 1,35265852 | **2,55382296** | 5,76692745 | 93,2424938 | 4,62641E-22 | 4,42003E-20 | protein_coding |
| ENSMUSG00000053007 | Creb5 | cAMP responsive element binding protein 5 [Source:MGI Symbol;Acc:MGI:2443973] | 8,787073261 | 1,34976319 | **2,54870287** | 3,15490945 | 30,3343942 | 3,63621E-08 | 5,26364E-07 | protein_coding |

| | | | | | | | | | | |
|---|---|---|---|---|---|---|---|---|---|---|
| ENSMUSG00000020644 | Id2 | inhibitor of DNA binding 2 [Source:MGI Symbol;Acc:MGI:96397] | 168,5107621 | 1,34644485 | **2,54284733** | 7,39848406 | 46,5659699 | 8,85854E-12 | 2,29549E-10 | protein_coding |
| ENSMUSG00000036875 | Dna2 | DNA replication helicase 2 homolog (yeast) [Source:MGI Symbol;Acc:MGI:2443732] | 4,691062019 | 1,34601128 | **2,54208325** | 2,23081461 | 10,9426102 | 0,000939777 | 0,004868141 | protein_coding |
| ENSMUSG00000004891 | Nes | nestin [Source:MGI Symbol;Acc:MGI:101784] | 2,680571584 | 1,34495854 | **2,54022897** | 1,44346049 | 8,13575431 | 0,004340079 | 0,017987343 | protein_coding |
| ENSMUSG00000027669 | Gnb4 | guanine nucleotide binding protein (G protein), beta 4 [Source:MGI Symbol;Acc:MGI:104581] | 4,929731158 | 1,34131022 | **2,53381329** | 2,31094101 | 11,1663225 | 0,000832956 | 0,004380892 | protein_coding |
| ENSMUSG00000023827 | Agpat4 | 1-acylglycerol-3-phosphate O-acyltransferase 4 (lysophosphatidic acid acyltransferase, delta) [Source:MGI Symbol;Acc:I | 12,53083637 | 1,33928097 | **2,53025182** | 3,66258708 | 35,0129505 | 3,2752E-09 | 5,62259E-08 | protein_coding |
| ENSMUSG00000037594 | BC022687 | cDNA sequence BC022687 [Source:MGI Symbol;Acc:MGI:2443738] | 3,822425059 | 1,33792855 | **2,527881** | 1,93324731 | 14,2900293 | 0,000156693 | 0,001012695 | protein_coding |
| ENSMUSG00000026797 | Stxbp1 | syntaxin binding protein 1 [Source:MGI Symbol;Acc:MGI:107363] | 33,76164033 | 1,337734 | **2,52754013** | 5,08249223 | 55,2649988 | 1,05328E-13 | 3,63495E-12 | protein_coding |
| ENSMUSG00000005237 | Dnahc2 | dynein, axonemal, heavy chain 2 [Source:MGI Symbol;Acc:MGI:107731] | 2,636769388 | 1,33469617 | **2,52222358** | 1,39520931 | 12,6614889 | 0,000373265 | 0,002172172 | protein_coding |
| ENSMUSG00000030032 | Wdr54 | WD repeat domain 54 [Source:MGI Symbol;Acc:MGI:1922909] | 1,667257441 | 1,33445425 | **2,52180068** | 0,7789161 | 8,20756818 | 0,004171602 | 0,01740729 | protein_coding |
| ENSMUSG00000022885 | St6gal1 | beta galactoside alpha 2,6 sialyltransferase 1 [Source:MGI Symbol;Acc:MGI:108470] | 8,951275728 | 1,33353977 | **2,52020269** | 3,17057206 | 20,217584 | 6,91147E-06 | 6,2499E-05 | protein_coding |
| ENSMUSG00000031150 | Ccdc120 | coiled-coil domain containing 120 [Source:MGI Symbol;Acc:MGI:1859619] | 2,41853796 | 1,3319307 | **2,51739342** | 1,28394287 | 11,7394709 | 0,000611884 | 0,003347994 | protein_coding |
| ENSMUSG00000028599 | Tnfrsf1b | tumor necrosis factor receptor superfamily, member 1b [Source:MGI Symbol;Acc:MGI:1314883] | 887,865038 | 1,33087901 | **2,51555897** | 9,79437367 | 47,2751377 | 6,16897E-12 | 1,63322E-10 | protein_coding |
| ENSMUSG00000063193 | Cd300lb | CD300 antigen like family member B [Source:MGI Symbol;Acc:MGI:2685099] | 87,81172794 | 1,33006882 | **2,51414668** | 6,4585681 | 57,843747 | 2,83788E-14 | 1,06054E-12 | protein_coding |
| ENSMUSG00000072115 | Ang | angiogenin, ribonuclease, RNase A family, 5 [Source:MGI Symbol;Acc:MGI:88022] | 3,024599016 | 1,32911504 | **2,5124851** | 1,58839783 | 11,5416719 | 0,000680534 | 0,003681452 | protein_coding |
| ENSMUSG00000021728 | Emb | embigin [Source:MGI Symbol;Acc:MGI:95321] | 91,6372549 | 1,32820306 | **2,51089736** | 6,52202672 | 67,3435496 | 2,28087E-16 | 1,16018E-14 | protein_coding |
| ENSMUSG00000021196 | Pfkp | phosphofructokinase, platelet [Source:MGI Symbol;Acc:MGI:1891833] | 90,33467935 | 1,32764761 | **2,50993083** | 6,49960353 | 87,6711933 | 7,72919E-21 | 6,38501E-19 | protein_coding |
| ENSMUSG00000049848 | Ceacam19 | carcinoembryonic antigen-related cell adhesion molecule 19 [Source:MGI Symbol;Acc:MGI:2443001] | 5,688115743 | 1,32448119 | **2,50442809** | 2,52511716 | 15,3157756 | 9,09538E-05 | 0,000627313 | protein_coding |
| ENSMUSG00000025366 | Esyt1 | extended synaptotagmin-like protein 1 [Source:MGI Symbol;Acc:MGI:1344426] | 277,2289964 | 1,32426642 | **2,50405529** | 8,11537286 | 46,803481 | 7,84739E-12 | 2,04796E-10 | protein_coding |
| ENSMUSG00000061436 | Hipk2 | homeodomain interacting protein kinase 2 [Source:MGI Symbol;Acc:MGI:1314872] | 81,75133771 | 1,32060143 | **2,49770211** | 6,35449537 | 61,1896059 | 5,18345E-15 | 2,15722E-13 | protein_coding |
| ENSMUSG00000030060 | 8430410A17Rik | RIKEN cDNA 8430410A17 gene [Source:MGI Symbol;Acc:MGI:1914053] | 10,41962393 | 1,31869355 | **2,49440125** | 3,39708883 | 32,7011385 | 1,07474E-08 | 1,70392E-07 | protein_coding |
| ENSMUSG00000021458 | 2010111I01Rik | RIKEN cDNA 2010111I01 gene [Source:MGI Symbol;Acc:MGI:1919311] | 28,80148731 | 1,31708637 | **2,49162399** | 4,85138663 | 40,2828592 | 2,19728E-10 | 4,6786E-09 | protein_coding |
| ENSMUSG00000030108 | Slc6a13 | solute carrier family 6 (neurotransmitter transporter, GABA), member 13 [Source:MGI Symbol;Acc:MGI:95629] | 3,839975225 | 1,31654067 | **2,49068171** | 1,93535153 | 14,1981417 | 0,000164533 | 0,001057148 | protein_coding |
| ENSMUSG00000032902 | Slc16a1 | solute carrier family 16 (monocarboxylic acid transporters), member 1 [Source:MGI Symbol;Acc:MGI:106013] | 11,89723946 | 1,31627683 | **2,49022626** | 3,58685854 | 30,1371118 | 4,02554E-08 | 5,75144E-07 | protein_coding |
| ENSMUSG00000044813 | Shb | src homology 2 domain-containing transforming protein B [Source:MGI Symbol;Acc:MGI:98294] | 4,595357904 | 1,31205655 | **2,4829523** | 2,20064477 | 16,9496451 | 3,83845E-05 | 0,000289451 | protein_coding |
| ENSMUSG00000030852 | Tacc2 | transforming, acidic coiled-coil containing protein 2 [Source:MGI Symbol;Acc:MGI:1928899] | 9,205182132 | 1,31120278 | **2,48148336** | 3,20363189 | 25,0281708 | 5,64988E-07 | 6,39292E-06 | protein_coding |
| ENSMUSG00000040734 | Ppp1r13l | protein phosphatase 1, regulatory (inhibitor) subunit 13 like [Source:MGI Symbol;Acc:MGI:3525053] | 5,604828609 | 1,30979927 | **2,47907044** | 2,50336092 | 17,7467081 | 2,52354E-05 | 0,000199612 | protein_coding |
| ENSMUSG00000023067 | Cdkn1a | cyclin-dependent kinase inhibitor 1A (P21) [Source:MGI Symbol;Acc:MGI:104556] | 371,9374981 | 1,30782092 | **2,47567326** | 8,53939399 | 35,812312 | 2,17271E-09 | 3,90696E-08 | protein_coding |
| ENSMUSG00000003657 | Calb2 | calbindin 2 [Source:MGI Symbol;Acc:MGI:101914] | 11,12284518 | 1,30600675 | **2,4725621** | 3,47830941 | 25,1775979 | 5,2286E-07 | 5,95302E-06 | protein_coding |
| ENSMUSG00000001768 | Rin2 | Ras and Rab interactor 2 [Source:MGI Symbol;Acc:MGI:1921280] | 57,46778259 | 1,30576045 | **2,47214001** | 5,85145886 | 49,2732429 | 2,22679E-12 | 6,33828E-11 | protein_coding |
| ENSMUSG00000019992 | Fam54a | family with sequence similarity 54, member A [Source:MGI Symbol;Acc:MGI:1919054] | 5,746067801 | 1,30428043 | **2,4696052** | 2,55183787 | 15,5779079 | 7,91743E-05 | 0,000555484 | protein_coding |
| ENSMUSG00000049625 | Tifab | TRAF-interacting protein with forkhead-associated domain, family member B [Source:MGI Symbol;Acc:MGI:2385852] | 24,69823183 | 1,30424999 | **2,46955311** | 4,62826607 | 55,9978305 | 7,2551E-14 | 2,53859E-12 | protein_coding |
| ENSMUSG00000039959 | Hip1 | huntingtin interacting protein 1 [Source:MGI Symbol;Acc:MGI:1099804] | 87,53836214 | 1,30368462 | **2,4685855** | 6,45450464 | 47,8934403 | 4,50025E-12 | 1,20596E-10 | protein_coding |
| ENSMUSG00000056501 | Cebpb | CCAAT/enhancer binding protein (C/EBP), beta [Source:MGI Symbol;Acc:MGI:88373] | 119,3148017 | 1,3034705 | **2,46821916** | 6,90065133 | 79,6452986 | 4,48035E-19 | 3,01997E-17 | protein_coding |
| ENSMUSG00000044042 | Fmn1 | formin 1 [Source:MGI Symbol;Acc:MGI:101815] | 54,09022114 | 1,30314099 | **2,46765549** | 5,76241283 | 50,0497368 | 1,49898E-12 | 4,35695E-11 | protein_coding |
| ENSMUSG00000020696 | Rffl | ring finger and FYVE like domain containing protein [Source:MGI Symbol;Acc:MGI:1914588] | 16,60014233 | 1,30175915 | **2,46529305** | 4,06738128 | 38,9343137 | 4,38308E-10 | 8,90146E-09 | protein_coding |
| ENSMUSG00000034157 | 2310044G17Rik | RIKEN cDNA 2310044G17 gene [Source:MGI Symbol;Acc:MGI:1919185] | 78,05176996 | 1,29948237 | **2,46140553** | 6,28770136 | 41,4831735 | 1,1889E-10 | 2,61772E-09 | protein_coding |
| ENSMUSG00000040415 | Dtx3 | deltex 3 homolog (Drosophila) [Source:MGI Symbol;Acc:MGI:2135752] | 24,13202636 | 1,29579986 | **2,45513076** | 4,59796012 | 49,7413254 | 1,75412E-12 | 5,07172E-11 | protein_coding |
| ENSMUSG00000032369 | Plscr1 | phospholipid scramblase 1 [Source:MGI Symbol;Acc:MGI:893575] | 19,14648717 | 1,2893006 | **2,4440954** | 4,2660634 | 46,3091378 | 1,00992E-11 | 2,59252E-10 | protein_coding |
| ENSMUSG00000036273 | Lrrk2 | leucine-rich repeat kinase 2 [Source:MGI Symbol;Acc:MGI:1913975] | 5,362303348 | 1,28281858 | **2,43313872** | 2,42754688 | 14,3626135 | 0,000150766 | 0,000979001 | protein_coding |
| ENSMUSG00000063954 | Hist2h2aa1 | histone cluster 2, H2aa1 [Source:MGI Symbol;Acc:MGI:96097] | 12,63020997 | 1,28043755 | **2,42912638** | 3,64141388 | 17,6184486 | 2,69957E-05 | 0,00021201 | protein_coding |
| ENSMUSG00000043346 | Gm6741 | predicted gene 6741 [Source:MGI Symbol;Acc:MGI:3643504] | 4,465784272 | 1,28009723 | **2,42855344** | 2,13217806 | 7,52095159 | 0,006098546 | 0,023955924 | protein_coding |
| ENSMUSG00000006219 | Fblim1 | filamin binding LIM protein 1 [Source:MGI Symbol;Acc:MGI:1921452] | 90,50402793 | 1,27864754 | **2,42611432** | 6,50017484 | 45,0292157 | 1,94117E-11 | 4,82525E-10 | protein_coding |
| ENSMUSG00000046711 | Hmga1 | high mobility group AT-hook 1 [Source:MGI Symbol;Acc:MGI:96160] | 258,0699648 | 1,27579704 | **2,42132551** | 8,01182743 | 49,6067357 | 1,87868E-12 | 5,41759E-11 | protein_coding |
| ENSMUSG00000026478 | Lamc1 | laminin, gamma 1 [Source:MGI Symbol;Acc:MGI:99914] | 30,50354261 | 1,27466458 | **2,4194256** | 4,93778077 | 46,50589 | 9,13435E-12 | 2,36139E-10 | protein_coding |
| ENSMUSG00000019947 | Arid5b | AT rich interactive domain 5B (MRF1-like) [Source:MGI Symbol;Acc:MGI:2175912] | 16,12906294 | 1,27443101 | **2,41903393** | 4,02397358 | 42,2490407 | 8,03594E-11 | 1,81295E-09 | protein_coding |
| ENSMUSG00000059013 | Sh2d3c | SH2 domain containing 3C [Source:MGI Symbol;Acc:MGI:1351631] | 47,89886367 | 1,26960597 | **2,41095709** | 5,57645427 | 35,8178702 | 2,16652E-09 | 3,90221E-08 | protein_coding |
| ENSMUSG00000001627 | Ifrd1 | interferon-related developmental regulator 1 [Source:MGI Symbol;Acc:MGI:1316717] | 73,49437115 | 1,26953819 | **2,41084382** | 6,20350387 | 71,251042 | 3,14545E-17 | 1,70242E-15 | protein_coding |
| ENSMUSG00000035919 | Bbs9 | Bardet-Biedl syndrome 9 (human) [Source:MGI Symbol;Acc:MGI:2442833] | 3,951178334 | 1,26729822 | **2,40710357** | 1,99127408 | 14,5770509 | 0,000134543 | 0,000885165 | protein_coding |
| ENSMUSG00000026785 | Pkn3 | protein kinase N3 [Source:MGI Symbol;Acc:MGI:2388285] | 3,745707933 | 1,26704955 | **2,40668872** | 1,92210645 | 13,6915763 | 0,000215419 | 0,001338501 | protein_coding |
| ENSMUSG00000000278 | Scpep1 | serine carboxypeptidase 1 [Source:MGI Symbol;Acc:MGI:1921867] | 169,0997395 | 1,26259076 | **2,39926209** | 7,4034439 | 59,5473705 | 1,19386E-14 | 4,66793E-13 | protein_coding |
| ENSMUSG00000009687 | Fxyd5 | FXYD domain-containing ion transport regulator 5 [Source:MGI Symbol;Acc:MGI:1201785] | 180,3656575 | 1,25919837 | **2,39362702** | 7,49602047 | 63,6009533 | 1,52355E-15 | 7,03332E-14 | protein_coding |
| ENSMUSG00000030122 | Ptms | parathymosin [Source:MGI Symbol;Acc:MGI:1916452] | 113,7150915 | 1,25774962 | **2,39122456** | 6,82947333 | 73,3237649 | 1,10035E-17 | 6,32961E-16 | protein_coding |
| ENSMUSG00000071454 | Dtnb | dystrobrevin, beta [Source:MGI Symbol;Acc:MGI:1203728] | 6,082456058 | 1,25714738 | **2,39022657** | 2,60514438 | 17,2459895 | 3,2839E-05 | 0,000251605 | protein_coding |
| ENSMUSG00000034957 | Cebpa | CCAAT/enhancer binding protein (C/EBP), alpha [Source:MGI Symbol;Acc:MGI:99480] | 62,26641796 | 1,25381291 | **2,38470847** | 5,96079445 | 36,6672732 | 1,40112E-09 | 2,57858E-08 | protein_coding |
| ENSMUSG00000022257 | Laptm4b | lysosomal-associated protein transmembrane 4B [Source:MGI Symbol;Acc:MGI:1890494] | 7,286145139 | 1,25051389 | **2,37926157** | 2,87807211 | 17,8131366 | 2,43695E-05 | 0,000193599 | protein_coding |
| ENSMUSG00000054640 | Slc8a1 | solute carrier family 8 (sodium/calcium exchanger), member 1 [Source:MGI Symbol;Acc:MGI:107956] | 6,67901221 | 1,24965974 | **2,37785335** | 2,76205452 | 16,8734856 | 3,99558E-05 | 0,00029986 | protein_coding |
| ENSMUSG00000035042 | Ccl5 | chemokine (C-C motif) ligand 5 [Source:MGI Symbol;Acc:MGI:98262] | 7,881119518 | 1,24772079 | **2,37465972** | 3,00438007 | 24,9528328 | 5,87501E-07 | 6,62718E-06 | protein_coding |
| ENSMUSG00000035235 | Trim13 | tripartite motif-containing 13 [Source:MGI Symbol;Acc:MGI:1913847] | 13,22161629 | 1,2470314 | **2,37352525** | 3,73560989 | 33,3662684 | 7,63366E-09 | 1,2334E-07 | protein_coding |
| ENSMUSG00000026043 | Col3a1 | collagen, type III, alpha 1 [Source:MGI Symbol;Acc:MGI:88453] | 8,087729199 | 1,24428206 | **2,36900633** | 3,04531489 | 11,6091727 | 0,000656273 | 0,003558971 | protein_coding |
| ENSMUSG00000028417 | Tal2 | T cell acute lymphocytic leukemia 2 [Source:MGI Symbol;Acc:MGI:99540] | 7,220157268 | 1,24422653 | **2,36891517** | 2,8638598 | 24,0286016 | 9,49152E-07 | 1,04283E-05 | protein_coding |
| ENSMUSG00000028063 | Lmna | lamin A [Source:MGI Symbol;Acc:MGI:96794] | 213,816664 | 1,24420776 | **2,36888434** | 7,74021702 | 51,0900504 | 8,82243E-13 | 2,62689E-11 | protein_coding |
| ENSMUSG00000057329 | Bcl2 | B cell leukemia/lymphoma 2 [Source:MGI Symbol;Acc:MGI:88138] | 7,307337362 | 1,2426105 | **2,36626312** | 2,87307446 | 15,6709694 | 7,53722E-05 | 0,000533235 | protein_coding |
| ENSMUSG00000089942 | Pira2 | paired-Ig-like receptor A2 [Source:MGI Symbol;Acc:MGI:1195970] | 43,44785375 | 1,24094115 | **2,36352668** | 5,4405674 | 48,5400947 | 3,23605E-12 | 9,04693E-11 | protein_coding |
| ENSMUSG00000048612 | Myof | myoferlin [Source:MGI Symbol;Acc:MGI:1919192] | 245,914846 | 1,24045834 | **2,36273584** | 7,94256659 | 48,5019636 | 3,29958E-12 | 9,17785E-11 | protein_coding |
| ENSMUSG00000019951 | Uhrf1bp1l | UHRF1 (ICBP90) binding protein 1-like [Source:MGI Symbol;Acc:MGI:2442888] | 65,49731515 | 1,23936575 | **2,36094715** | 6,03764771 | 49,2787926 | 2,2205E-12 | 6,33679E-11 | protein_coding |
| ENSMUSG00000027087 | Itgav | integrin alpha V [Source:MGI Symbol;Acc:MGI:96608] | 164,1531845 | 1,23825884 | **2,35913641** | 7,35980402 | 48,1841105 | 3,8802E-12 | 1,06049E-10 | protein_coding |
| ENSMUSG00000022150 | Dab2 | disabled 2, mitogen-responsive phosphoprotein [Source:MGI Symbol;Acc:MGI:109175] | 221,4022074 | 1,23654705 | **2,3563389** | 7,79234982 | 67,1103828 | 2,56723E-16 | 1,28795E-14 | protein_coding |
| ENSMUSG00000040713 | Creg1 | cellular repressor of E1A-stimulated genes 1 [Source:MGI Symbol;Acc:MGI:1344382] | 506,1037432 | 1,23624811 | **2,3558507** | 8,98361437 | 27,6724994 | 1,43691E-07 | 1,82091E-06 | protein_coding |
| ENSMUSG00000032377 | Plscr4 | phospholipid scramblase 4 [Source:MGI Symbol;Acc:MGI:2143267] | 7,957292323 | 1,2327047 | **2,35007157** | 2,99035812 | 20,9326264 | 4,75723E-06 | 4,47114E-05 | protein_coding |
| ENSMUSG00000047767 | Atg16l2 | autophagy related 16-like 2 (S. cerevisiae) [Source:MGI Symbol;Acc:MGI:1920933] | 8,343900019 | 1,23081305 | **2,3469922** | 3,06532982 | 20,5795412 | 5,72042E-06 | 5,25539E-05 | protein_coding |
| ENSMUSG00000097101 | | | 2,67429371 | 1,23080404 | **2,34697755** | 1,41442787 | 9,01581788 | 0,002676531 | 0,011983311 | lincRNA |

| | | | | | | | | | | |
|---|---|---|---|---|---|---|---|---|---|---|
| ENSMUSG00000024164 | C3 | complement component 3 [Source:MGI Symbol;Acc:MGI:88227] | 167,7057776 | 1,22903254 | **2,34409743** | 7,39387882 | 71,6374868 | 2,58597E-17 | 1,4206E-15 | protein_coding |
| ENSMUSG00000028716 | Pdzk1ip1 | PDZK1 interacting protein 1 [Source:MGI Symbol;Acc:MGI:1914432] | 5,853256476 | 1,22852422 | **2,34327166** | 2,54006929 | 18,9539876 | 1,33909E-05 | 0,000113261 | protein_coding |
| ENSMUSG00000029490 | Mfsd7a | major facilitator superfamily domain containing 7A [Source:MGI Symbol;Acc:MGI:2442629] | 5,451226035 | 1,22735292 | **2,34136997** | 2,45551522 | 18,5705792 | 1,63728E-05 | 0,000136176 | protein_coding |
| ENSMUSG00000029223 | Uchl1 | ubiquitin carboxy-terminal hydrolase L1 [Source:MGI Symbol;Acc:MGI:103149] | 48,92189781 | 1,22727834 | **2,34124894** | 5,61189894 | 53,8295943 | 2,18654E-13 | 7,17121E-12 | protein_coding |
| ENSMUSG00000002059 | Rab34 | RAB34, member of RAS oncogene family [Source:MGI Symbol;Acc:MGI:104606] | 16,21502353 | 1,22661717 | **2,34017622** | 4,02742367 | 28,0807469 | 1,16358E-07 | 1,50935E-06 | protein_coding |
| ENSMUSG00000096210 | H1f0 | H1 histone family, member 0 [Source:MGI Symbol;Acc:MGI:95893] | 27,08333685 | 1,22515376 | **2,33780364** | 4,77253997 | 59,6697929 | 1,12185E-14 | 4,43374E-13 | protein_coding |
| ENSMUSG00000075297 | H60b | histocompatibility 60b [Source:MGI Symbol;Acc:MGI:3649078] | 2,240474082 | 1,22484081 | **2,33729658** | 1,19893425 | 10,0017607 | 0,001563906 | 0,007582806 | protein_coding |
| ENSMUSG00000026389 | Steap3 | STEAP family member 3 [Source:MGI Symbol;Acc:MGI:1915678] | 28,55379133 | 1,22125572 | **2,33149562** | 4,83865419 | 29,7981241 | 4,79456E-08 | 6,77092E-07 | protein_coding |
| ENSMUSG00000023495 | Pcbp4 | poly(rC) binding protein 4 [Source:MGI Symbol;Acc:MGI:1890471] | 6,135459736 | 1,22093763 | **2,33098162** | 2,63330705 | 13,7517696 | 0,000208624 | 0,001299407 | protein_coding |
| ENSMUSG00000037465 | Klf10 | Kruppel-like factor 10 [Source:MGI Symbol;Acc:MGI:1101353] | 35,23368844 | 1,21941587 | **2,32852419** | 5,14493348 | 44,349646 | 2,74662E-11 | 6,67634E-10 | protein_coding |
| ENSMUSG00000022074 | Tnfrsf10b | tumor necrosis factor receptor superfamily, member 10b [Source:MGI Symbol;Acc:MGI:1341090] | 9,766651318 | 1,21360732 | **2,31916798** | 3,30784663 | 19,3763374 | 1,07329E-05 | 9,2343E-05 | protein_coding |
| ENSMUSG00000002944 | Cd36 | CD36 antigen [Source:MGI Symbol;Acc:MGI:107899] | 1990,109281 | 1,21299385 | **2,31818202** | 10,9587052 | 18,4844293 | 1,71298E-05 | 0,000141828 | protein_coding |
| ENSMUSG00000031808 | Slc27a1 | solute carrier family 27 (fatty acid transporter), member 1 [Source:MGI Symbol;Acc:MGI:1347098] | 130,8563747 | 1,21060612 | **2,31434849** | 7,03174273 | 37,1716455 | 1,08175E-09 | 2,03166E-08 | protein_coding |
| ENSMUSG00000027435 | Cd93 | CD93 antigen [Source:MGI Symbol;Acc:MGI:106664] | 230,747153 | 1,21013674 | **2,31359564** | 7,85194611 | 38,4840943 | 5,52017E-10 | 1,08498E-08 | protein_coding |
| ENSMUSG00000028717 | Tal1 | T cell acute lymphocytic leukemia 1 [Source:MGI Symbol;Acc:MGI:98480] | 9,826242813 | 1,20863083 | **2,31118193** | 3,30579149 | 27,8456117 | 1,31392E-07 | 1,68842E-06 | protein_coding |
| ENSMUSG00000054000 | Tusc1 | tumor suppressor candidate 1 [Source:MGI Symbol;Acc:MGI:2684283] | 7,147155142 | 1,20575184 | **2,30657443** | 2,825041 | 20,1951044 | 6,99317E-06 | 6,31339E-05 | protein_coding |
| ENSMUSG00000015837 | Sqstm1 | sequestosome 1 [Source:MGI Symbol;Acc:MGI:107931] | 1105,291959 | 1,20542442 | **2,306051** | 10,110325 | 31,4887672 | 2,00597E-08 | 3,03576E-07 | protein_coding |
| ENSMUSG00000042659 | Arrdc4 | arrestin domain containing 4 [Source:MGI Symbol;Acc:MGI:1913662] | 23,37622609 | 1,20482437 | **2,30509205** | 4,55740164 | 34,254778 | 4,83487E-09 | 8,07306E-08 | protein_coding |
| ENSMUSG00000021796 | Bmpr1a | bone morphogenetic protein receptor, type 1A [Source:MGI Symbol;Acc:MGI:1338938] | 3,437133174 | 1,20209547 | **2,30073604** | 1,79928409 | 8,42720102 | 0,003696489 | 0,015790562 | protein_coding |
| ENSMUSG00000072844 | G530011O06Rik | RIKEN cDNA G530011O06 gene [Source:MGI Symbol;Acc:MGI:3603513] | 7,571548202 | 1,20044499 | **2,29810544** | 2,92993623 | 16,5711156 | 4,68593E-05 | 0,000346929 | lincRNA |
| ENSMUSG00000050106 | Tmc8 | transmembrane channel-like gene family 8 [Source:MGI Symbol;Acc:MGI:2669037] | 4,411019505 | 1,19902601 | **2,29584623** | 2,15776606 | 13,9847766 | 0,000184297 | 0,001167745 | protein_coding |
| ENSMUSG00000028412 | Slc44a1 | solute carrier family 44, member 1 [Source:MGI Symbol;Acc:MGI:2140592] | 49,60896576 | 1,19848981 | **2,29499308** | 5,63765241 | 38,5470449 | 5,34496E-10 | 1,06002E-08 | protein_coding |
| ENSMUSG00000079037 | Prnp | prion protein [Source:MGI Symbol;Acc:MGI:97769] | 27,49536577 | 1,19778511 | **2,29387234** | 4,78626104 | 30,3090886 | 3,68396E-08 | 5,31874E-07 | protein_coding |
| ENSMUSG00000031379 | Pir | pirin [Source:MGI Symbol;Acc:MGI:1916906] | 4,898302557 | 1,19728866 | **2,29308314** | 2,31422049 | 11,9625662 | 0,000542801 | 0,003009728 | protein_coding |
| ENSMUSG00000043085 | Tmem82 | transmembrane protein 82 [Source:MGI Symbol;Acc:MGI:2384869] | 6,886203869 | 1,1968091 | **2,29232103** | 2,77940253 | 18,5989866 | 1,61306E-05 | 0,000134563 | protein_coding |
| ENSMUSG00000021876 | Rnase4 | ribonuclease, RNase A family 4 [Source:MGI Symbol;Acc:MGI:1926217] | 29,31594699 | 1,19649708 | **2,2918253** | 4,88557989 | 53,5719087 | 2,49299E-13 | 8,12773E-12 | protein_coding |
| ENSMUSG00000037905 | Bri3bp | Bri3 binding protein [Source:MGI Symbol;Acc:MGI:1924059] | 20,48602902 | 1,1960343 | **2,29109027** | 4,36232265 | 41,3706761 | 1,25932E-10 | 2,76724E-09 | protein_coding |
| ENSMUSG00000032122 | Slc37a2 | solute carrier family 37 (glycerol-3-phosphate transporter), member 2 [Source:MGI Symbol;Acc:MGI:1929693] | 185,6792138 | 1,19189842 | **2,28453164** | 7,53722663 | 41,0997892 | 1,44652E-10 | 3,1347E-09 | protein_coding |
| ENSMUSG00000033039 | Micall1 | microtubule associated monoxygenase, calponin and LIM domain containing -like 1 [Source:MGI Symbol;Acc:MGI:10587 | 36,02072314 | 1,18824968 | **2,27876109** | 5,17436254 | 44,8813336 | 2,09344E-11 | 5,13407E-10 | protein_coding |
| ENSMUSG00000026814 | Eng | endoglin [Source:MGI Symbol;Acc:MGI:95392] | 20,99526108 | 1,18528351 | **2,27408079** | 4,39742031 | 30,6407554 | 3,10503E-08 | 4,56082E-07 | protein_coding |
| ENSMUSG00000041716 | AK010878 | cDNA sequence AK010878 [Source:MGI Symbol;Acc:MGI:4845848] | 3,920576208 | 1,18297396 | **2,27044322** | 1,99624739 | 7,7249824 | 0,005446189 | 0,021790706 | protein_coding |
| ENSMUSG00000023947 | Nfkbie | nuclear factor of kappa light polypeptide gene enhancer in B cells inhibitor, epsilon [Source:MGI Symbol;Acc:MGI:11949 | 12,06454794 | 1,18130918 | **2,26782479** | 3,60515546 | 31,9293688 | 1,59881E-08 | 2,45338E-07 | protein_coding |
| ENSMUSG00000044340 | Phlpp1 | PH domain and leucine rich repeat protein phosphatase 1 [Source:MGI Symbol;Acc:MGI:2138327] | 20,80143355 | 1,18064373 | **2,26677899** | 4,38358902 | 37,2467182 | 1,0409E-09 | 1,96164E-08 | protein_coding |
| ENSMUSG00000001313 | Rnd2 | Rho family GTPase 2 [Source:MGI Symbol;Acc:MGI:1338755] | 6,022645808 | 1,17464296 | **2,25737008** | 2,58799501 | 18,5764411 | 1,63225E-05 | 0,00013586 | protein_coding |
| ENSMUSG00000064220 | Hist2h2aa2 | histone cluster 2, H2aa2 [Source:MGI Symbol;Acc:MGI:2448283] | 7,885203623 | 1,17412293 | **2,25655654** | 2,97109543 | 10,6035785 | 0,00112869 | 0,005685886 | protein_coding |
| ENSMUSG00000043953 | Ccrl2 | chemokine (C-C motif) receptor-like 2 [Source:MGI Symbol;Acc:MGI:1920904] | 11,6192632 | 1,17216605 | **2,25349782** | 3,56796331 | 27,0597644 | 1,97261E-07 | 2,42158E-06 | protein_coding |
| ENSMUSG00000067825 | Pex26 | peroxisomal biogenesis factor 26 [Source:MGI Symbol;Acc:MGI:1921293] | 6,438837324 | 1,16797011 | **2,24695325** | 2,68936062 | 19,6675904 | 9,21511E-06 | 8,07388E-05 | protein_coding |
| ENSMUSG00000030427 | Lilra6 | leukocyte immunoglobulin-like receptor, subfamily A (with TM domain), member 6 [Source:MGI Symbol;Acc:MGI:119596 | 2,811667152 | 1,16763653 | **2,24643377** | 1,47613002 | 9,99597084 | 0,001568831 | 0,007603329 | protein_coding |
| ENSMUSG00000062593 | Lilrb4 | leukocyte immunoglobulin-like receptor, subfamily B, member 4 [Source:MGI Symbol;Acc:MGI:102701] | 303,4071589 | 1,16745836 | **2,24615636** | 8,2458666 | 38,5433254 | 5,35516E-10 | 1,06013E-08 | protein_coding |
| ENSMUSG00000075010 | AW112010 | expressed sequence AW112010 [Source:MGI Symbol;Acc:MGI:2147706] | 2,892483995 | 1,16665528 | **2,24490638** | 1,56001125 | 10,6201458 | 0,001118622 | 0,005650714 | protein_coding |
| ENSMUSG00000027935 | Rab13 | RAB13, member RAS oncogene family [Source:MGI Symbol;Acc:MGI:1927232] | 4,843685742 | 1,16600473 | **2,24389431** | 2,29764538 | 11,9764494 | 0,000538771 | 0,002994174 | protein_coding |
| ENSMUSG00000042289 | Hsd3b7 | hydroxy-delta-5-steroid dehydrogenase, 3 beta- and steroid delta-isomerase 7 [Source:MGI Symbol;Acc:MGI:2141879] | 68,57609514 | 1,16402768 | **2,24082142** | 6,10021507 | 36,9241903 | 1,22813E-09 | 2,27162E-08 | protein_coding |
| ENSMUSG00000050931 | Sgms2 | sphingomyelin synthase 2 [Source:MGI Symbol;Acc:MGI:1921692] | 6,51479303 | 1,16175129 | **2,23728848** | 2,71441445 | 12,8260194 | 0,000341832 | 0,002008401 | protein_coding |
| ENSMUSG00000001473 | Tubb6 | tubulin, beta 6 class V [Source:MGI Symbol;Acc:MGI:1915201] | 61,33388105 | 1,16004126 | **2,23463818** | 5,94145288 | 58,5158208 | 2,01663E-14 | 7,64024E-13 | protein_coding |
| ENSMUSG00000041308 | Sntb2 | syntrophin, basic 2 [Source:MGI Symbol;Acc:MGI:101771] | 20,34389259 | 1,15797329 | **2,23143733** | 4,35618495 | 31,1000323 | 2,45067E-08 | 3,63859E-07 | protein_coding |
| ENSMUSG00000031373 | Car5b | carbonic anhydrase 5b, mitochondrial [Source:MGI Symbol;Acc:MGI:1926249] | 4,581165783 | 1,15771746 | **2,23104167** | 2,21607835 | 13,0506107 | 0,000303186 | 0,001801568 | protein_coding |
| ENSMUSG00000023050 | Map3k12 | mitogen-activated protein kinase kinase kinase 12 [Source:MGI Symbol;Acc:MGI:1346881] | 8,11411258 | 1,15706114 | **2,23002694** | 3,03070097 | 19,1414994 | 1,21377E-05 | 0,000103217 | protein_coding |
| ENSMUSG00000032350 | Gclc | glutamate-cysteine ligase, catalytic subunit [Source:MGI Symbol;Acc:MGI:104990] | 59,71058705 | 1,1561112 | **2,22855906** | 5,90650692 | 52,8388195 | 3,62074E-13 | 1,14313E-11 | protein_coding |
| ENSMUSG00000034854 | Mfsd12 | major facilitator superfamily domain containing 12 [Source:MGI Symbol;Acc:MGI:3604804] | 220,9827624 | 1,15547419 | **2,22757529** | 7,78741893 | 40,4557241 | 2,01122E-10 | 4,30747E-09 | protein_coding |
| ENSMUSG00000027318 | Adam33 | a disintegrin and metallopeptidase domain 33 [Source:MGI Symbol;Acc:MGI:1341813] | 3,000013825 | 1,15360771 | **2,22469523** | 1,58706102 | 9,84780285 | 0,001700347 | 0,008179385 | protein_coding |
| ENSMUSG00000039960 | Rhou | ras homolog gene family, member U [Source:MGI Symbol;Acc:MGI:1916831] | 4,759584744 | 1,15338909 | **2,22435814** | 2,29397102 | 14,1158403 | 0,00017189 | 0,001095451 | protein_coding |
| ENSMUSG00000025037 | Maoa | monoamine oxidase A [Source:MGI Symbol;Acc:MGI:96915] | 21,1485747 | 1,15304763 | **2,22383173** | 4,40444111 | 29,4947739 | 5,60677E-08 | 7,80755E-07 | protein_coding |
| ENSMUSG00000017009 | Sdc4 | syndecan 4 [Source:MGI Symbol;Acc:MGI:1349164] | 49,95256171 | 1,15258551 | **2,22311951** | 5,65150116 | 51,731358 | 6,36382E-13 | 1,93682E-11 | protein_coding |
| ENSMUSG00000047205 | Dusp18 | dual specificity phosphatase 18 [Source:MGI Symbol;Acc:MGI:1922469] | 6,341018485 | 1,15113054 | **2,22087861** | 2,67120933 | 18,0832883 | 2,11449E-05 | 0,000171327 | protein_coding |
| ENSMUSG00000035678 | Tnfsf9 | tumor necrosis factor (ligand) superfamily, member 9 [Source:MGI Symbol;Acc:MGI:1101058] | 2,947023342 | 1,14811703 | **2,21624447** | 1,5966305 | 8,98230992 | 0,002726058 | 0,012175285 | protein_coding |
| ENSMUSG00000029553 | Tfec | transcription factor EC [Source:MGI Symbol;Acc:MGI:1333760] | 8,842369153 | 1,14680322 | **2,21422714** | 3,16180729 | 21,9970293 | 2,73073E-06 | 2,70781E-05 | protein_coding |
| ENSMUSG00000015143 | Actn1 | actinin, alpha 1 [Source:MGI Symbol;Acc:MGI:2137706] | 94,6302749 | 1,14671577 | **2,21409292** | 6,56575357 | 21,3827035 | 3,76149E-06 | 3,61887E-05 | protein_coding |
| ENSMUSG00000040522 | Tlr8 | toll-like receptor 8 [Source:MGI Symbol;Acc:MGI:2176887] | 81,96277448 | 1,14592855 | **2,21288511** | 6,36140343 | 59,8187775 | 1,04006E-14 | 4,14028E-13 | protein_coding |
| ENSMUSG00000033318 | Gstt2 | glutathione S-transferase, theta 2 [Source:MGI Symbol;Acc:MGI:106188] | 3,414629652 | 1,14569685 | **2,21252975** | 1,78100926 | 11,975246 | 0,000539119 | 0,002994593 | protein_coding |
| ENSMUSG00000071266 | Zfp946 | zinc finger protein 946 [Source:MGI Symbol;Acc:MGI:1921399] | 8,113039528 | 1,1448583 | **2,21124412** | 3,02891558 | 17,4877648 | 2,89163E-05 | 0,000224843 | protein_coding |
| ENSMUSG00000027230 | Creb3l1 | cAMP responsive element binding protein 3-like 1 [Source:MGI Symbol;Acc:MGI:1347062] | 7,793377874 | 1,14448764 | **2,21067606** | 2,9627636 | 16,1838316 | 5,74827E-05 | 0,000416873 | protein_coding |
| ENSMUSG00000039981 | Zc3h12d | zinc finger CCCH type containing 12D [Source:MGI Symbol;Acc:MGI:3045313] | 40,84263183 | 1,14428447 | **2,21036477** | 5,35581998 | 59,0337874 | 1,54988E-14 | 5,97491E-13 | protein_coding |
| ENSMUSG00000045404 | Kcnk13 | potassium channel, subfamily K, member 13 [Source:MGI Symbol;Acc:MGI:2384976] | 46,35292254 | 1,14379738 | **2,20961862** | 5,53946766 | 53,4274283 | 2,68324E-13 | 8,67082E-12 | protein_coding |
| ENSMUSG00000037992 | Rara | retinoic acid receptor, alpha [Source:MGI Symbol;Acc:MGI:97856] | 14,28457678 | 1,14196292 | **2,20681076** | 3,83976703 | 29,6536548 | 5,16551E-08 | 7,23897E-07 | protein_coding |
| ENSMUSG00000020773 | Trim47 | tripartite motif-containing 47 [Source:MGI Symbol;Acc:MGI:1917374] | 8,958475861 | 1,14161328 | **2,206276** | 3,17123009 | 22,9937508 | 1,62529E-06 | 1,68781E-05 | protein_coding |
| ENSMUSG00000030671 | Pde3b | phosphodiesterase 3B, cGMP-inhibited [Source:MGI Symbol;Acc:MGI:1333863] | 5,681766656 | 1,14054431 | **2,20464186** | 2,51964266 | 11,8438524 | 0,00057852 | 0,003187663 | protein_coding |
| ENSMUSG00000027074 | Slc43a3 | solute carrier family 43, member 3 [Source:MGI Symbol;Acc:MGI:1931054] | 28,22760809 | 1,13973494 | **2,20340538** | 4,82762245 | 31,1306415 | 2,41233E-08 | 3,58651E-07 | protein_coding |
| ENSMUSG00000036381 | P2ry14 | purinergic receptor P2Y, G-protein coupled, 14 [Source:MGI Symbol;Acc:MGI:2155705] | 5,292267217 | 1,13902667 | **2,20232391** | 2,40590092 | 14,1414054 | 0,00016957 | 0,001084297 | protein_coding |
| ENSMUSG00000026536 | Mnda | myeloid cell nuclear differentiation antigen [Source:MGI Symbol;Acc:MGI:3041120] | 48,55497791 | 1,13887705 | **2,20209552** | 5,61142082 | 45,9433252 | 1,21724E-11 | 3,085E-10 | protein_coding |
| ENSMUSG00000018983 | E2f2 | E2F transcription factor 2 [Source:MGI Symbol;Acc:MGI:1096341] | 6,627224749 | 1,13789434 | **2,20059605** | 2,71994738 | 13,0601472 | 0,000301646 | 0,001795331 | protein_coding |
| ENSMUSG00000008398 | Elk3 | ELK3, member of ETS oncogene family [Source:MGI Symbol;Acc:MGI:101762] | 44,01370744 | 1,13774499 | **2,20036825** | 5,46266536 | 58,8087071 | 1,73771E-14 | 6,62923E-13 | protein_coding |

| | | | | | | | | | | |
|---|---|---|---|---|---|---|---|---|---|---|
| ENSMUSG00000028108 | Ecm1 | extracellular matrix protein 1 [Source:MGI Symbol;Acc:MGI:103060] | 31,20258614 | 1,13610811 | **2,19787313** | 4,97073421 | 25,0196173 | 5,675E-07 | 6,41473E-06 | protein_coding |
| ENSMUSG00000006800 | Sulf2 | sulfatase 2 [Source:MGI Symbol;Acc:MGI:1919293] | 6,139039842 | 1,13238117 | **2,19220266** | 2,63336827 | 10,9759046 | 0,000923042 | 0,004788225 | protein_coding |
| ENSMUSG00000045679 | Pqlc3 | PQ loop repeat containing [Source:MGI Symbol;Acc:MGI:2444067] | 70,01802161 | 1,131533 | **2,19091422** | 6,13246576 | 33,9723074 | 5,59021E-09 | 9,27789E-08 | protein_coding |
| ENSMUSG00000000957 | Mmp14 | matrix metallopeptidase 14 (membrane-inserted) [Source:MGI Symbol;Acc:MGI:101900] | 66,18078327 | 1,13095872 | **2,19004228** | 6,05857377 | 72,359924 | 1,79319E-17 | 1,00009E-15 | protein_coding |
| ENSMUSG00000035929 | H2-Q4 | histocompatibility 2, Q region locus 4 [Source:MGI Symbol;Acc:MGI:95933] | 60,22056075 | 1,12660186 | **2,18343844** | 5,91235939 | 63,2685856 | 1,80358E-15 | 8,1884E-14 | protein_coding |
| ENSMUSG00000040613 | Apobec1 | apolipoprotein B mRNA editing enzyme, catalytic polypeptide 1 [Source:MGI Symbol;Acc:MGI:103298] | 124,4742511 | 1,12515188 | **2,18124508** | 6,96099347 | 52,4598172 | 4,39142E-13 | 1,36295E-11 | protein_coding |
| ENSMUSG00000055148 | Klf2 | Kruppel-like factor 2 (lung) [Source:MGI Symbol;Acc:MGI:1342772] | 10,17303236 | 1,12446969 | **2,18021391** | 3,32738154 | 26,8201908 | 2,23292E-07 | 2,71384E-06 | protein_coding |
| ENSMUSG00000081534 | Slc48a1 | solute carrier family 48 (heme transporter), member 1 [Source:MGI Symbol;Acc:MGI:1914989] | 421,1782366 | 1,12306919 | **2,17809849** | 8,71874715 | 28,8352589 | 7,88034E-08 | 1,0581E-06 | protein_coding |
| ENSMUSG00000032688 | Malt1 | mucosa associated lymphoid tissue lymphoma translocation gene 1 [Source:MGI Symbol;Acc:MGI:2445027] | 29,79193839 | 1,12243245 | **2,17713738** | 4,9016628 | 50,8172075 | 1,01381E-12 | 2,97827E-11 | protein_coding |
| ENSMUSG00000073421 | H2-Ab1 | histocompatibility 2, class II antigen A, beta 1 [Source:MGI Symbol;Acc:MGI:103070] | 9,258540238 | 1,12224016 | **2,17684722** | 3,2017232 | 17,9091006 | 2,3171E-05 | 0,000185554 | protein_coding |
| ENSMUSG00000097855 | | | 1,651862121 | 1,12026896 | **2,17387496** | 0,71165306 | 6,55215312 | 0,010475731 | 0,037750612 | lincRNA |
| ENSMUSG00000074800 | Gm4149 | predicted pseudogene 4149 [Source:MGI Symbol;Acc:MGI:3782325] | 6,990019792 | 1,11780335 | **2,17016292** | 2,83191585 | 6,42099147 | 0,011277918 | 0,040126452 | ɔcessed_pseudogeɪ |
| ENSMUSG00000078880 | Gm14308 | predicted gene 14308 [Source:MGI Symbol;Acc:MGI:3782921] | 3,367797263 | 1,11596445 | **2,16739852** | 1,83245446 | 6,73569917 | 0,009450219 | 0,034679209 | protein_coding |
| ENSMUSG00000029925 | Tbxas1 | thromboxane A synthase 1, platelet [Source:MGI Symbol;Acc:MGI:98497] | 80,82710468 | 1,11514675 | **2,16617042** | 6,33944845 | 56,1429096 | 6,73902E-14 | 2,38803E-12 | protein_coding |
| ENSMUSG00000040212 | Emp3 | epithelial membrane protein 3 [Source:MGI Symbol;Acc:MGI:1098729] | 141,713698 | 1,11434378 | **2,16496511** | 7,14826048 | 35,7133098 | 2,28597E-09 | 4,09054E-08 | protein_coding |
| ENSMUSG00000032507 | Fbxl2 | F-box and leucine-rich repeat protein 2 [Source:MGI Symbol;Acc:MGI:1919429] | 5,248099141 | 1,1139779 | **2,16441613** | 2,392557 | 12,1565823 | 0,000489149 | 0,002753219 | protein_coding |
| ENSMUSG00000052151 | Ppap2c | phosphatidic acid phosphatase type 2C [Source:MGI Symbol;Acc:MGI:1354945] | 11,71877823 | 1,11331675 | **2,16342446** | 3,56482353 | 20,749734 | 5,23388E-06 | 4,87741E-05 | protein_coding |
| ENSMUSG00000037649 | H2-DMa | histocompatibility 2, class II, locus DMa [Source:MGI Symbol;Acc:MGI:95921] | 6,590656377 | 1,11307146 | **2,16305667** | 2,73169492 | 17,7318785 | 2,54329E-05 | 0,000200885 | protein_coding |
| ENSMUSG00000018906 | P4ha2 | procollagen-proline, 2-oxoglutarate 4-dioxygenase (proline 4-hydroxylase), alpha II polypeptide [Source:MGI Symbol;Acc | 15,53399185 | 1,11273061 | **2,16254569** | 3,97163745 | 24,0021179 | 9,62298E-07 | 1,05622E-05 | protein_coding |
| ENSMUSG00000026315 | Serpinb8 | serine (or cysteine) peptidase inhibitor, clade B, member 8 [Source:MGI Symbol;Acc:MGI:894657] | 31,27371486 | 1,11267596 | **2,16246376** | 4,97476322 | 45,7117169 | 1,37001E-11 | 3,46029E-10 | protein_coding |
| ENSMUSG00000020261 | Slc36a1 | solute carrier family 36 (proton/amino acid symporter), member 1 [Source:MGI Symbol;Acc:MGI:2445299] | 56,59031474 | 1,11123534 | **2,1603055** | 5,82511247 | 55,0136153 | 1,19698E-13 | 4,06603E-12 | protein_coding |
| ENSMUSG00000028862 | Map3k6 | mitogen-activated protein kinase kinase kinase 6 [Source:MGI Symbol;Acc:MGI:1855691] | 4,762213614 | 1,10974906 | **2,15808107** | 2,25484817 | 13,2839883 | 0,000267682 | 0,001615949 | protein_coding |
| ENSMUSG00000051344 | Plekhm3 | pleckstrin homology domain containing, family M, member 3 [Source:MGI Symbol;Acc:MGI:2443627] | 60,48912273 | 1,10915339 | **2,1571902** | 5,92236952 | 41,1101546 | 1,43887E-10 | 3,12428E-09 | protein_coding |
| ENSMUSG00000074802 | Gas2l3 | growth arrest-specific 2 like 3 [Source:MGI Symbol;Acc:MGI:1918780] | 54,98218348 | 1,10674782 | **2,15359628** | 5,78292305 | 36,2043249 | 1,77676E-09 | 3,22665E-08 | protein_coding |
| ENSMUSG00000020085 | Aifm2 | apoptosis-inducing factor, mitochondrion-associated 2 [Source:MGI Symbol;Acc:MGI:1918611] | 15,47903518 | 1,1062637 | **2,15287373** | 3,96041429 | 23,9004384 | 1,01449E-06 | 1,10687E-05 | protein_coding |
| ENSMUSG00000024781 | Lipa | lysosomal acid lipase A [Source:MGI Symbol;Acc:MGI:96789] | 723,5152877 | 1,10466207 | **2,150485** | 9,49905661 | 19,6382938 | 9,35748E-06 | 8,17905E-05 | protein_coding |
| ENSMUSG00000022272 | Myo10 | myosin X [Source:MGI Symbol;Acc:MGI:107716] | 54,17800421 | 1,1032978 | **2,14845237** | 5,76289704 | 38,50133 | 5,47164E-10 | 1,07748E-08 | protein_coding |
| ENSMUSG00000038668 | Lpar1 | lysophosphatidic acid receptor 1 [Source:MGI Symbol;Acc:MGI:108429] | 4,487529187 | 1,10244863 | **2,14718818** | 2,20921087 | 11,1671949 | 0,000832564 | 0,004380892 | protein_coding |
| ENSMUSG00000021451 | Sema4d | sema domain, immunoglobulin domain (Ig), transmembrane domain (TM) and short cytoplasmic domain, (semaphorin) 4 | 122,2782026 | 1,09791961 | **2,14045812** | 6,93420368 | 40,7805414 | 1,70321E-10 | 3,66206E-09 | protein_coding |
| ENSMUSG00000056602 | Fry | furry homolog (Drosophila) [Source:MGI Symbol;Acc:MGI:2443895] | 4,107802365 | 1,09517924 | **2,13639622** | 2,03038231 | 11,2538131 | 0,000794596 | 0,004209367 | protein_coding |
| ENSMUSG00000002489 | Tiam1 | T cell lymphoma invasion and metastasis 1 [Source:MGI Symbol;Acc:MGI:103306] | 15,24292854 | 1,09417938 | **2,13491611** | 3,93021111 | 25,317862 | 4,86186E-07 | 5,58757E-06 | protein_coding |
| ENSMUSG00000073274 | Gm14636 | predicted gene 14636 [Source:MGI Symbol;Acc:MGI:3641976] | 2,044746098 | 1,09373617 | **2,13426034** | 1,03729186 | 6,50019257 | 0,010786281 | 0,038639996 | lincRNA |
| ENSMUSG00000049676 | Catsperg1 | cation channel, sperm-associated, gamma 1 [Source:MGI Symbol;Acc:MGI:2443617] | 2,945687446 | 1,0922219 | **2,13202137** | 1,53847089 | 9,65164762 | 0,001891818 | 0,00892914 | protein_coding |
| ENSMUSG00000056888 | Glipr1 | GLI pathogenesis-related 1 (glioma) [Source:MGI Symbol;Acc:MGI:1920940] | 222,151258 | 1,09151726 | **2,13098031** | 7,79622271 | 27,6265976 | 1,47142E-07 | 1,85183E-06 | protein_coding |
| ENSMUSG00000035158 | Mitf | microphthalmia-associated transcription factor [Source:MGI Symbol;Acc:MGI:104554] | 72,38912198 | 1,09055609 | **2,12956104** | 6,18127746 | 56,1774134 | 6,62179E-14 | 2,35448E-12 | protein_coding |
| ENSMUSG00000005802 | Slc30a4 | solute carrier family 30 (zinc transporter), member 4 [Source:MGI Symbol;Acc:MGI:1345282] | 10,00961969 | 1,0901861 | **2,12901498** | 3,33146919 | 20,3240954 | 6,53721E-06 | 5,9359E-05 | protein_coding |
| ENSMUSG00000029822 | Osbpl3 | oxysterol binding protein-like 3 [Source:MGI Symbol;Acc:MGI:1918970] | 11,73418245 | 1,08818709 | **2,12606703** | 3,5600664 | 21,9635935 | 2,77872E-06 | 2,74302E-05 | protein_coding |
| ENSMUSG00000036353 | P2ry12 | purinergic receptor P2Y, G-protein coupled 12 [Source:MGI Symbol;Acc:MGI:1918089] | 1,791112427 | 1,08795461 | **2,12572447** | 0,85997419 | 6,39763435 | 0,011427253 | 0,040549959 | protein_coding |
| ENSMUSG00000022305 | Lrp12 | low density lipoprotein-related protein 12 [Source:MGI Symbol;Acc:MGI:2443132] | 68,43469114 | 1,08681225 | **2,12404193** | 6,09938297 | 39,6995596 | 2,96192E-10 | 6,17508E-09 | protein_coding |
| ENSMUSG00000029392 | Rilpl1 | Rab interacting lysosomal protein-like 1 [Source:MGI Symbol;Acc:MGI:1922945] | 5,553994359 | 1,08668072 | **2,1238483** | 2,49115773 | 13,9382642 | 0,000188913 | 0,001194241 | protein_coding |
| ENSMUSG00000040829 | Zmynd15 | zinc finger, MYND-type containing 15 [Source:MGI Symbol;Acc:MGI:3603821] | 12,92829515 | 1,08631752 | **2,12331368** | 3,70059338 | 30,4039685 | 3,5081E-08 | 5,09161E-07 | protein_coding |
| ENSMUSG00000036620 | Mgat4b | mannoside acetylglucosaminyltransferase 4, isoenzyme B [Source:MGI Symbol;Acc:MGI:2143974] | 72,48174107 | 1,08528085 | **2,12178849** | 6,18103081 | 46,0616794 | 1,14588E-11 | 2,9143E-10 | protein_coding |
| ENSMUSG00000029781 | Fkbp9 | FK506 binding protein 9 [Source:MGI Symbol;Acc:MGI:1350921] | 5,426384615 | 1,08520754 | **2,12168068** | 2,46333475 | 8,86547813 | 0,002906141 | 0,012864534 | protein_coding |
| ENSMUSG00000015340 | Cybb | cytochrome b-245, beta polypeptide [Source:MGI Symbol;Acc:MGI:88574] | 524,1290028 | 1,08498573 | **2,1213545** | 9,03448565 | 40,2072937 | 2,28394E-10 | 4,84433E-09 | protein_coding |
| ENSMUSG00000015396 | Cd83 | CD83 antigen [Source:MGI Symbol;Acc:MGI:1328316] | 32,37820232 | 1,08433597 | **2,1203993** | 5,02831267 | 45,5355551 | 1,49893E-11 | 3,77724E-10 | protein_coding |
| ENSMUSG00000021756 | Il6st | interleukin 6 signal transducer [Source:MGI Symbol;Acc:MGI:96560] | 28,68757016 | 1,08065382 | **2,11499437** | 4,85183075 | 37,5239237 | 9,02986E-10 | 1,71645E-08 | protein_coding |
| ENSMUSG00000074677 | Sirpb1c | signal-regulatory protein beta 1C [Source:MGI Symbol;Acc:MGI:3807521] | 28,92439944 | 1,07713612 | **2,10984369** | 4,86301514 | 36,2801746 | 1,70893E-09 | 3,11378E-08 | protein_coding |
| ENSMUSG00000024242 | Map4k3 | mitogen-activated protein kinase kinase kinase kinase 3 [Source:MGI Symbol;Acc:MGI:2154405] | 48,14692796 | 1,07646395 | **2,10886091** | 5,59103826 | 41,1790971 | 1,389E-10 | 3,02642E-09 | protein_coding |
| ENSMUSG00000022114 | Spry2 | sprouty homolog 2 (Drosophila) [Source:MGI Symbol;Acc:MGI:1345138] | 4,770772812 | 1,07339986 | **2,10438673** | 2,26966052 | 10,1333353 | 0,001456117 | 0,007113542 | protein_coding |
| ENSMUSG00000022221 | Ripk3 | receptor-interacting serine-threonine kinase 3 [Source:MGI Symbol;Acc:MGI:2154952] | 30,29232672 | 1,07017359 | **2,099686** | 4,93181904 | 46,0963831 | 1,12576E-11 | 2,86978E-10 | protein_coding |
| ENSMUSG00000033307 | Mif | macrophage migration inhibitory factor [Source:MGI Symbol;Acc:MGI:96982] | 107,0015009 | 1,06805457 | **2,09660426** | 6,7432995 | 40,0868979 | 2,42913E-10 | 5,12262E-09 | protein_coding |
| ENSMUSG00000029561 | Oasl2 | 2'-5' oligoadenylate synthetase-like 2 [Source:MGI Symbol;Acc:MGI:1344390] | 135,7627169 | 1,06804485 | **2,09659013** | 7,08607644 | 41,7738259 | 1,02466E-10 | 2,26063E-09 | protein_coding |
| ENSMUSG00000012519 | Mlkl | mixed lineage kinase domain-like [Source:MGI Symbol;Acc:MGI:1921818] | 26,5735369 | 1,06671303 | **2,09465555** | 4,73887068 | 28,0472537 | 1,18389E-07 | 1,53389E-06 | protein_coding |
| ENSMUSG00000047959 | Kcna3 | potassium voltage-gated channel, shaker-related subfamily, member 3 [Source:MGI Symbol;Acc:MGI:96660] | 3,237371736 | 1,0661014 | **2,09376773** | 1,7213886 | 7,59824928 | 0,005842501 | 0,02314043 | protein_coding |
| ENSMUSG00000015745 | Plekho1 | pleckstrin homology domain containing, family O member 1 [Source:MGI Symbol;Acc:MGI:1914470] | 108,0963892 | 1,06528278 | **2,09258** | 6,75683728 | 50,0750478 | 1,47977E-12 | 4,31252E-11 | protein_coding |
| ENSMUSG00000022562 | Oplah | 5-oxoprolinase (ATP-hydrolysing) [Source:MGI Symbol;Acc:MGI:1922725] | 3,753795807 | 1,06470597 | **2,09174353** | 1,91578942 | 9,65177761 | 0,001891684 | 0,00892914 | protein_coding |
| ENSMUSG00000082016 | Pgam1-ps2 | phosphoglycerate mutase 1, pseudogene 2 [Source:MGI Symbol;Acc:MGI:3645709] | 81,45243298 | 1,06353657 | **2,09004871** | 6,35729358 | 42,6431571 | 6,56938E-11 | 1,51316E-09 | pseudogene |
| ENSMUSG00000073678 | Pgap1 | post-GPI attachment to proteins 1 [Source:MGI Symbol;Acc:MGI:2443342] | 29,60828501 | 1,06251607 | **2,08857083** | 4,89236131 | 34,6786569 | 3,88872E-09 | 6,62408E-08 | protein_coding |
| ENSMUSG00000015501 | Hivep2 | human immunodeficiency virus type I enhancer binding protein 2 [Source:MGI Symbol;Acc:MGI:1338076] | 34,80057883 | 1,06245972 | **2,08848926** | 5,13464434 | 55,0775103 | 1,15869E-13 | 3,97829E-12 | protein_coding |
| ENSMUSG00000033161 | Atp1a1 | ATPase, Na+/K+ transporting, alpha 1 polypeptide [Source:MGI Symbol;Acc:MGI:88105] | 433,2406117 | 1,06205865 | **2,08790874** | 8,75943868 | 33,8385027 | 5,98819E-09 | 9,86391E-08 | protein_coding |
| ENSMUSG00000052688 | 5430435G22Rik | RIKEN cDNA 5430435G22 gene [Source:MGI Symbol;Acc:MGI:2442295] | 132,9831732 | 1,06038486 | **2,08548777** | 7,05688159 | 32,780033 | 1,03199E-08 | 1,63852E-07 | protein_coding |
| ENSMUSG00000029098 | Acox3 | acyl-Coenzyme A oxidase 3, pristanoyl [Source:MGI Symbol;Acc:MGI:1933156] | 54,26720872 | 1,05920014 | **2,08377592** | 5,762402 | 50,8376196 | 1,00332E-12 | 2,95536E-11 | protein_coding |
| ENSMUSG00000023249 | Parp3 | poly (ADP-ribose) polymerase family, member 3 [Source:MGI Symbol;Acc:MGI:1891258] | 26,18848729 | 1,05865993 | **2,0829958** | 4,71150464 | 37,3492821 | 9,87572E-10 | 1,86434E-08 | protein_coding |
| ENSMUSG00000030530 | Furin | furin (paired basic amino acid cleaving enzyme) [Source:MGI Symbol;Acc:MGI:97513] | 85,11028469 | 1,05825689 | **2,08241396** | 6,41150489 | 57,9181889 | 2,73249E-14 | 1,02464E-12 | protein_coding |
| ENSMUSG00000033871 | Ppargc1b | peroxisome proliferative activated receptor, gamma, coactivator 1 beta [Source:MGI Symbol;Acc:MGI:2444934] | 4,182010383 | 1,05554097 | **2,07849744** | 2,07970095 | 9,52184447 | 0,002030404 | 0,009492787 | protein_coding |
| ENSMUSG00000097242 | | | 4,197626292 | 1,05224168 | **2,07374956** | 2,06534779 | 10,2235737 | 0,001386569 | 0,006807076 | lincRNA |
| ENSMUSG00000025351 | Cd63 | CD63 antigen [Source:MGI Symbol;Acc:MGI:99529] | 456,9928752 | 1,04996045 | **2,07047308** | 8,83640088 | 28,4347755 | 9,69088E-08 | 1,27666E-06 | protein_coding |
| ENSMUSG00000047407 | Tgif1 | TGFB-induced factor homeobox 1 [Source:MGI Symbol;Acc:MGI:1194497] | 54,55838443 | 1,04839758 | **2,06823136** | 5,77611872 | 39,4919218 | 3,29419E-10 | 6,81606E-09 | protein_coding |
| ENSMUSG00000047747 | Rnf150 | ring finger protein 150 [Source:MGI Symbol;Acc:MGI:2443860] | 22,70456042 | 1,0473906 | **2,06678827** | 4,50823339 | 30,8348181 | 2,80951E-08 | 4,15453E-07 | protein_coding |
| ENSMUSG00000029186 | Pi4k2b | phosphatidylinositol 4-kinase type 2 beta [Source:MGI Symbol;Acc:MGI:1914323] | 12,13527191 | 1,04722919 | **2,06655704** | 3,60975615 | 20,4219332 | 6,21138E-06 | 5,67758E-05 | protein_coding |
| ENSMUSG00000021384 | Susd3 | sushi domain containing 3 [Source:MGI Symbol;Acc:MGI:1913579] | 12,24600484 | 1,04663496 | **2,06570604** | 3,60772215 | 27,5826777 | 1,50521E-07 | 1,88357E-06 | protein_coding |

| | | | | | | | | | | |
|---|---|---|---|---|---|---|---|---|---|---|
| ENSMUSG00000036825 | Ssx2ip | synovial sarcoma, X breakpoint 2 interacting protein [Source:MGI Symbol;Acc:MGI:2139150] | 6,528333761 | 1,04471507 | **2,06295889** | 2,72763882 | 14,8768834 | 0,000114761 | 0,000769766 | protein_coding |
| ENSMUSG00000020100 | Slc29a3 | solute carrier family 29 (nucleoside transporters), member 3 [Source:MGI Symbol;Acc:MGI:1918529] | 63,55333014 | 1,04428579 | **2,06234513** | 5,99252751 | 49,3276605 | 2,16587E-12 | 6,21316E-11 | protein_coding |
| ENSMUSG00000015243 | Abca1 | ATP-binding cassette, sub-family A (ABC1), member 1 [Source:MGI Symbol;Acc:MGI:99607] | 669,4807427 | 1,04388725 | **2,0617755** | 9,38689897 | 35,656882 | 2,35315E-09 | 4,18351E-08 | protein_coding |
| ENSMUSG00000038539 | Atf5 | activating transcription factor 5 [Source:MGI Symbol;Acc:MGI:2141857] | 75,49339502 | 1,0408161 | **2,05739114** | 6,23582047 | 51,507823 | 7,13124E-13 | 2,1466E-11 | protein_coding |
| ENSMUSG00000043664 | Tmem221 | transmembrane protein 221 [Source:MGI Symbol;Acc:MGI:3525074] | 7,145604013 | 1,04061523 | **2,05710471** | 2,84162236 | 16,0268467 | 6,24506E-05 | 0,000450522 | protein_coding |
| ENSMUSG00000078875 | Gm14419 | predicted gene 14419 [Source:MGI Symbol;Acc:MGI:3702410] | 2,306656514 | 1,04049911 | **2,05693914** | 1,21079035 | 6,02271012 | 0,014122943 | 0,047891598 | protein_coding |
| ENSMUSG00000043391 | 2510009E07Rik | RIKEN cDNA 2510009E07 gene [Source:MGI Symbol;Acc:MGI:1919440] | 53,81268749 | 1,03983077 | **2,05598647** | 5,75790747 | 41,8003649 | 1,01085E-10 | 2,23464E-09 | protein_coding |
| ENSMUSG00000031762 | Mt2 | metallothionein 2 [Source:MGI Symbol;Acc:MGI:97172] | 32,49590382 | 1,03645797 | **2,0511855** | 5,02846956 | 27,4674726 | 1,59759E-07 | 1,99463E-06 | protein_coding |
| ENSMUSG00000022802 | Lmln | leishmanolysin-like (metallopeptidase M8 family) [Source:MGI Symbol;Acc:MGI:2444736] | 3,997606204 | 1,03600912 | **2,05054744** | 2,0079481 | 10,3831945 | 0,001271675 | 0,006310701 | protein_coding |
| ENSMUSG00000032567 | Aste1 | asteroid homolog 1 (Drosophila) [Source:MGI Symbol;Acc:MGI:1913845] | 4,423933305 | 1,03317667 | **2,04652554** | 2,14689331 | 9,72990328 | 0,00181294 | 0,008652813 | protein_coding |
| ENSMUSG00000061589 | Dot1l | DOT1-like, histone H3 methyltransferase (S. cerevisiae) [Source:MGI Symbol;Acc:MGI:2143886] | 106,0199222 | 1,03294732 | **2,04620023** | 6,72827549 | 38,8596787 | 4,5539E-10 | 9,21432E-09 | protein_coding |
| ENSMUSG00000023473 | Celsr3 | cadherin, EGF LAG seven-pass G-type receptor 3 (flamingo homolog, Drosophila) [Source:MGI Symbol;Acc:MGI:18582 | 5,083559175 | 1,03200749 | **2,04486767** | 2,34865823 | 12,2296749 | 0,000470354 | 0,002665177 | protein_coding |
| ENSMUSG00000025212 | Sfxn3 | sideroflexin 3 [Source:MGI Symbol;Acc:MGI:2137679] | 39,61454635 | 1,03170025 | **2,04443224** | 5,31062061 | 36,2145604 | 1,76745E-09 | 3,21506E-08 | protein_coding |
| ENSMUSG00000042210 | Abhd14a | abhydrolase domain containing 14A [Source:MGI Symbol;Acc:MGI:1915894] | 3,140169702 | 1,03165534 | **2,04436861** | 1,68883041 | 7,74358596 | 0,005390365 | 0,021590938 | protein_coding |
| ENSMUSG00000007867 | Ift43 | intraflagellar transport 43 homolog (Chlamydomonas) [Source:MGI Symbol;Acc:MGI:1923661] | 3,590715674 | 1,03083542 | **2,04320707** | 1,85554388 | 8,09263694 | 0,004444545 | 0,018323533 | protein_coding |
| ENSMUSG00000039195 | 1110008P14Rik | RIKEN cDNA 1110008P14 gene [Source:MGI Symbol;Acc:MGI:1920987] | 11,53396215 | 1,02670787 | **2,03736981** | 3,52867059 | 21,5733538 | 3,4055E-06 | 3,30754E-05 | protein_coding |
| ENSMUSG00000074158 | 9830147E19Rik | RIKEN cDNA 9830147E19 gene [Source:MGI Symbol;Acc:MGI:3036263] | 3,762899521 | 1,02647209 | **2,03703687** | 1,89610879 | 8,93344685 | 0,002799955 | 0,012459741 | protein_coding |
| ENSMUSG00000028803 | Nipal3 | NIPA-like domain containing 3 [Source:MGI Symbol;Acc:MGI:1921802] | 12,19779898 | 1,02645891 | **2,03701826** | 3,62495851 | 26,0543555 | 3,31939E-07 | 3,91731E-06 | protein_coding |
| ENSMUSG00000046798 | Cldn12 | claudin 12 [Source:MGI Symbol;Acc:MGI:1929288] | 3,572319627 | 1,02509861 | **2,03509848** | 1,85574875 | 8,67178342 | 0,003231748 | 0,014067833 | protein_coding |
| ENSMUSG00000042363 | Lgalsl | lectin, galactoside binding-like [Source:MGI Symbol;Acc:MGI:1916114] | 3,3865265 | 1,02029053 | **2,02832738** | 1,77599677 | 7,7329255 | 0,005422282 | 0,021710866 | protein_coding |
| ENSMUSG00000078247 | Airn | antisense Igf2r RNA [Source:MGI Symbol;Acc:MGI:1353471] | 3,965860811 | 1,01755242 | **2,02448145** | 2,00508723 | 8,37512115 | 0,003803922 | 0,016153149 | antisense |
| ENSMUSG00000025647 | Shisa5 | shisa homolog 5 (Xenopus laevis) [Source:MGI Symbol;Acc:MGI:1915044] | 203,4451707 | 1,01707141 | **2,02380658** | 7,66964328 | 37,7040341 | 8,23341E-10 | 1,57872E-08 | protein_coding |
| ENSMUSG00000035168 | Tanc1 | tetratricopeptide repeat, ankyrin repeat and coiled-coil containing 1 [Source:MGI Symbol;Acc:MGI:1914110] | 19,78276114 | 1,01699413 | **2,02369817** | 4,30954432 | 26,3010762 | 2,92126E-07 | 3,50009E-06 | protein_coding |
| ENSMUSG00000074417 | Gm14548 | predicted gene 14548 [Source:MGI Symbol;Acc:MGI:3709645] | 6,37029061 | 1,01680108 | **2,02342739** | 2,67348858 | 9,85902718 | 0,001690004 | 0,008136757 | protein_coding |
| ENSMUSG00000037960 | 1110007C09Rik | RIKEN cDNA 1110007C09 gene [Source:MGI Symbol;Acc:MGI:1915730] | 52,16056571 | 1,01553159 | **2,02164767** | 5,70577555 | 39,9453007 | 2,61175E-10 | 5,48668E-09 | protein_coding |
| ENSMUSG00000037110 | Ralgapa2 | Ral GTPase activating protein, alpha subunit 2 (catalytic) [Source:MGI Symbol;Acc:MGI:3036245] | 36,98429191 | 1,0137633 | **2,01917129** | 5,2170685 | 27,8185363 | 1,33243E-07 | 1,70623E-06 | protein_coding |
| ENSMUSG00000050914 | Ankrd37 | ankyrin repeat domain 37 [Source:MGI Symbol;Acc:MGI:3603344] | 2,758835962 | 1,01295622 | **2,01804203** | 1,48107873 | 6,31370366 | 0,011980831 | 0,04214966 | protein_coding |
| ENSMUSG00000026107 | Nabp1 | nucleic acid binding protein 1 [Source:MGI Symbol;Acc:MGI:1923258] | 30,01981436 | 1,01205002 | **2,01677483** | 4,91432257 | 28,7672665 | 8,1619E-08 | 1,08961E-06 | protein_coding |
| ENSMUSG00000039982 | Dtx4 | deltex 4 homolog (Drosophila) [Source:MGI Symbol;Acc:MGI:2672905] | 55,52540035 | 1,01075021 | **2,01495861** | 5,79884703 | 29,9405338 | 4,45502E-08 | 6,31577E-07 | protein_coding |
| ENSMUSG00000038175 | Mylip | myosin regulatory light chain interacting protein [Source:MGI Symbol;Acc:MGI:2388271] | 75,09433008 | 1,00558291 | **2,00775456** | 6,23089603 | 52,9818785 | 3,3664E-13 | 1,06898E-11 | protein_coding |
| ENSMUSG00000037966 | Ninj1 | ninjurin 1 [Source:MGI Symbol;Acc:MGI:1196617] | 166,8411989 | 1,00406689 | **2,00564586** | 7,38347775 | 39,7528996 | 2,88212E-10 | 6,02013E-09 | protein_coding |
| ENSMUSG00000007891 | Ctsd | cathepsin D [Source:MGI Symbol;Acc:MGI:88562] | 5035,457245 | 1,00107008 | **2,00148399** | 12,2979203 | 11,5616972 | 0,000673244 | 0,003645603 | protein_coding |
| ENSMUSG00000035637 | Grhpr | glyoxylate reductase/hydroxypyruvate reductase [Source:MGI Symbol;Acc:MGI:1923488] | 3,356194158 | 1,00088205 | **2,00122315** | 1,7466402 | 8,00273115 | 0,004670685 | 0,019098182 | protein_coding |
| ENSMUSG00000021360 | Gcnt2 | glucosaminyl (N-acetyl) transferase 2, I-branching enzyme [Source:MGI Symbol;Acc:MGI:1100870] | 2,192652156 | 0,9996133 | **1,999464** | 1,15539284 | 6,15156227 | 0,013129607 | 0,045277775 | protein_coding |
| ENSMUSG00000020782 | Llgl2 | lethal giant larvae homolog 2 (Drosophila) [Source:MGI Symbol;Acc:MGI:1918843] | 4,076187646 | 0,9995698 | **1,9994037** | 2,02751128 | 8,7356182 | 0,003120535 | 0,013681292 | protein_coding |
| ENSMUSG00000027002 | Nckap1 | NCK-associated protein 1 [Source:MGI Symbol;Acc:MGI:1355333] | 6,436467719 | 0,99929828 | **1,99902744** | 2,70551676 | 9,45517738 | 0,002105539 | 0,009802356 | protein_coding |
| ENSMUSG00000070427 | Il18bp | interleukin 18 binding protein [Source:MGI Symbol;Acc:MGI:1333800] | 9,699660507 | 0,99873094 | **1,99824149** | 3,27896255 | 13,4599005 | 0,000243717 | 0,001491763 | protein_coding |
| ENSMUSG00000031521 | Aga | aspartylglucosaminidase [Source:MGI Symbol;Acc:MGI:104873] | 24,2272612 | 0,9982016 | **1,99750843** | 4,60664106 | 28,7953215 | 8,04452E-08 | 1,07655E-06 | protein_coding |
| ENSMUSG00000066595 | Mfsd7b | major facilitator superfamily domain containing 7B [Source:MGI Symbol;Acc:MGI:2444881] | 42,01245936 | 0,99647511 | **1,99511943** | 5,3968511 | 29,0878505 | 6,91694E-08 | 9,44055E-07 | protein_coding |
| ENSMUSG00000036181 | Hist1h1c | histone cluster 1, H1c [Source:MGI Symbol;Acc:MGI:1931526] | 63,20629476 | 0,9959194 | **1,99435107** | 5,98264019 | 54,9304652 | 1,2487E-13 | 4,20844E-12 | protein_coding |
| ENSMUSG00000028970 | Abcb1b | ATP-binding cassette, sub-family B (MDR/TAP), member 1B [Source:MGI Symbol;Acc:MGI:97568] | 18,55538609 | 0,99479495 | **1,99279727** | 4,21796421 | 18,1052431 | 2,09025E-05 | 0,000169738 | protein_coding |
| ENSMUSG00000058056 | Palld | palladin, cytoskeletal associated protein [Source:MGI Symbol;Acc:MGI:1919583] | 9,366787296 | 0,9947003 | **1,99266654** | 3,23439578 | 12,5027351 | 0,000406357 | 0,002341185 | protein_coding |
| ENSMUSG00000042272 | Sestd1 | SEC14 and spectrin domains 1 [Source:MGI Symbol;Acc:MGI:1916262] | 43,96652686 | 0,99465451 | **1,99260329** | 5,46245018 | 29,1881674 | 6,56791E-08 | 8,99771E-07 | protein_coding |
| ENSMUSG00000041440 | Gk5 | glycerol kinase 5 (putative) [Source:MGI Symbol;Acc:MGI:2443336] | 3,035296728 | 0,99431806 | **1,99213865** | 1,60995224 | 8,01463867 | 0,004640073 | 0,019036775 | protein_coding |
| ENSMUSG00000026811 | St6galnac6 | ST6 (alpha-N-acetyl-neuraminyl-2,3-beta-galactosyl-1,3)-N-acetylgalactosaminide alpha-2,6-sialyltransferase 6 [Source:I | 24,11068047 | 0,99414571 | **1,99190067** | 4,59801365 | 36,0563012 | 1,91698E-09 | 3,46412E-08 | protein_coding |
| ENSMUSG00000033446 | Lpar6 | lysophosphatidic acid receptor 6 [Source:MGI Symbol;Acc:MGI:1914418] | 21,41979969 | 0,99355283 | **1,99108227** | 4,42194365 | 34,8159971 | 3,62384E-09 | 6,19208E-08 | protein_coding |
| ENSMUSG00000030231 | Plekha5 | pleckstrin homology domain containing, family A member 5 [Source:MGI Symbol;Acc:MGI:1923802] | 2,413068711 | 0,99350405 | **1,99101495** | 1,29974475 | 7,13293937 | 0,007568056 | 0,028761755 | protein_coding |
| ENSMUSG00000069206 | Zfp874a | zinc finger protein 874a [Source:MGI Symbol;Acc:MGI:3040703] | 7,609519648 | 0,98972077 | **1,98580061** | 2,93933607 | 11,5111363 | 0,000691805 | 0,003727738 | protein_coding |
| ENSMUSG00000020184 | Mdm2 | transformed mouse 3T3 cell double minute 2 [Source:MGI Symbol;Acc:MGI:96952] | 209,3593302 | 0,98866274 | **1,98434481** | 7,71085338 | 32,9270443 | 9,56829E-09 | 1,52801E-07 | protein_coding |
| ENSMUSG00000051790 | Nlgn2 | neuroligin 2 [Source:MGI Symbol;Acc:MGI:2681835] | 10,27342578 | 0,98801093 | **1,98344849** | 3,3583918 | 17,5597622 | 2,78418E-05 | 0,000218031 | protein_coding |
| ENSMUSG00000020635 | Fkbp1b | FK506 binding protein 1b [Source:MGI Symbol;Acc:MGI:1336205] | 5,615343495 | 0,98769593 | **1,98301547** | 2,49601097 | 11,670269 | 0,000635068 | 0,003459343 | protein_coding |
| ENSMUSG00000025330 | Padi4 | peptidyl arginine deiminase, type IV [Source:MGI Symbol;Acc:MGI:1338898] | 7,145217349 | 0,98750004 | **1,98274623** | 2,83516693 | 14,1424919 | 0,000169472 | 0,001084297 | protein_coding |
| ENSMUSG00000018932 | Map2k3 | mitogen-activated protein kinase kinase 3 [Source:MGI Symbol;Acc:MGI:1346868] | 64,19161138 | 0,98731744 | **1,98249529** | 6,00705193 | 44,1242139 | 3,08185E-11 | 7,40925E-10 | protein_coding |
| ENSMUSG00000019796 | Lrp11 | low density lipoprotein receptor-related protein 11 [Source:MGI Symbol;Acc:MGI:2442989] | 11,04508716 | 0,98647722 | **1,98134103** | 3,47702102 | 20,7376385 | 5,26705E-06 | 4,9E-05 | protein_coding |
| ENSMUSG00000026558 | Uck2 | uridine-cytidine kinase 2 [Source:MGI Symbol;Acc:MGI:1931744] | 5,698471924 | 0,98483026 | **1,97908045** | 2,52983599 | 11,8360678 | 0,000580944 | 0,003199413 | protein_coding |
| ENSMUSG00000033249 | Hsf4 | heat shock transcription factor 4 [Source:MGI Symbol;Acc:MGI:1347058] | 4,150304269 | 0,98423855 | **1,97826892** | 2,03393666 | 8,38854722 | 0,003775926 | 0,016067427 | protein_coding |
| ENSMUSG00000023045 | Soat2 | sterol O-acyltransferase 2 [Source:MGI Symbol;Acc:MGI:1332226] | 2,899780951 | 0,98399624 | **1,97793668** | 1,52236043 | 6,5144752 | 0,010699989 | 0,038395162 | protein_coding |
| ENSMUSG00000035202 | Lars2 | leucyl-tRNA synthetase, mitochondrial [Source:MGI Symbol;Acc:MGI:2142973] | 126,19837 | 0,98260712 | **1,97603311** | 6,98277914 | 19,8198104 | 8,50961E-06 | 7,52779E-05 | protein_coding |
| ENSMUSG00000024899 | Papss2 | 3'-phosphoadenosine 5'-phosphosulfate synthase 2 [Source:MGI Symbol;Acc:MGI:1330223] | 4,869867612 | 0,98062112 | **1,9733148** | 2,30886856 | 7,62488253 | 0,005756842 | 0,022834087 | protein_coding |
| ENSMUSG00000034485 | Uaca | uveal autoantigen with coiled-coil domains and ankyrin repeats [Source:MGI Symbol;Acc:MGI:1919815] | 6,512648709 | 0,97981395 | **1,97221106** | 2,71824757 | 8,25961187 | 0,004053679 | 0,017012137 | protein_coding |
| ENSMUSG00000053580 | Tanc2 | tetratricopeptide repeat, ankyrin repeat and coiled-coil containing 2 [Source:MGI Symbol;Acc:MGI:2444121] | 55,91234531 | 0,97967059 | **1,97201509** | 5,81111833 | 35,5540784 | 2,48067E-09 | 4,37482E-08 | protein_coding |
| ENSMUSG00000068923 | Syt11 | synaptotagmin XI [Source:MGI Symbol;Acc:MGI:1859547] | 12,21490436 | 0,97912828 | **1,97127394** | 3,61913753 | 17,0078638 | 3,72253E-05 | 0,000281289 | protein_coding |
| ENSMUSG00000070056 | Mfhas1 | malignant fibrous histiocytoma amplified sequence 1 [Source:MGI Symbol;Acc:MGI:1098644] | 36,75664224 | 0,9763153 | **1,96743408** | 5,20315449 | 39,7859785 | 2,83372E-10 | 5,9303E-09 | protein_coding |
| ENSMUSG00000040061 | Plcb2 | phospholipase C, beta 2 [Source:MGI Symbol;Acc:MGI:107465] | 33,79460869 | 0,97603314 | **1,96704934** | 5,07886253 | 35,4582384 | 2,60578E-09 | 4,58075E-08 | protein_coding |
| ENSMUSG00000009073 | Nf2 | neurofibromatosis 2 [Source:MGI Symbol;Acc:MGI:97307] | 111,047077 | 0,97180035 | **1,96128658** | 6,79571628 | 40,343188 | 2,13047E-10 | 4,55398E-09 | protein_coding |
| ENSMUSG00000022125 | Cln5 | ceroid-lipofuscinosis, neuronal 5 [Source:MGI Symbol;Acc:MGI:2442253] | 144,322567 | 0,97149982 | **1,96087806** | 7,17597088 | 48,5226425 | 3,26497E-12 | 9,10464E-11 | protein_coding |
| ENSMUSG00000028773 | Fabp3 | fatty acid binding protein 3, muscle and heart [Source:MGI Symbol;Acc:MGI:95476] | 5,613616652 | 0,97083839 | **1,95997926** | 2,50498798 | 8,12774024 | 0,004359305 | 0,018033012 | protein_coding |
| ENSMUSG00000025993 | Slc40a1 | solute carrier family 40 (iron-regulated transporter), member 1 [Source:MGI Symbol;Acc:MGI:1315204] | 12,75336406 | 0,97011106 | **1,95899139** | 3,69715465 | 6,51592871 | 0,010691247 | 0,038395162 | protein_coding |
| ENSMUSG00000042302 | Ehbp1 | EH domain binding protein 1 [Source:MGI Symbol;Acc:MGI:2667252] | 6,041205564 | 0,96904229 | **1,95754069** | 2,61634366 | 12,3813841 | 0,000433636 | 0,00247242 | protein_coding |
| ENSMUSG00000031971 | 1700054N08Rik | RIKEN cDNA 1700054N08 gene [Source:MGI Symbol;Acc:MGI:1920670] | 4,73409665 | 0,96824599 | **1,95646051** | 2,2352464 | 9,6999639 | 0,001842716 | 0,008756724 | protein_coding |
| ENSMUSG00000022440 | C1qtnf6 | C1q and tumor necrosis factor related protein 6 [Source:MGI Symbol;Acc:MGI:1919959] | 3,28967068 | 0,96696028 | **1,95471772** | 1,73298173 | 7,25312082 | 0,00707779 | 0,027171096 | protein_coding |

| | | | | | | | | | | |
|---|---|---|---|---|---|---|---|---|---|---|
| ENSMUSG00000078122 | F630028O10Rik | RIKEN cDNA F630028O10 gene [Source:MGI Symbol;Acc:MGI:3641813] | 8,668878046 | 0,96579124 | **1,95313442** | 3,1226356 | 14,2442157 | 0,000160553 | 0,001034605 | antisense |
| ENSMUSG00000030545 | Pex11a | peroxisomal biogenesis factor 11 alpha [Source:MGI Symbol;Acc:MGI:1338788] | 5,733082604 | 0,965022 | **1,95209329** | 2,54167106 | 11,2888507 | 0,000779739 | 0,004146658 | protein_coding |
| ENSMUSG00000027999 | Pla2g12a | phospholipase A2, group XIIA [Source:MGI Symbol;Acc:MGI:1913600] | 11,81103122 | 0,96483626 | **1,95184198** | 3,56351015 | 20,4890931 | 5,9972E-06 | 5,49552E-05 | protein_coding |
| ENSMUSG00000027660 | Skil | SKI-like [Source:MGI Symbol;Acc:MGI:106203] | 135,2863605 | 0,96443702 | **1,95130192** | 7,08237677 | 47,924435 | 4,42966E-12 | 1,19286E-10 | protein_coding |
| ENSMUSG00000016526 | Dyrk3 | dual-specificity tyrosine-(Y)-phosphorylation regulated kinase 3 [Source:MGI Symbol;Acc:MGI:1330300] | 2,33077551 | 0,96431518 | **1,95113713** | 1,21018203 | 6,27371958 | 0,012254164 | 0,04291887 | protein_coding |
| ENSMUSG00000050605 | Zfp61 | zinc finger protein 61 [Source:MGI Symbol;Acc:MGI:99663] | 4,868207316 | 0,96262966 | **1,94885892** | 2,2921242 | 9,71216581 | 0,001830522 | 0,008714012 | protein_coding |
| ENSMUSG00000003545 | Fosb | FBJ osteosarcoma oncogene B [Source:MGI Symbol;Acc:MGI:95575] | 2,744593963 | 0,95949094 | **1,9446236** | 1,47544365 | 7,03217914 | 0,008005788 | 0,030185171 | protein_coding |
| ENSMUSG00000053113 | Socs3 | suppressor of cytokine signaling 3 [Source:MGI Symbol;Acc:MGI:1201791] | 57,5761231 | 0,95940247 | **1,94450437** | 5,85559866 | 28,9168459 | 7,5553E-08 | 1,02395E-06 | protein_coding |
| ENSMUSG00000017493 | Igfbp4 | insulin-like growth factor binding protein 4 [Source:MGI Symbol;Acc:MGI:96439] | 65,94031674 | 0,95922835 | **1,94426969** | 6,04788309 | 38,4796996 | 5,53262E-10 | 1,08548E-08 | protein_coding |
| ENSMUSG00000063524 | Eno1 | enolase 1, alpha non-neuron [Source:MGI Symbol;Acc:MGI:95393] | 733,2715062 | 0,9583049 | **1,94302559** | 9,5184665 | 24,2537667 | 8,44418E-07 | 9,36188E-06 | protein_coding |
| ENSMUSG00000021823 | Vcl | vinculin [Source:MGI Symbol;Acc:MGI:98927] | 59,05601811 | 0,95816507 | **1,94283727** | 5,892409 | 27,8666863 | 1,29968E-07 | 1,67209E-06 | protein_coding |
| ENSMUSG00000058881 | Zfp516 | zinc finger protein 516 [Source:MGI Symbol;Acc:MGI:2443957] | 43,24283083 | 0,9579757 | **1,94258227** | 5,43734182 | 32,0656459 | 1,4905E-08 | 2,30002E-07 | protein_coding |
| ENSMUSG00000032489 | Kif9 | kinesin family member 9 [Source:MGI Symbol;Acc:MGI:1098237] | 2,565535718 | 0,95758559 | **1,94205705** | 1,3452574 | 6,39928189 | 0,011416653 | 0,040549959 | protein_coding |
| ENSMUSG00000031722 | Hp | haptoglobin [Source:MGI Symbol;Acc:MGI:96211] | 12,81388338 | 0,95657719 | **1,9407001** | 3,72725879 | 17,7963485 | 2,45855E-05 | 0,000195033 | protein_coding |
| ENSMUSG00000033033 | Calhm2 | calcium homeostasis modulator 2 [Source:MGI Symbol;Acc:MGI:1919941] | 10,90897236 | 0,95537073 | **1,93907785** | 3,44890179 | 19,6863987 | 9,12485E-06 | 8,00757E-05 | protein_coding |
| ENSMUSG00000020053 | Igf1 | insulin-like growth factor 1 [Source:MGI Symbol;Acc:MGI:96432] | 199,3606232 | 0,95297849 | **1,93586519** | 7,63936133 | 28,2033512 | 1,09215E-07 | 1,42343E-06 | protein_coding |
| ENSMUSG00000068854 | Hist2h2be | histone cluster 2, H2be [Source:MGI Symbol;Acc:MGI:2448415] | 2,480953768 | 0,95059141 | **1,93266476** | 1,33851222 | 6,58259718 | 0,010298053 | 0,037316856 | protein_coding |
| ENSMUSG00000025324 | Atp10a | ATPase, class V, type 10A [Source:MGI Symbol;Acc:MGI:1330809] | 5,043522762 | 0,9493743 | **1,93103498** | 2,35264303 | 7,96978566 | 0,004756457 | 0,019398364 | protein_coding |
| ENSMUSG00000042215 | Bag2 | BCL2-associated athanogene 2 [Source:MGI Symbol;Acc:MGI:1891254] | 3,717233999 | 0,94828891 | **1,92958274** | 1,89885088 | 6,19313452 | 0,012824683 | 0,044416297 | protein_coding |
| ENSMUSG00000030539 | Sema4b | sema domain, immunoglobulin domain (Ig), transmembrane domain (TM) and short cytoplasmic domain, (semaphorin) 4 | 12,72293115 | 0,9481899 | **1,92945032** | 3,67891645 | 18,107036 | 2,08828E-05 | 0,000169704 | protein_coding |
| ENSMUSG00000041235 | Chd7 | chromodomain helicase DNA binding protein 7 [Source:MGI Symbol;Acc:MGI:2444748] | 16,78524049 | 0,94587516 | **1,92635709** | 4,07775323 | 23,960049 | 9,83556E-07 | 1,07526E-05 | protein_coding |
| ENSMUSG00000028864 | Hgf | hepatocyte growth factor [Source:MGI Symbol;Acc:MGI:96079] | 11,63996422 | 0,94432769 | **1,92429194** | 3,52221276 | 18,3424983 | 1,84544E-05 | 0,0001512 | protein_coding |
| ENSMUSG00000041439 | Mfsd6 | major facilitator superfamily domain containing 6 [Source:MGI Symbol;Acc:MGI:1922925] | 33,46359911 | 0,94412964 | **1,92402779** | 5,06567165 | 32,5077509 | 1,18718E-08 | 1,8687E-07 | protein_coding |
| ENSMUSG00000037552 | Plekhg2 | pleckstrin homology domain containing, family G (with RhoGef domain) member 2 [Source:MGI Symbol;Acc:MGI:21418 | 19,75418298 | 0,94145691 | **1,92046665** | 4,3161463 | 33,2614283 | 8,05649E-09 | 1,29221E-07 | protein_coding |
| ENSMUSG00000040451 | Sgms1 | sphingomyelin synthase 1 [Source:MGI Symbol;Acc:MGI:2444110] | 25,47980719 | 0,94038702 | **1,91904297** | 4,67979451 | 26,0646864 | 3,30168E-07 | 3,9006E-06 | protein_coding |
| ENSMUSG00000027459 | Fam110a | family with sequence similarity 110, member A [Source:MGI Symbol;Acc:MGI:1921097] | 8,220582712 | 0,93951686 | **1,91788586** | 3,03949065 | 14,5379099 | 0,000137367 | 0,00090051 | protein_coding |
| ENSMUSG00000023809 | Rps6ka2 | ribosomal protein S6 kinase, polypeptide 2 [Source:MGI Symbol;Acc:MGI:1342290] | 31,67587147 | 0,93950267 | **1,91786699** | 4,98601546 | 27,6644345 | 1,44291E-07 | 1,82431E-06 | protein_coding |
| ENSMUSG00000029426 | Scarb2 | scavenger receptor class B, member 2 [Source:MGI Symbol;Acc:MGI:1196458] | 52,68692059 | 0,93947881 | **1,91783527** | 5,72301108 | 30,0652946 | 4,17741E-08 | 5,94523E-07 | protein_coding |
| ENSMUSG00000026547 | Tagln2 | transgelin 2 [Source:MGI Symbol;Acc:MGI:1312985] | 186,9059008 | 0,93518644 | **1,91213773** | 7,54728623 | 36,5401703 | 1,49552E-09 | 2,73855E-08 | protein_coding |
| ENSMUSG00000056220 | Pla2g4a | phospholipase A2, group IVA (cytosolic, calcium-dependent) [Source:MGI Symbol;Acc:MGI:1195256] | 45,6662821 | 0,93444303 | **1,91115267** | 5,52073856 | 35,2427608 | 2,91062E-09 | 5,03606E-08 | protein_coding |
| ENSMUSG00000031149 | Praf2 | PRA1 domain family 2 [Source:MGI Symbol;Acc:MGI:1859607] | 14,33425621 | 0,93179572 | **1,90764897** | 3,8535029 | 25,1473063 | 5,31138E-07 | 6,03476E-06 | protein_coding |
| ENSMUSG00000035107 | Dcbld2 | discoidin, CUB and LCCL domain containing 2 [Source:MGI Symbol;Acc:MGI:1920629] | 13,5853788 | 0,93130615 | **1,90700173** | 3,7692249 | 22,727614 | 1,86666E-06 | 1,91852E-05 | protein_coding |
| ENSMUSG00000046079 | Lrrc8d | leucine rich repeat containing 8D [Source:MGI Symbol;Acc:MGI:1922368] | 75,91637264 | 0,928854 | **1,90376315** | 6,24836731 | 33,8259034 | 6,0271E-09 | 9,91314E-08 | protein_coding |
| ENSMUSG00000054150 | 4831426I19Rik | RIKEN cDNA 4831426I19 gene [Source:MGI Symbol;Acc:MGI:2442408] | 14,82792669 | 0,92754523 | **1,90203689** | 3,89321754 | 19,119917 | 1,22757E-05 | 0,00010431 | protein_coding |
| ENSMUSG00000035239 | Neu3 | neuraminidase 3 [Source:MGI Symbol;Acc:MGI:1355305] | 2,342037719 | 0,9269793 | **1,90129093** | 1,21972924 | 5,97889078 | 0,014478105 | 0,048899767 | protein_coding |
| ENSMUSG00000042286 | Stab1 | stabilin 1 [Source:MGI Symbol;Acc:MGI:2178742] | 527,893626 | 0,92657477 | **1,90075788** | 9,04444874 | 24,8283933 | 6,26675E-07 | 7,05459E-06 | protein_coding |
| ENSMUSG00000018819 | Lsp1 | lymphocyte specific 1 [Source:MGI Symbol;Acc:MGI:96832] | 4,739997203 | 0,92654611 | **1,90072012** | 2,23269058 | 8,63058824 | 0,003305648 | 0,014355398 | protein_coding |
| ENSMUSG00000004085 | B230120H23Rik | RIKEN cDNA B230120H23 gene [Source:MGI Symbol;Acc:MGI:2443258] | 22,64750436 | 0,92620725 | **1,90027373** | 4,50759595 | 19,6055445 | 9,51926E-06 | 8,30723E-05 | protein_coding |
| ENSMUSG00000044080 | S100a1 | S100 calcium binding protein A1 [Source:MGI Symbol;Acc:MGI:1338917] | 23,82933041 | 0,92593115 | **1,89991009** | 4,57629572 | 20,6250193 | 5,58613E-06 | 5,16189E-05 | protein_coding |
| ENSMUSG00000022385 | Gtse1 | G two S phase expressed protein 1 [Source:MGI Symbol;Acc:MGI:1352755] | 11,32790387 | 0,92571372 | **1,89962378** | 3,51218827 | 10,2623794 | 0,001357705 | 0,006686284 | protein_coding |
| ENSMUSG00000038172 | Ttc39b | tetratricopeptide repeat domain 39B [Source:MGI Symbol;Acc:MGI:1917113] | 18,14664122 | 0,92529025 | **1,89906626** | 4,19616479 | 22,934218 | 1,67641E-06 | 1,73761E-05 | protein_coding |
| ENSMUSG00000074506 | Gm10705 | predicted gene 10705 [Source:MGI Symbol;Acc:MGI:3708678] | 12,67544363 | 0,92485922 | **1,89849897** | 3,67591179 | 8,86829762 | 0,002901656 | 0,012849857 | ocessed_pseudoge |
| ENSMUSG00000001700 | Gramd3 | GRAM domain containing 3 [Source:MGI Symbol;Acc:MGI:1914815] | 7,221318892 | 0,92340578 | **1,89658729** | 2,87268641 | 12,0811845 | 0,000509332 | 0,002853663 | protein_coding |
| ENSMUSG00000002602 | Axl | AXL receptor tyrosine kinase [Source:MGI Symbol;Acc:MGI:1347244] | 10,52118047 | 0,92232087 | **1,8951616** | 3,42219998 | 9,72380276 | 0,001818968 | 0,008667252 | protein_coding |
| ENSMUSG00000014444 | Fam38a | family with sequence similarity 38, member A [Source:MGI Symbol;Acc:MGI:3603204] | 281,3913263 | 0,92075898 | **1,89311097** | 8,13694936 | 23,9149758 | 1,00685E-06 | 1,09963E-05 | protein_coding |
| ENSMUSG00000064267 | Hvcn1 | hydrogen voltage-gated channel 1 [Source:MGI Symbol;Acc:MGI:1921346] | 133,5715072 | 0,91970735 | **1,89173151** | 7,06208003 | 31,3022107 | 2,20828E-08 | 3,301E-07 | protein_coding |
| ENSMUSG00000032661 | Oas3 | 2'-5' oligoadenylate synthetase 3 [Source:MGI Symbol;Acc:MGI:2180850] | 131,3502024 | 0,9167056 | **1,88779957** | 7,03843362 | 22,6742865 | 1,91919E-06 | 1,96397E-05 | protein_coding |
| ENSMUSG00000028874 | Fgr | Gardner-Rasheed feline sarcoma viral (Fgr) oncogene homolog [Source:MGI Symbol;Acc:MGI:95527] | 18,75382413 | 0,91659458 | **1,88765431** | 4,24472511 | 28,8691129 | 7,7438E-08 | 1,04394E-06 | protein_coding |
| ENSMUSG00000069516 | Lyz2 | lysozyme 2 [Source:MGI Symbol;Acc:MGI:96897] | 8025,586036 | 0,91643885 | **1,88745056** | 12,9704067 | 10,9290084 | 0,000946702 | 0,004895724 | protein_coding |
| ENSMUSG00000038527 | C1rl | complement component 1, r subcomponent-like [Source:MGI Symbol;Acc:MGI:2660692] | 5,140960677 | 0,91544177 | **1,88614655** | 2,36783845 | 10,4095788 | 0,001253633 | 0,006234776 | protein_coding |
| ENSMUSG00000017737 | Mmp9 | matrix metallopeptidase 9 [Source:MGI Symbol;Acc:MGI:97011] | 21,02930783 | 0,91450959 | **1,88492822** | 4,44082274 | 30,166446 | 3,9651E-08 | 5,67987E-07 | protein_coding |
| ENSMUSG00000061232 | H2-K1 | histocompatibility 2, K1, K region [Source:MGI Symbol;Acc:MGI:95904] | 727,6869558 | 0,91424769 | **1,88458608** | 9,50721555 | 25,7145087 | 3,9584E-07 | 4,59068E-06 | protein_coding |
| ENSMUSG00000019986 | Ahi1 | Abelson helper integration site 1 [Source:MGI Symbol;Acc:MGI:87971] | 5,590696153 | 0,91051117 | **1,87971139** | 2,50876073 | 10,7044023 | 0,001068809 | 0,005431548 | protein_coding |
| ENSMUSG00000022945 | Chaf1b | chromatin assembly factor 1, subunit B (p60) [Source:MGI Symbol;Acc:MGI:1314881] | 10,89120623 | 0,91002609 | **1,87907948** | 3,4497334 | 15,8674385 | 6,79376E-05 | 0,000484381 | protein_coding |
| ENSMUSG00000035900 | Gramd4 | GRAM domain containing 4 [Source:MGI Symbol;Acc:MGI:2676308] | 7,324186192 | 0,90886111 | **1,87756273** | 2,87607939 | 11,9452623 | 0,000547865 | 0,003032441 | protein_coding |
| ENSMUSG00000027078 | Ube2l6 | ubiquitin-conjugating enzyme E2L 6 [Source:MGI Symbol;Acc:MGI:1914500] | 50,05403142 | 0,90871491 | **1,87737248** | 5,65023249 | 27,2717459 | 1,76776E-07 | 2,19462E-06 | protein_coding |
| ENSMUSG00000021559 | Dapk1 | death associated protein kinase 1 [Source:MGI Symbol;Acc:MGI:1916885] | 35,20142461 | 0,90699212 | **1,87513195** | 5,14238609 | 23,853518 | 1,03952E-06 | 1,12706E-05 | protein_coding |
| ENSMUSG00000035954 | Dock4 | dedicator of cytokinesis 4 [Source:MGI Symbol;Acc:MGI:1918006] | 38,54552268 | 0,9059343 | **1,87375756** | 5,27673967 | 28,8482535 | 7,82765E-08 | 1,05395E-06 | protein_coding |
| ENSMUSG00000042460 | C1galt1 | core 1 synthase, glycoprotein-N-acetylgalactosamine 3-beta-galactosyltransferase, 1 [Source:MGI Symbol;Acc:MGI:215 | 10,09917412 | 0,90210343 | **1,86878867** | 3,33926739 | 15,5783223 | 7,91569E-05 | 0,000555484 | protein_coding |
| ENSMUSG00000033669 | Zfp7 | zinc finger protein 7 [Source:MGI Symbol;Acc:MGI:99208] | 2,545889217 | 0,90145194 | **1,86794495** | 1,36368742 | 5,95998095 | 0,014634199 | 0,049260401 | protein_coding |
| ENSMUSG00000028048 | Gba | glucosidase, beta, acid [Source:MGI Symbol;Acc:MGI:95665] | 143,2366268 | 0,89969747 | **1,86567472** | 7,1639926 | 33,2703663 | 8,01954E-09 | 1,28902E-07 | protein_coding |
| ENSMUSG00000006585 | Cdt1 | chromatin licensing and DNA replication factor 1 [Source:MGI Symbol;Acc:MGI:1914427] | 24,09919684 | 0,89837012 | **1,86395899** | 4,59244043 | 17,8016206 | 2,45174E-05 | 0,000194634 | protein_coding |
| ENSMUSG00000062382 | Gm10116 | predicted pseudogene 10116 [Source:MGI Symbol;Acc:MGI:3779109] | 4,75582358 | 0,89770961 | **1,86310581** | 2,23573728 | 8,12491594 | 0,004366101 | 0,018040748 | protein_coding |
| ENSMUSG00000032657 | Fam189b | family with sequence similarity 189, member B [Source:MGI Symbol;Acc:MGI:1915771] | 5,792996274 | 0,8971314 | **1,86235925** | 2,52642047 | 10,5149795 | 0,001184107 | 0,005924312 | protein_coding |
| ENSMUSG00000038244 | Mical2 | microtubule associated monoxygenase, calponin and LIM domain containing 2 [Source:MGI Symbol;Acc:MGI:2444947] | 27,49707397 | 0,89474043 | **1,85927533** | 4,78072439 | 20,9665478 | 4,67373E-06 | 4,40775E-05 | protein_coding |
| ENSMUSG00000036931 | Nfkbid | nuclear factor of kappa light polypeptide gene enhancer in B cells inhibitor, delta [Source:MGI Symbol;Acc:MGI:3041243 | 19,53648678 | 0,89206408 | **1,85582938** | 4,30068239 | 26,4289296 | 2,73415E-07 | 3,29387E-06 | protein_coding |
| ENSMUSG00000025498 | Irf7 | interferon regulatory factor 7 [Source:MGI Symbol;Acc:MGI:1859212] | 176,8437226 | 0,89176723 | **1,85544756** | 7,46706004 | 28,1090661 | 1,14667E-07 | 1,49095E-06 | protein_coding |
| ENSMUSG00000027330 | Cdc25b | cell division cycle 25B [Source:MGI Symbol;Acc:MGI:99701] | 8,483668621 | 0,89143663 | **1,85502243** | 3,09245164 | 11,9620879 | 0,00054294 | 0,003009728 | protein_coding |
| ENSMUSG00000062373 | Tmem65 | transmembrane protein 65 [Source:MGI Symbol;Acc:MGI:1922118] | 83,36535901 | 0,89139223 | **1,85496533** | 6,38332947 | 27,8671163 | 1,29939E-07 | 1,67209E-06 | protein_coding |
| ENSMUSG00000035769 | Xylb | xylulokinase homolog (H. influenzae) [Source:MGI Symbol;Acc:MGI:2142985] | 5,280589726 | 0,88762543 | **1,85012844** | 2,39884062 | 8,01378254 | 0,004642267 | 0,019038667 | protein_coding |
| ENSMUSG00000038650 | Rnh1 | ribonuclease/angiogenin inhibitor 1 [Source:MGI Symbol;Acc:MGI:1195456] | 321,4575305 | 0,88604147 | **1,84809826** | 8,32845768 | 26,7577887 | 2,3062E-07 | 2,79363E-06 | protein_coding |

| | | | | | | | | | | |
|---|---|---|---|---|---|---|---|---|---|---|
| ENSMUSG00000037295 | Ldlrap1 | low density lipoprotein receptor adaptor protein 1 [Source:MGI Symbol;Acc:MGI:2140175] | 67,52922531 | 0,88598644 | **1,84802777** | 6,07822026 | 27,0443827 | 1,98837E-07 | 2,43548E-06 | protein_coding |
| ENSMUSG00000021939 | Ctsb | cathepsin B [Source:MGI Symbol;Acc:MGI:88561] | 7028,320286 | 0,88513148 | **1,84693294** | 12,7789718 | 9,03479231 | 0,002648891 | 0,011876788 | protein_coding |
| ENSMUSG00000046562 | Unc119b | unc-119 homolog B (C. elegans) [Source:MGI Symbol;Acc:MGI:2147162] | 12,2140052 | 0,88182688 | **1,84270723** | 3,62356167 | 17,2481514 | 3,28017E-05 | 0,000251495 | protein_coding |
| ENSMUSG00000024521 | Pmaip1 | phorbol-12-myristate-13-acetate-induced protein 1 [Source:MGI Symbol;Acc:MGI:1930146] | 24,6704801 | 0,87919944 | **1,83935434** | 4,63381206 | 21,2137468 | 4,10807E-06 | 3,92482E-05 | protein_coding |
| ENSMUSG00000023259 | Slc26a6 | solute carrier family 26, member 6 [Source:MGI Symbol;Acc:MGI:2159728] | 5,510384321 | 0,87903236 | **1,83914135** | 2,46771426 | 9,72209092 | 0,001820663 | 0,008670837 | protein_coding |
| ENSMUSG00000031765 | Mt1 | metallothionein 1 [Source:MGI Symbol;Acc:MGI:97171] | 82,12800998 | 0,87600631 | **1,8352878** | 6,36083602 | 27,7703919 | 1,36601E-07 | 1,742E-06 | protein_coding |
| ENSMUSG00000029772 | Ahcyl2 | S-adenosylhomocysteine hydrolase-like 2 [Source:MGI Symbol;Acc:MGI:1921590] | 52,84522221 | 0,87444688 | **1,83330508** | 5,73029906 | 35,0896302 | 3,14873E-09 | 5,43094E-08 | protein_coding |
| ENSMUSG00000073411 | H2-D1 | histocompatibility 2, D region locus 1 [Source:MGI Symbol;Acc:MGI:95896] | 1569,957018 | 0,87442636 | **1,833279** | 10,6165271 | 15,2022239 | 9,65896E-05 | 0,00066038 | protein_coding |
| ENSMUSG00000026688 | Mgst3 | microsomal glutathione S-transferase 3 [Source:MGI Symbol;Acc:MGI:1913697] | 6,916268914 | 0,87415341 | **1,83293219** | 2,78827323 | 7,03069946 | 0,008012405 | 0,03019679 | protein_coding |
| ENSMUSG00000058351 | 2010107H07Rik | RIKEN cDNA 2010107H07 gene [Source:MGI Symbol;Acc:MGI:1913737] | 7,931371423 | 0,8733354 | **1,83189321** | 2,97925959 | 12,0735289 | 0,000511428 | 0,00286262 | protein_coding |
| ENSMUSG00000068566 | Myadm | myeloid-associated differentiation marker [Source:MGI Symbol;Acc:MGI:1355332] | 76,86702125 | 0,87303578 | **1,83151279** | 6,27169768 | 38,6076639 | 5,18151E-10 | 1,0332E-08 | protein_coding |
| ENSMUSG00000087598 | Zfp111 | zinc finger protein 111 [Source:MGI Symbol;Acc:MGI:1929114] | 8,160017628 | 0,87296819 | **1,83142699** | 3,04222717 | 12,8062198 | 0,000345469 | 0,002025436 | protein_coding |
| ENSMUSG00000032261 | Sh3bgrl2 | SH3 domain binding glutamic acid-rich protein like 2 [Source:MGI Symbol;Acc:MGI:1915350] | 76,33636327 | 0,87200892 | **1,83020966** | 6,25579614 | 27,8290034 | 1,32524E-07 | 1,699E-06 | protein_coding |
| ENSMUSG00000037697 | Ddhd1 | DDHD domain containing 1 [Source:MGI Symbol;Acc:MGI:2150302] | 12,09790609 | 0,87148351 | **1,82954323** | 3,60952354 | 16,9689535 | 3,79961E-05 | 0,000286916 | protein_coding |
| ENSMUSG00000017607 | Tns4 | tensin 4 [Source:MGI Symbol;Acc:MGI:2144377] | 3,544555393 | 0,86716027 | **1,82406896** | 1,85800209 | 6,85042385 | 0,008862025 | 0,032860973 | protein_coding |
| ENSMUSG00000020604 | Arsg | arylsulfatase G [Source:MGI Symbol;Acc:MGI:1921258] | 3,143568993 | 0,86558955 | **1,8220841** | 1,63246657 | 6,298082 | 0,012086868 | 0,04242761 | protein_coding |
| ENSMUSG00000028152 | Tspan5 | tetraspanin 5 [Source:MGI Symbol;Acc:MGI:1928096] | 13,85559236 | 0,8650225 | **1,82136807** | 3,80358885 | 20,1822056 | 7,04048E-06 | 6,34336E-05 | protein_coding |
| ENSMUSG00000027381 | Bcl2l11 | BCL2-like 11 (apoptosis facilitator) [Source:MGI Symbol;Acc:MGI:1197519] | 49,8138669 | 0,86494447 | **1,82126956** | 5,63860899 | 27,6886888 | 1,42493E-07 | 1,80991E-06 | protein_coding |
| ENSMUSG00000021867 | Gm9746 | predicted gene 9746 [Source:MGI Symbol;Acc:MGI:3710397] | 5,497375734 | 0,86309171 | **1,81893213** | 2,46834679 | 9,31546204 | 0,002272281 | 0,010463348 | protein_coding |
| ENSMUSG00000038495 | Otud7b | OTU domain containing 7B [Source:MGI Symbol;Acc:MGI:2654703] | 21,41195407 | 0,86202356 | **1,81758591** | 4,42420602 | 27,7694015 | 1,36671E-07 | 1,742E-06 | protein_coding |
| ENSMUSG00000029338 | Antxr2 | anthrax toxin receptor 2 [Source:MGI Symbol;Acc:MGI:1919164] | 41,46816891 | 0,86191666 | **1,81745125** | 5,37665849 | 26,1519019 | 3,15586E-07 | 3,76476E-06 | protein_coding |
| ENSMUSG00000035790 | Cep19 | centrosomal protein 19 [Source:MGI Symbol;Acc:MGI:1914244] | 6,085475509 | 0,8618342 | **1,81734736** | 2,62001904 | 8,90946503 | 0,002836964 | 0,012597353 | protein_coding |
| ENSMUSG00000037419 | Endod1 | endonuclease domain containing 1 [Source:MGI Symbol;Acc:MGI:1919196] | 8,431016211 | 0,86048529 | **1,81564895** | 3,08402126 | 13,0706239 | 0,000299963 | 0,001787254 | protein_coding |
| ENSMUSG00000019088 | Dnase1l1 | deoxyribonuclease 1-like 1 [Source:MGI Symbol;Acc:MGI:109628] | 71,31117455 | 0,86037098 | **1,8155051** | 6,16004386 | 28,1057824 | 1,14862E-07 | 1,49171E-06 | protein_coding |
| ENSMUSG00000028789 | Adc | arginine decarboxylase [Source:MGI Symbol;Acc:MGI:2442093] | 2,882793452 | 0,86006815 | **1,81512405** | 1,53902031 | 5,96832439 | 0,014565115 | 0,049072957 | protein_coding |
| ENSMUSG00000030785 | Cox6a2 | cytochrome c oxidase, subunit VI a, polypeptide 2 [Source:MGI Symbol;Acc:MGI:104649] | 3,183338185 | 0,85912673 | **1,81393999** | 1,65722492 | 5,95684781 | 0,014660229 | 0,049332906 | protein_coding |
| ENSMUSG00000042225 | Ammecr1 | Alport syndrome, mental retardation, midface hypoplasia and elliptocytosis chromosomal region gene 1 [Source:MGI Syr | 9,756415421 | 0,85899239 | **1,81377109** | 3,29433633 | 15,3343373 | 9,00645E-05 | 0,000622351 | protein_coding |
| ENSMUSG00000019979 | Apaf1 | apoptotic peptidase activating factor 1 [Source:MGI Symbol;Acc:MGI:1306796] | 47,3723614 | 0,8588064 | **1,81353728** | 5,57092683 | 28,1457398 | 1,12515E-07 | 1,4647E-06 | protein_coding |
| ENSMUSG00000022439 | Parvg | parvin, gamma [Source:MGI Symbol;Acc:MGI:2158329] | 22,98959096 | 0,85774966 | **1,81220939** | 4,51332482 | 27,6907612 | 1,42341E-07 | 1,80991E-06 | protein_coding |
| ENSMUSG00000038188 | Scarf1 | scavenger receptor class F, member 1 [Source:MGI Symbol;Acc:MGI:2449455] | 36,83613681 | 0,8573399 | **1,81169475** | 5,2106148 | 22,3543928 | 2,26694E-06 | 2,27876E-05 | protein_coding |
| ENSMUSG00000056498 | Tmem154 | transmembrane protein 154 [Source:MGI Symbol;Acc:MGI:2444725] | 4,154403824 | 0,85692833 | **1,81117799** | 2,04482965 | 6,6836952 | 0,009729872 | 0,035586584 | protein_coding |
| ENSMUSG00000052485 | Tmem171 | transmembrane protein 171 [Source:MGI Symbol;Acc:MGI:2685751] | 7,909135521 | 0,85672338 | **1,81092072** | 3,00396444 | 11,0491822 | 0,000887263 | 0,004628852 | protein_coding |
| ENSMUSG00000030894 | Tpp1 | tripeptidyl peptidase I [Source:MGI Symbol;Acc:MGI:1336194] | 291,8796983 | 0,85660065 | **1,81076666** | 8,18967425 | 24,2262399 | 8,56573E-07 | 9,48706E-06 | protein_coding |
| ENSMUSG00000024228 | Nudt12 | nudix (nucleoside diphosphate linked moiety X)-type motif 12 [Source:MGI Symbol;Acc:MGI:1915243] | 3,912340047 | 0,85579264 | **1,8097528** | 1,96398921 | 7,12820359 | 0,007588071 | 0,028807927 | protein_coding |
| ENSMUSG00000031016 | Wee1 | WEE 1 homolog 1 (S. pombe) [Source:MGI Symbol;Acc:MGI:103075] | 4,063879233 | 0,85529039 | **1,80912287** | 2,03299034 | 6,31447149 | 0,011975644 | 0,042144906 | protein_coding |
| ENSMUSG00000013076 | Amotl1 | angiomotin-like 1 [Source:MGI Symbol;Acc:MGI:1922973] | 4,783365071 | 0,85421997 | **1,80778107** | 2,29149622 | 6,84164822 | 0,008905663 | 0,033000514 | protein_coding |
| ENSMUSG00000037280 | Galnt6 | UDP-N-acetyl-alpha-D-galactosamine:polypeptide N-acetylgalactosaminyltransferase 6 [Source:MGI Symbol;Acc:MGI:18 | 143,612763 | 0,85316847 | **1,80646395** | 7,16759183 | 26,3168073 | 2,89756E-07 | 3,47549E-06 | protein_coding |
| ENSMUSG00000067212 | H2-T23 | histocompatibility 2, T region locus 23 [Source:MGI Symbol;Acc:MGI:95957] | 122,7656637 | 0,85245102 | **1,80556583** | 6,94062614 | 37,5073016 | 9,10714E-10 | 1,7265E-08 | protein_coding |
| ENSMUSG00000070780 | Rbm47 | RNA binding motif protein 47 [Source:MGI Symbol;Acc:MGI:2384294] | 62,51290872 | 0,85217733 | **1,80522333** | 5,96733258 | 27,60657 | 1,48673E-07 | 1,86256E-06 | protein_coding |
| ENSMUSG00000032497 | Lrrfip2 | leucine rich repeat (in FLII) interacting protein 2 [Source:MGI Symbol;Acc:MGI:1918518] | 47,28757064 | 0,85155473 | **1,80444446** | 5,56735284 | 30,3902815 | 3,53294E-08 | 5,12089E-07 | protein_coding |
| ENSMUSG00000081723 | Gm15931 | predicted gene 15931 [Source:MGI Symbol;Acc:MGI:3805553] | 4,477486978 | 0,85048963 | **1,80311278** | 2,16491263 | 7,51454684 | 0,006120268 | 0,024024075 | pseudogene |
| ENSMUSG00000027217 | Tspan18 | tetraspanin 18 [Source:MGI Symbol;Acc:MGI:1917186] | 4,569354997 | 0,84850312 | **1,8006317** | 2,19468558 | 6,58051203 | 0,010310123 | 0,037335966 | protein_coding |
| ENSMUSG00000016256 | Ctsz | cathepsin Z [Source:MGI Symbol;Acc:MGI:1891190] | 1065,998198 | 0,8470971 | **1,79887769** | 10,0581007 | 17,2755979 | 3,23312E-05 | 0,000248582 | protein_coding |
| ENSMUSG00000066621 | Tecpr1 | tectonin beta-propeller repeat containing 1 [Source:MGI Symbol;Acc:MGI:1917631] | 104,1392862 | 0,84581022 | **1,79727382** | 6,70169998 | 30,5047361 | 3,33052E-08 | 4,8725E-07 | protein_coding |
| ENSMUSG00000041801 | Phlda3 | pleckstrin homology-like domain, family A, member 3 [Source:MGI Symbol;Acc:MGI:1351485] | 29,93361891 | 0,84460983 | **1,79577902** | 4,9026931 | 15,2549936 | 9,39281E-05 | 0,00064459 | protein_coding |
| ENSMUSG00000024190 | Dusp1 | dual specificity phosphatase 1 [Source:MGI Symbol;Acc:MGI:105120] | 44,05395124 | 0,84407714 | **1,79511608** | 5,47485514 | 27,2597444 | 1,77876E-07 | 2,20579E-06 | protein_coding |
| ENSMUSG00000031586 | Rbpms | RNA binding protein gene with multiple splicing [Source:MGI Symbol;Acc:MGI:1334446] | 5,895035469 | 0,84289835 | **1,79364994** | 2,57641099 | 8,43064783 | 0,003689489 | 0,015776345 | protein_coding |
| ENSMUSG00000040511 | Pvr | poliovirus receptor [Source:MGI Symbol;Acc:MGI:107741] | 33,4538421 | 0,84199344 | **1,79252525** | 5,07460958 | 25,7223541 | 3,94234E-07 | 4,5787E-06 | protein_coding |
| ENSMUSG00000015980 | Lrrc27 | leucine rich repeat containing 27 [Source:MGI Symbol;Acc:MGI:1923862] | 5,798506174 | 0,84058657 | **1,79077809** | 2,53855162 | 7,59443741 | 0,005854866 | 0,0231727 | protein_coding |
| ENSMUSG00000047821 | Trim16 | tripartite motif-containing 16 [Source:MGI Symbol;Acc:MGI:2137356] | 4,420855331 | 0,83882257 | **1,78858981** | 2,1387553 | 7,0265912 | 0,008030808 | 0,030238001 | protein_coding |
| ENSMUSG00000044786 | Zfp36 | zinc finger protein 36 [Source:MGI Symbol;Acc:MGI:99180] | 130,2593519 | 0,83744334 | **1,78688072** | 7,02777266 | 36,6205884 | 1,43508E-09 | 2,63666E-08 | protein_coding |
| ENSMUSG00000029403 | Cdkl2 | cyclin-dependent kinase-like 2 (CDC2-related kinase) [Source:MGI Symbol;Acc:MGI:1858227] | 6,421024021 | 0,8363874 | **1,78557335** | 2,69071126 | 6,11102093 | 0,013434151 | 0,046096506 | protein_coding |
| ENSMUSG00000028641 | Lepre1 | leprecan 1 [Source:MGI Symbol;Acc:MGI:1888921] | 7,703760735 | 0,83309741 | **1,78150608** | 2,95785285 | 10,7754301 | 0,001028563 | 0,005258643 | protein_coding |
| ENSMUSG00000048458 | 6530418L21Rik | RIKEN cDNA 6530418L21 gene [Source:MGI Symbol;Acc:MGI:1923497] | 5,957827339 | 0,83215788 | **1,78034629** | 2,58142343 | 8,76369969 | 0,003072851 | 0,013499165 | protein_coding |
| ENSMUSG00000038352 | Arl5c | ADP-ribosylation factor-like 5C [Source:MGI Symbol;Acc:MGI:3028577] | 24,62927305 | 0,83214877 | **1,78033504** | 4,64619212 | 29,0175671 | 7,17249E-08 | 9,76508E-07 | protein_coding |
| ENSMUSG00000063354 | Slc39a4 | solute carrier family 39 (zinc transporter), member 4 [Source:MGI Symbol;Acc:MGI:1919277] | 59,83161942 | 0,82664839 | **1,77356032** | 5,90683193 | 27,9507064 | 1,24446E-07 | 1,60668E-06 | protein_coding |
| ENSMUSG00000000275 | Trim25 | tripartite motif-containing 25 [Source:MGI Symbol;Acc:MGI:102749] | 243,8204166 | 0,82652865 | **1,77341311** | 7,93026238 | 27,247994 | 1,78961E-07 | 2,21673E-06 | protein_coding |
| ENSMUSG00000024743 | Syt7 | synaptotagmin VII [Source:MGI Symbol;Acc:MGI:1859545] | 11,36005944 | 0,82384435 | **1,77011655** | 3,54803053 | 13,4760552 | 0,000241627 | 0,001480624 | protein_coding |
| ENSMUSG00000061731 | Ext1 | exostoses (multiple) 1 [Source:MGI Symbol;Acc:MGI:894663] | 15,52356426 | 0,82270236 | **1,76871593** | 3,97848601 | 19,9723822 | 7,85688E-06 | 6,98976E-05 | protein_coding |
| ENSMUSG00000054364 | Rhob | ras homolog gene family, member B [Source:MGI Symbol;Acc:MGI:107949] | 146,0586818 | 0,82244039 | **1,76839479** | 7,19029073 | 31,3284493 | 2,17864E-08 | 3,26558E-07 | protein_coding |
| ENSMUSG00000026701 | Prdx6 | peroxiredoxin 6 [Source:MGI Symbol;Acc:MGI:894320] | 145,7200288 | 0,82177321 | **1,76757718** | 7,1918052 | 37,1553892 | 1,09081E-09 | 2,04517E-08 | protein_coding |
| ENSMUSG00000090213 | Tmem189 | transmembrane protein 189 [Source:MGI Symbol;Acc:MGI:2142624] | 169,4850794 | 0,82175147 | **1,76755054** | 7,40554474 | 31,8982041 | 1,62467E-08 | 2,48613E-07 | protein_coding |
| ENSMUSG00000021263 | Degs2 | degenerative spermatocyte homolog 2 (Drosophila), lipid desaturase [Source:MGI Symbol;Acc:MGI:1917309] | 14,95708039 | 0,8207097 | **1,76627465** | 3,9086365 | 20,8489727 | 4,96959E-06 | 4,6548E-05 | protein_coding |
| ENSMUSG00000031304 | Il2rg | interleukin 2 receptor, gamma chain [Source:MGI Symbol;Acc:MGI:96551] | 106,593443 | 0,82058998 | **1,76612809** | 6,74035806 | 39,6149038 | 3,09314E-10 | 6,41213E-09 | protein_coding |
| ENSMUSG00000037148 | Arhgap10 | Rho GTPase activating protein 10 [Source:MGI Symbol;Acc:MGI:1925764] | 64,20175597 | 0,81858084 | **1,76367024** | 6,00840838 | 23,2976428 | 1,3877E-06 | 1,45901E-05 | protein_coding |
| ENSMUSG00000004707 | Ly9 | lymphocyte antigen 9 [Source:MGI Symbol;Acc:MGI:96885] | 446,700498 | 0,81766424 | **1,76255006** | 8,80364684 | 19,4483315 | 1,03358E-05 | 8,92764E-05 | protein_coding |
| ENSMUSG00000021556 | Golm1 | golgi membrane protein 1 [Source:MGI Symbol;Acc:MGI:1917329] | 11,39179992 | 0,81583491 | **1,76031658** | 3,51958015 | 15,3875286 | 8,75643E-05 | 0,000607367 | protein_coding |
| ENSMUSG00000027698 | Nceh1 | arylacetamide deacetylase-like 1 [Source:MGI Symbol;Acc:MGI:2443191] | 175,8683178 | 0,81495988 | **1,75924923** | 7,45904521 | 21,2111011 | 4,11375E-06 | 3,92683E-05 | protein_coding |
| ENSMUSG00000020611 | Gna13 | guanine nucleotide binding protein, alpha 13 [Source:MGI Symbol;Acc:MGI:95768] | 351,40375 | 0,81361218 | **1,75760658** | 8,45764184 | 27,7694333 | 1,36668E-07 | 1,742E-06 | protein_coding |
| ENSMUSG00000021236 | Entpd5 | ectonucleoside triphosphate diphosphohydrolase 5 [Source:MGI Symbol;Acc:MGI:1321385] | 14,88185074 | 0,8129553 | **1,75680651** | 3,9099722 | 14,4031431 | 0,000147556 | 0,000959855 | protein_coding |
| ENSMUSG00000022708 | Zbtb20 | zinc finger and BTB domain containing 20 [Source:MGI Symbol;Acc:MGI:1929213] | 28,61712541 | 0,81268799 | **1,75648103** | 4,83910746 | 21,0340202 | 4,512E-06 | 4,26621E-05 | protein_coding |

| | | | | | | | | | | |
|---|---|---|---|---|---|---|---|---|---|---|
| ENSMUSG00000041431 | Ccnb1 | cyclin B1 [Source:MGI Symbol;Acc:MGI:88302] | 12,40313027 | 0,81031147 | **1,75359** | 3,64621502 | 9,8410587 | 0,001706592 | 0,008205834 | protein_coding |
| ENSMUSG00000061981 | Flot2 | flotillin 2 [Source:MGI Symbol;Acc:MGI:103309] | 62,47761723 | 0,80960626 | **1,75273302** | 5,97016339 | 30,2090168 | 3,87902E-08 | 5,57108E-07 | protein_coding |
| ENSMUSG00000021215 | Net1 | neuroepithelial cell transforming gene 1 [Source:MGI Symbol;Acc:MGI:1927138] | 6,473521505 | 0,80823689 | **1,75107017** | 2,71902683 | 8,7126637 | 0,003160071 | 0,013788604 | protein_coding |
| ENSMUSG00000031337 | Mtm1 | X-linked myotubular myopathy gene 1 [Source:MGI Symbol;Acc:MGI:1099452] | 24,3031305 | 0,80766486 | **1,750376** | 4,60810617 | 26,1031505 | 3,23655E-07 | 3,84018E-06 | protein_coding |
| ENSMUSG00000038024 | Dennd4c | DENN/MADD domain containing 4C [Source:MGI Symbol;Acc:MGI:1914769] | 94,73263 | 0,80607409 | **1,74844703** | 6,56765108 | 23,5731085 | 1,20256E-06 | 1,27781E-05 | protein_coding |
| ENSMUSG00000052512 | Nav2 | neuron navigator 2 [Source:MGI Symbol;Acc:MGI:2183691] | 33,87014542 | 0,80543884 | **1,74767732** | 5,08342489 | 9,59430024 | 0,001951823 | 0,009184017 | protein_coding |
| ENSMUSG00000030095 | Tmem43 | transmembrane protein 43 [Source:MGI Symbol;Acc:MGI:1921372] | 66,47018135 | 0,80534749 | **1,74756667** | 6,05788143 | 29,365845 | 5,99245E-08 | 8,28165E-07 | protein_coding |
| ENSMUSG00000021262 | Evl | Ena-vasodilator stimulated phosphoprotein [Source:MGI Symbol;Acc:MGI:1194884] | 137,622436 | 0,80520042 | **1,74738853** | 7,10645593 | 35,5841171 | 2,44271E-09 | 4,3148E-08 | protein_coding |
| ENSMUSG00000003032 | Klf4 | Kruppel-like factor 4 (gut) [Source:MGI Symbol;Acc:MGI:1342287] | 11,44925105 | 0,8034922 | **1,74532076** | 3,52125291 | 15,6782135 | 7,50841E-05 | 0,000531776 | protein_coding |
| ENSMUSG00000032737 | Inppl1 | inositol polyphosphate phosphatase-like 1 [Source:MGI Symbol;Acc:MGI:1333787] | 165,6027954 | 0,80328578 | **1,74507105** | 7,37276013 | 31,644 | 1,85186E-08 | 2,82588E-07 | protein_coding |
| ENSMUSG00000000489 | Pdgfb | platelet derived growth factor, B polypeptide [Source:MGI Symbol;Acc:MGI:97528] | 22,29283378 | 0,80287237 | **1,74457106** | 4,47712823 | 18,5181785 | 1,68292E-05 | 0,000139549 | protein_coding |
| ENSMUSG00000028007 | Snx7 | sorting nexin 7 [Source:MGI Symbol;Acc:MGI:1923811] | 12,29986656 | 0,80220149 | **1,74376** | 3,62727677 | 13,8649752 | 0,000196425 | 0,001233919 | protein_coding |
| ENSMUSG00000045658 | Pid1 | phosphotyrosine interaction domain containing 1 [Source:MGI Symbol;Acc:MGI:2138391] | 145,8136966 | 0,80113386 | **1,74247005** | 7,19227854 | 35,4678946 | 2,59289E-09 | 4,5654E-08 | protein_coding |
| ENSMUSG00000018507 | Trpv2 | transient receptor potential cation channel, subfamily V, member 2 [Source:MGI Symbol;Acc:MGI:1341836] | 112,0095394 | 0,801028 | **1,7423422** | 6,80944908 | 29,7092997 | 5,01934E-08 | 7,06114E-07 | protein_coding |
| ENSMUSG00000048779 | P2ry6 | pyrimidinergic receptor P2Y, G-protein coupled, 6 [Source:MGI Symbol;Acc:MGI:2673874] | 105,5504811 | 0,79879482 | **1,73964728** | 6,72333446 | 37,5058091 | 9,11411E-10 | 1,7265E-08 | protein_coding |
| ENSMUSG00000042724 | Map3k9 | mitogen-activated protein kinase kinase kinase 9 [Source:MGI Symbol;Acc:MGI:2449952] | 8,312926041 | 0,79499137 | **1,73506699** | 3,0635843 | 8,50286345 | 0,00354588 | 0,015218198 | protein_coding |
| ENSMUSG00000032120 | C2cd2l | C2 calcium-dependent domain containing 2-like [Source:MGI Symbol;Acc:MGI:1919014] | 16,29604201 | 0,79497849 | **1,7350515** | 4,02108698 | 21,4158653 | 3,69699E-06 | 3,56619E-05 | protein_coding |
| ENSMUSG00000042766 | Trim46 | tripartite motif-containing 46 [Source:MGI Symbol;Acc:MGI:2673000] | 4,150197611 | 0,79486561 | **1,73491575** | 2,04795091 | 6,20324158 | 0,012751656 | 0,044238222 | protein_coding |
| ENSMUSG00000095028 | Sirpb1b | signal-regulatory protein beta 1B [Source:MGI Symbol;Acc:MGI:3779828] | 10,42956707 | 0,79464743 | **1,7346534** | 3,3823041 | 11,6405145 | 0,000645308 | 0,003508161 | protein_coding |
| ENSMUSG00000085894 | Gm15832 | predicted gene 15832 [Source:MGI Symbol;Acc:MGI:3834078] | 6,966374702 | 0,79417159 | **1,73408136** | 2,80201138 | 8,9006185 | 0,002850741 | 0,012649874 | rocessed_transcrip |
| ENSMUSG00000021669 | Col4a3bp | collagen, type IV, alpha 3 (Goodpasture antigen) binding protein [Source:MGI Symbol;Acc:MGI:1915268] | 69,45463488 | 0,79323747 | **1,73295894** | 6,12024363 | 31,0127978 | 2,56333E-08 | 3,79559E-07 | protein_coding |
| ENSMUSG00000063160 | Numbl | numb-like [Source:MGI Symbol;Acc:MGI:894702] | 11,105239 | 0,79231934 | **1,73185643** | 3,46881751 | 12,8434493 | 0,000338663 | 0,001990844 | protein_coding |
| ENSMUSG00000032192 | Gnb5 | guanine nucleotide binding protein (G protein), beta 5 [Source:MGI Symbol;Acc:MGI:101848] | 4,125722005 | 0,79160309 | **1,73099684** | 2,05591517 | 6,33102099 | 0,0118644 | 0,041833812 | protein_coding |
| ENSMUSG00000042599 | Jhdm1d | jumonji C domain-containing histone demethylase 1 homolog D (S. cerevisiae) [Source:MGI Symbol;Acc:MGI:2443388] | 48,59154018 | 0,7905979 | **1,72979119** | 5,60691001 | 32,2083997 | 1,38491E-08 | 2,15524E-07 | protein_coding |
| ENSMUSG00000029217 | Tec | tec protein tyrosine kinase [Source:MGI Symbol;Acc:MGI:98662] | 40,14993352 | 0,78974056 | **1,72876355** | 5,32635634 | 24,3520384 | 8,02418E-07 | 8,94134E-06 | protein_coding |
| ENSMUSG00000026640 | Plxna2 | plexin A2 [Source:MGI Symbol;Acc:MGI:107684] | 13,68842621 | 0,78948834 | **1,72846135** | 3,78113075 | 11,8057728 | 0,000590473 | 0,003245389 | protein_coding |
| ENSMUSG00000034993 | Vat1 | vesicle amine transport protein 1 homolog (T californica) [Source:MGI Symbol;Acc:MGI:1349450] | 564,6533162 | 0,78895661 | **1,72782441** | 9,14138572 | 16,2919641 | 5,42937E-05 | 0,000397366 | protein_coding |
| ENSMUSG00000030980 | 2310008H09Rik | RIKEN cDNA 2310008H09 gene [Source:MGI Symbol;Acc:MGI:1913606] | 39,25674869 | 0,78864148 | **1,72744704** | 5,3003741 | 21,7468812 | 3,11095E-06 | 3,04907E-05 | protein_coding |
| ENSMUSG00000014361 | Mertk | c-mer proto-oncogene tyrosine kinase [Source:MGI Symbol;Acc:MGI:96965] | 42,30999353 | 0,78498346 | **1,72307257** | 5,40764474 | 17,0672873 | 3,60784E-05 | 0,000273375 | protein_coding |
| ENSMUSG00000026566 | Mpzl1 | myelin protein zero-like 1 [Source:MGI Symbol;Acc:MGI:1915731] | 10,00796543 | 0,78453452 | **1,72253647** | 3,34250661 | 11,740558 | 0,000611527 | 0,003347706 | protein_coding |
| ENSMUSG00000028693 | Nasp | nuclear autoantigenic sperm protein (histone-binding) [Source:MGI Symbol;Acc:MGI:1355328] | 20,41489391 | 0,78416271 | **1,72209259** | 4,36622829 | 19,6625262 | 9,23956E-06 | 8,08241E-05 | protein_coding |
| ENSMUSG00000027663 | Zmat3 | zinc finger matrin type 3 [Source:MGI Symbol;Acc:MGI:1195270] | 75,59055575 | 0,78335539 | **1,72112919** | 6,24175536 | 17,5176403 | 2,84654E-05 | 0,000221966 | protein_coding |
| ENSMUSG00000059326 | Csf2ra | colony stimulating factor 2 receptor, alpha, low-affinity (granulocyte-macrophage) [Source:MGI Symbol;Acc:MGI:133975 | 156,8207301 | 0,78201511 | **1,71953099** | 7,29220664 | 26,6042697 | 2,49691E-07 | 3,01468E-06 | protein_coding |
| ENSMUSG00000035352 | Ccl12 | chemokine (C-C motif) ligand 12 [Source:MGI Symbol;Acc:MGI:108224] | 5,334817832 | 0,78150173 | **1,7189192** | 2,47246904 | 6,38026556 | 0,011539616 | 0,040898634 | protein_coding |
| ENSMUSG00000074063 | Osgin1 | oxidative stress induced growth inhibitor 1 [Source:MGI Symbol;Acc:MGI:1919089] | 21,28481259 | 0,78086539 | **1,71816119** | 4,41648526 | 16,2044857 | 5,68593E-05 | 0,000412897 | protein_coding |
| ENSMUSG00000026335 | Pam | peptidylglycine alpha-amidating monooxygenase [Source:MGI Symbol;Acc:MGI:97475] | 30,46566964 | 0,78059091 | **1,71783433** | 4,9377867 | 22,7659452 | 1,8298E-06 | 1,88593E-05 | protein_coding |
| ENSMUSG00000030591 | Psmd8 | proteasome (prosome, macropain) 26S subunit, non-ATPase, 8 [Source:MGI Symbol;Acc:MGI:1888669] | 211,1316913 | 0,77921168 | **1,71619286** | 7,72224177 | 26,409411 | 2,76192E-07 | 3,32004E-06 | protein_coding |
| ENSMUSG00000020829 | Slc46a1 | solute carrier family 46, member 1 [Source:MGI Symbol;Acc:MGI:1098733] | 14,16644818 | 0,77801552 | **1,71477053** | 3,82384772 | 16,6494689 | 4,49629E-05 | 0,000333563 | protein_coding |
| ENSMUSG00000042073 | Abhd14b | abhydrolase domain containing 14b [Source:MGI Symbol;Acc:MGI:1923741] | 7,366809591 | 0,77785856 | **1,71458397** | 2,89445578 | 9,13652149 | 0,00250557 | 0,01134417 | protein_coding |
| ENSMUSG00000024300 | Myo1f | myosin IF [Source:MGI Symbol;Acc:MGI:107711] | 299,55911 | 0,77707987 | **1,71365878** | 8,22658571 | 23,7746077 | 1,08302E-06 | 1,16658E-05 | protein_coding |
| ENSMUSG00000047766 | Lrrc49 | leucine rich repeat containing 49 [Source:MGI Symbol;Acc:MGI:2442689] | 7,152062451 | 0,77688252 | **1,71342437** | 2,84320347 | 9,0345659 | 0,00264922 | 0,011876788 | protein_coding |
| ENSMUSG00000048234 | Rnf149 | ring finger protein 149 [Source:MGI Symbol;Acc:MGI:2677438] | 191,8629691 | 0,77541848 | **1,71168648** | 7,58556045 | 28,6756327 | 8,55737E-08 | 1,13551E-06 | protein_coding |
| ENSMUSG00000042303 | Sgsm3 | small G protein signaling modulator 3 [Source:MGI Symbol;Acc:MGI:1916329] | 19,41119751 | 0,77280193 | **1,7085849** | 4,27869543 | 20,2028747 | 6,96482E-06 | 6,29297E-05 | protein_coding |
| ENSMUSG00000027035 | Lass6 | LAG1 homolog, ceramide synthase 6 [Source:MGI Symbol;Acc:MGI:2442564] | 49,0973745 | 0,77106352 | **1,70652733** | 5,62160627 | 27,2117714 | 1,82345E-07 | 2,25399E-06 | protein_coding |
| ENSMUSG00000040918 | Slc19a2 | solute carrier family 19 (thiamine transporter), member 2 [Source:MGI Symbol;Acc:MGI:1928761] | 16,51987393 | 0,77074752 | **1,70615359** | 4,05428934 | 12,487867 | 0,000409604 | 0,002357416 | protein_coding |
| ENSMUSG00000003153 | Slc2a3 | solute carrier family 2 (facilitated glucose transporter), member 3 [Source:MGI Symbol;Acc:MGI:95757] | 7,016406533 | 0,76654005 | **1,70118501** | 2,83104666 | 9,34374815 | 0,002237474 | 0,010316043 | protein_coding |
| ENSMUSG00000044847 | Lsm11 | U7 snRNP-specific Sm-like protein LSM11 [Source:MGI Symbol;Acc:MGI:1919540] | 8,517557703 | 0,76649729 | **1,70113459** | 3,09831461 | 11,299058 | 0,000775464 | 0,00412992 | protein_coding |
| ENSMUSG00000039782 | Cpeb2 | cytoplasmic polyadenylation element binding protein 2 [Source:MGI Symbol;Acc:MGI:2442640] | 47,36197588 | 0,76597323 | **1,70051676** | 5,5688359 | 19,8894739 | 8,20509E-06 | 7,28185E-05 | protein_coding |
| ENSMUSG00000063229 | Ldha | lactate dehydrogenase A [Source:MGI Symbol;Acc:MGI:96759] | 583,5984732 | 0,7654587 | **1,69991039** | 9,18924836 | 18,8118046 | 1,44271E-05 | 0,000121371 | protein_coding |
| ENSMUSG00000047557 | Lxn | latexin [Source:MGI Symbol;Acc:MGI:107633] | 9,118798737 | 0,7640312 | **1,69822922** | 3,19777996 | 10,9105348 | 0,000956189 | 0,004939187 | protein_coding |
| ENSMUSG00000019699 | Akt3 | thymoma viral proto-oncogene 3 [Source:MGI Symbol;Acc:MGI:1345147] | 23,26143662 | 0,7636849 | **1,69782163** | 4,5469431 | 19,0948684 | 1,24379E-05 | 0,000105607 | protein_coding |
| ENSMUSG00000018395 | Kif3a | kinesin family member 3A [Source:MGI Symbol;Acc:MGI:107689] | 25,99872048 | 0,76367234 | **1,69780685** | 4,70850182 | 23,4886257 | 1,25654E-06 | 1,32747E-05 | protein_coding |
| ENSMUSG00000079884 | Gm10698 | predicted gene 10698 [Source:MGI Symbol;Acc:MGI:3704329] | 24,59001193 | 0,76313545 | **1,69717514** | 4,64001866 | 12,9572001 | 0,000318693 | 0,001883531 | ocessed_pseudoge |
| ENSMUSG00000031669 | Gins3 | GINS complex subunit 3 (Psf3 homolog) [Source:MGI Symbol;Acc:MGI:1926083] | 5,043947452 | 0,76219207 | **1,69606572** | 2,35233059 | 6,90060001 | 0,008616683 | 0,032059428 | protein_coding |
| ENSMUSG00000038141 | Tmem181a | transmembrane protein 181A [Source:MGI Symbol;Acc:MGI:1924356] | 28,41903148 | 0,76212469 | **1,6959865** | 4,84016643 | 21,456894 | 3,61873E-06 | 3,49991E-05 | protein_coding |
| ENSMUSG00000079547 | H2-DMb1 | histocompatibility 2, class II, locus Mb1 [Source:MGI Symbol;Acc:MGI:95922] | 16,34801052 | 0,76206698 | **1,69591867** | 4,03593129 | 17,1114562 | 3,5249E-05 | 0,000267459 | protein_coding |
| ENSMUSG00000032076 | Cadm1 | cell adhesion molecule 1 [Source:MGI Symbol;Acc:MGI:1889272] | 38,37553394 | 0,76105306 | **1,6947272** | 5,26023977 | 15,5676857 | 7,96035E-05 | 0,000558139 | protein_coding |
| ENSMUSG00000036718 | Micall2 | MICAL-like 2 [Source:MGI Symbol;Acc:MGI:2444818] | 32,29540435 | 0,76033052 | **1,69387864** | 5,01939385 | 21,6510493 | 3,27031E-06 | 3,18536E-05 | protein_coding |
| ENSMUSG00000048787 | Dcun1d3 | DCN1, defective in cullin neddylation 1, domain containing 3 (S. cerevisiae) [Source:MGI Symbol;Acc:MGI:2679003] | 22,20547588 | 0,76006869 | **1,69357126** | 4,48630707 | 19,234791 | 1,15588E-05 | 9,87528E-05 | protein_coding |
| ENSMUSG00000066900 | Suds3 | suppressor of defective silencing 3 homolog (S. cerevisiae) [Source:MGI Symbol;Acc:MGI:1919204] | 47,22718828 | 0,75961259 | **1,69303593** | 5,56610887 | 25,9113457 | 3,57462E-07 | 4,18703E-06 | protein_coding |
| ENSMUSG00000079065 | BC005561 | cDNA sequence BC005561 [Source:MGI Symbol;Acc:MGI:3040669] | 8,501652196 | 0,75823441 | **1,69141937** | 3,08684468 | 10,6536417 | 0,001098544 | 0,005562075 | protein_coding |
| ENSMUSG00000092341 | Malat1 | metastasis associated lung adenocarcinoma transcript 1 (non-coding RNA) [Source:MGI Symbol;Acc:MGI:1919539] | 783,4868167 | 0,75780359 | **1,69091436** | 9,61372035 | 14,5704922 | 0,000135012 | 0,000886659 | lincRNA |
| ENSMUSG00000021639 | Gtf2h2 | general transcription factor II H, polypeptide 2 [Source:MGI Symbol;Acc:MGI:1345669] | 15,44016567 | 0,75473661 | **1,68732351** | 3,95500593 | 14,3345472 | 0,000153031 | 0,000993117 | protein_coding |
| ENSMUSG00000021140 | Pcnx | pecanex homolog (Drosophila) [Source:MGI Symbol;Acc:MGI:1891924] | 58,68572732 | 0,75213722 | **1,6842861** | 5,8774379 | 22,6313522 | 1,96256E-06 | 2,00396E-05 | protein_coding |
| ENSMUSG00000039242 | B3galnt2 | UDP-GalNAc:betaGlcNAc beta 1,3-galactosaminyltransferase, polypeptide 2 [Source:MGI Symbol;Acc:MGI:2145517] | 12,93809267 | 0,75167243 | **1,68374357** | 3,70245637 | 12,3546312 | 0,000439894 | 0,002504204 | protein_coding |
| ENSMUSG00000032294 | Pkm | pyruvate kinase, muscle [Source:MGI Symbol;Acc:MGI:97591] | 2520,143097 | 0,75043916 | **1,68230485** | 11,2993287 | 9,73925136 | 0,001803743 | 0,008616404 | protein_coding |
| ENSMUSG00000035778 | Ggta1 | glycoprotein galactosyltransferase alpha 1, 3 [Source:MGI Symbol;Acc:MGI:95704] | 216,9052895 | 0,74953957 | **1,68125618** | 7,76271388 | 27,1596968 | 1,87323E-07 | 2,30731E-06 | protein_coding |
| ENSMUSG00000036934 | 4921524J17Rik | RIKEN cDNA 4921524J17 gene [Source:MGI Symbol;Acc:MGI:1913964] | 9,293366623 | 0,74861744 | **1,68018192** | 3,22935052 | 9,70993681 | 0,001832743 | 0,008720809 | protein_coding |
| ENSMUSG00000024646 | Cyb5 | cytochrome b-5 [Source:MGI Symbol;Acc:MGI:1926952] | 206,6176764 | 0,74833542 | **1,6798535** | 7,69215693 | 23,3099171 | 1,37887E-06 | 1,45112E-05 | protein_coding |
| ENSMUSG00000027610 | Gss | glutathione synthetase [Source:MGI Symbol;Acc:MGI:95852] | 25,35527842 | 0,74740798 | **1,67877394** | 4,67107913 | 22,0101583 | 2,71211E-06 | 2,69385E-05 | protein_coding |
| ENSMUSG00000030652 | Coq7 | demethyl-Q 7 [Source:MGI Symbol;Acc:MGI:107207] | 14,99678405 | 0,74642919 | **1,67763537** | 3,91503963 | 13,4374431 | 0,000246652 | 0,001508047 | protein_coding |

| | | | | | | | | | | |
|---|---|---|---|---|---|---|---|---|---|---|
| ENSMUSG00000037622 | Wdtc1 | WD and tetratricopeptide repeats 1 [Source:MGI Symbol;Acc:MGI:2685541] | 39,95333737 | 0,74478495 | **1,67572447** | 5,32306777 | 22,5406359 | 2,05745E-06 | 2,08728E-05 | protein_coding |
| ENSMUSG00000020315 | Spnb2 | spectrin beta 2 [Source:MGI Symbol;Acc:MGI:98388] | 79,72746885 | 0,74440745 | **1,67528605** | 6,31996222 | 21,8698661 | 2,9178E-06 | 2,87E-05 | protein_coding |
| ENSMUSG00000031153 | Gripap1 | GRIP1 associated protein 1 [Source:MGI Symbol;Acc:MGI:1859616] | 64,75106703 | 0,74312704 | **1,67379987** | 6,01795351 | 29,4033302 | 5,87766E-08 | 8,16503E-07 | protein_coding |
| ENSMUSG00000018986 | Slfn3 | schlafen 3 [Source:MGI Symbol;Acc:MGI:1329005] | 7,436928438 | 0,74242715 | **1,67298806** | 2,90762683 | 7,90477442 | 0,004930449 | 0,01995978 | protein_coding |
| ENSMUSG00000028016 | Ints12 | integrator complex subunit 12 [Source:MGI Symbol;Acc:MGI:1919043] | 32,07498768 | 0,74210676 | **1,67261656** | 5,00931209 | 26,2151767 | 3,05413E-07 | 3,65133E-06 | protein_coding |
| ENSMUSG00000069910 | Ccdc99 | coiled-coil domain containing 99 [Source:MGI Symbol;Acc:MGI:1917635] | 11,14131511 | 0,74183859 | **1,67230569** | 3,48561951 | 9,87369273 | 0,001676587 | 0,008075694 | protein_coding |
| ENSMUSG00000003382 | Etv3 | ets variant gene 3 [Source:MGI Symbol;Acc:MGI:1350926] | 127,0598569 | 0,74084464 | **1,67115394** | 6,99036712 | 31,4803318 | 2,01471E-08 | 3,0406E-07 | protein_coding |
| ENSMUSG00000025701 | Alox5 | arachidonate 5-lipoxygenase [Source:MGI Symbol;Acc:MGI:87999] | 6,740074352 | 0,73986798 | **1,67002301** | 2,75662366 | 7,82546652 | 0,005151515 | 0,020785785 | protein_coding |
| ENSMUSG00000045594 | Glb1 | galactosidase, beta 1 [Source:MGI Symbol;Acc:MGI:88151] | 103,1145474 | 0,73975187 | **1,66988861** | 6,69044052 | 27,2003821 | 1,83422E-07 | 2,26434E-06 | protein_coding |
| ENSMUSG00000073542 | Cep76 | centrosomal protein 76 [Source:MGI Symbol;Acc:MGI:1923401] | 5,93696076 | 0,73966044 | **1,66978279** | 2,56867048 | 7,55257884 | 0,005992426 | 0,023618767 | protein_coding |
| ENSMUSG00000024589 | Nedd4l | neural precursor cell expressed, developmentally down-regulated gene 4-like [Source:MGI Symbol;Acc:MGI:1933754] | 25,06445996 | 0,73961428 | **1,66972936** | 4,64782967 | 18,2229145 | 1,965E-05 | 0,000159922 | protein_coding |
| ENSMUSG00000046794 | Ppp1r3b | protein phosphatase 1, regulatory (inhibitor) subunit 3B [Source:MGI Symbol;Acc:MGI:2177268] | 14,01188964 | 0,73889111 | **1,66889259** | 3,81193502 | 16,2321268 | 5,60357E-05 | 0,000408537 | protein_coding |
| ENSMUSG00000038543 | BC028528 | cDNA sequence BC028528 [Source:MGI Symbol;Acc:MGI:2385885] | 14,07906175 | 0,7386862 | **1,66865557** | 3,8235207 | 15,6773718 | 7,51175E-05 | 0,000531776 | protein_coding |
| ENSMUSG00000036887 | C1qa | complement component 1, q subcomponent, alpha polypeptide [Source:MGI Symbol;Acc:MGI:88223] | 560,1323141 | 0,73807211 | **1,66794545** | 9,12967719 | 18,5608254 | 1,64568E-05 | 0,000136771 | protein_coding |
| ENSMUSG00000032411 | Tfdp2 | transcription factor Dp 2 [Source:MGI Symbol;Acc:MGI:107167] | 7,138263216 | 0,73797519 | **1,66783341** | 2,84761053 | 8,98359816 | 0,002724137 | 0,012171652 | protein_coding |
| ENSMUSG00000028527 | Ak4 | adenylate kinase 4 [Source:MGI Symbol;Acc:MGI:87979] | 9,818003725 | 0,73735259 | **1,6671138** | 3,31587502 | 10,2132561 | 0,001394348 | 0,006836099 | protein_coding |
| ENSMUSG00000004609 | Cd33 | CD33 antigen [Source:MGI Symbol;Acc:MGI:99440] | 9,486123271 | 0,73630725 | **1,66590629** | 3,2354524 | 11,5680651 | 0,000670942 | 0,003634931 | protein_coding |
| ENSMUSG00000026360 | Rgs2 | regulator of G-protein signaling 2 [Source:MGI Symbol;Acc:MGI:1098271] | 41,3892329 | 0,73571715 | **1,66522503** | 5,35823822 | 26,7842851 | 2,2748E-07 | 2,75863E-06 | protein_coding |
| ENSMUSG00000019769 | Syne1 | synaptic nuclear envelope 1 [Source:MGI Symbol;Acc:MGI:1927152] | 22,04663667 | 0,73441909 | **1,66372742** | 4,46475325 | 17,3028908 | 3,18702E-05 | 0,00024538 | protein_coding |
| ENSMUSG00000061175 | Fnip2 | folliculin interacting protein 2 [Source:MGI Symbol;Acc:MGI:2683054] | 344,9928728 | 0,73193794 | **1,6608686** | 8,43061297 | 15,853515 | 6,84393E-05 | 0,000487009 | protein_coding |
| ENSMUSG00000024424 | Ttc39c | tetratricopeptide repeat domain 39C [Source:MGI Symbol;Acc:MGI:1919997] | 13,28318801 | 0,73039815 | **1,6590969** | 3,73900541 | 14,139463 | 0,000169745 | 0,001084297 | protein_coding |
| ENSMUSG00000017418 | Arl5b | ADP-ribosylation factor-like 5B [Source:MGI Symbol;Acc:MGI:1923119] | 10,67153255 | 0,72968113 | **1,65827253** | 3,43224658 | 11,9963168 | 0,000533058 | 0,00297144 | protein_coding |
| ENSMUSG00000063286 | Gm8995 | predicted gene 8995 [Source:MGI Symbol;Acc:MGI:3644223] | 18,23547714 | 0,72941576 | **1,65796754** | 4,19693255 | 13,2877542 | 0,000267145 | 0,001613593 | ocessed_pseudoge |
| ENSMUSG00000024924 | Vldlr | very low density lipoprotein receptor [Source:MGI Symbol;Acc:MGI:98935] | 5,38988481 | 0,728815 | **1,65727728** | 2,43202397 | 6,08910157 | 0,013601823 | 0,046468667 | protein_coding |
| ENSMUSG00000039616 | Mocos | molybdenum cofactor sulfurase [Source:MGI Symbol;Acc:MGI:1915841] | 42,38141579 | 0,72873724 | **1,65718796** | 5,40840863 | 14,673051 | 0,000127861 | 0,000846783 | protein_coding |
| ENSMUSG00000063382 | Bcl9l | B cell CLL/lymphoma 9-like [Source:MGI Symbol;Acc:MGI:1933114] | 10,10142854 | 0,7276179 | **1,65590269** | 3,32295564 | 10,8089212 | 0,001010121 | 0,005176401 | protein_coding |
| ENSMUSG00000032324 | Tspan3 | tetraspanin 3 [Source:MGI Symbol;Acc:MGI:1928098] | 165,6181291 | 0,72615175 | **1,65422073** | 7,37504246 | 29,8618827 | 4,63945E-08 | 6,56031E-07 | protein_coding |
| ENSMUSG00000091780 | Sco2 | SCO cytochrome oxidase deficient homolog 2 (yeast) [Source:MGI Symbol;Acc:MGI:3818630] | 8,998866216 | 0,72518073 | **1,65310771** | 3,16964584 | 10,4196161 | 0,001246838 | 0,006207073 | protein_coding |
| ENSMUSG00000063856 | Gpx1 | glutathione peroxidase 1 [Source:MGI Symbol;Acc:MGI:104887] | 106,1733031 | 0,72469299 | **1,65254893** | 6,7276249 | 31,2044071 | 2,32238E-08 | 3,46215E-07 | protein_coding |
| ENSMUSG00000011179 | Odc1 | ornithine decarboxylase, structural 1 [Source:MGI Symbol;Acc:MGI:97402] | 76,53687865 | 0,72450816 | **1,65233723** | 6,26266924 | 27,6290542 | 1,46955E-07 | 1,8516E-06 | protein_coding |
| ENSMUSG00000025950 | Idh1 | isocitrate dehydrogenase 1 (NADP+), soluble [Source:MGI Symbol;Acc:MGI:96413] | 134,1390321 | 0,72395131 | **1,65169958** | 7,06808614 | 30,5175316 | 3,30863E-08 | 4,84692E-07 | protein_coding |
| ENSMUSG00000039747 | Orai2 | ORAI calcium release-activated calcium modulator 2 [Source:MGI Symbol;Acc:MGI:2443195] | 38,55235888 | 0,72379825 | **1,65152436** | 5,27698576 | 25,1844639 | 5,21002E-07 | 5,93802E-06 | protein_coding |
| ENSMUSG00000057134 | Ado | 2-aminoethanethiol (cysteamine) dioxygenase [Source:MGI Symbol;Acc:MGI:2685083] | 38,22188065 | 0,72294474 | **1,65054759** | 5,25962268 | 25,1665686 | 5,25859E-07 | 5,98097E-06 | protein_coding |
| ENSMUSG00000020063 | Sirt1 | sirtuin 1 (silent mating type information regulation 2, homolog) 1 (S. cerevisiae) [Source:MGI Symbol;Acc:MGI:2135607] | 15,54160526 | 0,72210743 | **1,64958993** | 3,96090058 | 15,9237784 | 6,5945E-05 | 0,000471704 | protein_coding |
| ENSMUSG00000090124 | Ugt1a7c | UDP glucuronosyltransferase 1 family, polypeptide A7C [Source:MGI Symbol;Acc:MGI:3032636] | 163,8205719 | 0,72167402 | **1,64909444** | 7,35789879 | 21,3412551 | 3,8437E-06 | 3,68506E-05 | protein_coding |
| ENSMUSG00000026923 | Notch1 | notch 1 [Source:MGI Symbol;Acc:MGI:97363] | 124,4822392 | 0,72149937 | **1,64889481** | 6,96013833 | 29,3663475 | 5,9909E-08 | 8,28165E-07 | protein_coding |
| ENSMUSG00000036026 | Tmem63b | transmembrane protein 63b [Source:MGI Symbol;Acc:MGI:2387609] | 31,16320437 | 0,72110308 | **1,64844194** | 4,9652548 | 22,1925662 | 2,46627E-06 | 2,46882E-05 | protein_coding |
| ENSMUSG00000062190 | Lancl2 | LanC (bacterial lantibiotic synthetase component C)-like 2 [Source:MGI Symbol;Acc:MGI:1919085] | 15,88625562 | 0,72045775 | **1,64770474** | 3,99626254 | 12,4868922 | 0,000409817 | 0,002357416 | protein_coding |
| ENSMUSG00000045165 | AI467606 | expressed sequence AI467606 [Source:MGI Symbol;Acc:MGI:2141979] | 16,96190008 | 0,72043145 | **1,64767471** | 4,07943581 | 18,7141559 | 1,51851E-05 | 0,000127164 | protein_coding |
| ENSMUSG00000004565 | Pnpla6 | patatin-like phospholipase domain containing 6 [Source:MGI Symbol;Acc:MGI:1354723] | 26,43162014 | 0,71983793 | **1,646997** | 4,72330854 | 22,8872522 | 1,71787E-06 | 1,77557E-05 | protein_coding |
| ENSMUSG00000038508 | Gdf15 | growth differentiation factor 15 [Source:MGI Symbol;Acc:MGI:1346047] | 43,99223827 | 0,71975276 | **1,64689977** | 5,46079416 | 12,099394 | 0,000504382 | 0,002830259 | protein_coding |
| ENSMUSG00000073557 | Ppp1r12b | protein phosphatase 1, regulatory (inhibitor) subunit 12B [Source:MGI Symbol;Acc:MGI:1916417] | 12,875524 | 0,71919917 | **1,64626795** | 3,68079699 | 13,1172191 | 0,000292594 | 0,001749989 | protein_coding |
| ENSMUSG00000022010 | Tsc22d1 | TSC22 domain family, member 1 [Source:MGI Symbol;Acc:MGI:109127] | 22,41555748 | 0,71905402 | **1,64610233** | 4,49101005 | 17,7026973 | 2,5826E-05 | 0,000203844 | protein_coding |
| ENSMUSG00000034265 | Zdhhc14 | zinc finger, DHHC domain containing 14 [Source:MGI Symbol;Acc:MGI:2653229] | 29,29443594 | 0,71843019 | **1,6453907** | 4,87300732 | 18,8020152 | 1,45014E-05 | 0,000121809 | protein_coding |
| ENSMUSG00000029007 | Agtrap | angiotensin II, type I receptor-associated protein [Source:MGI Symbol;Acc:MGI:1339977] | 48,37470753 | 0,71817367 | **1,64509817** | 5,60031755 | 28,2574156 | 1,06207E-07 | 1,38751E-06 | protein_coding |
| ENSMUSG00000016552 | Foxred2 | FAD-dependent oxidoreductase domain containing 2 [Source:MGI Symbol;Acc:MGI:106315] | 6,556664594 | 0,71772157 | **1,64458271** | 2,71423648 | 6,72530477 | 0,009505449 | 0,034870239 | protein_coding |
| ENSMUSG00000058163 | Gm5431 | predicted gene 5431 [Source:MGI Symbol;Acc:MGI:3645205] | 23,86128175 | 0,71735991 | **1,64417049** | 4,5864868 | 16,2909529 | 5,43227E-05 | 0,000397366 | protein_coding |
| ENSMUSG00000026836 | Acvr1 | activin A receptor, type 1 [Source:MGI Symbol;Acc:MGI:87911] | 14,88239589 | 0,71695918 | **1,64371387** | 3,9104028 | 10,8063605 | 0,00101152 | 0,00518115 | protein_coding |
| ENSMUSG00000033960 | 9430020K01Rik | RIKEN cDNA 9430020K01 gene [Source:MGI Symbol;Acc:MGI:2685174] | 7,580427549 | 0,71671801 | **1,64343912** | 2,94038192 | 7,33259834 | 0,006771509 | 0,026178246 | protein_coding |
| ENSMUSG00000060639 | Hist1h4i | histone cluster 1, H4i [Source:MGI Symbol;Acc:MGI:2448432] | 5,172953065 | 0,71486678 | **1,64133165** | 2,37199967 | 5,97605547 | 0,0145014 | 0,04894835 | protein_coding |
| ENSMUSG00000042759 | Apobr | apolipoprotein B receptor [Source:MGI Symbol;Acc:MGI:2176230] | 78,53137039 | 0,71482937 | **1,64128909** | 6,29927815 | 29,2803307 | 6,26282E-08 | 8,6012E-07 | protein_coding |
| ENSMUSG00000028184 | Lphn2 | latrophilin 2 [Source:MGI Symbol;Acc:MGI:2139714] | 114,9478428 | 0,71451576 | **1,64093235** | 6,84921839 | 26,0890991 | 3,26019E-07 | 3,86405E-06 | protein_coding |
| ENSMUSG00000057531 | Dtnbp1 | dystrobrevin binding protein 1 [Source:MGI Symbol;Acc:MGI:2137586] | 62,66278303 | 0,71387646 | **1,64020536** | 5,97003017 | 21,4701419 | 3,59381E-06 | 3,47887E-05 | protein_coding |
| ENSMUSG00000024527 | Afg3l2 | AFG3(ATPase family gene 3)-like 2 (yeast) [Source:MGI Symbol;Acc:MGI:1916847] | 71,80784503 | 0,71316083 | **1,63939196** | 6,1679907 | 23,9700813 | 9,78444E-07 | 1,07073E-05 | protein_coding |
| ENSMUSG00000002871 | Tpra1 | transmembrane protein, adipocyte asscociated 1 [Source:MGI Symbol;Acc:MGI:1345190] | 46,90159899 | 0,7112187 | **1,63718653** | 5,55357514 | 18,9126798 | 1,3684E-05 | 0,000115562 | protein_coding |
| ENSMUSG00000021306 | Gpr137b | G protein-coupled receptor 137B [Source:MGI Symbol;Acc:MGI:1891463] | 272,258747 | 0,710613 | **1,63649932** | 8,08896726 | 14,4533329 | 0,000143675 | 0,000939061 | protein_coding |
| ENSMUSG00000024197 | Plin3 | perilipin 3 [Source:MGI Symbol;Acc:MGI:1914155] | 92,96652908 | 0,70958059 | **1,63532864** | 6,53938019 | 23,3230131 | 1,36952E-06 | 1,44265E-05 | protein_coding |
| ENSMUSG00000079334 | Nat6 | N-acetyltransferase 6 [Source:MGI Symbol;Acc:MGI:1888902] | 25,64241831 | 0,70954742 | **1,63529104** | 4,67845578 | 19,2070579 | 1,17279E-05 | 0,00010012 | protein_coding |
| ENSMUSG00000023074 | Mospd1 | motile sperm domain containing 1 [Source:MGI Symbol;Acc:MGI:1917630] | 8,547110848 | 0,70894755 | **1,63461123** | 3,1073367 | 7,17425127 | 0,007395731 | 0,028175067 | protein_coding |
| ENSMUSG00000020190 | Mknk2 | MAP kinase-interacting serine/threonine kinase 2 [Source:MGI Symbol;Acc:MGI:894279] | 99,4402503 | 0,70816966 | **1,6337301** | 6,63352784 | 28,8348744 | 7,8819E-08 | 1,0581E-06 | protein_coding |
| ENSMUSG00000028920 | Fbxo42 | F-box protein 42 [Source:MGI Symbol;Acc:MGI:1924992] | 44,74151933 | 0,70735554 | **1,63280844** | 5,48919469 | 24,2588764 | 8,42181E-07 | 9,3465E-06 | protein_coding |
| ENSMUSG00000019810 | Fuca2 | fucosidase, alpha-L- 2, plasma [Source:MGI Symbol;Acc:MGI:1914098] | 85,93204534 | 0,70730605 | **1,63275243** | 6,4284238 | 21,9212419 | 2,84072E-06 | 2,80171E-05 | protein_coding |
| ENSMUSG00000086513 | 9130208D14Rik | RIKEN cDNA 9130208D14 gene [Source:MGI Symbol;Acc:MGI:1924950] | 20,44353634 | 0,70521969 | **1,63039292** | 4,36429992 | 17,9351704 | 2,28558E-05 | 0,000183431 | pseudogene |
| ENSMUSG00000021895 | Arhgef3 | Rho guanine nucleotide exchange factor (GEF) 3 [Source:MGI Symbol;Acc:MGI:1918954] | 22,50708878 | 0,70327846 | **1,62820061** | 4,50099363 | 19,6269577 | 9,41317E-06 | 8,22118E-05 | protein_coding |
| ENSMUSG00000000530 | Acvrl1 | activin A receptor, type II-like 1 [Source:MGI Symbol;Acc:MGI:1338946] | 55,17982166 | 0,70160469 | **1,62631271** | 5,79342255 | 19,8751322 | 8,26687E-06 | 7,32485E-05 | protein_coding |
| ENSMUSG00000005054 | Cstb | cystatin B [Source:MGI Symbol;Acc:MGI:109514] | 349,0963837 | 0,70126836 | **1,62593362** | 8,44788346 | 17,1154495 | 3,5175E-05 | 0,000267082 | protein_coding |
| ENSMUSG00000039191 | Rbpj | recombination signal binding protein for immunoglobulin kappa J region [Source:MGI Symbol;Acc:MGI:96522] | 440,666426 | 0,70041176 | **1,62496851** | 8,78420062 | 19,2947447 | 1,12014E-05 | 9,59236E-05 | protein_coding |
| ENSMUSG00000073889 | Il11ra1 | interleukin 11 receptor, alpha chain 1 [Source:MGI Symbol;Acc:MGI:107426] | 41,91809611 | 0,69874658 | **1,62309403** | 5,38755524 | 19,8760156 | 8,26305E-06 | 7,32485E-05 | protein_coding |
| ENSMUSG00000097829 | | myeloid-associated differentiation marker [Source:RefSeq peptide;Acc:NP_001087235] | 12,81376554 | 0,69810973 | **1,62237771** | 3,69052344 | 10,3176733 | 0,001317623 | 0,00650347 | protein_coding |
| ENSMUSG00000026198 | Abcb6 | ATP-binding cassette, sub-family B (MDR/TAP), member 6 [Source:MGI Symbol;Acc:MGI:1921354] | 17,03620714 | 0,69761351 | **1,62181978** | 4,09678246 | 12,1889971 | 0,000480722 | 0,002712735 | protein_coding |
| ENSMUSG00000040596 | Pogk | pogo transposable element with KRAB domain [Source:MGI Symbol;Acc:MGI:1918842] | 52,87329777 | 0,69745661 | **1,6216434** | 5,72992998 | 17,1849747 | 3,39108E-05 | 0,000258914 | protein_coding |

| | | | | | | | | | | |
|---|---|---|---|---|---|---|---|---|---|---|
| ENSMUSG00000038642 | Ctss | cathepsin S [Source:MGI Symbol;Acc:MGI:107341] | 1132,532188 | 0,69670981 | **1,62080419** | 10,1453548 | 11,2589192 | 0,000792413 | 0,00420301 | protein_coding |
| ENSMUSG00000059316 | Slc27a4 | solute carrier family 27 (fatty acid transporter), member 4 [Source:MGI Symbol;Acc:MGI:1347347] | 24,09778606 | 0,69659978 | **1,62068059** | 4,59597427 | 20,1772526 | 7,05874E-06 | 6,35171E-05 | protein_coding |
| ENSMUSG00000041075 | Fzd7 | frizzled homolog 7 (Drosophila) [Source:MGI Symbol;Acc:MGI:108570] | 9,242024007 | 0,69508179 | **1,61897621** | 3,21659358 | 9,5036253 | 0,002050663 | 0,009575282 | protein_coding |
| ENSMUSG00000040151 | Hs2st1 | heparan sulfate 2-O-sulfotransferase 1 [Source:MGI Symbol;Acc:MGI:1346049] | 38,24999637 | 0,69311684 | **1,61677267** | 5,26454483 | 23,5468323 | 1,2191E-06 | 1,29413E-05 | protein_coding |
| ENSMUSG00000028645 | Slc2a1 | solute carrier family 2 (facilitated glucose transporter), member 1 [Source:MGI Symbol;Acc:MGI:95755] | 101,2674511 | 0,69253221 | **1,61611763** | 6,66407519 | 20,9376054 | 4,74488E-06 | 4,46718E-05 | protein_coding |
| ENSMUSG00000079442 | St6galnac4 | ST6 (alpha-N-acetyl-neuraminyl-2,3-beta-galactosyl-1,3)-N-acetylgalactosaminide alpha-2,6-sialyltransferase 4 [Source:I | 43,65173928 | 0,69244781 | **1,61602309** | 5,44987479 | 22,5434528 | 2,05444E-06 | 2,08728E-05 | protein_coding |
| ENSMUSG00000048279 | Sacs | sacsin [Source:MGI Symbol;Acc:MGI:1354724] | 19,37130482 | 0,69200972 | **1,61553244** | 4,28070518 | 15,896546 | 6,69007E-05 | 0,000477608 | protein_coding |
| ENSMUSG00000027883 | Gpsm2 | G-protein signalling modulator 2 (AGS3-like, C. elegans) [Source:MGI Symbol;Acc:MGI:1923373] | 10,07994085 | 0,69183101 | **1,61533233** | 3,34396956 | 9,29467111 | 0,002298214 | 0,010542997 | protein_coding |
| ENSMUSG00000056952 | Tatdn2 | TatD DNase domain containing 2 [Source:MGI Symbol;Acc:MGI:3576210] | 76,77855273 | 0,69159447 | **1,61506751** | 6,26268186 | 24,7341591 | 6,58074E-07 | 7,37782E-06 | protein_coding |
| ENSMUSG00000025810 | Nrp1 | neuropilin 1 [Source:MGI Symbol;Acc:MGI:106206] | 129,161475 | 0,69123785 | **1,61466833** | 7,01324083 | 21,4516654 | 3,62861E-06 | 3,50638E-05 | protein_coding |
| ENSMUSG00000024187 | Itfg3 | integrin alpha FG-GAP repeat containing 3 [Source:MGI Symbol;Acc:MGI:2146854] | 119,9148261 | 0,6897996 | **1,61305944** | 6,90726782 | 22,4961962 | 2,1056E-06 | 2,13022E-05 | protein_coding |
| ENSMUSG00000028247 | Coq3 | coenzyme Q3 homolog, methyltransferase (yeast) [Source:MGI Symbol;Acc:MGI:101813] | 11,41738045 | 0,68949762 | **1,61272183** | 3,52728517 | 8,10160848 | 0,004422599 | 0,018250431 | protein_coding |
| ENSMUSG00000026437 | Cdk18 | cyclin-dependent kinase 18 [Source:MGI Symbol;Acc:MGI:97518] | 240,5478614 | 0,68917581 | **1,61236214** | 7,91028722 | 17,6013608 | 2,72393E-05 | 0,000213761 | protein_coding |
| ENSMUSG00000046985 | Tapt1 | transmembrane anterior posterior transformation 1 [Source:MGI Symbol;Acc:MGI:2683537] | 26,74235965 | 0,68892016 | **1,61207644** | 4,75095125 | 17,3198913 | 3,15863E-05 | 0,000243707 | protein_coding |
| ENSMUSG00000016520 | Lnx2 | ligand of numb-protein X 2 [Source:MGI Symbol;Acc:MGI:2155959] | 9,00617488 | 0,68837289 | **1,61146504** | 3,18170235 | 9,26584186 | 0,002334671 | 0,010692386 | protein_coding |
| ENSMUSG00000028692 | Akr1a1 | aldo-keto reductase family 1, member A1 (aldehyde reductase) [Source:MGI Symbol;Acc:MGI:1929955] | 1251,708745 | 0,68604836 | **1,60887067** | 10,2897233 | 10,7397477 | 0,001048586 | 0,005348569 | protein_coding |
| ENSMUSG00000048216 | Gpr85 | G protein-coupled receptor 85 [Source:MGI Symbol;Acc:MGI:1927851] | 21,41443843 | 0,68434925 | **1,60697697** | 4,43356514 | 15,6224955 | 7,73291E-05 | 0,000544975 | protein_coding |
| ENSMUSG00000021281 | Tnfaip2 | tumor necrosis factor, alpha-induced protein 2 [Source:MGI Symbol;Acc:MGI:104960] | 374,6732587 | 0,68376371 | **1,60632488** | 8,54993842 | 18,3528482 | 1,83544E-05 | 0,000150493 | protein_coding |
| ENSMUSG00000022799 | Arhgap31 | Rho GTPase activating protein 31 [Source:MGI Symbol;Acc:MGI:1333857] | 101,0743762 | 0,68330025 | **1,60580894** | 6,66158101 | 27,2114108 | 1,82379E-07 | 2,25399E-06 | protein_coding |
| ENSMUSG00000037890 | Wdr19 | WD repeat domain 19 [Source:MGI Symbol;Acc:MGI:2443231] | 7,58223987 | 0,68303827 | **1,60551737** | 2,91670118 | 7,91783089 | 0,004894993 | 0,019837671 | protein_coding |
| ENSMUSG00000041654 | Slc39a11 | solute carrier family 39 (metal ion transporter), member 11 [Source:MGI Symbol;Acc:MGI:1917056] | 38,50534097 | 0,68157757 | **1,60389263** | 5,27160448 | 16,0294266 | 6,23656E-05 | 0,000450204 | protein_coding |
| ENSMUSG00000042694 | Obfc1 | oligonucleotide/oligosaccharide-binding fold containing 1 [Source:MGI Symbol;Acc:MGI:1915581] | 6,230588856 | 0,6813784 | **1,60367123** | 2,65081928 | 6,33456049 | 0,011840745 | 0,041790643 | protein_coding |
| ENSMUSG00000022353 | Mtss1 | metastasis suppressor 1 [Source:MGI Symbol;Acc:MGI:2384818] | 130,8836353 | 0,68099531 | **1,60324544** | 7,03184418 | 22,7141965 | 1,87974E-06 | 1,93016E-05 | protein_coding |
| ENSMUSG00000021224 | Numb | numb gene homolog (Drosophila) [Source:MGI Symbol;Acc:MGI:107423] | 42,83322667 | 0,68072977 | **1,60295038** | 5,4226225 | 21,0933084 | 4,37453E-06 | 4,14751E-05 | protein_coding |
| ENSMUSG00000068220 | Lgals1 | lectin, galactose binding, soluble 1 [Source:MGI Symbol;Acc:MGI:96777] | 695,7002364 | 0,68056532 | **1,60276767** | 9,44258331 | 12,5782789 | 0,000390256 | 0,002262661 | protein_coding |
| ENSMUSG00000039680 | Mrps6 | mitochondrial ribosomal protein S6 [Source:MGI Symbol;Acc:MGI:2153111] | 14,93529109 | 0,68018909 | **1,60234976** | 3,89667918 | 14,3089812 | 0,000155123 | 0,001004915 | protein_coding |
| ENSMUSG00000009772 | Nuak2 | NUAK family, SNF1-like kinase, 2 [Source:MGI Symbol;Acc:MGI:1921387] | 22,66129408 | 0,6801747 | **1,60233377** | 4,50106131 | 16,3924943 | 5,14887E-05 | 0,000378072 | protein_coding |
| ENSMUSG00000036986 | Pml | promyelocytic leukemia [Source:MGI Symbol;Acc:MGI:104662] | 53,92029611 | 0,68017004 | **1,6023286** | 5,75648344 | 22,6522404 | 1,94134E-06 | 1,98414E-05 | protein_coding |
| ENSMUSG00000040552 | C3ar1 | complement component 3a receptor 1 [Source:MGI Symbol;Acc:MGI:1097680] | 1292,854051 | 0,6798263 | **1,60194687** | 10,3364736 | 10,4472751 | 0,001228305 | 0,006128696 | protein_coding |
| ENSMUSG00000057789 | Bak1 | BCL2-antagonist/killer 1 [Source:MGI Symbol;Acc:MGI:1097161] | 66,86877442 | 0,67766346 | **1,59954709** | 6,06598968 | 23,769589 | 1,08585E-06 | 1,16848E-05 | protein_coding |
| ENSMUSG00000026384 | Ptpn4 | protein tyrosine phosphatase, non-receptor type 4 [Source:MGI Symbol;Acc:MGI:1099792] | 5,925622387 | 0,67555327 | **1,59720918** | 2,56271089 | 6,13852496 | 0,01322676 | 0,045470092 | protein_coding |
| ENSMUSG00000050022 | Amz1 | archaelysin family metallopeptidase 1 [Source:MGI Symbol;Acc:MGI:2442258] | 17,59108555 | 0,67377131 | **1,59523759** | 4,13757552 | 10,5730873 | 0,001147459 | 0,005769856 | protein_coding |
| ENSMUSG00000029267 | Mtf2 | metal response element binding transcription factor 2 [Source:MGI Symbol;Acc:MGI:105050] | 31,44413661 | 0,67197835 | **1,59325628** | 4,98163964 | 17,8292985 | 2,41634E-05 | 0,000192259 | protein_coding |
| ENSMUSG00000043154 | Ppp2r3a | protein phosphatase 2, regulatory subunit B'', alpha [Source:MGI Symbol;Acc:MGI:2442104] | 7,592552339 | 0,67130933 | **1,59251761** | 2,91937851 | 6,47347395 | 0,010949629 | 0,039110395 | protein_coding |
| ENSMUSG00000017466 | Timp2 | tissue inhibitor of metalloproteinase 2 [Source:MGI Symbol;Acc:MGI:98753] | 252,8449013 | 0,67061999 | **1,59175687** | 7,98185222 | 15,7286218 | 7,31095E-05 | 0,000518898 | protein_coding |
| ENSMUSG00000025154 | Arhgap19 | Rho GTPase activating protein 19 [Source:MGI Symbol;Acc:MGI:1918335] | 74,05523056 | 0,66970723 | **1,59075012** | 6,21167315 | 18,9769229 | 1,32309E-05 | 0,000111994 | protein_coding |
| ENSMUSG00000040746 | Rnf167 | ring finger protein 167 [Source:MGI Symbol;Acc:MGI:1917760] | 14,03956092 | 0,66856132 | **1,58948711** | 3,79385421 | 13,0554898 | 0,000302397 | 0,001798827 | protein_coding |
| ENSMUSG00000018378 | Cuedc1 | CUE domain containing 1 [Source:MGI Symbol;Acc:MGI:2144281] | 8,327558281 | 0,66795189 | **1,58881581** | 3,05106146 | 7,69394961 | 0,005540625 | 0,022120219 | protein_coding |
| ENSMUSG00000020189 | Osbpl8 | oxysterol binding protein-like 8 [Source:MGI Symbol;Acc:MGI:2443807] | 653,6781775 | 0,66707159 | **1,58784666** | 9,35255959 | 11,861395 | 0,000573096 | 0,003160947 | protein_coding |
| ENSMUSG00000042029 | Ncapg2 | non-SMC condensin II complex, subunit G2 [Source:MGI Symbol;Acc:MGI:1923294] | 21,10804804 | 0,66678221 | **1,58752819** | 4,4035414 | 15,2292529 | 9,5217E-05 | 0,00065262 | protein_coding |
| ENSMUSG00000032265 | Fam46a | family with sequence similarity 46, member A [Source:MGI Symbol;Acc:MGI:2670964] | 35,95286452 | 0,66519648 | **1,58578423** | 5,17238824 | 22,1918021 | 2,46725E-06 | 2,46882E-05 | protein_coding |
| ENSMUSG00000029657 | Hsph1 | heat shock 105kDa/110kDa protein 1 [Source:MGI Symbol;Acc:MGI:105053] | 45,06669262 | 0,66437934 | **1,5848863** | 5,50415965 | 19,4750349 | 1,01923E-05 | 8,81755E-05 | protein_coding |
| ENSMUSG00000021175 | Cdca7l | cell division cycle associated 7 like [Source:MGI Symbol;Acc:MGI:2384982] | 14,44557366 | 0,66358305 | **1,58401176** | 3,86111369 | 9,18949584 | 0,002434079 | 0,011085183 | protein_coding |
| ENSMUSG00000020629 | Adi1 | acireductone dioxygenase 1 [Source:MGI Symbol;Acc:MGI:2144929] | 27,849783 | 0,66321397 | **1,58360658** | 4,79875495 | 18,2501209 | 1,93714E-05 | 0,000157771 | protein_coding |
| ENSMUSG00000079834 | Tmlhe | trimethyllysine hydroxylase, epsilon [Source:MGI Symbol;Acc:MGI:2180203] | 17,16664793 | 0,66160487 | **1,5818413** | 4,11120146 | 13,5384193 | 0,000233729 | 0,001435429 | protein_coding |
| ENSMUSG00000034908 | Sidt2 | SID1 transmembrane family, member 2 [Source:MGI Symbol;Acc:MGI:2446134] | 54,11264207 | 0,66031436 | **1,58042696** | 5,75957155 | 23,8584342 | 1,03687E-06 | 1,1257E-05 | protein_coding |
| ENSMUSG00000008435 | Rdh13 | retinol dehydrogenase 13 (all-trans and 9-cis) [Source:MGI Symbol;Acc:MGI:1918732] | 23,19566934 | 0,6591663 | **1,5791698** | 4,53959762 | 15,9202429 | 6,60683E-05 | 0,000471972 | protein_coding |
| ENSMUSG00000025572 | Tmc6 | transmembrane channel-like gene family 6 [Source:MGI Symbol;Acc:MGI:1098686] | 17,15854727 | 0,65912993 | **1,57912998** | 4,09466737 | 15,0621248 | 0,00010403 | 0,000706413 | protein_coding |
| ENSMUSG00000035232 | Pdk3 | pyruvate dehydrogenase kinase, isoenzyme 3 [Source:MGI Symbol;Acc:MGI:2384308] | 9,239191376 | 0,65908671 | **1,57908268** | 3,22909212 | 8,09155581 | 0,004447197 | 0,018327589 | protein_coding |
| ENSMUSG00000096370 | | RNA-binding protein 4 [Source:UniProtKB/Swiss-Prot;Acc:Q8C7Q4] | 10,22480881 | 0,65705464 | **1,57686006** | 3,35700771 | 8,58277797 | 0,003393572 | 0,014667656 | protein_coding |
| ENSMUSG00000078780 | Gm5150 | predicted gene 5150 [Source:MGI Symbol;Acc:MGI:3779469] | 26,26410398 | 0,65618593 | **1,57591085** | 4,74347299 | 18,6395878 | 1,57907E-05 | 0,000132135 | protein_coding |
| ENSMUSG00000031788 | Kifc3 | kinesin family member C3 [Source:MGI Symbol;Acc:MGI:109202] | 13,72667195 | 0,65505696 | **1,57467813** | 3,76302298 | 11,9680587 | 0,000541203 | 0,00300313 | protein_coding |
| ENSMUSG00000037885 | Stk35 | serine/threonine kinase 35 [Source:MGI Symbol;Acc:MGI:1914583] | 31,82515792 | 0,65492344 | **1,5745324** | 4,99651522 | 19,8550475 | 8,35419E-06 | 7,39625E-05 | protein_coding |
| ENSMUSG00000020888 | Dvl2 | dishevelled 2, dsh homolog (Drosophila) [Source:MGI Symbol;Acc:MGI:106613] | 15,98319633 | 0,65373022 | **1,57323067** | 3,99350296 | 13,8637014 | 0,000196558 | 0,00123405 | protein_coding |
| ENSMUSG00000039361 | Picalm | phosphatidylinositol binding clathrin assembly protein [Source:MGI Symbol;Acc:MGI:2385902] | 399,881803 | 0,65325461 | **1,57271212** | 8,64419203 | 17,1700955 | 3,41774E-05 | 0,000260588 | protein_coding |
| ENSMUSG00000020472 | Zkscan17 | zinc finger with KRAB and SCAN domains 17 [Source:MGI Symbol;Acc:MGI:2679270] | 11,30832776 | 0,6491412 | **1,56823439** | 3,50238936 | 9,60099674 | 0,001944718 | 0,009154505 | protein_coding |
| ENSMUSG00000021668 | Polk | polymerase (DNA directed), kappa [Source:MGI Symbol;Acc:MGI:1349767] | 22,35479494 | 0,64786395 | **1,56684661** | 4,48997004 | 10,6762782 | 0,001085182 | 0,005504567 | protein_coding |
| ENSMUSG00000030254 | Rad18 | RAD18 homolog (S. cerevisiae) [Source:MGI Symbol;Acc:MGI:1890476] | 7,760962166 | 0,64760181 | **1,56656194** | 2,97470465 | 6,73609042 | 0,009448146 | 0,034679209 | protein_coding |
| ENSMUSG00000034902 | Pip5k1c | phosphatidylinositol-4-phosphate 5-kinase, type 1 gamma [Source:MGI Symbol;Acc:MGI:1298224] | 170,0607242 | 0,64622364 | **1,56506616** | 7,40881121 | 20,9481157 | 4,71892E-06 | 4,44655E-05 | protein_coding |
| ENSMUSG00000039357 | Fut11 | fucosyltransferase 11 [Source:MGI Symbol;Acc:MGI:1920318] | 11,72217714 | 0,64566251 | **1,56445755** | 3,54448999 | 10,6131794 | 0,001122845 | 0,00566423 | protein_coding |
| ENSMUSG00000026074 | Map4k4 | mitogen-activated protein kinase kinase kinase kinase 4 [Source:MGI Symbol;Acc:MGI:1349394] | 77,78770886 | 0,64555559 | **1,5643416** | 6,28542762 | 21,7691384 | 3,07507E-06 | 3,01659E-05 | protein_coding |
| ENSMUSG00000078945 | Naip2 | NLR family, apoptosis inhibitory protein 2 [Source:MGI Symbol;Acc:MGI:1298226] | 85,35035488 | 0,64475303 | **1,56347162** | 6,41736203 | 22,0298155 | 2,68448E-06 | 2,66917E-05 | protein_coding |
| ENSMUSG00000037251 | 4930444A02Rik | RIKEN cDNA 4930444A02 gene [Source:MGI Symbol;Acc:MGI:1921903] | 12,5180582 | 0,64418943 | **1,56286096** | 3,66580057 | 11,1777568 | 0,000827838 | 0,004360238 | protein_coding |
| ENSMUSG00000030768 | Disp1 | dispatched homolog 1 (Drosophila) [Source:MGI Symbol;Acc:MGI:1916147] | 12,96873894 | 0,64405787 | **1,56271844** | 3,69805977 | 10,0319271 | 0,0015385 | 0,007476116 | protein_coding |
| ENSMUSG00000031904 | Slc7a6 | solute carrier family 7 (cationic amino acid transporter, y+ system), member 6 [Source:MGI Symbol;Acc:MGI:2142598] | 20,32038678 | 0,64238308 | **1,56090538** | 4,36107951 | 12,4601641 | 0,000415723 | 0,002381415 | protein_coding |
| ENSMUSG00000069792 | Wfdc17 | WAP four-disulfide core domain 17 [Source:MGI Symbol;Acc:MGI:3649773] | 90,97424035 | 0,64201276 | **1,56050477** | 6,5150476 | 22,7849386 | 1,8118E-06 | 1,86913E-05 | protein_coding |
| ENSMUSG00000032252 | Glce | glucuronyl C5-epimerase [Source:MGI Symbol;Acc:MGI:2136405] | 17,7832833 | 0,6415281 | **1,55998061** | 4,16449246 | 14,0562625 | 0,000177422 | 0,001127435 | protein_coding |
| ENSMUSG00000024381 | Bin1 | bridging integrator 1 [Source:MGI Symbol;Acc:MGI:108092] | 99,49879869 | 0,64082121 | **1,55921645** | 6,63802605 | 21,6773101 | 3,22584E-06 | 3,15043E-05 | protein_coding |
| ENSMUSG00000070639 | Lrrc8b | leucine rich repeat containing 8 family, member B [Source:MGI Symbol;Acc:MGI:2141353] | 7,029612651 | 0,6406182 | **1,55899706** | 2,81313205 | 6,50273943 | 0,010770841 | 0,038597272 | protein_coding |
| ENSMUSG00000046962 | Zfp295 | zinc finger protein 295 [Source:MGI Symbol;Acc:MGI:1927240] | 33,61629313 | 0,64035394 | **1,55871152** | 5,07530068 | 17,0223247 | 3,69429E-05 | 0,000279347 | protein_coding |
| ENSMUSG00000020536 | Llgl1 | lethal giant larvae homolog 1 (Drosophila) [Source:MGI Symbol;Acc:MGI:102682] | 33,5837778 | 0,63976043 | **1,55807041** | 5,0713684 | 18,503423 | 1,69599E-05 | 0,000140527 | protein_coding |

| | | | | | | | | | | |
|---|---|---|---|---|---|---|---|---|---|---|
| ENSMUSG00000022992 | Kansl2 | KAT8 regulatory NSL complex subunit 2 [Source:MGI Symbol;Acc:MGI:1916862] | 43,03109602 | 0,63918407 | **1,55744809** | 5,43299918 | 19,5679761 | 9,70829E-06 | 8,45206E-05 | protein_coding |
| ENSMUSG00000015711 | Prune | prune homolog (Drosophila) [Source:MGI Symbol;Acc:MGI:1925152] | 20,68427599 | 0,63886393 | **1,55710252** | 4,37297511 | 15,3202645 | 9,07379E-05 | 0,000626217 | protein_coding |
| ENSMUSG00000029456 | Acad10 | acyl-Coenzyme A dehydrogenase family, member 10 [Source:MGI Symbol;Acc:MGI:1919235] | 6,524612726 | 0,63843102 | **1,55663534** | 2,71900728 | 6,13503294 | 0,013252908 | 0,04554573 | protein_coding |
| ENSMUSG00000028617 | Lrrc42 | leucine rich repeat containing 42 [Source:MGI Symbol;Acc:MGI:1925059] | 16,47300525 | 0,63651463 | **1,55456898** | 4,06249034 | 12,1492947 | 0,000491064 | 0,002761169 | protein_coding |
| ENSMUSG00000032815 | Fanca | Fanconi anemia, complementation group A [Source:MGI Symbol;Acc:MGI:1341823] | 7,803605748 | 0,63576573 | **1,55376222** | 2,98057295 | 6,48879389 | 0,010855661 | 0,03883329 | protein_coding |
| ENSMUSG00000015568 | Lpl | lipoprotein lipase [Source:MGI Symbol;Acc:MGI:96820] | 2598,259155 | 0,6345278 | **1,55242956** | 11,3433551 | 6,06467798 | 0,01379119 | 0,047042472 | protein_coding |
| ENSMUSG00000020806 | Rhbdf2 | rhomboid 5 homolog 2 (Drosophila) [Source:MGI Symbol;Acc:MGI:2442473] | 71,46537771 | 0,63352856 | **1,55135468** | 6,16584731 | 18,6218753 | 1,59381E-05 | 0,000133165 | protein_coding |
| ENSMUSG00000033526 | Ppip5k1 | diphosphoinositol pentakisphosphate kinase 1 [Source:MGI Symbol;Acc:MGI:2443281] | 18,82467592 | 0,63350957 | **1,55133426** | 4,23327925 | 15,0487139 | 0,000104772 | 0,000710275 | protein_coding |
| ENSMUSG00000027695 | Pld1 | phospholipase D1 [Source:MGI Symbol;Acc:MGI:109585] | 17,79830734 | 0,63126021 | **1,5489174** | 4,1634014 | 14,6139601 | 0,000131934 | 0,000871126 | protein_coding |
| ENSMUSG00000032300 | 1700017B05Rik | RIKEN cDNA 1700017B05 gene [Source:MGI Symbol;Acc:MGI:1921461] | 86,65466002 | 0,62950242 | **1,54703133** | 6,43839312 | 17,3314654 | 3,13945E-05 | 0,000242568 | protein_coding |
| ENSMUSG00000006050 | Sra1 | steroid receptor RNA activator 1 [Source:MGI Symbol;Acc:MGI:1344414] | 34,64812398 | 0,62916104 | **1,54666531** | 5,11982981 | 19,5953766 | 9,57005E-06 | 8,33831E-05 | protein_coding |
| ENSMUSG00000041779 | Tram2 | translocating chain-associating membrane protein 2 [Source:MGI Symbol;Acc:MGI:1924817] | 9,941872394 | 0,62661271 | **1,54393575** | 3,32258143 | 8,34542744 | 0,003866592 | 0,016339325 | protein_coding |
| ENSMUSG00000055912 | Tmem150a | transmembrane protein 150A [Source:MGI Symbol;Acc:MGI:2385244] | 9,251613472 | 0,62566676 | **1,54292375** | 3,21153533 | 7,75679022 | 0,005351097 | 0,021457119 | protein_coding |
| ENSMUSG00000054499 | Dedd2 | death effector domain-containing DNA binding protein 2 [Source:MGI Symbol;Acc:MGI:1914629] | 8,125324409 | 0,62539311 | **1,5426311** | 3,02020297 | 6,59528818 | 0,0102249 | 0,037088472 | protein_coding |
| ENSMUSG00000021094 | Dhrs7 | dehydrogenase/reductase (SDR family) member 7 [Source:MGI Symbol;Acc:MGI:1913625] | 15,28077058 | 0,62537585 | **1,54261265** | 3,92790488 | 12,0615338 | 0,000514729 | 0,002876567 | protein_coding |
| ENSMUSG00000057137 | Tmem140 | transmembrane protein 140 [Source:MGI Symbol;Acc:MGI:1915737] | 39,18361627 | 0,6240833 | **1,5412312** | 5,29239953 | 16,9197891 | 3,8993E-05 | 0,000293636 | protein_coding |
| ENSMUSG00000028967 | Errfi1 | ERBB receptor feedback inhibitor 1 [Source:MGI Symbol;Acc:MGI:1921405] | 11,41856973 | 0,62128196 | **1,53824143** | 3,54086825 | 7,05804209 | 0,007891019 | 0,02982409 | protein_coding |
| ENSMUSG00000022263 | Trio | triple functional domain (PTPRF interacting) [Source:MGI Symbol;Acc:MGI:1927230] | 101,7295873 | 0,62002237 | **1,53689901** | 6,66752133 | 20,3724891 | 6,37396E-06 | 5,80204E-05 | protein_coding |
| ENSMUSG00000006301 | Tmbim1 | transmembrane BAX inhibitor motif containing 1 [Source:MGI Symbol;Acc:MGI:1916910] | 104,5865247 | 0,6183425 | **1,53511049** | 6,71078745 | 20,2749645 | 6,70725E-06 | 6,07523E-05 | protein_coding |
| ENSMUSG00000019990 | Pde7b | phosphodiesterase 7B [Source:MGI Symbol;Acc:MGI:1352752] | 15,33121452 | 0,61802863 | **1,53477656** | 3,94726562 | 8,14687914 | 0,004313533 | 0,017904341 | protein_coding |
| ENSMUSG00000048720 | Tbc1d12 | TBC1D12: TBC1 domain family, member 12 [Source:MGI Symbol;Acc:MGI:2384803] | 10,09027009 | 0,61748851 | **1,53420207** | 3,34443686 | 8,13314105 | 0,004346339 | 0,017999707 | protein_coding |
| ENSMUSG00000056919 | 4922501C03Rik | RIKEN cDNA 4922501C03 gene [Source:MGI Symbol;Acc:MGI:1925343] | 17,39946489 | 0,61744666 | **1,53415756** | 4,12606485 | 11,5400489 | 0,000681128 | 0,003682854 | protein_coding |
| ENSMUSG00000053581 | Zfand2a | zinc finger, AN1-type domain 2A [Source:MGI Symbol;Acc:MGI:2140729] | 78,32041923 | 0,61697337 | **1,53365435** | 6,29248161 | 17,8209507 | 2,42696E-05 | 0,000192945 | protein_coding |
| ENSMUSG00000031639 | Tlr3 | toll-like receptor 3 [Source:MGI Symbol;Acc:MGI:2156367] | 13,10335391 | 0,61518941 | **1,53175909** | 3,71966152 | 8,61508909 | 0,003333895 | 0,014460917 | protein_coding |
| ENSMUSG00000025417 | Pip4k2c | phosphatidylinositol-5-phosphate 4-kinase, type II, gamma [Source:MGI Symbol;Acc:MGI:2152214] | 61,76619108 | 0,61504848 | **1,53160946** | 5,95167263 | 16,5587541 | 4,71657E-05 | 0,000348728 | protein_coding |
| ENSMUSG00000049232 | Tigd2 | tigger transposable element derived 2 [Source:MGI Symbol;Acc:MGI:1915390] | 14,8681829 | 0,61501101 | **1,53156968** | 3,90046723 | 10,9896206 | 0,000916236 | 0,004757411 | protein_coding |
| ENSMUSG00000030172 | Erc1 | ELKS/RAB6-interacting/CAST family member 1 [Source:MGI Symbol;Acc:MGI:2151013] | 18,58500721 | 0,6142377 | **1,53074896** | 4,23062849 | 9,24994649 | 0,002355023 | 0,010776609 | protein_coding |
| ENSMUSG00000001227 | Sema6b | sema domain, transmembrane domain (TM), and cytoplasmic domain, (semaphorin) 6B [Source:MGI Symbol;Acc:MGI:1 | 10,21042952 | 0,61159048 | **1,52794274** | 3,34658519 | 8,00709636 | 0,004659439 | 0,019094576 | protein_coding |
| ENSMUSG00000002297 | Dbf4 | DBF4 homolog (S. cerevisiae) [Source:MGI Symbol;Acc:MGI:1351328] | 18,44153411 | 0,61157898 | **1,52793056** | 4,21337419 | 11,1839304 | 0,000825088 | 0,004348645 | protein_coding |
| ENSMUSG00000019960 | Dusp6 | dual specificity phosphatase 6 [Source:MGI Symbol;Acc:MGI:1914853] | 17,88391566 | 0,61128861 | **1,52762307** | 4,16974586 | 12,2733905 | 0,000459463 | 0,002607499 | protein_coding |
| ENSMUSG00000079043 | Fastkd5 | FAST kinase domains 5 [Source:MGI Symbol;Acc:MGI:2139469] | 9,240114993 | 0,61103883 | **1,52735861** | 3,2216281 | 7,36434695 | 0,006652976 | 0,025783508 | protein_coding |
| ENSMUSG00000009035 | Tmem184b | transmembrane protein 184b [Source:MGI Symbol;Acc:MGI:2445179] | 71,60501374 | 0,61072471 | **1,52702608** | 6,16286707 | 16,4135584 | 5,09197E-05 | 0,000374719 | protein_coding |
| ENSMUSG00000049323 | Smcr8 | Smith-Magenis syndrome chromosome region, candidate 8 homolog (human) [Source:MGI Symbol;Acc:MGI:2444720] | 159,3123565 | 0,61041225 | **1,5266954** | 7,31717328 | 21,153472 | 4,23933E-06 | 4,03827E-05 | protein_coding |
| ENSMUSG00000026979 | Psd4 | pleckstrin and Sec7 domain containing 4 [Source:MGI Symbol;Acc:MGI:2674093] | 131,1799197 | 0,61027519 | **1,52655036** | 7,0359462 | 20,1234121 | 7,26025E-06 | 6,50671E-05 | protein_coding |
| ENSMUSG00000049225 | Pdp1 | pyruvate dehyrogenase phosphatase catalytic subunit 1 [Source:MGI Symbol;Acc:MGI:2685870] | 19,92238069 | 0,61019582 | **1,52646639** | 4,32756809 | 11,919797 | 0,000555405 | 0,003069536 | protein_coding |
| ENSMUSG00000021510 | A530054K11Rik | RIKEN cDNA A530054K11 gene [Source:MGI Symbol;Acc:MGI:3036250] | 18,30009573 | 0,61015096 | **1,52641892** | 4,1950944 | 12,1497801 | 0,000490937 | 0,002761169 | protein_coding |
| ENSMUSG00000029924 | Slc37a3 | solute carrier family 37 (glycerol-3-phosphate transporter), member 3 [Source:MGI Symbol;Acc:MGI:1919394] | 13,31933741 | 0,60826806 | **1,52442805** | 3,74559047 | 8,58915984 | 0,0033817 | 0,01463361 | protein_coding |
| ENSMUSG00000041161 | Otud3 | OTU domain containing 3 [Source:MGI Symbol;Acc:MGI:1920412] | 9,795329751 | 0,60709397 | **1,52318795** | 3,28783848 | 8,24951861 | 0,004076281 | 0,01707438 | protein_coding |
| ENSMUSG00000028744 | Pqlc2 | PQ loop repeat containing 2 [Source:MGI Symbol;Acc:MGI:2384837] | 25,04871539 | 0,60593415 | **1,52196391** | 4,65005779 | 14,1407782 | 0,000169627 | 0,001084297 | protein_coding |
| ENSMUSG00000031609 | Sap30 | sin3 associated polypeptide [Source:MGI Symbol;Acc:MGI:1929129] | 20,07801882 | 0,60430687 | **1,52024819** | 4,34337948 | 13,8265054 | 0,000200488 | 0,00125585 | protein_coding |
| ENSMUSG00000023079 | Gtf2ird1 | general transcription factor II I repeat domain-containing 1 [Source:MGI Symbol;Acc:MGI:1861942] | 17,9982993 | 0,60403373 | **1,51996039** | 4,17613262 | 10,1243009 | 0,001463272 | 0,007145317 | protein_coding |
| ENSMUSG00000040820 | Hlcs | holocarboxylase synthetase (biotin- [propriony-Coenzyme A-carboxylase (ATP-hydrolysing)] ligase) [Source:MGI Symbol | 7,502566031 | 0,60391407 | **1,51983433** | 2,918225 | 6,25821011 | 0,012361904 | 0,043103852 | protein_coding |
| ENSMUSG00000029311 | Hsd17b11 | hydroxysteroid (17-beta) dehydrogenase 11 [Source:MGI Symbol;Acc:MGI:2149821] | 44,14444789 | 0,60303054 | **1,51890385** | 5,46575567 | 15,637113 | 7,67337E-05 | 0,000541821 | protein_coding |
| ENSMUSG00000026643 | Nmt2 | N-myristoyltransferase 2 [Source:MGI Symbol;Acc:MGI:1202298] | 37,00879364 | 0,60297978 | **1,5188504** | 5,21628762 | 17,8481892 | 2,39247E-05 | 0,000190894 | protein_coding |
| ENSMUSG00000037253 | Mex3c | mex3 homolog C (C. elegans) [Source:MGI Symbol;Acc:MGI:2652843] | 44,29374017 | 0,60279009 | **1,51865072** | 5,47509314 | 19,1748808 | 1,19273E-05 | 0,000101585 | protein_coding |
| ENSMUSG00000027274 | Mkks | McKusick-Kaufman syndrome [Source:MGI Symbol;Acc:MGI:1891836] | 19,99512047 | 0,60201059 | **1,51783039** | 4,33061343 | 13,4100395 | 0,000250281 | 0,001528538 | protein_coding |
| ENSMUSG00000011877 | Git1 | G protein-coupled receptor kinase-interactor 1 [Source:MGI Symbol;Acc:MGI:1927140] | 73,57162462 | 0,60159195 | **1,51739002** | 6,2000661 | 18,5846383 | 1,62525E-05 | 0,00013538 | protein_coding |
| ENSMUSG00000036698 | Eif2c2 | eukaryotic translation initiation factor 2C, 2 [Source:MGI Symbol;Acc:MGI:2446632] | 143,8960018 | 0,60146867 | **1,51726036** | 7,17006481 | 18,8217084 | 1,43524E-05 | 0,000121021 | protein_coding |
| ENSMUSG00000009569 | Mkl2 | MKL/myocardin-like 2 [Source:MGI Symbol;Acc:MGI:3050795] | 62,03432393 | 0,60059005 | **1,51633661** | 5,95650028 | 17,7820434 | 2,4771E-05 | 0,000196363 | protein_coding |
| ENSMUSG00000028673 | Fuca1 | fucosidase, alpha-L- 1, tissue [Source:MGI Symbol;Acc:MGI:95593] | 96,91827669 | 0,60003939 | **1,51575795** | 6,59845805 | 18,5976188 | 1,61422E-05 | 0,000134563 | protein_coding |
| ENSMUSG00000021676 | Iqgap2 | IQ motif containing GTPase activating protein 2 [Source:MGI Symbol;Acc:MGI:2449975] | 58,8330914 | 0,5969939 | **1,51256161** | 5,88153253 | 15,6426082 | 7,65111E-05 | 0,000540596 | protein_coding |
| ENSMUSG00000047881 | Rell1 | RELT-like 1 [Source:MGI Symbol;Acc:MGI:2140767] | 15,97479153 | 0,59696827 | **1,51253473** | 4,00153012 | 12,3600857 | 0,000438611 | 0,002498193 | protein_coding |
| ENSMUSG00000026159 | Agfg1 | ArfGAP with FG repeats 1 [Source:MGI Symbol;Acc:MGI:1333754] | 83,24663218 | 0,59620238 | **1,51173197** | 6,38528577 | 20,4387312 | 6,1571E-06 | 5,63265E-05 | protein_coding |
| ENSMUSG00000025355 | Mmp19 | matrix metallopeptidase 19 [Source:MGI Symbol;Acc:MGI:1927899] | 45,78372508 | 0,59561747 | **1,51111921** | 5,52241701 | 16,8963327 | 3,94778E-05 | 0,00029688 | protein_coding |
| ENSMUSG00000027630 | Tbl1xr1 | transducin (beta)-like 1X-linked receptor 1 [Source:MGI Symbol;Acc:MGI:2441730] | 66,3772281 | 0,59525906 | **1,51074384** | 6,05020216 | 21,0930423 | 4,37514E-06 | 4,14751E-05 | protein_coding |
| ENSMUSG00000073016 | Uprt | uracil phosphoribosyltransferase (FUR1) homolog (S. cerevisiae) [Source:MGI Symbol;Acc:MGI:2685620] | 11,08484097 | 0,59505539 | **1,51053058** | 3,47164534 | 7,63076073 | 0,00573811 | 0,022776232 | protein_coding |
| ENSMUSG00000030323 | Ift122 | intraflagellar transport 122 [Source:MGI Symbol;Acc:MGI:1932386] | 8,278502254 | 0,59474341 | **1,51020396** | 3,05473338 | 6,56840876 | 0,010380472 | 0,037516528 | protein_coding |
| ENSMUSG00000056267 | Cep70 | centrosomal protein 70 [Source:MGI Symbol;Acc:MGI:1915371] | 7,886590631 | 0,59397312 | **1,50939784** | 2,9762181 | 5,99501022 | 0,014346398 | 0,048529518 | protein_coding |
| ENSMUSG00000052102 | Gnpda1 | glucosamine-6-phosphate deaminase 1 [Source:MGI Symbol;Acc:MGI:1347054] | 124,029163 | 0,59312918 | **1,50851514** | 6,95533169 | 18,4667934 | 1,72891E-05 | 0,000143038 | protein_coding |
| ENSMUSG00000011752 | Pgam1 | phosphoglycerate mutase 1 [Source:MGI Symbol;Acc:MGI:97552] | 554,5506734 | 0,59270112 | **1,50806763** | 9,11540807 | 10,2286593 | 0,001382751 | 0,006794405 | protein_coding |
| ENSMUSG00000015536 | Mocs2 | molybdenum cofactor synthesis 2 [Source:MGI Symbol;Acc:MGI:1336894] | 31,43289081 | 0,59260669 | **1,50796892** | 4,97697442 | 14,96202 | 0,000109697 | 0,000739412 | protein_coding |
| ENSMUSG00000029050 | Ski | ski sarcoma viral oncogene homolog (avian) [Source:MGI Symbol;Acc:MGI:98310] | 93,89865902 | 0,59202873 | **1,50736492** | 6,55122363 | 19,8176008 | 8,51946E-06 | 7,53043E-05 | protein_coding |
| ENSMUSG00000038517 | Tbkbp1 | TBK1 binding protein 1 [Source:MGI Symbol;Acc:MGI:1920424] | 29,8730881 | 0,59197568 | **1,50730951** | 4,90260193 | 14,1513044 | 0,00016868 | 0,001081896 | protein_coding |
| ENSMUSG00000059588 | Calcrl | calcitonin receptor-like [Source:MGI Symbol;Acc:MGI:1926944] | 28,34958738 | 0,5918418 | **1,50716963** | 4,82979283 | 9,38067412 | 0,002192849 | 0,010148623 | protein_coding |
| ENSMUSG00000026970 | Rbms1 | RNA binding motif, single stranded interacting protein 1 [Source:MGI Symbol;Acc:MGI:1861774] | 168,7462436 | 0,59110842 | **1,50640367** | 7,39937774 | 17,6823601 | 2,61036E-05 | 0,000205739 | protein_coding |
| ENSMUSG00000039810 | Zc3h10 | zinc finger CCCH type containing 10 [Source:MGI Symbol;Acc:MGI:2143670] | 15,64548161 | 0,59054875 | **1,5058194** | 3,9745619 | 10,4427278 | 0,001231333 | 0,006138228 | protein_coding |
| ENSMUSG00000066571 | 4931406P16Rik | RIKEN cDNA 4931406P16 gene [Source:MGI Symbol;Acc:MGI:1924311] | 21,88082576 | 0,58887961 | **1,50407823** | 4,46158369 | 12,3423252 | 0,000442803 | 0,002518156 | protein_coding |
| ENSMUSG00000027281 | 2210009G21Rik | RIKEN cDNA 2210009G21 gene [Source:MGI Symbol;Acc:MGI:1921493] | 10,56993351 | 0,5887643 | **1,50395802** | 3,40783225 | 7,9747436 | 0,004743447 | 0,019359679 | protein_coding |
| ENSMUSG00000026596 | Abl2 | v-abl Abelson murine leukemia viral oncogene 2 (arg, Abelson-related gene) [Source:MGI Symbol;Acc:MGI:87860] | 92,16095378 | 0,58793322 | **1,5030919** | 6,52851927 | 17,2085502 | 3,34926E-05 | 0,000256077 | protein_coding |
| ENSMUSG00000018796 | Acsl1 | acyl-CoA synthetase long-chain family member 1 [Source:MGI Symbol;Acc:MGI:102797] | 59,8413048 | 0,58670965 | **1,50181765** | 5,90587796 | 18,436268 | 1,75682E-05 | 0,00014502 | protein_coding |
| ENSMUSG00000026820 | Ptges2 | prostaglandin E synthase 2 [Source:MGI Symbol;Acc:MGI:1917592] | 12,67302706 | 0,58603142 | **1,50111179** | 3,66419475 | 9,06544545 | 0,002604851 | 0,011724496 | protein_coding |

| | | | | | | | | | | |
|---|---|---|---|---|---|---|---|---|---|---|
| ENSMUSG00000026509 | Capn2 | calpain 2 [Source:MGI Symbol;Acc:MGI:88264] | 218,7096643 | 0,58534528 | **1,50039803** | 7,77337047 | 15,6348726 | 7,68247E-05 | 0,000542115 | protein_coding |
| ENSMUSG00000022335 | Zfat | zinc finger and AT hook domain containing [Source:MGI Symbol;Acc:MGI:2681865] | 13,87862457 | 0,58534081 | **1,50039339** | 3,79867046 | 8,3722122 | 0,003810016 | 0,01616241 | protein_coding |
| ENSMUSG00000025986 | Slc39a10 | solute carrier family 39 (zinc transporter), member 10 [Source:MGI Symbol;Acc:MGI:1914515] | 25,03512127 | 0,58441631 | **1,49943222** | 4,65372453 | 15,3479354 | 8,94186E-05 | 0,000619056 | protein_coding |
| ENSMUSG00000019066 | Rab3d | RAB3D, member RAS oncogene family [Source:MGI Symbol;Acc:MGI:97844] | 24,81522457 | 0,58428328 | **1,49929396** | 4,63935629 | 13,913716 | 0,000191397 | 0,001207161 | protein_coding |
| ENSMUSG00000022514 | Il1rap | interleukin 1 receptor accessory protein [Source:MGI Symbol;Acc:MGI:104975] | 10,22609224 | 0,58418839 | **1,49919536** | 3,36675255 | 6,33360152 | 0,011847149 | 0,041799817 | protein_coding |
| ENSMUSG00000035133 | Arhgap5 | Rho GTPase activating protein 5 [Source:MGI Symbol;Acc:MGI:1332637] | 21,5704189 | 0,58350081 | **1,49848102** | 4,43209847 | 12,8240813 | 0,000342186 | 0,002009408 | protein_coding |
| ENSMUSG00000079252 | Tor1aip2 | torsin A interacting protein 2 [Source:MGI Symbol;Acc:MGI:3582695] | 121,150145 | 0,58224277 | **1,4971749** | 6,92399301 | 15,3036366 | 9,15401E-05 | 0,00063017 | protein_coding |
| ENSMUSG00000028961 | Pgd | phosphogluconate dehydrogenase [Source:MGI Symbol;Acc:MGI:97553] | 423,3900395 | 0,58208614 | **1,49701236** | 8,72637367 | 11,4702431 | 0,000707194 | 0,003799483 | protein_coding |
| ENSMUSG00000046791 | 2410016O06Rik | RIKEN cDNA 2410016O06 gene [Source:MGI Symbol;Acc:MGI:1919202] | 10,93269506 | 0,58193711 | **1,49685773** | 3,45585811 | 7,31569781 | 0,006835485 | 0,026406989 | protein_coding |
| ENSMUSG00000039512 | Uhrf1bp1 | UHRF1 (ICBP90) binding protein 1 [Source:MGI Symbol;Acc:MGI:3041238] | 43,20521462 | 0,58158229 | **1,49648964** | 5,43378521 | 18,0367275 | 2,16684E-05 | 0,000174795 | protein_coding |
| ENSMUSG00000035969 | Rusc2 | RUN and SH3 domain containing 2 [Source:MGI Symbol;Acc:MGI:2140371] | 82,13031036 | 0,58131007 | **1,49620729** | 6,35943463 | 12,5178096 | 0,000403091 | 0,00232359 | protein_coding |
| ENSMUSG00000047534 | Mis18bp1 | MIS18 binding protein 1 [Source:MGI Symbol;Acc:MGI:2145099] | 9,107988098 | 0,57959101 | **1,49442554** | 3,19672255 | 6,27224587 | 0,012264359 | 0,042920632 | protein_coding |
| ENSMUSG00000051149 | Adnp | activity-dependent neuroprotective protein [Source:MGI Symbol;Acc:MGI:1338758] | 27,21808114 | 0,57884881 | **1,49365692** | 4,76761411 | 9,54115786 | 0,00200915 | 0,009405422 | protein_coding |
| ENSMUSG00000038178 | Slc43a2 | solute carrier family 43, member 2 [Source:MGI Symbol;Acc:MGI:2442746] | 365,0363087 | 0,57877704 | **1,49358261** | 8,51174316 | 12,8794843 | 0,000332204 | 0,001957063 | protein_coding |
| ENSMUSG00000016487 | Ppfibp1 | PTPRF interacting protein, binding protein 1 (liprin beta 1) [Source:MGI Symbol;Acc:MGI:1914783] | 75,15235452 | 0,57788904 | **1,49266357** | 6,2339365 | 15,268258 | 9,32708E-05 | 0,000640879 | protein_coding |
| ENSMUSG00000030223 | Ptpro | protein tyrosine phosphatase, receptor type, O [Source:MGI Symbol;Acc:MGI:1097152] | 22,38755627 | 0,57717354 | **1,49192347** | 4,49071483 | 10,236632 | 0,001376787 | 0,006768127 | protein_coding |
| ENSMUSG00000017774 | Myo1c | myosin IC [Source:MGI Symbol;Acc:MGI:106612] | 254,2161018 | 0,57709034 | **1,49183745** | 7,99031957 | 15,6971919 | 7,43344E-05 | 0,000527252 | protein_coding |
| ENSMUSG00000009418 | Nav1 | neuron navigator 1 [Source:MGI Symbol;Acc:MGI:2183683] | 94,39898891 | 0,57661252 | **1,49134343** | 6,56278376 | 15,3122674 | 9,11228E-05 | 0,000628084 | protein_coding |
| ENSMUSG00000071547 | Nt5dc2 | 5'-nucleotidase domain containing 2 [Source:MGI Symbol;Acc:MGI:1917271] | 15,35697883 | 0,57659363 | **1,4913239** | 3,94958162 | 8,41038434 | 0,003730838 | 0,015931098 | protein_coding |
| ENSMUSG00000015312 | Gadd45b | growth arrest and DNA-damage-inducible 45 beta [Source:MGI Symbol;Acc:MGI:107776] | 57,25206192 | 0,57617739 | **1,49089369** | 5,84096269 | 18,6016775 | 1,61079E-05 | 0,000134481 | protein_coding |
| ENSMUSG00000021279 | Cdc42bpb | CDC42 binding protein kinase beta [Source:MGI Symbol;Acc:MGI:2136459] | 92,01966118 | 0,57600974 | **1,49072045** | 6,52456 | 17,5358819 | 2,81936E-05 | 0,000220003 | protein_coding |
| ENSMUSG00000021918 | Nek4 | NIMA (never in mitosis gene a)-related expressed kinase 4 [Source:MGI Symbol;Acc:MGI:1344404] | 10,0857577 | 0,57317361 | **1,48779279** | 3,33679643 | 7,38499347 | 0,006577031 | 0,025543244 | protein_coding |
| ENSMUSG00000079254 | Itprip | inositol 1,4,5-triphosphate receptor interacting protein [Source:MGI Symbol;Acc:MGI:3042776] | 9,067437811 | 0,57265865 | **1,48726183** | 3,18598656 | 6,77158616 | 0,009262048 | 0,034102588 | protein_coding |
| ENSMUSG00000031360 | Ctps2 | cytidine 5'-triphosphate synthase 2 [Source:MGI Symbol;Acc:MGI:1933185] | 37,23430659 | 0,57203316 | **1,48661716** | 5,22459003 | 15,3971446 | 8,71198E-05 | 0,000605431 | protein_coding |
| ENSMUSG00000039789 | Zfp597 | zinc finger protein 597 [Source:MGI Symbol;Acc:MGI:1918313] | 10,63381006 | 0,57001038 | **1,48453425** | 3,41246905 | 7,66280737 | 0,005637072 | 0,022409565 | protein_coding |
| ENSMUSG00000027469 | Tpx2 | TPX2, microtubule-associated protein homolog (Xenopus laevis) [Source:MGI Symbol;Acc:MGI:1919369] | 18,25970307 | 0,56838377 | **1,48286141** | 4,20257733 | 10,2569041 | 0,00136174 | 0,006697152 | protein_coding |
| ENSMUSG00000035469 | Rcbtb1 | regulator of chromosome condensation (RCC1) and BTB (POZ) domain containing protein 1 [Source:MGI Symbol;Acc:M | 29,83728009 | 0,56799792 | **1,48246487** | 4,90582221 | 12,4851861 | 0,000410192 | 0,00235798 | protein_coding |
| ENSMUSG00000002985 | Apoe | apolipoprotein E [Source:MGI Symbol;Acc:MGI:88057] | 1003,168218 | 0,56719583 | **1,4816409** | 9,97038823 | 8,36833346 | 0,003818156 | 0,016180786 | protein_coding |
| ENSMUSG00000010663 | Fads1 | fatty acid desaturase 1 [Source:MGI Symbol;Acc:MGI:1923517] | 61,63681021 | 0,5668531 | **1,48128896** | 5,94579344 | 17,4076753 | 3,01606E-05 | 0,000233691 | protein_coding |
| ENSMUSG00000007659 | Bcl2l1 | BCL2-like 1 [Source:MGI Symbol;Acc:MGI:88139] | 103,4980691 | 0,56675441 | **1,48118763** | 6,69555871 | 18,3176349 | 1,86968E-05 | 0,000152503 | protein_coding |
| ENSMUSG00000029446 | Psph | phosphoserine phosphatase [Source:MGI Symbol;Acc:MGI:97788] | 10,16543427 | 0,56674724 | **1,48118028** | 3,35083175 | 6,63051847 | 0,010024608 | 0,03649449 | protein_coding |
| ENSMUSG00000021687 | Scamp1 | secretory carrier membrane protein 1 [Source:MGI Symbol;Acc:MGI:1349480] | 71,78794767 | 0,56616262 | **1,48058018** | 6,16991956 | 17,4004389 | 3,02756E-05 | 0,000234417 | protein_coding |
| ENSMUSG00000062232 | Rapgef2 | Rap guanine nucleotide exchange factor (GEF) 2 [Source:MGI Symbol;Acc:MGI:2659071] | 122,6807447 | 0,56599482 | **1,48040799** | 6,94528822 | 18,0759977 | 2,1226E-05 | 0,000171604 | protein_coding |
| ENSMUSG00000046675 | Tmem251 | transmembrane protein 251 [Source:MGI Symbol;Acc:MGI:2443862] | 19,3778537 | 0,5655268 | **1,4799278** | 4,27931139 | 9,80112347 | 0,001744053 | 0,008373791 | protein_coding |
| ENSMUSG00000074151 | Nlrc5 | NLR family, CARD domain containing 5 [Source:MGI Symbol;Acc:MGI:3612191] | 70,4673034 | 0,56374219 | **1,47809827** | 6,1409981 | 16,5540629 | 4,72826E-05 | 0,000349357 | lincRNA |
| ENSMUSG00000040997 | Abhd4 | abhydrolase domain containing 4 [Source:MGI Symbol;Acc:MGI:1915938] | 63,27954467 | 0,56270351 | **1,47703448** | 5,98641759 | 14,8417003 | 0,000116921 | 0,000782678 | protein_coding |
| ENSMUSG00000026134 | Prim2 | DNA primase, p58 subunit [Source:MGI Symbol;Acc:MGI:97758] | 8,822168452 | 0,56235206 | **1,47667472** | 3,15632265 | 6,01629303 | 0,014174389 | 0,048051222 | protein_coding |
| ENSMUSG00000032846 | Zswim6 | zinc finger, SWIM domain containing 6 [Source:MGI Symbol;Acc:MGI:1914513] | 46,15913374 | 0,56227264 | **1,47659342** | 5,53700359 | 16,9230608 | 3,89258E-05 | 0,000293332 | protein_coding |
| ENSMUSG00000059689 | Zfp637 | zinc finger protein 637 [Source:MGI Symbol;Acc:MGI:2448537] | 10,27241861 | 0,56175447 | **1,47606318** | 3,3644606 | 7,18878415 | 0,007336067 | 0,027996308 | protein_coding |
| ENSMUSG00000024085 | Man2a1 | mannosidase 2, alpha 1 [Source:MGI Symbol;Acc:MGI:104669] | 70,13277085 | 0,56172845 | **1,47603655** | 6,13535534 | 14,2862079 | 0,000157011 | 0,001014157 | protein_coding |
| ENSMUSG00000024529 | Lox | lysyl oxidase [Source:MGI Symbol;Acc:MGI:96817] | 304,0596486 | 0,56079838 | **1,4750853** | 8,24864088 | 12,53286 | 0,000399857 | 0,00230737 | protein_coding |
| ENSMUSG00000006715 | Gmnn | geminin [Source:MGI Symbol;Acc:MGI:1927344] | 12,21435295 | 0,56056967 | **1,47485147** | 3,62283565 | 7,17084411 | 0,00740979 | 0,028218844 | protein_coding |
| ENSMUSG00000036833 | Pnpla7 | patatin-like phospholipase domain containing 7 [Source:MGI Symbol;Acc:MGI:2385325] | 75,12178562 | 0,56046099 | **1,47474037** | 6,22917224 | 16,3954816 | 5,14076E-05 | 0,000377803 | protein_coding |
| ENSMUSG00000029156 | Sgcb | sarcoglycan, beta (dystrophin-associated glycoprotein) [Source:MGI Symbol;Acc:MGI:1346523] | 18,88737692 | 0,55919457 | **1,47344638** | 4,24581455 | 10,3399917 | 0,001301786 | 0,006439767 | protein_coding |
| ENSMUSG00000023175 | Bsg | basigin [Source:MGI Symbol;Acc:MGI:88208] | 179,3481716 | 0,55861315 | **1,4728527** | 7,4876538 | 16,5663334 | 4,69776E-05 | 0,000347571 | protein_coding |
| ENSMUSG00000066150 | Slc31a1 | solute carrier family 31, member 1 [Source:MGI Symbol;Acc:MGI:1333843] | 113,5106309 | 0,5582221 | **1,47245353** | 6,8300401 | 18,1104373 | 2,08456E-05 | 0,000169526 | protein_coding |
| ENSMUSG00000051934 | Spats2 | spermatogenesis associated, serine-rich 2 [Source:MGI Symbol;Acc:MGI:1919822] | 15,0526807 | 0,55732583 | **1,47153905** | 3,92499265 | 9,40366405 | 0,002165522 | 0,010043308 | protein_coding |
| ENSMUSG00000001482 | Def8 | differentially expressed in FDCP 8 [Source:MGI Symbol;Acc:MGI:1346331] | 35,50600095 | 0,55664966 | **1,47084952** | 5,15048418 | 13,3651695 | 0,000256341 | 0,001556031 | protein_coding |
| ENSMUSG00000025503 | Taldo1 | transaldolase 1 [Source:MGI Symbol;Acc:MGI:1274789] | 234,1491879 | 0,55659014 | **1,47078884** | 7,87233238 | 11,285718 | 0,000781056 | 0,004149644 | protein_coding |
| ENSMUSG00000014778 | Fhod1 | formin homology 2 domain containing 1 [Source:MGI Symbol;Acc:MGI:2679008] | 19,48311487 | 0,55624827 | **1,47044036** | 4,28169233 | 11,4799327 | 0,000703517 | 0,003785279 | protein_coding |
| ENSMUSG00000024014 | Pim1 | proviral integration site 1 [Source:MGI Symbol;Acc:MGI:97584] | 92,61830242 | 0,55611692 | **1,47030649** | 6,53610803 | 18,631985 | 1,58538E-05 | 0,000132562 | protein_coding |
| ENSMUSG00000002250 | Ppard | peroxisome proliferator activator receptor delta [Source:MGI Symbol;Acc:MGI:101884] | 28,79276244 | 0,55497935 | **1,4691476** | 4,84895649 | 12,5357462 | 0,00039924 | 0,002305019 | protein_coding |
| ENSMUSG00000033793 | Atp6v1h | ATPase, H+ transporting, lysosomal V1 subunit H [Source:MGI Symbol;Acc:MGI:1914864] | 162,6795116 | 0,55429246 | **1,46844829** | 7,34747955 | 14,703933 | 0,000125784 | 0,000835037 | protein_coding |
| ENSMUSG00000056708 | Ier5 | immediate early response 5 [Source:MGI Symbol;Acc:MGI:1337072] | 85,88671141 | 0,55142542 | **1,46553297** | 6,42327097 | 15,9359072 | 6,55238E-05 | 0,000468997 | protein_coding |
| ENSMUSG00000034947 | Tmem106a | transmembrane protein 106A [Source:MGI Symbol;Acc:MGI:1922056] | 229,7630424 | 0,55073276 | **1,46482951** | 7,84486981 | 13,9188298 | 0,000190877 | 0,001204573 | protein_coding |
| ENSMUSG00000061666 | Gdpd1 | glycerophosphodiester phosphodiesterase domain containing 1 [Source:MGI Symbol;Acc:MGI:1913819] | 23,07397402 | 0,54920353 | **1,46327764** | 4,53375304 | 12,1619571 | 0,000487742 | 0,002746703 | protein_coding |
| ENSMUSG00000001521 | Tulp3 | tubby-like protein 3 [Source:MGI Symbol;Acc:MGI:1329045] | 12,83251221 | 0,54862238 | **1,46268832** | 3,69119734 | 7,03951813 | 0,007973048 | 0,030082376 | protein_coding |
| ENSMUSG00000029103 | Lrpap1 | low density lipoprotein receptor-related protein associated protein 1 [Source:MGI Symbol;Acc:MGI:96829] | 138,1940255 | 0,5485188 | **1,46258331** | 7,1113218 | 13,9216767 | 0,000190588 | 0,001203441 | protein_coding |
| ENSMUSG00000030314 | Atg7 | autophagy related 7 [Source:MGI Symbol;Acc:MGI:1921494] | 60,38218057 | 0,54694091 | **1,46098454** | 5,91782783 | 14,883709 | 0,000114346 | 0,000767454 | protein_coding |
| ENSMUSG00000002814 | Top3a | topoisomerase (DNA) III alpha [Source:MGI Symbol;Acc:MGI:1197527] | 21,30456146 | 0,5468103 | **1,46085228** | 4,41517259 | 11,8550903 | 0,000575039 | 0,003170075 | protein_coding |
| ENSMUSG00000032462 | Pik3cb | phosphatidylinositol 3-kinase, catalytic, beta polypeptide [Source:MGI Symbol;Acc:MGI:1922019] | 47,24758897 | 0,54667437 | **1,46071464** | 5,56537793 | 11,8894723 | 0,000564521 | 0,003116781 | protein_coding |
| ENSMUSG00000050002 | Idnk | idnK gluconokinase homolog (E. coli) [Source:MGI Symbol;Acc:MGI:1922981] | 9,668813604 | 0,54667352 | **1,46071378** | 3,28636047 | 6,05078545 | 0,013900112 | 0,047311194 | protein_coding |
| ENSMUSG00000044345 | Marveld1 | MARVEL (membrane-associating) domain containing 1 [Source:MGI Symbol;Acc:MGI:2147570] | 17,67314621 | 0,54576557 | **1,45979478** | 4,16204325 | 9,37303521 | 0,002202006 | 0,010182424 | protein_coding |
| ENSMUSG00000032231 | Anxa2 | annexin A2 [Source:MGI Symbol;Acc:MGI:88246] | 592,962299 | 0,54465798 | **1,4586745** | 9,21194136 | 10,0858245 | 0,001494143 | 0,007273438 | protein_coding |
| ENSMUSG00000023104 | Rfc2 | replication factor C (activator 1) 2 [Source:MGI Symbol;Acc:MGI:1341868] | 31,56298268 | 0,5442611 | **1,45827328** | 4,97953096 | 12,8163817 | 0,000343598 | 0,00201554 | protein_coding |
| ENSMUSG00000059495 | Arhgef12 | Rho guanine nucleotide exchange factor (GEF) 12 [Source:MGI Symbol;Acc:MGI:1916882] | 49,22035721 | 0,54383267 | **1,45784028** | 5,62905415 | 14,1275088 | 0,000170827 | 0,001090575 | protein_coding |
| ENSMUSG00000025477 | Inpp5a | inositol polyphosphate-5-phosphatase A [Source:MGI Symbol;Acc:MGI:2686961] | 15,52382412 | 0,54295658 | **1,45695526** | 3,95916885 | 9,3603567 | 0,00221729 | 0,010231568 | protein_coding |
| ENSMUSG00000062127 | Cttnbp2nl | CTTNBP2 N-terminal like [Source:MGI Symbol;Acc:MGI:1933137] | 102,4875693 | 0,54189525 | **1,45588384** | 6,68217055 | 15,9545329 | 6,48822E-05 | 0,000465922 | protein_coding |
| ENSMUSG00000020231 | Dip2a | DIP2 disco-interacting protein 2 homolog A (Drosophila) [Source:MGI Symbol;Acc:MGI:2385920] | 44,19243685 | 0,54130545 | **1,45528876** | 5,46924975 | 14,6223401 | 0,000131348 | 0,000867783 | protein_coding |
| ENSMUSG00000018398 | Sept8 | septin 8 [Source:MGI Symbol;Acc:MGI:894310] | 104,8797851 | 0,54082056 | **1,45479973** | 6,71439678 | 16,8538501 | 4,03714E-05 | 0,000302359 | protein_coding |
| ENSMUSG00000019726 | Lyst | lysosomal trafficking regulator [Source:MGI Symbol;Acc:MGI:107448] | 89,42505709 | 0,53930484 | **1,45327209** | 6,48273374 | 13,6871971 | 0,000215921 | 0,001339542 | protein_coding |

| | | | | | | | | | | |
|---|---|---|---|---|---|---|---|---|---|---|
| ENSMUSG00000066026 | Dhrs3 | dehydrogenase/reductase (SDR family) member 3 [Source:MGI Symbol;Acc:MGI:1315215] | 116,3354817 | 0,538709 | **1,452672** | 6,86245219 | 17,2115764 | 3,34392E-05 | 0,000255847 | protein_coding |
| ENSMUSG00000022967 | Ifnar1 | interferon (alpha and beta) receptor 1 [Source:MGI Symbol;Acc:MGI:107658] | 115,7469803 | 0,53787058 | **1,45182803** | 6,85678401 | 17,6357036 | 2,67519E-05 | 0,000210245 | protein_coding |
| ENSMUSG00000052566 | Hook2 | hook homolog 2 (Drosophila) [Source:MGI Symbol;Acc:MGI:2181664] | 24,40668872 | 0,53748669 | **1,45144177** | 4,61512766 | 10,5467983 | 0,001163895 | 0,005841808 | protein_coding |
| ENSMUSG00000028980 | H6pd | hexose-6-phosphate dehydrogenase (glucose 1-dehydrogenase) [Source:MGI Symbol;Acc:MGI:2140356] | 69,47886828 | 0,53657715 | **1,450527** | 6,11985438 | 14,3315873 | 0,000153271 | 0,000994093 | protein_coding |
| ENSMUSG00000019978 | Epb4.1l2 | erythrocyte protein band 4.1-like 2 [Source:MGI Symbol;Acc:MGI:103009] | 111,4011722 | 0,53622576 | **1,45017374** | 6,80100624 | 13,6849988 | 0,000216174 | 0,001339599 | protein_coding |
| ENSMUSG00000034640 | Tiparp | TCDD-inducible poly(ADP-ribose) polymerase [Source:MGI Symbol;Acc:MGI:2159210] | 74,49251141 | 0,53596675 | **1,44991342** | 6,22323314 | 15,9842253 | 6,38725E-05 | 0,000458971 | protein_coding |
| ENSMUSG00000033880 | Lgals3bp | lectin, galactoside-binding, soluble, 3 binding protein [Source:MGI Symbol;Acc:MGI:99554] | 444,5586457 | 0,53503303 | **1,44897533** | 8,79645429 | 8,32600592 | 0,003908149 | 0,016489549 | protein_coding |
| ENSMUSG00000022564 | Grina | glutamate receptor, ionotropic, N-methyl D-aspartate-associated protein 1 (glutamate binding) [Source:MGI Symbol;Acc: | 281,3312666 | 0,53421395 | **1,44815292** | 8,13627768 | 12,5555018 | 0,000395042 | 0,002284379 | protein_coding |
| ENSMUSG00000030533 | Unc45a | unc-45 homolog A (C. elegans) [Source:MGI Symbol;Acc:MGI:2142246] | 27,6053512 | 0,53252481 | **1,44645838** | 4,78898505 | 12,6740943 | 0,000370757 | 0,00216101 | protein_coding |
| ENSMUSG00000005514 | Por | P450 (cytochrome) oxidoreductase [Source:MGI Symbol;Acc:MGI:97744] | 421,1811486 | 0,53107171 | **1,44500222** | 8,71850048 | 9,73930033 | 0,001803695 | 0,008616404 | protein_coding |
| ENSMUSG00000028080 | Lrba | LPS-responsive beige-like anchor [Source:MGI Symbol;Acc:MGI:1933162] | 13,12551957 | 0,53099414 | **1,44492453** | 3,72235055 | 7,1318626 | 0,007572602 | 0,028769081 | protein_coding |
| ENSMUSG00000031214 | Ophn1 | oligophrenin 1 [Source:MGI Symbol;Acc:MGI:2151070] | 62,87261641 | 0,53069187 | **1,44462183** | 5,97922108 | 15,2994167 | 9,17448E-05 | 0,000631184 | protein_coding |
| ENSMUSG00000040006 | Ginm1 | glycoprotein integral membrane 1 [Source:MGI Symbol;Acc:MGI:2384905] | 24,13498103 | 0,53061498 | **1,44454484** | 4,60597354 | 11,4287247 | 0,000723173 | 0,00387964 | protein_coding |
| ENSMUSG00000028842 | Eif2c3 | eukaryotic translation initiation factor 2C, 3 [Source:MGI Symbol;Acc:MGI:2446634] | 72,22344808 | 0,52941401 | **1,44334282** | 6,17721614 | 14,5413302 | 0,000137118 | 0,000899413 | protein_coding |
| ENSMUSG00000040339 | Fam102b | family with sequence similarity 102, member B [Source:MGI Symbol;Acc:MGI:3036259] | 49,3769297 | 0,5290285 | **1,4429572** | 5,62438118 | 15,2138491 | 9,59968E-05 | 0,000657145 | protein_coding |
| ENSMUSG00000051341 | Zfp52 | zinc finger protein 52 [Source:MGI Symbol;Acc:MGI:99199] | 17,80769829 | 0,52761733 | **1,44154646** | 4,15367093 | 10,584087 | 0,001140652 | 0,005738253 | protein_coding |
| ENSMUSG00000030161 | Gabarapl1 | gamma-aminobutyric acid (GABA) A receptor-associated protein-like 1 [Source:MGI Symbol;Acc:MGI:1914980] | 107,5598065 | 0,52690077 | **1,44083065** | 6,75139597 | 13,7900524 | 0,000204416 | 0,001276814 | protein_coding |
| ENSMUSG00000019916 | P4ha1 | procollagen-proline, 2-oxoglutarate 4-dioxygenase (proline 4-hydroxylase), alpha 1 polypeptide [Source:MGI Symbol;Acc | 91,10373119 | 0,52656385 | **1,4404942** | 6,51612427 | 13,1119542 | 0,000293417 | 0,00175396 | protein_coding |
| ENSMUSG00000085795 | Zfp703 | zinc finger protein 703 [Source:MGI Symbol;Acc:MGI:2662729] | 48,08878778 | 0,52615643 | **1,44008746** | 5,59286256 | 12,6971479 | 0,000366214 | 0,002137933 | protein_coding |
| ENSMUSG00000038866 | Zcchc2 | zinc finger, CCHC domain containing 2 [Source:MGI Symbol;Acc:MGI:2444114] | 83,3390137 | 0,52576393 | **1,43969572** | 6,38241163 | 15,0281835 | 0,000105918 | 0,000716574 | protein_coding |
| ENSMUSG00000039206 | Daglb | diacylglycerol lipase, beta [Source:MGI Symbol;Acc:MGI:2442032] | 60,17089085 | 0,52540637 | **1,43933895** | 5,9101013 | 13,872993 | 0,000195589 | 0,001230777 | protein_coding |
| ENSMUSG00000029385 | Ccng2 | cyclin G2 [Source:MGI Symbol;Acc:MGI:1095734] | 18,52651014 | 0,52409949 | **1,4380357** | 4,21301613 | 10,2165119 | 0,001391888 | 0,006830138 | protein_coding |
| ENSMUSG00000053398 | Phgdh | 3-phosphoglycerate dehydrogenase [Source:MGI Symbol;Acc:MGI:1355330] | 17,81692683 | 0,52401203 | **1,43794852** | 4,14107513 | 6,44601304 | 0,011120161 | 0,039655051 | protein_coding |
| ENSMUSG00000066877 | Nck2 | non-catalytic region of tyrosine kinase adaptor protein 2 [Source:MGI Symbol;Acc:MGI:1306821] | 30,93634012 | 0,52382022 | **1,43775736** | 4,95585017 | 9,98648181 | 0,001576936 | 0,007635873 | protein_coding |
| ENSMUSG00000054808 | Actn4 | actinin alpha 4 [Source:MGI Symbol;Acc:MGI:1890773] | 237,9898906 | 0,52289532 | **1,43683592** | 7,89504308 | 11,2173947 | 0,000810342 | 0,004286579 | protein_coding |
| ENSMUSG00000027132 | 2410042D21Rik | RIKEN cDNA 2410042D21 gene [Source:MGI Symbol;Acc:MGI:1919675] | 25,13742659 | 0,52287014 | **1,43681084** | 4,65904637 | 9,08276802 | 0,002580292 | 0,01162348 | protein_coding |
| ENSMUSG00000036968 | Cnpy4 | canopy 4 homolog (zebrafish) [Source:MGI Symbol;Acc:MGI:1913705] | 13,78587627 | 0,5226706 | **1,43661212** | 3,79917238 | 7,66681422 | 0,005624568 | 0,022383206 | protein_coding |
| ENSMUSG00000049295 | Zfp219 | zinc finger protein 219 [Source:MGI Symbol;Acc:MGI:1917140] | 25,62437241 | 0,52252928 | **1,43647141** | 4,6761583 | 10,7197236 | 0,001059995 | 0,005393734 | protein_coding |
| ENSMUSG00000004221 | Ikbkg | inhibitor of kappaB kinase gamma [Source:MGI Symbol;Acc:MGI:1338074] | 93,02629182 | 0,52206707 | **1,43601127** | 6,541126 | 13,7273456 | 0,000211355 | 0,001314179 | protein_coding |
| ENSMUSG00000032469 | Dbr1 | debranching enzyme homolog 1 (S. cerevisiae) [Source:MGI Symbol;Acc:MGI:1931520] | 11,3159699 | 0,52195222 | **1,43589696** | 3,50103798 | 6,63504067 | 0,009999191 | 0,036426099 | protein_coding |
| ENSMUSG00000004846 | Plod3 | procollagen-lysine, 2-oxoglutarate 5-dioxygenase 3 [Source:MGI Symbol;Acc:MGI:1347008] | 80,40071736 | 0,52137983 | **1,43532738** | 6,33050133 | 13,320566 | 0,000262511 | 0,001590848 | protein_coding |
| ENSMUSG00000022558 | Heatr7a | HEAT repeat containing 7A [Source:MGI Symbol;Acc:MGI:2442558] | 199,5205303 | 0,52124797 | **1,4351962** | 7,64042022 | 11,1941038 | 0,000820577 | 0,004334459 | protein_coding |
| ENSMUSG00000024560 | Cxxc1 | CXXC finger 1 (PHD domain) [Source:MGI Symbol;Acc:MGI:1921572] | 38,32961763 | 0,52070171 | **1,43465287** | 5,26080475 | 13,5708789 | 0,000229722 | 0,001413188 | protein_coding |
| ENSMUSG00000027187 | Cat | catalase [Source:MGI Symbol;Acc:MGI:88271] | 408,8903968 | 0,52047322 | **1,43442568** | 8,67625983 | 6,63598133 | 0,009993913 | 0,036418945 | protein_coding |
| ENSMUSG00000029821 | Dfna5 | deafness, autosomal dominant 5 (human) [Source:MGI Symbol;Acc:MGI:1889850] | 13,39735362 | 0,51961453 | **1,43357217** | 3,74124999 | 7,48037664 | 0,006237502 | 0,024423178 | protein_coding |
| ENSMUSG00000014353 | Tmem87b | transmembrane protein 87B [Source:MGI Symbol;Acc:MGI:1919727] | 71,86255538 | 0,51948648 | **1,43344493** | 6,16851265 | 13,9442723 | 0,000188311 | 0,001191116 | protein_coding |
| ENSMUSG00000034659 | Tmem109 | transmembrane protein 109 [Source:MGI Symbol;Acc:MGI:1915789] | 44,11387919 | 0,51886065 | **1,43282325** | 5,46684389 | 10,2780066 | 0,001346253 | 0,006638817 | protein_coding |
| ENSMUSG00000044629 | Cnrip1 | cannabinoid receptor interacting protein 1 [Source:MGI Symbol;Acc:MGI:1917505] | 9,893825165 | 0,51878173 | **1,43274487** | 3,31137991 | 6,09702591 | 0,013540959 | 0,046361644 | protein_coding |
| ENSMUSG00000021843 | Ktn1 | kinectin 1 [Source:MGI Symbol;Acc:MGI:109153] | 35,89910489 | 0,51842787 | **1,43239349** | 5,17057968 | 10,9871058 | 0,00091748 | 0,00476162 | protein_coding |
| ENSMUSG00000042650 | Alkbh5 | alkB, alkylation repair homolog 5 (E. coli) [Source:MGI Symbol;Acc:MGI:2144489] | 96,22160841 | 0,51804856 | **1,43201694** | 6,58985887 | 15,0483382 | 0,000104792 | 0,000710275 | protein_coding |
| ENSMUSG00000021871 | Pnp | purine-nucleoside phosphorylase [Source:MGI Symbol;Acc:MGI:97365] | 191,0159933 | 0,51747002 | **1,4314428** | 7,57964053 | 14,7215532 | 0,000124614 | 0,000828771 | protein_coding |
| ENSMUSG00000031400 | G6pdx | glucose-6-phosphate dehydrogenase X-linked [Source:MGI Symbol;Acc:MGI:105979] | 203,0530671 | 0,51740129 | **1,43137461** | 7,66837894 | 11,4619909 | 0,000710342 | 0,003814527 | protein_coding |
| ENSMUSG00000040043 | Rbms2 | RNA binding motif, single stranded interacting protein 2 [Source:MGI Symbol;Acc:MGI:1861776] | 27,66959678 | 0,51684648 | **1,43082425** | 4,79934167 | 10,9286517 | 0,000946884 | 0,004895724 | protein_coding |
| ENSMUSG00000034216 | Vps18 | vacuolar protein sorting 18 (yeast) [Source:MGI Symbol;Acc:MGI:2443626] | 121,0420067 | 0,5160654 | **1,43004982** | 6,91913688 | 14,2104479 | 0,00016346 | 0,001050872 | protein_coding |
| ENSMUSG00000024782 | Ak3 | adenylate kinase 3 [Source:MGI Symbol;Acc:MGI:1860835] | 34,94547296 | 0,5157622 | **1,4297493** | 5,1323021 | 11,2584097 | 0,000792631 | 0,00420301 | protein_coding |
| ENSMUSG00000025058 | 5430427O19Rik | RIKEN cDNA 5430427O19 gene [Source:MGI Symbol;Acc:MGI:1918648] | 11,25955245 | 0,51526099 | **1,42925268** | 3,49375767 | 6,08293907 | 0,01364935 | 0,04661654 | protein_coding |
| ENSMUSG00000017715 | Pgs1 | phosphatidylglycerophosphate synthase 1 [Source:MGI Symbol;Acc:MGI:1921701] | 94,43762732 | 0,51504072 | **1,42903448** | 6,56275347 | 15,5504957 | 8,03306E-05 | 0,000562256 | protein_coding |
| ENSMUSG00000046876 | Atxn1 | ataxin 1 [Source:MGI Symbol;Acc:MGI:104783] | 36,91460163 | 0,51502287 | **1,42901679** | 5,20409057 | 12,484148 | 0,00041042 | 0,00235798 | protein_coding |
| ENSMUSG00000097119 | | | 11,60673942 | 0,51452524 | **1,42852396** | 3,53220665 | 6,10499472 | 0,013480035 | 0,046196241 | lincRNA |
| ENSMUSG00000033365 | Ipo13 | importin 13 [Source:MGI Symbol;Acc:MGI:2385205] | 71,52600074 | 0,51247983 | **1,42650008** | 6,16338627 | 13,7917142 | 0,000204235 | 0,001276411 | protein_coding |
| ENSMUSG00000020821 | Kif1c | kinesin family member 1C [Source:MGI Symbol;Acc:MGI:1098260] | 195,4171978 | 0,51241182 | **1,42643284** | 7,61100085 | 12,9360611 | 0,000322312 | 0,001903896 | protein_coding |
| ENSMUSG00000040225 | Prrc2c | proline-rich coiled-coil 2C [Source:MGI Symbol;Acc:MGI:1913754] | 250,1683981 | 0,51234624 | **1,426368** | 7,9676301 | 11,4220493 | 0,000725776 | 0,003891704 | protein_coding |
| ENSMUSG00000039886 | Tmem120a | transmembrane protein 120A [Source:MGI Symbol;Acc:MGI:2686991] | 55,46518699 | 0,51176176 | **1,42579025** | 5,79673914 | 13,2140428 | 0,000277859 | 0,001674625 | protein_coding |
| ENSMUSG00000067787 | Blcap | bladder cancer associated protein homolog (human) [Source:MGI Symbol;Acc:MGI:1858907] | 35,13874724 | 0,51148074 | **1,42551255** | 5,13861597 | 11,1942284 | 0,000820521 | 0,004334459 | protein_coding |
| ENSMUSG00000020097 | Sgpl1 | sphingosine phosphate lyase 1 [Source:MGI Symbol;Acc:MGI:1261415] | 608,6246577 | 0,51131036 | **1,42534421** | 9,2496324 | 8,52375307 | 0,003505408 | 0,015094242 | protein_coding |
| ENSMUSG00000026469 | Xpr1 | xenotropic and polytropic retrovirus receptor 1 [Source:MGI Symbol;Acc:MGI:97932] | 92,19026352 | 0,51120519 | **1,42524031** | 6,5280849 | 14,0448313 | 0,000178504 | 0,001133654 | protein_coding |
| ENSMUSG00000028567 | Txndc12 | thioredoxin domain containing 12 (endoplasmic reticulum) [Source:MGI Symbol;Acc:MGI:1913323] | 26,05114851 | 0,51078426 | **1,42482453** | 4,71269205 | 10,8454925 | 0,000990365 | 0,00509421 | protein_coding |
| ENSMUSG00000027628 | Aar2 | AAR2 splicing factor homolog (S. cerevisiae) [Source:MGI Symbol;Acc:MGI:1915545] | 35,43968394 | 0,51056773 | **1,4246107** | 5,15081994 | 12,9745858 | 0,000315748 | 0,001869138 | protein_coding |
| ENSMUSG00000032198 | Dock6 | dedicator of cytokinesis 6 [Source:MGI Symbol;Acc:MGI:1914789] | 16,97341195 | 0,51035085 | **1,42439656** | 4,089634 | 8,65606396 | 0,003259747 | 0,014172868 | protein_coding |
| ENSMUSG00000015846 | Rxra | retinoid X receptor alpha [Source:MGI Symbol;Acc:MGI:98214] | 66,19869163 | 0,50972896 | **1,42378269** | 6,05173012 | 10,432826 | 0,001237952 | 0,006167217 | protein_coding |
| ENSMUSG00000027340 | Slc23a2 | solute carrier family 23 (nucleobase transporters), member 2 [Source:MGI Symbol;Acc:MGI:1859682] | 182,6224622 | 0,50939159 | **1,42344977** | 7,51312044 | 12,4954481 | 0,000407945 | 0,002349103 | protein_coding |
| ENSMUSG00000023088 | Abcc1 | ATP-binding cassette, sub-family C (CFTR/MRP), member 1 [Source:MGI Symbol;Acc:MGI:102676] | 345,5260934 | 0,50919415 | **1,42325499** | 8,43361962 | 9,64858755 | 0,001894972 | 0,008935649 | protein_coding |
| ENSMUSG00000039208 | Metrnl | meteorin, glial cell differentiation regulator-like [Source:MGI Symbol;Acc:MGI:2384806] | 91,28197942 | 0,50898744 | **1,42305107** | 6,51424161 | 13,1398681 | 0,000289078 | 0,00173179 | protein_coding |
| ENSMUSG00000040751 | Lat2 | linker for activation of T cells family, member 2 [Source:MGI Symbol;Acc:MGI:1926479] | 144,3802748 | 0,50830793 | **1,42238098** | 7,17300385 | 11,6564806 | 0,000639793 | 0,003483351 | protein_coding |
| ENSMUSG00000063317 | Usp31 | ubiquitin specific peptidase 31 [Source:MGI Symbol;Acc:MGI:1923429] | 34,31671046 | 0,507908 | **1,42198673** | 5,1053244 | 12,5182723 | 0,000402991 | 0,00232359 | protein_coding |
| ENSMUSG00000003119 | Cdk12 | cyclin-dependent kinase 12 [Source:MGI Symbol;Acc:MGI:1098802] | 39,80486158 | 0,50776192 | **1,42184276** | 5,31882791 | 12,5903932 | 0,000387735 | 0,002251607 | protein_coding |
| ENSMUSG00000050552 | 0910001L09Rik | RIKEN cDNA 0910001L09 gene [Source:MGI Symbol;Acc:MGI:1913346] | 32,20459505 | 0,50763736 | **1,42172** | 5,00823185 | 10,701571 | 0,001070446 | 0,005437351 | protein_coding |
| ENSMUSG00000029254 | Stap1 | signal transducing adaptor family member 1 [Source:MGI Symbol;Acc:MGI:1926193] | 188,9825278 | 0,50761138 | **1,4216944** | 7,562986 | 10,7857549 | 0,001022842 | 0,005234263 | protein_coding |
| ENSMUSG00000073489 | Ifi204 | interferon activated gene 204 [Source:MGI Symbol;Acc:MGI:96429] | 97,53839808 | 0,50722814 | **1,42131678** | 6,61229807 | 10,9901877 | 0,000915955 | 0,004757411 | protein_coding |
| ENSMUSG00000027088 | Phospho2 | phosphatase, orphan 2 [Source:MGI Symbol;Acc:MGI:1920623] | 18,05599539 | 0,50607729 | **1,42018344** | 4,17204609 | 9,78611014 | 0,00175835 | 0,008425205 | protein_coding |
| ENSMUSG00000037134 | Prmt10 | protein arginine methyltransferase 10 (putative) [Source:MGI Symbol;Acc:MGI:2142651] | 24,03171466 | 0,50538004 | **1,41949723** | 4,59126111 | 9,79905219 | 0,001746018 | 0,008377076 | protein_coding |

| | | | | | | | | | | |
|---|---|---|---|---|---|---|---|---|---|---|
| ENSMUSG00000096188 | Cmtm4 | CKLF-like MARVEL transmembrane domain containing 4 [Source:MGI Symbol;Acc:MGI:2142888] | 35,32156127 | 0,5052642 | **1,41938327** | 5,14821176 | 9,6870794 | 0,001855683 | 0,008804108 | protein_coding |
| ENSMUSG00000097328 | Tnfsf12 | tumor necrosis factor (ligand) superfamily, member 12 [Source:MGI Symbol;Acc:MGI:1196259] | 37,41899512 | 0,50519701 | **1,41931716** | 5,22044437 | 9,19647307 | 0,002424819 | 0,011068336 | protein_coding |
| ENSMUSG00000006611 | Hfe | hemochromatosis [Source:MGI Symbol;Acc:MGI:109191] | 38,95487672 | 0,50451886 | **1,41865016** | 5,29014756 | 12,1925138 | 0,000479817 | 0,002711804 | protein_coding |
| ENSMUSG00000034361 | Cpne2 | copine II [Source:MGI Symbol;Acc:MGI:2387578] | 57,9874591 | 0,50387748 | **1,41801961** | 5,86063496 | 13,0344433 | 0,000305814 | 0,001815224 | protein_coding |
| ENSMUSG00000025764 | Phf17 | PHD finger protein 17 [Source:MGI Symbol;Acc:MGI:1925835] | 12,49133902 | 0,50283486 | **1,41699519** | 3,64788813 | 6,01109611 | 0,014216194 | 0,048168474 | protein_coding |
| ENSMUSG00000031506 | Ptpn7 | protein tyrosine phosphatase, non-receptor type 7 [Source:MGI Symbol;Acc:MGI:2156893] | 113,7490271 | 0,50250218 | **1,41666847** | 6,83106604 | 15,3208308 | 9,07107E-05 | 0,000626217 | protein_coding |
| ENSMUSG00000029364 | Wsb2 | WD repeat and SOCS box-containing 2 [Source:MGI Symbol;Acc:MGI:2144041] | 126,2528001 | 0,50240444 | **1,4165725** | 6,98021403 | 13,5367924 | 0,000233932 | 0,001435871 | protein_coding |
| ENSMUSG00000037366 | Pafah2 | platelet-activating factor acetylhydrolase 2 [Source:MGI Symbol;Acc:MGI:2140321] | 32,54145408 | 0,50211274 | **1,41628611** | 5,0310354 | 9,99324933 | 0,001571151 | 0,007611216 | protein_coding |
| ENSMUSG00000006932 | Ctnnb1 | catenin (cadherin associated protein), beta 1 [Source:MGI Symbol;Acc:MGI:88276] | 362,3910405 | 0,50198041 | **1,41615621** | 8,50177628 | 9,36338422 | 0,00221363 | 0,01022445 | protein_coding |
| ENSMUSG00000000555 | Itga5 | integrin alpha 5 (fibronectin receptor alpha) [Source:MGI Symbol;Acc:MGI:96604] | 226,128083 | 0,50170867 | **1,4158895** | 7,82249074 | 11,772125 | 0,000601243 | 0,003297981 | protein_coding |
| ENSMUSG00000020228 | Helb | helicase (DNA) B [Source:MGI Symbol;Acc:MGI:2152895] | 25,8338932 | 0,50099083 | **1,41518517** | 4,69911079 | 10,9379231 | 0,000942157 | 0,004878172 | protein_coding |
| ENSMUSG00000025340 | Rabgef1 | RAB guanine nucleotide exchange factor (GEF) 1 [Source:MGI Symbol;Acc:MGI:1929459] | 34,68971292 | 0,50065802 | **1,41485874** | 5,12337634 | 11,7926803 | 0,000594641 | 0,003265026 | protein_coding |
| ENSMUSG00000079362 | Gbp6 | guanylate binding protein 6 [Source:MGI Symbol;Acc:MGI:2140937] | 23,21679387 | 0,50061562 | **1,41481716** | 4,54031756 | 8,51395182 | 0,003524339 | 0,015149417 | protein_coding |
| ENSMUSG00000036880 | Acaa2 | acetyl-Coenzyme A acyltransferase 2 (mitochondrial 3-oxoacyl-Coenzyme A thiolase) [Source:MGI Symbol;Acc:MGI:109 | 18,54501219 | 0,50036545 | **1,41457184** | 4,21909507 | 8,91539774 | 0,002827762 | 0,012563131 | protein_coding |
| ENSMUSG00000034610 | Zcchc11 | zinc finger, CCHC domain containing 11 [Source:MGI Symbol;Acc:MGI:2445126] | 22,95785848 | 0,49962878 | **1,41384971** | 4,52164228 | 8,14603507 | 0,004315542 | 0,017905912 | protein_coding |
| ENSMUSG00000058761 | Rnf169 | ring finger protein 169 [Source:MGI Symbol;Acc:MGI:1920257] | 42,2549384 | 0,49842728 | **1,41267273** | 5,40287542 | 11,3195623 | 0,000766948 | 0,004090513 | protein_coding |
| ENSMUSG00000028986 | Klhl7 | kelch-like 7 (Drosophila) [Source:MGI Symbol;Acc:MGI:1196453] | 19,1653827 | 0,49800015 | **1,41225455** | 4,2690627 | 7,18642445 | 0,007345721 | 0,028023392 | protein_coding |
| ENSMUSG00000034377 | Tulp4 | tubby like protein 4 [Source:MGI Symbol;Acc:MGI:1916092] | 187,6095254 | 0,49797146 | **1,41222647** | 7,55208618 | 11,3565073 | 0,000751841 | 0,004017741 | protein_coding |
| ENSMUSG00000040479 | Dgkz | diacylglycerol kinase zeta [Source:MGI Symbol;Acc:MGI:1278339] | 273,8727883 | 0,49552856 | **1,40983718** | 8,09696999 | 10,8410488 | 0,000992745 | 0,005101632 | protein_coding |
| ENSMUSG00000063234 | Gpr84 | G protein-coupled receptor 84 [Source:MGI Symbol;Acc:MGI:1934129] | 28,85994944 | 0,49543807 | **1,40974876** | 4,86465401 | 8,2507477 | 0,004073522 | 0,01707438 | protein_coding |
| ENSMUSG00000046949 | Nqo2 | NAD(P)H dehydrogenase, quinone 2 [Source:MGI Symbol;Acc:MGI:104513] | 31,93779286 | 0,49541959 | **1,4097307** | 5,00314701 | 8,24390369 | 0,00408891 | 0,017120751 | protein_coding |
| ENSMUSG00000027937 | Jtb | jumping translocation breakpoint [Source:MGI Symbol;Acc:MGI:1346082] | 27,01558963 | 0,4944406 | **1,4087744** | 4,75995935 | 11,3350826 | 0,000760564 | 0,004062383 | protein_coding |
| ENSMUSG00000030872 | Gga2 | golgi associated, gamma adaptin ear containing, ARF binding protein 2 [Source:MGI Symbol;Acc:MGI:1921355] | 60,2395573 | 0,49366142 | **1,40801374** | 5,9126519 | 13,0898794 | 0,000296895 | 0,001771858 | protein_coding |
| ENSMUSG00000031429 | Psmd10 | proteasome (prosome, macropain) 26S subunit, non-ATPase, 10 [Source:MGI Symbol;Acc:MGI:1858898] | 38,59660837 | 0,49273693 | **1,40711177** | 5,2790943 | 11,9767312 | 0,00053869 | 0,002994174 | protein_coding |
| ENSMUSG00000051495 | Irf2bp2 | interferon regulatory factor 2 binding protein 2 [Source:MGI Symbol;Acc:MGI:2443921] | 109,129855 | 0,49265603 | **1,40703286** | 6,76746263 | 14,6594894 | 0,000128785 | 0,000852384 | protein_coding |
| ENSMUSG00000041736 | Tspo | translocator protein [Source:MGI Symbol;Acc:MGI:88222] | 88,60250886 | 0,49242391 | **1,40680651** | 6,46797568 | 14,4779802 | 0,000141808 | 0,000927405 | protein_coding |
| ENSMUSG00000020641 | Rsad2 | radical S-adenosyl methionine domain containing 2 [Source:MGI Symbol;Acc:MGI:1929628] | 94,40794985 | 0,49203833 | **1,40643056** | 6,56801097 | 11,369967 | 0,000746412 | 0,003992614 | protein_coding |
| ENSMUSG00000023456 | Tpi1 | triosephosphate isomerase 1 [Source:MGI Symbol;Acc:MGI:98797] | 334,7799177 | 0,49112493 | **1,4055404** | 8,38767708 | 10,6710386 | 0,00108826 | 0,005512546 | protein_coding |
| ENSMUSG00000020132 | Rab21 | RAB21, member RAS oncogene family [Source:MGI Symbol;Acc:MGI:894308] | 53,52369783 | 0,49096848 | **1,405388** | 5,74565274 | 12,5660882 | 0,00039281 | 0,002275067 | protein_coding |
| ENSMUSG00000003541 | Ier3 | immediate early response 3 [Source:MGI Symbol;Acc:MGI:104814] | 35,91717314 | 0,49088564 | **1,4053073** | 5,18095695 | 12,0667535 | 0,00051329 | 0,002869984 | protein_coding |
| ENSMUSG00000024095 | Hnrpll | heterogeneous nuclear ribonucleoprotein L-like [Source:MGI Symbol;Acc:MGI:1919942] | 51,39192361 | 0,49001618 | **1,40446062** | 5,68825568 | 12,0951066 | 0,000505543 | 0,002833879 | protein_coding |
| ENSMUSG00000027342 | Pcna | proliferating cell nuclear antigen [Source:MGI Symbol;Acc:MGI:97503] | 201,436742 | 0,48789848 | **1,40240056** | 7,65497326 | 9,36317257 | 0,002213886 | 0,01022445 | protein_coding |
| ENSMUSG00000034674 | Tdg | thymine DNA glycosylase [Source:MGI Symbol;Acc:MGI:108247] | 31,7720308 | 0,48758241 | **1,40209335** | 4,99240909 | 11,219742 | 0,000809317 | 0,004283223 | protein_coding |
| ENSMUSG00000031431 | Tsc22d3 | TSC22 domain family, member 3 [Source:MGI Symbol;Acc:MGI:1196284] | 41,80883595 | 0,48579933 | **1,40036153** | 5,37712362 | 11,9260719 | 0,000553538 | 0,003062295 | protein_coding |
| ENSMUSG00000029145 | Eif2b4 | eukaryotic translation initiation factor 2B, subunit 4 delta [Source:MGI Symbol;Acc:MGI:95300] | 14,70156649 | 0,48558977 | **1,40015813** | 3,89117919 | 7,36790951 | 0,006639808 | 0,025741556 | protein_coding |
| ENSMUSG00000052751 | Repin1 | replication initiator 1 [Source:MGI Symbol;Acc:MGI:1889817] | 15,85862743 | 0,48476485 | **1,39935776** | 3,9809104 | 7,77829154 | 0,005287778 | 0,021249751 | protein_coding |
| ENSMUSG00000021911 | Parg | poly (ADP-ribose) glycohydrolase [Source:MGI Symbol;Acc:MGI:1347094] | 26,41013497 | 0,48394658 | **1,3985643** | 4,72753684 | 10,6795522 | 0,001083275 | 0,005497431 | protein_coding |
| ENSMUSG00000001418 | 0610031J06Rik | RIKEN cDNA 0610031J06 gene [Source:MGI Symbol;Acc:MGI:1913318] | 187,4202718 | 0,48371716 | **1,39834191** | 7,55052491 | 10,7000723 | 0,001071313 | 0,005439243 | protein_coding |
| ENSMUSG00000028256 | Odf2l | outer dense fiber of sperm tails 2-like [Source:MGI Symbol;Acc:MGI:1098600] | 12,39919166 | 0,48323524 | **1,39787489** | 3,63069124 | 6,18947486 | 0,012851232 | 0,044485344 | protein_coding |
| ENSMUSG00000056666 | Retsat | retinol saturase (all trans retinol 13,14 reductase) [Source:MGI Symbol;Acc:MGI:1914692] | 24,58506989 | 0,482149 | **1,39682279** | 4,62601877 | 8,81830581 | 0,002982234 | 0,013148398 | protein_coding |
| ENSMUSG00000021930 | Spryd7 | SPRY domain containing 7 [Source:MGI Symbol;Acc:MGI:1913924] | 27,81129272 | 0,48115192 | **1,39585774** | 4,79786763 | 8,85295576 | 0,002926147 | 0,012932249 | protein_coding |
| ENSMUSG00000048109 | Rbm15 | RNA binding motif protein 15 [Source:MGI Symbol;Acc:MGI:2443205] | 21,12249654 | 0,48110376 | **1,39581114** | 4,38788077 | 8,81570899 | 0,002986481 | 0,013161842 | protein_coding |
| ENSMUSG00000036553 | Sh3tc1 | SH3 domain and tetratricopeptide repeats 1 [Source:MGI Symbol;Acc:MGI:2678949] | 56,56801663 | 0,480787 | **1,39550471** | 5,8165068 | 12,0221889 | 0,000525709 | 0,002936435 | protein_coding |
| ENSMUSG00000044700 | Tmem201 | transmembrane protein 201 [Source:MGI Symbol;Acc:MGI:1196277] | 13,66412846 | 0,48077401 | **1,39549215** | 3,76584224 | 6,67219339 | 0,00979286 | 0,035757444 | protein_coding |
| ENSMUSG00000027641 | Rbl1 | retinoblastoma-like 1 (p107) [Source:MGI Symbol;Acc:MGI:103300] | 18,85797284 | 0,48007993 | **1,39482094** | 4,2492581 | 8,02317045 | 0,004618265 | 0,018954383 | protein_coding |
| ENSMUSG00000030615 | Tmem126a | transmembrane protein 126A [Source:MGI Symbol;Acc:MGI:1913521] | 22,08449337 | 0,47980863 | **1,39455867** | 4,4701553 | 8,62030763 | 0,003324357 | 0,014425241 | protein_coding |
| ENSMUSG00000016206 | H2-M3 | histocompatibility 2, M region locus 3 [Source:MGI Symbol;Acc:MGI:95915] | 22,34563346 | 0,47910593 | **1,39387958** | 4,48501532 | 8,78260437 | 0,003041167 | 0,013376021 | protein_coding |
| ENSMUSG00000020893 | Per1 | period homolog 1 (Drosophila) [Source:MGI Symbol;Acc:MGI:1098283] | 28,21005882 | 0,47864801 | **1,39343723** | 4,81937231 | 10,3195121 | 0,001316311 | 0,006502836 | protein_coding |
| ENSMUSG00000005907 | Pex1 | peroxisomal biogenesis factor 1 [Source:MGI Symbol;Acc:MGI:1918632] | 17,02870028 | 0,47861225 | **1,39340269** | 4,09486734 | 6,89607544 | 0,008638518 | 0,032118916 | protein_coding |
| ENSMUSG00000035596 | Mboat7 | membrane bound O-acyltransferase domain containing 7 [Source:MGI Symbol;Acc:MGI:1924832] | 37,65911249 | 0,47806365 | **1,39287293** | 5,23738573 | 11,3779629 | 0,000743206 | 0,0039774 | protein_coding |
| ENSMUSG00000030522 | Mtmr10 | myotubularin related protein 10 [Source:MGI Symbol;Acc:MGI:2142292] | 39,98184367 | 0,4779052 | **1,39271997** | 5,32262957 | 10,6243251 | 0,001116097 | 0,005640551 | protein_coding |
| ENSMUSG00000031485 | Prosc | proline synthetase co-transcribed [Source:MGI Symbol;Acc:MGI:1891207] | 45,64150144 | 0,47757872 | **1,39240483** | 5,51717302 | 11,3083519 | 0,000771592 | 0,004111292 | protein_coding |
| ENSMUSG00000014498 | Ankrd52 | ankyrin repeat domain 52 [Source:MGI Symbol;Acc:MGI:2444029] | 103,3517411 | 0,47677106 | **1,39162554** | 6,69262578 | 13,629791 | 0,000222625 | 0,001374917 | protein_coding |
| ENSMUSG00000026790 | Odf2 | outer dense fiber of sperm tails 2 [Source:MGI Symbol;Acc:MGI:1098824] | 36,96628559 | 0,47675045 | **1,39160566** | 5,21132883 | 9,96569948 | 0,001594836 | 0,007715747 | protein_coding |
| ENSMUSG00000026721 | Rabgap1l | RAB GTPase activating protein 1-like [Source:MGI Symbol;Acc:MGI:1352507] | 12,87440146 | 0,47616164 | **1,39103782** | 3,69308939 | 6,18254308 | 0,012901672 | 0,044617774 | protein_coding |
| ENSMUSG00000057101 | Zfp180 | zinc finger protein 180 [Source:MGI Symbol;Acc:MGI:1923701] | 20,57022088 | 0,47608505 | **1,39096397** | 4,36547791 | 9,18826978 | 0,00243571 | 0,011085811 | protein_coding |
| ENSMUSG00000022744 | Cldn25 | claudin 25 [Source:MGI Symbol;Acc:MGI:2447860] | 25,45813278 | 0,47601318 | **1,39089468** | 4,68560175 | 9,37040333 | 0,00220517 | 0,010192765 | protein_coding |
| ENSMUSG00000033400 | Agl | amylo-1,6-glucosidase, 4-alpha-glucanotransferase [Source:MGI Symbol;Acc:MGI:1924809] | 62,49100411 | 0,47554206 | **1,39044055** | 5,96932519 | 10,9183792 | 0,000952149 | 0,00492063 | protein_coding |
| ENSMUSG00000021242 | Npc2 | Niemann Pick type C2 [Source:MGI Symbol;Acc:MGI:1915213] | 480,766569 | 0,47507539 | **1,38999086** | 8,90923569 | 8,05453741 | 0,004538983 | 0,01866385 | protein_coding |
| ENSMUSG00000026177 | Slc11a1 | solute carrier family 11 (proton-coupled divalent metal ion transporters), member 1 [Source:MGI Symbol;Acc:MGI:13452 | 583,4701219 | 0,47504164 | **1,38995834** | 9,18889598 | 7,17849392 | 0,007378262 | 0,028118267 | protein_coding |
| ENSMUSG00000025591 | Tma16 | translation machinery associated 16 homolog (S. cerevisiae) [Source:MGI Symbol;Acc:MGI:1913532] | 34,79154843 | 0,47434621 | **1,38928849** | 5,14271858 | 10,8753772 | 0,000974512 | 0,005024384 | protein_coding |
| ENSMUSG00000035891 | Cerk | ceramide kinase [Source:MGI Symbol;Acc:MGI:2386052] | 131,2273324 | 0,47368262 | **1,38864961** | 7,03527866 | 11,151466 | 0,000839653 | 0,004403469 | protein_coding |
| ENSMUSG00000040433 | Zbtb38 | zinc finger and BTB domain containing 38 [Source:MGI Symbol;Acc:MGI:2442866] | 27,66871911 | 0,47356036 | **1,38853194** | 4,7846211 | 9,42513599 | 0,002140312 | 0,009947382 | protein_coding |
| ENSMUSG00000025157 | Zdhhc16 | zinc finger, DHHC domain containing 16 [Source:MGI Symbol;Acc:MGI:1921418] | 17,58634906 | 0,47333108 | **1,38831128** | 4,15192864 | 8,32627496 | 0,00390757 | 0,016489549 | protein_coding |
| ENSMUSG00000036155 | Mgat5 | mannoside acetylglucosaminyltransferase 5 [Source:MGI Symbol;Acc:MGI:894701] | 36,96824765 | 0,47299904 | **1,38799179** | 5,20963864 | 8,54032278 | 0,00347364 | 0,01498425 | protein_coding |
| ENSMUSG00000025260 | Hsd17b10 | hydroxysteroid (17-beta) dehydrogenase 10 [Source:MGI Symbol;Acc:MGI:1333871] | 29,23797763 | 0,47257061 | **1,38757967** | 4,880307 | 9,56881216 | 0,001979108 | 0,009284672 | protein_coding |
| ENSMUSG00000050619 | Zscan29 | zinc finger SCAN domains 29 [Source:MGI Symbol;Acc:MGI:2139317] | 38,74276508 | 0,47242267 | **1,38743739** | 5,27973263 | 10,3725304 | 0,001279041 | 0,006341528 | protein_coding |
| ENSMUSG00000042472 | Zfp410 | zinc finger protein 410 [Source:MGI Symbol;Acc:MGI:1289280] | 17,58862673 | 0,4718368 | **1,38687408** | 4,14119446 | 8,39327962 | 0,003766108 | 0,016031859 | protein_coding |
| ENSMUSG00000055943 | Emc7 | ER membrane protein complex subunit 7 [Source:MGI Symbol;Acc:MGI:1920274] | 73,88981882 | 0,47047918 | **1,3855696** | 6,21081623 | 12,4140403 | 0,000426118 | 0,002434611 | protein_coding |
| ENSMUSG00000035399 | 3230401D17Rik | RIKEN cDNA 3230401D17 gene [Source:MGI Symbol;Acc:MGI:1913930] | 41,00416072 | 0,4701649 | **1,38526779** | 5,36381869 | 11,0726288 | 0,000876114 | 0,004581562 | protein_coding |
| ENSMUSG00000039005 | Tlr4 | toll-like receptor 4 [Source:MGI Symbol;Acc:MGI:96824] | 65,59820707 | 0,47006598 | **1,38517281** | 6,03843825 | 12,3781948 | 0,000434377 | 0,002475363 | protein_coding |

| | | | | | | | | | | |
|---|---|---|---|---|---|---|---|---|---|---|
| ENSMUSG00000030284 | Creld1 | cysteine-rich with EGF-like domains 1 [Source:MGI Symbol;Acc:MGI:2152539] | 18,95831707 | 0,469964 | **1,38507491** | 4,24461322 | 7,28047412 | 0,006970813 | 0,026826034 | protein_coding |
| ENSMUSG00000034714 | Ttyh2 | tweety homolog 2 (Drosophila) [Source:MGI Symbol;Acc:MGI:2157091] | 55,3660094 | 0,46937393 | **1,38450852** | 5,7916844 | 8,31246661 | 0,003937388 | 0,016587456 | protein_coding |
| ENSMUSG00000025078 | Nhlrc2 | NHL repeat containing 2 [Source:MGI Symbol;Acc:MGI:1914116] | 19,21826805 | 0,4691208 | **1,38426562** | 4,2663228 | 8,51032904 | 0,003531362 | 0,01517367 | protein_coding |
| ENSMUSG00000020716 | Nf1 | neurofibromatosis 1 [Source:MGI Symbol;Acc:MGI:97306] | 63,3236391 | 0,46903063 | **1,38417911** | 5,9865476 | 11,6908682 | 0,000628076 | 0,00342465 | protein_coding |
| ENSMUSG00000069520 | Tmem19 | transmembrane protein 19 [Source:MGI Symbol;Acc:MGI:1914476] | 35,64235535 | 0,46884573 | **1,38400172** | 5,15783821 | 9,57596439 | 0,001971413 | 0,009256372 | protein_coding |
| ENSMUSG00000017144 | Rnd3 | Rho family GTPase 3 [Source:MGI Symbol;Acc:MGI:1921444] | 17,62961551 | 0,46874604 | **1,38390608** | 4,15159827 | 8,10124307 | 0,004423491 | 0,018250431 | protein_coding |
| ENSMUSG00000066440 | Zfyve26 | zinc finger, FYVE domain containing 26 [Source:MGI Symbol;Acc:MGI:1924767] | 59,67495601 | 0,46863079 | **1,38379553** | 5,90091005 | 11,4722416 | 0,000706434 | 0,003797257 | protein_coding |
| ENSMUSG00000056429 | Tgoln1 | trans-golgi network protein [Source:MGI Symbol;Acc:MGI:105080] | 198,1533307 | 0,4684063 | **1,38358022** | 7,63155404 | 11,0727593 | 0,000876052 | 0,004581562 | protein_coding |
| ENSMUSG00000034006 | Pqlc1 | PQ loop repeat containing 1 [Source:MGI Symbol;Acc:MGI:1914193] | 19,09486809 | 0,46822432 | **1,38340572** | 4,25572723 | 7,54956286 | 0,006002463 | 0,023637658 | protein_coding |
| ENSMUSG00000022003 | Slc25a30 | solute carrier family 25, member 30 [Source:MGI Symbol;Acc:MGI:1914804] | 16,89756974 | 0,46789426 | **1,38308925** | 4,07689253 | 6,89102561 | 0,008662955 | 0,032198879 | protein_coding |
| ENSMUSG00000048677 | Tpcn2 | two pore segment channel 2 [Source:MGI Symbol;Acc:MGI:2385297] | 58,46277307 | 0,46788626 | **1,38308158** | 5,8715005 | 10,7865815 | 0,001022385 | 0,005234263 | protein_coding |
| ENSMUSG00000032309 | Fbxo22 | F-box protein 22 [Source:MGI Symbol;Acc:MGI:1926014] | 30,6238093 | 0,46773458 | **1,38293618** | 4,94309918 | 9,49006678 | 0,002065873 | 0,009634018 | protein_coding |
| ENSMUSG00000047648 | Fbxo30 | F-box protein 30 [Source:MGI Symbol;Acc:MGI:1919115] | 25,77800486 | 0,46714407 | **1,38237024** | 4,70141952 | 8,91615019 | 0,002826597 | 0,012563036 | protein_coding |
| ENSMUSG00000047777 | Phf13 | PHD finger protein 13 [Source:MGI Symbol;Acc:MGI:2446217] | 25,43730446 | 0,46625721 | **1,38152073** | 4,67503711 | 9,31167301 | 0,002276985 | 0,010480616 | protein_coding |
| ENSMUSG00000004567 | Mcoln1 | mucolipin 1 [Source:MGI Symbol;Acc:MGI:1890498] | 60,13984873 | 0,46616164 | **1,38142921** | 5,91201305 | 9,79729512 | 0,001747687 | 0,008381424 | protein_coding |
| ENSMUSG00000022797 | Tfrc | transferrin receptor [Source:MGI Symbol;Acc:MGI:98822] | 218,8745826 | 0,46583952 | **1,38112081** | 7,77552931 | 7,42797989 | 0,006421739 | 0,025019734 | protein_coding |
| ENSMUSG00000035671 | Zswim4 | zinc finger, SWIM domain containing 4 [Source:MGI Symbol;Acc:MGI:2443726] | 36,35983414 | 0,46467195 | **1,38000353** | 5,19433389 | 10,7190407 | 0,001060386 | 0,005393734 | protein_coding |
| ENSMUSG00000031007 | Atp6ap2 | ATPase, H+ transporting, lysosomal accessory protein 2 [Source:MGI Symbol;Acc:MGI:1917745] | 248,010176 | 0,46411086 | **1,37946691** | 7,95533892 | 9,55496633 | 0,001994092 | 0,009342894 | protein_coding |
| ENSMUSG00000002870 | Mcm2 | minichromosome maintenance deficient 2 mitotin (S. cerevisiae) [Source:MGI Symbol;Acc:MGI:105380] | 27,0732427 | 0,46403465 | **1,37939405** | 4,76884912 | 7,81030931 | 0,005194901 | 0,020930099 | protein_coding |
| ENSMUSG00000037204 | 9430023L20Rik | RIKEN cDNA 9430023L20 gene [Source:MGI Symbol;Acc:MGI:1915368] | 19,84609736 | 0,46204202 | **1,37749017** | 4,31855373 | 7,1526323 | 0,007485404 | 0,028477195 | protein_coding |
| ENSMUSG00000060260 | Pwwp2b | PWWP domain containing 2B [Source:MGI Symbol;Acc:MGI:2142008] | 24,71019452 | 0,4619875 | **1,37743811** | 4,62518889 | 8,15714846 | 0,004289176 | 0,017830183 | protein_coding |
| ENSMUSG00000026618 | Iars2 | isoleucine-tRNA synthetase 2, mitochondrial [Source:MGI Symbol;Acc:MGI:1919586] | 70,34488031 | 0,46176861 | **1,37722914** | 6,13941406 | 10,9980361 | 0,000912085 | 0,004740339 | protein_coding |
| ENSMUSG00000023048 | Prr13 | proline rich 13 [Source:MGI Symbol;Acc:MGI:1913401] | 218,2161738 | 0,46169465 | **1,37715853** | 7,77025534 | 10,2713575 | 0,001351114 | 0,006659795 | protein_coding |
| ENSMUSG00000032359 | Ctsh | cathepsin H [Source:MGI Symbol;Acc:MGI:107285] | 156,1002931 | 0,46158168 | **1,3770507** | 7,28732298 | 11,5243319 | 0,00068691 | 0,003706825 | protein_coding |
| ENSMUSG00000025289 | Prdx4 | peroxiredoxin 4 [Source:MGI Symbol;Acc:MGI:1859815] | 26,86145082 | 0,46052738 | **1,37604474** | 4,75769675 | 8,38484088 | 0,003783633 | 0,016081539 | protein_coding |
| ENSMUSG00000025877 | Hk3 | hexokinase 3 [Source:MGI Symbol;Acc:MGI:2670962] | 361,8141952 | 0,46038574 | **1,37590965** | 8,49939776 | 6,95696663 | 0,008349347 | 0,031202134 | protein_coding |
| ENSMUSG00000029310 | Nudt9 | nudix (nucleoside diphosphate linked moiety X)-type motif 9 [Source:MGI Symbol;Acc:MGI:1921417] | 27,91466259 | 0,46026811 | **1,37579747** | 4,8084889 | 9,01003436 | 0,002685014 | 0,012007604 | protein_coding |
| ENSMUSG00000032228 | Tcf12 | transcription factor 12 [Source:MGI Symbol;Acc:MGI:101877] | 52,32343337 | 0,45936423 | **1,37493578** | 5,71361678 | 9,05721752 | 0,002616599 | 0,011767732 | protein_coding |
| ENSMUSG00000034430 | Zxdc | ZXD family zinc finger C [Source:MGI Symbol;Acc:MGI:1933108] | 22,80331891 | 0,45858416 | **1,37419254** | 4,51170105 | 7,81401694 | 0,005184254 | 0,020902485 | protein_coding |
| ENSMUSG00000052837 | Junb | Jun-B oncogene [Source:MGI Symbol;Acc:MGI:96647] | 127,6149929 | 0,45714809 | **1,37282534** | 6,99665111 | 10,889466 | 0,000967127 | 0,00498865 | protein_coding |
| ENSMUSG00000027394 | Ttl | tubulin tyrosine ligase [Source:MGI Symbol;Acc:MGI:1916987] | 19,15160845 | 0,45693977 | **1,37262713** | 4,26600221 | 7,15789473 | 0,007463475 | 0,028413442 | protein_coding |
| ENSMUSG00000026307 | Scly | selenocysteine lyase [Source:MGI Symbol;Acc:MGI:1355310] | 32,50363717 | 0,45656453 | **1,37227016** | 5,02462606 | 9,53829908 | 0,002012281 | 0,009416071 | protein_coding |
| ENSMUSG00000035311 | Gnptab | N-acetylglucosamine-1-phosphate transferase, alpha and beta subunits [Source:MGI Symbol;Acc:MGI:3643902] | 83,21556838 | 0,45644234 | **1,37215394** | 6,38164369 | 11,1848227 | 0,000824691 | 0,004348645 | protein_coding |
| ENSMUSG00000054404 | Slfn5 | schlafen 5 [Source:MGI Symbol;Acc:MGI:1329004] | 190,479972 | 0,45608123 | **1,37181053** | 7,57446655 | 9,37819535 | 0,002195816 | 0,010158077 | protein_coding |
| ENSMUSG00000028367 | Txn1 | thioredoxin 1 [Source:MGI Symbol;Acc:MGI:98874] | 163,8573245 | 0,45563719 | **1,37138837** | 7,35917102 | 9,90059251 | 0,001652256 | 0,007965482 | protein_coding |
| ENSMUSG00000027303 | Ptpra | protein tyrosine phosphatase, receptor type, A [Source:MGI Symbol;Acc:MGI:97808] | 117,9847814 | 0,45550372 | **1,3712615** | 6,88295476 | 9,65071469 | 0,001892779 | 0,00892914 | protein_coding |
| ENSMUSG00000041598 | Cdc42ep4 | CDC42 effector protein (Rho GTPase binding) 4 [Source:MGI Symbol;Acc:MGI:1929760] | 18,45153697 | 0,4552326 | **1,37100383** | 4,21391828 | 7,52601169 | 0,006081439 | 0,023897272 | protein_coding |
| ENSMUSG00000005949 | Ctns | cystinosis, nephropathic [Source:MGI Symbol;Acc:MGI:1932872] | 27,60605727 | 0,45519014 | **1,37096348** | 4,79403412 | 9,19215947 | 0,00243054 | 0,011076043 | protein_coding |
| ENSMUSG00000040648 | Ppip5k2 | diphosphoinositol pentakisphosphate kinase 2 [Source:MGI Symbol;Acc:MGI:2142810] | 45,31667627 | 0,45395583 | **1,36979104** | 5,50505891 | 9,72475951 | 0,001818021 | 0,008667252 | protein_coding |
| ENSMUSG00000043858 | Nup62 | nucleoporin 62 [Source:MGI Symbol;Acc:MGI:1351500] | 91,58304106 | 0,45337241 | **1,36923722** | 6,51989901 | 11,9693553 | 0,000540827 | 0,003002557 | protein_coding |
| ENSMUSG00000020451 | Limk2 | LIM motif-containing protein kinase 2 [Source:MGI Symbol;Acc:MGI:1197517] | 18,99008052 | 0,4530051 | **1,36888865** | 4,2513407 | 7,93689194 | 0,004843696 | 0,019652025 | protein_coding |
| ENSMUSG00000026821 | Ralgds | ral guanine nucleotide dissociation stimulator [Source:MGI Symbol;Acc:MGI:107485] | 95,0118052 | 0,45202894 | **1,36796274** | 6,57283332 | 11,9917459 | 0,000534367 | 0,002975718 | protein_coding |
| ENSMUSG00000028164 | Manba | mannosidase, beta A, lysosomal [Source:MGI Symbol;Acc:MGI:88175] | 44,16480283 | 0,4502752 | **1,36630086** | 5,46653961 | 10,8292967 | 0,000999066 | 0,005131714 | protein_coding |
| ENSMUSG00000052144 | Ppp4r2 | protein phosphatase 4, regulatory subunit 2 [Source:MGI Symbol;Acc:MGI:3027896] | 92,03353661 | 0,45001557 | **1,366055** | 6,52764189 | 11,5212477 | 0,000688052 | 0,003711157 | protein_coding |
| ENSMUSG00000028850 | Gpatch3 | G patch domain containing 3 [Source:MGI Symbol;Acc:MGI:2442492] | 16,96371749 | 0,44922897 | **1,36531039** | 4,0782545 | 7,19918408 | 0,007293673 | 0,027884877 | protein_coding |
| ENSMUSG00000018286 | Psmb6 | proteasome (prosome, macropain) subunit, beta type 6 [Source:MGI Symbol;Acc:MGI:104880] | 136,7032211 | 0,44897098 | **1,36506626** | 7,09596087 | 10,8462416 | 0,000989965 | 0,00509421 | protein_coding |
| ENSMUSG00000020232 | Hmg20b | high mobility group 20B [Source:MGI Symbol;Acc:MGI:1341190] | 35,68252503 | 0,44892242 | **1,36502031** | 5,15589259 | 9,67685516 | 0,001866038 | 0,008840946 | protein_coding |
| ENSMUSG00000078517 | Emc1 | ER membrane protein complex subunit 1 [Source:MGI Symbol;Acc:MGI:2443696] | 45,25520983 | 0,44852289 | **1,36464234** | 5,5020497 | 10,4092027 | 0,001253889 | 0,006234776 | protein_coding |
| ENSMUSG00000027905 | Ddx20 | DEAD (Asp-Glu-Ala-Asp) box polypeptide 20 [Source:MGI Symbol;Acc:MGI:1858415] | 18,09371065 | 0,44789355 | **1,36404718** | 4,17934619 | 6,65222213 | 0,009903224 | 0,03611242 | protein_coding |
| ENSMUSG00000038312 | Edem2 | ER degradation enhancer, mannosidase alpha-like 2 [Source:MGI Symbol;Acc:MGI:1915540] | 64,45646506 | 0,44772704 | **1,36388976** | 6,00857581 | 11,3174641 | 0,000767815 | 0,004093151 | protein_coding |
| ENSMUSG00000032306 | Mpi | mannose phosphate isomerase [Source:MGI Symbol;Acc:MGI:97075] | 17,16326102 | 0,44678798 | **1,36300229** | 4,11136602 | 6,3969975 | 0,011431353 | 0,040549959 | protein_coding |
| ENSMUSG00000034807 | Glt25d1 | glycosyltransferase 25 domain containing 1 [Source:MGI Symbol;Acc:MGI:1924348] | 124,9597101 | 0,44660729 | **1,36283159** | 6,96617984 | 11,641453 | 0,000644982 | 0,003508127 | protein_coding |
| ENSMUSG00000032712 | 2810474O19Rik | RIKEN cDNA 2810474O19 gene [Source:MGI Symbol;Acc:MGI:1914496] | 119,6700518 | 0,44614298 | **1,36239305** | 6,90308189 | 11,5795318 | 0,000666817 | 0,003614367 | protein_coding |
| ENSMUSG00000025287 | Acot9 | acyl-CoA thioesterase 9 [Source:MGI Symbol;Acc:MGI:1928939] | 42,91369261 | 0,44538229 | **1,36167489** | 5,42973524 | 9,30846365 | 0,002280977 | 0,010490202 | protein_coding |
| ENSMUSG00000049339 | Fam134a | family with sequence similarity 134, member A [Source:MGI Symbol;Acc:MGI:2388278] | 78,4165541 | 0,44490143 | **1,36122111** | 6,29399649 | 11,5201024 | 0,000688476 | 0,003711622 | protein_coding |
| ENSMUSG00000028136 | Snx27 | sorting nexin family member 27 [Source:MGI Symbol;Acc:MGI:1923992] | 83,47656367 | 0,4446339 | **1,36096871** | 6,38770526 | 10,5518751 | 0,001160703 | 0,005828447 | protein_coding |
| ENSMUSG00000030138 | Bms1 | BMS1 homolog, ribosome assembly protein (yeast) [Source:MGI Symbol;Acc:MGI:2446132] | 65,06730738 | 0,4442773 | **1,36063235** | 6,02549056 | 11,1425737 | 0,000843688 | 0,004422517 | protein_coding |
| ENSMUSG00000038388 | Mpp6 | membrane protein, palmitoylated 6 (MAGUK p55 subfamily member 6) [Source:MGI Symbol;Acc:MGI:1927340] | 45,73447279 | 0,44420357 | **1,36056282** | 5,5200708 | 9,9397814 | 0,001617448 | 0,007818256 | protein_coding |
| ENSMUSG00000038534 | Osbpl7 | oxysterol binding protein-like 7 [Source:MGI Symbol;Acc:MGI:1918490] | 16,04486116 | 0,44405843 | **1,36042595** | 3,98937166 | 6,53458222 | 0,010579707 | 0,038086252 | protein_coding |
| ENSMUSG00000032459 | Mrps22 | mitochondrial ribosomal protein S22 [Source:MGI Symbol;Acc:MGI:1928137] | 15,46640551 | 0,4433875 | **1,35979342** | 3,95849241 | 6,04441831 | 0,013950328 | 0,047423347 | protein_coding |
| ENSMUSG00000048661 | Lemd3 | LEM domain containing 3 [Source:MGI Symbol;Acc:MGI:3580376] | 15,92783254 | 0,44290425 | **1,35933802** | 3,99952021 | 6,20204288 | 0,012760295 | 0,044254218 | protein_coding |
| ENSMUSG00000027613 | Eif6 | eukaryotic translation initiation factor 6 [Source:MGI Symbol;Acc:MGI:1196288] | 81,14618791 | 0,44273611 | **1,3591796** | 6,34233955 | 12,1835347 | 0,000482132 | 0,002719294 | protein_coding |
| ENSMUSG00000097715 | | | 40,6810249 | 0,44267877 | **1,35912559** | 5,3436581 | 7,85079109 | 0,005079847 | 0,020519222 | lincRNA |
| ENSMUSG00000028917 | Plekhm2 | pleckstrin homology domain containing, family M (with RUN domain) member 2 [Source:MGI Symbol;Acc:MGI:1916832] | 88,49966324 | 0,44221165 | **1,3586856** | 6,46688514 | 10,7412474 | 0,001047737 | 0,005346718 | protein_coding |
| ENSMUSG00000027782 | Kpna4 | karyopherin (importin) alpha 4 [Source:MGI Symbol;Acc:MGI:1100848] | 148,0915261 | 0,44157935 | **1,35809024** | 7,21319987 | 9,87777469 | 0,001672871 | 0,008061332 | protein_coding |
| ENSMUSG00000022946 | Dopey2 | dopey family member 2 [Source:MGI Symbol;Acc:MGI:1917278] | 55,55584576 | 0,44125946 | **1,35778915** | 5,79613825 | 9,57880884 | 0,001968361 | 0,009245994 | protein_coding |
| ENSMUSG00000027893 | Ahcyl1 | S-adenosylhomocysteine hydrolase-like 1 [Source:MGI Symbol;Acc:MGI:2385184] | 130,4899453 | 0,44086789 | **1,35742067** | 7,0293376 | 10,4088838 | 0,001254105 | 0,006234776 | protein_coding |
| ENSMUSG00000021814 | Anxa7 | annexin A7 [Source:MGI Symbol;Acc:MGI:88031] | 131,0882264 | 0,4401533 | **1,35674848** | 7,03642058 | 9,02723915 | 0,002659859 | 0,011913523 | protein_coding |
| ENSMUSG00000004931 | Apba3 | amyloid beta (A4) precursor protein-binding, family A, member 3 [Source:MGI Symbol;Acc:MGI:1888527] | 22,6793805 | 0,43946511 | **1,35610145** | 4,49243915 | 6,99778423 | 0,008161067 | 0,030633975 | protein_coding |
| ENSMUSG00000037012 | Hk1 | hexokinase 1 [Source:MGI Symbol;Acc:MGI:96103] | 97,15531616 | 0,43915329 | **1,35580838** | 6,60292369 | 10,2580835 | 0,00136087 | 0,006696503 | protein_coding |
| ENSMUSG00000029270 | Fam69a | family with sequence similarity 69, member A [Source:MGI Symbol;Acc:MGI:1914516] | 25,73232231 | 0,43912701 | **1,35578368** | 4,69372311 | 8,59716041 | 0,003366876 | 0,014575202 | protein_coding |

| | | | | | | | | | | |
|---|---|---|---|---|---|---|---|---|---|---|
| ENSMUSG00000030287 | Itpr2 | inositol 1,4,5-triphosphate receptor 2 [Source:MGI Symbol;Acc:MGI:99418] | 40,6822114 | 0,43905638 | **1,35571731** | 5,34733525 | 9,16754735 | 0,002463445 | 0,011188867 | protein_coding |
| ENSMUSG00000028672 | Hmgcl | 3-hydroxy-3-methylglutaryl-Coenzyme A lyase [Source:MGI Symbol;Acc:MGI:96158] | 40,4349126 | 0,43857794 | **1,35526778** | 5,3376187 | 8,36804329 | 0,003818766 | 0,016180786 | protein_coding |
| ENSMUSG00000058013 | Sept11 | septin 11 [Source:MGI Symbol;Acc:MGI:1277214] | 45,38436098 | 0,43822981 | **1,35494079** | 5,51013048 | 9,41033575 | 0,002157657 | 0,010015285 | protein_coding |
| ENSMUSG00000022180 | Slc7a8 | solute carrier family 7 (cationic amino acid transporter, y+ system), member 8 [Source:MGI Symbol;Acc:MGI:1355323] | 95,24610962 | 0,43781471 | **1,354551** | 6,57697459 | 10,0464386 | 0,001526428 | 0,007424019 | protein_coding |
| ENSMUSG00000018965 | Ywhah | tyrosine 3-monooxygenase/tryptophan 5-monooxygenase activation protein, eta polypeptide [Source:MGI Symbol;Acc:M | 338,2301316 | 0,43757861 | **1,35432934** | 8,402409 | 7,78303217 | 0,00527392 | 0,021209577 | protein_coding |
| ENSMUSG00000006527 | Sfmbt1 | Scm-like with four mbt domains 1 [Source:MGI Symbol;Acc:MGI:1859609] | 81,72985245 | 0,43737419 | **1,35413746** | 6,35575829 | 10,4448899 | 0,001229892 | 0,00613383 | protein_coding |
| ENSMUSG00000028854 | Slc9a1 | solute carrier family 9 (sodium/hydrogen exchanger), member 1 [Source:MGI Symbol;Acc:MGI:102462] | 46,25725096 | 0,43733468 | **1,35410037** | 5,53078031 | 9,14685056 | 0,002491465 | 0,011302118 | protein_coding |
| ENSMUSG00000040964 | Arhgef10l | Rho guanine nucleotide exchange factor (GEF) 10-like [Source:MGI Symbol;Acc:MGI:1920004] | 55,78181469 | 0,43667954 | **1,3534856** | 5,79797927 | 10,5296108 | 0,001174769 | 0,005888318 | protein_coding |
| ENSMUSG00000038563 | Eftud1 | elongation factor Tu GTP binding domain containing 1 [Source:MGI Symbol;Acc:MGI:2141969] | 18,21505189 | 0,4362391 | **1,35307246** | 4,19353678 | 6,87987564 | 0,008717163 | 0,032389404 | protein_coding |
| ENSMUSG00000038564 | Ift172 | intraflagellar transport 172 [Source:MGI Symbol;Acc:MGI:2682064] | 17,28091134 | 0,4359935 | **1,35284214** | 4,1049271 | 6,47187451 | 0,010959488 | 0,039132886 | protein_coding |
| ENSMUSG00000021171 | Esyt2 | extended synaptotagmin-like protein 2 [Source:MGI Symbol;Acc:MGI:1261845] | 45,39927208 | 0,43543562 | **1,3523191** | 5,508386 | 9,03305522 | 0,00265141 | 0,011880522 | protein_coding |
| ENSMUSG00000052962 | Mrpl35 | mitochondrial ribosomal protein L35 [Source:MGI Symbol;Acc:MGI:1913473] | 25,90314954 | 0,43505454 | **1,35196194** | 4,70377915 | 8,35996462 | 0,00383578 | 0,016221601 | protein_coding |
| ENSMUSG00000017615 | Tnfaip1 | tumor necrosis factor, alpha-induced protein 1 (endothelial) [Source:MGI Symbol;Acc:MGI:104961] | 61,02964955 | 0,43497889 | **1,35189105** | 5,9382766 | 9,93282512 | 0,001623572 | 0,007844407 | protein_coding |
| ENSMUSG00000003444 | Med29 | mediator complex subunit 29 [Source:MGI Symbol;Acc:MGI:1914474] | 18,74295128 | 0,43464996 | **1,35158286** | 4,23565017 | 6,99345106 | 0,008180847 | 0,030697734 | protein_coding |
| ENSMUSG00000035765 | Dym | dymeclin [Source:MGI Symbol;Acc:MGI:1918480] | 26,84739998 | 0,43375678 | **1,35074634** | 4,7527117 | 6,90247906 | 0,008607631 | 0,032036599 | protein_coding |
| ENSMUSG00000033020 | Polr2f | polymerase (RNA) II (DNA directed) polypeptide F [Source:MGI Symbol;Acc:MGI:1349393] | 21,06939126 | 0,43327059 | **1,35029122** | 4,39662104 | 7,5861997 | 0,005881681 | 0,023245333 | protein_coding |
| ENSMUSG00000054008 | Ndst1 | N-deacetylase/N-sulfotransferase (heparan glucosaminyl) 1 [Source:MGI Symbol;Acc:MGI:104719] | 29,60418407 | 0,4331788 | **1,35020531** | 4,88494134 | 6,97330561 | 0,008273452 | 0,030992301 | protein_coding |
| ENSMUSG00000006818 | Sod2 | superoxide dismutase 2, mitochondrial [Source:MGI Symbol;Acc:MGI:98352] | 278,956336 | 0,432729 | **1,34978442** | 8,12579297 | 8,72823592 | 0,003133195 | 0,013714906 | protein_coding |
| ENSMUSG00000053870 | Fpgt | fucose-1-phosphate guanylyltransferase [Source:MGI Symbol;Acc:MGI:1922790] | 22,64326564 | 0,43267487 | **1,34973377** | 4,49873751 | 7,55467767 | 0,00598545 | 0,023604502 | protein_coding |
| ENSMUSG00000044763 | Trmt10c | tRNA methyltransferase 10 homolog C (S. cerevisiae) [Source:MGI Symbol;Acc:MGI:1196261] | 22,82052623 | 0,43178733 | **1,34890368** | 4,51356415 | 7,05316008 | 0,007912554 | 0,029884919 | protein_coding |
| ENSMUSG00000027286 | Lrrc57 | leucine rich repeat containing 57 [Source:MGI Symbol;Acc:MGI:1913856] | 16,70525483 | 0,4317038 | **1,34882558** | 4,0659901 | 6,53635472 | 0,01056917 | 0,038060791 | protein_coding |
| ENSMUSG00000035725 | Prkx | protein kinase, X-linked [Source:MGI Symbol;Acc:MGI:1309999] | 56,80565914 | 0,4309253 | **1,34809793** | 5,83480593 | 9,36104473 | 0,002216458 | 0,010231568 | protein_coding |
| ENSMUSG00000030779 | Rbbp6 | retinoblastoma binding protein 6 [Source:MGI Symbol;Acc:MGI:894835] | 91,05655379 | 0,4293318 | **1,34660974** | 6,50860306 | 11,102705 | 0,000862019 | 0,0045143 | protein_coding |
| ENSMUSG00000024236 | Svil | supervillin [Source:MGI Symbol;Acc:MGI:2147319] | 43,56533854 | 0,42894092 | **1,34624494** | 5,44230922 | 9,33746381 | 0,00224516 | 0,010342799 | protein_coding |
| ENSMUSG00000005813 | Metap1 | methionyl aminopeptidase 1 [Source:MGI Symbol;Acc:MGI:1922874] | 57,89117714 | 0,42831234 | **1,34565851** | 5,85990949 | 9,13558282 | 0,002506856 | 0,01134417 | protein_coding |
| ENSMUSG00000058325 | Dock1 | dedicator of cytokinesis 1 [Source:MGI Symbol;Acc:MGI:2429765] | 21,20460828 | 0,42825448 | **1,34560455** | 4,41225044 | 6,25066416 | 0,012414675 | 0,043246681 | protein_coding |
| ENSMUSG00000003847 | Nfat5 | nuclear factor of activated T cells 5 [Source:MGI Symbol;Acc:MGI:1859333] | 137,2700064 | 0,4281094 | **1,34546923** | 7,10169255 | 10,8454773 | 0,000990373 | 0,00509421 | protein_coding |
| ENSMUSG00000024308 | Tapbp | TAP binding protein [Source:MGI Symbol;Acc:MGI:1201689] | 229,7115947 | 0,42729148 | **1,34470665** | 7,84345925 | 8,5192994 | 0,003513997 | 0,015116793 | protein_coding |
| ENSMUSG00000028792 | Ak2 | adenylate kinase 2 [Source:MGI Symbol;Acc:MGI:87978] | 51,41408906 | 0,42665545 | **1,34411395** | 5,68615092 | 9,65118984 | 0,00189229 | 0,00892914 | protein_coding |
| ENSMUSG00000026718 | Stam | signal transducing adaptor molecule (SH3 domain and ITAM motif) 1 [Source:MGI Symbol;Acc:MGI:1329014] | 42,42100892 | 0,42533726 | **1,3428864** | 5,41250336 | 9,14905984 | 0,002488459 | 0,011293143 | protein_coding |
| ENSMUSG00000024451 | Arap3 | ArfGAP with RhoGAP domain, ankyrin repeat and PH domain 3 [Source:MGI Symbol;Acc:MGI:2147274] | 36,73607612 | 0,42352611 | **1,3412016** | 5,20044544 | 6,25963226 | 0,012351984 | 0,043103852 | protein_coding |
| ENSMUSG00000043263 | Pyhin1 | pyrin and HIN domain family, member 1 [Source:MGI Symbol;Acc:MGI:2138243] | 52,79146985 | 0,42311178 | **1,34081648** | 5,72392905 | 9,12420191 | 0,002522499 | 0,011391162 | protein_coding |
| ENSMUSG00000031226 | 2610029G23Rik | RIKEN cDNA 2610029G23 gene [Source:MGI Symbol;Acc:MGI:1914933] | 31,84845052 | 0,42199295 | **1,33977705** | 5,00089539 | 6,37368431 | 0,011582488 | 0,041037341 | protein_coding |
| ENSMUSG00000035021 | Baz1a | bromodomain adjacent to zinc finger domain 1A [Source:MGI Symbol;Acc:MGI:1309478] | 55,07658634 | 0,42191249 | **1,33970234** | 5,78991235 | 9,42081354 | 0,002145363 | 0,009962428 | protein_coding |
| ENSMUSG00000041827 | Oasl1 | 2'-5' oligoadenylate synthetase-like 1 [Source:MGI Symbol;Acc:MGI:2180849] | 23,52197687 | 0,42189464 | **1,33968576** | 4,56110905 | 6,78637381 | 0,009185631 | 0,033843906 | protein_coding |
| ENSMUSG00000032475 | Nck1 | non-catalytic region of tyrosine kinase adaptor protein 1 [Source:MGI Symbol;Acc:MGI:109601] | 27,5816465 | 0,42186505 | **1,33965829** | 4,79367465 | 6,79844025 | 0,009123754 | 0,033672385 | protein_coding |
| ENSMUSG00000049624 | Slc17a5 | solute carrier family 17 (anion/sugar transporter), member 5 [Source:MGI Symbol;Acc:MGI:1924105] | 40,66667541 | 0,42163785 | **1,33944733** | 5,34725725 | 8,24977442 | 0,004075707 | 0,01707438 | protein_coding |
| ENSMUSG00000037536 | Fbxo34 | F-box protein 34 [Source:MGI Symbol;Acc:MGI:1926188] | 26,06390109 | 0,42067562 | **1,33855426** | 4,70466422 | 7,87850719 | 0,005002576 | 0,020227821 | protein_coding |
| ENSMUSG00000041747 | Utp15 | UTP15, U3 small nucleolar ribonucleoprotein, homolog (yeast) [Source:MGI Symbol;Acc:MGI:2145443] | 32,90529581 | 0,42020643 | **1,33811901** | 5,04981871 | 8,21538136 | 0,004153678 | 0,017345673 | protein_coding |
| ENSMUSG00000021583 | Erap1 | endoplasmic reticulum aminopeptidase 1 [Source:MGI Symbol;Acc:MGI:1933403] | 71,84591943 | 0,41881109 | **1,33682544** | 6,16902309 | 8,77029447 | 0,00306176 | 0,013455823 | protein_coding |
| ENSMUSG00000002728 | Naa20 | N(alpha)-acetyltransferase 20, NatB catalytic subunit [Source:MGI Symbol;Acc:MGI:1915127] | 43,85275285 | 0,41847089 | **1,33651024** | 5,45958253 | 8,70875081 | 0,003166861 | 0,013807264 | protein_coding |
| ENSMUSG00000030726 | Pold3 | polymerase (DNA-directed), delta 3, accessory subunit [Source:MGI Symbol;Acc:MGI:1915217] | 34,00614626 | 0,41762403 | **1,33572594** | 5,0955322 | 8,71808405 | 0,00315069 | 0,013769542 | protein_coding |
| ENSMUSG00000022521 | Crebbp | CREB binding protein [Source:MGI Symbol;Acc:MGI:1098280] | 49,21620516 | 0,41755981 | **1,33566649** | 5,62169703 | 9,47816858 | 0,002079314 | 0,009688476 | protein_coding |
| ENSMUSG00000009563 | Tor2a | torsin family 2, member A [Source:MGI Symbol;Acc:MGI:1353596] | 25,89478587 | 0,41650943 | **1,33469438** | 4,68976978 | 7,35090661 | 0,006702895 | 0,025949508 | protein_coding |
| ENSMUSG00000027439 | Gzf1 | GDNF-inducible zinc finger protein 1 [Source:MGI Symbol;Acc:MGI:1921783] | 43,58226556 | 0,41619859 | **1,33440684** | 5,45275928 | 8,57882344 | 0,003400951 | 0,014693765 | protein_coding |
| ENSMUSG00000023008 | Fmnl3 | formin-like 3 [Source:MGI Symbol;Acc:MGI:109569] | 50,84131131 | 0,41605063 | **1,33427** | 5,66649184 | 8,22563126 | 0,004130284 | 0,017274239 | protein_coding |
| ENSMUSG00000060733 | Ipmk | inositol polyphosphate multikinase [Source:MGI Symbol;Acc:MGI:1916968] | 26,99774549 | 0,41596359 | **1,3341895** | 4,75770407 | 8,12660207 | 0,004362042 | 0,018037545 | protein_coding |
| ENSMUSG00000059920 | 4930453N24Rik | RIKEN cDNA 4930453N24 gene [Source:MGI Symbol;Acc:MGI:1914859] | 17,93635983 | 0,41541121 | **1,33367876** | 4,17744902 | 5,9434074 | 0,014772433 | 0,049649654 | protein_coding |
| ENSMUSG00000028684 | Urod | uroporphyrinogen decarboxylase [Source:MGI Symbol;Acc:MGI:98916] | 21,04180384 | 0,41479174 | **1,33310622** | 4,40144275 | 6,63666163 | 0,009990097 | 0,036417118 | protein_coding |
| ENSMUSG00000040721 | Zfhx2 | zinc finger homeobox 2 [Source:MGI Symbol;Acc:MGI:2686934] | 22,36790885 | 0,4145404 | **1,33287399** | 4,48800865 | 6,26460226 | 0,012317382 | 0,04305793 | protein_coding |
| ENSMUSG00000029056 | Pank4 | pantothenate kinase 4 [Source:MGI Symbol;Acc:MGI:2387466] | 24,48987303 | 0,41367486 | **1,33207458** | 4,60778694 | 7,33353695 | 0,006767974 | 0,026173789 | protein_coding |
| ENSMUSG00000030225 | Dera | 2-deoxyribose-5-phosphate aldolase homolog (C. elegans) [Source:MGI Symbol;Acc:MGI:1913762] | 46,62313114 | 0,41365843 | **1,33205941** | 5,54647554 | 8,93968167 | 0,002790413 | 0,012422315 | protein_coding |
| ENSMUSG00000037857 | Nufip2 | nuclear fragile X mental retardation protein interacting protein 2 [Source:MGI Symbol;Acc:MGI:1915814] | 95,19782752 | 0,41346872 | **1,33188427** | 6,57301022 | 10,3884695 | 0,001268047 | 0,006295541 | protein_coding |
| ENSMUSG00000035614 | Fam179b | family with sequence similarity 179, member B [Source:MGI Symbol;Acc:MGI:2684313] | 30,85851848 | 0,412833 | **1,3312975** | 4,95042501 | 6,97083845 | 0,008284867 | 0,031024483 | protein_coding |
| ENSMUSG00000026064 | Ptp4a1 | protein tyrosine phosphatase 4a1 [Source:MGI Symbol;Acc:MGI:1277096] | 114,0216894 | 0,41280909 | **1,33127544** | 6,83514339 | 8,38638967 | 0,003780411 | 0,016080283 | protein_coding |
| ENSMUSG00000055371 | Stam2 | signal transducing adaptor molecule (SH3 domain and ITAM motif) 2 [Source:MGI Symbol;Acc:MGI:1929100] | 68,60285806 | 0,41225063 | **1,3307602** | 6,1032611 | 9,73196794 | 0,001810905 | 0,008646855 | protein_coding |
| ENSMUSG00000024130 | Abca3 | ATP-binding cassette, sub-family A (ABC1), member 3 [Source:MGI Symbol;Acc:MGI:1351617] | 82,8772688 | 0,41188826 | **1,33042599** | 6,37304826 | 9,928675 | 0,001627236 | 0,007858657 | protein_coding |
| ENSMUSG00000045917 | 6330416G13Rik | RIKEN cDNA 6330416G13 gene [Source:MGI Symbol;Acc:MGI:1913920] | 63,87333685 | 0,41147929 | **1,3300489** | 5,99692783 | 9,0098834 | 0,002685235 | 0,012007604 | protein_coding |
| ENSMUSG00000031875 | Cmtm3 | CKLF-like MARVEL transmembrane domain containing 3 [Source:MGI Symbol;Acc:MGI:2447162] | 95,53291563 | 0,41113958 | **1,32973575** | 6,57936822 | 9,85070067 | 0,001697671 | 0,008170088 | protein_coding |
| ENSMUSG00000075706 | Gpx4 | glutathione peroxidase 4 [Source:MGI Symbol;Acc:MGI:104767] | 124,3949442 | 0,41084777 | **1,32946682** | 6,95841156 | 8,58440945 | 0,003390533 | 0,014666059 | protein_coding |
| ENSMUSG00000046718 | Bst2 | bone marrow stromal cell antigen 2 [Source:MGI Symbol;Acc:MGI:1916800] | 319,7991487 | 0,41083804 | **1,32945785** | 8,32130767 | 6,57281147 | 0,010354825 | 0,037448473 | protein_coding |
| ENSMUSG00000032602 | Slc25a20 | solute carrier family 25 (mitochondrial carnitine/acylcarnitine translocase), member 20 [Source:MGI Symbol;Acc:MGI:192 | 27,08263142 | 0,41082811 | **1,3294487** | 4,76529433 | 6,71206279 | 0,009576289 | 0,035083256 | protein_coding |
| ENSMUSG00000029466 | Anapc7 | anaphase promoting complex subunit 7 [Source:MGI Symbol;Acc:MGI:1929711] | 33,50511918 | 0,41081932 | **1,32944061** | 5,06907364 | 8,18813966 | 0,004216514 | 0,017561349 | protein_coding |
| ENSMUSG00000027907 | S100a11 | S100 calcium binding protein A11 (calgizzarin) [Source:MGI Symbol;Acc:MGI:1338798] | 88,2455585 | 0,41072902 | **1,32935739** | 6,46791496 | 8,02370457 | 0,004616904 | 0,018954383 | protein_coding |
| ENSMUSG00000006717 | Acot13 | acyl-CoA thioesterase 13 [Source:MGI Symbol;Acc:MGI:1914084] | 18,1680134 | 0,41046379 | **1,32911302** | 4,18197507 | 5,97075605 | 0,014545043 | 0,049020365 | protein_coding |
| ENSMUSG00000024294 | Mib1 | mindbomb homolog 1 (Drosophila) [Source:MGI Symbol;Acc:MGI:2443157] | 112,6960759 | 0,41023604 | **1,32890322** | 6,81643814 | 9,51080323 | 0,002042657 | 0,009546013 | protein_coding |
| ENSMUSG00000003437 | Paf1 | Paf1, RNA polymerase II associated factor, homolog (S. cerevisiae) [Source:MGI Symbol;Acc:MGI:1923988] | 59,99376223 | 0,40981575 | **1,32851614** | 5,90849152 | 9,12439917 | 0,002522227 | 0,011391162 | protein_coding |
| ENSMUSG00000027994 | Ccdc109b | coiled-coil domain containing 109B [Source:MGI Symbol;Acc:MGI:1914065] | 28,92418082 | 0,40975352 | **1,32845883** | 4,86100202 | 7,66264642 | 0,005637575 | 0,022409565 | protein_coding |
| ENSMUSG00000029635 | Cdk8 | cyclin-dependent kinase 8 [Source:MGI Symbol;Acc:MGI:1196224] | 36,79343478 | 0,40901275 | **1,3277769** | 5,20483446 | 8,72030222 | 0,003146859 | 0,013763749 | protein_coding |
| ENSMUSG00000034312 | Iqsec1 | IQ motif and Sec7 domain 1 [Source:MGI Symbol;Acc:MGI:1196356] | 106,2758227 | 0,40886271 | **1,32763881** | 6,73113926 | 9,13457042 | 0,002508243 | 0,011345439 | protein_coding |
| ENSMUSG00000028437 | Ubap1 | ubiquitin-associated protein 1 [Source:MGI Symbol;Acc:MGI:2149543] | 87,05171591 | 0,40878296 | **1,32756543** | 6,44708689 | 9,97477215 | 0,001586996 | 0,007681202 | protein_coding |

| | | | | | | | | | | |
|---|---|---|---|---|---|---|---|---|---|---|
| ENSMUSG00000023027 | Atf1 | activating transcription factor 1 [Source:MGI Symbol;Acc:MGI:1298366] | 40,18965852 | 0,40746387 | **1,32635215** | 5,33189418 | 8,36504591 | 0,00382507 | 0,016195006 | protein_coding |
| ENSMUSG00000033487 | Fndc3a | fibronectin type III domain containing 3A [Source:MGI Symbol;Acc:MGI:1196463] | 145,1532027 | 0,40735726 | **1,32625414** | 7,18203703 | 8,96461433 | 0,002752588 | 0,012268837 | protein_coding |
| ENSMUSG00000056234 | Ncoa4 | nuclear receptor coactivator 4 [Source:MGI Symbol;Acc:MGI:1350932] | 199,2032805 | 0,4070393 | **1,32596188** | 7,63826775 | 8,52473183 | 0,003503524 | 0,015094242 | protein_coding |
| ENSMUSG00000030983 | Bccip | BRCA2 and CDKN1A interacting protein [Source:MGI Symbol;Acc:MGI:1913415] | 34,83774026 | 0,40699294 | **1,32591927** | 5,12293664 | 8,07510757 | 0,004487744 | 0,018480827 | protein_coding |
| ENSMUSG00000037553 | Zdhhc18 | zinc finger, DHHC domain containing 18 [Source:MGI Symbol;Acc:MGI:3527792] | 42,68612562 | 0,40683507 | **1,32577419** | 5,41807003 | 8,30095193 | 0,00396243 | 0,016673773 | protein_coding |
| ENSMUSG00000024942 | Capn1 | calpain 1 [Source:MGI Symbol;Acc:MGI:88263] | 61,67011335 | 0,40577334 | **1,32479886** | 5,94527627 | 8,70913005 | 0,003166203 | 0,013807264 | protein_coding |
| ENSMUSG00000024480 | Ap3s1 | adaptor-related protein complex 3, sigma 1 subunit [Source:MGI Symbol;Acc:MGI:1337062] | 112,121118 | 0,40572589 | **1,32475529** | 6,81185383 | 9,2545706 | 0,002349084 | 0,010753911 | protein_coding |
| ENSMUSG00000032244 | Fem1b | feminization 1 homolog b (C. elegans) [Source:MGI Symbol;Acc:MGI:1335087] | 95,46874768 | 0,40559506 | **1,32463516** | 6,57904217 | 8,38025641 | 0,003793189 | 0,01611592 | protein_coding |
| ENSMUSG00000041168 | Lonp1 | lon peptidase 1, mitochondrial [Source:MGI Symbol;Acc:MGI:1921392] | 65,1728158 | 0,40523575 | **1,3243053** | 6,02745016 | 9,3107571 | 0,002278123 | 0,010481466 | protein_coding |
| ENSMUSG00000097815 | | | 97,74772994 | 0,40513679 | **1,32421446** | 6,61895555 | 9,49401759 | 0,002061429 | 0,009617377 | lincRNA |
| ENSMUSG00000040455 | Usp45 | ubiquitin specific petidase 45 [Source:MGI Symbol;Acc:MGI:101850] | 31,2621023 | 0,40508298 | **1,32416507** | 4,97191985 | 6,57048822 | 0,01036835 | 0,03748505 | protein_coding |
| ENSMUSG00000027447 | Cst3 | cystatin C [Source:MGI Symbol;Acc:MGI:102519] | 313,4532475 | 0,40495481 | **1,32404744** | 8,29190449 | 7,1537311 | 0,00748082 | 0,028469612 | protein_coding |
| ENSMUSG00000029468 | P2rx7 | purinergic receptor P2X, ligand-gated ion channel, 7 [Source:MGI Symbol;Acc:MGI:1339957] | 47,11345535 | 0,40386835 | **1,3230507** | 5,55728093 | 8,76142688 | 0,003076682 | 0,013510596 | protein_coding |
| ENSMUSG00000041354 | Rgl2 | ral guanine nucleotide dissociation stimulator-like 2 [Source:MGI Symbol;Acc:MGI:107483] | 102,9019476 | 0,40355772 | **1,32276586** | 6,68264569 | 10,2252084 | 0,001385341 | 0,006804086 | protein_coding |
| ENSMUSG00000024885 | Aldh3b1 | aldehyde dehydrogenase 3 family, member B1 [Source:MGI Symbol;Acc:MGI:1914939] | 55,25760258 | 0,40350646 | **1,32271887** | 5,79144889 | 8,65418141 | 0,003263116 | 0,014181907 | protein_coding |
| ENSMUSG00000079437 | Tmem179b | transmembrane protein 179B [Source:MGI Symbol;Acc:MGI:1914956] | 29,07801423 | 0,40291083 | **1,32217288** | 4,8594962 | 7,39740749 | 0,006531795 | 0,025385506 | protein_coding |
| ENSMUSG00000096401 | | Uncharacterized protein [Source:UniProtKB/TrEMBL;Acc:J3QN48] | 33,47376949 | 0,40286608 | **1,32213187** | 5,06605055 | 7,36855233 | 0,006637435 | 0,025741438 | protein_coding |
| ENSMUSG00000037217 | Syn1 | synapsin I [Source:MGI Symbol;Acc:MGI:98460] | 50,25298378 | 0,40259407 | **1,32188262** | 5,65553056 | 8,80645137 | 0,003001673 | 0,013223488 | protein_coding |
| ENSMUSG00000036779 | Papd5 | PAP associated domain containing 5 [Source:MGI Symbol;Acc:MGI:1917820] | 45,77167383 | 0,40242615 | **1,32172877** | 5,52099322 | 8,73299054 | 0,003125035 | 0,013690097 | protein_coding |
| ENSMUSG00000027227 | Sord | sorbitol dehydrogenase [Source:MGI Symbol;Acc:MGI:98266] | 60,96525677 | 0,40164823 | **1,32101627** | 5,93213805 | 6,73883207 | 0,009433637 | 0,034641499 | protein_coding |
| ENSMUSG00000038604 | Fam65a | family with sequence similarity 65, member A [Source:MGI Symbol;Acc:MGI:1922937] | 36,17935964 | 0,4012312 | **1,32063446** | 5,1746562 | 8,31417883 | 0,003933678 | 0,016578184 | protein_coding |
| ENSMUSG00000045216 | Hs6st1 | heparan sulfate 6-O-sulfotransferase 1 [Source:MGI Symbol;Acc:MGI:1354958] | 33,02347123 | 0,40114418 | **1,3205548** | 5,0498537 | 7,70340437 | 0,005511677 | 0,02201265 | protein_coding |
| ENSMUSG00000031652 | N4bp1 | NEDD4 binding protein 1 [Source:MGI Symbol;Acc:MGI:2136825] | 89,03274015 | 0,40062561 | **1,32008023** | 6,48101361 | 9,76852058 | 0,001775252 | 0,008502484 | protein_coding |
| ENSMUSG00000005615 | Pcyt1a | phosphate cytidylyltransferase 1, choline, alpha isoform [Source:MGI Symbol;Acc:MGI:88557] | 119,7253923 | 0,39963711 | **1,31917605** | 6,90313759 | 8,1477303 | 0,004311509 | 0,017902702 | protein_coding |
| ENSMUSG00000024349 | Tmem173 | transmembrane protein 173 [Source:MGI Symbol;Acc:MGI:1919762] | 25,43465823 | 0,39961074 | **1,31915194** | 4,67139309 | 7,13923901 | 0,007541516 | 0,028670807 | protein_coding |
| ENSMUSG00000027194 | Ttc17 | tetratricopeptide repeat domain 17 [Source:MGI Symbol;Acc:MGI:1921819] | 38,67044574 | 0,39934949 | **1,31891308** | 5,27515413 | 8,00577072 | 0,004662851 | 0,019094576 | protein_coding |
| ENSMUSG00000038843 | Gcnt1 | glucosaminyl (N-acetyl) transferase 1, core 2 [Source:MGI Symbol;Acc:MGI:95676] | 57,08931204 | 0,39854559 | **1,31817836** | 5,83190171 | 7,55229579 | 0,005993367 | 0,023618767 | protein_coding |
| ENSMUSG00000030123 | Plxnd1 | plexin D1 [Source:MGI Symbol;Acc:MGI:2154244] | 214,5273076 | 0,39818321 | **1,3178473** | 7,74437594 | 7,30697981 | 0,006868728 | 0,026521996 | protein_coding |
| ENSMUSG00000025178 | Pi4k2a | phosphatidylinositol 4-kinase type 2 alpha [Source:MGI Symbol;Acc:MGI:1934031] | 156,1042966 | 0,3971332 | **1,3168885** | 7,28691086 | 9,19217494 | 0,002430519 | 0,011076043 | protein_coding |
| ENSMUSG00000024953 | Prdx5 | peroxiredoxin 5 [Source:MGI Symbol;Acc:MGI:1859821] | 246,921194 | 0,39651922 | **1,31632818** | 7,94828199 | 7,45744408 | 0,006317464 | 0,024683492 | protein_coding |
| ENSMUSG00000026627 | Tmem206 | transmembrane protein 206 [Source:MGI Symbol;Acc:MGI:1914200] | 38,78735578 | 0,39611829 | **1,31596242** | 5,2796787 | 6,63125764 | 0,010020449 | 0,03649144 | protein_coding |
| ENSMUSG00000061273 | Mmgt1 | membrane magnesium transporter 1 [Source:MGI Symbol;Acc:MGI:2384305] | 35,17902874 | 0,39578768 | **1,31566089** | 5,14339651 | 7,55807428 | 0,00597418 | 0,023576979 | protein_coding |
| ENSMUSG00000045980 | Tmem104 | transmembrane protein 104 [Source:MGI Symbol;Acc:MGI:2444222] | 182,9666887 | 0,39546627 | **1,31536781** | 7,51490707 | 8,20668621 | 0,00417363 | 0,017409141 | protein_coding |
| ENSMUSG00000021504 | B4galt7 | xylosylprotein beta1,4-galactosyltransferase, polypeptide 7 (galactosyltransferase I) [Source:MGI Symbol;Acc:MGI:2384 | 33,28571761 | 0,39531506 | **1,31522995** | 5,05454067 | 7,76069705 | 0,005339535 | 0,021426395 | protein_coding |
| ENSMUSG00000026003 | Acadl | acyl-Coenzyme A dehydrogenase, long-chain [Source:MGI Symbol;Acc:MGI:87866] | 76,74301131 | 0,39499466 | **1,31493789** | 6,26436729 | 7,66282279 | 0,005637024 | 0,022409565 | protein_coding |
| ENSMUSG00000027514 | Zbp1 | Z-DNA binding protein 1 [Source:MGI Symbol;Acc:MGI:1927449] | 46,72385401 | 0,39484892 | **1,31480507** | 5,5539622 | 8,74569579 | 0,003103337 | 0,013616757 | protein_coding |
| ENSMUSG00000032366 | Tpm1 | tropomyosin 1, alpha [Source:MGI Symbol;Acc:MGI:98809] | 146,0605868 | 0,39440306 | **1,31439879** | 7,19004892 | 7,25224059 | 0,00708126 | 0,027174923 | protein_coding |
| ENSMUSG00000069539 | Scyl2 | SCY1-like 2 (S. cerevisiae) [Source:MGI Symbol;Acc:MGI:1289172] | 51,71918092 | 0,39418242 | **1,31419779** | 5,69765751 | 7,6689875 | 0,005617797 | 0,022372734 | protein_coding |
| ENSMUSG00000003154 | Foxj2 | forkhead box J2 [Source:MGI Symbol;Acc:MGI:1926805] | 46,89965555 | 0,39385336 | **1,31389807** | 5,54831047 | 8,28313899 | 0,004001489 | 0,016825245 | protein_coding |
| ENSMUSG00000040268 | Plekha1 | pleckstrin homology domain containing, family A (phosphoinositide binding specific) member 1 [Source:MGI Symbol;Acc: | 41,76953639 | 0,39380479 | **1,31385384** | 5,38549123 | 8,12535375 | 0,004365047 | 0,018040748 | protein_coding |
| ENSMUSG00000015597 | Zfp318 | zinc finger protein 318 [Source:MGI Symbol;Acc:MGI:1889348] | 22,99461124 | 0,39320327 | **1,31330615** | 4,53128999 | 6,21472221 | 0,012669255 | 0,043980017 | protein_coding |
| ENSMUSG00000041438 | Cirh1a | cirrhosis, autosomal recessive 1A (human) [Source:MGI Symbol;Acc:MGI:1096573] | 35,66258672 | 0,39165173 | **1,31189452** | 5,16975546 | 7,35172501 | 0,006699844 | 0,025946841 | protein_coding |
| ENSMUSG00000001150 | Mcm3ap | minichromosome maintenance deficient 3 (S. cerevisiae) associated protein [Source:MGI Symbol;Acc:MGI:1930089] | 53,51282457 | 0,39136963 | **1,31163802** | 5,74535606 | 7,94484934 | 0,004822443 | 0,019601992 | protein_coding |
| ENSMUSG00000036534 | Slc38a7 | solute carrier family 38, member 7 [Source:MGI Symbol;Acc:MGI:2679005] | 84,65518451 | 0,39092531 | **1,31123412** | 6,4028134 | 9,19254779 | 0,002430024 | 0,011076043 | protein_coding |
| ENSMUSG00000041341 | Atg2b | autophagy related 2B [Source:MGI Symbol;Acc:MGI:1923809] | 37,47941271 | 0,39068405 | **1,31101487** | 5,23237658 | 7,59278051 | 0,00586025 | 0,023177308 | protein_coding |
| ENSMUSG00000027555 | Car13 | carbonic anhydrase 13 [Source:MGI Symbol;Acc:MGI:1931322] | 68,67433096 | 0,39064819 | **1,31098229** | 6,10601159 | 8,36154686 | 0,003832442 | 0,016217759 | protein_coding |
| ENSMUSG00000059456 | Ptk2b | PTK2 protein tyrosine kinase 2 beta [Source:MGI Symbol;Acc:MGI:104908] | 223,3327299 | 0,38953686 | **1,3099728** | 7,80312147 | 6,34653026 | 0,011761111 | 0,041562987 | protein_coding |
| ENSMUSG00000073643 | Wdfy1 | WD repeat and FYVE domain containing 1 [Source:MGI Symbol;Acc:MGI:1916618] | 64,09346237 | 0,38946087 | **1,3099038** | 6,00546874 | 8,23320532 | 0,004113082 | 0,017215404 | protein_coding |
| ENSMUSG00000024335 | Brd2 | bromodomain containing 2 [Source:MGI Symbol;Acc:MGI:99495] | 284,7948533 | 0,38921392 | **1,3096796** | 8,1539963 | 7,23990174 | 0,007130088 | 0,027333663 | protein_coding |
| ENSMUSG00000039740 | Alg2 | asparagine-linked glycosylation 2 (alpha-1,3-mannosyltransferase) [Source:MGI Symbol;Acc:MGI:1914731] | 28,68838179 | 0,38839511 | **1,3089365** | 4,84895029 | 6,9113163 | 0,008565191 | 0,031900257 | protein_coding |
| ENSMUSG00000039220 | Ppp1r10 | protein phosphatase 1, regulatory subunit 10 [Source:MGI Symbol;Acc:MGI:1289273] | 60,88432549 | 0,38836844 | **1,3089123** | 5,92984902 | 8,40788924 | 0,003735962 | 0,01594678 | protein_coding |
| ENSMUSG00000040855 | Reps2 | RALBP1 associated Eps domain containing protein 2 [Source:MGI Symbol;Acc:MGI:2663511] | 30,44505076 | 0,38748972 | **1,30811531** | 4,91861296 | 6,31077221 | 0,012000656 | 0,042205892 | protein_coding |
| ENSMUSG00000033581 | Igf2bp2 | insulin-like growth factor 2 mRNA binding protein 2 [Source:MGI Symbol;Acc:MGI:1890358] | 110,0267352 | 0,38729506 | **1,30793882** | 6,78481945 | 8,22205456 | 0,004138432 | 0,017301732 | protein_coding |
| ENSMUSG00000030619 | Eed | embryonic ectoderm development [Source:MGI Symbol;Acc:MGI:95286] | 48,82653927 | 0,38709406 | **1,30775661** | 5,61357445 | 8,47454587 | 0,003601503 | 0,015438826 | protein_coding |
| ENSMUSG00000016619 | Nup50 | nucleoporin 50 [Source:MGI Symbol;Acc:MGI:1351502] | 82,02166954 | 0,38640678 | **1,30713376** | 6,36072378 | 8,49874351 | 0,003553918 | 0,015243739 | protein_coding |
| ENSMUSG00000035666 | Gtf3c4 | general transcription factor IIIC, polypeptide 4 [Source:MGI Symbol;Acc:MGI:2138937] | 39,15082125 | 0,38614115 | **1,30689311** | 5,29315838 | 7,27568316 | 0,00698943 | 0,026888261 | protein_coding |
| ENSMUSG00000007812 | Zfp655 | zinc finger protein 655 [Source:MGI Symbol;Acc:MGI:1919861] | 47,82688347 | 0,38600293 | **1,30676791** | 5,58125398 | 7,77487616 | 0,005297785 | 0,021274401 | protein_coding |
| ENSMUSG00000009112 | Bcl2l13 | BCL2-like 13 (apoptosis facilitator) [Source:MGI Symbol;Acc:MGI:2136959] | 34,06756847 | 0,38491911 | **1,30578657** | 5,09675147 | 6,70340863 | 0,009622877 | 0,035218706 | protein_coding |
| ENSMUSG00000029428 | Stx2 | syntaxin 2 [Source:MGI Symbol;Acc:MGI:108059] | 86,94875375 | 0,38350486 | **1,30450716** | 6,44241943 | 9,11090297 | 0,002540904 | 0,011464852 | protein_coding |
| ENSMUSG00000011960 | Ccnt1 | cyclin T1 [Source:MGI Symbol;Acc:MGI:1328363] | 57,01636304 | 0,38328415 | **1,3043076** | 5,83665918 | 7,72041108 | 0,005459996 | 0,021837997 | protein_coding |
| ENSMUSG00000022971 | Ifnar2 | interferon (alpha and beta) receptor 2 [Source:MGI Symbol;Acc:MGI:1098243] | 78,1233663 | 0,38304164 | **1,30408837** | 6,28887032 | 8,97211871 | 0,002741305 | 0,01222847 | protein_coding |
| ENSMUSG00000032382 | Snx1 | sorting nexin 1 [Source:MGI Symbol;Acc:MGI:1928395] | 188,94255 | 0,38300371 | **1,30405409** | 7,56275453 | 7,82747073 | 0,005145806 | 0,020770377 | protein_coding |
| ENSMUSG00000025981 | Coq10b | coenzyme Q10 homolog B (S. cerevisiae) [Source:MGI Symbol;Acc:MGI:1915126] | 25,57393273 | 0,38298873 | **1,30404054** | 4,68899511 | 6,27138885 | 0,012270293 | 0,042920632 | protein_coding |
| ENSMUSG00000028271 | Gtf2b | general transcription factor IIB [Source:MGI Symbol;Acc:MGI:2385191] | 34,90046133 | 0,38280833 | **1,3038775** | 5,12936614 | 7,11916622 | 0,007626417 | 0,028923521 | protein_coding |
| ENSMUSG00000029333 | Rasgef1b | RasGEF domain family, member 1B [Source:MGI Symbol;Acc:MGI:2443755] | 206,5859501 | 0,38262636 | **1,30371305** | 7,69254797 | 6,93973663 | 0,008430152 | 0,031471996 | protein_coding |
| ENSMUSG00000026339 | Ccdc93 | coiled-coil domain containing 93 [Source:MGI Symbol;Acc:MGI:1918079] | 102,4040948 | 0,38212412 | **1,30325927** | 6,68048748 | 9,06043001 | 0,002612005 | 0,011751885 | protein_coding |
| ENSMUSG00000046873 | Mbtps2 | membrane-bound transcription factor peptidase, site 2 [Source:MGI Symbol;Acc:MGI:2444506] | 36,19522878 | 0,38158798 | **1,30277504** | 5,18052762 | 6,04571231 | 0,013940108 | 0,04740327 | protein_coding |
| ENSMUSG00000038811 | Gngt2 | guanine nucleotide binding protein (G protein), gamma transducing activity polypeptide 2 [Source:MGI Symbol;Acc:MGI: | 38,2873911 | 0,38146706 | **1,30266585** | 5,25282769 | 7,4000252 | 0,006522296 | 0,025357562 | protein_coding |
| ENSMUSG00000032376 | Usp3 | ubiquitin specific peptidase 3 [Source:MGI Symbol;Acc:MGI:2152450] | 36,32097754 | 0,38108676 | **1,3023225** | 5,18870269 | 7,29934992 | 0,006897956 | 0,026601561 | protein_coding |
| ENSMUSG00000024456 | Diap1 | diaphanous homolog 1 (Drosophila) [Source:MGI Symbol;Acc:MGI:1194490] | 201,1164786 | 0,38067621 | **1,30195196** | 7,65255441 | 6,98734855 | 0,008208787 | 0,030792059 | protein_coding |
| ENSMUSG00000034401 | Spata6 | spermatogenesis associated 6 [Source:MGI Symbol;Acc:MGI:1915196] | 27,26453702 | 0,38052587 | **1,30181629** | 4,76941008 | 6,51985231 | 0,010667686 | 0,038352705 | protein_coding |

| | | | | | | | | | | |
|---|---|---|---|---|---|---|---|---|---|---|
| ENSMUSG00000025579 | Gaa | glucosidase, alpha, acid [Source:MGI Symbol;Acc:MGI:95609] | 152,3516213 | 0,38009979 | **1,30143187** | 7,25111334 | 7,53162328 | 0,006062526 | 0,023857081 | protein_coding |
| ENSMUSG00000020230 | Prmt2 | protein arginine N-methyltransferase 2 [Source:MGI Symbol;Acc:MGI:1316652] | 28,82249443 | 0,37953652 | **1,30092386** | 4,85235788 | 6,35517813 | 0,011703919 | 0,041414158 | protein_coding |
| ENSMUSG00000024696 | Lpxn | leupaxin [Source:MGI Symbol;Acc:MGI:2147677] | 122,4229941 | 0,37943189 | **1,30082951** | 6,93606353 | 8,13642308 | 0,004338478 | 0,017987343 | protein_coding |
| ENSMUSG00000006494 | Pdk1 | pyruvate dehydrogenase kinase, isoenzyme 1 [Source:MGI Symbol;Acc:MGI:1926119] | 42,3697947 | 0,37883043 | **1,30028731** | 5,41289579 | 7,43915261 | 0,006381993 | 0,024900199 | protein_coding |
| ENSMUSG00000024104 | D6Wsu116e | DNA segment, Chr 6, Wayne State University 116, expressed [Source:MGI Symbol;Acc:MGI:106463] | 147,623559 | 0,37814233 | **1,29966727** | 7,20690043 | 7,88293191 | 0,004990351 | 0,020187404 | protein_coding |
| ENSMUSG00000025059 | Gyk | glycerol kinase [Source:MGI Symbol;Acc:MGI:106594] | 50,02392045 | 0,37775604 | **1,29931933** | 5,65159012 | 7,87798351 | 0,005004025 | 0,020227821 | protein_coding |
| ENSMUSG00000054199 | Gon4l | gon-4-like (C.elegans) [Source:MGI Symbol;Acc:MGI:1917579] | 44,91386604 | 0,37764631 | **1,29922051** | 5,48946223 | 7,6827592 | 0,005575086 | 0,022241638 | protein_coding |
| ENSMUSG00000006589 | Aprt | adenine phosphoribosyl transferase [Source:MGI Symbol;Acc:MGI:88061] | 115,252553 | 0,37708106 | **1,29871157** | 6,8496723 | 7,65499725 | 0,005661528 | 0,022496639 | protein_coding |
| ENSMUSG00000028330 | Ncbp1 | nuclear cap binding protein subunit 1 [Source:MGI Symbol;Acc:MGI:1891840] | 46,90384852 | 0,37667667 | **1,29834759** | 5,55696465 | 7,33135794 | 0,006776184 | 0,026187103 | protein_coding |
| ENSMUSG00000060538 | Tmem219 | transmembrane protein 219 [Source:MGI Symbol;Acc:MGI:1915992] | 28,31584958 | 0,37499245 | **1,29683276** | 4,81599503 | 6,35321897 | 0,011716851 | 0,041446568 | protein_coding |
| ENSMUSG00000002147 | Stat6 | signal transducer and activator of transcription 6 [Source:MGI Symbol;Acc:MGI:103034] | 246,8831908 | 0,37435416 | **1,29625914** | 7,94778426 | 7,06118192 | 0,007877201 | 0,029782109 | protein_coding |
| ENSMUSG00000017802 | Fam134c | family with sequence similarity 134, member C [Source:MGI Symbol;Acc:MGI:1915248] | 63,83978021 | 0,37343228 | **1,29543109** | 5,99986549 | 7,96547174 | 0,004767806 | 0,019437145 | protein_coding |
| ENSMUSG00000091512 | Lamtor3 | late endosomal/lysosomal adaptor, MAPK and MTOR activator 3 [Source:MGI Symbol;Acc:MGI:1929467] | 75,4724898 | 0,37317861 | **1,29520334** | 6,23879969 | 7,42888889 | 0,006418496 | 0,025015969 | protein_coding |
| ENSMUSG00000066148 | Prpf4 | PRP4 pre-mRNA processing factor 4 homolog (yeast) [Source:MGI Symbol;Acc:MGI:1917302] | 26,31342321 | 0,37184279 | **1,29400464** | 4,72367017 | 6,49484686 | 0,010818761 | 0,038731096 | protein_coding |
| ENSMUSG00000027531 | Impa1 | inositol (myo)-1(or 4)-monophosphatase 1 [Source:MGI Symbol;Acc:MGI:1933158] | 45,11581251 | 0,37168091 | **1,29385945** | 5,4997317 | 7,3426833 | 0,006733625 | 0,026059296 | protein_coding |
| ENSMUSG00000004394 | Tmed4 | transmembrane emp24 protein transport domain containing 4 [Source:MGI Symbol;Acc:MGI:1915070] | 44,80825114 | 0,37162918 | **1,29381306** | 5,49035854 | 7,14264065 | 0,007527224 | 0,028626379 | protein_coding |
| ENSMUSG00000075600 | Zc3h3 | zinc finger CCCH type containing 3 [Source:MGI Symbol;Acc:MGI:2663721] | 25,23158348 | 0,37100465 | **1,2932531** | 4,65629475 | 5,95087202 | 0,014710008 | 0,049470111 | protein_coding |
| ENSMUSG00000015839 | Nfe2l2 | nuclear factor, erythroid derived 2, like 2 [Source:MGI Symbol;Acc:MGI:108420] | 230,5117233 | 0,37098792 | **1,2932381** | 7,8495865 | 6,71224517 | 0,009575309 | 0,035083256 | protein_coding |
| ENSMUSG00000073987 | Ggh | gamma-glutamyl hydrolase [Source:MGI Symbol;Acc:MGI:1329035] | 45,73641826 | 0,37071491 | **1,2929934** | 5,52234791 | 7,29158975 | 0,006927815 | 0,026669901 | protein_coding |
| ENSMUSG00000049643 | 2310022A10Rik | RIKEN cDNA 2310022A10 gene [Source:MGI Symbol;Acc:MGI:1913617] | 45,48402964 | 0,37070988 | **1,29298889** | 5,50636373 | 6,29960627 | 0,012076479 | 0,042421335 | protein_coding |
| ENSMUSG00000022462 | Slc38a2 | solute carrier family 38, member 2 [Source:MGI Symbol;Acc:MGI:1915010] | 128,9600303 | 0,37035912 | **1,29267457** | 7,01099975 | 7,72924782 | 0,005433338 | 0,021747207 | protein_coding |
| ENSMUSG00000002741 | Ykt6 | YKT6 homolog (S. Cerevisiae) [Source:MGI Symbol;Acc:MGI:1927550] | 135,3698946 | 0,37016123 | **1,29249727** | 7,08142244 | 7,93923003 | 0,004837441 | 0,019641921 | protein_coding |
| ENSMUSG00000028779 | Pef1 | penta-EF hand domain containing 1 [Source:MGI Symbol;Acc:MGI:1915148] | 46,21432036 | 0,36963582 | **1,29202665** | 5,53123323 | 7,47404166 | 0,006259487 | 0,02450053 | protein_coding |
| ENSMUSG00000041515 | Irf8 | interferon regulatory factor 8 [Source:MGI Symbol;Acc:MGI:96395] | 142,3512083 | 0,36731243 | **1,28994757** | 7,15299927 | 8,05755121 | 0,004531439 | 0,018639805 | protein_coding |
| ENSMUSG00000025068 | Gsto1 | glutathione S-transferase omega 1 [Source:MGI Symbol;Acc:MGI:1342273] | 71,48207738 | 0,36720749 | **1,28985375** | 6,15990379 | 6,58094168 | 0,010307635 | 0,037335966 | protein_coding |
| ENSMUSG00000039530 | Tusc3 | tumor suppressor candidate 3 [Source:MGI Symbol;Acc:MGI:1933134] | 27,15801933 | 0,36658721 | **1,2892993** | 4,76882868 | 6,14427333 | 0,013183833 | 0,045379315 | protein_coding |
| ENSMUSG00000019943 | Atp2b1 | ATPase, Ca++ transporting, plasma membrane 1 [Source:MGI Symbol;Acc:MGI:104653] | 208,8385256 | 0,36558355 | **1,28840267** | 7,7067896 | 6,9076345 | 0,008582847 | 0,03195518 | protein_coding |
| ENSMUSG00000025234 | Arih1 | ariadne ubiquitin-conjugating enzyme E2 binding protein homolog 1 (Drosophila) [Source:MGI Symbol;Acc:MGI:1344363 | 135,6823513 | 0,36556001 | **1,28838165** | 7,08605845 | 7,1309134 | 0,007576612 | 0,028774365 | protein_coding |
| ENSMUSG00000025510 | Cd151 | CD151 antigen [Source:MGI Symbol;Acc:MGI:1096360] | 52,90176782 | 0,36439479 | **1,28734148** | 5,73020686 | 6,4313708 | 0,011212201 | 0,039918488 | protein_coding |
| ENSMUSG00000048379 | Socs4 | suppressor of cytokine signaling 4 [Source:MGI Symbol;Acc:MGI:1914546] | 41,82384373 | 0,36378424 | **1,28679679** | 5,38524994 | 6,82094707 | 0,009009478 | 0,03335146 | protein_coding |
| ENSMUSG00000001082 | Mfsd10 | major facilitator superfamily domain containing 10 [Source:MGI Symbol;Acc:MGI:1915544] | 36,42895817 | 0,3635423 | **1,28658101** | 5,18514776 | 6,78682909 | 0,009183288 | 0,033843906 | protein_coding |
| ENSMUSG00000022270 | Fam134b | family with sequence similarity 134, member B [Source:MGI Symbol;Acc:MGI:1913520] | 44,66084576 | 0,36269146 | **1,28582246** | 5,48528992 | 7,5179906 | 0,006108578 | 0,023986758 | protein_coding |
| ENSMUSG00000039756 | Dnttip2 | deoxynucleotidyltransferase, terminal, interacting protein 2 [Source:MGI Symbol;Acc:MGI:1923173] | 59,81766718 | 0,36265776 | **1,28579243** | 5,90657746 | 7,23991407 | 0,007130039 | 0,027333663 | protein_coding |
| ENSMUSG00000030720 | Cln3 | ceroid lipofuscinosis, neuronal 3, juvenile (Batten, Spielmeyer-Vogt disease) [Source:MGI Symbol;Acc:MGI:107537] | 101,4190857 | 0,36260177 | **1,28574253** | 6,66461909 | 7,98808187 | 0,004708627 | 0,019239007 | protein_coding |
| ENSMUSG00000057554 | Lgals8 | lectin, galactose binding, soluble 8 [Source:MGI Symbol;Acc:MGI:1928481] | 97,67262183 | 0,36046414 | **1,28383886** | 6,61281179 | 6,71715573 | 0,009548979 | 0,035006552 | protein_coding |
| ENSMUSG00000023068 | Nus1 | nuclear undecaprenyl pyrophosphate synthase 1 homolog (S. cerevisiae) [Source:MGI Symbol;Acc:MGI:1196365] | 109,5198354 | 0,35947834 | **1,28296191** | 6,77683832 | 7,30386374 | 0,006880649 | 0,026551846 | protein_coding |
| ENSMUSG00000039662 | Icmt | isoprenylcysteine carboxyl methyltransferase [Source:MGI Symbol;Acc:MGI:1888594] | 44,39582341 | 0,3589448 | **1,28248753** | 5,47217157 | 6,86929885 | 0,008768905 | 0,032548635 | protein_coding |
| ENSMUSG00000034974 | Dapk3 | death-associated protein kinase 3 [Source:MGI Symbol;Acc:MGI:1203520] | 31,25331264 | 0,35889225 | **1,28244082** | 4,96130831 | 6,32482077 | 0,011905952 | 0,041939947 | protein_coding |
| ENSMUSG00000057738 | Spna2 | spectrin alpha 2 [Source:MGI Symbol;Acc:MGI:98386] | 207,0211487 | 0,35612256 | **1,27998115** | 7,69441231 | 6,4101441 | 0,011347021 | 0,040346186 | protein_coding |
| ENSMUSG00000022551 | Cyc1 | cytochrome c-1 [Source:MGI Symbol;Acc:MGI:1913695] | 82,21982586 | 0,35601948 | **1,2798897** | 6,36309661 | 7,50722358 | 0,006145203 | 0,024104727 | protein_coding |
| ENSMUSG00000050697 | Prkaa1 | protein kinase, AMP-activated, alpha 1 catalytic subunit [Source:MGI Symbol;Acc:MGI:2145955] | 61,43554062 | 0,35520525 | **1,27916755** | 5,94469451 | 6,91200087 | 0,008561913 | 0,031898859 | protein_coding |
| ENSMUSG00000030595 | Nfkbib | nuclear factor of kappa light polypeptide gene enhancer in B cells inhibitor, beta [Source:MGI Symbol;Acc:MGI:104752] | 33,38702057 | 0,35517368 | **1,27913956** | 5,06293427 | 6,24493093 | 0,012454924 | 0,043366638 | protein_coding |
| ENSMUSG00000029501 | Ankle2 | ankyrin repeat and LEM domain containing 2 [Source:MGI Symbol;Acc:MGI:1261856] | 47,42322845 | 0,35459987 | **1,27863091** | 5,56991122 | 6,81517562 | 0,009038641 | 0,033436887 | protein_coding |
| ENSMUSG00000030741 | Spns1 | spinster homolog 1 [Source:MGI Symbol;Acc:MGI:1920908] | 39,82078839 | 0,35458513 | **1,27861785** | 5,31119688 | 6,00022251 | 0,014304074 | 0,048401252 | protein_coding |
| ENSMUSG00000029922 | Mkrn1 | makorin, ring finger protein, 1 [Source:MGI Symbol;Acc:MGI:1859353] | 89,94834702 | 0,35380778 | **1,27792908** | 6,4916554 | 7,23593701 | 0,007145851 | 0,027365445 | protein_coding |
| ENSMUSG00000022193 | Psmb5 | proteasome (prosome, macropain) subunit, beta type 5 [Source:MGI Symbol;Acc:MGI:1194513] | 120,0323976 | 0,35340485 | **1,27757222** | 6,90837242 | 6,80148967 | 0,009108185 | 0,033648832 | protein_coding |
| ENSMUSG00000022974 | Gcfc1 | GC-rich sequence DNA-binding factor 1 [Source:MGI Symbol;Acc:MGI:1914617] | 53,93247088 | 0,3513394 | **1,27574448** | 5,74878525 | 6,9266905 | 0,008491868 | 0,031670115 | protein_coding |
| ENSMUSG00000037434 | Slc30a1 | solute carrier family 30 (zinc transporter), member 1 [Source:MGI Symbol;Acc:MGI:1345281] | 45,35030437 | 0,35036238 | **1,27488082** | 5,50976946 | 6,15874509 | 0,013076395 | 0,045122597 | protein_coding |
| ENSMUSG00000071203 | Naip5 | NLR family, apoptosis inhibitory protein 5 [Source:MGI Symbol;Acc:MGI:1298220] | 38,9383954 | 0,34927884 | **1,27392367** | 5,2793198 | 6,2606595 | 0,012344824 | 0,043103852 | protein_coding |
| ENSMUSG00000078606 | Gm4070 | predicted gene 4070 [Source:MGI Symbol;Acc:MGI:3782245] | 143,3884141 | 0,34786632 | **1,27267701** | 7,16522888 | 6,65809749 | 0,009870624 | 0,036005494 | protein_coding |
| ENSMUSG00000017801 | Mlx | MAX-like protein X [Source:MGI Symbol;Acc:MGI:108398] | 34,7206216 | 0,34782282 | **1,27263863** | 5,12341412 | 5,9870856 | 0,014410993 | 0,048703038 | protein_coding |
| ENSMUSG00000032388 | Spg21 | spastic paraplegia 21 homolog (human) [Source:MGI Symbol;Acc:MGI:106403] | 91,59160569 | 0,34722857 | **1,27211453** | 6,51818292 | 6,84670783 | 0,008880477 | 0,032918285 | protein_coding |
| ENSMUSG00000040761 | Spen | SPEN homolog, transcriptional regulator (Drosophila) [Source:MGI Symbol;Acc:MGI:1891706] | 107,4350492 | 0,34701196 | **1,27192356** | 6,74748101 | 7,67888646 | 0,005587063 | 0,022279228 | protein_coding |
| ENSMUSG00000028156 | Eif4e | eukaryotic translation initiation factor 4E [Source:MGI Symbol;Acc:MGI:95305] | 145,9563582 | 0,34640949 | **1,27139251** | 7,1917008 | 6,51897701 | 0,010672938 | 0,038359034 | protein_coding |
| ENSMUSG00000038780 | Smurf1 | SMAD specific E3 ubiquitin protein ligase 1 [Source:MGI Symbol;Acc:MGI:1923038] | 50,67935465 | 0,34380905 | **1,2691029** | 5,6626703 | 6,59962798 | 0,010200008 | 0,037010398 | protein_coding |
| ENSMUSG00000026626 | Ppp2r5a | protein phosphatase 2, regulatory subunit B (B56), alpha isoform [Source:MGI Symbol;Acc:MGI:2388479] | 81,33861445 | 0,34367509 | **1,26898506** | 6,34736936 | 7,38055487 | 0,006593283 | 0,025588271 | protein_coding |
| ENSMUSG00000024077 | Strn | striatin, calmodulin binding protein [Source:MGI Symbol;Acc:MGI:1333757] | 62,8396145 | 0,34336299 | **1,26871057** | 5,97732707 | 6,35207501 | 0,011724408 | 0,041459953 | protein_coding |
| ENSMUSG00000071054 | Safb | scaffold attachment factor B [Source:MGI Symbol;Acc:MGI:2146974] | 58,523966 | 0,34274498 | **1,26816721** | 5,86860502 | 6,8098044 | 0,00906587 | 0,033526324 | protein_coding |
| ENSMUSG00000013662 | Atad1 | ATPase family, AAA domain containing 1 [Source:MGI Symbol;Acc:MGI:1915229] | 59,66191397 | 0,34256356 | **1,26800775** | 5,90177883 | 6,33780099 | 0,011819132 | 0,041727765 | protein_coding |
| ENSMUSG00000015341 | Golga7 | golgi autoantigen, golgin subfamily a, 7 [Source:MGI Symbol;Acc:MGI:1931029] | 89,00039824 | 0,34233415 | **1,26780613** | 6,47840626 | 7,23771903 | 0,007138761 | 0,027357367 | protein_coding |
| ENSMUSG00000021114 | Atp6v1d | ATPase, H+ transporting, lysosomal V1 subunit D [Source:MGI Symbol;Acc:MGI:1921084] | 120,8613289 | 0,34130086 | **1,26689843** | 6,91864951 | 6,57713473 | 0,010329704 | 0,037386216 | protein_coding |
| ENSMUSG00000042331 | Specc1 | sperm antigen with calponin homology and coiled-coil domains 1 [Source:MGI Symbol;Acc:MGI:2442356] | 137,2559203 | 0,34053898 | **1,26622956** | 7,1008093 | 6,79676124 | 0,009132339 | 0,033692749 | protein_coding |
| ENSMUSG00000029131 | Dnajb6 | DnaJ (Hsp40) homolog, subfamily B, member 6 [Source:MGI Symbol;Acc:MGI:1344381] | 115,9149889 | 0,34047317 | **1,2661718** | 6,85883159 | 6,53820168 | 0,010558203 | 0,038033761 | protein_coding |
| ENSMUSG00000006373 | Pgrmc1 | progesterone receptor membrane component 1 [Source:MGI Symbol;Acc:MGI:1858305] | 56,21641761 | 0,34037992 | **1,26608996** | 5,81588826 | 6,00477045 | 0,01426725 | 0,048291521 | protein_coding |
| ENSMUSG00000021733 | Slc4a7 | solute carrier family 4, sodium bicarbonate cotransporter, member 7 [Source:MGI Symbol;Acc:MGI:2443878] | 105,6158827 | 0,33926333 | **1,26511044** | 6,72609008 | 6,61797147 | 0,010095476 | 0,036643208 | protein_coding |
| ENSMUSG00000037321 | Tap1 | transporter 1, ATP-binding cassette, sub-family B (MDR/TAP) [Source:MGI Symbol;Acc:MGI:98483] | 60,94216083 | 0,33767073 | **1,26371465** | 5,92996126 | 6,40844528 | 0,011357882 | 0,040371741 | protein_coding |
| ENSMUSG00000034042 | Gpbp1l1 | GC-rich promoter binding protein 1-like 1 [Source:MGI Symbol;Acc:MGI:1924360] | 45,69695492 | 0,33672658 | **1,2628879** | 5,51579901 | 6,27260745 | 0,012261857 | 0,042920632 | protein_coding |
| ENSMUSG00000015961 | Adss | adenylosuccinate synthetase, non muscle [Source:MGI Symbol;Acc:MGI:87948] | 76,52695365 | 0,33652738 | **1,26271353** | 6,26072723 | 6,25947338 | 0,012353092 | 0,043103852 | protein_coding |
| ENSMUSG00000004455 | Ppp1cc | protein phosphatase 1, catalytic subunit, gamma isoform [Source:MGI Symbol;Acc:MGI:104872] | 152,9467968 | 0,33559798 | **1,26190034** | 7,25758767 | 6,46344249 | 0,011011611 | 0,039280705 | protein_coding |
| ENSMUSG00000015889 | Lta4h | leukotriene A4 hydrolase [Source:MGI Symbol;Acc:MGI:96836] | 43,8542871 | 0,3350463 | **1,26141789** | 5,45969304 | 6,12067619 | 0,013360968 | 0,045874049 | protein_coding |
| ENSMUSG00000033943 | Mga | MAX gene associated [Source:MGI Symbol;Acc:MGI:1352483] | 98,77767168 | 0,33432669 | **1,26078886** | 6,62636403 | 7,10065614 | 0,007705574 | 0,029193497 | protein_coding |

| | | | | | | | | | | |
|---|---|---|---|---|---|---|---|---|---|---|
| ENSMUSG00000030805 | Stx4a | syntaxin 4A (placental) [Source:MGI Symbol;Acc:MGI:893577] | 102,3879388 | 0,33347461 | **1,26004443** | 6,67891037 | 6,51104331 | 0,010720659 | 0,038429977 | protein_coding |
| ENSMUSG00000006736 | Tspan31 | tetraspanin 31 [Source:MGI Symbol;Acc:MGI:1914375] | 78,09492766 | 0,33237357 | **1,25908316** | 6,28998075 | 6,19363743 | 0,01282104 | 0,044416297 | protein_coding |
| ENSMUSG00000064120 | Mocs1 | molybdenum cofactor synthesis 1 [Source:MGI Symbol;Acc:MGI:1928904] | 43,07010355 | 0,3309906 | **1,25787678** | 5,43170483 | 5,97687465 | 0,014494666 | 0,048940655 | protein_coding |
| ENSMUSG00000022000 | Zc3h13 | zinc finger CCCH type containing 13 [Source:MGI Symbol;Acc:MGI:1914552] | 52,07149181 | 0,33016432 | **1,25715656** | 5,70129652 | 6,1138124 | 0,01341295 | 0,04603814 | protein_coding |
| ENSMUSG00000027889 | Ampd2 | adenosine monophosphate deaminase 2 [Source:MGI Symbol;Acc:MGI:88016] | 58,0478576 | 0,3283635 | **1,25558831** | 5,86125129 | 6,10173255 | 0,013504941 | 0,04625274 | protein_coding |
| ENSMUSG00000022508 | Bcl6 | B cell leukemia/lymphoma 6 [Source:MGI Symbol;Acc:MGI:107187] | 79,58151711 | 0,32823016 | **1,25547227** | 6,31546526 | 6,70461962 | 0,009616344 | 0,035206523 | protein_coding |
| ENSMUSG00000041926 | Rnpep | arginyl aminopeptidase (aminopeptidase B) [Source:MGI Symbol;Acc:MGI:2384902] | 176,6578374 | 0,32705412 | **1,25444926** | 7,46535073 | 6,0926375 | 0,01357463 | 0,046419067 | protein_coding |
| ENSMUSG00000021831 | Ero1l | ERO1-like (S. cerevisiae) [Source:MGI Symbol;Acc:MGI:1354385] | 110,2183924 | 0,32248069 | **1,25047887** | 6,78745524 | 6,28442798 | 0,012180339 | 0,042701142 | protein_coding |
| ENSMUSG00000021109 | Hif1a | hypoxia inducible factor 1, alpha subunit [Source:MGI Symbol;Acc:MGI:106918] | 142,7230366 | 0,32170774 | **1,24980909** | 7,15849866 | 6,16063291 | 0,013062446 | 0,045102796 | protein_coding |
| ENSMUSG00000030452 | Nipa2 | non imprinted in Prader-Willi/Angelman syndrome 2 homolog (human) [Source:MGI Symbol;Acc:MGI:1913918] | 71,16000481 | 0,32169294 | **1,24979627** | 6,15581308 | 6,20987261 | 0,012703977 | 0,044086733 | protein_coding |
| ENSMUSG00000002658 | Gtf2f1 | general transcription factor IIF, polypeptide 1 [Source:MGI Symbol;Acc:MGI:1923848] | 82,85950005 | 0,31325148 | **1,24250484** | 6,37306213 | 6,14622786 | 0,01316927 | 0,04535725 | protein_coding |
| ENSMUSG00000068856 | Sf3b4 | splicing factor 3b, subunit 4 [Source:MGI Symbol;Acc:MGI:109580] | 85,48424384 | 0,31171375 | **1,2411812** | 6,4151332 | 6,05674942 | 0,013853245 | 0,047195534 | protein_coding |
| ENSMUSG00000031545 | Agpat6 | 1-acylglycerol-3-phosphate O-acyltransferase 6 (lysophosphatidic acid acyltransferase, zeta) [Source:MGI Symbol;Acc:M | 81,43941307 | 0,31053587 | **1,24016825** | 6,35032178 | 5,96639005 | 0,014581101 | 0,049111759 | protein_coding |
| ENSMUSG00000043004 | Gng2 | guanine nucleotide binding protein (G protein), gamma 2 [Source:MGI Symbol;Acc:MGI:102705] | 98,92475965 | -0,309336 | **-1,2391373** | 6,6268706 | 6,1661737 | 0,013021595 | 0,04499002 | protein_coding |
| ENSMUSG00000024997 | Prdx3 | peroxiredoxin 3 [Source:MGI Symbol;Acc:MGI:88034] | 88,78955888 | -0,3119027 | **-1,24134377** | 6,47307417 | 6,05754663 | 0,013846992 | 0,047188865 | protein_coding |
| ENSMUSG00000006057 | Atp5g1 | ATP synthase, H+ transporting, mitochondrial F0 complex, subunit c1 (subunit 9) [Source:MGI Symbol;Acc:MGI:107653] | 71,65179268 | -0,314141 | **-1,24327119** | 6,16008923 | 6,02505681 | 0,014104178 | 0,04784273 | protein_coding |
| ENSMUSG00000056941 | Commd7 | COMM domain containing 7 [Source:MGI Symbol;Acc:MGI:1914197] | 74,87045364 | -0,3159119 | **-1,24479821** | 6,224665 | 5,93619062 | 0,014833044 | 0,049838122 | protein_coding |
| ENSMUSG00000074212 | Dnajb14 | DnaJ (Hsp40) homolog, subfamily B, member 14 [Source:MGI Symbol;Acc:MGI:1917854] | 91,62176373 | -0,3167334 | **-1,24550721** | 6,51721847 | 6,10212202 | 0,013501965 | 0,04625274 | protein_coding |
| ENSMUSG00000032481 | Smarcc1 | SWI/SNF related, matrix associated, actin dependent regulator of chromatin, subfamily c, member 1 [Source:MGI Symbo | 94,7268046 | -0,317133 | **-1,24585223** | 6,56719953 | 6,08067562 | 0,013666849 | 0,0466618 | protein_coding |
| ENSMUSG00000040848 | Sft2d2 | SFT2 domain containing 2 [Source:MGI Symbol;Acc:MGI:1917362] | 104,7964734 | -0,3177035 | **-1,24634506** | 6,71020584 | 6,39660893 | 0,011433856 | 0,040549959 | protein_coding |
| ENSMUSG00000027170 | Eif3m | eukaryotic translation initiation factor 3, subunit M [Source:MGI Symbol;Acc:MGI:1351744] | 101,9273491 | -0,319025 | **-1,24748716** | 6,67044551 | 6,0653588 | 0,013785874 | 0,047038945 | protein_coding |
| ENSMUSG00000020580 | Rock2 | Rho-associated coiled-coil containing protein kinase 2 [Source:MGI Symbol;Acc:MGI:107926] | 104,9923663 | -0,319473 | **-1,24787466** | 6,71376454 | 6,51381582 | 0,010703958 | 0,038395162 | protein_coding |
| ENSMUSG00000025224 | Gbf1 | golgi-specific brefeldin A-resistance factor 1 [Source:MGI Symbol;Acc:MGI:1861607] | 89,39334802 | -0,320149 | **-1,24845949** | 6,48221813 | 6,27151649 | 0,012269409 | 0,042920632 | protein_coding |
| ENSMUSG00000020647 | Ncoa1 | nuclear receptor coactivator 1 [Source:MGI Symbol;Acc:MGI:1276523] | 67,36243824 | -0,3209853 | **-1,24918343** | 6,06968716 | 6,1650809 | 0,013029641 | 0,045003669 | protein_coding |
| ENSMUSG00000062901 | Klhl24 | kelch-like 24 (Drosophila) [Source:MGI Symbol;Acc:MGI:1923035] | 102,4889002 | -0,3219333 | **-1,25000448** | 6,67671865 | 6,22706422 | 0,012581215 | 0,04372438 | protein_coding |
| ENSMUSG00000021774 | Ube2e1 | ubiquitin-conjugating enzyme E2E 1 [Source:MGI Symbol;Acc:MGI:107411] | 68,82957941 | -0,3219372 | **-1,25000788** | 6,10400158 | 5,97154221 | 0,01453856 | 0,049013551 | protein_coding |
| ENSMUSG00000018585 | Atox1 | ATX1 (antioxidant protein 1) homolog 1 (yeast) [Source:MGI Symbol;Acc:MGI:1333855] | 83,18615235 | -0,3241634 | **-1,25193824** | 6,37447443 | 6,39229002 | 0,011461707 | 0,040635616 | protein_coding |
| ENSMUSG00000032633 | Flcn | folliculin [Source:MGI Symbol;Acc:MGI:2442184] | 119,9634085 | -0,3243733 | **-1,25212041** | 6,90400791 | 6,19278147 | 0,012827242 | 0,044416297 | protein_coding |
| ENSMUSG00000067194 | Eif1ax | eukaryotic translation initiation factor 1A, X-linked [Source:MGI Symbol;Acc:MGI:1913485] | 116,3322971 | -0,3245477 | **-1,25227174** | 6,86324806 | 6,25831464 | 0,012361174 | 0,043103852 | protein_coding |
| ENSMUSG00000031295 | Phka2 | phosphorylase kinase alpha 2 [Source:MGI Symbol;Acc:MGI:97577] | 91,15027484 | -0,3251566 | **-1,25280038** | 6,50679634 | 6,31506835 | 0,011971613 | 0,042144221 | protein_coding |
| ENSMUSG00000039770 | Ypel5 | yippee-like 5 (Drosophila) [Source:MGI Symbol;Acc:MGI:1916937] | 98,22741051 | -0,3254158 | **-1,25302556** | 6,61690351 | 6,29947726 | 0,012077358 | 0,042421335 | protein_coding |
| ENSMUSG00000034940 | Synrg | synergin, gamma [Source:MGI Symbol;Acc:MGI:1354742] | 69,37779906 | -0,3259147 | **-1,25345889** | 6,11705939 | 6,33187984 | 0,011858655 | 0,041826982 | protein_coding |
| ENSMUSG00000031154 | Otud5 | OTU domain containing 5 [Source:MGI Symbol;Acc:MGI:1859615] | 106,2084952 | -0,3260666 | **-1,25359085** | 6,72955312 | 6,09544201 | 0,013553102 | 0,046388764 | protein_coding |
| ENSMUSG00000042404 | Dennd4b | DENN/MADD domain containing 4B [Source:MGI Symbol;Acc:MGI:2446201] | 120,0541054 | -0,3263687 | **-1,2538534** | 6,90383419 | 6,25323255 | 0,012396687 | 0,043197718 | protein_coding |
| ENSMUSG00000073676 | Hspe1 | heat shock protein 1 (chaperonin 10) [Source:MGI Symbol;Acc:MGI:104680] | 77,6791006 | -0,3274103 | **-1,25475896** | 6,27929015 | 6,29850885 | 0,012083958 | 0,04242761 | protein_coding |
| ENSMUSG00000039997 | Ifi203 | interferon activated gene 203 [Source:MGI Symbol;Acc:MGI:96428] | 63,9589638 | -0,3277384 | **-1,25504442** | 5,99566051 | 6,1985437 | 0,012785547 | 0,044327802 | protein_coding |
| ENSMUSG00000019868 | Vta1 | Vps20-associated 1 homolog (S. cerevisiae) [Source:MGI Symbol;Acc:MGI:1913451] | 51,53045458 | -0,3283893 | **-1,25561073** | 5,68602624 | 5,97948269 | 0,01473247 | 0,048898389 | protein_coding |
| ENSMUSG00000020564 | Atxn7l1 | ataxin 7-like 1 [Source:MGI Symbol;Acc:MGI:3584458] | 54,48650636 | -0,3284429 | **-1,25565745** | 5,76292046 | 6,03185066 | 0,014049995 | 0,047686481 | protein_coding |
| ENSMUSG00000017485 | Top2b | topoisomerase (DNA) II beta [Source:MGI Symbol;Acc:MGI:98791] | 90,98984196 | -0,3294152 | **-1,25650391** | 6,50479944 | 6,36639375 | 0,011630171 | 0,041166459 | protein_coding |
| ENSMUSG00000028790 | Khdrbs1 | KH domain containing, RNA binding, signal transduction associated 1 [Source:MGI Symbol;Acc:MGI:893579] | 139,1955802 | -0,3302819 | **-1,25725904** | 7,12052948 | 6,47946719 | 0,010912769 | 0,038991413 | protein_coding |
| ENSMUSG00000031532 | Tmem66 | transmembrane protein 66 [Source:MGI Symbol;Acc:MGI:1915137] | 149,062394 | -0,3309564 | **-1,25784693** | 7,21973164 | 6,48238194 | 0,010894889 | 0,038952861 | protein_coding |
| ENSMUSG00000056608 | Chd9 | chromodomain helicase DNA binding protein 9 [Source:MGI Symbol;Acc:MGI:1924001] | 77,98896893 | -0,331829 | **-1,25860801** | 6,28230071 | 6,62432038 | 0,010059552 | 0,03656927 | protein_coding |
| ENSMUSG00000030216 | Wbp11 | WW domain binding protein 11 [Source:MGI Symbol;Acc:MGI:1891823] | 151,1286573 | -0,3324485 | **-1,25914856** | 7,23951995 | 6,1552746 | 0,013102077 | 0,045197025 | protein_coding |
| ENSMUSG00000029817 | Tra2a | transformer 2 alpha homolog (Drosophila) [Source:MGI Symbol;Acc:MGI:1933972] | 59,32271743 | -0,3326129 | **-1,25929201** | 5,89196817 | 6,24126325 | 0,012480742 | 0,043421759 | protein_coding |
| ENSMUSG00000033355 | Rtp4 | receptor transporter protein 4 [Source:MGI Symbol;Acc:MGI:1915025] | 129,0770383 | -0,3341927 | **-1,26067174** | 7,01251558 | 6,43455404 | 0,011192125 | 0,039872854 | protein_coding |
| ENSMUSG00000039682 | Lap3 | leucine aminopeptidase 3 [Source:MGI Symbol;Acc:MGI:1914238] | 58,11543512 | -0,3341948 | **-1,26067356** | 5,86401893 | 6,39869156 | 0,01142045 | 0,040549959 | protein_coding |
| ENSMUSG00000036002 | B230312A22Rik | RIKEN cDNA B230312A22 gene [Source:MGI Symbol;Acc:MGI:2441854] | 133,3726464 | -0,3345193 | **-1,26095715** | 7,05761786 | 6,26391151 | 0,012322185 | 0,04306102 | protein_coding |
| ENSMUSG00000025511 | Tspan4 | tetraspanin 4 [Source:MGI Symbol;Acc:MGI:1928097] | 91,02739073 | -0,3348267 | **-1,26122593** | 6,50589521 | 6,72057046 | 0,009530714 | 0,034951252 | protein_coding |
| ENSMUSG00000079557 | 41335 | membrane-associated ring finger (C3HC4) 2 [Source:MGI Symbol;Acc:MGI:1925915] | 49,43751054 | -0,335239 | **-1,26158636** | 5,62452046 | 6,39813448 | 0,011424034 | 0,040549959 | protein_coding |
| ENSMUSG00000006095 | Tbcb | tubulin folding cofactor B [Source:MGI Symbol;Acc:MGI:1913661] | 70,2356665 | -0,3355078 | **-1,26182145** | 6,13198038 | 6,36950976 | 0,011609766 | 0,041107478 | protein_coding |
| ENSMUSG00000068329 | Htra2 | HtrA serine peptidase 2 [Source:MGI Symbol;Acc:MGI:1928676] | 63,69404694 | -0,3355156 | **-1,26182832** | 5,98852854 | 6,00999347 | 0,01422508 | 0,04817847 | protein_coding |
| ENSMUSG00000066357 | Wdr6 | WD repeat domain 6 [Source:MGI Symbol;Acc:MGI:1930140] | 41,82210382 | -0,3364313 | **-1,26262941** | 5,37901415 | 6,06342822 | 0,013800952 | 0,04704656 | protein_coding |
| ENSMUSG00000033272 | Slc35a4 | solute carrier family 35, member A4 [Source:MGI Symbol;Acc:MGI:1915093] | 111,1470762 | -0,3364427 | **-1,26263939** | 6,79415229 | 6,51434475 | 0,010700774 | 0,038395162 | protein_coding |
| ENSMUSG00000033953 | Ppp3r1 | protein phosphatase 3, regulatory subunit B, alpha isoform (calcineurin B, type I) [Source:MGI Symbol;Acc:MGI:107172] | 210,4019168 | -0,3367046 | **-1,26286869** | 7,71669622 | 6,09356528 | 0,013567504 | 0,04641582 | protein_coding |
| ENSMUSG00000029763 | Exoc4 | exocyst complex component 4 [Source:MGI Symbol;Acc:MGI:1096376] | 126,0854183 | -0,3371813 | **-1,26328603** | 6,97816655 | 6,70564367 | 0,009610823 | 0,035198038 | protein_coding |
| ENSMUSG00000019373 | Cops3 | COP9 (constitutive photomorphogenic) homolog, subunit 3 (Arabidopsis thaliana) [Source:MGI Symbol;Acc:MGI:134940 | 50,0307381 | -0,3374718 | **-1,26354038** | 5,64578731 | 5,94347583 | 0,014771859 | 0,049649654 | protein_coding |
| ENSMUSG00000026511 | Srp9 | signal recognition particle 9 [Source:MGI Symbol;Acc:MGI:1350930] | 55,31386875 | -0,3391747 | **-1,26503271** | 5,78680567 | 6,15079678 | 0,013135291 | 0,045283164 | protein_coding |
| ENSMUSG00000031221 | Igbp1 | immunoglobulin (CD79A) binding protein 1 [Source:MGI Symbol;Acc:MGI:1346500] | 54,81332951 | -0,3392547 | **-1,26510289** | 5,77283509 | 6,27496812 | 0,012245532 | 0,042902316 | protein_coding |
| ENSMUSG00000034998 | Foxn2 | forkhead box N2 [Source:MGI Symbol;Acc:MGI:1347478] | 61,86579316 | -0,3392579 | **-1,26510567** | 5,9506063 | 6,51441632 | 0,010700344 | 0,038395162 | protein_coding |
| ENSMUSG00000026519 | Tmem63a | transmembrane protein 63a [Source:MGI Symbol;Acc:MGI:2384789] | 50,93445061 | -0,3393282 | **-1,2651673** | 5,66964423 | 6,33008506 | 0,011870662 | 0,041835925 | protein_coding |
| ENSMUSG00000047888 | Tnrc6b | trinucleotide repeat containing 6b [Source:MGI Symbol;Acc:MGI:2443730] | 58,09928723 | -0,3399287 | **-1,26569408** | 5,85384369 | 6,46367578 | 0,011010166 | 0,039280705 | protein_coding |
| ENSMUSG00000026623 | Lpgat1 | lysophosphatidylglycerol acyltransferase 1 [Source:MGI Symbol;Acc:MGI:2446186] | 96,54838129 | -0,3399769 | **-1,26573632** | 6,59217493 | 6,96277173 | 0,008322301 | 0,031122233 | protein_coding |
| ENSMUSG00000025142 | Aspscr1 | alveolar soft part sarcoma chromosome region, candidate 1 (human) [Source:MGI Symbol;Acc:MGI:1916188] | 52,97519173 | -0,3401001 | **-1,26584443** | 5,7237879 | 6,06408217 | 0,013795843 | 0,047043739 | protein_coding |
| ENSMUSG00000024150 | Mcfd2 | multiple coagulation factor deficiency 2 [Source:MGI Symbol;Acc:MGI:2183439] | 133,3503812 | -0,3410923 | **-1,26671528** | 7,05694246 | 6,67841743 | 0,009758723 | 0,035668359 | protein_coding |
| ENSMUSG00000035392 | Dennd1a | DENN/MADD domain containing 1A [Source:MGI Symbol;Acc:MGI:2442794] | 131,104004 | -0,3415285 | **-1,26709838** | 7,03282073 | 6,09058457 | 0,013590412 | 0,046444122 | protein_coding |
| ENSMUSG00000028759 | Hp1bp3 | heterochromatin protein 1, binding protein 3 [Source:MGI Symbol;Acc:MGI:109369] | 139,4336355 | -0,3419736 | **-1,26748932** | 7,12209359 | 6,97516017 | 0,008264882 | 0,030970758 | protein_coding |
| ENSMUSG00000028439 | 2310028H24Rik | RIKEN cDNA 2310028H24 gene [Source:MGI Symbol;Acc:MGI:1919151] | 85,95514849 | -0,3432872 | **-1,26864396** | 6,42287011 | 7,2607918 | 0,007047621 | 0,027093147 | protein_coding |
| ENSMUSG00000043157 | Arl11 | ADP-ribosylation factor-like 11 [Source:MGI Symbol;Acc:MGI:2444054] | 67,47082634 | -0,3433533 | **-1,26870204** | 6,06949749 | 6,41810527 | 0,011296262 | 0,040178709 | protein_coding |
| ENSMUSG00000024072 | Yipf4 | Yip1 domain family, member 4 [Source:MGI Symbol;Acc:MGI:1915114] | 70,31728416 | -0,3435112 | **-1,26884089** | 6,13604272 | 7,12338904 | 0,007608475 | 0,028865439 | protein_coding |
| ENSMUSG00000040359 | Ufl1 | UFM1 specific ligase 1 [Source:MGI Symbol;Acc:MGI:1914740] | 41,31063167 | -0,3441013 | **-1,26936004** | 5,36376146 | 6,10719127 | 0,013463292 | 0,046182075 | protein_coding |
| ENSMUSG00000003134 | Tbc1d8 | TBC1 domain family, member 8 [Source:MGI Symbol;Acc:MGI:1927225] | 42,17373785 | -0,3442382 | **-1,26948043** | 5,39376696 | 6,14597715 | 0,013171137 | 0,04535725 | protein_coding |

| | | | | | | | | | | |
|---|---|---|---|---|---|---|---|---|---|---|
| ENSMUSG00000020257 | Wdr82 | WD repeat domain containing 82 [Source:MGI Symbol;Acc:MGI:1924555] | 61,85981302 | -0,344545 | **-1,26975048** | 5,94930103 | 6,223026 | 0,012609941 | 0,043801905 | protein_coding |
| ENSMUSG00000036323 | Srp72 | signal recognition particle 72 [Source:MGI Symbol;Acc:MGI:1333795] | 184,1442561 | -0,3448715 | **-1,27003784** | 7,52509078 | 6,62582741 | 0,010051044 | 0,036566497 | protein_coding |
| ENSMUSG00000003345 | Csnk1g2 | casein kinase 1, gamma 2 [Source:MGI Symbol;Acc:MGI:1920014] | 67,25995996 | -0,3450432 | **-1,27018905** | 6,06443015 | 5,96068767 | 0,014628334 | 0,04925575 | protein_coding |
| ENSMUSG00000026767 | Fam188a | family with sequence similarity 188, member A [Source:MGI Symbol;Acc:MGI:1914210] | 38,20015564 | -0,3452099 | **-1,2703358** | 5,25996787 | 6,13083649 | 0,0132844 | 0,045639683 | protein_coding |
| ENSMUSG00000044783 | Hjurp | Holliday junction recognition protein [Source:MGI Symbol;Acc:MGI:2685821] | 55,70875188 | -0,3455632 | **-1,2706469** | 5,8022167 | 6,51170247 | 0,010716686 | 0,038428273 | protein_coding |
| ENSMUSG00000031662 | Snx20 | sorting nexin 20 [Source:MGI Symbol;Acc:MGI:1918857] | 94,47942782 | -0,34568 | **-1,27074978** | 6,55973449 | 6,47974462 | 0,010911066 | 0,038991413 | protein_coding |
| ENSMUSG00000071796 | 6820431F20Rik | RIKEN cDNA 6820431F20 gene [Source:MGI Symbol;Acc:MGI:3694236] | 55,73565802 | -0,3462228 | **-1,27122803** | 5,79757411 | 5,99087489 | 0,014380068 | 0,048628443 | pseudogene |
| ENSMUSG00000029538 | Srsf9 | serine/arginine-rich splicing factor 9 [Source:MGI Symbol;Acc:MGI:104896] | 52,70147322 | -0,3472599 | **-1,27214219** | 5,7156322 | 5,97408366 | 0,014517623 | 0,048977478 | protein_coding |
| ENSMUSG00000028811 | Yars | tyrosyl-tRNA synthetase [Source:MGI Symbol;Acc:MGI:2147627] | 65,25795919 | -0,3477282 | **-1,27255516** | 6,02403073 | 6,67151111 | 0,009796609 | 0,035759251 | protein_coding |
| ENSMUSG00000020271 | Fbxw11 | F-box and WD-40 domain protein 11 [Source:MGI Symbol;Acc:MGI:2144023] | 168,8678672 | -0,3480129 | **-1,27280634** | 7,40018759 | 6,6210509 | 0,010078035 | 0,036604089 | protein_coding |
| ENSMUSG00000024966 | Stip1 | stress-induced phosphoprotein 1 [Source:MGI Symbol;Acc:MGI:109130] | 126,7269216 | -0,3484032 | **-1,27315072** | 6,98583763 | 6,68113273 | 0,009743869 | 0,035625918 | protein_coding |
| ENSMUSG00000038116 | Phf20 | PHD finger protein 20 [Source:MGI Symbol;Acc:MGI:2444148] | 110,2616774 | -0,3487933 | **-1,27349501** | 6,78424715 | 7,46794305 | 0,006280727 | 0,02457491 | protein_coding |
| ENSMUSG00000029681 | Bcl7b | B cell CLL/lymphoma 7B [Source:MGI Symbol;Acc:MGI:1332238] | 50,18268526 | -0,3489098 | **-1,27359784** | 5,64369819 | 6,04192546 | 0,01397004 | 0,047460985 | protein_coding |
| ENSMUSG00000042613 | Pbxip1 | pre B cell leukemia transcription factor interacting protein 1 [Source:MGI Symbol;Acc:MGI:2441670] | 71,71935629 | -0,3489324 | **-1,27361784** | 6,15933229 | 6,55369409 | 0,010466663 | 0,037741131 | protein_coding |
| ENSMUSG00000050627 | Gpd1l | glycerol-3-phosphate dehydrogenase 1-like [Source:MGI Symbol;Acc:MGI:1289257] | 38,82623615 | -0,3497129 | **-1,27430701** | 5,27207588 | 6,05458552 | 0,013870231 | 0,047238755 | protein_coding |
| ENSMUSG00000020802 | Ube2o | ubiquitin-conjugating enzyme E2O [Source:MGI Symbol;Acc:MGI:2444266] | 38,66752994 | -0,349914 | **-1,27448461** | 5,27529419 | 6,0915197 | 0,013583221 | 0,04643399 | protein_coding |
| ENSMUSG00000034189 | Hsdl1 | hydroxysteroid dehydrogenase like 1 [Source:MGI Symbol;Acc:MGI:1919802] | 72,85638772 | -0,349919 | **-1,27448904** | 6,18361784 | 7,10539952 | 0,007685209 | 0,029126387 | protein_coding |
| ENSMUSG00000022205 | Sub1 | SUB1 homolog (S. cerevisiae) [Source:MGI Symbol;Acc:MGI:104811] | 70,62652206 | -0,3501143 | **-1,27466164** | 6,14006883 | 6,68631949 | 0,009715558 | 0,035546067 | protein_coding |
| ENSMUSG00000022973 | Synj1 | synaptojanin 1 [Source:MGI Symbol;Acc:MGI:1354961] | 222,7059356 | -0,3504086 | **-1,27492166** | 7,79878218 | 6,26965511 | 0,012282305 | 0,042948976 | protein_coding |
| ENSMUSG00000004591 | Pkn2 | protein kinase N2 [Source:MGI Symbol;Acc:MGI:109211] | 53,45502296 | -0,3505793 | **-1,27507252** | 5,74020998 | 6,87114061 | 0,008759872 | 0,032526096 | protein_coding |
| ENSMUSG00000022450 | Ndufa6 | NADH dehydrogenase (ubiquinone) 1 alpha subcomplex, 6 (B14) [Source:MGI Symbol;Acc:MGI:1914380] | 42,72929463 | -0,3507862 | **-1,27525542** | 5,41221359 | 6,34877416 | 0,011746244 | 0,041523803 | protein_coding |
| ENSMUSG00000040242 | Fgfr1op2 | FGFR1 oncogene partner 2 [Source:MGI Symbol;Acc:MGI:1914779] | 93,92543098 | -0,3510728 | **-1,27550879** | 6,55187559 | 7,19281365 | 0,007319612 | 0,027962648 | protein_coding |
| ENSMUSG00000049550 | Clip1 | CAP-GLY domain containing linker protein 1 [Source:MGI Symbol;Acc:MGI:1928401] | 97,38444579 | -0,3513097 | **-1,27571824** | 6,60498899 | 7,58108722 | 0,005898385 | 0,023294594 | protein_coding |
| ENSMUSG00000002458 | Rgs19 | regulator of G-protein signaling 19 [Source:MGI Symbol;Acc:MGI:1915153] | 86,60956461 | -0,3516951 | **-1,27605902** | 6,43317577 | 7,21708323 | 0,007221295 | 0,027644729 | protein_coding |
| ENSMUSG00000031833 | Mast3 | microtubule associated serine/threonine kinase 3 [Source:MGI Symbol;Acc:MGI:2683541] | 114,3644752 | -0,3517596 | **-1,27611611** | 6,8338933 | 6,95206508 | 0,008372253 | 0,031266468 | protein_coding |
| ENSMUSG00000026755 | Arpc5l | actin related protein 2/3 complex, subunit 5-like [Source:MGI Symbol;Acc:MGI:1921442] | 46,87190239 | -0,3519853 | **-1,27631577** | 5,54628069 | 6,17265621 | 0,012973967 | 0,044853674 | protein_coding |
| ENSMUSG00000028420 | Tmem38b | transmembrane protein 38B [Source:MGI Symbol;Acc:MGI:1098718] | 121,3733793 | -0,3531873 | **-1,2773796** | 6,92405306 | 7,0254479 | 0,008035937 | 0,030246947 | protein_coding |
| ENSMUSG00000060261 | Gtf2i | general transcription factor II I [Source:MGI Symbol;Acc:MGI:1202722] | 172,873719 | -0,3532914 | **-1,27747179** | 7,43210984 | 7,19381205 | 0,00731554 | 0,027956815 | protein_coding |
| ENSMUSG00000034786 | Gpsm3 | G-protein signalling modulator 3 (AGS3-like, C. elegans) [Source:MGI Symbol;Acc:MGI:2146785] | 101,8752825 | -0,3547569 | **-1,27877005** | 6,66772352 | 7,292769 | 0,006923269 | 0,026668976 | protein_coding |
| ENSMUSG00000031907 | Zfp90 | zinc finger protein 90 [Source:MGI Symbol;Acc:MGI:104786] | 26,48657463 | -0,3551048 | **-1,27907849** | 4,72244159 | 5,9735838 | 0,014521739 | 0,048977478 | protein_coding |
| ENSMUSG00000031232 | Magt1 | magnesium transporter 1 [Source:MGI Symbol;Acc:MGI:1914325] | 88,13599916 | -0,3563889 | **-1,28021748** | 6,46064142 | 7,45862206 | 0,006313332 | 0,02467612 | protein_coding |
| ENSMUSG00000039263 | Npepl1 | aminopeptidase-like 1 [Source:MGI Symbol;Acc:MGI:2448523] | 82,95183201 | -0,3580288 | **-1,28167353** | 6,36788166 | 6,92366049 | 0,008506268 | 0,031713053 | protein_coding |
| ENSMUSG00000029674 | Limk1 | LIM-domain containing, protein kinase [Source:MGI Symbol;Acc:MGI:104572] | 36,14069684 | -0,3583272 | **-1,28193863** | 5,16848363 | 6,5765232 | 0,010333254 | 0,037386216 | protein_coding |
| ENSMUSG00000019843 | Fyn | Fyn proto-oncogene [Source:MGI Symbol;Acc:MGI:95602] | 66,98317278 | -0,3588913 | **-1,28243995** | 6,06410854 | 7,20860862 | 0,007255472 | 0,027756222 | protein_coding |
| ENSMUSG00000024687 | Osbp | oxysterol binding protein [Source:MGI Symbol;Acc:MGI:97447] | 70,46325217 | -0,3590486 | **-1,28257977** | 6,13794723 | 7,00370125 | 0,008134136 | 0,03054332 | protein_coding |
| ENSMUSG00000042570 | Mier2 | mesoderm induction early response 1, family member 2 [Source:MGI Symbol;Acc:MGI:1917677] | 39,69629776 | -0,3593221 | **-1,28282301** | 5,30946557 | 6,05198057 | 0,013890707 | 0,047293834 | protein_coding |
| ENSMUSG00000014418 | Hps5 | Hermansky-Pudlak syndrome 5 homolog (human) [Source:MGI Symbol;Acc:MGI:2180307] | 71,17609144 | -0,3593245 | **-1,28282508** | 6,15290017 | 7,66672939 | 0,005624832 | 0,022383206 | protein_coding |
| ENSMUSG00000022237 | Ankrd33b | ankyrin repeat domain 33B [Source:MGI Symbol;Acc:MGI:1917904] | 70,79159152 | -0,3594793 | **-1,28296279** | 6,14846059 | 7,58817097 | 0,005875253 | 0,023228284 | protein_coding |
| ENSMUSG00000028101 | Pias3 | protein inhibitor of activated STAT 3 [Source:MGI Symbol;Acc:MGI:1913126] | 34,40882334 | -0,3601097 | **-1,28352346** | 5,09490229 | 6,10589199 | 0,013473193 | 0,046187199 | protein_coding |
| ENSMUSG00000020700 | Map3k3 | mitogen-activated protein kinase kinase kinase 3 [Source:MGI Symbol;Acc:MGI:1346874] | 110,9055815 | -0,3607277 | **-1,28407339** | 6,79213783 | 7,19905986 | 0,007294178 | 0,027884877 | protein_coding |
| ENSMUSG00000009995 | Taz | tafazzin [Source:MGI Symbol;Acc:MGI:109626] | 67,34267814 | -0,3612546 | **-1,2845425** | 6,06928256 | 6,62288516 | 0,010067661 | 0,036578504 | protein_coding |
| ENSMUSG00000042726 | Trafd1 | TRAF type zinc finger domain containing 1 [Source:MGI Symbol;Acc:MGI:1923551] | 170,7113646 | -0,3619324 | **-1,28514611** | 7,41422977 | 7,29228173 | 0,006925147 | 0,026668976 | protein_coding |
| ENSMUSG00000021972 | Hmbox1 | homeobox containing 1 [Source:MGI Symbol;Acc:MGI:2445066] | 71,58039541 | -0,3629296 | **-1,28603469** | 6,15781354 | 7,45174168 | 0,00633751 | 0,024747009 | protein_coding |
| ENSMUSG00000023915 | Tnfrsf21 | tumor necrosis factor receptor superfamily, member 21 [Source:MGI Symbol;Acc:MGI:2151075] | 33,9340438 | -0,3630385 | **-1,28613182** | 5,082806 | 6,286635 | 0,01216518 | 0,042661612 | protein_coding |
| ENSMUSG00000029104 | Htt | huntingtin [Source:MGI Symbol;Acc:MGI:96067] | 181,2720771 | -0,3638635 | **-1,2868675** | 7,50151335 | 7,29661505 | 0,006908464 | 0,026623393 | protein_coding |
| ENSMUSG00000068551 | Zfp467 | zinc finger protein 467 [Source:MGI Symbol;Acc:MGI:1916160] | 32,49399375 | -0,3639853 | **-1,28697614** | 5,0156685 | 6,24463506 | 0,012457005 | 0,043366638 | protein_coding |
| ENSMUSG00000052429 | Prmt1 | protein arginine N-methyltransferase 1 [Source:MGI Symbol;Acc:MGI:107846] | 74,20884023 | -0,3647314 | **-1,28764183** | 6,21278195 | 7,52626351 | 0,006080589 | 0,023897272 | protein_coding |
| ENSMUSG00000057963 | Itpk1 | inositol 1,3,4-triphosphate 5/6 kinase [Source:MGI Symbol;Acc:MGI:2446159] | 35,52591201 | -0,3649314 | **-1,28782043** | 5,14683254 | 6,75619771 | 0,009342262 | 0,034346946 | protein_coding |
| ENSMUSG00000029227 | Fip1l1 | FIP1 like 1 (S. cerevisiae) [Source:MGI Symbol;Acc:MGI:1914149] | 97,63341793 | -0,3650342 | **-1,28791212** | 6,61156298 | 8,09770942 | 0,004432123 | 0,018279182 | protein_coding |
| ENSMUSG00000045302 | Preb | prolactin regulatory element binding [Source:MGI Symbol;Acc:MGI:1355326] | 113,879691 | -0,3657542 | **-1,28855504** | 6,82984487 | 7,43126508 | 0,006410026 | 0,025000695 | protein_coding |
| ENSMUSG00000028059 | Arhgef2 | rho/rac guanine nucleotide exchange factor (GEF) 2 [Source:MGI Symbol;Acc:MGI:103264] | 253,8433453 | -0,3662507 | **-1,28899858** | 7,98698749 | 6,43589334 | 0,01118369 | 0,039855725 | protein_coding |
| ENSMUSG00000073131 | Vma21 | VMA21 vacuolar H+-ATPase homolog (S. cerevisiae) [Source:MGI Symbol;Acc:MGI:1914298] | 87,27018048 | -0,3663321 | **-1,28907131** | 6,45035178 | 7,92722572 | 0,004869641 | 0,019742709 | protein_coding |
| ENSMUSG00000070939 | Tgfbrap1 | transforming growth factor, beta receptor associated protein 1 [Source:MGI Symbol;Acc:MGI:2447427] | 79,4741242 | -0,3687246 | **-1,29121088** | 6,31078182 | 7,59943458 | 0,005838661 | 0,023133562 | protein_coding |
| ENSMUSG00000018846 | Pank3 | pantothenate kinase 3 [Source:MGI Symbol;Acc:MGI:2387464] | 80,14642051 | -0,3687499 | **-1,29123349** | 6,32159842 | 6,96801756 | 0,008297938 | 0,031052262 | protein_coding |
| ENSMUSG00000027746 | Ufm1 | ubiquitin-fold modifier 1 [Source:MGI Symbol;Acc:MGI:1915140] | 63,99619747 | -0,3688555 | **-1,29132804** | 6,0002649 | 7,40121861 | 0,006517971 | 0,025349715 | protein_coding |
| ENSMUSG00000033186 | Mzt1 | mitotic spindle organizing protein 1 [Source:MGI Symbol;Acc:MGI:1924039] | 38,58076769 | -0,3690542 | **-1,29150584** | 5,26737272 | 6,9330738 | 0,008461614 | 0,031578721 | protein_coding |
| ENSMUSG00000021537 | Cetn3 | centrin 3 [Source:MGI Symbol;Acc:MGI:1097706] | 30,22540312 | -0,3690815 | **-1,29153028** | 4,91242426 | 6,22090967 | 0,012625023 | 0,043840432 | protein_coding |
| ENSMUSG00000027189 | Trim44 | tripartite motif-containing 44 [Source:MGI Symbol;Acc:MGI:1931835] | 121,1322321 | -0,3691737 | **-1,29161286** | 6,92002501 | 7,86720083 | 0,005033952 | 0,020341313 | protein_coding |
| ENSMUSG00000002319 | Ipo4 | importin 4 [Source:MGI Symbol;Acc:MGI:1923001] | 91,99488876 | -0,369721 | **-1,29210295** | 6,5226367 | 8,129648 | 0,004354728 | 0,018020864 | protein_coding |
| ENSMUSG00000032405 | Pias1 | protein inhibitor of activated STAT 1 [Source:MGI Symbol;Acc:MGI:1913125] | 84,29540116 | -0,3702566 | **-1,29258275** | 6,39581375 | 8,16168134 | 0,004278469 | 0,017792406 | protein_coding |
| ENSMUSG00000031299 | Pdha1 | pyruvate dehydrogenase E1 alpha 1 [Source:MGI Symbol;Acc:MGI:97532] | 110,2660798 | -0,3706211 | **-1,29290935** | 6,78447938 | 7,81811592 | 0,005172509 | 0,020862833 | protein_coding |
| ENSMUSG00000001419 | Mef2d | myocyte enhancer factor 2D [Source:MGI Symbol;Acc:MGI:99533] | 193,1006604 | -0,3709716 | **-1,29322344** | 7,59213036 | 7,59490834 | 0,005853337 | 0,0231727 | protein_coding |
| ENSMUSG00000036918 | Ttc7 | tetratricopeptide repeat domain 7 [Source:MGI Symbol;Acc:MGI:1920999] | 57,88410489 | -0,3718059 | **-1,29397155** | 5,84725237 | 8,00655634 | 0,004660829 | 0,019094576 | protein_coding |
| ENSMUSG00000036528 | Ppfibp2 | PTPRF interacting protein, binding protein 2 (liprin beta 2) [Source:MGI Symbol;Acc:MGI:894649] | 339,9588565 | -0,3724606 | **-1,29455893** | 8,4092911 | 6,10626466 | 0,013470352 | 0,046187199 | protein_coding |
| ENSMUSG00000000838 | Fmr1 | fragile X mental retardation syndrome 1 [Source:MGI Symbol;Acc:MGI:95564] | 45,07631039 | -0,3727326 | **-1,29480298** | 5,48884472 | 7,95541792 | 0,004794364 | 0,019516737 | protein_coding |
| ENSMUSG00000022124 | Fbxl3 | F-box and leucine-rich repeat protein 3 [Source:MGI Symbol;Acc:MGI:1354702] | 83,16834845 | -0,3738585 | **-1,29581382** | 6,37623685 | 8,366863 | 0,003821247 | 0,016185057 | protein_coding |
| ENSMUSG00000021629 | Slc30a5 | solute carrier family 30 (zinc transporter), member 5 [Source:MGI Symbol;Acc:MGI:1916298] | 87,6117725 | -0,3740899 | **-1,29602176** | 6,45176407 | 8,37465776 | 0,003804892 | 0,016153149 | protein_coding |
| ENSMUSG00000044600 | 9130011J15Rik | RIKEN cDNA 9130011J15 gene [Source:MGI Symbol;Acc:MGI:1914068] | 77,0673649 | -0,3741949 | **-1,29611606** | 6,26663903 | 7,93849094 | 0,004839418 | 0,019641921 | protein_coding |
| ENSMUSG00000085328 | Gm17131 | predicted gene 17131 [Source:MGI Symbol;Acc:MGI:4937958] | 130,424252 | -0,3748626 | **-1,29671603** | 7,02520636 | 7,2598344 | 0,007051379 | 0,027098113 | pseudogene |
| ENSMUSG00000028910 | Mecr | mitochondrial trans-2-enoyl-CoA reductase [Source:MGI Symbol;Acc:MGI:1349441] | 34,81906598 | -0,3752313 | **-1,29704749** | 5,12383847 | 7,00757772 | 0,008116542 | 0,030498102 | protein_coding |
| ENSMUSG00000030082 | Sec61a1 | Sec61 alpha 1 subunit (S. cerevisiae) [Source:MGI Symbol;Acc:MGI:1858417] | 179,802262 | -0,3754305 | **-1,29722658** | 7,48874535 | 7,37519078 | 0,006612978 | 0,025655644 | protein_coding |

| | | | | | | | | | | |
|---|---|---|---|---|---|---|---|---|---|---|
| ENSMUSG00000052296 | Ppp6r1 | protein phosphatase 6, regulatory subunit 1 [Source:MGI Symbol;Acc:MGI:2442163] | 180,826663 | -0,3759077 | **-1,29765572** | 7,49732528 | 7,68905133 | 0,005555682 | 0,022172278 | protein_coding |
| ENSMUSG00000063015 | Ccni | cyclin I [Source:MGI Symbol;Acc:MGI:1341077] | 216,8015562 | -0,3761602 | **-1,29788288** | 7,75939454 | 7,29685603 | 0,006907538 | 0,026623393 | protein_coding |
| ENSMUSG00000031403 | Dkc1 | dyskeratosis congenita 1, dyskerin [Source:MGI Symbol;Acc:MGI:1861727] | 25,69987234 | -0,3761621 | **-1,29788457** | 4,69001146 | 6,3190245 | 0,011944932 | 0,042063773 | protein_coding |
| ENSMUSG00000021775 | Nr1d2 | nuclear receptor subfamily 1, group D, member 2 [Source:MGI Symbol;Acc:MGI:2449205] | 46,09325434 | -0,3764807 | **-1,29817123** | 5,52318005 | 7,78884017 | 0,005256993 | 0,021149244 | protein_coding |
| ENSMUSG00000030990 | Pgap2 | post-GPI attachment to proteins 2 [Source:MGI Symbol;Acc:MGI:2385286] | 198,1697739 | -0,3765372 | **-1,29822204** | 7,63101621 | 7,02835934 | 0,008022882 | 0,030218515 | protein_coding |
| ENSMUSG00000036438 | Calm2 | calmodulin 2 [Source:MGI Symbol;Acc:MGI:103250] | 353,8296236 | -0,3768853 | **-1,29853534** | 8,46680402 | 6,04381019 | 0,013955134 | 0,047425011 | protein_coding |
| ENSMUSG00000006005 | Tpr | translocated promoter region [Source:MGI Symbol;Acc:MGI:1922066] | 366,2896839 | -0,3771125 | **-1,29873991** | 8,51646323 | 6,13870865 | 0,013225386 | 0,045470092 | protein_coding |
| ENSMUSG00000040209 | Zfp704 | zinc finger protein 704 [Source:MGI Symbol;Acc:MGI:2180715] | 45,91311945 | -0,3779508 | **-1,29949477** | 5,51288125 | 7,74672476 | 0,005381004 | 0,021561304 | protein_coding |
| ENSMUSG00000038658 | C030046E11Rik | RIKEN cDNA C030046E11 gene [Source:MGI Symbol;Acc:MGI:1924893] | 123,1301908 | -0,3782526 | **-1,29976664** | 6,94423194 | 7,64898331 | 0,005680433 | 0,022563598 | protein_coding |
| ENSMUSG00000062908 | Acadm | acyl-Coenzyme A dehydrogenase, medium chain [Source:MGI Symbol;Acc:MGI:87867] | 61,2566138 | -0,3784713 | **-1,2999637** | 5,93571951 | 7,81335796 | 0,005186145 | 0,020902485 | protein_coding |
| ENSMUSG00000003123 | Lipe | lipase, hormone sensitive [Source:MGI Symbol;Acc:MGI:96790] | 38,27729017 | -0,3794459 | **-1,30084214** | 5,25113994 | 6,52016402 | 0,010665817 | 0,038352705 | protein_coding |
| ENSMUSG00000037089 | Slc35b2 | solute carrier family 35, member B2 [Source:MGI Symbol;Acc:MGI:1921086] | 67,16758438 | -0,380429 | **-1,30172891** | 6,06528168 | 7,67053823 | 0,005612971 | 0,022368413 | protein_coding |
| ENSMUSG00000016344 | Ppdpf | pancreatic progenitor cell differentiation and proliferation factor homolog (zebrafish)RIKEN cDNA 2700038C09 gene [Sou | 29,15796985 | -0,3805039 | **-1,30179644** | 4,85764497 | 6,2423108 | 0,012473363 | 0,04340983 | protein_coding |
| ENSMUSG00000041133 | Smc1a | structural maintenance of chromosomes 1A [Source:MGI Symbol;Acc:MGI:1344345] | 91,21000879 | -0,3809518 | **-1,30220064** | 6,50912757 | 8,54618737 | 0,003462467 | 0,014947789 | protein_coding |
| ENSMUSG00000022329 | Stk3 | serine/threonine kinase 3 [Source:MGI Symbol;Acc:MGI:1928487] | 30,78642493 | -0,3817427 | **-1,30291478** | 4,94892518 | 7,06205203 | 0,007873376 | 0,029777895 | protein_coding |
| ENSMUSG00000029860 | Zyx | zyxin [Source:MGI Symbol;Acc:MGI:103072] | 174,591984 | -0,3821837 | **-1,3033131** | 7,44556924 | 7,76206337 | 0,005335497 | 0,021418016 | protein_coding |
| ENSMUSG00000053436 | Mapk14 | mitogen-activated protein kinase 14 [Source:MGI Symbol;Acc:MGI:1346865] | 149,5522987 | -0,3825255 | **-1,30362189** | 7,22354467 | 8,02494919 | 0,004613732 | 0,018949934 | protein_coding |
| ENSMUSG00000031805 | Jak3 | Janus kinase 3 [Source:MGI Symbol;Acc:MGI:99928] | 40,8127667 | -0,3826695 | **-1,30375206** | 5,34188327 | 6,44293371 | 0,011139453 | 0,039710958 | protein_coding |
| ENSMUSG00000042178 | Armc5 | armadillo repeat containing 5 [Source:MGI Symbol;Acc:MGI:2384586] | 50,93972213 | -0,3830025 | **-1,30405296** | 5,66034198 | 6,80078911 | 0,009111759 | 0,033650722 | protein_coding |
| ENSMUSG00000033128 | Gga1 | golgi associated, gamma adaptin ear containing, ARF binding protein 1 [Source:MGI Symbol;Acc:MGI:2146207] | 151,7447018 | -0,383161 | **-1,30419631** | 7,24327843 | 8,11004914 | 0,004402053 | 0,018175633 | protein_coding |
| ENSMUSG00000020091 | Eif4ebp2 | eukaryotic translation initiation factor 4E binding protein 2 [Source:MGI Symbol;Acc:MGI:109198] | 43,82822696 | -0,3833195 | **-1,30433959** | 5,44669591 | 7,01662499 | 0,008075631 | 0,030354757 | protein_coding |
| ENSMUSG00000024165 | Hn1l | hematological and neurological expressed 1-like [Source:MGI Symbol;Acc:MGI:1196260] | 62,64300542 | -0,3847011 | **-1,30558923** | 5,97152253 | 8,28510856 | 0,003997151 | 0,016813439 | protein_coding |
| ENSMUSG00000030347 | D6Wsu163e | DNA segment, Chr 6, Wayne State University 163, expressed [Source:MGI Symbol;Acc:MGI:107893] | 42,93813666 | -0,3849597 | **-1,30582336** | 5,42461733 | 7,54211179 | 0,006027336 | 0,023727101 | protein_coding |
| ENSMUSG00000060657 | Marf1 | meiosis arrest female 1 [Source:MGI Symbol;Acc:MGI:2444505] | 135,9443096 | -0,3855506 | **-1,30635822** | 7,08569777 | 8,60477046 | 0,003352836 | 0,014520146 | protein_coding |
| ENSMUSG00000049751 | Rpl36al | ribosomal protein L36A-like [Source:MGI Symbol;Acc:MGI:1913733] | 152,1534317 | -0,3861599 | **-1,30691011** | 7,24824057 | 7,79084606 | 0,00525116 | 0,021133515 | protein_coding |
| ENSMUSG00000023277 | Twf2 | twinfilin, actin-binding protein, homolog 2 (Drosophila) [Source:MGI Symbol;Acc:MGI:1346078] | 104,8964876 | -0,3864914 | **-1,30721043** | 6,7072958 | 7,0220656 | 0,00805113 | 0,030283384 | protein_coding |
| ENSMUSG00000023272 | Creld2 | cysteine-rich with EGF-like domains 2 [Source:MGI Symbol;Acc:MGI:1923987] | 27,66627756 | -0,3866117 | **-1,30731943** | 4,78671218 | 6,30719749 | 0,012024877 | 0,042264019 | protein_coding |
| ENSMUSG00000013787 | Ehmt2 | euchromatic histone lysine N-methyltransferase 2 [Source:MGI Symbol;Acc:MGI:2148922] | 231,3380285 | -0,3886392 | **-1,30915802** | 7,85251744 | 7,00484818 | 0,008128927 | 0,030534194 | protein_coding |
| ENSMUSG00000024818 | Slc25a45 | solute carrier family 25, member 45 [Source:MGI Symbol;Acc:MGI:2147731] | 132,8806006 | -0,3887133 | **-1,30922522** | 7,05174356 | 8,27723116 | 0,00401453 | 0,016860719 | protein_coding |
| ENSMUSG00000026094 | Stk17b | serine/threonine kinase 17b (apoptosis-inducing) [Source:MGI Symbol;Acc:MGI:2138162] | 138,755188 | -0,3891118 | **-1,30958693** | 7,12025687 | 8,85381518 | 0,002924769 | 0,012931363 | protein_coding |
| ENSMUSG00000031865 | Dctn1 | dynactin 1 [Source:MGI Symbol;Acc:MGI:107745] | 170,1723772 | -0,3903067 | **-1,310672** | 7,40925379 | 8,63795214 | 0,003292313 | 0,014303141 | protein_coding |
| ENSMUSG00000035397 | Klf16 | Kruppel-like factor 16 [Source:MGI Symbol;Acc:MGI:2153049] | 27,40248996 | -0,3904463 | **-1,31079888** | 4,77196545 | 6,67660334 | 0,00976866 | 0,035692806 | protein_coding |
| ENSMUSG00000014606 | Slc25a11 | solute carrier family 25 (mitochondrial carrier oxoglutarate carrier), member 11 [Source:MGI Symbol;Acc:MGI:1915113] | 120,7224009 | -0,3916122 | **-1,31185857** | 6,91370439 | 9,12449947 | 0,002522089 | 0,011391162 | protein_coding |
| ENSMUSG00000003099 | Ppp5c | protein phosphatase 5, catalytic subunit [Source:MGI Symbol;Acc:MGI:102666] | 92,23080436 | -0,3917335 | **-1,31196887** | 6,52536935 | 8,92462048 | 0,002813518 | 0,01251503 | protein_coding |
| ENSMUSG00000018428 | Akap1 | A kinase (PRKA) anchor protein 1 [Source:MGI Symbol;Acc:MGI:104729] | 22,80223485 | -0,3920525 | **-1,31225902** | 4,50956193 | 6,26207093 | 0,012334993 | 0,043092073 | protein_coding |
| ENSMUSG00000030291 | Med21 | mediator complex subunit 21 [Source:MGI Symbol;Acc:MGI:1347064] | 28,38515642 | -0,3920732 | **-1,31227782** | 4,82923498 | 6,51806638 | 0,010678404 | 0,03836613 | protein_coding |
| ENSMUSG00000020743 | Mif4gd | MIF4G domain containing [Source:MGI Symbol;Acc:MGI:1916924] | 36,5907843 | -0,3920777 | **-1,31228195** | 5,18662658 | 7,70704219 | 0,005500581 | 0,021992315 | protein_coding |
| ENSMUSG00000040459 | Arglu1 | arginine and glutamate rich 1 [Source:MGI Symbol;Acc:MGI:2442985] | 49,22280192 | -0,3923834 | **-1,31256002** | 5,61712932 | 8,32531055 | 0,003909645 | 0,016489549 | protein_coding |
| ENSMUSG00000029192 | Tbc1d14 | TBC1 domain family, member 14 [Source:MGI Symbol;Acc:MGI:1098708] | 109,5931086 | -0,3924057 | **-1,31258032** | 6,77447702 | 8,16767406 | 0,004264356 | 0,01774043 | protein_coding |
| ENSMUSG00000033697 | Arhgap39 | Rho GTPase activating protein 39 [Source:MGI Symbol;Acc:MGI:107858] | 27,04001927 | -0,3927034 | **-1,31285123** | 4,7427336 | 6,96875277 | 0,008294529 | 0,031050082 | protein_coding |
| ENSMUSG00000061288 | Taok3 | TAO kinase 3 [Source:MGI Symbol;Acc:MGI:3041177] | 221,0622508 | -0,3935829 | **-1,31365182** | 7,78770942 | 7,96999164 | 0,004755916 | 0,019398364 | protein_coding |
| ENSMUSG00000036241 | Ube2r2 | ubiquitin-conjugating enzyme E2R 2 [Source:MGI Symbol;Acc:MGI:1914865] | 128,6277819 | -0,3936196 | **-1,31368516** | 7,00613508 | 8,61274805 | 0,003338183 | 0,014473801 | protein_coding |
| ENSMUSG00000021794 | Glud1 | glutamate dehydrogenase 1 [Source:MGI Symbol;Acc:MGI:95753] | 368,0148096 | -0,3940133 | **-1,31404373** | 8,52345332 | 6,56366937 | 0,010408153 | 0,037567141 | protein_coding |
| ENSMUSG00000090137 | Uba52 | ubiquitin A-52 residue ribosomal protein fusion product 1 [Source:MGI Symbol;Acc:MGI:98887] | 190,4666268 | -0,3948066 | **-1,31476654** | 7,57074895 | 8,45427705 | 0,00364186 | 0,015593575 | protein_coding |
| ENSMUSG00000031681 | Smad1 | SMAD family member 1 [Source:MGI Symbol;Acc:MGI:109452] | 35,25997566 | -0,3949526 | **-1,31489953** | 5,14044589 | 7,02256015 | 0,008048907 | 0,030283384 | protein_coding |
| ENSMUSG00000031198 | Fundc2 | FUN14 domain containing 2 [Source:MGI Symbol;Acc:MGI:1914641] | 43,89768913 | -0,3950053 | **-1,3149476** | 5,45467424 | 8,5747327 | 0,0034086 | 0,014721025 | protein_coding |
| ENSMUSG00000001964 | Emd | emerin [Source:MGI Symbol;Acc:MGI:108117] | 45,17241832 | -0,3950304 | **-1,31497044** | 5,49847626 | 8,15387665 | 0,004296921 | 0,017848873 | protein_coding |
| ENSMUSG00000075415 | Fnbp1 | formin binding protein 1 [Source:MGI Symbol;Acc:MGI:109606] | 279,8735972 | -0,3951343 | **-1,31506515** | 8,12818092 | 7,38976257 | 0,006559615 | 0,025484613 | protein_coding |
| ENSMUSG00000047123 | Ticam1 | toll-like receptor adaptor molecule 1 [Source:MGI Symbol;Acc:MGI:2147032] | 68,47108165 | -0,3958389 | **-1,31570761** | 6,09645708 | 8,69618255 | 0,003188772 | 0,013891766 | protein_coding |
| ENSMUSG00000020941 | Map3k14 | mitogen-activated protein kinase kinase kinase 14 [Source:MGI Symbol;Acc:MGI:1858204] | 47,54409372 | -0,3959366 | **-1,31579669** | 5,56624343 | 7,80594827 | 0,005207453 | 0,02097298 | protein_coding |
| ENSMUSG00000025648 | Pfkfb4 | 6-phosphofructo-2-kinase/fructose-2,6-biphosphatase 4 [Source:MGI Symbol;Acc:MGI:2687284] | 42,74928657 | -0,3962193 | **-1,31605454** | 5,41023352 | 6,48842543 | 0,010857911 | 0,03883329 | protein_coding |
| ENSMUSG00000036054 | Sugp2 | SURP and G patch domain containing 2 [Source:MGI Symbol;Acc:MGI:2678085] | 44,22138711 | -0,3964001 | **-1,31621948** | 5,46160592 | 7,70412103 | 0,005509489 | 0,02201265 | protein_coding |
| ENSMUSG00000000628 | Hk2 | hexokinase 2 [Source:MGI Symbol;Acc:MGI:1315197] | 154,5144169 | -0,3964006 | **-1,31621991** | 7,27150574 | 8,47149573 | 0,003607547 | 0,015452677 | protein_coding |
| ENSMUSG00000028282 | Casp8ap2 | caspase 8 associated protein 2 [Source:MGI Symbol;Acc:MGI:1349399] | 27,49637813 | -0,3968929 | **-1,31666918** | 4,76885201 | 7,52738011 | 0,006076822 | 0,02389622 | protein_coding |
| ENSMUSG00000027466 | Rbck1 | RanBP-type and C3HC4-type zinc finger containing 1 [Source:MGI Symbol;Acc:MGI:1344372] | 72,1664888 | -0,3969614 | **-1,31673172** | 6,16678374 | 7,46346648 | 0,006296364 | 0,024627325 | protein_coding |
| ENSMUSG00000057530 | Ece1 | endothelin converting enzyme 1 [Source:MGI Symbol;Acc:MGI:1101357] | 59,98323502 | -0,3979033 | **-1,31759168** | 5,90408086 | 8,73998626 | 0,003113069 | 0,013654006 | protein_coding |
| ENSMUSG00000059555 | A830007P12Rik | RIKEN cDNA A830007P12 gene [Source:MGI Symbol;Acc:MGI:2442720] | 151,7641403 | -0,3982703 | **-1,31792688** | 7,24328588 | 8,25684366 | 0,004059865 | 0,017025092 | protein_coding |
| ENSMUSG00000040327 | Cul9 | cullin 9 [Source:MGI Symbol;Acc:MGI:1925559] | 60,4606281 | -0,3983132 | **-1,31796603** | 5,90916316 | 9,07883517 | 0,002585847 | 0,01164373 | protein_coding |
| ENSMUSG00000049988 | Lrrc25 | leucine rich repeat containing 25 [Source:MGI Symbol;Acc:MGI:2445284] | 272,5559531 | -0,3985441 | **-1,31817704** | 8,08968715 | 6,86601277 | 0,008785044 | 0,032586524 | protein_coding |
| ENSMUSG00000073982 | Rhog | ras homolog gene family, member G [Source:MGI Symbol;Acc:MGI:1928370] | 363,7892053 | -0,3986331 | **-1,31825831** | 8,50651625 | 6,29401515 | 0,012114631 | 0,042511482 | protein_coding |
| ENSMUSG00000058997 | Vwa8 | von Willebrand factor A domain containing 8 [Source:MGI Symbol;Acc:MGI:1919008] | 95,55029769 | -0,399236 | **-1,3188093** | 6,57750796 | 9,49471816 | 0,002060642 | 0,009617377 | protein_coding |
| ENSMUSG00000040760 | Appl1 | adaptor protein, phosphotyrosine interaction, PH domain and leucine zipper containing 1 [Source:MGI Symbol;Acc:MGI:' | 74,02585332 | -0,3995962 | **-1,31913867** | 6,20872158 | 9,21783313 | 0,00239669 | 0,010953593 | protein_coding |
| ENSMUSG00000035649 | Zcchc7 | zinc finger, CCHC domain containing 7 [Source:MGI Symbol;Acc:MGI:2442912] | 33,96205244 | -0,4002118 | **-1,31970164** | 5,07553106 | 7,43950272 | 0,006380752 | 0,024900199 | protein_coding |
| ENSMUSG00000002014 | Ssr4 | signal sequence receptor, delta [Source:MGI Symbol;Acc:MGI:1099464] | 87,30174883 | -0,400597 | **-1,32005403** | 6,44428822 | 9,29361061 | 0,002299545 | 0,010544699 | protein_coding |
| ENSMUSG00000024063 | Lbh | limb-bud and heart [Source:MGI Symbol;Acc:MGI:1925139] | 34,61328131 | -0,4010952 | **-1,32050996** | 5,11518173 | 6,23762992 | 0,012506373 | 0,043483393 | protein_coding |
| ENSMUSG00000037965 | Zc3h7a | zinc finger CCCH type containing 7 A [Source:MGI Symbol;Acc:MGI:2445044] | 105,7351639 | -0,4015365 | **-1,32091395** | 6,72199166 | 9,04316306 | 0,00263679 | 0,011843996 | protein_coding |
| ENSMUSG00000031402 | Mpp1 | membrane protein, palmitoylated [Source:MGI Symbol;Acc:MGI:105941] | 269,4161894 | -0,4015484 | **-1,32092485** | 8,07335057 | 7,80505126 | 0,005210039 | 0,020975704 | protein_coding |
| ENSMUSG00000032349 | Elovl5 | ELOVL family member 5, elongation of long chain fatty acids (yeast) [Source:MGI Symbol;Acc:MGI:1916051] | 47,6418588 | -0,402821 | **-1,32209055** | 5,56911912 | 8,18906786 | 0,004214357 | 0,017559023 | protein_coding |
| ENSMUSG00000026078 | Pdcl3 | phosducin-like 3 [Source:MGI Symbol;Acc:MGI:1916083] | 51,33791234 | -0,4034165 | **-1,32263641** | 5,68499497 | 8,72995717 | 0,003130238 | 0,013707425 | protein_coding |
| ENSMUSG00000019188 | H13 | histocompatibility 13 [Source:MGI Symbol;Acc:MGI:95886] | 116,5662861 | -0,4035679 | **-1,3227752** | 6,86279372 | 9,18913191 | 0,002434563 | 0,011085183 | protein_coding |
| ENSMUSG00000021275 | Tecpr2 | tectonin beta-propeller repeat containing 2 [Source:MGI Symbol;Acc:MGI:2144865] | 44,6730425 | -0,4039701 | **-1,32314401** | 5,47616095 | 8,40501166 | 0,00374188 | 0,015965839 | protein_coding |

| | | | | | | | | | | |
|---|---|---|---|---|---|---|---|---|---|---|
| ENSMUSG00000023007 | Prpf40b | PRP40 pre-mRNA processing factor 40 homolog B (yeast) [Source:MGI Symbol;Acc:MGI:1925583] | 18,25895292 | -0,4042963 | **-1,32344321** | 4,18171953 | 6,12190593 | 0,013351677 | 0,045856478 | protein_coding |
| ENSMUSG00000027363 | Usp8 | ubiquitin specific peptidase 8 [Source:MGI Symbol;Acc:MGI:1934029] | 241,7937245 | -0,4043587 | **-1,32350042** | 7,91734496 | 8,18228165 | 0,004230152 | 0,017604803 | protein_coding |
| ENSMUSG00000022336 | Eif3e | eukaryotic translation initiation factor 3, subunit E [Source:MGI Symbol;Acc:MGI:99257] | 169,4797503 | -0,4046009 | **-1,32372263** | 7,40459835 | 9,12755657 | 0,002517878 | 0,011384331 | protein_coding |
| ENSMUSG00000054920 | Klhl5 | kelch-like 5 (Drosophila) [Source:MGI Symbol;Acc:MGI:1919028] | 114,1934196 | -0,4046035 | **-1,32372505** | 6,83337539 | 9,53109889 | 0,002020191 | 0,00944906 | protein_coding |
| ENSMUSG00000039853 | Trim14 | tripartite motif-containing 14 [Source:MGI Symbol;Acc:MGI:1921985] | 44,71897625 | -0,4057315 | **-1,32476047** | 5,47355832 | 8,73439078 | 0,003122636 | 0,013685044 | protein_coding |
| ENSMUSG00000042626 | Shc1 | src homology 2 domain-containing transforming protein C1 [Source:MGI Symbol;Acc:MGI:98296] | 104,9303302 | -0,4059381 | **-1,32495017** | 6,71091129 | 8,42954431 | 0,003691729 | 0,01577636 | protein_coding |
| ENSMUSG00000036257 | Pnpla8 | patatin-like phospholipase domain containing 8 [Source:MGI Symbol;Acc:MGI:1914702] | 54,7689764 | -0,4061143 | **-1,32511203** | 5,77788227 | 9,39433791 | 0,002176566 | 0,01008601 | protein_coding |
| ENSMUSG00000030760 | Acer3 | alkaline ceramidase 3 [Source:MGI Symbol;Acc:MGI:1913440] | 31,6261066 | -0,4062288 | **-1,32521716** | 4,98516836 | 8,00333044 | 0,004669139 | 0,019098182 | protein_coding |
| ENSMUSG00000027854 | Sike1 | suppressor of IKBKE 1 [Source:MGI Symbol;Acc:MGI:1913891] | 44,56379604 | -0,4062526 | **-1,32523902** | 5,47569349 | 8,47171321 | 0,003607115 | 0,015452677 | protein_coding |
| ENSMUSG00000025375 | Aatk | apoptosis-associated tyrosine kinase [Source:MGI Symbol;Acc:MGI:1197518] | 24,56188993 | -0,4064034 | **-1,32537753** | 4,60320684 | 6,82472736 | 0,008990428 | 0,033292158 | protein_coding |
| ENSMUSG00000051864 | Tbc1d22a | TBC1 domain family, member 22a [Source:MGI Symbol;Acc:MGI:1289265] | 100,6624729 | -0,4065304 | **-1,32549428** | 6,64930415 | 9,95486346 | 0,00160425 | 0,007757877 | protein_coding |
| ENSMUSG00000054428 | Atpif1 | ATPase inhibitory factor 1 [Source:MGI Symbol;Acc:MGI:1196457] | 92,14933815 | -0,4065523 | **-1,32551435** | 6,52383577 | 9,92487445 | 0,0016306 | 0,007871441 | protein_coding |
| ENSMUSG00000028708 | Mknk1 | MAP kinase-interacting serine/threonine kinase 1 [Source:MGI Symbol;Acc:MGI:894316] | 77,20399946 | -0,4066334 | **-1,32558886** | 6,26968912 | 8,58278908 | 0,003393552 | 0,014667656 | protein_coding |
| ENSMUSG00000059436 | Max | Max protein [Source:MGI Symbol;Acc:MGI:96921] | 82,00858345 | -0,4069043 | **-1,3258378** | 6,35470356 | 9,6801602 | 0,001862684 | 0,008828864 | protein_coding |
| ENSMUSG00000033454 | Zbtb1 | zinc finger and BTB domain containing 1 [Source:MGI Symbol;Acc:MGI:2442326] | 52,40360191 | -0,4070504 | **-1,3259721** | 5,70737834 | 8,66071962 | 0,003251429 | 0,014142299 | protein_coding |
| ENSMUSG00000057229 | Atp5sl | ATP5S-like [Source:MGI Symbol;Acc:MGI:1913599] | 20,96126284 | -0,4074796 | **-1,32636665** | 4,37448996 | 6,58605488 | 0,010278069 | 0,037256729 | protein_coding |
| ENSMUSG00000029723 | Tsc22d4 | TSC22 domain family, member 4 [Source:MGI Symbol;Acc:MGI:1926079] | 145,5111627 | -0,4075686 | **-1,32644842** | 7,18250927 | 9,42157875 | 0,002144468 | 0,009962428 | protein_coding |
| ENSMUSG00000025223 | Ldb1 | LIM domain binding 1 [Source:MGI Symbol;Acc:MGI:894762] | 95,1703143 | -0,4079767 | **-1,32682371** | 6,56844374 | 9,82819844 | 0,001718566 | 0,008257895 | protein_coding |
| ENSMUSG00000016933 | Plcg1 | phospholipase C, gamma 1 [Source:MGI Symbol;Acc:MGI:97615] | 24,97846408 | -0,408132 | **-1,32696656** | 4,64088855 | 7,30662528 | 0,006870083 | 0,026521996 | protein_coding |
| ENSMUSG00000020272 | Stk10 | serine/threonine kinase 10 [Source:MGI Symbol;Acc:MGI:1099439] | 58,04614149 | -0,4082665 | **-1,32709024** | 5,85164059 | 8,79270563 | 0,003024374 | 0,013318154 | protein_coding |
| ENSMUSG00000022159 | Rab2b | RAB2B, member RAS oncogene family [Source:MGI Symbol;Acc:MGI:1923588] | 23,65170982 | -0,4083256 | **-1,32714467** | 4,55616865 | 5,98972188 | 0,014389471 | 0,048645267 | protein_coding |
| ENSMUSG00000032299 | Commd4 | COMM domain containing 4 [Source:MGI Symbol;Acc:MGI:1913449] | 31,00951475 | -0,4084729 | **-1,32728016** | 4,949405 | 7,49888052 | 0,006173736 | 0,024202212 | protein_coding |
| ENSMUSG00000037013 | Ss18 | synovial sarcoma translocation, Chromosome 18 [Source:MGI Symbol;Acc:MGI:107708] | 67,35283417 | -0,4090716 | **-1,32783106** | 6,07351674 | 9,90896759 | 0,001644754 | 0,007932795 | protein_coding |
| ENSMUSG00000040945 | Rcc2 | regulator of chromosome condensation 2 [Source:MGI Symbol;Acc:MGI:1919784] | 202,5271949 | -0,4093553 | **-1,32809215** | 7,66052859 | 8,68651547 | 0,003205729 | 0,013960105 | protein_coding |
| ENSMUSG00000024663 | Rab3il1 | RAB3A interacting protein (rabin3)-like 1 [Source:MGI Symbol;Acc:MGI:1922010] | 193,1042331 | -0,4094384 | **-1,32816873** | 7,59226289 | 8,13210372 | 0,004348826 | 0,018003222 | protein_coding |
| ENSMUSG00000029142 | Mrpl33 | mitochondrial ribosomal protein L33 [Source:MGI Symbol;Acc:MGI:2137225] | 30,66283384 | -0,4104619 | **-1,32911124** | 4,93451444 | 8,18403559 | 0,004226064 | 0,017594454 | protein_coding |
| ENSMUSG00000007880 | Arid1a | AT rich interactive domain 1A (SWI-like) [Source:MGI Symbol;Acc:MGI:1935147] | 218,8147433 | -0,4110969 | **-1,32969646** | 7,77300064 | 8,40227227 | 0,003747523 | 0,015977506 | protein_coding |
| ENSMUSG00000045410 | Akr1e1 | aldo-keto reductase family 1, member E1 [Source:MGI Symbol;Acc:MGI:1914758] | 43,65784052 | -0,4111205 | **-1,32971812** | 5,4494195 | 8,97454173 | 0,002737672 | 0,012217224 | protein_coding |
| ENSMUSG00000073491 | Pydc4 | pyrin domain containing 4 [Source:MGI Symbol;Acc:MGI:3695276] | 19,22798444 | -0,411682 | **-1,33023577** | 4,27096847 | 6,5763589 | 0,010334207 | 0,037386216 | protein_coding |
| ENSMUSG00000021785 | Ngly1 | N-glycanase 1 [Source:MGI Symbol;Acc:MGI:1913276] | 27,17700714 | -0,4118272 | **-1,3303697** | 4,76278147 | 6,96414586 | 0,008315912 | 0,031108929 | protein_coding |
| ENSMUSG00000037573 | Tob1 | transducer of ErbB-2.1 [Source:MGI Symbol;Acc:MGI:1349721] | 29,24031264 | -0,4118924 | **-1,33042978** | 4,86313416 | 8,11516423 | 0,00438965 | 0,018131233 | protein_coding |
| ENSMUSG00000003423 | Pih1d1 | PIH1 domain containing 1 [Source:MGI Symbol;Acc:MGI:1916095] | 36,16687186 | -0,4121714 | **-1,33068708** | 5,17036487 | 6,86832666 | 0,008773677 | 0,032555348 | protein_coding |
| ENSMUSG00000055720 | Ubl7 | ubiquitin-like 7 (bone marrow stromal cell-derived) [Source:MGI Symbol;Acc:MGI:1916709] | 52,36721798 | -0,4139565 | **-1,33233466** | 5,70554424 | 8,71321499 | 0,003159116 | 0,013788604 | protein_coding |
| ENSMUSG00000040651 | D14Abb1e | DNA segment, Chr 14, Abbott 1 expressed [Source:MGI Symbol;Acc:MGI:1921694] | 42,89133097 | -0,414204 | **-1,33256324** | 5,41762129 | 7,64292093 | 0,005699555 | 0,022631373 | protein_coding |
| ENSMUSG00000025040 | Fundc1 | FUN14 domain containing 1 [Source:MGI Symbol;Acc:MGI:1919268] | 41,57366115 | -0,4142246 | **-1,33258229** | 5,37210828 | 8,71312651 | 0,003159269 | 0,013788604 | protein_coding |
| ENSMUSG00000047676 | Rpsa-ps10 | ribosomal protein SA, pseudogene 10 [Source:MGI Symbol;Acc:MGI:3704228] | 132,9633068 | -0,4143394 | **-1,33268827** | 7,05701188 | 7,62346368 | 0,005761373 | 0,022843812 | protein_coding |
| ENSMUSG00000021427 | Ssr1 | signal sequence receptor, alpha [Source:MGI Symbol;Acc:MGI:105082] | 156,3062375 | -0,4145412 | **-1,33287472** | 7,28775753 | 9,26753736 | 0,002332511 | 0,010686947 | protein_coding |
| ENSMUSG00000061758 | Akr1b10 | aldo-keto reductase family 1, member B10 (aldose reductase) [Source:MGI Symbol;Acc:MGI:1915111] | 50,9239701 | -0,4146903 | **-1,33301247** | 5,67060674 | 9,48863423 | 0,002067487 | 0,009637453 | protein_coding |
| ENSMUSG00000040410 | Fbxl4 | F-box and leucine-rich repeat protein 4 [Source:MGI Symbol;Acc:MGI:2140367] | 19,50445402 | -0,414806 | **-1,33311943** | 4,2752907 | 6,25549998 | 0,01238083 | 0,043156148 | protein_coding |
| ENSMUSG00000038502 | Ptov1 | prostate tumor over expressed gene 1 [Source:MGI Symbol;Acc:MGI:1933946] | 75,28013324 | -0,4155234 | **-1,3337825** | 6,22927713 | 9,04164714 | 0,002638978 | 0,011848977 | protein_coding |
| ENSMUSG00000039879 | Heca | headcase homolog (Drosophila) [Source:MGI Symbol;Acc:MGI:2685715] | 35,03650577 | -0,4155628 | **-1,33381894** | 5,13400845 | 7,7491983 | 0,005373639 | 0,021539647 | protein_coding |
| ENSMUSG00000042548 | Asxl1 | additional sex combs like 1 [Source:MGI Symbol;Acc:MGI:2684063] | 116,7503884 | -0,4157152 | **-1,33395985** | 6,86694532 | 9,66072384 | 0,001882494 | 0,008899727 | protein_coding |
| ENSMUSG00000020576 | Nbas | neuroblastoma amplified sequence [Source:MGI Symbol;Acc:MGI:1918419] | 26,04267873 | -0,4165188 | **-1,33470307** | 4,70202704 | 8,00605605 | 0,004662117 | 0,019094576 | protein_coding |
| ENSMUSG00000026277 | Stk25 | serine/threonine kinase 25 (yeast) [Source:MGI Symbol;Acc:MGI:1891699] | 72,24536159 | -0,4168285 | **-1,3349896** | 6,17254027 | 8,89231771 | 0,00286373 | 0,012702382 | protein_coding |
| ENSMUSG00000006299 | Aamp | angio-associated migratory protein [Source:MGI Symbol;Acc:MGI:107809] | 179,5883986 | -0,4170211 | **-1,33516783** | 7,48703855 | 9,04931393 | 0,002627934 | 0,011809044 | protein_coding |
| ENSMUSG00000020640 | Itsn2 | intersectin 2 [Source:MGI Symbol;Acc:MGI:1338049] | 178,3687193 | -0,417277 | **-1,33540466** | 7,47871046 | 9,6624034 | 0,001880774 | 0,008895422 | protein_coding |
| ENSMUSG00000061331 | Gm17132 | predicted gene 17132 [Source:MGI Symbol;Acc:MGI:4937959] | 31,93929853 | -0,4175145 | **-1,33562453** | 4,98664695 | 7,41574863 | 0,006465541 | 0,025181459 | pseudogene |
| ENSMUSG00000025937 | Lactb2 | lactamase, beta 2 [Source:MGI Symbol;Acc:MGI:2442551] | 51,19152195 | -0,4178154 | **-1,33590311** | 5,67145017 | 7,96348468 | 0,004773043 | 0,019444356 | protein_coding |
| ENSMUSG00000003746 | Man1a | mannosidase 1, alpha [Source:MGI Symbol;Acc:MGI:104677] | 140,1181358 | -0,4178544 | **-1,33593925** | 7,12788358 | 10,1171013 | 0,001468999 | 0,007170096 | protein_coding |
| ENSMUSG00000014426 | Map3k4 | mitogen-activated protein kinase kinase kinase 4 [Source:MGI Symbol;Acc:MGI:1346875] | 28,70694267 | -0,4181084 | **-1,33617449** | 4,8396266 | 7,51185173 | 0,006129432 | 0,024051455 | protein_coding |
| ENSMUSG00000027108 | Ola1 | Obg-like ATPase 1 [Source:MGI Symbol;Acc:MGI:1914309] | 73,58860328 | -0,418152 | **-1,33621487** | 6,20087531 | 10,6375293 | 0,001108156 | 0,005605577 | protein_coding |
| ENSMUSG00000036622 | Atp13a2 | ATPase type 13A2 [Source:MGI Symbol;Acc:MGI:1922022] | 538,308573 | -0,4181996 | **-1,33625898** | 9,071598 | 6,03395088 | 0,014033289 | 0,047646397 | protein_coding |
| ENSMUSG00000085438 | 1700020I14Rik | RIKEN cDNA 1700020I14 gene [Source:MGI Symbol;Acc:MGI:1913852] | 107,4395342 | -0,4184402 | **-1,33648182** | 6,74343482 | 10,09043 | 0,001490413 | 0,007258497 | lincRNA |
| ENSMUSG00000050628 | Fam100b | family with sequence similarity 100, member B [Source:MGI Symbol;Acc:MGI:1914635] | 30,16792146 | -0,4191799 | **-1,33716723** | 4,92046507 | 7,40149654 | 0,006516964 | 0,025349715 | protein_coding |
| ENSMUSG00000024219 | Anks1 | ankyrin repeat and SAM domain containing 1 [Source:MGI Symbol;Acc:MGI:2446180] | 110,5701042 | -0,4192155 | **-1,33720021** | 6,78825038 | 8,51465728 | 0,003522973 | 0,015149417 | protein_coding |
| ENSMUSG00000034616 | Ssh3 | slingshot homolog 3 (Drosophila) [Source:MGI Symbol;Acc:MGI:2683546] | 128,3295565 | -0,4192246 | **-1,33720869** | 7,00098133 | 8,85062572 | 0,002929884 | 0,01293836 | protein_coding |
| ENSMUSG00000030083 | Abtb1 | ankyrin repeat and  BTB (POZ) domain containing 1 [Source:MGI Symbol;Acc:MGI:1933148] | 19,69115537 | -0,4193279 | **-1,33730438** | 4,28614502 | 6,14188731 | 0,013201634 | 0,045426354 | protein_coding |
| ENSMUSG00000039704 | Lmbrd2 | LMBR1 domain containing 2 [Source:MGI Symbol;Acc:MGI:2444173] | 35,25691374 | -0,4201314 | **-1,33804944** | 5,13805162 | 9,09607986 | 0,002561579 | 0,011548655 | protein_coding |
| ENSMUSG00000010205 | Raver1 | ribonucleoprotein, PTB-binding 1 [Source:MGI Symbol;Acc:MGI:1919016] | 115,4056475 | -0,4201987 | **-1,33811181** | 6,84848238 | 10,4323501 | 0,001238271 | 0,006167217 | protein_coding |
| ENSMUSG00000090946 | Ccdc71l | coiled-coil domain containing 71 like [Source:MGI Symbol;Acc:MGI:1919373] | 73,66098587 | -0,4202808 | **-1,33818801** | 6,2008373 | 8,79128547 | 0,003026729 | 0,013323186 | protein_coding |
| ENSMUSG00000035443 | Thyn1 | thymocyte nuclear protein 1 [Source:MGI Symbol;Acc:MGI:1925112] | 35,48821724 | -0,4204589 | **-1,33835324** | 5,1466881 | 8,43230962 | 0,003686118 | 0,015770788 | protein_coding |
| ENSMUSG00000022678 | Nde1 | nuclear distribution gene E homolog 1 (A nidulans) [Source:MGI Symbol;Acc:MGI:1914453] | 62,76438586 | -0,4208419 | **-1,33870854** | 5,96830646 | 10,1414299 | 0,001449737 | 0,007091833 | protein_coding |
| ENSMUSG00000020009 | Ifngr1 | interferon gamma receptor 1 [Source:MGI Symbol;Acc:MGI:107655] | 78,30300532 | -0,4212615 | **-1,33909795** | 6,28837266 | 10,1447031 | 0,001447165 | 0,007082405 | protein_coding |
| ENSMUSG00000043881 | Kbtbd7 | kelch repeat and BTB (POZ) domain containing 7 [Source:MGI Symbol;Acc:MGI:2685141] | 18,21625687 | -0,4214887 | **-1,33930886** | 4,179463 | 6,4970162 | 0,010805569 | 0,038696474 | protein_coding |
| ENSMUSG00000027479 | Mapre1 | microtubule-associated protein, RP/EB family, member 1 [Source:MGI Symbol;Acc:MGI:891995] | 264,3273241 | -0,4216984 | **-1,33950356** | 8,04601659 | 8,40387222 | 0,003744226 | 0,015969647 | protein_coding |
| ENSMUSG00000027009 | Itga4 | integrin alpha 4 [Source:MGI Symbol;Acc:MGI:96603] | 120,9189134 | -0,4222138 | **-1,33998218** | 6,91689794 | 10,2040994 | 0,001401288 | 0,006864 | protein_coding |
| ENSMUSG00000025246 | Tbl1x | transducin (beta)-like 1 X-linked [Source:MGI Symbol;Acc:MGI:1336172] | 160,7089903 | -0,4233907 | **-1,34107569** | 7,32783759 | 9,76461696 | 0,001779026 | 0,008516844 | protein_coding |
| ENSMUSG00000093904 | Tomm20 | translocase of outer mitochondrial membrane 20 homolog (yeast) [Source:MGI Symbol;Acc:MGI:1915202] | 101,7443584 | -0,423478 | **-1,34115683** | 6,6665326 | 11,198891 | 0,000818462 | 0,004327452 | protein_coding |
| ENSMUSG00000040007 | Bahd1 | bromo adjacent homology domain containing 1 [Source:MGI Symbol;Acc:MGI:2139371] | 151,9347915 | -0,4243572 | **-1,34197446** | 7,24761192 | 10,5374988 | 0,001169766 | 0,005868594 | protein_coding |
| ENSMUSG00000095687 | Rnaset2a | ribonuclease T2A [Source:MGI Symbol;Acc:MGI:1915445] | 30,40452618 | -0,4253624 | **-1,34290977** | 4,92127433 | 7,91654232 | 0,00489848 | 0,019837671 | protein_coding |
| ENSMUSG00000057110 | Cep110 | centrosomal protein 110 [Source:MGI Symbol;Acc:MGI:1889576] | 66,67867439 | -0,4254615 | **-1,34300204** | 6,05306655 | 10,1339187 | 0,001455656 | 0,007113542 | protein_coding |

| | | | | | | | | | | |
|---|---|---|---|---|---|---|---|---|---|---|
| ENSMUSG00000036246 | Gmip | Gem-interacting protein [Source:MGI Symbol;Acc:MGI:1926066] | 262,4538713 | -0,4263822 | **-1,34385936** | 8,03511931 | 8,75483128 | 0,003087829 | 0,013554128 | protein_coding |
| ENSMUSG00000031969 | Acad8 | acyl-Coenzyme A dehydrogenase family, member 8 [Source:MGI Symbol;Acc:MGI:1914198] | 27,87249773 | -0,4264231 | **-1,34389747** | 4,79667078 | 7,97492347 | 0,004742976 | 0,019359679 | protein_coding |
| ENSMUSG00000029992 | Gfpt1 | glutamine fructose-6-phosphate transaminase 1 [Source:MGI Symbol;Acc:MGI:95698] | 100,6965982 | -0,4264936 | **-1,34396321** | 6,6519745 | 9,68947517 | 0,001853265 | 0,008799403 | protein_coding |
| ENSMUSG00000050732 | Vamp8 | vesicle-associated membrane protein 8 [Source:MGI Symbol;Acc:MGI:1336882] | 159,0479994 | -0,427104 | **-1,34453196** | 7,31229655 | 9,39062407 | 0,00218098 | 0,010102201 | protein_coding |
| ENSMUSG00000022089 | Bin3 | bridging integrator 3 [Source:MGI Symbol;Acc:MGI:1929883] | 65,49638917 | -0,4275193 | **-1,34491906** | 6,03067767 | 9,11982461 | 0,002528542 | 0,01141376 | protein_coding |
| ENSMUSG00000002222 | Rmnd5a | required for meiotic nuclear division 5 homolog A (S. cerevisiae) [Source:MGI Symbol;Acc:MGI:1915727] | 86,71013786 | -0,4276059 | **-1,34499971** | 6,43716035 | 9,56601715 | 0,001982123 | 0,009294746 | protein_coding |
| ENSMUSG00000036099 | Vezt | vezatin, adherens junctions transmembrane protein [Source:MGI Symbol;Acc:MGI:2143698] | 61,98112375 | -0,4277032 | **-1,34509041** | 5,95258075 | 10,0375503 | 0,00153381 | 0,007456626 | protein_coding |
| ENSMUSG00000028633 | Ctps | cytidine 5'-triphosphate synthase [Source:MGI Symbol;Acc:MGI:1858304] | 18,62458176 | -0,4282625 | **-1,34561203** | 4,23744251 | 6,75248698 | 0,00936171 | 0,034400372 | protein_coding |
| ENSMUSG00000066551 | Hmgb1 | high mobility group box 1 [Source:MGI Symbol;Acc:MGI:96113] | 93,00869313 | -0,4283091 | **-1,34565546** | 6,53962162 | 10,7620395 | 0,001036032 | 0,005291901 | protein_coding |
| ENSMUSG00000048930 | Tada3 | transcriptional adaptor 3 [Source:MGI Symbol;Acc:MGI:1915724] | 31,90144321 | -0,4283869 | **-1,34572803** | 4,98470225 | 8,3066002 | 0,003950126 | 0,016628367 | protein_coding |
| ENSMUSG00000019139 | Isyna1 | myo-inositol 1-phosphate synthase A1 [Source:MGI Symbol;Acc:MGI:1919030] | 66,70000178 | -0,4283888 | **-1,34572984** | 6,05809145 | 10,3678708 | 0,001282273 | 0,006351821 | protein_coding |
| ENSMUSG00000031029 | Eif3f | eukaryotic translation initiation factor 3, subunit F [Source:MGI Symbol;Acc:MGI:1913335] | 196,4821931 | -0,4292038 | **-1,34649029** | 7,61634946 | 9,18426088 | 0,002441051 | 0,011105518 | protein_coding |
| ENSMUSG00000022770 | Dlg1 | discs, large homolog 1 (Drosophila) [Source:MGI Symbol;Acc:MGI:107231] | 78,44859916 | -0,4294196 | **-1,3466917** | 6,2924702 | 11,0078321 | 0,000907277 | 0,004722052 | protein_coding |
| ENSMUSG00000047694 | Yipf6 | Yip1 domain family, member 6 [Source:MGI Symbol;Acc:MGI:1925179] | 60,85214873 | -0,4301556 | **-1,3473789** | 5,92577906 | 10,509964 | 0,001187326 | 0,005937709 | protein_coding |
| ENSMUSG00000032440 | Tgfbr2 | transforming growth factor, beta receptor II [Source:MGI Symbol;Acc:MGI:98729] | 183,8363866 | -0,4302702 | **-1,34748594** | 7,52193286 | 9,24279405 | 0,002364239 | 0,010814279 | protein_coding |
| ENSMUSG00000003062 | Stard3nl | STARD3 N-terminal like [Source:MGI Symbol;Acc:MGI:1923455] | 58,92507538 | -0,4302972 | **-1,34751112** | 5,87755954 | 8,78340472 | 0,003039833 | 0,013375508 | protein_coding |
| ENSMUSG00000039585 | Myo9a | myosin IXa [Source:MGI Symbol;Acc:MGI:107735] | 57,94075976 | -0,4303599 | **-1,34756966** | 5,85328686 | 10,3543897 | 0,001291672 | 0,006392611 | protein_coding |
| ENSMUSG00000056612 | Ppp1r14b | protein phosphatase 1, regulatory (inhibitor) subunit 14B [Source:MGI Symbol;Acc:MGI:107682] | 114,6205036 | -0,4306985 | **-1,34788605** | 6,83816752 | 10,1086952 | 0,001475714 | 0,00719648 | protein_coding |
| ENSMUSG00000027248 | Pdia3 | protein disulfide isomerase associated 3 [Source:MGI Symbol;Acc:MGI:95834] | 584,1991318 | -0,4307655 | **-1,3479486** | 9,19038679 | 6,46982669 | 0,010972123 | 0,039165275 | protein_coding |
| ENSMUSG00000042423 | Fbrs | fibrosin [Source:MGI Symbol;Acc:MGI:104648] | 115,32281 | -0,431154 | **-1,34831168** | 6,8467435 | 9,82781635 | 0,001718923 | 0,008257895 | protein_coding |
| ENSMUSG00000025130 | P4hb | prolyl 4-hydroxylase, beta polypeptide [Source:MGI Symbol;Acc:MGI:97464] | 475,4980739 | -0,4312129 | **-1,34836673** | 8,89322072 | 6,56532466 | 0,010398477 | 0,037544549 | protein_coding |
| ENSMUSG00000022858 | Tra2b | transformer 2 beta homolog (Drosophila) [Source:MGI Symbol;Acc:MGI:106016] | 94,01492342 | -0,4314812 | **-1,34861746** | 6,55567991 | 10,6158651 | 0,001121215 | 0,005658607 | protein_coding |
| ENSMUSG00000070343 | Gm10288 | predicted gene 10288 [Source:MGI Symbol;Acc:MGI:3704227] | 219,429066 | -0,4321109 | **-1,34920623** | 7,77693222 | 8,69867708 | 0,003184411 | 0,013878271 | protein_coding |
| ENSMUSG00000002603 | Tgfb1 | transforming growth factor, beta 1 [Source:MGI Symbol;Acc:MGI:98725] | 368,8585296 | -0,4323434 | **-1,34942367** | 8,52643533 | 8,0044905 | 0,004666149 | 0,019098154 | protein_coding |
| ENSMUSG00000059883 | Irak4 | interleukin-1 receptor-associated kinase 4 [Source:MGI Symbol;Acc:MGI:2182474] | 51,46124951 | -0,4327079 | **-1,34976469** | 5,68046809 | 9,72685276 | 0,001815952 | 0,008663422 | protein_coding |
| ENSMUSG00000070394 | 1810027O10Rik | RIKEN cDNA 1810027O10 gene [Source:MGI Symbol;Acc:MGI:1916436] | 53,5267531 | -0,4328381 | **-1,34988653** | 5,74166748 | 9,72364787 | 0,001819121 | 0,008667252 | protein_coding |
| ENSMUSG00000038387 | Rras | Harvey rat sarcoma oncogene, subgroup R [Source:MGI Symbol;Acc:MGI:98179] | 76,51945334 | -0,433064 | **-1,35009785** | 6,25481759 | 8,27797497 | 0,004012886 | 0,016860259 | protein_coding |
| ENSMUSG00000045767 | B230219D22Rik | RIKEN cDNA B230219D22 gene [Source:MGI Symbol;Acc:MGI:1925771] | 87,05464088 | -0,4338315 | **-1,35081635** | 6,44397272 | 11,3792851 | 0,000742677 | 0,003976507 | protein_coding |
| ENSMUSG00000073423 | Zfp414 | zinc finger protein 414 [Source:MGI Symbol;Acc:MGI:1915641] | 19,86441375 | -0,4347304 | **-1,35165819** | 4,30199745 | 6,98125406 | 0,008236787 | 0,030886546 | protein_coding |
| ENSMUSG00000071337 | Tia1 | cytotoxic granule-associated RNA binding protein 1 [Source:MGI Symbol;Acc:MGI:107914] | 34,63489202 | -0,4348631 | **-1,35178255** | 5,10473397 | 9,0130562 | 0,002680578 | 0,011996543 | protein_coding |
| ENSMUSG00000030059 | Tmf1 | TATA element modulatory factor 1 [Source:MGI Symbol;Acc:MGI:2684999] | 82,14328903 | -0,4361097 | **-1,35295107** | 6,35740243 | 11,8822669 | 0,000566709 | 0,00312729 | protein_coding |
| ENSMUSG00000027787 | Nmd3 | NMD3 homolog (S. cerevisiae) [Source:MGI Symbol;Acc:MGI:2140103] | 98,61336415 | -0,4363586 | **-1,35318455** | 6,62286764 | 11,5296061 | 0,000684965 | 0,003698139 | protein_coding |
| ENSMUSG00000062647 | Rpl7a | ribosomal protein L7A [Source:MGI Symbol;Acc:MGI:1353472] | 471,3711551 | -0,4367937 | **-1,3535927** | 8,88028396 | 7,03266576 | 0,008003613 | 0,030185171 | protein_coding |
| ENSMUSG00000021033 | Gstz1 | glutathione transferase zeta 1 (maleylacetoacetate isomerase) [Source:MGI Symbol;Acc:MGI:1341859] | 19,88877751 | -0,4371602 | **-1,35393664** | 4,31442955 | 7,05583891 | 0,00790073 | 0,029850523 | protein_coding |
| ENSMUSG00000032026 | Rexo2 | REX2, RNA exonuclease 2 homolog (S. cerevisiae) [Source:MGI Symbol;Acc:MGI:1888981] | 82,19606685 | -0,4373212 | **-1,35408771** | 6,35934249 | 11,0556234 | 0,000884186 | 0,004617183 | protein_coding |
| ENSMUSG00000070003 | Ssbp4 | single stranded DNA binding protein 4 [Source:MGI Symbol;Acc:MGI:1924150] | 105,8167932 | -0,4378604 | **-1,35459393** | 6,72227753 | 11,26766 | 0,000788691 | 0,004186158 | protein_coding |
| ENSMUSG00000063077 | Kif1b | kinesin family member 1B [Source:MGI Symbol;Acc:MGI:108426] | 143,9767792 | -0,43808 | **-1,35480012** | 7,17118051 | 10,7782899 | 0,001026975 | 0,005252969 | protein_coding |
| ENSMUSG00000045411 | 2410002F23Rik | RIKEN cDNA 2410002F23 gene [Source:MGI Symbol;Acc:MGI:1914226] | 31,74898048 | -0,4384018 | **-1,35510237** | 4,97831747 | 8,39457055 | 0,003763434 | 0,016031859 | protein_coding |
| ENSMUSG00000027900 | Dram2 | DNA-damage regulated autophagy modulator 2 [Source:MGI Symbol;Acc:MGI:1914421] | 133,5429447 | -0,4389057 | **-1,35557574** | 7,06008484 | 11,152305 | 0,000839273 | 0,004403469 | protein_coding |
| ENSMUSG00000022617 | Chkb | choline kinase beta [Source:MGI Symbol;Acc:MGI:1328313] | 54,35076881 | -0,4392131 | **-1,35586459** | 5,75757832 | 9,66355691 | 0,001879593 | 0,008893666 | protein_coding |
| ENSMUSG00000097695 | | | 295,0343698 | -0,4397572 | **-1,35637607** | 8,20390925 | 8,07648443 | 0,004484336 | 0,018473715 | lincRNA |
| ENSMUSG00000040952 | Rps19 | ribosomal protein S19 [Source:MGI Symbol;Acc:MGI:1333780] | 440,4641451 | -0,440088 | **-1,35668709** | 8,78232579 | 7,6282649 | 0,005746056 | 0,022799535 | protein_coding |
| ENSMUSG00000039234 | Sec24d | Sec24 related gene family, member D (S. cerevisiae) [Source:MGI Symbol;Acc:MGI:1916858] | 94,63645735 | -0,4406474 | **-1,35721327** | 6,56612006 | 11,5387003 | 0,000681623 | 0,003683713 | protein_coding |
| ENSMUSG00000057036 | Gm7536 | predicted gene 7536 [Source:MGI Symbol;Acc:MGI:3645137] | 465,6081088 | -0,4406708 | **-1,3572352** | 8,8624885 | 7,26529779 | 0,007029961 | 0,027034716 | protein_coding |
| ENSMUSG00000035545 | Leng8 | leukocyte receptor cluster (LRC) member 8 [Source:MGI Symbol;Acc:MGI:2142195] | 213,0918913 | -0,4418688 | **-1,35836274** | 7,73254034 | 8,62907022 | 0,003308404 | 0,014361689 | protein_coding |
| ENSMUSG00000032812 | Arap1 | ArfGAP with RhoGAP domain, ankyrin repeat and PH domain 1 [Source:MGI Symbol;Acc:MGI:1916960] | 631,4288242 | -0,4426201 | **-1,35907036** | 9,3022247 | 6,53144994 | 0,010598353 | 0,038140879 | protein_coding |
| ENSMUSG00000028745 | Capzb | capping protein (actin filament) muscle Z-line, beta [Source:MGI Symbol;Acc:MGI:104652] | 511,087109 | -0,4427713 | **-1,35921272** | 8,99729124 | 7,08820294 | 0,007759301 | 0,029386917 | protein_coding |
| ENSMUSG00000039725 | 2810408M09Rik | RIKEN cDNA 2810408M09 gene [Source:MGI Symbol;Acc:MGI:1918294] | 100,5113088 | -0,4430869 | **-1,35951013** | 6,65281598 | 12,555857 | 0,000394966 | 0,002284379 | protein_coding |
| ENSMUSG00000027357 | Crls1 | cardiolipin synthase 1 [Source:MGI Symbol;Acc:MGI:1913836] | 28,68165772 | -0,4431616 | **-1,35958057** | 4,83507003 | 8,03735746 | 0,004582233 | 0,018827599 | protein_coding |
| ENSMUSG00000045752 | Tssc4 | tumor-suppressing subchromosomal transferable fragment 4 [Source:MGI Symbol;Acc:MGI:1861712] | 19,41194266 | -0,4445591 | **-1,36089816** | 4,26771136 | 7,24363302 | 0,007115286 | 0,027295967 | protein_coding |
| ENSMUSG00000026395 | Ptprc | protein tyrosine phosphatase, receptor type, C [Source:MGI Symbol;Acc:MGI:97810] | 628,0677518 | -0,4446173 | **-1,3609531** | 9,29464068 | 6,7677521 | 0,009281967 | 0,034153039 | protein_coding |
| ENSMUSG00000041313 | Slc7a1 | solute carrier family 7 (cationic amino acid transporter, y+ system), member 1 [Source:MGI Symbol;Acc:MGI:88117] | 68,7540206 | -0,4454048 | **-1,36169614** | 6,09879642 | 8,36936544 | 0,003815988 | 0,016180786 | protein_coding |
| ENSMUSG00000034007 | Scaper | S phase cyclin A-associated protein in the ER [Source:MGI Symbol;Acc:MGI:1925976] | 19,86731769 | -0,4457138 | **-1,36198785** | 4,31141748 | 7,67840186 | 0,005588564 | 0,022279228 | protein_coding |
| ENSMUSG00000034673 | Pbx2 | pre B cell leukemia homeobox 2 [Source:MGI Symbol;Acc:MGI:1341793] | 76,96898154 | -0,4460083 | **-1,36226583** | 6,26271925 | 9,70708928 | 0,001835585 | 0,008730552 | protein_coding |
| ENSMUSG00000044952 | Kctd21 | potassium channel tetramerisation domain containing 21 [Source:MGI Symbol;Acc:MGI:3643121] | 16,70127666 | -0,446286 | **-1,36252815** | 4,05477765 | 6,55207961 | 0,010476164 | 0,037750612 | protein_coding |
| ENSMUSG00000020107 | Anapc16 | anaphase promoting complex subunit 16 [Source:MGI Symbol;Acc:MGI:1289325] | 36,25759062 | -0,4467303 | **-1,36294776** | 5,17185681 | 9,47477385 | 0,002083166 | 0,009702306 | protein_coding |
| ENSMUSG00000027523 | Gnas | GNAS (guanine nucleotide binding protein, alpha stimulating) complex locus [Source:MGI Symbol;Acc:MGI:95777] | 793,8724213 | -0,4468571 | **-1,36306758** | 9,63256133 | 5,97338176 | 0,014523402 | 0,048977478 | protein_coding |
| ENSMUSG00000024401 | Tnf | tumor necrosis factor [Source:MGI Symbol;Acc:MGI:104798] | 180,4077834 | -0,4469171 | **-1,3631243** | 7,49724402 | 10,5165834 | 0,00118308 | 0,005921869 | protein_coding |
| ENSMUSG00000001065 | Zfp276 | zinc finger protein (C2H2 type) 276 [Source:MGI Symbol;Acc:MGI:1888495] | 32,17245633 | -0,4470324 | **-1,36323323** | 4,99463024 | 8,81936306 | 0,002980507 | 0,013147669 | protein_coding |
| ENSMUSG00000031165 | Was | Wiskott-Aldrich syndrome homolog (human) [Source:MGI Symbol;Acc:MGI:105059] | 90,669966 | -0,4474232 | **-1,36360255** | 6,49866314 | 10,1335168 | 0,001455974 | 0,007113542 | protein_coding |
| ENSMUSG00000033720 | Sfxn5 | sideroflexin 5 [Source:MGI Symbol;Acc:MGI:2137681] | 57,37918024 | -0,4478955 | **-1,36404905** | 5,83968954 | 9,03437549 | 0,002649496 | 0,011876788 | protein_coding |
| ENSMUSG00000040296 | Ddx58 | DEAD (Asp-Glu-Ala-Asp) box polypeptide 58 [Source:MGI Symbol;Acc:MGI:2442858] | 160,0011713 | -0,4482427 | **-1,3643773** | 7,32168136 | 11,500862 | 0,000695639 | 0,003746563 | protein_coding |
| ENSMUSG00000000282 | Mnt | max binding protein [Source:MGI Symbol;Acc:MGI:109150] | 67,43161327 | -0,4486825 | **-1,36479333** | 6,0685163 | 11,0852835 | 0,000870155 | 0,004554738 | protein_coding |
| ENSMUSG00000078853 | Igtp | interferon gamma induced GTPase [Source:MGI Symbol;Acc:MGI:107729] | 76,55026159 | -0,4488133 | **-1,36491705** | 6,25991316 | 10,4952151 | 0,001196841 | 0,005982571 | protein_coding |
| ENSMUSG00000024158 | Hagh | hydroxyacyl glutathione hydrolase [Source:MGI Symbol;Acc:MGI:95745] | 21,48720337 | -0,449082 | **-1,36517131** | 4,41961861 | 8,04741185 | 0,004556871 | 0,018730393 | protein_coding |
| ENSMUSG00000038671 | Arfrp1 | ADP-ribosylation factor related protein 1 [Source:MGI Symbol;Acc:MGI:1923938] | 33,35282023 | -0,4495298 | **-1,36559508** | 5,05669394 | 8,53665503 | 0,003480647 | 0,015008582 | protein_coding |
| ENSMUSG00000074129 | Rpl13a | ribosomal protein L13A [Source:MGI Symbol;Acc:MGI:1351455] | 740,8718118 | -0,4499357 | **-1,36597938** | 9,53272661 | 6,28161184 | 0,012199709 | 0,042755407 | protein_coding |
| ENSMUSG00000018474 | Chd3 | chromodomain helicase DNA binding protein 3 [Source:MGI Symbol;Acc:MGI:1344395] | 264,0403807 | -0,4499699 | **-1,36601178** | 8,0438758 | 9,21366105 | 0,002402158 | 0,010974018 | protein_coding |
| ENSMUSG00000090272 | Mndal | myeloid nuclear differentiation antigen like [Source:MGI Symbol;Acc:MGI:3780953] | 65,20455182 | -0,4499892 | **-1,36603004** | 6,02513978 | 11,1887959 | 0,000822927 | 0,004344787 | protein_coding |
| ENSMUSG00000032557 | Uba5 | ubiquitin-like modifier activating enzyme 5 [Source:MGI Symbol;Acc:MGI:1913913] | 60,73419706 | -0,450351 | **-1,36637262** | 5,92225386 | 9,22287336 | 0,002390102 | 0,010928025 | protein_coding |
| ENSMUSG00000056124 | B4galt6 | UDP-Gal:betaGlcNAc beta 1,4-galactosyltransferase, polypeptide 6 [Source:MGI Symbol;Acc:MGI:1928380] | 304,2760469 | -0,450478 | **-1,36649294** | 8,24924697 | 9,15819019 | 0,002476073 | 0,011241577 | protein_coding |

| | | | | | | | | | | |
|---|---|---|---|---|---|---|---|---|---|---|
| ENSMUSG00000022364 | Wdr67 | WD repeat domain 67 [Source:MGI Symbol;Acc:MGI:2684931] | 33,11974255 | -0,4506008 | **-1,36660925** | 5,03950999 | 9,74953055 | 0,001793685 | 0,008579547 | protein_coding |
| ENSMUSG00000060477 | Irak2 | interleukin-1 receptor-associated kinase 2 [Source:MGI Symbol;Acc:MGI:2429603] | 134,3090028 | -0,4508391 | **-1,36683499** | 7,0700351 | 11,6169529 | 0,000653534 | 0,003545865 | protein_coding |
| ENSMUSG00000024937 | Ehbp1l1 | EH domain binding protein 1-like 1 [Source:MGI Symbol;Acc:MGI:3612340] | 507,6719955 | -0,4515371 | **-1,3674965** | 8,98723447 | 7,07868653 | 0,007800617 | 0,02953321 | protein_coding |
| ENSMUSG00000033068 | Entpd6 | ectonucleoside triphosphate diphosphohydrolase 6 [Source:MGI Symbol;Acc:MGI:1202295] | 30,66926812 | -0,4521829 | **-1,36810874** | 4,93380576 | 9,45121008 | 0,002110098 | 0,00981942 | protein_coding |
| ENSMUSG00000028719 | Cmpk1 | cytidine monophosphate (UMP-CMP) kinase 1 [Source:MGI Symbol;Acc:MGI:1913838] | 179,5066723 | -0,4523938 | **-1,36830875** | 7,48845994 | 11,7303414 | 0,000614893 | 0,00336111 | protein_coding |
| ENSMUSG00000000552 | Zfp385a | zinc finger protein 385A [Source:MGI Symbol;Acc:MGI:1352495] | 83,27853319 | -0,4524575 | **-1,36836918** | 6,37390332 | 12,6774233 | 0,000370097 | 0,002158311 | protein_coding |
| ENSMUSG00000037752 | Xkr8 | X Kell blood group precursor related family member 8 homolog [Source:MGI Symbol;Acc:MGI:2685877] | 16,9755442 | -0,4525444 | **-1,36845159** | 4,08609941 | 6,17048651 | 0,012989888 | 0,04489459 | protein_coding |
| ENSMUSG00000090841 | Myl6 | myosin, light polypeptide 6, alkali, smooth muscle and non-muscle [Source:MGI Symbol;Acc:MGI:109318] | 549,2295102 | -0,4528674 | **-1,36875804** | 9,10112448 | 7,25461242 | 0,007071913 | 0,027158026 | protein_coding |
| ENSMUSG00000041417 | Pik3r1 | phosphatidylinositol 3-kinase, regulatory subunit, polypeptide 1 (p85 alpha) [Source:MGI Symbol;Acc:MGI:97583] | 169,1184768 | -0,453011 | **-1,36889422** | 7,40090909 | 11,2540145 | 0,00079451 | 0,004209367 | protein_coding |
| ENSMUSG00000028382 | Rod1 | ROD1 regulator of differentiation 1 (S. pombe) [Source:MGI Symbol;Acc:MGI:1923334] | 464,3946249 | -0,4531489 | **-1,36902513** | 8,85913538 | 7,407883 | 0,006493869 | 0,025273873 | protein_coding |
| ENSMUSG00000043483 | Gm6863 | predicted gene 6863 [Source:MGI Symbol;Acc:MGI:3647386] | 82,96652313 | -0,4535514 | **-1,36940707** | 6,37319885 | 10,5713385 | 0,001148545 | 0,005772675 | ocessed_pseudoger |
| ENSMUSG00000056131 | Pgm3 | phosphoglucomutase 3 [Source:MGI Symbol;Acc:MGI:97566] | 17,44652427 | -0,4539578 | **-1,36979295** | 4,12025345 | 6,5569657 | 0,010447436 | 0,037694682 | protein_coding |
| ENSMUSG00000021886 | Gpr65 | G-protein coupled receptor 65 [Source:MGI Symbol;Acc:MGI:108031] | 32,56854817 | -0,4544236 | **-1,37023527** | 5,02573249 | 8,87548817 | 0,002890249 | 0,01280967 | protein_coding |
| ENSMUSG00000021936 | Mapk8 | mitogen-activated protein kinase 8 [Source:MGI Symbol;Acc:MGI:1346861] | 17,77924645 | -0,4551706 | **-1,37094496** | 4,15133558 | 7,55077478 | 0,005998428 | 0,023630235 | protein_coding |
| ENSMUSG00000028161 | Ppp3ca | protein phosphatase 3, catalytic subunit, alpha isoform [Source:MGI Symbol;Acc:MGI:107164] | 119,0859803 | -0,4553105 | **-1,37107787** | 6,89332717 | 12,3990163 | 0,00042956 | 0,002453002 | protein_coding |
| ENSMUSG00000047735 | Samd9l | sterile alpha motif domain containing 9-like [Source:MGI Symbol;Acc:MGI:1343184] | 155,8900315 | -0,4555885 | **-1,37134208** | 7,28299321 | 12,0684929 | 0,000512811 | 0,002868767 | protein_coding |
| ENSMUSG00000033909 | Usp36 | ubiquitin specific peptidase 36 [Source:MGI Symbol;Acc:MGI:1919594] | 63,86377762 | -0,4558755 | **-1,37161495** | 5,99395255 | 10,7256006 | 0,001056634 | 0,005382121 | protein_coding |
| ENSMUSG00000040158 | Tax1bp3 | Tax1 (human T cell leukemia virus type I) binding protein 3 [Source:MGI Symbol;Acc:MGI:1923531] | 42,38773268 | -0,4560611 | **-1,37179134** | 5,40525509 | 10,0157164 | 0,0015521 | 0,007532211 | protein_coding |
| ENSMUSG00000073481 | Mosc2 | MOCO sulphurase C-terminal domain containing 2 [Source:MGI Symbol;Acc:MGI:1914497] | 50,69417094 | -0,4562623 | **-1,37198267** | 5,65773618 | 10,6358241 | 0,001109178 | 0,005608166 | protein_coding |
| ENSMUSG00000021000 | Ctage5 | CTAGE family, member 5 [Source:MGI Symbol;Acc:MGI:1346056] | 127,2573635 | -0,4567829 | **-1,37247793** | 6,99021945 | 11,2221934 | 0,000808249 | 0,00427963 | protein_coding |
| ENSMUSG00000039621 | Prex1 | phosphatidylinositol-3,4,5-trisphosphate-dependent Rac exchange factor 1 [Source:MGI Symbol;Acc:MGI:3040696] | 773,9917972 | -0,4575375 | **-1,37319595** | 9,59598957 | 6,22672251 | 0,012583643 | 0,04372438 | protein_coding |
| ENSMUSG00000028452 | Vcp | valosin containing protein [Source:MGI Symbol;Acc:MGI:99919] | 740,2108338 | -0,457553 | **-1,37321072** | 9,53179826 | 6,52902139 | 0,010612833 | 0,038180483 | protein_coding |
| ENSMUSG00000005774 | Rfx5 | regulatory factor X, 5 (influences HLA class II expression) [Source:MGI Symbol;Acc:MGI:1858421] | 18,99715303 | -0,4576753 | **-1,37332716** | 4,23811208 | 6,75585718 | 0,009344045 | 0,034346946 | protein_coding |
| ENSMUSG00000074064 | Mlycd | malonyl-CoA decarboxylase [Source:MGI Symbol;Acc:MGI:1928485] | 18,51305617 | -0,4581249 | **-1,37375512** | 4,19728565 | 7,52922222 | 0,006070611 | 0,023880345 | protein_coding |
| ENSMUSG00000020133 | 2310011J03Rik | RIKEN cDNA 2310011J03 gene [Source:MGI Symbol;Acc:MGI:1913624] | 65,92131922 | -0,4586306 | **-1,37423679** | 6,03687863 | 10,8600751 | 0,000982597 | 0,005063693 | protein_coding |
| ENSMUSG00000029196 | Tada2b | transcriptional adaptor 2B [Source:MGI Symbol;Acc:MGI:3035274] | 21,89416441 | -0,4595753 | **-1,37513697** | 4,44697092 | 7,11483454 | 0,007644866 | 0,028983486 | protein_coding |
| ENSMUSG00000028149 | Rap1gds1 | RAP1, GTP-GDP dissociation stimulator 1 [Source:MGI Symbol;Acc:MGI:2385189] | 213,8384662 | -0,4596244 | **-1,37518376** | 7,7407005 | 10,46242 | 0,001218276 | 0,006081416 | protein_coding |
| ENSMUSG00000030680 | 2900092E17Rik | RIKEN cDNA 2900092E17 gene [Source:MGI Symbol;Acc:MGI:1914528] | 19,01011093 | -0,4597398 | **-1,3752938** | 4,23207863 | 7,18978146 | 0,007331991 | 0,027990474 | protein_coding |
| ENSMUSG00000019849 | Prep | prolyl endopeptidase [Source:MGI Symbol;Acc:MGI:1270863] | 99,65238298 | -0,4600017 | **-1,3755434** | 6,63887056 | 12,2012541 | 0,000477574 | 0,002701908 | protein_coding |
| ENSMUSG00000025007 | Aldh18a1 | aldehyde dehydrogenase 18 family, member A1 [Source:MGI Symbol;Acc:MGI:1888908] | 36,04045882 | -0,4613019 | **-1,37678369** | 5,16221804 | 8,22052521 | 0,004141921 | 0,017309733 | protein_coding |
| ENSMUSG00000024750 | Zfand5 | zinc finger, AN1-type domain 5 [Source:MGI Symbol;Acc:MGI:1278334] | 367,6366097 | -0,4626261 | **-1,37804794** | 8,5221211 | 8,7269212 | 0,003135455 | 0,013719333 | protein_coding |
| ENSMUSG00000086784 | Isoc2a | isochorismatase domain containing 2a [Source:MGI Symbol;Acc:MGI:3609243] | 23,93464479 | -0,4630466 | **-1,37844972** | 4,57717065 | 8,85172904 | 0,002928114 | 0,01293574 | protein_coding |
| ENSMUSG00000037443 | Ccdc21 | coiled-coil domain containing 21 [Source:MGI Symbol;Acc:MGI:1917262] | 37,74437063 | -0,463234 | **-1,3786287** | 5,2350742 | 10,2871194 | 0,001339621 | 0,006609076 | protein_coding |
| ENSMUSG00000060376 | Bckdha | branched chain ketoacid dehydrogenase E1, alpha polypeptide [Source:MGI Symbol;Acc:MGI:107701] | 37,87431664 | -0,4633532 | **-1,37874267** | 5,22916868 | 11,0095257 | 0,000906448 | 0,004719975 | protein_coding |
| ENSMUSG00000038056 | Mll3 | myeloid/lymphoid or mixed-lineage leukemia 3 [Source:MGI Symbol;Acc:MGI:2444959] | 103,0845796 | -0,4633796 | **-1,37876786** | 6,68592928 | 13,6266241 | 0,000223 | 0,001376464 | protein_coding |
| ENSMUSG00000064345 | mt-Nd2 | mitochondrially encoded NADH dehydrogenase 2 [Source:MGI Symbol;Acc:MGI:102500] | 683,1521599 | -0,4636851 | **-1,37905989** | 9,41612422 | 7,12394947 | 0,007606097 | 0,028865439 | protein_coding |
| ENSMUSG00000061838 | Suclg2 | succinate-Coenzyme A ligase, GDP-forming, beta subunit [Source:MGI Symbol;Acc:MGI:1306824] | 26,69015199 | -0,4639187 | **-1,37928321** | 4,73891017 | 9,54566863 | 0,002004218 | 0,009386336 | protein_coding |
| ENSMUSG00000066258 | Trim12a | tripartite motif-containing 12A [Source:MGI Symbol;Acc:MGI:1923931] | 38,61280576 | -0,4642929 | **-1,37964101** | 5,26325902 | 10,8279263 | 0,000999806 | 0,005133114 | protein_coding |
| ENSMUSG00000039048 | Foxred1 | FAD-dependent oxidoreductase domain containing 1 [Source:MGI Symbol;Acc:MGI:2446262] | 29,52231732 | -0,4645773 | **-1,37991303** | 4,87387629 | 8,66288387 | 0,003247569 | 0,014131106 | protein_coding |
| ENSMUSG00000035967 | Ddx26b | DEAD/H (Asp-Glu-Ala-Asp/His) box polypeptide 26B [Source:MGI Symbol;Acc:MGI:2442593] | 89,07822926 | -0,4646145 | **-1,37994856** | 6,47183092 | 12,4276535 | 0,000423023 | 0,002419445 | protein_coding |
| ENSMUSG00000031839 | Hsbp1 | heat shock factor binding protein 1 [Source:MGI Symbol;Acc:MGI:1915446] | 197,7740552 | -0,4670069 | **-1,38223881** | 7,6278142 | 9,4265924 | 0,002138613 | 0,009943691 | protein_coding |
| ENSMUSG00000001175 | Calm1 | calmodulin 1 [Source:MGI Symbol;Acc:MGI:88251] | 881,5939263 | -0,4677523 | **-1,38295319** | 9,78395617 | 6,3452805 | 0,0117694 | 0,041578905 | protein_coding |
| ENSMUSG00000090247 | Bloc1s1 | biogenesis of lysosome-related organelles complex-1, subunit 1 [Source:MGI Symbol;Acc:MGI:1195276] | 30,59543968 | -0,4684822 | **-1,38365299** | 4,92810703 | 7,93920152 | 0,004837518 | 0,019641921 | protein_coding |
| ENSMUSG00000033705 | Stard9 | START domain containing 9 [Source:MGI Symbol;Acc:MGI:3045258] | 165,0108563 | -0,4685551 | **-1,38372295** | 7,36430497 | 12,702086 | 0,000365248 | 0,002133428 | protein_coding |
| ENSMUSG00000021830 | Txndc16 | thioredoxin domain containing 16 [Source:MGI Symbol;Acc:MGI:1917811] | 34,07993782 | -0,4688524 | **-1,3840081** | 5,08464985 | 11,0670727 | 0,000878743 | 0,004593126 | protein_coding |
| ENSMUSG00000059005 | Hnrnpa3 | heterogeneous nuclear ribonucleoprotein A3 [Source:MGI Symbol;Acc:MGI:1917171] | 225,0532206 | -0,4691238 | **-1,38426854** | 7,81336891 | 11,3568661 | 0,000751696 | 0,004017741 | protein_coding |
| ENSMUSG00000063253 | Scoc | short coiled-coil protein [Source:MGI Symbol;Acc:MGI:1927654] | 52,08683025 | -0,4693025 | **-1,38443996** | 5,70173041 | 12,3200136 | 0,000448127 | 0,002547116 | protein_coding |
| ENSMUSG00000032615 | Nt5m | 5',3'-nucleotidase, mitochondrial [Source:MGI Symbol;Acc:MGI:1917127] | 16,91132063 | -0,4700745 | **-1,38518104** | 4,06581949 | 7,07641396 | 0,007810516 | 0,029560504 | protein_coding |
| ENSMUSG00000053063 | Clec12a | C-type lectin domain family 12, member a [Source:MGI Symbol;Acc:MGI:3040968] | 413,4032531 | -0,4701169 | **-1,38522174** | 8,69128113 | 8,27447628 | 0,004020626 | 0,016879869 | protein_coding |
| ENSMUSG00000073079 | Srp54c | signal recognition particle 54C [Source:MGI Symbol;Acc:MGI:3714359] | 43,49240407 | -0,470205 | **-1,38530633** | 5,45434274 | 11,2889971 | 0,000779678 | 0,004146658 | protein_coding |
| ENSMUSG00000092329 | Gm20388 | predicted gene 20388 [Source:MGI Symbol;Acc:MGI:5141853] | 59,02260481 | -0,4705675 | **-1,38565445** | 5,88499597 | 6,19506776 | 0,012810682 | 0,044400935 | protein_coding |
| ENSMUSG00000024982 | Zdhhc6 | zinc finger, DHHC domain containing 6 [Source:MGI Symbol;Acc:MGI:1914230] | 82,49918154 | -0,4715166 | **-1,38656629** | 6,36687761 | 12,2171512 | 0,000473522 | 0,002680363 | protein_coding |
| ENSMUSG00000073609 | D2hgdh | D-2-hydroxyglutarate dehydrogenase [Source:MGI Symbol;Acc:MGI:2138209] | 17,62577281 | -0,4716354 | **-1,38668044** | 4,12982559 | 7,84556098 | 0,005094565 | 0,020571107 | protein_coding |
| ENSMUSG00000029033 | Acap3 | ArfGAP with coiled-coil, ankyrin repeat and PH domains 3 [Source:MGI Symbol;Acc:MGI:2153589] | 22,04166007 | -0,4719221 | **-1,38695607** | 4,4526905 | 8,94345416 | 0,002784656 | 0,012401711 | protein_coding |
| ENSMUSG00000028568 | Btf3l4 | basic transcription factor 3-like 4 [Source:MGI Symbol;Acc:MGI:1915312] | 42,91670107 | -0,472017 | **-1,38704732** | 5,41825013 | 11,1853997 | 0,000824435 | 0,004348645 | protein_coding |
| ENSMUSG00000049734 | Trex1 | three prime repair exonuclease 1 [Source:MGI Symbol;Acc:MGI:1328317] | 73,00645911 | -0,4720523 | **-1,38708121** | 6,18776281 | 11,6553313 | 0,000640188 | 0,003483777 | protein_coding |
| ENSMUSG00000020770 | Unk | unkempt homolog (Drosophila) [Source:MGI Symbol;Acc:MGI:2442456] | 29,76430257 | -0,4720565 | **-1,38708531** | 4,88737009 | 10,7193277 | 0,001060222 | 0,005393734 | protein_coding |
| ENSMUSG00000034709 | Klraq1 | KLRAQ motif containing 1 [Source:MGI Symbol;Acc:MGI:1921075] | 85,462878 | -0,4720753 | **-1,38710332** | 6,41727805 | 11,82769 | 0,000583563 | 0,00321223 | protein_coding |
| ENSMUSG00000026737 | Pip4k2a | phosphatidylinositol-5-phosphate 4-kinase, type II, alpha [Source:MGI Symbol;Acc:MGI:1298206] | 195,0564445 | -0,4722275 | **-1,38724973** | 7,60639614 | 10,8193315 | 0,001004457 | 0,005154589 | protein_coding |
| ENSMUSG00000015733 | Capza2 | capping protein (actin filament) muscle Z-line, alpha 2 [Source:MGI Symbol;Acc:MGI:106222] | 655,6448602 | -0,4725961 | **-1,38760415** | 9,35671341 | 7,17943597 | 0,007374389 | 0,028113258 | protein_coding |
| ENSMUSG00000038368 | BC057079 | cDNA sequence BC057079 [Source:MGI Symbol;Acc:MGI:2676921] | 20,60389315 | -0,4727732 | **-1,38777452** | 4,35855896 | 8,81913939 | 0,002980872 | 0,013147669 | protein_coding |
| ENSMUSG00000061665 | Cd2ap | CD2-associated protein [Source:MGI Symbol;Acc:MGI:1330281] | 156,5722036 | -0,4727799 | **-1,38778102** | 7,29069814 | 10,8096909 | 0,001009702 | 0,005176401 | protein_coding |
| ENSMUSG00000032508 | Myd88 | myeloid differentiation primary response gene 88 [Source:MGI Symbol;Acc:MGI:108005] | 124,9024691 | -0,4728489 | **-1,38784738** | 6,96328462 | 9,14058772 | 0,002500008 | 0,011331512 | protein_coding |
| ENSMUSG00000014554 | Dguok | deoxyguanosine kinase [Source:MGI Symbol;Acc:MGI:1351602] | 20,07578933 | -0,4728779 | **-1,38787521** | 4,30997104 | 6,62827935 | 0,010037218 | 0,036528291 | protein_coding |
| ENSMUSG00000035048 | Anapc13 | anaphase promoting complex subunit 13 [Source:MGI Symbol;Acc:MGI:1916260] | 14,86992282 | -0,4740246 | **-1,38897884** | 3,8863829 | 6,77090868 | 0,009265565 | 0,034104107 | protein_coding |
| ENSMUSG00000003316 | Glg1 | golgi apparatus protein 1 [Source:MGI Symbol;Acc:MGI:104967] | 623,9072989 | -0,4742208 | **-1,38916772** | 9,28513321 | 7,88392626 | 0,004987608 | 0,020183739 | protein_coding |
| ENSMUSG00000021893 | Capn7 | calpain 7 [Source:MGI Symbol;Acc:MGI:1338030] | 73,05444289 | -0,4743228 | **-1,38926599** | 6,19048065 | 13,014604 | 0,000309071 | 0,001833566 | protein_coding |
| ENSMUSG00000056228 | Cars2 | cysteinyl-tRNA synthetase 2 (mitochondrial)(putative) [Source:MGI Symbol;Acc:MGI:1919191] | 13,71720442 | -0,4744882 | **-1,38942526** | 3,76127485 | 6,04624789 | 0,01393588 | 0,04740327 | protein_coding |
| ENSMUSG00000034088 | Hdlbp | high density lipoprotein (HDL) binding protein [Source:MGI Symbol;Acc:MGI:99256] | 419,0437622 | -0,4748473 | **-1,38977111** | 8,71085461 | 8,40103171 | 0,003750081 | 0,015982212 | protein_coding |
| ENSMUSG00000034595 | Ppp1r18 | protein phosphatase 1, regulatory subunit 18 [Source:MGI Symbol;Acc:MGI:1923698] | 251,9949169 | -0,4753471 | **-1,39025265** | 7,97619773 | 9,34086581 | 0,002240996 | 0,010327947 | protein_coding |
| ENSMUSG00000028863 | Meaf6 | MYST/Esa1-associated factor 6 [Source:MGI Symbol;Acc:MGI:1917338] | 21,39963395 | -0,4753673 | **-1,39027214** | 4,41133896 | 7,30334178 | 0,006882648 | 0,026551846 | protein_coding |

| | | | | | | | | | | |
|---|---|---|---|---|---|---|---|---|---|---|
| ENSMUSG00000090877 | Hspa1b | heat shock protein 1B [Source:MGI Symbol;Acc:MGI:99517] | 18,17094365 | -0,4753883 | **-1,39029241** | 4,22476043 | 6,29152105 | 0,01213169 | 0,04255775 | protein_coding |
| ENSMUSG00000041084 | Ostc | oligosaccharyltransferase complex subunit [Source:MGI Symbol;Acc:MGI:1913607] | 59,93107348 | -0,4756884 | **-1,39058158** | 5,90599156 | 11,9922103 | 0,000534234 | 0,002975718 | protein_coding |
| ENSMUSG00000020553 | Pctp | phosphatidylcholine transfer protein [Source:MGI Symbol;Acc:MGI:107375] | 52,69480583 | -0,4768822 | **-1,39173272** | 5,71810082 | 12,7210334 | 0,000361566 | 0,00211417 | protein_coding |
| ENSMUSG00000071041 | Gm15210 | predicted gene 15210 [Source:MGI Symbol;Acc:MGI:3705743] | 26,49847178 | -0,477365 | **-1,39219854** | 4,72091313 | 9,64698224 | 0,001896629 | 0,008939624 | pseudogene |
| ENSMUSG00000028467 | Gba2 | glucosidase beta 2 [Source:MGI Symbol;Acc:MGI:2654325] | 24,99687077 | -0,4777048 | **-1,39252652** | 4,62735704 | 8,21903255 | 0,004145329 | 0,017317389 | protein_coding |
| ENSMUSG00000022982 | Sod1 | superoxide dismutase 1, soluble [Source:MGI Symbol;Acc:MGI:98351] | 195,3670776 | -0,4782788 | **-1,39308068** | 7,60952386 | 12,4442835 | 0,000419273 | 0,002400497 | protein_coding |
| ENSMUSG00000095677 | Dynlt1f | dynein light chain Tctex-type 1F [Source:MGI Symbol;Acc:MGI:3780996] | 37,96096929 | -0,4784263 | **-1,39322311** | 5,25029049 | 11,1575119 | 0,000836921 | 0,004394804 | protein_coding |
| ENSMUSG00000029575 | Mmab | methylmalonic aciduria (cobalamin deficiency) type B homolog (human) [Source:MGI Symbol;Acc:MGI:1924947] | 16,72959177 | -0,4796097 | **-1,39436643** | 4,04864615 | 6,81820938 | 0,0090233 | 0,033391375 | protein_coding |
| ENSMUSG00000028868 | Wasf2 | WAS protein family, member 2 [Source:MGI Symbol;Acc:MGI:1098641] | 387,1733547 | -0,4796504 | **-1,39440577** | 8,59646706 | 9,17491662 | 0,002453545 | 0,011153125 | protein_coding |
| ENSMUSG00000086583 | Gm15500 | predicted pseudogene 15500 [Source:MGI Symbol;Acc:MGI:3782947] | 623,8122435 | -0,4811172 | **-1,39582411** | 9,28479636 | 7,70402412 | 0,005509785 | 0,02201265 | pseudogene |
| ENSMUSG00000055805 | Fmnl1 | formin-like 1 [Source:MGI Symbol;Acc:MGI:1888994] | 224,6937695 | -0,4812465 | **-1,3959493** | 7,8100949 | 10,6426385 | 0,001105099 | 0,005592687 | protein_coding |
| ENSMUSG00000044465 | Fam160a2 | family with sequence similarity 160, member A2 [Source:MGI Symbol;Acc:MGI:1921599] | 29,19521201 | -0,4812709 | **-1,39597285** | 4,86133041 | 10,6729295 | 0,001087148 | 0,005511996 | protein_coding |
| ENSMUSG00000030275 | Etnk1 | ethanolamine kinase 1 [Source:MGI Symbol;Acc:MGI:1922570] | 137,666717 | -0,4816174 | **-1,39630815** | 7,10350964 | 13,302671 | 0,000265028 | 0,00160345 | protein_coding |
| ENSMUSG00000036565 | Ttyh3 | tweety homolog 3 (Drosophila) [Source:MGI Symbol;Acc:MGI:1925589] | 139,725975 | -0,4827403 | **-1,39739544** | 7,12372067 | 11,6970432 | 0,000625995 | 0,003414998 | protein_coding |
| ENSMUSG00000023030 | Slc11a2 | solute carrier family 11 (proton-coupled divalent metal ion transporters), member 2 [Source:MGI Symbol;Acc:MGI:13452 | 334,8334027 | -0,4837982 | **-1,39842042** | 8,38721245 | 10,1061595 | 0,001477746 | 0,007199997 | protein_coding |
| ENSMUSG00000021285 | Ppp1r13b | protein phosphatase 1, regulatory (inhibitor) subunit 13B [Source:MGI Symbol;Acc:MGI:1336199] | 46,61878134 | -0,4838618 | **-1,39848207** | 5,5417276 | 11,1835862 | 0,000825241 | 0,004348645 | protein_coding |
| ENSMUSG00000023022 | Lima1 | LIM domain and actin binding 1 [Source:MGI Symbol;Acc:MGI:1920992] | 228,8946593 | -0,4838737 | **-1,39849367** | 7,83881496 | 12,1893457 | 0,000480632 | 0,002712735 | protein_coding |
| ENSMUSG00000030727 | Rabep2 | rabaptin, RAB GTPase binding effector protein 2 [Source:MGI Symbol;Acc:MGI:1917564] | 32,82245951 | -0,4839493 | **-1,39856696** | 5,02284324 | 10,9019889 | 0,000960611 | 0,004959694 | protein_coding |
| ENSMUSG00000046230 | Vps13a | vacuolar protein sorting 13A (yeast) [Source:MGI Symbol;Acc:MGI:2444304] | 59,39215084 | -0,4840805 | **-1,39869414** | 5,88763463 | 13,5246862 | 0,000235446 | 0,001444356 | protein_coding |
| ENSMUSG00000028556 | Dock7 | dedicator of cytokinesis 7 [Source:MGI Symbol;Acc:MGI:1914549] | 132,5690361 | -0,4855391 | **-1,40010891** | 7,05063697 | 13,6604085 | 0,000219024 | 0,001356245 | protein_coding |
| ENSMUSG00000032399 | Rpl4 | ribosomal protein L4 [Source:MGI Symbol;Acc:MGI:1915141] | 1171,074289 | -0,4866246 | **-1,4011628** | 10,193506 | 6,14568751 | 0,013173294 | 0,04535725 | protein_coding |
| ENSMUSG00000041164 | Zmiz2 | zinc finger, MIZ-type containing 2 [Source:MGI Symbol;Acc:MGI:106374] | 184,3818914 | -0,4869916 | **-1,40151931** | 7,52464674 | 11,8223386 | 0,000585243 | 0,003218251 | protein_coding |
| ENSMUSG00000007850 | Hnrnph1 | heterogeneous nuclear ribonucleoprotein H1 [Source:MGI Symbol;Acc:MGI:1891925] | 215,1805179 | -0,4883207 | **-1,40281109** | 7,74812381 | 12,0170632 | 0,000527157 | 0,002943025 | protein_coding |
| ENSMUSG00000026942 | Traf2 | TNF receptor-associated factor 2 [Source:MGI Symbol;Acc:MGI:101835] | 44,78143827 | -0,4883424 | **-1,4028321** | 5,48250638 | 11,5524281 | 0,000676608 | 0,003662018 | protein_coding |
| ENSMUSG00000034118 | Tpst1 | protein-tyrosine sulfotransferase 1 [Source:MGI Symbol;Acc:MGI:1298231] | 15,95529969 | -0,4884555 | **-1,40294211** | 4,00031758 | 8,23086767 | 0,004118384 | 0,017231028 | protein_coding |
| ENSMUSG00000039656 | Rxrb | retinoid X receptor beta [Source:MGI Symbol;Acc:MGI:98215] | 46,20836584 | -0,4888395 | **-1,40331558** | 5,52147594 | 10,3180815 | 0,001317332 | 0,00650347 | protein_coding |
| ENSMUSG00000055435 | Maf | avian musculoaponeurotic fibrosarcoma (v-maf) AS42 oncogene homolog [Source:MGI Symbol;Acc:MGI:96909] | 695,9688949 | -0,4890656 | **-1,40353551** | 9,44271685 | 7,01751586 | 0,008071614 | 0,030350041 | protein_coding |
| ENSMUSG00000046245 | Pilra | paired immunoglobin-like type 2 receptor alpha [Source:MGI Symbol;Acc:MGI:2450529] | 64,3967816 | -0,4891364 | **-1,40360444** | 6,01139531 | 8,52272898 | 0,003507382 | 0,015094242 | protein_coding |
| ENSMUSG00000038291 | Snx25 | sorting nexin 25 [Source:MGI Symbol;Acc:MGI:2142610] | 25,59052591 | -0,4892669 | **-1,40373136** | 4,67953636 | 10,5522164 | 0,001160488 | 0,005828447 | protein_coding |
| ENSMUSG00000018008 | Cyth4 | cytohesin 4 [Source:MGI Symbol;Acc:MGI:2441702] | 934,5783313 | -0,489398 | **-1,40385897** | 9,86802039 | 6,49175921 | 0,010837568 | 0,038785784 | protein_coding |
| ENSMUSG00000020372 | Gnb2l1 | guanine nucleotide binding protein (G protein), beta polypeptide 2 like 1 [Source:MGI Symbol;Acc:MGI:101849] | 953,6859418 | -0,4895512 | **-1,40400808** | 9,89719783 | 6,65826678 | 0,009869686 | 0,036005494 | protein_coding |
| ENSMUSG00000025935 | Tram1 | translocating chain-associating membrane protein 1 [Source:MGI Symbol;Acc:MGI:1919515] | 168,5681581 | -0,4897137 | **-1,40416622** | 7,39632584 | 13,3741649 | 0,000255114 | 0,001552875 | protein_coding |
| ENSMUSG00000014177 | Fam18b | family with sequence similarity 18, member B [Source:MGI Symbol;Acc:MGI:1914760] | 25,21288693 | -0,4908395 | **-1,40526231** | 4,64847728 | 8,71725154 | 0,003152129 | 0,013770354 | protein_coding |
| ENSMUSG00000006304 | Arpc2 | actin related protein 2/3 complex, subunit 2 [Source:MGI Symbol;Acc:MGI:1923959] | 959,9555852 | -0,4908564 | **-1,40527881** | 9,90676143 | 6,74244967 | 0,009414526 | 0,034582882 | protein_coding |
| ENSMUSG00000068874 | Selenbp1 | selenium binding protein 1 [Source:MGI Symbol;Acc:MGI:96825] | 17,7918025 | -0,4910455 | **-1,40546303** | 4,14293619 | 9,13684354 | 0,002505129 | 0,01134417 | protein_coding |
| ENSMUSG00000035696 | Rnf38 | ring finger protein 38 [Source:MGI Symbol;Acc:MGI:1920719] | 67,95965411 | -0,4912293 | **-1,40564207** | 6,08482696 | 13,6596904 | 0,000219108 | 0,001356245 | protein_coding |
| ENSMUSG00000032870 | Smap2 | stromal membrane-associated GTPase-activating protein 2 [Source:MGI Symbol;Acc:MGI:1917030] | 120,8401631 | -0,4916743 | **-1,40607577** | 6,91486106 | 13,2916666 | 0,000266588 | 0,001611115 | protein_coding |
| ENSMUSG00000000386 | Mx1 | myxovirus (influenza virus) resistance 1 [Source:MGI Symbol;Acc:MGI:97243] | 38,40475908 | -0,4929383 | **-1,40730819** | 5,2731556 | 11,4902955 | 0,000699605 | 0,003766076 | protein_coding |
| ENSMUSG00000025915 | Sgk3 | serum/glucocorticoid regulated kinase 3 [Source:MGI Symbol;Acc:MGI:2182368] | 54,77513698 | -0,4931693 | **-1,40753352** | 5,77514166 | 13,1279699 | 0,00029092 | 0,001740924 | protein_coding |
| ENSMUSG00000025241 | Fyco1 | FYVE and coiled-coil domain containing 1 [Source:MGI Symbol;Acc:MGI:107277] | 85,09475128 | -0,4932482 | **-1,40761053** | 6,40874533 | 14,4352716 | 0,00014506 | 0,000946421 | protein_coding |
| ENSMUSG00000045932 | Ifit2 | interferon-induced protein with tetratricopeptide repeats 2 [Source:MGI Symbol;Acc:MGI:99449] | 78,25650819 | -0,4934532 | **-1,40781058** | 6,29713113 | 14,7548697 | 0,000122431 | 0,000815241 | protein_coding |
| ENSMUSG00000020101 | 4632428N05Rik | RIKEN cDNA 4632428N05 gene [Source:MGI Symbol;Acc:MGI:1921298] | 538,0603231 | -0,4938794 | **-1,40822651** | 9,07139725 | 7,96482724 | 0,004769504 | 0,019437145 | protein_coding |
| ENSMUSG00000027236 | Eif3j1 | eukaryotic translation initiation factor 3, subunit J1 [Source:MGI Symbol;Acc:MGI:1925905] | 30,85344774 | -0,4938802 | **-1,40822727** | 4,94902482 | 11,630083 | 0,000648937 | 0,003526145 | protein_coding |
| ENSMUSG00000030979 | Uros | uroporphyrinogen III synthase [Source:MGI Symbol;Acc:MGI:98917] | 12,33686313 | -0,4939667 | **-1,40831171** | 3,61360685 | 6,03631461 | 0,01401451 | 0,04759735 | protein_coding |
| ENSMUSG00000051768 | Xrcc1 | X-ray repair complementing defective repair in Chinese hamster cells 1 [Source:MGI Symbol;Acc:MGI:99137] | 39,45609068 | -0,4940851 | **-1,40842734** | 5,29523601 | 12,4835133 | 0,000410559 | 0,00235798 | protein_coding |
| ENSMUSG00000056629 | Fkbp2 | FK506 binding protein 2 [Source:MGI Symbol;Acc:MGI:95542] | 32,93662438 | -0,4944595 | **-1,40879285** | 5,0339208 | 11,923744 | 0,00055423 | 0,003064581 | protein_coding |
| ENSMUSG00000038623 | Tm6sf1 | transmembrane 6 superfamily member 1 [Source:MGI Symbol;Acc:MGI:1933209] | 125,0778937 | -0,494924 | **-1,40924649** | 6,96599549 | 13,7744762 | 0,000206118 | 0,001286713 | protein_coding |
| ENSMUSG00000055897 | Ppp4r1l-ps | protein phosphatase 4, regulatory subunit 1-like, pseudogene [Source:MGI Symbol;Acc:MGI:1924560] | 22,37686727 | -0,4951649 | **-1,40948189** | 4,46674841 | 8,81960752 | 0,002980108 | 0,013147669 | pseudogene |
| ENSMUSG00000004070 | Hmox2 | heme oxygenase (decycling) 2 [Source:MGI Symbol;Acc:MGI:109373] | 161,9307721 | -0,4954228 | **-1,40973383** | 7,33828034 | 13,385207 | 0,000253617 | 0,001547188 | protein_coding |
| ENSMUSG00000043998 | Mgat2 | mannoside acetylglucosaminyltransferase 2 [Source:MGI Symbol;Acc:MGI:2384966] | 91,07771303 | -0,4955553 | **-1,4098633** | 6,50777885 | 12,2688729 | 0,000460576 | 0,002612469 | protein_coding |
| ENSMUSG00000055541 | Lair1 | leukocyte-associated Ig-like receptor 1 [Source:MGI Symbol;Acc:MGI:105492] | 108,5932015 | -0,4957531 | **-1,41005659** | 6,76069629 | 15,3584655 | 8,89216E-05 | 0,000616004 | protein_coding |
| ENSMUSG00000038058 | Nod1 | nucleotide-binding oligomerization domain containing 1 [Source:MGI Symbol;Acc:MGI:1341839] | 29,08968852 | -0,4964857 | **-1,4107728** | 4,87791454 | 11,3334255 | 0,000761244 | 0,004064034 | protein_coding |
| ENSMUSG00000048865 | Arhgap30 | Rho GTPase activating protein 30 [Source:MGI Symbol;Acc:MGI:2684948] | 438,1281124 | -0,4970369 | **-1,41131194** | 8,77483027 | 9,13552847 | 0,00250693 | 0,01134417 | protein_coding |
| ENSMUSG00000027472 | Pdrg1 | p53 and DNA damage regulated 1 [Source:MGI Symbol;Acc:MGI:1915809] | 61,67195567 | -0,4977988 | **-1,41205748** | 5,94359544 | 12,9789418 | 0,000315014 | 0,0018658 | protein_coding |
| ENSMUSG00000004451 | Ralb | v-ral simian leukemia viral oncogene homolog B (ras related) [Source:MGI Symbol;Acc:MGI:1927244] | 101,1937766 | -0,4981369 | **-1,4123884** | 6,65923478 | 13,7919538 | 0,000204209 | 0,001276411 | protein_coding |
| ENSMUSG00000004446 | Bid | BH3 interacting domain death agonist [Source:MGI Symbol;Acc:MGI:108093] | 60,27821268 | -0,4983085 | **-1,41255639** | 5,91112826 | 12,2785741 | 0,000458188 | 0,00260161 | protein_coding |
| ENSMUSG00000030681 | Mvp | major vault protein [Source:MGI Symbol;Acc:MGI:1925638] | 372,6316249 | -0,4983425 | **-1,41258971** | 8,54141446 | 10,0179445 | 0,001550224 | 0,007526429 | protein_coding |
| ENSMUSG00000024642 | Tle4 | transducin-like enhancer of split 4, homolog of Drosophila E(spl) [Source:MGI Symbol;Acc:MGI:104633] | 98,42978458 | -0,4983693 | **-1,41261595** | 6,62105611 | 14,0704853 | 0,000176085 | 0,001120237 | protein_coding |
| ENSMUSG00000021252 | 0610007P14Rik | RIKEN cDNA 0610007P14 gene [Source:MGI Symbol;Acc:MGI:1915571] | 28,8099619 | -0,4992722 | **-1,4135003** | 4,83880537 | 6,97689368 | 0,00825688 | 0,030951329 | protein_coding |
| ENSMUSG00000041992 | Rapgef5 | Rap guanine nucleotide exchange factor (GEF) 5 [Source:MGI Symbol;Acc:MGI:2444365] | 228,754809 | -0,4996081 | **-1,41382946** | 7,83775018 | 12,190334 | 0,000480378 | 0,002712735 | protein_coding |
| ENSMUSG00000050786 | Ccdc126 | coiled-coil domain containing 126 [Source:MGI Symbol;Acc:MGI:1889376] | 13,29912015 | -0,4998083 | **-1,41402567** | 3,71754398 | 6,04928984 | 0,013911891 | 0,047336621 | protein_coding |
| ENSMUSG00000008475 | Arpc5 | actin related protein 2/3 complex, subunit 5 [Source:MGI Symbol;Acc:MGI:1915021] | 775,4649963 | -0,5002174 | **-1,41442667** | 9,59888469 | 7,56212126 | 0,005960779 | 0,023532547 | protein_coding |
| ENSMUSG00000000823 | Znf512b | zinc finger protein 512B [Source:MGI Symbol;Acc:MGI:2685478] | 44,02048385 | -0,500451 | **-1,41465577** | 5,45077569 | 11,8249389 | 0,000584426 | 0,003215369 | protein_coding |
| ENSMUSG00000033326 | Kdm4a | lysine (K)-specific demethylase 4A [Source:MGI Symbol;Acc:MGI:2446210] | 109,7659168 | -0,5005536 | **-1,41475629** | 6,7747041 | 13,8062114 | 0,000202665 | 0,001268042 | protein_coding |
| ENSMUSG00000024038 | Ndufv3 | NADH dehydrogenase (ubiquinone) flavoprotein 3 [Source:MGI Symbol;Acc:MGI:1890894] | 54,68068851 | -0,5010689 | **-1,41526176** | 5,76997817 | 13,6237009 | 0,000223348 | 0,001377835 | protein_coding |
| ENSMUSG00000018750 | Zbtb4 | zinc finger and BTB domain containing 4 [Source:MGI Symbol;Acc:MGI:1922830] | 127,6415661 | -0,5013326 | **-1,41552041** | 6,99422194 | 14,6749649 | 0,000127732 | 0,000846434 | protein_coding |
| ENSMUSG00000022265 | Ank | progressive ankylosis [Source:MGI Symbol;Acc:MGI:3045421] | 54,76106729 | -0,501618 | **-1,41580052** | 5,7662531 | 13,6373401 | 0,000221731 | 0,00137017 | protein_coding |
| ENSMUSG00000019054 | Fis1 | fission 1 (mitochondrial outer membrane) homolog (yeast) [Source:MGI Symbol;Acc:MGI:1913687] | 81,66040713 | -0,5018493 | **-1,41602752** | 6,34662446 | 14,7109534 | 0,000125316 | 0,00083294 | protein_coding |
| ENSMUSG00000066315 | Gm12918 | predicted gene 12918 [Source:MGI Symbol;Acc:MGI:3652005] | 156,3506158 | -0,5018755 | **-1,41605326** | 7,28770131 | 10,0310644 | 0,001539221 | 0,007476312 | pseudogene |
| ENSMUSG00000040659 | Efhd2 | EF hand domain containing 2 [Source:MGI Symbol;Acc:MGI:106504] | 1089,337277 | -0,5019698 | **-1,41614576** | 10,0890956 | 6,42885952 | 0,011228065 | 0,039962019 | protein_coding |
| ENSMUSG00000002668 | Dennd1c | DENN/MADD domain containing 1C [Source:MGI Symbol;Acc:MGI:1918035] | 63,40467528 | -0,5022407 | **-1,4164117** | 5,97956358 | 13,0682469 | 0,000300344 | 0,001788553 | protein_coding |

| | | | | | | | | | | |
|---|---|---|---|---|---|---|---|---|---|---|
| ENSMUSG00000043716 | Rpl7 | ribosomal protein L7 [Source:MGI Symbol;Acc:MGI:98073] | 569,2593234 | -0,5023511 | **-1,41652015** | 9,15268927 | 8,06015123 | 0,004524941 | 0,018620046 | protein_coding |
| ENSMUSG00000029434 | Vps33a | vacuolar protein sorting 33A (yeast) [Source:MGI Symbol;Acc:MGI:1924823] | 178,7142484 | -0,5029491 | **-1,41710735** | 7,48089269 | 14,1137542 | 0,000172081 | 0,001096031 | protein_coding |
| ENSMUSG00000017561 | Crlf3 | cytokine receptor-like factor 3 [Source:MGI Symbol;Acc:MGI:1860086] | 98,52185363 | -0,5032313 | **-1,41738462** | 6,62346094 | 14,5856595 | 0,00013393 | 0,000881658 | protein_coding |
| ENSMUSG00000034801 | Sos2 | son of sevenless homolog 2 (Drosophila) [Source:MGI Symbol;Acc:MGI:98355] | 75,17127734 | -0,5044893 | **-1,41862113** | 6,23065014 | 13,8557349 | 0,000197393 | 0,001237171 | protein_coding |
| ENSMUSG00000032570 | Atp2c1 | ATPase, Ca++-sequestering [Source:MGI Symbol;Acc:MGI:1889008] | 132,1933603 | -0,5061795 | **-1,4202841** | 7,04701517 | 14,0163468 | 0,000181228 | 0,001149627 | protein_coding |
| ENSMUSG00000020460 | Rps27a | ribosomal protein S27A [Source:MGI Symbol;Acc:MGI:1925544] | 334,5609956 | -0,5062338 | **-1,42033751** | 8,38566542 | 10,1063792 | 0,00147757 | 0,007199997 | protein_coding |
| ENSMUSG00000029171 | Pgm1 | phosphoglucomutase 1 [Source:MGI Symbol;Acc:MGI:97564] | 48,99467617 | -0,5072434 | **-1,42133181** | 5,61247451 | 13,0883977 | 0,00029713 | 0,001772297 | protein_coding |
| ENSMUSG00000051557 | Pusl1 | pseudouridylate synthase-like 1 [Source:MGI Symbol;Acc:MGI:3047787] | 12,55433855 | -0,5074579 | **-1,42154319** | 3,62953053 | 6,43251668 | 0,01120497 | 0,039905675 | protein_coding |
| ENSMUSG00000034330 | Plcg2 | phospholipase C, gamma 2 [Source:MGI Symbol;Acc:MGI:97616] | 246,9912293 | -0,5075107 | **-1,42159515** | 7,94729473 | 11,9051497 | 0,00055979 | 0,003092212 | protein_coding |
| ENSMUSG00000030447 | Cyfip1 | cytoplasmic FMR1 interacting protein 1 [Source:MGI Symbol;Acc:MGI:1338801] | 974,2476623 | -0,5077027 | **-1,42178443** | 9,92821583 | 7,25846056 | 0,007056776 | 0,027109369 | protein_coding |
| ENSMUSG00000060803 | Gstp1 | glutathione S-transferase, pi 1 [Source:MGI Symbol;Acc:MGI:95865] | 22,24669136 | -0,5079238 | **-1,42200234** | 4,47258494 | 9,29826139 | 0,002293714 | 0,010528402 | protein_coding |
| ENSMUSG00000097830 | | | 22,24669136 | -0,5079245 | **-1,42200296** | 4,47258503 | 9,29797393 | 0,002294074 | 0,010528402 | protein_coding |
| ENSMUSG00000059447 | Hadhb | hydroxyacyl-Coenzyme A dehydrogenase/3-ketoacyl-Coenzyme A thiolase/enoyl-Coenzyme A hydratase (trifunctional pr | 75,51841525 | -0,5081432 | **-1,42221855** | 6,23911087 | 15,3666565 | 8,85369E-05 | 0,000613726 | protein_coding |
| ENSMUSG00000028163 | Nfkb1 | nuclear factor of kappa light polypeptide gene enhancer in B cells 1, p105 [Source:MGI Symbol;Acc:MGI:97312] | 138,6976745 | -0,5082177 | **-1,42229198** | 7,11589913 | 13,3652915 | 0,000256324 | 0,001556031 | protein_coding |
| ENSMUSG00000028669 | Pithd1 | PITH (C-terminal proteasome-interacting domain of thioredoxin-like) domain containing 1 [Source:MGI Symbol;Acc:MGI: | 19,38756768 | -0,5082725 | **-1,42234607** | 4,26706554 | 8,3935784 | 0,003765489 | 0,016031859 | protein_coding |
| ENSMUSG00000028573 | Fggy | FGGY carbohydrate kinase domain containing [Source:MGI Symbol;Acc:MGI:1922828] | 17,3977291 | -0,5083466 | **-1,42241912** | 4,12002435 | 9,68690553 | 0,001855858 | 0,008804108 | protein_coding |
| ENSMUSG00000053332 | Gas5 | growth arrest specific 5 [Source:MGI Symbol;Acc:MGI:95659] | 97,51913315 | -0,5084305 | **-1,42250178** | 6,60412444 | 14,5244523 | 0,000138352 | 0,000906425 | non_coding |
| ENSMUSG00000087107 | AI662270 | expressed sequence AI662270 [Source:MGI Symbol;Acc:MGI:2144254] | 83,75413538 | -0,5088131 | **-1,42287907** | 6,38713715 | 14,8409063 | 0,000116971 | 0,000782678 | lincRNA |
| ENSMUSG00000058835 | Abi1 | abl-interactor 1 [Source:MGI Symbol;Acc:MGI:104913] | 367,2126963 | -0,5093006 | **-1,42336002** | 8,52024132 | 10,6077008 | 0,001126176 | 0,005675826 | protein_coding |
| ENSMUSG00000021816 | Ppp3cb | protein phosphatase 3, catalytic subunit, beta isoform [Source:MGI Symbol;Acc:MGI:107163] | 82,79697957 | -0,5097818 | **-1,42383485** | 6,37064589 | 14,7096586 | 0,000125402 | 0,000833008 | protein_coding |
| ENSMUSG00000025794 | Rpl14 | ribosomal protein L14 [Source:MGI Symbol;Acc:MGI:1914365] | 398,1453647 | -0,5108906 | **-1,42492955** | 8,63640172 | 9,70125809 | 0,001841419 | 0,008754509 | protein_coding |
| ENSMUSG00000022680 | Pdxdc1 | pyridoxal-dependent decarboxylase domain containing 1 [Source:MGI Symbol;Acc:MGI:1920909] | 158,4858134 | -0,511271 | **-1,42530533** | 7,30743477 | 14,6021762 | 0,000132761 | 0,000875538 | protein_coding |
| ENSMUSG00000056724 | Nbeal2 | neurobeachin-like 2 [Source:MGI Symbol;Acc:MGI:2448554] | 76,37396659 | -0,5115874 | **-1,42561799** | 6,24772037 | 15,0446366 | 0,000104998 | 0,00071123 | protein_coding |
| ENSMUSG00000060519 | Tor3a | torsin family 3, member A [Source:MGI Symbol;Acc:MGI:1353652] | 207,1712109 | -0,5118295 | **-1,42585717** | 7,69350932 | 13,6857988 | 0,000216082 | 0,001339599 | protein_coding |
| ENSMUSG00000028032 | Papss1 | 3'-phosphoadenosine 5'-phosphosulfate synthase 1 [Source:MGI Symbol;Acc:MGI:1330587] | 31,89970121 | -0,5121772 | **-1,42620088** | 4,98858163 | 10,4051257 | 0,00125666 | 0,006244651 | protein_coding |
| ENSMUSG00000081406 | Rps6-ps4 | ribosomal protein S6, pseudogene 4 [Source:MGI Symbol;Acc:MGI:3650907] | 780,1865327 | -0,5125963 | **-1,4266153** | 9,60754659 | 7,66888122 | 0,005618128 | 0,022372734 | pseudogene |
| ENSMUSG00000026791 | Slc2a8 | solute carrier family 2, (facilitated glucose transporter), member 8 [Source:MGI Symbol;Acc:MGI:1860103] | 15,6303119 | -0,512804 | **-1,42682067** | 3,95090426 | 8,53466899 | 0,003484447 | 0,015019074 | protein_coding |
| ENSMUSG00000038991 | Txndc5 | thioredoxin domain containing 5 [Source:MGI Symbol;Acc:MGI:2145316] | 127,6727876 | -0,5131545 | **-1,42716732** | 6,99525847 | 14,2167655 | 0,000162913 | 0,001047963 | protein_coding |
| ENSMUSG00000002107 | Celf2 | CUGBP, Elav-like family member 2 [Source:MGI Symbol;Acc:MGI:1338822] | 177,9640168 | -0,5131592 | **-1,42717197** | 7,47438041 | 14,2525839 | 0,000159841 | 0,001030619 | protein_coding |
| ENSMUSG00000018548 | Trim37 | tripartite motif-containing 37 [Source:MGI Symbol;Acc:MGI:2153072] | 29,44440694 | -0,513384 | **-1,42739436** | 4,87758722 | 12,139832 | 0,000493562 | 0,002772376 | protein_coding |
| ENSMUSG00000032741 | Tpcn1 | two pore channel 1 [Source:MGI Symbol;Acc:MGI:2182472] | 172,8239289 | -0,5144167 | **-1,4284165** | 7,43062882 | 15,1362438 | 0,000100024 | 0,000680897 | protein_coding |
| ENSMUSG00000012405 | Rpl15 | ribosomal protein L15 [Source:MGI Symbol;Acc:MGI:1913730] | 100,6715473 | -0,5144892 | **-1,4284883** | 6,65382478 | 16,7437287 | 4,27834E-05 | 0,000318254 | protein_coding |
| ENSMUSG00000021371 | Ccdc90a | coiled-coil domain containing 90A [Source:MGI Symbol;Acc:MGI:1923387] | 79,40913989 | -0,5146281 | **-1,42862584** | 6,30981521 | 14,9197467 | 0,000112183 | 0,000754315 | protein_coding |
| ENSMUSG00000024431 | Nr3c1 | nuclear receptor subfamily 3, group C, member 1 [Source:MGI Symbol;Acc:MGI:95824] | 140,1807506 | -0,5148293 | **-1,42882505** | 7,13019596 | 14,9914714 | 0,000107998 | 0,000728855 | protein_coding |
| ENSMUSG00000062082 | Cd200r4 | CD200 receptor 4 [Source:MGI Symbol;Acc:MGI:3036289] | 34,08295238 | -0,5149191 | **-1,42891398** | 5,07238942 | 13,275551 | 0,00026889 | 0,001622346 | protein_coding |
| ENSMUSG00000023106 | Denr | density-regulated protein [Source:MGI Symbol;Acc:MGI:1915434] | 123,7895918 | -0,5149628 | **-1,4289573** | 6,95221996 | 13,1525782 | 0,000287124 | 0,001723842 | protein_coding |
| ENSMUSG00000002731 | Prkra | protein kinase, interferon inducible double stranded RNA dependent activator [Source:MGI Symbol;Acc:MGI:1344375] | 29,3345737 | -0,5150175 | **-1,42901143** | 4,86847598 | 12,1721261 | 0,00048509 | 0,002734573 | protein_coding |
| ENSMUSG00000044068 | Zrsr1 | zinc finger (CCCH type), RNA binding motif and serine/arginine rich 1 [Source:MGI Symbol;Acc:MGI:98885] | 14,77983765 | -0,5152602 | **-1,42925186** | 3,87811664 | 8,00408208 | 0,004667202 | 0,019098154 | protein_coding |
| ENSMUSG00000041935 | AW549877 | expressed sequence AW549877 [Source:MGI Symbol;Acc:MGI:2146232] | 73,40202462 | -0,5159646 | **-1,42994993** | 6,19694062 | 15,0286958 | 0,000105889 | 0,000716574 | protein_coding |
| ENSMUSG00000037503 | Fam168b | family with sequence similarity 168, member B [Source:MGI Symbol;Acc:MGI:2448487] | 215,4530308 | -0,5160776 | **-1,43006186** | 7,75034226 | 13,4279328 | 0,000247905 | 0,001514869 | protein_coding |
| ENSMUSG00000042167 | Papd4 | PAP associated domain containing 4 [Source:MGI Symbol;Acc:MGI:2140950] | 75,65912616 | -0,5164457 | **-1,43042684** | 6,23930352 | 15,6283862 | 7,70886E-05 | 0,000543628 | protein_coding |
| ENSMUSG00000073455 | Gm3435 | predicted gene 3435 [Source:MGI Symbol;Acc:MGI:3810125] | 12,5223364 | -0,5166274 | **-1,43060696** | 3,62944255 | 6,80569 | 0,009086783 | 0,033592358 | protein_coding |
| ENSMUSG00000034744 | Nagk | N-acetylglucosamine kinase [Source:MGI Symbol;Acc:MGI:1860418] | 75,61537414 | -0,5169578 | **-1,43093465** | 6,23715722 | 15,5878441 | 7,87593E-05 | 0,000554342 | protein_coding |
| ENSMUSG00000042462 | Dctpp1 | dCTP pyrophosphatase 1 [Source:MGI Symbol;Acc:MGI:1913672] | 12,66442253 | -0,5175011 | **-1,43147365** | 3,66004135 | 6,30810851 | 0,0120187 | 0,042255824 | protein_coding |
| ENSMUSG00000026393 | Nek7 | NIMA (never in mitosis gene a)-related expressed kinase 7 [Source:MGI Symbol;Acc:MGI:1890645] | 119,6512522 | -0,5176072 | **-1,43157895** | 6,89978181 | 16,1738144 | 5,77874E-05 | 0,000418806 | protein_coding |
| ENSMUSG00000036712 | Cyld | cylindromatosis (turban tumor syndrome) [Source:MGI Symbol;Acc:MGI:1921506] | 77,29785123 | -0,5176449 | **-1,43161635** | 6,26916323 | 14,2685747 | 0,000158489 | 0,0010225 | protein_coding |
| ENSMUSG00000041650 | Pcca | propionyl-Coenzyme A carboxylase, alpha polypeptide [Source:MGI Symbol;Acc:MGI:97499] | 28,5464456 | -0,5181431 | **-1,43211083** | 4,83376421 | 11,748121 | 0,000609047 | 0,003336054 | protein_coding |
| ENSMUSG00000037470 | Uggt1 | UDP-glucose glycoprotein glucosyltransferase 1 [Source:MGI Symbol;Acc:MGI:2443162] | 104,5217014 | -0,5185107 | **-1,43247571** | 6,70704783 | 17,1353901 | 3,48076E-05 | 0,000265025 | protein_coding |
| ENSMUSG00000038622 | Med30 | mediator complex subunit 30 [Source:MGI Symbol;Acc:MGI:1917040] | 12,84084378 | -0,5190136 | **-1,4329752** | 3,67072748 | 7,18580314 | 0,007348265 | 0,028023392 | protein_coding |
| ENSMUSG00000001289 | Pfdn5 | prefoldin 5 [Source:MGI Symbol;Acc:MGI:1928753] | 108,5043753 | -0,519116 | **-1,43307685** | 6,75817718 | 15,0952202 | 0,000102222 | 0,000694994 | protein_coding |
| ENSMUSG00000030788 | Rnf141 | ring finger protein 141 [Source:MGI Symbol;Acc:MGI:1914400] | 98,05265726 | -0,5192244 | **-1,43318452** | 6,61526971 | 12,4312023 | 0,00042222 | 0,00241611 | protein_coding |
| ENSMUSG00000058454 | Dhcr7 | 7-dehydrocholesterol reductase [Source:MGI Symbol;Acc:MGI:1298378] | 26,91948619 | -0,5196042 | **-1,43356194** | 4,74108365 | 7,04839924 | 0,007933612 | 0,029954156 | protein_coding |
| ENSMUSG00000031539 | Ap3m2 | adaptor-related protein complex 3, mu 2 subunit [Source:MGI Symbol;Acc:MGI:1929214] | 25,3025377 | -0,5198733 | **-1,43382932** | 4,65168266 | 10,4711207 | 0,001212551 | 0,006055592 | protein_coding |
| ENSMUSG00000038872 | Zfhx3 | zinc finger homeobox 3 [Source:MGI Symbol;Acc:MGI:99948] | 68,36012778 | -0,5200638 | **-1,43401868** | 6,08590749 | 14,241245 | 0,000160807 | 0,001035232 | protein_coding |
| ENSMUSG00000024516 | Sec11c | SEC11 homolog C (S. cerevisiae) [Source:MGI Symbol;Acc:MGI:1913536] | 90,53946698 | -0,5201994 | **-1,43415343** | 6,49667587 | 15,952451 | 6,49536E-05 | 0,00046613 | protein_coding |
| ENSMUSG00000029482 | Aacs | acetoacetyl-CoA synthetase [Source:MGI Symbol;Acc:MGI:1926144] | 44,32808147 | -0,5202622 | **-1,43421588** | 5,45737357 | 9,76325786 | 0,001780341 | 0,00851943 | protein_coding |
| ENSMUSG00000018293 | Pfn1 | profilin 1 [Source:MGI Symbol;Acc:MGI:97549] | 1470,829479 | -0,5203756 | **-1,43432865** | 10,5223319 | 6,34462426 | 0,011773755 | 0,04158092 | protein_coding |
| ENSMUSG00000044477 | Zfand3 | zinc finger, AN1-type domain 3 [Source:MGI Symbol;Acc:MGI:1096572] | 111,5539246 | -0,5204289 | **-1,43438157** | 6,79951393 | 14,5740154 | 0,00013476 | 0,000886062 | protein_coding |
| ENSMUSG00000033016 | Nfatc1 | nuclear factor of activated T cells, cytoplasmic, calcineurin dependent 1 [Source:MGI Symbol;Acc:MGI:102469] | 68,78218643 | -0,5209604 | **-1,43491014** | 6,09925208 | 14,7649482 | 0,000127426 | 0,000844917 | protein_coding |
| ENSMUSG00000050751 | Pgbd5 | piggyBac transposable element derived 5 [Source:MGI Symbol;Acc:MGI:2429955] | 18,2853862 | -0,5217919 | **-1,43573738** | 4,17855091 | 8,43025346 | 0,003690289 | 0,015776345 | protein_coding |
| ENSMUSG00000029528 | Pxn | paxillin [Source:MGI Symbol;Acc:MGI:108295] | 203,078551 | -0,5219175 | **-1,43586241** | 7,66447301 | 12,4720961 | 0,000413076 | 0,002369958 | protein_coding |
| ENSMUSG00000063316 | Rpl27 | ribosomal protein L27 [Source:MGI Symbol;Acc:MGI:98036] | 23,9896086 | -0,5222679 | **-1,43621114** | 4,59325501 | 11,0506037 | 0,000886583 | 0,004627501 | protein_coding |
| ENSMUSG00000034868 | Myl12b | myosin, light chain 12B, regulatory [Source:MGI Symbol;Acc:MGI:107494] | 132,8387986 | -0,5223237 | **-1,43626674** | 7,05183268 | 14,7750826 | 0,000121125 | 0,000808509 | protein_coding |
| ENSMUSG00000018334 | Ksr1 | kinase suppressor of ras 1 [Source:MGI Symbol;Acc:MGI:105051] | 16,96884105 | -0,522402 | **-1,43634465** | 4,07331144 | 7,49866904 | 0,006174461 | 0,024202212 | protein_coding |
| ENSMUSG00000035227 | Spcs2 | signal peptidase complex subunit 2 homolog (S. cerevisiae) [Source:MGI Symbol;Acc:MGI:1913874] | 134,3258648 | -0,5231892 | **-1,43712867** | 7,06818587 | 14,8446479 | 0,000116739 | 0,000782079 | protein_coding |
| ENSMUSG00000028603 | Scp2 | sterol carrier protein 2, liver [Source:MGI Symbol;Acc:MGI:98254] | 156,7100815 | -0,5234239 | **-1,43736244** | 7,29020408 | 14,7252238 | 0,000124371 | 0,00082766 | protein_coding |
| ENSMUSG00000021068 | Nin | ninein [Source:MGI Symbol;Acc:MGI:105108] | 98,98435081 | -0,523561 | **-1,43749904** | 6,62632548 | 14,8487801 | 0,000116483 | 0,000780844 | protein_coding |
| ENSMUSG00000029387 | Gtf2h3 | general transcription factor IIH, polypeptide 3 [Source:MGI Symbol;Acc:MGI:1277143] | 11,59190475 | -0,5237708 | **-1,43770808** | 3,52654722 | 6,09331114 | 0,013569456 | 0,04641582 | protein_coding |
| ENSMUSG00000020048 | Hsp90b1 | heat shock protein 90, beta (Grp94), member 1 [Source:MGI Symbol;Acc:MGI:98817] | 577,8116934 | -0,5238152 | **-1,43775239** | 9,17445935 | 9,40285355 | 0,00216648 | 0,010043509 | protein_coding |
| ENSMUSG00000027843 | Ptpn22 | protein tyrosine phosphatase, non-receptor type 22 (lymphoid) [Source:MGI Symbol;Acc:MGI:107170] | 33,11235283 | -0,5242589 | **-1,43819465** | 5,04019438 | 13,1424151 | 0,000288686 | 0,001730381 | protein_coding |
| ENSMUSG00000000538 | Tom1l2 | target of myb1-like 2 (chicken) [Source:MGI Symbol;Acc:MGI:2443306] | 38,43827693 | -0,5248151 | **-1,43874916** | 5,25525112 | 12,1986248 | 0,000478248 | 0,002704326 | protein_coding |

| | | | | | | | | | | |
|---|---|---|---|---|---|---|---|---|---|---|
| ENSMUSG00000020484 | Xbp1 | X-box binding protein 1 [Source:MGI Symbol;Acc:MGI:98970] | 135,9762555 | -0,5248174 | **-1,43875143** | 7,08698448 | 12,9316607 | 0,000323071 | 0,001907352 | protein_coding |
| ENSMUSG00000063882 | Uqcrh | ubiquinol-cytochrome c reductase hinge protein [Source:MGI Symbol;Acc:MGI:1913826] | 96,21923501 | -0,5252723 | **-1,43920515** | 6,5852478 | 14,6107993 | 0,000132155 | 0,000872065 | protein_coding |
| ENSMUSG00000024772 | Ehd1 | EH-domain containing 1 [Source:MGI Symbol;Acc:MGI:1341878] | 626,5882636 | -0,5252769 | **-1,43920979** | 9,29132059 | 8,06878472 | 0,004503431 | 0,018538478 | protein_coding |
| ENSMUSG00000026131 | Dst | dystonin [Source:MGI Symbol;Acc:MGI:104627] | 285,4737963 | -0,525415 | **-1,43934756** | 8,15643683 | 12,5669634 | 0,000392626 | 0,002275067 | protein_coding |
| ENSMUSG00000061315 | Naca | nascent polypeptide-associated complex alpha polypeptide [Source:MGI Symbol;Acc:MGI:106095] | 258,3806573 | -0,5265381 | **-1,44046853** | 8,01249781 | 13,2705955 | 0,000269602 | 0,001625748 | protein_coding |
| ENSMUSG00000059669 | Taf1b | TATA box binding protein (Tbp)-associated factor, RNA polymerase I, B [Source:MGI Symbol;Acc:MGI:109577] | 12,16721477 | -0,5265892 | **-1,44051952** | 3,60331858 | 6,95715972 | 0,008348446 | 0,031202134 | protein_coding |
| ENSMUSG00000020585 | Laptm4a | lysosomal-associated protein transmembrane 4A [Source:MGI Symbol;Acc:MGI:108017] | 540,2409846 | -0,5266498 | **-1,44058006** | 9,07727581 | 9,14470258 | 0,002494392 | 0,011310723 | protein_coding |
| ENSMUSG00000028799 | Zfp362 | zinc finger protein 362 [Source:MGI Symbol;Acc:MGI:2652839] | 33,25682855 | -0,5270971 | **-1,44102671** | 5,04655839 | 14,0096965 | 0,00018187 | 0,001153035 | protein_coding |
| ENSMUSG00000074785 | Plxnc1 | plexin C1 [Source:MGI Symbol;Acc:MGI:1890127] | 111,6346474 | -0,5271768 | **-1,44110634** | 6,79679454 | 16,9635792 | 3,81038E-05 | 0,000287532 | protein_coding |
| ENSMUSG00000028843 | Sh3bgrl3 | SH3 domain binding glutamic acid-rich protein-like 3 [Source:MGI Symbol;Acc:MGI:1920973] | 591,502144 | -0,527521 | **-1,44145018** | 9,20783195 | 9,58621067 | 0,001960441 | 0,009212732 | protein_coding |
| ENSMUSG00000021365 | Nedd9 | neural precursor cell expressed, developmentally down-regulated gene 9 [Source:MGI Symbol;Acc:MGI:97302] | 20,64316188 | -0,5275301 | **-1,44145926** | 4,35529853 | 10,5363971 | 0,001170464 | 0,005869413 | protein_coding |
| ENSMUSG00000059355 | BC056474 | cDNA sequence BC056474 [Source:MGI Symbol;Acc:MGI:3041257] | 50,03933247 | -0,528695 | **-1,44262368** | 5,6383703 | 12,4680835 | 0,000413965 | 0,002373815 | protein_coding |
| ENSMUSG00000028081 | Rps3a | ribosomal protein S3A [Source:MGI Symbol;Acc:MGI:1202063] | 692,3119143 | -0,5287457 | **-1,4426744** | 9,43512025 | 8,86156648 | 0,002912375 | 0,01288175 | protein_coding |
| ENSMUSG00000033767 | D930015E06Rik | RIKEN cDNA D930015E06 gene [Source:MGI Symbol;Acc:MGI:2443399] | 42,26877593 | -0,5289185 | **-1,44284714** | 5,39597683 | 12,6621885 | 0,000373125 | 0,002172172 | protein_coding |
| ENSMUSG00000007833 | Aldh16a1 | aldehyde dehydrogenase 16 family, member A1 [Source:MGI Symbol;Acc:MGI:1916998] | 64,16029908 | -0,5303563 | **-1,44428583** | 5,99766273 | 14,5015114 | 0,000140047 | 0,000916437 | protein_coding |
| ENSMUSG00000073616 | Myeov2 | myeloma overexpressed 2 [Source:MGI Symbol;Acc:MGI:1914165] | 48,65721198 | -0,5303899 | **-1,44431945** | 5,59913505 | 14,903708 | 0,00011314 | 0,00076029 | protein_coding |
| ENSMUSG00000038615 | Nfe2l1 | nuclear factor, erythroid derived 2,-like 1 [Source:MGI Symbol;Acc:MGI:99421] | 437,2161306 | -0,5304584 | **-1,44438802** | 8,7717366 | 8,95835895 | 0,002762029 | 0,012305925 | protein_coding |
| ENSMUSG00000041362 | 4930506M07Rik | RIKEN cDNA 4930506M07 gene [Source:MGI Symbol;Acc:MGI:1918903] | 247,2404494 | -0,5306868 | **-1,4446167** | 7,94917197 | 13,6889562 | 0,000215719 | 0,001339044 | protein_coding |
| ENSMUSG00000032249 | Anp32a | acidic (leucine-rich) nuclear phosphoprotein 32 family, member A [Source:MGI Symbol;Acc:MGI:108447] | 174,3792577 | -0,5307035 | **-1,44463351** | 7,44583136 | 14,5959887 | 0,000133198 | 0,000877789 | protein_coding |
| ENSMUSG00000054277 | Arfgap3 | ADP-ribosylation factor GTPase activating protein 3 [Source:MGI Symbol;Acc:MGI:1913501] | 39,32567201 | -0,5313065 | **-1,44523736** | 5,29086368 | 13,3658662 | 0,000256246 | 0,001556031 | protein_coding |
| ENSMUSG00000019370 | Calm3 | calmodulin 3 [Source:MGI Symbol;Acc:MGI:103249] | 279,4707183 | -0,5314343 | **-1,44536539** | 8,12572717 | 12,7501647 | 0,000355978 | 0,002082603 | protein_coding |
| ENSMUSG00000030432 | Rpl28 | ribosomal protein L28 [Source:MGI Symbol;Acc:MGI:101839] | 47,44237563 | -0,5318093 | **-1,44574116** | 5,56119611 | 12,5539718 | 0,000395365 | 0,002285048 | protein_coding |
| ENSMUSG00000057858 | D19Ertd737e | DNA segment, Chr 19, ERATO Doi 737, expressed [Source:MGI Symbol;Acc:MGI:1289174] | 14,62620435 | -0,5320954 | **-1,4460279** | 3,86662321 | 8,1397128 | 0,004330614 | 0,017961668 | protein_coding |
| ENSMUSG00000046229 | Scand1 | SCAN domain-containing 1 [Source:MGI Symbol;Acc:MGI:1343132] | 39,84074855 | -0,5321753 | **-1,44610798** | 5,30074497 | 13,6046671 | 0,000225624 | 0,001391094 | protein_coding |
| ENSMUSG00000059182 | Skap2 | src family associated phosphoprotein 2 [Source:MGI Symbol;Acc:MGI:1889206] | 262,1506766 | -0,5325686 | **-1,44650231** | 8,03358831 | 13,3136943 | 0,000263475 | 0,001595809 | protein_coding |
| ENSMUSG00000024193 | Phf1 | PHD finger protein 1 [Source:MGI Symbol;Acc:MGI:98647] | 20,06356306 | -0,5333949 | **-1,44733096** | 4,31012789 | 10,2134354 | 0,001394212 | 0,006836099 | protein_coding |
| ENSMUSG00000086130 | Gm16211 | predicted gene 16211 [Source:MGI Symbol;Acc:MGI:3802078] | 33,09411675 | -0,5341553 | **-1,44809408** | 5,04792105 | 12,2237316 | 0,000471855 | 0,002672302 | lincRNA |
| ENSMUSG00000033706 | Smyd5 | SET and MYND domain containing 5 [Source:MGI Symbol;Acc:MGI:108048] | 75,81580518 | -0,5358775 | **-1,44982367** | 6,2422219 | 15,5448235 | 8,0572E-05 | 0,000563491 | protein_coding |
| ENSMUSG00000020246 | Hcfc2 | host cell factor C2 [Source:MGI Symbol;Acc:MGI:1915183] | 35,7057071 | -0,5373098 | **-1,45126378** | 5,15758422 | 13,1002369 | 0,000295258 | 0,001763046 | protein_coding |
| ENSMUSG00000047248 | C2cd3 | C2 calcium-dependent domain containing 3 [Source:MGI Symbol;Acc:MGI:2142166] | 31,11213575 | -0,5376088 | **-1,45156465** | 4,95465888 | 13,1855379 | 0,000282118 | 0,001695639 | protein_coding |
| ENSMUSG00000025137 | Pcyt2 | phosphate cytidylyltransferase 2, ethanolamine [Source:MGI Symbol;Acc:MGI:1915921] | 36,99110832 | -0,5377092 | **-1,45166563** | 5,19692202 | 9,0353969 | 0,002648016 | 0,011876788 | protein_coding |
| ENSMUSG00000020827 | Mink1 | misshapen-like kinase 1 (zebrafish) [Source:MGI Symbol;Acc:MGI:1355329] | 210,2351246 | -0,5377692 | **-1,45172598** | 7,71280024 | 15,5799394 | 7,90893E-05 | 0,000555484 | protein_coding |
| ENSMUSG00000024965 | Fermt3 | fermitin family homolog 3 (Drosophila) [Source:MGI Symbol;Acc:MGI:2147790] | 617,0769853 | -0,5383322 | **-1,45229266** | 9,26895593 | 9,59105963 | 0,001955271 | 0,009196301 | protein_coding |
| ENSMUSG00000006526 | Tmem110 | transmembrane protein 110 [Source:MGI Symbol;Acc:MGI:1921500] | 16,57610852 | -0,5395708 | **-1,45354004** | 4,03850979 | 10,2642414 | 0,001356335 | 0,006682535 | protein_coding |
| ENSMUSG00000031924 | Cyb5b | cytochrome b5 type B [Source:MGI Symbol;Acc:MGI:1913677] | 204,3425156 | -0,5398617 | **-1,45383315** | 7,67420443 | 12,1446737 | 0,000492283 | 0,002766603 | protein_coding |
| ENSMUSG00000019998 | Stx7 | syntaxin 7 [Source:MGI Symbol;Acc:MGI:1858210] | 194,1128097 | -0,5402935 | **-1,45426838** | 7,60063826 | 14,3999392 | 0,000147807 | 0,000960353 | protein_coding |
| ENSMUSG00000008683 | Rps15a | ribosomal protein S15A [Source:MGI Symbol;Acc:MGI:2389091] | 360,0135609 | -0,5403261 | **-1,45430119** | 8,49127095 | 11,7102218 | 0,000621577 | 0,003394268 | protein_coding |
| ENSMUSG00000027133 | Nop10 | NOP10 ribonucleoprotein homolog (yeast) [Source:MGI Symbol;Acc:MGI:1913431] | 32,83262752 | -0,5407979 | **-1,45477686** | 5,03479075 | 10,7608103 | 0,00103672 | 0,005292956 | protein_coding |
| ENSMUSG00000002733 | Plekha3 | pleckstrin homology domain-containing, family A (phosphoinositide binding specific) member 3 [Source:MGI Symbol;Acc | 63,58378209 | -0,5410621 | **-1,45504331** | 5,98888728 | 16,2208625 | 5,63699E-05 | 0,000410156 | protein_coding |
| ENSMUSG00000016664 | Pacsin2 | protein kinase C and casein kinase substrate in neurons 2 [Source:MGI Symbol;Acc:MGI:1345153] | 159,5972604 | -0,5412846 | **-1,45526774** | 7,31750156 | 16,2179221 | 5,64575E-05 | 0,000410522 | protein_coding |
| ENSMUSG00000022351 | Sqle | squalene epoxidase [Source:MGI Symbol;Acc:MGI:109296] | 50,44905484 | -0,5413129 | **-1,45529625** | 5,65081025 | 6,79969617 | 0,009117339 | 0,033660013 | protein_coding |
| ENSMUSG00000028207 | Asph | aspartate-beta-hydroxylase [Source:MGI Symbol;Acc:MGI:1914186] | 246,6278679 | -0,5416407 | **-1,45562699** | 7,94580212 | 14,7644264 | 0,000121812 | 0,000812597 | protein_coding |
| ENSMUSG00000031824 | 6430548M08Rik | RIKEN cDNA 6430548M08 gene [Source:MGI Symbol;Acc:MGI:2443793] | 61,44722941 | -0,541656 | **-1,45564244** | 5,93712191 | 15,4680695 | 8,39109E-05 | 0,000585351 | protein_coding |
| ENSMUSG00000045896 | Paip2b | poly(A) binding protein interacting protein 2B [Source:MGI Symbol;Acc:MGI:2386865] | 21,72615767 | -0,5425396 | **-1,45653419** | 4,44178561 | 11,0046189 | 0,000908851 | 0,004725768 | protein_coding |
| ENSMUSG00000050299 | Gm9843 | predicted gene 9843 [Source:MGI Symbol;Acc:MGI:3708621] | 447,4450765 | -0,5426203 | **-1,45661574** | 8,80449827 | 10,0122273 | 0,001555043 | 0,007543162 | protein_coding |
| ENSMUSG00000027346 | Gpcpd1 | glycerophosphocholine phosphodiesterase GDE1 homolog (S. cerevisiae) [Source:MGI Symbol;Acc:MGI:104898] | 42,50860971 | -0,5428602 | **-1,45685789** | 5,40381876 | 15,5501752 | 8,03442E-05 | 0,000562256 | protein_coding |
| ENSMUSG00000020064 | Herc4 | hect domain and RLD 4 [Source:MGI Symbol;Acc:MGI:1914595] | 102,2803981 | -0,5430178 | **-1,45701709** | 6,67525031 | 15,0745398 | 0,000103348 | 0,000702216 | protein_coding |
| ENSMUSG00000046020 | Pofut1 | protein O-fucosyltransferase 1 [Source:MGI Symbol;Acc:MGI:2153207] | 83,33534048 | -0,5433013 | **-1,4573034** | 6,37782419 | 14,7903718 | 0,000120147 | 0,000802468 | protein_coding |
| ENSMUSG00000034574 | Daam1 | dishevelled associated activator of morphogenesis 1 [Source:MGI Symbol;Acc:MGI:1914596] | 68,28320352 | -0,5448641 | **-1,45888296** | 6,09035421 | 15,9948346 | 6,35155E-05 | 0,000457004 | protein_coding |
| ENSMUSG00000078886 | Gm2026 | predicted gene 2026 [Source:MGI Symbol;Acc:MGI:3780195] | 13,74560203 | -0,5452891 | **-1,45931278** | 3,7666623 | 7,99105785 | 0,004700894 | 0,019214555 | protein_coding |
| ENSMUSG00000027806 | Tsc22d2 | TSC22 domain family, member 2 [Source:MGI Symbol;Acc:MGI:1919283] | 106,1868254 | -0,5457621 | **-1,45979125** | 6,72751426 | 13,2024271 | 0,000279587 | 0,001682267 | protein_coding |
| ENSMUSG00000034858 | BC031353 | cDNA sequence BC031353 [Source:MGI Symbol;Acc:MGI:2387648] | 16,01409995 | -0,5457881 | **-1,45981756** | 3,98707198 | 9,27211387 | 0,00232669 | 0,010664725 | protein_coding |
| ENSMUSG00000039450 | Dcxr | dicarbonyl L-xylulose reductase [Source:MGI Symbol;Acc:MGI:1915130] | 35,17464373 | -0,5458122 | **-1,45984199** | 5,12774506 | 13,5611743 | 0,000230913 | 0,001419718 | protein_coding |
| ENSMUSG00000039307 | Hexdc | hexosaminidase (glycosyl hydrolase family 20, catalytic domain) containing [Source:MGI Symbol;Acc:MGI:3605542] | 111,5719716 | -0,546177 | **-1,46021119** | 6,80221364 | 15,8617303 | 6,81428E-05 | 0,000485529 | protein_coding |
| ENSMUSG00000037795 | N4bp2 | NEDD4 binding protein 2 [Source:MGI Symbol;Acc:MGI:2684414] | 23,2770908 | -0,5461858 | **-1,46022001** | 4,53471009 | 11,0061938 | 0,000908079 | 0,00472399 | protein_coding |
| ENSMUSG00000059734 | Ndufs8 | NADH dehydrogenase (ubiquinone) Fe-S protein 8 [Source:MGI Symbol;Acc:MGI:2385079] | 30,13858062 | -0,5473693 | **-1,46141845** | 4,90007996 | 13,3339169 | 0,000260649 | 0,001581306 | protein_coding |
| ENSMUSG00000036339 | 6720456H20Rik | RIKEN cDNA 6720456H20 gene [Source:MGI Symbol;Acc:MGI:2443219] | 25,07750103 | -0,5481838 | **-1,46224378** | 4,63912185 | 10,8148254 | 0,001006905 | 0,005164737 | protein_coding |
| ENSMUSG00000019927 | Ube2d1 | ubiquitin-conjugating enzyme E2D 1 [Source:MGI Symbol;Acc:MGI:2384911] | 21,4035332 | -0,5485627 | **-1,46262781** | 4,41313765 | 9,60700383 | 0,001938366 | 0,009128518 | protein_coding |
| ENSMUSG00000028894 | Inpp5b | inositol polyphosphate-5-phosphatase B [Source:MGI Symbol;Acc:MGI:103257] | 96,07707563 | -0,5493537 | **-1,46342998** | 6,58649634 | 15,6691565 | 7,54445E-05 | 0,000533403 | protein_coding |
| ENSMUSG00000061132 | Blnk | B cell linker [Source:MGI Symbol;Acc:MGI:96878] | 251,1520812 | -0,5496435 | **-1,46372394** | 7,97137169 | 14,9199112 | 0,000112173 | 0,000754315 | protein_coding |
| ENSMUSG00000036672 | Cenpt | centromere protein T [Source:MGI Symbol;Acc:MGI:2443939] | 18,48130464 | -0,549722 | **-1,46380364** | 4,1994952 | 8,97135697 | 0,002742448 | 0,012228604 | protein_coding |
| ENSMUSG00000022361 | Zhx1 | zinc fingers and homeoboxes 1 [Source:MGI Symbol;Acc:MGI:109271] | 66,07691072 | -0,5502543 | **-1,46434378** | 6,04246613 | 17,3132115 | 3,16975E-05 | 0,000244222 | protein_coding |
| ENSMUSG00000007603 | Dus3l | dihydrouridine synthase 3-like (S. cerevisiae) [Source:MGI Symbol;Acc:MGI:2147092] | 33,92787106 | -0,5506605 | **-1,46475617** | 5,07317162 | 13,3717251 | 0,000255446 | 0,001554036 | protein_coding |
| ENSMUSG00000042106 | 6230427J02Rik | RIKEN cDNA 6230427J02 gene [Source:MGI Symbol;Acc:MGI:1915426] | 12,7786245 | -0,5507113 | **-1,46480777** | 3,66296232 | 7,21510894 | 0,007229243 | 0,027665513 | protein_coding |
| ENSMUSG00000020476 | Dbnl | drebrin-like [Source:MGI Symbol;Acc:MGI:700006] | 144,3190015 | -0,5519657 | **-1,46608192** | 7,1717144 | 16,2603124 | 5,52083E-05 | 0,000403307 | protein_coding |
| ENSMUSG00000054408 | Spcs3 | signal peptidase complex subunit 3 homolog (S. cerevisiae) [Source:MGI Symbol;Acc:MGI:1923937] | 146,0949361 | -0,5521968 | **-1,46631673** | 7,19057116 | 15,2553009 | 9,39128E-05 | 0,00064459 | protein_coding |
| ENSMUSG00000016921 | Srsf6 | serine/arginine-rich splicing factor 6 [Source:MGI Symbol;Acc:MGI:1915246] | 200,360201 | -0,5527988 | **-1,46692876** | 7,64549629 | 15,5859903 | 7,88366E-05 | 0,000554531 | protein_coding |
| ENSMUSG00000022337 | Emc2 | ER membrane protein complex subunit 2 [Source:MGI Symbol;Acc:MGI:1913986] | 68,52942639 | -0,5531273 | **-1,46726283** | 6,09872596 | 18,0698659 | 2,12945E-05 | 0,000172031 | protein_coding |
| ENSMUSG00000027615 | Hps3 | Hermansky-Pudlak syndrome 3 homolog (human) [Source:MGI Symbol;Acc:MGI:2153839] | 50,84227859 | -0,5537649 | **-1,46791143** | 5,6621012 | 16,8077527 | 4,1364E-05 | 0,000308531 | protein_coding |
| ENSMUSG00000061286 | Exosc5 | exosome component 5 [Source:MGI Symbol;Acc:MGI:107889] | 23,24655734 | -0,5546489 | **-1,46881113** | 4,52624959 | 11,32053 | 0,000766548 | 0,004090367 | protein_coding |
| ENSMUSG00000024083 | Pja2 | praja 2, RING-H2 motif containing [Source:MGI Symbol;Acc:MGI:2159342] | 106,4731836 | -0,5551971 | **-1,46936938** | 6,73383964 | 18,0761635 | 2,12242E-05 | 0,000171604 | protein_coding |

| | | | | | | | | | | |
|---|---|---|---|---|---|---|---|---|---|---|
| ENSMUSG00000025626 | Phf6 | PHD finger protein 6 [Source:MGI Symbol;Acc:MGI:1918248] | 70,22210068 | -0,5552543 | **-1,46942758** | 6,1343704 | 17,1716183 | 3,41501E-05 | 0,00026056 | protein_coding |
| ENSMUSG00000025290 | Rps24 | ribosomal protein S24 [Source:MGI Symbol;Acc:MGI:98147] | 643,539997 | -0,5568199 | **-1,47102311** | 9,32964903 | 10,0475735 | 0,001525487 | 0,007422733 | protein_coding |
| ENSMUSG00000017754 | Pltp | phospholipid transfer protein [Source:MGI Symbol;Acc:MGI:103151] | 1415,517049 | -0,5570132 | **-1,47122023** | 10,4669794 | 6,62392389 | 0,010061792 | 0,03656927 | protein_coding |
| ENSMUSG00000035064 | Eef2k | eukaryotic elongation factor-2 kinase [Source:MGI Symbol;Acc:MGI:1195261] | 14,19277123 | -0,5573079 | **-1,47152077** | 3,81288968 | 8,44495686 | 0,003660571 | 0,015667587 | protein_coding |
| ENSMUSG00000072940 | Gm10443 | predicted pseudogene 10443 [Source:MGI Symbol;Acc:MGI:3704272] | 94,75111701 | -0,5578406 | **-1,47206416** | 6,55981044 | 15,8564583 | 6,83329E-05 | 0,000486568 | pseudogene |
| ENSMUSG00000018547 | Pip4k2b | phosphatidylinositol-5-phosphate 4-kinase, type II, beta [Source:MGI Symbol;Acc:MGI:1934234] | 142,6271258 | -0,5583565 | **-1,47259074** | 7,15449602 | 14,6473354 | 0,000129618 | 0,000857381 | protein_coding |
| ENSMUSG00000027882 | Stxbp3a | syntaxin binding protein 3A [Source:MGI Symbol;Acc:MGI:107362] | 136,8911583 | -0,5588183 | **-1,47306214** | 7,09503732 | 15,6848911 | 7,48195E-05 | 0,000530349 | protein_coding |
| ENSMUSG00000021375 | Kif13a | kinesin family member 13A [Source:MGI Symbol;Acc:MGI:1098264] | 37,87593457 | -0,5600508 | **-1,47432112** | 5,23322748 | 14,4339842 | 0,000145159 | 0,000946506 | protein_coding |
| ENSMUSG00000024725 | Ostf1 | osteoclast stimulating factor 1 [Source:MGI Symbol;Acc:MGI:700012] | 308,7823237 | -0,560053 | **-1,47432339** | 8,26978122 | 13,768879 | 0,000206733 | 0,001289819 | protein_coding |
| ENSMUSG00000028463 | Car9 | carbonic anhydrase 9 [Source:MGI Symbol;Acc:MGI:2447188] | 13,83770396 | -0,5602052 | **-1,47447889** | 3,7888845 | 9,34998326 | 0,002229875 | 0,010285322 | protein_coding |
| ENSMUSG00000032563 | Mrpl3 | mitochondrial ribosomal protein L3 [Source:MGI Symbol;Acc:MGI:2137204] | 81,54893712 | -0,5608116 | **-1,47509878** | 6,34911903 | 17,8291079 | 2,41658E-05 | 0,000192259 | protein_coding |
| ENSMUSG00000033059 | Pygb | brain glycogen phosphorylase [Source:MGI Symbol;Acc:MGI:97828] | 107,2986452 | -0,5614799 | **-1,47578228** | 6,7444561 | 16,5272005 | 4,79571E-05 | 0,000354103 | protein_coding |
| ENSMUSG00000040616 | Tmem51 | transmembrane protein 51 [Source:MGI Symbol;Acc:MGI:2384874] | 40,9003056 | -0,5615304 | **-1,47583393** | 5,34806876 | 13,1818227 | 0,000282678 | 0,001698075 | protein_coding |
| ENSMUSG00000034192 | Lsm3 | LSM3 homolog, U6 small nuclear RNA associated (S. cerevisiae) [Source:MGI Symbol;Acc:MGI:1914928] | 19,13359528 | -0,561667 | **-1,47597366** | 4,25495249 | 11,1596931 | 0,000835938 | 0,00439237 | protein_coding |
| ENSMUSG00000000740 | Rpl13 | ribosomal protein L13 [Source:MGI Symbol;Acc:MGI:105922] | 442,0507901 | -0,5621354 | **-1,47645297** | 8,7869882 | 11,7843314 | 0,000597313 | 0,003278063 | protein_coding |
| ENSMUSG00000033857 | Engase | endo-beta-N-acetylglucosaminidase [Source:MGI Symbol;Acc:MGI:2443788] | 10,29590569 | -0,5626438 | **-1,47697338** | 3,35599657 | 6,91799094 | 0,008533279 | 0,031802964 | protein_coding |
| ENSMUSG00000020652 | Cenpo | centromere protein O [Source:MGI Symbol;Acc:MGI:1923800] | 15,44847754 | -0,5640898 | **-1,47845443** | 3,93878237 | 9,65736345 | 0,001885941 | 0,008912186 | protein_coding |
| ENSMUSG00000071359 | Tbpl1 | TATA box binding protein-like 1 [Source:MGI Symbol;Acc:MGI:1339946] | 31,31858419 | -0,5656131 | **-1,4800163** | 4,96212843 | 13,9484463 | 0,000187893 | 0,001189158 | protein_coding |
| ENSMUSG00000032038 | St3gal4 | ST3 beta-galactoside alpha-2,3-sialyltransferase 4 [Source:MGI Symbol;Acc:MGI:1316743] | 14,71411046 | -0,5659712 | **-1,48038376** | 3,88999075 | 9,30034594 | 0,002291106 | 0,01052357 | protein_coding |
| ENSMUSG00000030871 | Ears2 | glutamyl-tRNA synthetase 2 (mitochondrial)(putative) [Source:MGI Symbol;Acc:MGI:1914667] | 10,66576695 | -0,5659772 | **-1,4803899** | 3,40806291 | 6,5937821 | 0,010233554 | 0,037107609 | protein_coding |
| ENSMUSG00000027544 | Nfatc2 | nuclear factor of activated T cells, cytoplasmic, calcineurin dependent 2 [Source:MGI Symbol;Acc:MGI:102463] | 142,1303003 | -0,5663685 | **-1,48079147** | 7,14797501 | 13,3061005 | 0,000264544 | 0,001601402 | protein_coding |
| ENSMUSG00000067870 | Rpl31-ps8 | ribosomal protein L31, pseudogene 8 [Source:MGI Symbol;Acc:MGI:3647726] | 399,4762371 | -0,5665082 | **-1,48093487** | 8,64136485 | 12,0062421 | 0,000530227 | 0,002957157 | pseudogene |
| ENSMUSG00000054387 | Mdm4 | transformed mouse 3T3 cell double minute 4 [Source:MGI Symbol;Acc:MGI:107934] | 46,94765783 | -0,5666288 | **-1,48105865** | 5,54855035 | 14,1403509 | 0,000169665 | 0,001084297 | protein_coding |
| ENSMUSG00000022186 | Oxct1 | 3-oxoacid CoA transferase 1 [Source:MGI Symbol;Acc:MGI:1914291] | 177,7532395 | -0,5668153 | **-1,4812502** | 7,47350912 | 17,5072794 | 2,8621E-05 | 0,00022302 | protein_coding |
| ENSMUSG00000052146 | Rps10 | ribosomal protein S10 [Source:MGI Symbol;Acc:MGI:1914347] | 439,764333 | -0,5671992 | **-1,48164433** | 8,77972951 | 11,7600548 | 0,000605154 | 0,00331778 | protein_coding |
| ENSMUSG00000043895 | S1pr2 | sphingosine-1-phosphate receptor 2 [Source:MGI Symbol;Acc:MGI:99569] | 187,8010151 | -0,5675798 | **-1,48203527** | 7,55191049 | 17,9218066 | 2,30169E-05 | 0,000184589 | protein_coding |
| ENSMUSG00000037341 | Slc9a7 | solute carrier family 9 (sodium/hydrogen exchanger), member 7 [Source:MGI Symbol;Acc:MGI:2444530] | 10,00805918 | -0,567604 | **-1,4820601** | 3,3184235 | 7,19060539 | 0,007328625 | 0,027987349 | protein_coding |
| ENSMUSG00000055923 | Aasdh | aminoadipate-semialdehyde dehydrogenase [Source:MGI Symbol;Acc:MGI:2442517] | 9,815369735 | -0,5676227 | **-1,48207941** | 3,27524567 | 7,03026126 | 0,008014366 | 0,03019679 | protein_coding |
| ENSMUSG00000055228 | Zfp935 | zinc finger protein 935 [Source:MGI Symbol;Acc:MGI:1918758] | 9,694456168 | -0,5676311 | **-1,48208794** | 3,28026791 | 7,04602734 | 0,007944125 | 0,029983545 | protein_coding |
| ENSMUSG00000030560 | Ctsc | cathepsin C [Source:MGI Symbol;Acc:MGI:109553] | 481,7522361 | -0,5683974 | **-1,48287547** | 8,91220696 | 8,36109456 | 0,003833396 | 0,016217759 | protein_coding |
| ENSMUSG00000037337 | Map4k1 | mitogen-activated protein kinase kinase kinase kinase 1 [Source:MGI Symbol;Acc:MGI:1346882] | 13,43787537 | -0,5687549 | **-1,48324296** | 3,73032945 | 8,30685344 | 0,003949576 | 0,016628367 | protein_coding |
| ENSMUSG00000022957 | Itsn1 | intersectin 1 (SH3 domain protein 1A) [Source:MGI Symbol;Acc:MGI:1338069] | 428,821291 | -0,5695855 | **-1,48409713** | 8,74414003 | 11,7479748 | 0,000609095 | 0,003336054 | protein_coding |
| ENSMUSG00000028581 | Laptm5 | lysosomal-associated protein transmembrane 5 [Source:MGI Symbol;Acc:MGI:108046] | 2180,315264 | -0,5703547 | **-1,48488864** | 11,0902549 | 6,61996299 | 0,010084193 | 0,036614351 | protein_coding |
| ENSMUSG00000031712 | Il15 | interleukin 15 [Source:MGI Symbol;Acc:MGI:103014] | 10,13716625 | -0,5705303 | **-1,48506931** | 3,33092111 | 6,80336264 | 0,009098635 | 0,033624859 | protein_coding |
| ENSMUSG00000039270 | Megf9 | multiple EGF-like-domains 9 [Source:MGI Symbol;Acc:MGI:1918264] | 27,91639984 | -0,5707684 | **-1,48531446** | 4,80727986 | 12,8586438 | 0,000335924 | 0,001975801 | protein_coding |
| ENSMUSG00000025967 | Eef1b2 | eukaryotic translation elongation factor 1 beta 2 [Source:MGI Symbol;Acc:MGI:1929520] | 380,1357405 | -0,5710084 | **-1,48556157** | 8,56975173 | 12,8965194 | 0,000329194 | 0,001942455 | protein_coding |
| ENSMUSG00000028964 | Park7 | Parkinson disease (autosomal recessive, early onset) 7 [Source:MGI Symbol;Acc:MGI:2135637] | 86,84528828 | -0,5710557 | **-1,48561028** | 6,43840726 | 19,4793674 | 1,01692E-05 | 8,80451E-05 | protein_coding |
| ENSMUSG00000034165 | Ccnd3 | cyclin D3 [Source:MGI Symbol;Acc:MGI:88315] | 67,19412247 | -0,5715405 | **-1,4861096** | 6,06957169 | 14,6387032 | 0,000130213 | 0,000860799 | protein_coding |
| ENSMUSG00000032000 | Birc3 | baculoviral IAP repeat-containing 3 [Source:MGI Symbol;Acc:MGI:1197007] | 200,5676839 | -0,5720773 | **-1,48666261** | 7,64799037 | 12,2323883 | 0,00046967 | 0,002662677 | protein_coding |
| ENSMUSG00000024277 | Mapre2 | microtubule-associated protein, RP/EB family, member 2 [Source:MGI Symbol;Acc:MGI:106271] | 101,3436949 | -0,5722068 | **-1,48679612** | 6,66158598 | 20,5438721 | 5,82801E-06 | 5,3494E-05 | protein_coding |
| ENSMUSG00000028028 | Alpk1 | alpha-kinase 1 [Source:MGI Symbol;Acc:MGI:1918731] | 39,70867934 | -0,5723539 | **-1,48694774** | 5,31116823 | 13,5846736 | 0,00022804 | 0,001403626 | protein_coding |
| ENSMUSG00000033278 | Ptprm | protein tyrosine phosphatase, receptor type, M [Source:MGI Symbol;Acc:MGI:102694] | 201,9071755 | -0,5724675 | **-1,48706474** | 7,65705876 | 13,0528657 | 0,000302821 | 0,001800374 | protein_coding |
| ENSMUSG00000057367 | Birc2 | baculoviral IAP repeat-containing 2 [Source:MGI Symbol;Acc:MGI:1197009] | 31,13606689 | -0,5725981 | **-1,4871994** | 4,95958519 | 11,9591 | 0,000543811 | 0,003013008 | protein_coding |
| ENSMUSG00000042742 | B630005N14Rik | RIKEN cDNA B630005N14 gene [Source:MGI Symbol;Acc:MGI:2141466] | 193,9895435 | -0,5757007 | **-1,49040116** | 7,6003637 | 16,2363756 | 5,59104E-05 | 0,000407893 | protein_coding |
| ENSMUSG00000021877 | Arf4 | ADP-ribosylation factor 4 [Source:MGI Symbol;Acc:MGI:99433] | 181,8559265 | -0,576415 | **-1,49113926** | 7,50536001 | 15,9861508 | 6,38076E-05 | 0,000458805 | protein_coding |
| ENSMUSG00000021025 | Nfkbia | nuclear factor of kappa light polypeptide gene enhancer in B cells inhibitor, alpha [Source:MGI Symbol;Acc:MGI:104741] | 200,1659695 | -0,5765117 | **-1,49123925** | 7,64564221 | 13,1468647 | 0,000288001 | 0,001727219 | protein_coding |
| ENSMUSG00000042507 | C130039O16Rik | RIKEN cDNA C130039O16 gene [Source:MGI Symbol;Acc:MGI:2685106] | 99,71106852 | -0,5765876 | **-1,49131764** | 6,63787801 | 15,939089 | 6,54138E-05 | 0,000468514 | protein_coding |
| ENSMUSG00000021947 | Cryl1 | crystallin, lambda 1 [Source:MGI Symbol;Acc:MGI:1915881] | 20,52576232 | -0,5772059 | **-1,4919569** | 4,3596456 | 12,6581933 | 0,000373923 | 0,002174852 | protein_coding |
| ENSMUSG00000029720 | Gm20605 | predicted gene 20605 [Source:MGI Symbol;Acc:MGI:5306917] | 58,19263907 | -0,5786205 | **-1,49342056** | 5,84666092 | 17,1181371 | 3,51253E-05 | 0,000266889 | protein_coding |
| ENSMUSG00000081739 | Mdm4-ps | transformed mouse 3T3 cell double minute 4, pseudogene [Source:MGI Symbol;Acc:MGI:2136991] | 23,65494053 | -0,5792475 | **-1,49406978** | 4,55839042 | 10,489837 | 0,00120033 | 0,005997281 | pseudogene |
| ENSMUSG00000025508 | Rplp2 | ribosomal protein, large P2 [Source:MGI Symbol;Acc:MGI:1914436] | 367,8112762 | -0,5796462 | **-1,49448272** | 8,52162643 | 13,595631 | 0,000226713 | 0,001397022 | protein_coding |
| ENSMUSG00000066682 | Pilrb2 | paired immunoglobin-like type 2 receptor beta 2 [Source:MGI Symbol;Acc:MGI:2450535] | 12,32659209 | -0,5806024 | **-1,49547355** | 3,61790828 | 6,03137582 | 0,014053775 | 0,047686481 | protein_coding |
| ENSMUSG00000002111 | Sfpi1 | SFFV proviral integration 1 [Source:MGI Symbol;Acc:MGI:98282] | 605,8764547 | -0,5810999 | **-1,4959893** | 9,24262547 | 10,3694143 | 0,001281202 | 0,006349375 | protein_coding |
| ENSMUSG00000029455 | Aldh2 | aldehyde dehydrogenase 2, mitochondrial [Source:MGI Symbol;Acc:MGI:99600] | 427,2761436 | -0,5814082 | **-1,49630907** | 8,73868432 | 13,6398221 | 0,000221438 | 0,00136913 | protein_coding |
| ENSMUSG00000057069 | Ero1lb | ERO1-like beta (S. cerevisiae) [Source:MGI Symbol;Acc:MGI:1914725] | 40,23428191 | -0,5814557 | **-1,49635831** | 5,33112934 | 17,5503581 | 2,79798E-05 | 0,0002188 | protein_coding |
| ENSMUSG00000029461 | Fam168a | family with sequence similarity 168, member A [Source:MGI Symbol;Acc:MGI:2442372] | 77,00587388 | -0,5816059 | **-1,49651414** | 6,26363663 | 18,8152088 | 1,44014E-05 | 0,000121265 | protein_coding |
| ENSMUSG00000022378 | Fam49b | family with sequence similarity 49, member B [Source:MGI Symbol;Acc:MGI:1923520] | 307,0106539 | -0,5821232 | **-1,49705081** | 8,26216464 | 14,0183668 | 0,000181034 | 0,001149056 | protein_coding |
| ENSMUSG00000051978 | Erich1 | glutamate-rich 1 [Source:MGI Symbol;Acc:MGI:3588201] | 9,234055465 | -0,5824726 | **-1,49741342** | 3,2005521 | 6,78022714 | 0,009217316 | 0,033949263 | protein_coding |
| ENSMUSG00000022283 | Pabpc1 | poly(A) binding protein, cytoplasmic 1 [Source:MGI Symbol;Acc:MGI:1349722] | 1324,535714 | -0,5827958 | **-1,49774896** | 10,3711626 | 8,28149794 | 0,004005107 | 0,016834015 | protein_coding |
| ENSMUSG00000045962 | Wnk1 | WNK lysine deficient protein kinase 1 [Source:MGI Symbol;Acc:MGI:2442092] | 1194,044165 | -0,5834067 | **-1,49838332** | 10,2215595 | 8,86182609 | 0,002911961 | 0,01288175 | protein_coding |
| ENSMUSG00000031708 | Tecr | trans-2,3-enoyl-CoA reductase [Source:MGI Symbol;Acc:MGI:1915408] | 113,4754151 | -0,5835755 | **-1,49855857** | 6,8230408 | 17,9007496 | 2,32729E-05 | 0,000186234 | protein_coding |
| ENSMUSG00000030272 | Camk1 | calcium/calmodulin-dependent protein kinase I [Source:MGI Symbol;Acc:MGI:1098535] | 174,7526175 | -0,5838428 | **-1,49883632** | 7,44565629 | 16,8322123 | 4,08343E-05 | 0,000305201 | protein_coding |
| ENSMUSG00000032312 | Csk | c-src tyrosine kinase [Source:MGI Symbol;Acc:MGI:88537] | 276,8452908 | -0,5860539 | **-1,50113516** | 8,11219409 | 14,80629 | 0,000119137 | 0,000796691 | protein_coding |
| ENSMUSG00000062328 | Rpl17 | ribosomal protein L17 [Source:MGI Symbol;Acc:MGI:2448270] | 548,8395541 | -0,5882037 | **-1,50337374** | 9,09983259 | 11,3836137 | 0,000740948 | 0,003969185 | protein_coding |
| ENSMUSG00000004319 | Clcn3 | chloride channel 3 [Source:MGI Symbol;Acc:MGI:103555] | 96,14559963 | -0,5893526 | **-1,50457148** | 6,58419737 | 20,5845029 | 5,70561E-06 | 5,25021E-05 | protein_coding |
| ENSMUSG00000015488 | Cacfd1 | calcium channel flower domain containing 1 [Source:MGI Symbol;Acc:MGI:1924317] | 27,20998556 | -0,589419 | **-1,50464068** | 4,75697384 | 14,4100004 | 0,000147019 | 0,0009575 | protein_coding |
| ENSMUSG00000040818 | Fam116a | family with sequence similarity 116, member A [Source:MGI Symbol;Acc:MGI:2442980] | 80,24269358 | -0,5897883 | **-1,50502585** | 6,32501922 | 18,8149361 | 1,44035E-05 | 0,000121265 | protein_coding |
| ENSMUSG00000060636 | Rpl35a | ribosomal protein L35A [Source:MGI Symbol;Acc:MGI:1928894] | 303,5993273 | -0,5913204 | **-1,50662502** | 8,24519594 | 14,8837888 | 0,000114341 | 0,000767454 | protein_coding |
| ENSMUSG00000042333 | Tnfrsf14 | tumor necrosis factor receptor superfamily, member 14 (herpesvirus entry mediator) [Source:MGI Symbol;Acc:MGI:2675 | 21,21013684 | -0,5917457 | **-1,50706924** | 4,3988043 | 8,33310351 | 0,00389291 | 0,016444213 | protein_coding |
| ENSMUSG00000031828 | Klhl36 | kelch-like 36 (Drosophila) [Source:MGI Symbol;Acc:MGI:2385305] | 17,2664513 | -0,5929674 | **-1,50834598** | 4,1002276 | 10,5861392 | 0,001139387 | 0,005734513 | protein_coding |
| ENSMUSG00000021990 | Spata13 | spermatogenesis associated 13 [Source:MGI Symbol;Acc:MGI:104838] | 31,2544392 | -0,5933289 | **-1,508724** | 4,95747801 | 15,2258005 | 9,53912E-05 | 0,000653406 | protein_coding |

| | | | | | | | | | |
|---|---|---|---|---|---|---|---|---|---|
| ENSMUSG00000034994 | Eef2 | eukaryotic translation elongation factor 2 [Source:MGI Symbol;Acc:MGI:95288] | 2508,870584 | -0,5940591 | **-1,50948781** | 11,2927569 | 6,76679605 | 0,009286941 | 0,0341599 | protein_coding |
| ENSMUSG00000071708 | Sms | spermine synthase [Source:MGI Symbol;Acc:MGI:109490] | 65,59136895 | -0,5950991 | **-1,51057633** | 6,03419949 | 17,8464915 | 2,3946E-05 | 0,000190925 | protein_coding |
| ENSMUSG00000051355 | Commd1 | COMM domain containing 1 [Source:MGI Symbol;Acc:MGI:109474] | 157,12118 | -0,5954702 | **-1,51096498** | 7,29758403 | 17,8307166 | 2,41454E-05 | 0,000192259 | protein_coding |
| ENSMUSG00000037344 | Slc12a9 | solute carrier family 12 (potassium/chloride transporters), member 9 [Source:MGI Symbol;Acc:MGI:1933532] | 51,24020978 | -0,5961096 | **-1,51163472** | 5,67084803 | 14,1882608 | 0,000165399 | 0,001062093 | protein_coding |
| ENSMUSG00000022312 | Eif3h | eukaryotic translation initiation factor 3, subunit H [Source:MGI Symbol;Acc:MGI:1915385] | 193,1204743 | -0,5961801 | **-1,51170861** | 7,59221286 | 18,4380382 | 1,75519E-05 | 0,000144995 | protein_coding |
| ENSMUSG00000008668 | Rps18 | ribosomal protein S18 [Source:MGI Symbol;Acc:MGI:98146] | 527,9129607 | -0,5963064 | **-1,51184102** | 9,0436603 | 11,8021289 | 0,00059163 | 0,003250121 | protein_coding |
| ENSMUSG00000043323 | Fbrsl1 | fibrosin-like 1 [Source:MGI Symbol;Acc:MGI:1920907] | 98,41860168 | -0,5971786 | **-1,51275527** | 6,61263375 | 20,181334 | 7,04369E-06 | 6,34336E-05 | protein_coding |
| ENSMUSG00000038005 | 2700029M09Rik | RIKEN cDNA 2700029M09 gene [Source:MGI Symbol;Acc:MGI:1919862] | 70,89314718 | -0,5973718 | **-1,51295788** | 6,14693242 | 19,8157372 | 8,52777E-06 | 7,53172E-05 | protein_coding |
| ENSMUSG00000075232 | Amd1 | S-adenosylmethionine decarboxylase 1 [Source:MGI Symbol;Acc:MGI:88004] | 316,0445487 | -0,5976337 | **-1,51323257** | 8,30425629 | 14,3003183 | 0,000155839 | 0,001008956 | protein_coding |
| ENSMUSG00000015882 | Lcorl | ligand dependent nuclear receptor corepressor-like [Source:MGI Symbol;Acc:MGI:2651932] | 29,08156748 | -0,597908 | **-1,51352023** | 4,85978657 | 16,0121609 | 6,29369E-05 | 0,000453435 | protein_coding |
| ENSMUSG00000038274 | Fau | Finkel-Biskis-Reilly murine sarcoma virus (FBR-MuSV) ubiquitously expressed (fox derived) [Source:MGI Symbol;Acc:M | 73,79411666 | -0,5986194 | **-1,51426674** | 6,19843681 | 20,7223284 | 5,30933E-06 | 4,93099E-05 | protein_coding |
| ENSMUSG00000032855 | Pkd1 | polycystic kidney disease 1 homolog [Source:MGI Symbol;Acc:MGI:97603] | 103,1018533 | -0,5991548 | **-1,51482883** | 6,68506545 | 19,7470225 | 8,83993E-06 | 7,78864E-05 | protein_coding |
| ENSMUSG00000051906 | Cd209f | CD209f antigen [Source:MGI Symbol;Acc:MGI:1916392] | 481,4102401 | -0,599503 | **-1,51519455** | 8,91166028 | 12,8942815 | 0,000329588 | 0,001943735 | protein_coding |
| ENSMUSG00000032115 | Hyou1 | hypoxia up-regulated 1 [Source:MGI Symbol;Acc:MGI:108030] | 174,3616334 | -0,6001442 | **-1,51586803** | 7,4449694 | 17,6767676 | 2,61805E-05 | 0,000205993 | protein_coding |
| ENSMUSG00000018909 | Arrb1 | arrestin, beta 1 [Source:MGI Symbol;Acc:MGI:99473] | 459,1595394 | -0,6005658 | **-1,51631115** | 8,84238377 | 13,1479054 | 0,000287841 | 0,001727202 | protein_coding |
| ENSMUSG00000024177 | Nme4 | NME/NM23 nucleoside diphosphate kinase 4 [Source:MGI Symbol;Acc:MGI:1931148] | 33,1699349 | -0,6008335 | **-1,51659251** | 5,04773065 | 16,7881869 | 4,17927E-05 | 0,000311306 | protein_coding |
| ENSMUSG00000030956 | Fam53b | family with sequence similarity 53, member B [Source:MGI Symbol;Acc:MGI:1925188] | 69,29539132 | -0,6015428 | **-1,51733832** | 6,10898474 | 18,4260729 | 1,76625E-05 | 0,000145689 | protein_coding |
| ENSMUSG00000021322 | Aoah | acyloxyacyl hydrolase [Source:MGI Symbol;Acc:MGI:1350928] | 339,3939071 | -0,6016301 | **-1,51743016** | 8,40684238 | 13,5236105 | 0,000235581 | 0,001444378 | protein_coding |
| ENSMUSG00000061787 | Rps17 | ribosomal protein S17 [Source:MGI Symbol;Acc:MGI:1309526] | 372,1895294 | -0,6025579 | **-1,51840633** | 8,53920827 | 14,2408687 | 0,000160839 | 0,001035232 | protein_coding |
| ENSMUSG00000059291 | Rpl11 | ribosomal protein L11 [Source:MGI Symbol;Acc:MGI:1914275] | 260,9843621 | -0,6033522 | **-1,51924256** | 8,02653831 | 17,7720335 | 2,49017E-05 | 0,000197256 | protein_coding |
| ENSMUSG00000034832 | Tet3 | tet methylcytosine dioxygenase 3 [Source:MGI Symbol;Acc:MGI:2446229] | 164,5667222 | -0,6035776 | **-1,51947991** | 7,35933661 | 17,7421495 | 2,52959E-05 | 0,000199947 | protein_coding |
| ENSMUSG00000064357 | mt-Atp6 | mitochondrially encoded ATP synthase 6 [Source:MGI Symbol;Acc:MGI:99927] | 1854,867037 | -0,6038211 | **-1,51973637** | 10,8571332 | 7,93438307 | 0,004850416 | 0,019672027 | protein_coding |
| ENSMUSG00000053317 | Sec61b | Sec61 beta subunit [Source:MGI Symbol;Acc:MGI:1913462] | 49,77128237 | -0,6049188 | **-1,52089315** | 5,63075073 | 16,2283475 | 5,61476E-05 | 0,000409081 | protein_coding |
| ENSMUSG00000062006 | Rpl34 | ribosomal protein L34 [Source:MGI Symbol;Acc:MGI:1915686] | 308,1800243 | -0,6052866 | **-1,52128089** | 8,2665464 | 15,2475301 | 9,43E-05 | 0,000646738 | protein_coding |
| ENSMUSG00000060938 | Rpl26 | ribosomal protein L26 [Source:MGI Symbol;Acc:MGI:106022] | 564,9674119 | -0,6059727 | **-1,5220046** | 9,14170362 | 11,9581796 | 0,00054408 | 0,003013008 | protein_coding |
| ENSMUSG00000040687 | Madd | MAP-kinase activating death domain [Source:MGI Symbol;Acc:MGI:2444672] | 164,6348763 | -0,6063751 | **-1,52242913** | 7,36074919 | 18,9457622 | 1,34488E-05 | 0,000113663 | protein_coding |
| ENSMUSG00000030156 | Cd69 | CD69 antigen [Source:MGI Symbol;Acc:MGI:88343] | 13,64488667 | -0,6064116 | **-1,52246763** | 3,7853984 | 7,95957474 | 0,004783365 | 0,019479182 | protein_coding |
| ENSMUSG00000030793 | Pycard | PYD and CARD domain containing [Source:MGI Symbol;Acc:MGI:1931465] | 123,7528696 | -0,6066951 | **-1,52276683** | 6,94829169 | 13,2118796 | 0,00027818 | 0,001675639 | protein_coding |
| ENSMUSG00000024851 | Pitpnm1 | phosphatidylinositol transfer protein, membrane-associated 1 [Source:MGI Symbol;Acc:MGI:1197524] | 84,89776831 | -0,6070078 | **-1,52309695** | 6,40194007 | 22,1315973 | 2,54585E-06 | 2,54427E-05 | protein_coding |
| ENSMUSG00000097096 | | membrane-associated phosphatidylinositol transfer protein 1 isoform 1 [Source:RefSeq peptide;Acc:NP_032877] | 84,89776831 | -0,6070102 | **-1,52309949** | 6,40194626 | 22,107434 | 2,57809E-06 | 2,57037E-05 | protein_coding |
| ENSMUSG00000037731 | Themis2 | thymocyte selection associated family member 2 [Source:MGI Symbol;Acc:MGI:2446213] | 99,85969335 | -0,6071116 | **-1,52320658** | 6,64106363 | 19,5962988 | 9,56543E-06 | 8,33831E-05 | protein_coding |
| ENSMUSG00000026116 | Tmem131 | transmembrane protein 131 [Source:MGI Symbol;Acc:MGI:1927110] | 191,0045906 | -0,6072858 | **-1,52339051** | 7,57744632 | 19,533685 | 9,88413E-06 | 8,58474E-05 | protein_coding |
| ENSMUSG00000024807 | Syvn1 | synovial apoptosis inhibitor 1, synoviolin [Source:MGI Symbol;Acc:MGI:1921376] | 91,89354084 | -0,6073228 | **-1,52342953** | 6,51845506 | 19,6938296 | 9,08944E-06 | 7,98287E-05 | protein_coding |
| ENSMUSG00000074994 | Qser1 | glutamine and serine rich 1 [Source:MGI Symbol;Acc:MGI:2138986] | 38,15874756 | -0,6074845 | **-1,52360034** | 5,25121067 | 18,8067817 | 1,44652E-05 | 0,000121598 | protein_coding |
| ENSMUSG00000019471 | Cdc37 | cell division cycle 37 [Source:MGI Symbol;Acc:MGI:109531] | 263,8233497 | -0,607714 | **-1,52384274** | 8,04224024 | 17,6853362 | 2,60628E-05 | 0,000205565 | protein_coding |
| ENSMUSG00000093445 | Lrch4 | leucine-rich repeats and calponin homology (CH) domain containing 4 [Source:MGI Symbol;Acc:MGI:1917193] | 138,3336523 | -0,6082143 | **-1,52437128** | 7,1094939 | 17,3676411 | 3,08026E-05 | 0,00023833 | protein_coding |
| ENSMUSG00000014867 | Surf4 | surfeit gene 4 [Source:MGI Symbol;Acc:MGI:98445] | 346,9331629 | -0,6084096 | **-1,52457764** | 8,43833276 | 13,1938957 | 0,000280862 | 0,001689018 | protein_coding |
| ENSMUSG00000058600 | Rpl30 | ribosomal protein L30 [Source:MGI Symbol;Acc:MGI:98037] | 336,5038618 | -0,6084924 | **-1,52466509** | 8,39356538 | 15,0084781 | 0,000107029 | 0,00072365 | protein_coding |
| ENSMUSG00000026675 | Hsd17b7 | hydroxysteroid (17-beta) dehydrogenase 7 [Source:MGI Symbol;Acc:MGI:1330808] | 20,26472345 | -0,6089823 | **-1,52518294** | 4,33416274 | 8,13327707 | 0,004346013 | 0,017999707 | protein_coding |
| ENSMUSG00000034522 | Zfp395 | zinc finger protein 395 [Source:MGI Symbol;Acc:MGI:2682318] | 21,77302041 | -0,6090707 | **-1,52527641** | 4,42141295 | 13,3226505 | 0,000262219 | 0,001589958 | protein_coding |
| ENSMUSG00000026425 | Srgap2 | SLIT-ROBO Rho GTPase activating protein 2 [Source:MGI Symbol;Acc:MGI:109605] | 354,2905946 | -0,6098081 | **-1,52605616** | 8,46839354 | 14,4178367 | 0,000146409 | 0,00095409 | protein_coding |
| ENSMUSG00000006411 | Pvrl4 | poliovirus receptor-related 4 [Source:MGI Symbol;Acc:MGI:1918990] | 18,26727315 | -0,6101473 | **-1,52641508** | 4,17415814 | 11,1568919 | 0,000837201 | 0,004394804 | protein_coding |
| ENSMUSG00000059714 | Flot1 | flotillin 1 [Source:MGI Symbol;Acc:MGI:1100500] | 81,07870767 | -0,6102948 | **-1,52657109** | 6,33781778 | 20,5794133 | 5,7208E-06 | 5,25539E-05 | protein_coding |
| ENSMUSG00000058546 | Rpl23a | ribosomal protein L23A [Source:MGI Symbol;Acc:MGI:3040672] | 502,5782121 | -0,6115136 | **-1,52786127** | 8,97269969 | 12,1707388 | 0,000485451 | 0,002735204 | protein_coding |
| ENSMUSG00000039232 | Stx11 | syntaxin 11 [Source:MGI Symbol;Acc:MGI:1921982] | 36,95509761 | -0,6136521 | **-1,53012778** | 5,2076294 | 12,5824447 | 0,000389387 | 0,002258815 | protein_coding |
| ENSMUSG00000042524 | 41549 | Sad1 and UNC84 domain containing 2 [Source:MGI Symbol;Acc:MGI:2443011] | 173,549339 | -0,6138962 | **-1,53038668** | 7,43729321 | 20,9335759 | 4,75487E-06 | 4,47114E-05 | protein_coding |
| ENSMUSG00000032353 | Tmed3 | transmembrane emp24 domain containing 3 [Source:MGI Symbol;Acc:MGI:1913361] | 76,40951294 | -0,6139356 | **-1,53042842** | 6,2525664 | 17,4110026 | 3,01078E-05 | 0,000233447 | protein_coding |
| ENSMUSG00000041225 | Arhgap12 | Rho GTPase activating protein 12 [Source:MGI Symbol;Acc:MGI:1922665] | 150,946354 | -0,6141181 | **-1,53062201** | 7,23627545 | 20,1395818 | 7,19913E-06 | 6,47037E-05 | protein_coding |
| ENSMUSG00000022436 | Sh3bp1 | SH3-domain binding protein 1 [Source:MGI Symbol;Acc:MGI:104603] | 174,6448626 | -0,6142649 | **-1,5307778** | 7,44597887 | 16,5131348 | 4,83142E-05 | 0,0003565 | protein_coding |
| ENSMUSG00000020393 | Kremen1 | kringle containing transmembrane protein 1 [Source:MGI Symbol;Acc:MGI:1933988] | 125,2462098 | -0,6145458 | **-1,53107595** | 6,96927761 | 18,5216967 | 1,67981E-05 | 0,000139396 | protein_coding |
| ENSMUSG00000026031 | Cflar | CASP8 and FADD-like apoptosis regulator [Source:MGI Symbol;Acc:MGI:1336166] | 375,9693606 | -0,6145963 | **-1,5311295** | 8,55434007 | 13,7531941 | 0,000208466 | 0,001299407 | protein_coding |
| ENSMUSG00000039637 | Coro7 | coronin 7 [Source:MGI Symbol;Acc:MGI:1926135] | 164,2758062 | -0,6158019 | **-1,53240949** | 7,35798722 | 20,8402644 | 4,99223E-06 | 4,67203E-05 | protein_coding |
| ENSMUSG00000030786 | Itgam | integrin alpha M [Source:MGI Symbol;Acc:MGI:96607] | 1875,691757 | -0,6169301 | **-1,53360838** | 10,8731542 | 6,40263548 | 0,011395108 | 0,040490962 | protein_coding |
| ENSMUSG00000011884 | Gltp | glycolipid transfer protein [Source:MGI Symbol;Acc:MGI:1929253] | 285,9278309 | -0,617175 | **-1,53386873** | 8,1584665 | 17,2670117 | 3,24777E-05 | 0,000249359 | protein_coding |
| ENSMUSG00000028706 | Nsun4 | NOL1/NOP2/Sun domain family, member 4 [Source:MGI Symbol;Acc:MGI:1919431] | 15,8217012 | -0,6173764 | **-1,53408284** | 3,98166853 | 12,5600607 | 0,000394079 | 0,002281215 | protein_coding |
| ENSMUSG00000070808 | Gltscr1 | glioma tumor suppressor candidate region gene 1 [Source:MGI Symbol;Acc:MGI:2154263] | 44,59907381 | -0,617704 | **-1,53443125** | 5,4643395 | 17,4714057 | 2,91662E-05 | 0,000226626 | protein_coding |
| ENSMUSG00000091811 | Prr24 | proline rich 24 [Source:MGI Symbol;Acc:MGI:1913550] | 14,16028884 | -0,6178726 | **-1,53461057** | 3,79471359 | 11,0190596 | 0,000901798 | 0,004697988 | protein_coding |
| ENSMUSG00000034116 | Vav1 | vav 1 oncogene [Source:MGI Symbol;Acc:MGI:98923] | 630,9608374 | -0,6184676 | **-1,53524361** | 9,30127526 | 12,0952315 | 0,000505509 | 0,002833879 | protein_coding |
| ENSMUSG00000021156 | Zmynd11 | zinc finger, MYND domain containing 11 [Source:MGI Symbol;Acc:MGI:1913755] | 100,3044069 | -0,6185102 | **-1,53528895** | 6,64583144 | 23,24054 | 1,42952E-06 | 1,49868E-05 | protein_coding |
| ENSMUSG00000026991 | Pkp4 | plakophilin 4 [Source:MGI Symbol;Acc:MGI:109281] | 158,6459009 | -0,6201285 | **-1,53701211** | 7,30992343 | 16,6003635 | 4,61422E-05 | 0,00034185 | protein_coding |
| ENSMUSG00000052270 | Fpr2 | formyl peptide receptor 2 [Source:MGI Symbol;Acc:MGI:1278319] | 76,1066342 | -0,6202493 | **-1,53714074** | 6,25354402 | 17,1597297 | 3,43645E-05 | 0,000261832 | protein_coding |
| ENSMUSG00000092274 | Neat1 | nuclear paraspeckle assembly transcript 1 (non-protein coding) [Source:MGI Symbol;Acc:MGI:1914211] | 1367,011848 | -0,6202843 | **-1,53717807** | 10,4166549 | 8,89053649 | 0,002866525 | 0,012709649 | non_coding |
| ENSMUSG00000000088 | Cox5a | cytochrome c oxidase, subunit Va [Source:MGI Symbol;Acc:MGI:88474] | 289,6560515 | -0,620607 | **-1,53752194** | 8,17787092 | 16,4535244 | 4,98574E-05 | 0,000367393 | protein_coding |
| ENSMUSG00000095253 | Zfp799 | zinc finger protein 799 [Source:MGI Symbol;Acc:MGI:2443934] | 11,93870955 | -0,6210994 | **-1,53804675** | 3,56073795 | 9,17380114 | 0,002455041 | 0,01115531 | protein_coding |
| ENSMUSG00000047180 | Neurl3 | neuralized homolog 3 homolog (Drosophila) [Source:MGI Symbol;Acc:MGI:2429944] | 246,160896 | -0,6220466 | **-1,53905691** | 7,94250904 | 16,1606214 | 5,81913E-05 | 0,000421178 | protein_coding |
| ENSMUSG00000034120 | Srsf2 | serine/arginine-rich splicing factor 2 [Source:MGI Symbol;Acc:MGI:98284] | 235,4427003 | -0,6227549 | **-1,53981271** | 7,87825645 | 17,1279549 | 3,49442E-05 | 0,000265881 | protein_coding |
| ENSMUSG00000028233 | Tgs1 | trimethylguanosine synthase homolog (S. cerevisiae) [Source:MGI Symbol;Acc:MGI:2151797] | 75,88911976 | -0,6227808 | **-1,53984032** | 6,24195137 | 20,733225 | 5,2792E-06 | 4,90716E-05 | protein_coding |
| ENSMUSG00000032089 | Il10ra | interleukin 10 receptor, alpha [Source:MGI Symbol;Acc:MGI:96538] | 196,1085365 | -0,6228746 | **-1,53994053** | 7,61370137 | 16,7048114 | 4,36701E-05 | 0,00032463 | protein_coding |
| ENSMUSG00000025264 | Tsr2 | TSR2, 20S rRNA accumulation, homolog (S. cerevisiae) [Source:MGI Symbol;Acc:MGI:1916749] | 15,07983054 | -0,6230316 | **-1,54010809** | 3,90732673 | 11,9859257 | 0,000536039 | 0,002983514 | protein_coding |
| ENSMUSG00000058603 | Rpl28-ps1 | ribosomal protein L28, pseudogene 1 [Source:MGI Symbol;Acc:MGI:3705349] | 342,8450562 | -0,623348 | **-1,54044585** | 8,4201387 | 15,9997995 | 6,33492E-05 | 0,000456106 | pseudogene |
| ENSMUSG00000057322 | Rpl38 | ribosomal protein L38 [Source:MGI Symbol;Acc:MGI:1914921] | 194,6768954 | -0,6249984 | **-1,54220909** | 7,60318612 | 18,7888422 | 1,46019E-05 | 0,000122466 | protein_coding |

| | | | | | | | | | | |
|---|---|---|---|---|---|---|---|---|---|---|
| ENSMUSG00000060860 | Ube2s | ubiquitin-conjugating enzyme E2S [Source:MGI Symbol;Acc:MGI:1925141] | 101,9365498 | -0,6250728 | **-1,54228862** | 6,66928408 | 21,3559137 | 3,81442E-06 | 3,66338E-05 | protein_coding |
| ENSMUSG00000049517 | Rps23 | ribosomal protein S23 [Source:MGI Symbol;Acc:MGI:1913725] | 142,7382043 | -0,6251369 | **-1,54235714** | 7,15595535 | 19,3013365 | 1,11628E-05 | 9,57423E-05 | protein_coding |
| ENSMUSG00000017734 | Dbndd2 | dysbindin (dystrobrevin binding protein 1) domain containing 2 [Source:MGI Symbol;Acc:MGI:106562] | 24,87209556 | -0,6258054 | **-1,54307203** | 4,64098298 | 13,0009285 | 0,000311337 | 0,001846009 | protein_coding |
| ENSMUSG00000053175 | Bcl3 | B cell leukemia/lymphoma 3 [Source:MGI Symbol;Acc:MGI:88140] | 84,02128781 | -0,6260277 | **-1,54330976** | 6,38971656 | 18,0428915 | 2,15984E-05 | 0,000174358 | protein_coding |
| ENSMUSG00000067288 | Rps28 | ribosomal protein S28 [Source:MGI Symbol;Acc:MGI:1859516] | 184,7953031 | -0,6265165 | **-1,54383282** | 7,52704132 | 20,7508766 | 5,23076E-06 | 4,87741E-05 | protein_coding |
| ENSMUSG00000048118 | Arid4a | AT rich interactive domain 4A (RBP1-like) [Source:MGI Symbol;Acc:MGI:2444354] | 57,7326638 | -0,6268244 | **-1,54416226** | 5,84540345 | 21,2896601 | 3,94856E-06 | 3,78228E-05 | protein_coding |
| ENSMUSG00000097245 | | | 7,68424275 | -0,6278136 | **-1,54522143** | 2,91517891 | 6,67558792 | 0,009774227 | 0,035701273 | pseudogene |
| ENSMUSG00000039461 | Tcta | T cell leukemia translocation altered gene [Source:MGI Symbol;Acc:MGI:1918829] | 22,86254745 | -0,6294224 | **-1,54694557** | 4,50250404 | 12,4787968 | 0,000411597 | 0,002362706 | protein_coding |
| ENSMUSG00000036206 | Sh3bp4 | SH3-domain binding protein 4 [Source:MGI Symbol;Acc:MGI:2138297] | 40,29768485 | -0,6302053 | **-1,54778528** | 5,33409903 | 15,340773 | 8,97582E-05 | 0,000621016 | protein_coding |
| ENSMUSG00000085385 | 9430008C03Rik | RIKEN cDNA 9430008C03 gene [Source:MGI Symbol;Acc:MGI:1915358] | 26,3023494 | -0,6308083 | **-1,54843229** | 4,71022937 | 15,3978272 | 8,70884E-05 | 0,000605431 | lincRNA |
| ENSMUSG00000074884 | Serf2 | small EDRK-rich factor 2 [Source:MGI Symbol;Acc:MGI:1337041] | 180,4261735 | -0,6311769 | **-1,54882796** | 7,49342579 | 18,9815016 | 1,31992E-05 | 0,000111812 | protein_coding |
| ENSMUSG00000078193 | Gm2000 | predicted gene 2000 [Source:MGI Symbol;Acc:MGI:3780170] | 187,3437978 | -0,6316226 | **-1,54930654** | 7,54780633 | 18,4034731 | 1,78732E-05 | 0,000147096 | protein_coding |
| ENSMUSG00000034271 | Jdp2 | Jun dimerization protein 2 [Source:MGI Symbol;Acc:MGI:1932093] | 31,75113015 | -0,6323835 | **-1,55012381** | 4,98143899 | 15,557547 | 8,00315E-05 | 0,000560782 | protein_coding |
| ENSMUSG00000038205 | Prkab2 | protein kinase, AMP-activated, beta 2 non-catalytic subunit [Source:MGI Symbol;Acc:MGI:1336185] | 25,30086607 | -0,6331273 | **-1,55092326** | 4,66224558 | 18,3244075 | 1,86305E-05 | 0,000152075 | protein_coding |
| ENSMUSG00000009739 | Pou6f1 | POU domain, class 6, transcription factor 1 [Source:MGI Symbol;Acc:MGI:102935] | 40,05202393 | -0,633396 | **-1,55121219** | 5,31619262 | 19,5648821 | 9,72403E-06 | 8,45906E-05 | protein_coding |
| ENSMUSG00000020738 | Sumo2 | SMT3 suppressor of mif two 3 homolog 2 (yeast) [Source:MGI Symbol;Acc:MGI:2158813] | 72,00827301 | -0,6335258 | **-1,55135174** | 6,16268845 | 15,7638455 | 7,17608E-05 | 0,000509985 | protein_coding |
| ENSMUSG00000052299 | Ltn1 | listerin E3 ubiquitin protein ligase 1 [Source:MGI Symbol;Acc:MGI:1926163] | 120,8121408 | -0,6336623 | **-1,55149845** | 6,91719519 | 22,9990694 | 1,6208E-06 | 1,68474E-05 | protein_coding |
| ENSMUSG00000052248 | Gm13476 | predicted gene 13476 [Source:MGI Symbol;Acc:MGI:3652108] | 20,05533184 | -0,6340025 | **-1,55186439** | 4,32679168 | 14,1407845 | 0,000169626 | 0,001084297 | antisense |
| ENSMUSG00000009621 | Vav2 | vav 2 oncogene [Source:MGI Symbol;Acc:MGI:102718] | 87,09164482 | -0,6344134 | **-1,55230644** | 6,44196047 | 19,2417746 | 1,15165E-05 | 9,84687E-05 | protein_coding |
| ENSMUSG00000069609 | Gm11710 | predicted gene 11710 [Source:MGI Symbol;Acc:MGI:3702658] | 48,64843451 | -0,6345122 | **-1,55241277** | 5,61399718 | 18,4207002 | 1,77123E-05 | 0,000145991 | protein_coding |
| ENSMUSG00000037563 | Rps16 | ribosomal protein S16 [Source:MGI Symbol;Acc:MGI:98118] | 327,7319035 | -0,6345929 | **-1,55249957** | 8,35468641 | 16,1691919 | 5,79286E-05 | 0,000419553 | protein_coding |
| ENSMUSG00000040943 | Tet2 | tet methylcytosine dioxygenase 2 [Source:MGI Symbol;Acc:MGI:2443298] | 70,74221665 | -0,6357859 | **-1,55378393** | 6,14223378 | 20,8183719 | 5,04962E-06 | 4,72172E-05 | protein_coding |
| ENSMUSG00000063457 | Rps15 | ribosomal protein S15 [Source:MGI Symbol;Acc:MGI:98117] | 388,5741092 | -0,6361619 | **-1,55418895** | 8,6010191 | 15,1990566 | 9,67517E-05 | 0,000661077 | protein_coding |
| ENSMUSG00000032513 | Gorasp1 | golgi reassembly stacking protein 1 [Source:MGI Symbol;Acc:MGI:1921748] | 39,25219019 | -0,6363163 | **-1,55435532** | 5,28772178 | 16,7915951 | 4,17177E-05 | 0,000310958 | protein_coding |
| ENSMUSG00000010406 | Mrpl52 | mitochondrial ribosomal protein L52 [Source:MGI Symbol;Acc:MGI:1916086] | 30,90416482 | -0,6366375 | **-1,55470138** | 4,94286596 | 17,6000839 | 2,72576E-05 | 0,000213761 | protein_coding |
| ENSMUSG00000004798 | Ulk2 | unc-51 like kinase 2 [Source:MGI Symbol;Acc:MGI:1352758] | 77,68576218 | -0,6366582 | **-1,55472369** | 6,276135 | 20,1233198 | 7,2606E-06 | 6,50671E-05 | protein_coding |
| ENSMUSG00000027540 | Ptpn1 | protein tyrosine phosphatase, non-receptor type 1 [Source:MGI Symbol;Acc:MGI:97805] | 331,6411993 | -0,6367849 | **-1,55486023** | 8,3729784 | 14,1099397 | 0,00017243 | 0,001097619 | protein_coding |
| ENSMUSG00000009585 | Apobec3 | apolipoprotein B mRNA editing enzyme, catalytic polypeptide 3 [Source:MGI Symbol;Acc:MGI:1933111] | 69,62732811 | -0,6376069 | **-1,55574638** | 6,12031688 | 17,3464944 | 3,11472E-05 | 0,000240827 | protein_coding |
| ENSMUSG00000011832 | Evi5l | ecotropic viral integration site 5 like [Source:MGI Symbol;Acc:MGI:2442167] | 21,80853733 | -0,6376835 | **-1,55582898** | 4,44420754 | 14,5713921 | 0,000134948 | 0,000886659 | protein_coding |
| ENSMUSG00000024608 | Rps14 | ribosomal protein S14 [Source:MGI Symbol;Acc:MGI:98107] | 461,4061975 | -0,6384916 | **-1,55670071** | 8,84926951 | 13,5847347 | 0,000228032 | 0,001403626 | protein_coding |
| ENSMUSG00000021720 | Rnf180 | ring finger protein 180 [Source:MGI Symbol;Acc:MGI:1919066] | 8,748673824 | -0,6389654 | **-1,55721203** | 3,11413032 | 6,89658138 | 0,008636073 | 0,032118916 | protein_coding |
| ENSMUSG00000064356 | mt-Atp8 | mitochondrially encoded ATP synthase 8 [Source:MGI Symbol;Acc:MGI:99926] | 414,2894567 | -0,6404802 | **-1,55884789** | 8,69481391 | 16,4846335 | 4,9046E-05 | 0,000361656 | protein_coding |
| ENSMUSG00000005986 | Ankrd13d | ankyrin repeat domain 13 family, member D [Source:MGI Symbol;Acc:MGI:1915673] | 25,95366303 | -0,6405271 | **-1,55889861** | 4,68938164 | 15,7497342 | 7,22981E-05 | 0,000513471 | protein_coding |
| ENSMUSG00000019254 | Ppp1r12c | protein phosphatase 1, regulatory (inhibitor) subunit 12C [Source:MGI Symbol;Acc:MGI:1924258] | 269,6261662 | -0,6409993 | **-1,55940897** | 8,07361117 | 16,8498456 | 4,04566E-05 | 0,000302791 | protein_coding |
| ENSMUSG00000020719 | Ddx5 | DEAD (Asp-Glu-Ala-Asp) box polypeptide 5 [Source:MGI Symbol;Acc:MGI:105037] | 1261,99702 | -0,6430012 | **-1,56157426** | 10,3013792 | 9,6667614 | 0,001876318 | 0,008885821 | protein_coding |
| ENSMUSG00000031093 | Dock11 | dedicator of cytokinesis 11 [Source:MGI Symbol;Acc:MGI:1923224] | 141,9869905 | -0,6440092 | **-1,5626657** | 7,14926574 | 23,369483 | 1,33683E-06 | 1,41092E-05 | protein_coding |
| ENSMUSG00000040528 | Gm885 | predicted gene 885 [Source:MGI Symbol;Acc:MGI:2685731] | 137,7999413 | -0,6441744 | **-1,56284465** | 7,10642255 | 20,3107013 | 6,58314E-06 | 5,97266E-05 | protein_coding |
| ENSMUSG00000083596 | Rpl21-ps15 | ribosomal protein L21, pseudogene 15 [Source:MGI Symbol;Acc:MGI:3705426] | 62,72395373 | -0,6447833 | **-1,56350442** | 5,9614589 | 15,304929 | 9,14775E-05 | 0,000630134 | pseudogene |
| ENSMUSG00000068114 | Ccdc134 | coiled-coil domain containing 134 [Source:MGI Symbol;Acc:MGI:1923707] | 11,91197654 | -0,6448547 | **-1,56358183** | 3,56924808 | 8,25754095 | 0,004058306 | 0,017025052 | protein_coding |
| ENSMUSG00000020108 | Ddit4 | DNA-damage-inducible transcript 4 [Source:MGI Symbol;Acc:MGI:1921997] | 8,12788139 | -0,644874 | **-1,56360267** | 2,99867575 | 6,62454489 | 0,010058284 | 0,03656927 | protein_coding |
| ENSMUSG00000048578 | Mlec | malectin [Source:MGI Symbol;Acc:MGI:1924015] | 222,8316173 | -0,645202 | **-1,56395827** | 7,79852512 | 19,3803114 | 1,07106E-05 | 9,22956E-05 | protein_coding |
| ENSMUSG00000064358 | mt-Co3 | mitochondrially encoded cytochrome c oxidase III [Source:MGI Symbol;Acc:MGI:102502] | 2132,652972 | -0,6454228 | **-1,56419767** | 11,0584589 | 8,54494416 | 0,003464832 | 0,014952126 | protein_coding |
| ENSMUSG00000079426 | Arpc4 | actin related protein 2/3 complex, subunit 4 [Source:MGI Symbol;Acc:MGI:1915339] | 533,8860292 | -0,6455231 | **-1,56430636** | 9,06005502 | 13,8111211 | 0,000202136 | 0,001265453 | protein_coding |
| ENSMUSG00000024955 | Esrra | estrogen related receptor, alpha [Source:MGI Symbol;Acc:MGI:1346831] | 84,59338281 | -0,645949 | **-1,56476824** | 6,39727999 | 21,1130034 | 4,3298E-06 | 4,11163E-05 | protein_coding |
| ENSMUSG00000048997 | Atxn7l2 | ataxin 7-like 2 [Source:MGI Symbol;Acc:MGI:1919772] | 6,89056088 | -0,6471671 | **-1,56608998** | 2,76449169 | 6,32979346 | 0,011872614 | 0,041835925 | protein_coding |
| ENSMUSG00000052085 | Dock8 | dedicator of cytokinesis 8 [Source:MGI Symbol;Acc:MGI:1921396] | 243,6091113 | -0,6481926 | **-1,5672036** | 7,92752409 | 19,3786448 | 1,07199E-05 | 9,23038E-05 | protein_coding |
| ENSMUSG00000024143 | Rhoq | ras homolog gene family, member Q [Source:MGI Symbol;Acc:MGI:1931553] | 195,5903754 | -0,6491409 | **-1,56823409** | 7,61175324 | 15,9218549 | 6,60121E-05 | 0,000471877 | protein_coding |
| ENSMUSG00000023004 | Tuba1b | tubulin, alpha 1B [Source:MGI Symbol;Acc:MGI:107804] | 1064,515574 | -0,6492781 | **-1,56838323** | 10,0559548 | 11,0426699 | 0,000890385 | 0,004642935 | protein_coding |
| ENSMUSG00000024053 | Emilin2 | elastin microfibril interfacer 2 [Source:MGI Symbol;Acc:MGI:2389136] | 280,6465501 | -0,6497719 | **-1,56892008** | 8,13254409 | 17,491149 | 2,88648E-05 | 0,000224602 | protein_coding |
| ENSMUSG00000079165 | Sap25 | sin3 associated polypeptide [Source:MGI Symbol;Acc:MGI:3802945] | 20,3777273 | -0,6499459 | **-1,56910933** | 4,33393156 | 10,7744298 | 0,001029119 | 0,005259038 | protein_coding |
| ENSMUSG00000096581 | Bcl2a1d | B cell leukemia/lymphoma 2 related protein A1d [Source:MGI Symbol;Acc:MGI:1278325] | 58,66202553 | -0,650192 | **-1,56937704** | 5,8750183 | 12,7027779 | 0,000365113 | 0,002133428 | protein_coding |
| ENSMUSG00000031604 | Sc4mol | sterol-C4-methyl oxidase-like [Source:MGI Symbol;Acc:MGI:1913484] | 65,96896762 | -0,6507121 | **-1,56994288** | 6,03894883 | 8,32864378 | 0,003902478 | 0,016478297 | protein_coding |
| ENSMUSG00000075701 | Vimp | VCP-interacting membrane protein [Source:MGI Symbol;Acc:MGI:95994] | 59,95169632 | -0,6511081 | **-1,57037393** | 5,90329509 | 16,8781635 | 3,98575E-05 | 0,000299326 | protein_coding |
| ENSMUSG00000040618 | Pck2 | phosphoenolpyruvate carboxykinase 2 (mitochondrial) [Source:MGI Symbol;Acc:MGI:1860456] | 46,30181884 | -0,6516799 | **-1,57099642** | 5,51987011 | 13,8958926 | 0,00019322 | 0,001217265 | protein_coding |
| ENSMUSG00000027522 | Stx16 | syntaxin 16 [Source:MGI Symbol;Acc:MGI:1923396] | 99,78458685 | -0,6530281 | **-1,57246518** | 6,63697268 | 23,170208 | 1,48277E-06 | 1,55006E-05 | protein_coding |
| ENSMUSG00000000290 | Itgb2 | integrin beta 2 [Source:MGI Symbol;Acc:MGI:96611] | 1411,145339 | -0,6531975 | **-1,57264981** | 10,4625794 | 9,629229 | 0,00191505 | 0,009022578 | protein_coding |
| ENSMUSG00000017713 | Tha1 | threonine aldolase 1 [Source:MGI Symbol;Acc:MGI:1919026] | 8,240444063 | -0,6539005 | **-1,57341633** | 3,03188039 | 6,00533309 | 0,014262701 | 0,048291001 | protein_coding |
| ENSMUSG00000039953 | Clstn1 | calsyntenin 1 [Source:MGI Symbol;Acc:MGI:1929895] | 27,48799706 | -0,6539337 | **-1,57345254** | 4,76335068 | 20,3943119 | 6,30168E-06 | 5,74713E-05 | protein_coding |
| ENSMUSG00000028936 | Rpl22 | ribosomal protein L22 [Source:MGI Symbol;Acc:MGI:99262] | 232,8752203 | -0,6542817 | **-1,57383211** | 7,86211658 | 19,8036259 | 8,58197E-06 | 7,56743E-05 | protein_coding |
| ENSMUSG00000024858 | Adrbk1 | adrenergic receptor kinase, beta 1 [Source:MGI Symbol;Acc:MGI:87940] | 546,6426029 | -0,6555733 | **-1,57524184** | 9,09430343 | 13,7309888 | 0,000210945 | 0,001312375 | protein_coding |
| ENSMUSG00000024594 | Prrc1 | proline-rich coiled-coil 1 [Source:MGI Symbol;Acc:MGI:1916106] | 66,3769862 | -0,6563483 | **-1,57608824** | 6,04743313 | 19,3606978 | 1,08211E-05 | 9,30296E-05 | protein_coding |
| ENSMUSG00000057863 | Rpl36 | ribosomal protein L36 [Source:MGI Symbol;Acc:MGI:1860603] | 183,2529118 | -0,6571905 | **-1,57700853** | 7,51447823 | 21,6591286 | 3,25657E-06 | 3,17479E-05 | protein_coding |
| ENSMUSG00000007892 | Rplp1 | ribosomal protein, large, P1 [Source:MGI Symbol;Acc:MGI:1927099] | 1137,173982 | -0,6576063 | **-1,57746315** | 10,1509515 | 10,9627073 | 0,000929639 | 0,004817898 | protein_coding |
| ENSMUSG00000059970 | Hspa2 | heat shock protein 2 [Source:MGI Symbol;Acc:MGI:96243] | 9,663511726 | -0,657977 | **-1,57786858** | 3,2706802 | 8,50513839 | 0,00354145 | 0,015205123 | protein_coding |
| ENSMUSG00000039987 | Phtf2 | putative homeodomain transcription factor 2 [Source:MGI Symbol;Acc:MGI:1916020] | 77,78782041 | -0,659484 | **-1,57951762** | 6,28472441 | 25,8148993 | 3,75776E-07 | 4,37821E-06 | protein_coding |
| ENSMUSG00000019952 | Poc1b | POC1 centriolar protein homolog B (Chlamydomonas) [Source:MGI Symbol;Acc:MGI:1918511] | 21,84631939 | -0,6605727 | **-1,58070998** | 4,4448667 | 15,5127583 | 8,19503E-05 | 0,000572402 | protein_coding |
| ENSMUSG00000026049 | Tex30 | testis expressed 30 [Source:MGI Symbol;Acc:MGI:1922873] | 18,74681106 | -0,6620945 | **-1,58237829** | 4,22454237 | 13,3814017 | 0,000254132 | 0,001548611 | protein_coding |
| ENSMUSG00000026399 | Cd55 | CD55 antigen [Source:MGI Symbol;Acc:MGI:104850] | 352,9756039 | -0,6634906 | **-1,5839103** | 8,46355773 | 17,760938 | 2,50473E-05 | 0,000198267 | protein_coding |
| ENSMUSG00000036707 | Cab39 | calcium binding protein 39 [Source:MGI Symbol;Acc:MGI:107438] | 496,0091985 | -0,6636018 | **-1,5840323** | 8,95397768 | 15,443068 | 8,50282E-05 | 0,000592758 | protein_coding |
| ENSMUSG00000021244 | Ylpm1 | YLP motif containing 1 [Source:MGI Symbol;Acc:MGI:1926195] | 83,36714467 | -0,6636206 | **-1,584053** | 6,37952287 | 26,1722857 | 3,12272E-07 | 3,72928E-06 | protein_coding |
| ENSMUSG00000034343 | Ube2f | ubiquitin-conjugating enzyme E2F (putative) [Source:MGI Symbol;Acc:MGI:1915171] | 95,69787147 | -0,6636384 | **-1,58407249** | 6,57939322 | 19,4803194 | 1,01642E-05 | 8,80451E-05 | protein_coding |

| | | | | | | | | | | |
|---|---|---|---|---|---|---|---|---|---|---|
| ENSMUSG00000032193 | Ldlr | low density lipoprotein receptor [Source:MGI Symbol;Acc:MGI:96765] | 105,3678038 | -0,6637942 | **-1,58424363** | 6,71651605 | 6,15968997 | 0,013069411 | 0,045112668 | protein_coding |
| ENSMUSG00000025036 | Sfxn2 | sideroflexin 2 [Source:MGI Symbol;Acc:MGI:2137678] | 16,66894629 | -0,6647014 | **-1,58524009** | 4,05129847 | 14,2760156 | 0,000157864 | 0,001019064 | protein_coding |
| ENSMUSG00000031791 | Tmem38a | transmembrane protein 38A [Source:MGI Symbol;Acc:MGI:1921416] | 8,902317134 | -0,6647896 | **-1,58533709** | 3,14660297 | 8,92201946 | 0,002817528 | 0,012527794 | protein_coding |
| ENSMUSG00000027952 | Pmvk | phosphomevalonate kinase [Source:MGI Symbol;Acc:MGI:1915853] | 16,11538129 | -0,6649618 | **-1,58552629** | 3,996177 | 10,8413071 | 0,000992606 | 0,005101632 | protein_coding |
| ENSMUSG00000020143 | Dock2 | dedicator of cyto-kinesis 2 [Source:MGI Symbol;Acc:MGI:2149010] | 602,3672172 | -0,6651227 | **-1,58570315** | 9,23414192 | 14,3118146 | 0,00015489 | 0,001003996 | protein_coding |
| ENSMUSG00000062937 | Mtap | methylthioadenosine phosphorylase [Source:MGI Symbol;Acc:MGI:1914152] | 65,12444556 | -0,6655038 | **-1,5861221** | 6,02705067 | 25,3538822 | 4,77191E-07 | 5,48995E-06 | protein_coding |
| ENSMUSG00000032434 | Cmtm6 | CKLF-like MARVEL transmembrane domain containing 6 [Source:MGI Symbol;Acc:MGI:2447165] | 158,898438 | -0,6659012 | **-1,58655901** | 7,31078775 | 20,3630909 | 6,40534E-06 | 5,82578E-05 | protein_coding |
| ENSMUSG00000017664 | Slc35c2 | solute carrier family 35, member C2 [Source:MGI Symbol;Acc:MGI:2385166] | 72,43907839 | -0,6664202 | **-1,58712988** | 6,17396682 | 25,936322 | 3,52867E-07 | 4,14204E-06 | protein_coding |
| ENSMUSG00000051457 | Spn | sialophorin [Source:MGI Symbol;Acc:MGI:98384] | 43,05069532 | -0,6671778 | **-1,58796356** | 5,41569961 | 21,034169 | 4,51165E-06 | 4,26621E-05 | protein_coding |
| ENSMUSG00000032320 | Rcn2 | reticulocalbin 2 [Source:MGI Symbol;Acc:MGI:1349765] | 63,25352367 | -0,667194 | **-1,58798144** | 5,98114609 | 23,7402363 | 1,10253E-06 | 1,18412E-05 | protein_coding |
| ENSMUSG00000031668 | Eif2ak3 | eukaryotic translation initiation factor 2 alpha kinase 3 [Source:MGI Symbol;Acc:MGI:1341830] | 25,15303248 | -0,668453 | **-1,58936773** | 4,64414829 | 16,3874827 | 5,1625E-05 | 0,000378641 | protein_coding |
| ENSMUSG00000032041 | Tirap | toll-interleukin 1 receptor (TIR) domain-containing adaptor protein [Source:MGI Symbol;Acc:MGI:2152213] | 44,11680406 | -0,6684764 | **-1,5893935** | 5,4602096 | 16,7498799 | 4,2645E-05 | 0,000317439 | protein_coding |
| ENSMUSG00000032601 | Prkar2a | protein kinase, cAMP dependent regulatory, type II alpha [Source:MGI Symbol;Acc:MGI:108025] | 118,3208614 | -0,6693915 | **-1,59040198** | 6,88602641 | 22,6743142 | 1,91916E-06 | 1,96397E-05 | protein_coding |
| ENSMUSG00000073422 | H2-Ke6 | H2-K region expressed gene 6 [Source:MGI Symbol;Acc:MGI:95911] | 13,04042087 | -0,6697407 | **-1,59078702** | 3,69671286 | 12,4252629 | 0,000423565 | 0,002421284 | protein_coding |
| ENSMUSG00000079197 | Psme2 | proteasome (prosome, macropain) 28 subunit, beta [Source:MGI Symbol;Acc:MGI:1096365] | 274,8200343 | -0,6710242 | **-1,59220285** | 8,10201148 | 19,3269166 | 1,10143E-05 | 9,46159E-05 | protein_coding |
| ENSMUSG00000008393 | Carhsp1 | calcium regulated heat stable protein 1 [Source:MGI Symbol;Acc:MGI:1196368] | 39,16012192 | -0,6717384 | **-1,59299127** | 5,28807435 | 20,5959213 | 5,67168E-06 | 5,22775E-05 | protein_coding |
| ENSMUSG00000021134 | Srsf5 | serine/arginine-rich splicing factor 5 [Source:MGI Symbol;Acc:MGI:98287] | 431,2315316 | -0,6722746 | **-1,59358343** | 8,75160028 | 15,4419069 | 8,50805E-05 | 0,000592758 | protein_coding |
| ENSMUSG00000060743 | H3f3a | H3 histone, family 3A [Source:MGI Symbol;Acc:MGI:1097686] | 568,6865535 | -0,6724394 | **-1,59376549** | 9,15143441 | 13,9502291 | 0,000187715 | 0,001188716 | protein_coding |
| ENSMUSG00000032422 | Snx14 | sorting nexin 14 [Source:MGI Symbol;Acc:MGI:2155664] | 53,14798078 | -0,6737219 | **-1,595183** | 5,73135022 | 24,6592144 | 6,84168E-07 | 7,64696E-06 | protein_coding |
| ENSMUSG00000069874 | Irgm2 | immunity-related GTPase family M member 2 [Source:MGI Symbol;Acc:MGI:1926262] | 109,5254426 | -0,6738843 | **-1,59536252** | 6,77422285 | 22,4913028 | 2,11097E-06 | 2,13369E-05 | protein_coding |
| ENSMUSG00000051650 | B3gnt2 | UDP-GlcNAc:betaGal beta-1,3-N-acetylglucosaminyltransferase 2 [Source:MGI Symbol;Acc:MGI:1889505] | 139,4998298 | -0,6746964 | **-1,59626078** | 7,12252801 | 13,1090492 | 0,000293873 | 0,001755726 | protein_coding |
| ENSMUSG00000070738 | Dgkd | diacylglycerol kinase, delta [Source:MGI Symbol;Acc:MGI:2138334] | 146,1801304 | -0,6748744 | **-1,59645782** | 7,1902009 | 25,9004324 | 3,59489E-07 | 4,20249E-06 | protein_coding |
| ENSMUSG00000047921 | Trappc9 | trafficking protein particle complex 9 [Source:MGI Symbol;Acc:MGI:1923760] | 63,09144682 | -0,6752219 | **-1,59684231** | 5,9766169 | 23,6138751 | 1,17736E-06 | 1,25799E-05 | protein_coding |
| ENSMUSG00000039145 | Camk1d | calcium/calmodulin-dependent protein kinase ID [Source:MGI Symbol;Acc:MGI:2442190] | 207,9420389 | -0,6771528 | **-1,59898095** | 7,69933997 | 16,9166964 | 3,90565E-05 | 0,000293914 | protein_coding |
| ENSMUSG00000001323 | Srr | serine racemase [Source:MGI Symbol;Acc:MGI:1351636] | 13,96140743 | -0,6776503 | **-1,59953248** | 3,79202404 | 12,1198956 | 0,000498867 | 0,002800742 | protein_coding |
| ENSMUSG00000036634 | Mag | myelin-associated glycoprotein [Source:MGI Symbol;Acc:MGI:96912] | 8,967340255 | -0,6787932 | **-1,60080016** | 3,17836173 | 8,98911467 | 0,002715925 | 0,0121399 | protein_coding |
| ENSMUSG00000016529 | Il10 | interleukin 10 [Source:MGI Symbol;Acc:MGI:96537] | 36,59101276 | -0,6796735 | **-1,60177723** | 5,20198473 | 17,5451396 | 2,80567E-05 | 0,00021909 | protein_coding |
| ENSMUSG00000030759 | Far1 | fatty acyl CoA reductase 1 [Source:MGI Symbol;Acc:MGI:1914670] | 81,72528572 | -0,6797257 | **-1,60183515** | 6,34880649 | 25,8176058 | 3,7525E-07 | 4,37672E-06 | protein_coding |
| ENSMUSG00000046982 | Tshz1 | teashirt zinc finger family member 1 [Source:MGI Symbol;Acc:MGI:1346031] | 14,43464158 | -0,6797427 | **-1,60185403** | 3,85065506 | 12,8764471 | 0,000332743 | 0,001958889 | protein_coding |
| ENSMUSG00000040652 | Oaz2 | ornithine decarboxylase antizyme 2 [Source:MGI Symbol;Acc:MGI:109492] | 38,43902547 | -0,6798376 | **-1,60195941** | 5,25704029 | 20,6070025 | 5,63895E-06 | 5,20194E-05 | protein_coding |
| ENSMUSG00000045969 | Ing1 | inhibitor of growth family, member 1 [Source:MGI Symbol;Acc:MGI:1349481] | 23,81169449 | -0,6805908 | **-1,60279601** | 4,57245734 | 13,8597165 | 0,000196976 | 0,001235258 | protein_coding |
| ENSMUSG00000033949 | Trim36 | tripartite motif-containing 36 [Source:MGI Symbol;Acc:MGI:106264] | 12,49468439 | -0,6809002 | **-1,60313978** | 3,63574435 | 11,0619405 | 0,000881179 | 0,004603666 | protein_coding |
| ENSMUSG00000055093 | Gm8430 | predicted pseudogene 8430 [Source:MGI Symbol;Acc:MGI:3645406] | 251,4209375 | -0,681527 | **-1,60383641** | 7,97224768 | 20,6354647 | 5,55574E-06 | 5,14245E-05 | pseudogene |
| ENSMUSG00000058672 | Tubb2a | tubulin, beta 2A class IIA [Source:MGI Symbol;Acc:MGI:107861] | 221,2477655 | -0,6817982 | **-1,60413789** | 7,78965927 | 22,50512 | 2,09584E-06 | 2,12426E-05 | protein_coding |
| ENSMUSG00000032175 | Tyk2 | tyrosine kinase 2 [Source:MGI Symbol;Acc:MGI:1929470] | 116,6717258 | -0,68233 | **-1,6047293** | 6,8638927 | 23,6090178 | 1,18033E-06 | 1,25906E-05 | protein_coding |
| ENSMUSG00000048249 | A930001N09Rik | RIKEN cDNA A930001N09 gene [Source:MGI Symbol;Acc:MGI:1924378] | 43,81705829 | -0,6833319 | **-1,60584417** | 5,44404961 | 23,8923752 | 1,01874E-06 | 1,10931E-05 | protein_coding |
| ENSMUSG00000032402 | Smad3 | SMAD family member 3 [Source:MGI Symbol;Acc:MGI:1201674] | 46,53046902 | -0,685072 | **-1,6077822** | 5,53803704 | 22,4117812 | 2,2002E-06 | 2,21573E-05 | protein_coding |
| ENSMUSG00000083282 | Ctsf | cathepsin F [Source:MGI Symbol;Acc:MGI:1861434] | 10,71667899 | -0,6851766 | **-1,6078988** | 3,40586308 | 11,1059614 | 0,000860507 | 0,00450853 | protein_coding |
| ENSMUSG00000025453 | Nnt | nicotinamide nucleotide transhydrogenase [Source:MGI Symbol;Acc:MGI:109279] | 65,71267905 | -0,6863921 | **-1,60925405** | 6,03571547 | 25,7332142 | 3,92022E-07 | 4,56247E-06 | protein_coding |
| ENSMUSG00000032040 | Dcps | decapping enzyme, scavenger [Source:MGI Symbol;Acc:MGI:1916555] | 18,33940423 | -0,6865862 | **-1,60947056** | 4,19523068 | 16,4172636 | 5,08203E-05 | 0,000374237 | protein_coding |
| ENSMUSG00000044072 | Eml6 | echinoderm microtubule associated protein like 6 [Source:MGI Symbol;Acc:MGI:2442895] | 15,63548381 | -0,6882357 | **-1,61131184** | 3,95521717 | 12,9609218 | 0,000318061 | 0,001880803 | protein_coding |
| ENSMUSG00000031827 | Cotl1 | coactosin-like 1 (Dictyostelium) [Source:MGI Symbol;Acc:MGI:1919292] | 927,4319595 | -0,6894716 | **-1,61269276** | 9,85680526 | 13,1307789 | 0,000290484 | 0,001739263 | protein_coding |
| ENSMUSG00000024952 | Rps6ka4 | ribosomal protein S6 kinase, polypeptide 4 [Source:MGI Symbol;Acc:MGI:1930076] | 93,12800717 | -0,6897129 | **-1,61296251** | 6,53686324 | 19,9687903 | 7,87166E-06 | 6,99724E-05 | protein_coding |
| ENSMUSG00000091337 | Eid1 | EP300 interacting inhibitor of differentiation 1 [Source:MGI Symbol;Acc:MGI:1889651] | 87,69071809 | -0,689866 | **-1,61313364** | 6,45298151 | 23,9891127 | 9,6882E-07 | 1,06232E-05 | protein_coding |
| ENSMUSG00000051674 | Dcun1d4 | DCN1, defective in cullin neddylation 1, domain containing 4 (S. cerevisiae) [Source:MGI Symbol;Acc:MGI:2140972] | 13,18639047 | -0,6900078 | **-1,61329222** | 3,71465096 | 12,6205946 | 0,00038152 | 0,002217862 | protein_coding |
| ENSMUSG00000041453 | Rpl21 | ribosomal protein L21 [Source:MGI Symbol;Acc:MGI:1278340] | 76,70917757 | -0,6900536 | **-1,61334341** | 6,25789913 | 26,0350078 | 3,35282E-07 | 3,95252E-06 | protein_coding |
| ENSMUSG00000087267 | 4933427J07Rik | RIKEN cDNA 4933427J07 gene [Source:MGI Symbol;Acc:MGI:1918470] | 13,13315963 | -0,6901178 | **-1,6134153** | 3,69785987 | 10,528532 | 0,001175455 | 0,005889069 | antisense |
| ENSMUSG00000027247 | Arhgap1 | Rho GTPase activating protein 1 [Source:MGI Symbol;Acc:MGI:2445003] | 150,4144105 | -0,690128 | **-1,61342665** | 7,23165201 | 23,8447263 | 1,04428E-06 | 1,13023E-05 | protein_coding |
| ENSMUSG00000012848 | Rps5 | ribosomal protein S5 [Source:MGI Symbol;Acc:MGI:1097682] | 520,5931323 | -0,6901686 | **-1,61347201** | 9,02340752 | 15,5399152 | 8,07814E-05 | 0,000564596 | protein_coding |
| ENSMUSG00000015363 | Trabd | TraB domain containing [Source:MGI Symbol;Acc:MGI:1915226] | 73,68661767 | -0,6902322 | **-1,61354316** | 6,19975257 | 19,6720936 | 9,19342E-06 | 8,0613E-05 | protein_coding |
| ENSMUSG00000026411 | Tmem9 | transmembrane protein 9 [Source:MGI Symbol;Acc:MGI:1913491] | 31,13145522 | -0,6911508 | **-1,61457094** | 4,94874091 | 21,5720704 | 3,40778E-06 | 3,30754E-05 | protein_coding |
| ENSMUSG00000036879 | Phkb | phosphorylase kinase beta [Source:MGI Symbol;Acc:MGI:97578] | 41,51655703 | -0,6913994 | **-1,6148492** | 5,36614763 | 21,4132834 | 3,70198E-06 | 3,56786E-05 | protein_coding |
| ENSMUSG00000096199 | Ptrhd1 | peptidyl-tRNA hydrolase domain containing 1 [Source:MGI Symbol;Acc:MGI:1916959] | 13,96508797 | -0,6933273 | **-1,61700853** | 3,7831961 | 11,6190785 | 0,000652787 | 0,003543565 | protein_coding |
| ENSMUSG00000015355 | Cd48 | CD48 antigen [Source:MGI Symbol;Acc:MGI:88339] | 136,7660402 | -0,6936996 | **-1,61742582** | 7,09528234 | 25,2553062 | 5,02212E-07 | 5,75371E-06 | protein_coding |
| ENSMUSG00000032565 | Nudt16 | nudix (nucleoside diphosphate linked moiety X)-type motif 16 [Source:MGI Symbol;Acc:MGI:1922936] | 12,60632072 | -0,6938486 | **-1,61759296** | 3,63384905 | 11,6897997 | 0,000628437 | 0,003424917 | protein_coding |
| ENSMUSG00000087235 | Gm4750 | predicted gene 4750 [Source:MGI Symbol;Acc:MGI:3647391] | 7,712317382 | -0,6944535 | **-1,61827129** | 2,95540191 | 8,20079215 | 0,00418721 | 0,017455262 | pseudogene |
| ENSMUSG00000087569 | Gm8464 | predicted pseudogene 8464 [Source:MGI Symbol;Acc:MGI:3645517] | 7,712317382 | -0,6944544 | **-1,61827234** | 2,95540158 | 8,20050863 | 0,004187865 | 0,017455262 | pseudogene |
| ENSMUSG00000097431 | | | 12,83558583 | -0,6945405 | **-1,61836894** | 3,66573409 | 12,8757329 | 0,00033287 | 0,001958889 | lincRNA |
| ENSMUSG00000022498 | Txndc11 | thioredoxin domain containing 11 [Source:MGI Symbol;Acc:MGI:1923620] | 44,01101682 | -0,6947789 | **-1,61863634** | 5,45239074 | 19,9755454 | 7,84389E-06 | 6,98386E-05 | protein_coding |
| ENSMUSG00000030685 | Kctd13 | potassium channel tetramerisation domain containing 13 [Source:MGI Symbol;Acc:MGI:1923739] | 17,97473655 | -0,6964145 | **-1,62047248** | 4,15188941 | 14,9875523 | 0,000108223 | 0,000729922 | protein_coding |
| ENSMUSG00000020823 | Sec14l1 | SEC14-like 1 (S. cerevisiae) [Source:MGI Symbol;Acc:MGI:1921386] | 373,9093617 | -0,6974631 | **-1,62165067** | 8,54597868 | 17,5534734 | 2,7934E-05 | 0,000218598 | protein_coding |
| ENSMUSG00000014905 | Dnajb9 | DnaJ (Hsp40) homolog, subfamily B, member 9 [Source:MGI Symbol;Acc:MGI:1351618] | 46,70242152 | -0,6979925 | **-1,62224585** | 5,54431954 | 20,0636581 | 7,49066E-06 | 6,68562E-05 | protein_coding |
| ENSMUSG00000042129 | Rassf4 | Ras association (RalGDS/AF-6) domain family member 4 [Source:MGI Symbol;Acc:MGI:2386853] | 1568,401556 | -0,6981673 | **-1,62244241** | 10,6150594 | 10,111952 | 0,001473109 | 0,007186964 | protein_coding |
| ENSMUSG00000029624 | Ptcd1 | pentatricopeptide repeat domain 1 [Source:MGI Symbol;Acc:MGI:1919049] | 32,36641662 | -0,6982318 | **-1,62251494** | 5,00852035 | 18,3296904 | 1,85789E-05 | 0,000151791 | protein_coding |
| ENSMUSG00000022346 | Myc | myelocytomatosis oncogene [Source:MGI Symbol;Acc:MGI:97250] | 46,93652431 | -0,6985797 | **-1,62290634** | 5,55034744 | 23,9743658 | 9,76269E-07 | 1,06942E-05 | protein_coding |
| ENSMUSG00000039197 | Adk | adenosine kinase [Source:MGI Symbol;Acc:MGI:87930] | 118,4627207 | -0,6998337 | **-1,62431755** | 6,8880448 | 19,204259 | 1,17451E-05 | 0,000100189 | protein_coding |
| ENSMUSG00000030876 | Mettl9 | methyltransferase like 9 [Source:MGI Symbol;Acc:MGI:1914862] | 30,82860821 | -0,7004254 | **-1,6249839** | 4,9394994 | 18,456721 | 1,73807E-05 | 0,000143688 | protein_coding |
| ENSMUSG00000079168 | Cd209g | CD209g antigen [Source:MGI Symbol;Acc:MGI:1917442] | 187,8543489 | -0,7009141 | **-1,62553446** | 7,55346055 | 20,7206455 | 5,314E-06 | 4,93115E-05 | protein_coding |
| ENSMUSG00000037458 | Azin1 | antizyme inhibitor 1 [Source:MGI Symbol;Acc:MGI:1859169] | 499,9968255 | -0,7015234 | **-1,6262211** | 8,9652376 | 15,5783668 | 7,91551E-05 | 0,000555484 | protein_coding |
| ENSMUSG00000003644 | Rps6ka1 | ribosomal protein S6 kinase polypeptide 1 [Source:MGI Symbol;Acc:MGI:104558] | 281,0671156 | -0,7021456 | **-1,62692262** | 8,13389036 | 19,5065046 | 1,00258E-05 | 8,69401E-05 | protein_coding |
| ENSMUSG00000030839 | Sergef | secretion regulating guanine nucleotide exchange factor [Source:MGI Symbol;Acc:MGI:1351630] | 8,88943442 | -0,7030415 | **-1,62793323** | 3,14693727 | 10,1973578 | 0,00140642 | 0,006886069 | protein_coding |

| | | | | | | | | | | |
|---|---|---|---|---|---|---|---|---|---|---|
| ENSMUSG00000040563 | BC018242 | cDNA sequence BC018242 [Source:MGI Symbol;Acc:MGI:2384575] | 18,88299469 | -0,703166 | **-1,62807372** | 4,22205747 | 17,8895599 | 2,34102E-05 | 0,00018706 | protein_coding |
| ENSMUSG00000050621 | Gm9846 | predicted gene 9846 [Source:MGI Symbol;Acc:MGI:3704345] | 229,8389283 | -0,7036556 | **-1,62862632** | 7,8427767 | 21,4007076 | 3,72634E-06 | 3,58819E-05 | protein_coding |
| ENSMUSG00000004500 | Zfp324 | zinc finger protein 324 [Source:MGI Symbol;Acc:MGI:2444641] | 9,348784407 | -0,7039222 | **-1,62892732** | 3,21187717 | 8,3513154 | 0,003854082 | 0,016292728 | protein_coding |
| ENSMUSG00000047617 | BC029214 | cDNA sequence BC029214 [Source:MGI Symbol;Acc:MGI:2442831] | 5,162599073 | -0,7053428 | **-1,63053207** | 2,34864251 | 6,14060341 | 0,013211222 | 0,045445116 | protein_coding |
| ENSMUSG00000061983 | Rps12 | ribosomal protein S12 [Source:MGI Symbol;Acc:MGI:98105] | 496,178117 | -0,7059017 | **-1,63116385** | 8,95415371 | 16,2145679 | 5,65575E-05 | 0,000410977 | protein_coding |
| ENSMUSG00000030042 | Pole4 | polymerase (DNA-directed), epsilon 4 (p12 subunit) [Source:MGI Symbol;Acc:MGI:1914229] | 79,71864753 | -0,7062651 | **-1,63157475** | 6,31462325 | 28,9257807 | 7,52053E-08 | 1,02136E-06 | protein_coding |
| ENSMUSG00000001248 | Gramd1a | GRAM domain containing 1A [Source:MGI Symbol;Acc:MGI:105490] | 296,851193 | -0,7063562 | **-1,63167781** | 8,2134481 | 18,0058136 | 2,20231E-05 | 0,000177396 | protein_coding |
| ENSMUSG00000091747 | D17H6S56E-5 | DNA segment, Chr 17, human D6S56E 5 [Source:MGI Symbol;Acc:MGI:1306799] | 46,77327383 | -0,7065302 | **-1,63187463** | 5,56000551 | 26,111747 | 3,22218E-07 | 3,83048E-06 | protein_coding |
| ENSMUSG00000046330 | Rpl37a | ribosomal protein L37a [Source:MGI Symbol;Acc:MGI:98068] | 321,9371152 | -0,7073063 | **-1,63275273** | 8,32922658 | 20,7639943 | 5,19506E-06 | 4,84945E-05 | protein_coding |
| ENSMUSG00000033111 | 3830406C13Rik | RIKEN cDNA 3830406C13 gene [Source:MGI Symbol;Acc:MGI:1917937] | 22,8371441 | -0,7082795 | **-1,63385454** | 4,51137502 | 19,2959762 | 1,11942E-05 | 9,59236E-05 | protein_coding |
| ENSMUSG00000003429 | Rps11 | ribosomal protein S11 [Source:MGI Symbol;Acc:MGI:1351329] | 486,2684177 | -0,7086554 | **-1,63428027** | 8,92513003 | 16,3814176 | 5,17905E-05 | 0,000379601 | protein_coding |
| ENSMUSG00000027452 | Acss1 | acyl-CoA synthetase short-chain family member 1 [Source:MGI Symbol;Acc:MGI:1915988] | 28,30467773 | -0,708882 | **-1,63453691** | 4,80945241 | 21,2559499 | 4,01861E-06 | 3,84603E-05 | protein_coding |
| ENSMUSG00000024985 | Tcf7l2 | transcription factor 7 like 2, T cell specific, HMG box [Source:MGI Symbol;Acc:MGI:1202879] | 16,16457218 | -0,7090773 | **-1,6347583** | 4,01723419 | 17,5489641 | 2,80003E-05 | 0,000218805 | protein_coding |
| ENSMUSG00000026864 | Hspa5 | heat shock protein 5 [Source:MGI Symbol;Acc:MGI:95835] | 780,2816957 | -0,7106387 | **-1,63652851** | 9,60782136 | 13,6485088 | 0,000220416 | 0,001363578 | protein_coding |
| ENSMUSG00000002395 | Use1 | unconventional SNARE in the ER 1 homolog (S. cerevisiae) [Source:MGI Symbol;Acc:MGI:1914273] | 98,3733278 | -0,7111265 | **-1,63708188** | 6,61514665 | 21,7374344 | 3,12631E-06 | 3,06139E-05 | protein_coding |
| ENSMUSG00000000708 | Kat2b | K(lysine) acetyltransferase 2B [Source:MGI Symbol;Acc:MGI:1343094] | 60,86086243 | -0,7112685 | **-1,63724308** | 5,92567612 | 23,7628961 | 1,08963E-06 | 1,1714E-05 | protein_coding |
| ENSMUSG00000006850 | Tmco6 | transmembrane and coiled-coil domains 6 [Source:MGI Symbol;Acc:MGI:1919233] | 9,306702334 | -0,711735 | **-1,63777252** | 3,20236814 | 8,77207083 | 0,00305878 | 0,013448104 | protein_coding |
| ENSMUSG00000074505 | Fat3 | FAT tumor suppressor homolog 3 (Drosophila) [Source:MGI Symbol;Acc:MGI:2444314] | 6,434799909 | -0,713705 | **-1,64001044** | 2,65921177 | 7,42984529 | 0,006415086 | 0,025011549 | protein_coding |
| ENSMUSG00000039646 | Vasn | vasorin [Source:MGI Symbol;Acc:MGI:2177651] | 18,58368962 | -0,7138665 | **-1,64019399** | 4,188579 | 18,397967 | 1,79249E-05 | 0,000147411 | protein_coding |
| ENSMUSG00000029580 | Actb | actin, beta [Source:MGI Symbol;Acc:MGI:87904] | 7517,721048 | -0,7146149 | **-1,64104508** | 12,8760694 | 6,83833014 | 0,00892222 | 0,03305072 | protein_coding |
| ENSMUSG00000063001 | Rps23-ps | ribosomal protein S23, pseudogene [Source:MGI Symbol;Acc:MGI:3705429] | 496,4561538 | -0,7146581 | **-1,6410943** | 8,95494418 | 16,7002428 | 4,37754E-05 | 0,000325193 | pseudogene |
| ENSMUSG00000030220 | Arhgdib | Rho, GDP dissociation inhibitor (GDI) beta [Source:MGI Symbol;Acc:MGI:101940] | 990,1359016 | -0,7154735 | **-1,64202205** | 9,9514812 | 12,9704222 | 0,000316451 | 0,00187229 | protein_coding |
| ENSMUSG00000022425 | Enpp2 | ectonucleotide pyrophosphatase/phosphodiesterase 2 [Source:MGI Symbol;Acc:MGI:1321390] | 48,82092697 | -0,7155757 | **-1,64213838** | 5,61222248 | 16,0231079 | 6,25741E-05 | 0,000451116 | protein_coding |
| ENSMUSG00000055322 | Tns1 | tensin 1 [Source:MGI Symbol;Acc:MGI:104552] | 78,93518095 | -0,7156765 | **-1,64225307** | 6,29409607 | 29,3495975 | 6,04291E-08 | 8,33042E-07 | protein_coding |
| ENSMUSG00000025104 | Hdgfrp3 | hepatoma-derived growth factor, related protein 3 [Source:MGI Symbol;Acc:MGI:1352760] | 9,33445032 | -0,7160798 | **-1,64271223** | 3,21484189 | 10,5204113 | 0,001180632 | 0,005912308 | protein_coding |
| ENSMUSG00000020718 | Polg2 | polymerase (DNA directed), gamma 2, accessory subunit [Source:MGI Symbol;Acc:MGI:1354947] | 9,396114198 | -0,7170901 | **-1,64386303** | 3,2164509 | 10,6117292 | 0,001123726 | 0,005666073 | protein_coding |
| ENSMUSG00000027808 | Serp1 | stress-associated endoplasmic reticulum protein 1 [Source:MGI Symbol;Acc:MGI:92638] | 2244,396997 | -0,7171005 | **-1,64387492** | 11,1320978 | 9,68507516 | 0,001857708 | 0,008809079 | protein_coding |
| ENSMUSG00000027947 | Il6ra | interleukin 6 receptor, alpha [Source:MGI Symbol;Acc:MGI:105304] | 49,33113213 | -0,7173072 | **-1,64411043** | 5,61857343 | 20,8575012 | 4,94751E-06 | 4,63808E-05 | protein_coding |
| ENSMUSG00000024538 | Ppic | peptidylprolyl isomerase C [Source:MGI Symbol;Acc:MGI:97751] | 29,55733989 | -0,717612 | **-1,6444578** | 4,88376628 | 24,0358582 | 9,45582E-07 | 1,04099E-05 | protein_coding |
| ENSMUSG00000021892 | Sh3bp5 | SH3-domain binding protein 5 (BTK-associated) [Source:MGI Symbol;Acc:MGI:1344391] | 346,2336727 | -0,7177015 | **-1,6445598** | 8,43523689 | 20,0982245 | 7,35649E-06 | 6,57655E-05 | protein_coding |
| ENSMUSG00000004661 | Arid3b | AT rich interactive domain 3B (BRIGHT-like) [Source:MGI Symbol;Acc:MGI:1930768] | 53,30157701 | -0,7186893 | **-1,64568628** | 5,73088834 | 25,9866542 | 3,43786E-07 | 4,04408E-06 | protein_coding |
| ENSMUSG00000032841 | Prr5l | proline rich 5 like [Source:MGI Symbol;Acc:MGI:1919696] | 19,37074871 | -0,7196021 | **-1,64672781** | 4,26080146 | 16,1961791 | 5,71092E-05 | 0,000414438 | protein_coding |
| ENSMUSG00000069682 | Gm10275 | predicted pseudogene 10275 [Source:MGI Symbol;Acc:MGI:3704354] | 572,6138054 | -0,7197648 | **-1,64691354** | 9,16088607 | 16,8277625 | 4,09301E-05 | 0,00030571 | protein_coding |
| ENSMUSG00000018669 | Cdk5rap3 | CDK5 regulatory subunit associated protein 3 [Source:MGI Symbol;Acc:MGI:1933126] | 27,86154433 | -0,7198963 | **-1,64706368** | 4,79214276 | 18,1007925 | 2,09514E-05 | 0,00017001 | protein_coding |
| ENSMUSG00000090136 | Gm10177 | predicted gene 10177 [Source:MGI Symbol;Acc:MGI:3704330] | 67,89840143 | -0,7199431 | **-1,64711711** | 6,08112805 | 21,8181877 | 2,99745E-06 | 2,94308E-05 | pseudogene |
| ENSMUSG00000002043 | Trappc6a | trafficking protein particle complex 6A [Source:MGI Symbol;Acc:MGI:1914341] | 16,93943108 | -0,7207906 | **-1,64808489** | 4,0640418 | 16,8112277 | 4,12883E-05 | 0,000308176 | protein_coding |
| ENSMUSG00000039031 | Arhgap18 | Rho GTPase activating protein 18 [Source:MGI Symbol;Acc:MGI:1921160] | 58,81778502 | -0,7213323 | **-1,64870391** | 5,87116837 | 28,9160842 | 7,55827E-08 | 1,02395E-06 | protein_coding |
| ENSMUSG00000039001 | Rps21 | ribosomal protein S21 [Source:MGI Symbol;Acc:MGI:1913731] | 253,4287629 | -0,7213353 | **-1,64870728** | 7,98313765 | 23,1134492 | 1,52719E-06 | 1,59347E-05 | protein_coding |
| ENSMUSG00000030766 | Arhgap17 | Rho GTPase activating protein 17 [Source:MGI Symbol;Acc:MGI:1917747] | 175,442988 | -0,7217488 | **-1,64917989** | 7,45265766 | 27,0566089 | 1,97584E-07 | 2,42282E-06 | protein_coding |
| ENSMUSG00000078453 | 3110003A17Rik | RIKEN cDNA 3110003A17 gene [Source:MGI Symbol;Acc:MGI:1920362] | 92,12445305 | -0,7227795 | **-1,65035854** | 6,52680645 | 21,6709266 | 3,2366E-06 | 3,15813E-05 | protein_coding |
| ENSMUSG00000041912 | Tdrkh | tudor and KH domain containing protein [Source:MGI Symbol;Acc:MGI:1919884] | 15,45038817 | -0,7232292 | **-1,65087306** | 3,94941833 | 12,800076 | 0,000346605 | 0,002031014 | protein_coding |
| ENSMUSG00000026670 | Uap1 | UDP-N-acetylglucosamine pyrophosphorylase 1 [Source:MGI Symbol;Acc:MGI:1334459] | 20,24321102 | -0,7246763 | **-1,65252984** | 4,32744972 | 14,9981811 | 0,000107615 | 0,000726714 | protein_coding |
| ENSMUSG00000026311 | Asb1 | ankyrin repeat and SOCS box-containing 1 [Source:MGI Symbol;Acc:MGI:1929735] | 21,88897401 | -0,7255182 | **-1,65349447** | 4,44531541 | 16,3915972 | 5,15131E-05 | 0,000378072 | protein_coding |
| ENSMUSG00000028495 | Rps6 | ribosomal protein S6 [Source:MGI Symbol;Acc:MGI:98159] | 63,01023327 | -0,7258703 | **-1,65389806** | 5,9721465 | 12,0734414 | 0,000511452 | 0,00286262 | protein_coding |
| ENSMUSG00000024677 | Ms4a6b | membrane-spanning 4-domains, subfamily A, member 6B [Source:MGI Symbol;Acc:MGI:1917024] | 110,3017162 | -0,7261491 | **-1,65421774** | 6,78376052 | 19,3084208 | 1,11215E-05 | 9,54623E-05 | protein_coding |
| ENSMUSG00000049775 | Tmsb4x | thymosin, beta 4, X chromosome [Source:MGI Symbol;Acc:MGI:99510] | 9386,9507 | -0,726206 | **-1,65428297** | 13,196436 | 6,37256746 | 0,01158978 | 0,041049938 | protein_coding |
| ENSMUSG00000002983 | Relb | avian reticuloendotheliosis viral (v-rel) oncogene related B [Source:MGI Symbol;Acc:MGI:103289] | 63,60013344 | -0,726355 | **-1,6544538** | 5,98819697 | 21,9722615 | 2,7662E-06 | 2,73557E-05 | protein_coding |
| ENSMUSG00000004947 | Dtx2 | deltex 2 homolog (Drosophila) [Source:MGI Symbol;Acc:MGI:1921448] | 73,94876274 | -0,7278895 | **-1,65621445** | 6,2057295 | 21,5119276 | 3,51635E-06 | 3,40689E-05 | protein_coding |
| ENSMUSG00000033713 | Foxn3 | forkhead box N3 [Source:MGI Symbol;Acc:MGI:1918625] | 20,54621374 | -0,7286445 | **-1,65708148** | 4,34443993 | 17,1998663 | 3,3646E-05 | 0,000257071 | protein_coding |
| ENSMUSG00000046463 | 5930403N24Rik | RIKEN cDNA 5930403N24 gene [Source:MGI Symbol;Acc:MGI:2444171] | 5,574586079 | -0,7293586 | **-1,65790181** | 2,51515416 | 6,79477734 | 0,009142493 | 0,033718887 | protein_coding |
| ENSMUSG00000013698 | Pea15a | phosphoprotein enriched in astrocytes 15A [Source:MGI Symbol;Acc:MGI:104799] | 512,8360872 | -0,7305702 | **-1,65929474** | 9,00209099 | 17,0225588 | 3,69383E-05 | 0,000279347 | protein_coding |
| ENSMUSG00000035629 | 1700021K19Rik | RIKEN cDNA 1700021K19 gene [Source:MGI Symbol;Acc:MGI:1915160] | 90,93310997 | -0,7308487 | **-1,65961513** | 6,50124874 | 24,2019543 | 8,67442E-07 | 9,59691E-06 | protein_coding |
| ENSMUSG00000009927 | Rps25 | ribosomal protein S25 [Source:MGI Symbol;Acc:MGI:1922867] | 432,2667631 | -0,7321574 | **-1,66112131** | 8,75514461 | 19,5585178 | 9,75648E-06 | 8,48057E-05 | protein_coding |
| ENSMUSG00000046027 | Stard5 | StAR-related lipid transfer (START) domain containing 5 [Source:MGI Symbol;Acc:MGI:2156765] | 119,7897333 | -0,7323294 | **-1,66131928** | 6,90650663 | 20,6718713 | 5,45109E-06 | 5,04985E-05 | protein_coding |
| ENSMUSG00000079435 | Rpl36a | ribosomal protein L36A [Source:MGI Symbol;Acc:MGI:1201789] | 198,9995461 | -0,7346988 | **-1,66405002** | 7,63489387 | 25,9334278 | 3,53396E-07 | 4,14383E-06 | protein_coding |
| ENSMUSG00000078921 | Tgtp2 | T cell specific GTPase 2 [Source:MGI Symbol;Acc:MGI:3710083] | 16,44970034 | -0,7354941 | **-1,66496757** | 4,04204753 | 14,9444721 | 0,000110722 | 0,000745406 | protein_coding |
| ENSMUSG00000003617 | Cp | ceruloplasmin [Source:MGI Symbol;Acc:MGI:88476] | 256,2345339 | -0,7362618 | **-1,66585379** | 8,00130027 | 13,3704692 | 0,000255617 | 0,001554216 | protein_coding |
| ENSMUSG00000023393 | Slc17a9 | solute carrier family 17, member 9 [Source:MGI Symbol;Acc:MGI:1919107] | 10,37985953 | -0,7372285 | **-1,66697036** | 3,36082416 | 10,9708729 | 0,000925552 | 0,004798978 | protein_coding |
| ENSMUSG00000052056 | Zfp217 | zinc finger protein 217 [Source:MGI Symbol;Acc:MGI:2685408] | 158,8825946 | -0,7382163 | **-1,66811213** | 7,31033836 | 22,118127 | 2,56377E-06 | 2,55842E-05 | protein_coding |
| ENSMUSG00000031310 | Zmym3 | zinc finger, MYM-type 3 [Source:MGI Symbol;Acc:MGI:1927231] | 50,32197668 | -0,7387712 | **-1,66875385** | 5,64261315 | 26,1101204 | 3,22489E-07 | 3,83048E-06 | protein_coding |
| ENSMUSG00000038718 | Pbx3 | pre B cell leukemia homeobox 3 [Source:MGI Symbol;Acc:MGI:97496] | 17,86569502 | -0,7395698 | **-1,66967785** | 4,15330828 | 18,385385 | 1,80437E-05 | 0,000148166 | protein_coding |
| ENSMUSG00000044150 | A830080D01Rik | RIKEN cDNA A830080D01 gene [Source:MGI Symbol;Acc:MGI:2685992] | 23,64913702 | -0,7402739 | **-1,67049291** | 4,55838289 | 22,4657227 | 2,13927E-06 | 2,15833E-05 | protein_coding |
| ENSMUSG00000086717 | Gm15655 | predicted gene 15655 [Source:MGI Symbol;Acc:MGI:3805551] | 10,18354514 | -0,7406984 | **-1,67098455** | 3,37792286 | 7,94740679 | 0,004815633 | 0,019581555 | antisense |
| ENSMUSG00000072692 | Gm13826 | predicted gene 13826 [Source:MGI Symbol;Acc:MGI:3651519] | 128,4737196 | -0,7412184 | **-1,67158695** | 7,00098955 | 27,4010577 | 1,65341E-07 | 2,0573E-06 | pseudogene |
| ENSMUSG00000005951 | Shpk | sedoheptulokinase [Source:MGI Symbol;Acc:MGI:1921887] | 4,876131245 | -0,7416943 | **-1,67213842** | 2,25497431 | 6,0707142 | 0,013744138 | 0,046911102 | protein_coding |
| ENSMUSG00000071311 | Gpr31b | G protein-coupled receptor 31, D17Leh66b region [Source:MGI Symbol;Acc:MGI:1354372] | 21,65998848 | -0,7419728 | **-1,67246126** | 4,44567672 | 12,8224867 | 0,000342478 | 0,002010047 | protein_coding |
| ENSMUSG00000028788 | Ptp4a2 | protein tyrosine phosphatase 4a2 [Source:MGI Symbol;Acc:MGI:1277117] | 608,9242495 | -0,7423651 | **-1,67291608** | 9,249856 | 17,4310053 | 2,97926E-05 | 0,000231167 | protein_coding |
| ENSMUSG00000033444 | Specc1l | sperm antigen with calponin homology and coiled-coil domains 1-like [Source:MGI Symbol;Acc:MGI:1921642] | 170,8893867 | -0,7428528 | **-1,67348178** | 7,41536243 | 25,9701613 | 3,46735E-07 | 4,07442E-06 | protein_coding |
| ENSMUSG00000067038 | Rps12-ps3 | ribosomal protein S12, pseudogene 3 [Source:MGI Symbol;Acc:MGI:3704503] | 268,6398089 | -0,7433922 | **-1,6741075** | 8,06821077 | 20,2384285 | 6,83657E-06 | 6,18727E-05 | protein_coding |
| ENSMUSG00000089726 | Mir17hg | MIR17 host gene 1 (non-protein coding) [Source:MGI Symbol;Acc:MGI:1923207] | 5,557425295 | -0,7454184 | **-1,67646044** | 2,4705822 | 7,06935846 | 0,007841333 | 0,029666916 | lincRNA |
| ENSMUSG00000033014 | Trim33 | tripartite motif-containing 33 [Source:MGI Symbol;Acc:MGI:2137357] | 90,55273609 | -0,7469685 | **-1,67826266** | 6,49852211 | 30,2371105 | 3,82324E-08 | 5,50536E-07 | protein_coding |

| | | | | | | | | | | |
|---|---|---|---|---|---|---|---|---|---|---|
| ENSMUSG00000024975 | Pdcd4 | programmed cell death 4 [Source:MGI Symbol;Acc:MGI:107490] | 9,625353015 | -0,7484823 | **-1,68002448** | 3,24832542 | 9,1031122 | 0,002551749 | 0,01150906 | protein_coding |
| ENSMUSG00000024477 | Pggt1b | protein geranylgeranyltransferase type I, beta subunit [Source:MGI Symbol;Acc:MGI:1917514] | 54,50962941 | -0,749177 | **-1,6808337** | 5,76475947 | 27,6135247 | 1,48139E-07 | 1,86225E-06 | protein_coding |
| ENSMUSG00000027639 | Samhd1 | SAM domain and HD domain, 1 [Source:MGI Symbol;Acc:MGI:1927468] | 800,0463275 | -0,7494736 | **-1,68117927** | 9,64376278 | 15,0582688 | 0,000104243 | 0,00070742 | protein_coding |
| ENSMUSG00000024981 | Acsl5 | acyl-CoA synthetase long-chain family member 5 [Source:MGI Symbol;Acc:MGI:1919129] | 327,0985932 | -0,7512424 | **-1,68324181** | 8,35321336 | 17,9115965 | 2,31407E-05 | 0,000185446 | protein_coding |
| ENSMUSG00000031749 | St3gal2 | ST3 beta-galactoside alpha-2,3-sialyltransferase 2 [Source:MGI Symbol;Acc:MGI:99427] | 55,15600828 | -0,7520113 | **-1,68413907** | 5,7779794 | 22,3207655 | 2,30698E-06 | 2,3169E-05 | protein_coding |
| ENSMUSG00000085334 | Gm12940 | predicted gene 12940 [Source:MGI Symbol;Acc:MGI:3702626] | 6,487255396 | -0,7559833 | **-1,68878219** | 2,68471851 | 7,78138736 | 0,005278724 | 0,021221128 | processed_transcrip |
| ENSMUSG00000027219 | Slc28a2 | solute carrier family 28 (sodium-coupled nucleoside transporter), member 2 [Source:MGI Symbol;Acc:MGI:1913105] | 119,392611 | -0,7562451 | **-1,68908875** | 6,90072023 | 23,1082387 | 1,53133E-06 | 1,59628E-05 | protein_coding |
| ENSMUSG00000059493 | Nhs | Nance-Horan syndrome (human) [Source:MGI Symbol;Acc:MGI:2684894] | 45,36099223 | -0,7565728 | **-1,68947242** | 5,50215322 | 19,4017972 | 1,05907E-05 | 9,13347E-05 | protein_coding |
| ENSMUSG00000039936 | Pik3cd | phosphatidylinositol 3-kinase catalytic delta polypeptide [Source:MGI Symbol;Acc:MGI:1098211] | 249,7879799 | -0,7573125 | **-1,6903389** | 7,96387781 | 20,3960565 | 6,29594E-06 | 5,74713E-05 | protein_coding |
| ENSMUSG00000072235 | Tuba1a | tubulin, alpha 1A [Source:MGI Symbol;Acc:MGI:98869] | 92,44680752 | -0,7576767 | **-1,69076569** | 6,52714661 | 17,2814634 | 3,22316E-05 | 0,000247989 | protein_coding |
| ENSMUSG00000035711 | Dok3 | docking protein 3 [Source:MGI Symbol;Acc:MGI:1351490] | 127,6385165 | -0,7578257 | **-1,69094033** | 6,99150892 | 24,1597689 | 8,86653E-07 | 9,78078E-06 | protein_coding |
| ENSMUSG00000031728 | Zfp821 | zinc finger protein 821 [Source:MGI Symbol;Acc:MGI:1923121] | 27,43832056 | -0,7582487 | **-1,69143617** | 4,78210191 | 21,89759 | 2,87595E-06 | 2,83137E-05 | protein_coding |
| ENSMUSG00000067344 | Rps25-ps1 | ribosomal protein S25, pseudogene 1 [Source:MGI Symbol;Acc:MGI:3642902] | 19,81846232 | -0,7584461 | **-1,69166753** | 4,29749807 | 15,3915486 | 8,73782E-05 | 0,000606459 | pseudogene |
| ENSMUSG00000015702 | Anxa9 | annexin A9 [Source:MGI Symbol;Acc:MGI:1923711] | 8,549136451 | -0,7587396 | **-1,6920118** | 3,07124668 | 10,3646696 | 0,001284499 | 0,006359977 | protein_coding |
| ENSMUSG00000093930 | Hmgcs1 | 3-hydroxy-3-methylglutaryl-Coenzyme A synthase 1 [Source:MGI Symbol;Acc:MGI:107592] | 79,68002064 | -0,7598561 | **-1,69332172** | 6,31204052 | 12,3852898 | 0,000432729 | 0,002468535 | protein_coding |
| ENSMUSG00000001366 | Fbxo9 | f-box protein 9 [Source:MGI Symbol;Acc:MGI:1918788] | 18,39103776 | -0,7600659 | **-1,69356803** | 4,18756702 | 20,7661337 | 5,18925E-06 | 4,84816E-05 | protein_coding |
| ENSMUSG00000025362 | Rps26 | ribosomal protein S26 [Source:MGI Symbol;Acc:MGI:1351628] | 332,9977199 | -0,7610714 | **-1,69474871** | 8,3782033 | 21,9883728 | 2,74307E-06 | 2,7176E-05 | protein_coding |
| ENSMUSG00000030089 | Slc41a3 | solute carrier family 41, member 3 [Source:MGI Symbol;Acc:MGI:1918949] | 26,27791998 | -0,7616001 | **-1,69536995** | 4,69975822 | 21,5856084 | 3,38381E-06 | 3,293E-05 | protein_coding |
| ENSMUSG00000020122 | Egfr | epidermal growth factor receptor [Source:MGI Symbol;Acc:MGI:95294] | 8,604570654 | -0,7618008 | **-1,69560584** | 3,09624161 | 9,789652 | 0,001754967 | 0,008412661 | protein_coding |
| ENSMUSG00000022895 | Ets2 | E26 avian leukemia oncogene 2, 3' domain [Source:MGI Symbol;Acc:MGI:95456] | 326,0447119 | -0,7618886 | **-1,695709** | 8,34958609 | 20,8805944 | 4,88822E-06 | 4,59033E-05 | protein_coding |
| ENSMUSG00000022136 | Dnajc3 | DnaJ (Hsp40) homolog, subfamily C, member 3 [Source:MGI Symbol;Acc:MGI:107373] | 121,1223693 | -0,7620518 | **-1,69590077** | 6,91857234 | 28,2927479 | 1,04286E-07 | 1,36566E-06 | protein_coding |
| ENSMUSG00000058446 | Znrf2 | zinc and ring finger 2 [Source:MGI Symbol;Acc:MGI:1196246] | 244,8283104 | -0,763125 | **-1,69716283** | 7,93507812 | 24,7321402 | 6,58764E-07 | 7,37802E-06 | protein_coding |
| ENSMUSG00000033467 | Crlf2 | cytokine receptor-like factor 2 [Source:MGI Symbol;Acc:MGI:1889506] | 77,63027024 | -0,7634616 | **-1,69755881** | 6,26958514 | 31,4305678 | 2,06701E-08 | 3,11526E-07 | protein_coding |
| ENSMUSG00000042249 | Adrbk2 | adrenergic receptor kinase, beta 2 [Source:MGI Symbol;Acc:MGI:87941] | 75,28867822 | -0,764001 | **-1,69819368** | 6,23107601 | 24,9755035 | 5,80634E-07 | 6,55645E-06 | protein_coding |
| ENSMUSG00000000958 | Slc7a7 | solute carrier family 7 (cationic amino acid transporter, y+ system), member 7 [Source:MGI Symbol;Acc:MGI:1337120] | 58,62492161 | -0,7652614 | **-1,69967791** | 5,87278132 | 27,32199 | 1,72241E-07 | 2,14074E-06 | protein_coding |
| ENSMUSG00000031246 | Sh3bgrl | SH3-binding domain glutamic acid-rich protein like [Source:MGI Symbol;Acc:MGI:1930849] | 741,6573282 | -0,7660423 | **-1,70059817** | 9,53438104 | 18,3920442 | 1,79807E-05 | 0,000147759 | protein_coding |
| ENSMUSG00000022365 | Derl1 | Der1-like domain family, member 1 [Source:MGI Symbol;Acc:MGI:1915069] | 246,900918 | -0,7673664 | **-1,70215971** | 7,94675619 | 22,4891349 | 2,11336E-06 | 2,13414E-05 | protein_coding |
| ENSMUSG00000001467 | Cyp51 | cytochrome P450, family 51 [Source:MGI Symbol;Acc:MGI:106040] | 45,75230432 | -0,7680581 | **-1,70297596** | 5,51044199 | 7,35176326 | 0,006699702 | 0,025946841 | protein_coding |
| ENSMUSG00000045625 | Pigz | phosphatidylinositol glycan anchor biosynthesis, class Z [Source:MGI Symbol;Acc:MGI:2443822] | 12,61951567 | -0,7687094 | **-1,70374499** | 3,65491457 | 15,3931528 | 8,73041E-05 | 0,000606327 | protein_coding |
| ENSMUSG00000039115 | Itga9 | integrin alpha 9 [Source:MGI Symbol;Acc:MGI:104756] | 86,66028459 | -0,770087 | **-1,7053726** | 6,43813534 | 30,1968199 | 3,90349E-08 | 5,59891E-07 | protein_coding |
| ENSMUSG00000022463 | Srebf2 | sterol regulatory element binding factor 2 [Source:MGI Symbol;Acc:MGI:107585] | 147,9789252 | -0,7713936 | **-1,70691782** | 7,20637973 | 20,7411042 | 5,25752E-06 | 4,89529E-05 | protein_coding |
| ENSMUSG00000031328 | Flna | filamin, alpha [Source:MGI Symbol;Acc:MGI:95556] | 1988,719419 | -0,7716919 | **-1,70727079** | 10,9575789 | 12,5839249 | 0,000389079 | 0,002258219 | protein_coding |
| ENSMUSG00000030934 | Oat | ornithine aminotransferase [Source:MGI Symbol;Acc:MGI:97394] | 137,8261725 | -0,7728707 | **-1,70866633** | 7,10497048 | 31,3467698 | 2,15817E-08 | 3,24376E-07 | protein_coding |
| ENSMUSG00000020964 | Sel1l | sel-1 suppressor of lin-12-like (C. elegans) [Source:MGI Symbol;Acc:MGI:1329016] | 292,793671 | -0,773947 | **-1,70994155** | 8,19304197 | 23,2106325 | 1,45192E-06 | 1,51927E-05 | protein_coding |
| ENSMUSG00000030269 | Mtmr14 | myotubularin related protein 14 [Source:MGI Symbol;Acc:MGI:1916075] | 94,36673918 | -0,7740922 | **-1,71011366** | 6,55943321 | 31,1872167 | 2,34304E-08 | 3,48821E-07 | protein_coding |
| ENSMUSG00000046546 | Fam43a | family with sequence similarity 43, member A [Source:MGI Symbol;Acc:MGI:2676309] | 31,61548094 | -0,7742059 | **-1,71024841** | 4,9750817 | 15,6109652 | 7,78021E-05 | 0,000547956 | protein_coding |
| ENSMUSG00000067714 | Lpar5 | lysophosphatidic acid receptor 5 [Source:MGI Symbol;Acc:MGI:2685918] | 13,49167388 | -0,7742107 | **-1,71025417** | 3,71894203 | 15,4189757 | 8,61192E-05 | 0,000599234 | protein_coding |
| ENSMUSG00000045128 | Rpl18a | ribosomal protein L18A [Source:MGI Symbol;Acc:MGI:1924058] | 292,3968138 | -0,7755729 | **-1,71186968** | 8,19012062 | 23,8806561 | 1,02497E-06 | 1,11388E-05 | protein_coding |
| ENSMUSG00000026187 | Xrcc5 | X-ray repair complementing defective repair in Chinese hamster cells 5 [Source:MGI Symbol;Acc:MGI:104517] | 3,939777764 | -0,7761229 | **-1,71252239** | 1,97426019 | 5,95373888 | 0,014686105 | 0,049404848 | protein_coding |
| ENSMUSG00000031015 | Swap70 | SWA-70 protein [Source:MGI Symbol;Acc:MGI:1298390] | 241,9847967 | -0,7765529 | **-1,71303293** | 7,91786618 | 27,7128061 | 1,40728E-07 | 1,79163E-06 | protein_coding |
| ENSMUSG00000081344 | Gm14303 | predicted gene 14303 [Source:MGI Symbol;Acc:MGI:3651379] | 65,63684798 | -0,7765641 | **-1,71304625** | 6,03203105 | 24,876971 | 6,11081E-07 | 6,8861E-06 | pseudogene |
| ENSMUSG00000029672 | Fam3c | family with sequence similarity 3, member C [Source:MGI Symbol;Acc:MGI:107892] | 65,44912827 | -0,7767184 | **-1,71322942** | 6,02743565 | 32,2743026 | 1,33872E-08 | 2,08632E-07 | protein_coding |
| ENSMUSG00000005034 | Prkacb | protein kinase, cAMP dependent, catalytic, beta [Source:MGI Symbol;Acc:MGI:97594] | 223,3913217 | -0,7768289 | **-1,71336072** | 7,80242117 | 29,7879754 | 4,81972E-08 | 6,79772E-07 | protein_coding |
| ENSMUSG00000030410 | Dmwd | dystrophia myotonica-containing WD repeat motif [Source:MGI Symbol;Acc:MGI:94907] | 6,600628369 | -0,7786925 | **-1,71557532** | 2,81202589 | 7,49150229 | 0,006199082 | 0,024281395 | protein_coding |
| ENSMUSG00000066152 | Slc31a2 | solute carrier family 31, member 2 [Source:MGI Symbol;Acc:MGI:1333844] | 278,4538689 | -0,7814613 | **-1,71887104** | 8,12064916 | 26,0239148 | 3,37214E-07 | 3,97103E-06 | protein_coding |
| ENSMUSG00000020387 | Phf15 | PHD finger protein 15 [Source:MGI Symbol;Acc:MGI:1924151] | 224,405446 | -0,7829946 | **-1,72069886** | 7,8090106 | 24,7395144 | 6,56248E-07 | 7,36486E-06 | protein_coding |
| ENSMUSG00000087150 | BC064078 | cDNA sequence BC064078 [Source:MGI Symbol;Acc:MGI:3040692] | 5,083786841 | -0,7834414 | **-1,7212318** | 2,3270483 | 6,78766428 | 0,009178993 | 0,033842146 | pseudogene |
| ENSMUSG00000064363 | mt-Nd4 | mitochondrially encoded NADH dehydrogenase 4 [Source:MGI Symbol;Acc:MGI:102498] | 1003,262216 | -0,7840906 | **-1,72200656** | 9,97054526 | 16,8642042 | 4,01517E-05 | 0,000301124 | protein_coding |
| ENSMUSG00000020668 | Kif3c | kinesin family member 3C [Source:MGI Symbol;Acc:MGI:107979] | 216,8889826 | -0,7852534 | **-1,72339503** | 7,76110344 | 23,5992106 | 1,18636E-06 | 1,26426E-05 | protein_coding |
| ENSMUSG00000056185 | Snx32 | sorting nexin 32 [Source:MGI Symbol;Acc:MGI:2444704] | 14,46316453 | -0,7855132 | **-1,72370535** | 3,83511775 | 14,4009921 | 0,000147724 | 0,000960353 | protein_coding |
| ENSMUSG00000035199 | Arl6ip5 | ADP-ribosylation factor-like 6 interacting protein 5 [Source:MGI Symbol;Acc:MGI:1929501] | 202,0678457 | -0,7857255 | **-1,72395905** | 7,65804273 | 27,014968 | 2,01886E-07 | 2,46732E-06 | protein_coding |
| ENSMUSG00000038418 | Egr1 | early growth response 1 [Source:MGI Symbol;Acc:MGI:95295] | 8,607834931 | -0,7867311 | **-1,72516108** | 3,1031828 | 9,56879159 | 0,00197913 | 0,009284672 | protein_coding |
| ENSMUSG00000041841 | Rpl37 | ribosomal protein L37 [Source:MGI Symbol;Acc:MGI:1914531] | 112,4732139 | -0,7869164 | **-1,72538271** | 6,80919164 | 31,4841808 | 2,01072E-08 | 3,03875E-07 | protein_coding |
| ENSMUSG00000032047 | Acat1 | acetyl-Coenzyme A acetyltransferase 1 [Source:MGI Symbol;Acc:MGI:87870] | 39,99294607 | -0,7884378 | **-1,72720315** | 5,31706125 | 27,6109311 | 1,48338E-07 | 1,86256E-06 | protein_coding |
| ENSMUSG00000018401 | Mtmr4 | myotubularin related protein 4 [Source:MGI Symbol;Acc:MGI:2180699] | 56,29271645 | -0,7895824 | **-1,72857404** | 5,81080845 | 27,6357841 | 1,46444E-07 | 1,84728E-06 | protein_coding |
| ENSMUSG00000036908 | Unc93b1 | unc-93 homolog B1 (C. elegans) [Source:MGI Symbol;Acc:MGI:1859307] | 996,5263951 | -0,7898975 | **-1,72895161** | 9,96055077 | 15,2051329 | 9,64409E-05 | 0,000659774 | protein_coding |
| ENSMUSG00000024949 | Sf1 | splicing factor 1 [Source:MGI Symbol;Acc:MGI:1095403] | 305,5887729 | -0,7903804 | **-1,7295304** | 8,25432801 | 23,7917164 | 1,07343E-06 | 1,15853E-05 | protein_coding |
| ENSMUSG00000014503 | Pkd2l2 | polycystic kidney disease 2-like 2 [Source:MGI Symbol;Acc:MGI:1858231] | 5,680514841 | -0,7904847 | **-1,72965544** | 2,4734345 | 7,38229879 | 0,006586892 | 0,025572504 | protein_coding |
| ENSMUSG00000037685 | Atp8a1 | ATPase, aminophospholipid transporter (APLT), class I, type 8A, member 1 [Source:MGI Symbol;Acc:MGI:1330848] | 100,7279502 | -0,7913744 | **-1,73072246** | 6,65163876 | 33,8672247 | 5,90044E-09 | 9,74859E-08 | protein_coding |
| ENSMUSG00000030055 | Rab43 | RAB43, member RAS oncogene family [Source:MGI Symbol;Acc:MGI:1917084] | 183,0402721 | -0,7918697 | **-1,7313167** | 7,51456464 | 26,5143877 | 2,61583E-07 | 3,15478E-06 | protein_coding |
| ENSMUSG00000002105 | Slc39a13 | solute carrier family 39 (metal ion transporter), member 13 [Source:MGI Symbol;Acc:MGI:1915677] | 202,2400171 | -0,7923078 | **-1,73184255** | 7,6584573 | 20,2932493 | 6,64346E-06 | 6,02241E-05 | protein_coding |
| ENSMUSG00000018672 | Copz2 | coatomer protein complex, subunit zeta 2 [Source:MGI Symbol;Acc:MGI:1929008] | 5,64673868 | -0,7945455 | **-1,73453082** | 2,47820372 | 8,60822934 | 0,003346475 | 0,014498312 | protein_coding |
| ENSMUSG00000045193 | Cirbp | cold inducible RNA binding protein [Source:MGI Symbol;Acc:MGI:893588] | 19,39422206 | -0,7946099 | **-1,73460826** | 4,25612776 | 18,7802485 | 1,46678E-05 | 0,000122926 | protein_coding |
| ENSMUSG00000046364 | Rpl27a | ribosomal protein L27A [Source:MGI Symbol;Acc:MGI:1347076] | 50,56723316 | -0,7948943 | **-1,73495028** | 5,65482357 | 27,6070139 | 1,48639E-07 | 1,86256E-06 | protein_coding |
| ENSMUSG00000097164 | | | 18,93971041 | -0,7954464 | **-1,7356143** | 4,22427494 | 17,678862 | 2,61517E-05 | 0,00020597 | lincRNA |
| ENSMUSG00000051517 | Arhgef39 | Rho guanine nucleotide exchange factor (GEF) 39 [Source:MGI Symbol;Acc:MGI:3036286] | 5,275456821 | -0,7962649 | **-1,73659927** | 2,37236669 | 6,25847455 | 0,012360059 | 0,043103852 | protein_coding |
| ENSMUSG00000060244 | Alyref2 | Aly/REF export factor 2 [Source:MGI Symbol;Acc:MGI:1913144] | 8,576060227 | -0,7964989 | **-1,73688102** | 3,09076002 | 11,1768093 | 0,000828261 | 0,004360375 | protein_coding |
| ENSMUSG00000000579 | Dynlt1c | dynein light chain Tctex-type 1C [Source:MGI Symbol;Acc:MGI:3807476] | 16,29669056 | -0,7975345 | **-1,73812823** | 4,01191252 | 16,0853665 | 6,05501E-05 | 0,000437674 | protein_coding |
| ENSMUSG00000025153 | Fasn | fatty acid synthase [Source:MGI Symbol;Acc:MGI:95485] | 146,8265955 | -0,7980429 | **-1,73874077** | 7,19538966 | 12,2942307 | 0,000454361 | 0,00258121 | protein_coding |
| ENSMUSG00000020423 | Btg2 | B cell translocation gene 2, anti-proliferative [Source:MGI Symbol;Acc:MGI:108384] | 49,49467715 | -0,7985726 | **-1,73937937** | 5,62558928 | 23,5955904 | 1,1886E-06 | 1,26542E-05 | protein_coding |
| ENSMUSG00000034855 | Cxcl10 | chemokine (C-X-C motif) ligand 10 [Source:MGI Symbol;Acc:MGI:1352450] | 9,583670913 | -0,7988809 | **-1,73975111** | 3,29497418 | 10,7341407 | 0,001051768 | 0,00536231 | protein_coding |

| | | | | | | | | | | |
|---|---|---|---|---|---|---|---|---|---|---|
| ENSMUSG00000038608 | Dock10 | dedicator of cytokinesis 10 [Source:MGI Symbol;Acc:MGI:2146320] | 237,9570389 | -0,7991144 | **-1,74003264** | 7,89407568 | 32,3598546 | 1,28106E-08 | 2,00213E-07 | protein_coding |
| ENSMUSG00000026126 | Ptpn18 | protein tyrosine phosphatase, non-receptor type 18 [Source:MGI Symbol;Acc:MGI:108410] | 51,14010217 | -0,799781 | **-1,74083681** | 5,66471641 | 30,5391846 | 3,27191E-08 | 4,79953E-07 | protein_coding |
| ENSMUSG00000071415 | Rpl23 | ribosomal protein L23 [Source:MGI Symbol;Acc:MGI:1929455] | 762,7061744 | -0,7997951 | **-1,74085387** | 9,57466901 | 17,875341 | 2,35857E-05 | 0,000188326 | protein_coding |
| ENSMUSG00000030707 | Coro1a | coronin, actin binding protein 1A [Source:MGI Symbol;Acc:MGI:1345961] | 679,784878 | -0,8011493 | **-1,74248876** | 9,4086435 | 18,3293923 | 1,85818E-05 | 0,000151791 | protein_coding |
| ENSMUSG00000037972 | Snn | stannin [Source:MGI Symbol;Acc:MGI:1276549] | 8,389801038 | -0,8018581 | **-1,74334504** | 3,05238306 | 11,291659 | 0,000778561 | 0,004144403 | protein_coding |
| ENSMUSG00000058715 | Fcer1g | Fc receptor, IgE, high affinity I, gamma polypeptide [Source:MGI Symbol;Acc:MGI:95496] | 1087,976483 | -0,8029941 | **-1,74471828** | 10,087362 | 15,338387 | 8,98717E-05 | 0,00062141 | protein_coding |
| ENSMUSG00000081051 | Gm15427 | predicted pseudogene 15427 [Source:MGI Symbol;Acc:MGI:3642341] | 267,4312338 | -0,8041389 | **-1,74610325** | 8,06107442 | 27,0265529 | 2,0068E-07 | 2,45531E-06 | pseudogene |
| ENSMUSG00000043940 | Wdfy3 | WD repeat and FYVE domain containing 3 [Source:MGI Symbol;Acc:MGI:1096875] | 605,614365 | -0,8047499 | **-1,74684292** | 9,24201835 | 20,1047025 | 7,33162E-06 | 6,565E-05 | protein_coding |
| ENSMUSG00000029174 | Tbc1d1 | TBC1 domain family, member 1 [Source:MGI Symbol;Acc:MGI:1889508] | 72,88414531 | -0,8051218 | **-1,74729332** | 6,18409873 | 35,7018722 | 2,29943E-09 | 4,10179E-08 | protein_coding |
| ENSMUSG00000044162 | Tnip3 | TNFAIP3 interacting protein 3 [Source:MGI Symbol;Acc:MGI:3041165] | 159,8452027 | -0,8054973 | **-1,7477482** | 7,32091443 | 26,0721253 | 3,28898E-07 | 3,88978E-06 | protein_coding |
| ENSMUSG00000031349 | Nsdhl | NAD(P) dependent steroid dehydrogenase-like [Source:MGI Symbol;Acc:MGI:1099438] | 25,46535634 | -0,8072546 | **-1,74987827** | 4,66184184 | 14,1251637 | 0,00017104 | 0,001091301 | protein_coding |
| ENSMUSG00000033685 | Ucp2 | uncoupling protein 2 (mitochondrial, proton carrier) [Source:MGI Symbol;Acc:MGI:109354] | 785,5703503 | -0,8079175 | **-1,7506826** | 9,61722553 | 17,9998062 | 2,20927E-05 | 0,000177826 | protein_coding |
| ENSMUSG00000032652 | Crebl2 | cAMP responsive element binding protein-like 2 [Source:MGI Symbol;Acc:MGI:1889385] | 24,02202024 | -0,808298 | **-1,75114429** | 4,57503703 | 20,0347744 | 7,60466E-06 | 6,78185E-05 | protein_coding |
| ENSMUSG00000020321 | Mdh1 | malate dehydrogenase 1, NAD (soluble) [Source:MGI Symbol;Acc:MGI:97051] | 201,2274311 | -0,8086842 | **-1,75161316** | 7,65212715 | 34,2744376 | 4,78627E-09 | 8,01517E-08 | protein_coding |
| ENSMUSG00000025732 | Fam195a | family with sequence similarity 195, member A [Source:MGI Symbol;Acc:MGI:1915491] | 6,60842781 | -0,8088751 | **-1,75184501** | 2,6983698 | 9,1774353 | 0,002450171 | 0,011142397 | protein_coding |
| ENSMUSG00000006333 | Rps9 | ribosomal protein S9 [Source:MGI Symbol;Acc:MGI:1924096] | 693,2389835 | -0,8092056 | **-1,75224635** | 9,43672778 | 17,0404788 | 3,65914E-05 | 0,000277071 | protein_coding |
| ENSMUSG00000006442 | Srm | spermidine synthase [Source:MGI Symbol;Acc:MGI:102690] | 40,04337807 | -0,8099297 | **-1,75312604** | 5,31911343 | 28,5345723 | 9,20403E-08 | 1,2169E-06 | protein_coding |
| ENSMUSG00000004317 | Clcn5 | chloride channel 5 [Source:MGI Symbol;Acc:MGI:99486] | 135,2385888 | -0,8106489 | **-1,7540002** | 7,0785009 | 25,9001222 | 3,59546E-07 | 4,20249E-06 | protein_coding |
| ENSMUSG00000021311 | Mtr | 5-methyltetrahydrofolate-homocysteine methyltransferase [Source:MGI Symbol;Acc:MGI:894292] | 163,1780656 | -0,810726 | **-1,75409388** | 7,34947842 | 34,2717422 | 4,79291E-09 | 8,01517E-08 | protein_coding |
| ENSMUSG00000001270 | Ckb | creatine kinase, brain [Source:MGI Symbol;Acc:MGI:88407] | 488,345337 | -0,8125997 | **-1,7563735** | 8,93111113 | 24,1688194 | 8,82496E-07 | 9,7447E-06 | protein_coding |
| ENSMUSG00000007029 | Vars | valyl-tRNA synthetase [Source:MGI Symbol;Acc:MGI:90675] | 105,783746 | -0,8143704 | **-1,7585306** | 6,72082993 | 33,7799462 | 6,17117E-09 | 1,01047E-07 | protein_coding |
| ENSMUSG00000094856 | Gm17316 | predicted gene, 17316 [Source:MGI Symbol;Acc:MGI:4936950] | 8,26470372 | -0,8154126 | **-1,75980131** | 3,02382618 | 8,61062362 | 0,003342079 | 0,014484977 | protein_coding |
| ENSMUSG00000058793 | Cds2 | CDP-diacylglycerol synthase (phosphatidate cytidylyltransferase) 2 [Source:MGI Symbol;Acc:MGI:1332236] | 321,9005356 | -0,8154922 | **-1,75989852** | 8,32959639 | 28,7811548 | 8,10358E-08 | 1,08314E-06 | protein_coding |
| ENSMUSG00000003206 | Ebi3 | Epstein-Barr virus induced gene 3 [Source:MGI Symbol;Acc:MGI:1354171] | 261,5440388 | -0,8157544 | **-1,7602183** | 8,03008139 | 22,8682645 | 1,73493E-06 | 1,79151E-05 | protein_coding |
| ENSMUSG00000032589 | Bsn | bassoon [Source:MGI Symbol;Acc:MGI:1277955] | 35,4530684 | -0,8165064 | **-1,76113607** | 5,12975729 | 33,4618067 | 7,26772E-09 | 1,17774E-07 | protein_coding |
| ENSMUSG00000060036 | Rpl3 | ribosomal protein L3 [Source:MGI Symbol;Acc:MGI:1351605] | 771,7869024 | -0,8173758 | **-1,76219773** | 9,59179798 | 17,226812 | 3,31722E-05 | 0,000253981 | protein_coding |
| ENSMUSG00000079641 | Rpl39 | ribosomal protein L39 [Source:MGI Symbol;Acc:MGI:1914498] | 342,4493313 | -0,8174647 | **-1,76230628** | 8,41890959 | 23,8167788 | 1,05955E-06 | 1,14467E-05 | protein_coding |
| ENSMUSG00000026727 | Rsu1 | Ras suppressor protein 1 [Source:MGI Symbol;Acc:MGI:103040] | 122,4500831 | -0,8176305 | **-1,76250887** | 6,93504616 | 34,5625017 | 4,12782E-09 | 6,99882E-08 | protein_coding |
| ENSMUSG00000034731 | Dgkh | diacylglycerol kinase, eta [Source:MGI Symbol;Acc:MGI:2444188] | 44,04835597 | -0,8178221 | **-1,76274288** | 5,46446617 | 29,3688926 | 5,98304E-08 | 8,28165E-07 | protein_coding |
| ENSMUSG00000033450 | Tagap | T cell activation Rho GTPase activating protein [Source:MGI Symbol;Acc:MGI:3615484] | 119,3825986 | -0,818096 | **-1,76307761** | 6,8974629 | 23,5300872 | 1,22976E-06 | 1,30292E-05 | protein_coding |
| ENSMUSG00000002346 | Slc25a42 | solute carrier family 25, member 42 [Source:MGI Symbol;Acc:MGI:1920345] | 4,959244347 | -0,8190776 | **-1,76427763** | 2,29682917 | 7,94760455 | 0,004815107 | 0,019581555 | protein_coding |
| ENSMUSG00000040586 | Ofd1 | oral-facial-digital syndrome 1 gene homolog (human) [Source:MGI Symbol;Acc:MGI:1350328] | 29,44675575 | -0,8208187 | **-1,76640815** | 4,87748824 | 28,8743516 | 7,72288E-08 | 1,0424E-06 | protein_coding |
| ENSMUSG00000056091 | St3gal5 | ST3 beta-galactoside alpha-2,3-sialyltransferase 5 [Source:MGI Symbol;Acc:MGI:1339963] | 70,89973432 | -0,8213024 | **-1,76700048** | 6,15265849 | 39,6248965 | 3,07735E-10 | 6,39147E-09 | protein_coding |
| ENSMUSG00000002885 | Cd97 | CD97 antigen [Source:MGI Symbol;Acc:MGI:1347095] | 125,5640511 | -0,8213346 | **-1,76703985** | 6,97459878 | 32,41093 | 1,24783E-08 | 1,95576E-07 | protein_coding |
| ENSMUSG00000079470 | Utp14b | UTP14, U3 small nucleolar ribonucleoprotein, homolog B (yeast) [Source:MGI Symbol;Acc:MGI:2445092] | 10,00501442 | -0,8221637 | **-1,76805566** | 3,29961068 | 9,30523512 | 0,002285 | 0,010504307 | protein_coding |
| ENSMUSG00000066233 | Tmem42 | transmembrane protein 42 [Source:MGI Symbol;Acc:MGI:1277176] | 5,858634867 | -0,8224825 | **-1,76844638** | 2,51804378 | 8,38541033 | 0,003782448 | 0,016081539 | protein_coding |
| ENSMUSG00000064061 | Dzip3 | DAZ interacting protein 3, zinc finger [Source:MGI Symbol;Acc:MGI:1917433] | 22,9706537 | -0,8226645 | **-1,76866953** | 4,51288351 | 21,0552738 | 4,46223E-06 | 4,22642E-05 | protein_coding |
| ENSMUSG00000037999 | Arap2 | ArfGAP with RhoGAP domain, ankyrin repeat and PH domain 2 [Source:MGI Symbol;Acc:MGI:2684416] | 65,65104667 | -0,8234458 | **-1,76962762** | 6,03562456 | 39,2886904 | 3,65553E-10 | 7,47919E-09 | protein_coding |
| ENSMUSG00000040430 | Pitpnc1 | phosphatidylinositol transfer protein, cytoplasmic 1 [Source:MGI Symbol;Acc:MGI:1919045] | 328,6220991 | -0,8234624 | **-1,76964797** | 8,35988256 | 24,3191427 | 8,16239E-07 | 9,08613E-06 | protein_coding |
| ENSMUSG00000064141 | Zfp69 | zinc finger protein 69 [Source:MGI Symbol;Acc:MGI:107794] | 3,634825521 | -0,8235948 | **-1,76981036** | 1,84322322 | 6,01090364 | 0,014217745 | 0,048168474 | protein_coding |
| ENSMUSG00000022091 | Sorbs3 | sorbin and SH3 domain containing 3 [Source:MGI Symbol;Acc:MGI:700013] | 25,98617975 | -0,8238088 | **-1,77007295** | 4,68668937 | 24,0302908 | 9,4832E-07 | 1,04283E-05 | protein_coding |
| ENSMUSG00000060791 | Gmfg | glia maturation factor, gamma [Source:MGI Symbol;Acc:MGI:1927135] | 81,71960882 | -0,8242668 | **-1,7706349** | 6,35131286 | 26,8777929 | 2,16735E-07 | 2,63706E-06 | protein_coding |
| ENSMUSG00000000686 | Abhd15 | abhydrolase domain containing 15 [Source:MGI Symbol;Acc:MGI:1914727] | 4,569524419 | -0,8246324 | **-1,77108374** | 2,15636716 | 6,56667492 | 0,01039059 | 0,037528406 | protein_coding |
| ENSMUSG00000024248 | Cox7a2l | cytochrome c oxidase subunit VIIa polypeptide 2-like [Source:MGI Symbol;Acc:MGI:106015] | 195,3819 | -0,8270416 | **-1,77404376** | 7,60815126 | 27,436012 | 1,62379E-07 | 2,02504E-06 | protein_coding |
| ENSMUSG00000065947 | mt-Nd4l | mitochondrially encoded NADH dehydrogenase 4L [Source:MGI Symbol;Acc:MGI:102497] | 83,23003646 | -0,8280507 | **-1,77528505** | 6,38124257 | 38,8448185 | 4,5887E-10 | 9,26637E-09 | protein_coding |
| ENSMUSG00000067736 | Gm10222 | predicted gene 10222 [Source:MGI Symbol;Acc:MGI:3642643] | 83,23003646 | -0,8280513 | **-1,77528582** | 6,38124237 | 38,8415051 | 4,5965E-10 | 9,26637E-09 | protein_coding |
| ENSMUSG00000086841 | 2410006H16Rik | RIKEN cDNA 2410006H16 gene [Source:MGI Symbol;Acc:MGI:1916471] | 13,47604752 | -0,8282939 | **-1,77558429** | 3,71878092 | 17,121264 | 3,50675E-05 | 0,000266634 | lincRNA |
| ENSMUSG00000034252 | Senp6 | SUMO/sentrin specific peptidase 6 [Source:MGI Symbol;Acc:MGI:1922075] | 147,5893894 | -0,8290017 | **-1,77645568** | 7,20363742 | 33,5921375 | 6,79669E-09 | 1,10466E-07 | protein_coding |
| ENSMUSG00000008682 | Rpl10 | ribosomal protein L10 [Source:MGI Symbol;Acc:MGI:105943] | 529,0511044 | -0,8292885 | **-1,77680884** | 9,04668625 | 20,6306769 | 5,56965E-06 | 5,15099E-05 | protein_coding |
| ENSMUSG00000085586 | Gm11613 | predicted gene 11613 [Source:MGI Symbol;Acc:MGI:3651819] | 9,835056379 | -0,8309434 | **-1,77884824** | 3,27482039 | 14,7566045 | 0,000122318 | 0,000815212 | lincRNA |
| ENSMUSG00000069662 | Marcks | myristoylated alanine rich protein kinase C substrate [Source:MGI Symbol;Acc:MGI:96907] | 715,8908183 | -0,8318963 | **-1,78002349** | 9,48344446 | 16,2246212 | 5,62582E-05 | 0,000409615 | protein_coding |
| ENSMUSG00000061477 | Rps7 | ribosomal protein S7 [Source:MGI Symbol;Acc:MGI:1333818] | 401,1077967 | -0,8325839 | **-1,78087209** | 8,64664737 | 22,5952903 | 1,99974E-06 | 2,03625E-05 | protein_coding |
| ENSMUSG00000042292 | Mkl1 | MKL (megakaryoblastic leukemia)/myocardin-like 1 [Source:MGI Symbol;Acc:MGI:2384495] | 132,2382069 | -0,8331069 | **-1,78151784** | 7,04267051 | 35,2487633 | 2,90166E-09 | 5,02848E-08 | protein_coding |
| ENSMUSG00000031129 | Slc9a9 | solute carrier family 9 (sodium/hydrogen exchanger), member 9 [Source:MGI Symbol;Acc:MGI:2679732] | 91,26202754 | -0,8347619 | **-1,78356262** | 6,51070275 | 32,6717607 | 1,09111E-08 | 1,72738E-07 | protein_coding |
| ENSMUSG00000031925 | Maml2 | mastermind like 2 (Drosophila) [Source:MGI Symbol;Acc:MGI:2389460] | 19,40245057 | -0,8349505 | **-1,78379579** | 4,26413698 | 26,4112815 | 2,75924E-07 | 3,32004E-06 | protein_coding |
| ENSMUSG00000003031 | Cdkn1b | cyclin-dependent kinase inhibitor 1B [Source:MGI Symbol;Acc:MGI:104565] | 45,04947056 | -0,8350921 | **-1,78397095** | 5,48655035 | 27,4114123 | 1,64458E-07 | 2,04864E-06 | protein_coding |
| ENSMUSG00000024597 | Slc12a2 | solute carrier family 12, member 2 [Source:MGI Symbol;Acc:MGI:101924] | 20,22451588 | -0,8352292 | **-1,78414051** | 4,3157698 | 23,0390989 | 1,5874E-06 | 1,65159E-05 | protein_coding |
| ENSMUSG00000026548 | Slamf9 | SLAM family member 9 [Source:MGI Symbol;Acc:MGI:1923692] | 322,0262105 | -0,8357482 | **-1,78478241** | 8,33121267 | 29,30787 | 6,17444E-08 | 8,50108E-07 | protein_coding |
| ENSMUSG00000073702 | Rpl31 | ribosomal protein L31 [Source:MGI Symbol;Acc:MGI:2149632] | 18,42082005 | -0,8359434 | **-1,78502387** | 4,18350577 | 23,8823407 | 1,02407E-06 | 1,11388E-05 | protein_coding |
| ENSMUSG00000025150 | Cbr2 | carbonyl reductase 2 [Source:MGI Symbol;Acc:MGI:107200] | 5,682435729 | -0,8361612 | **-1,78529342** | 2,49933865 | 8,37365717 | 0,003806987 | 0,016155802 | protein_coding |
| ENSMUSG00000096463 | Gm17312 | predicted gene, 17312 [Source:MGI Symbol;Acc:MGI:4936946] | 6,446226372 | -0,8383615 | **-1,78801835** | 2,67465125 | 9,50658193 | 0,002047362 | 0,00956393 | protein_coding |
| ENSMUSG00000057841 | Rpl32 | ribosomal protein L32 [Source:MGI Symbol;Acc:MGI:98038] | 613,7567808 | -0,8387379 | **-1,78848488** | 9,26104433 | 22,0818699 | 2,61266E-06 | 2,60247E-05 | protein_coding |
| ENSMUSG00000064368 | mt-Nd6 | mitochondrially encoded NADH dehydrogenase 6 [Source:MGI Symbol;Acc:MGI:102495] | 239,7361925 | -0,8390546 | **-1,78887756** | 7,90610723 | 31,5002938 | 1,9941E-08 | 3,02196E-07 | protein_coding |
| ENSMUSG00000037706 | Cd81 | CD81 antigen [Source:MGI Symbol;Acc:MGI:1096398] | 276,3020122 | -0,8397797 | **-1,78977679** | 8,1102077 | 31,9952447 | 1,5455E-08 | 2,38155E-07 | protein_coding |
| ENSMUSG00000026496 | Parp1 | poly (ADP-ribose) polymerase family, member 1 [Source:MGI Symbol;Acc:MGI:1340806] | 198,5273791 | -0,8410431 | **-1,79134482** | 7,63205846 | 35,4516196 | 2,61465E-09 | 4,589E-08 | protein_coding |
| ENSMUSG00000045322 | Tlr9 | toll-like receptor 9 [Source:MGI Symbol;Acc:MGI:1932389] | 77,35176919 | -0,8413018 | **-1,79166608** | 6,26734707 | 25,4185137 | 4,61468E-07 | 5,31462E-06 | protein_coding |
| ENSMUSG00000028088 | Fmo5 | flavin containing monooxygenase 5 [Source:MGI Symbol;Acc:MGI:1310004] | 8,32352304 | -0,841403 | **-1,79179174** | 3,04902755 | 13,3817907 | 0,000254079 | 0,001548611 | protein_coding |
| ENSMUSG00000040723 | Rcsd1 | RCSD domain containing 1 [Source:MGI Symbol;Acc:MGI:2676394] | 73,56936826 | -0,8424985 | **-1,79315294** | 6,1976342 | 29,2651177 | 6,31218E-08 | 8,65817E-07 | protein_coding |
| ENSMUSG00000032640 | Chsy1 | chondroitin sulfate synthase 1 [Source:MGI Symbol;Acc:MGI:2681120] | 28,79756753 | -0,8438983 | **-1,79489359** | 4,84616255 | 22,9392174 | 1,67205E-06 | 1,73474E-05 | protein_coding |
| ENSMUSG00000004952 | Rasa4 | RAS p21 protein activator 4 [Source:MGI Symbol;Acc:MGI:1858600] | 456,4279968 | -0,8456553 | **-1,79708079** | 8,83367421 | 23,7215702 | 1,11328E-06 | 1,19449E-05 | protein_coding |
| ENSMUSG00000062997 | Rpl35 | ribosomal protein L35 [Source:MGI Symbol;Acc:MGI:1913739] | 180,4303064 | -0,8484453 | **-1,80055954** | 7,49249797 | 34,3459001 | 4,61371E-09 | 7,7509E-08 | protein_coding |

| | | | | | | | | | | |
|---|---|---|---|---|---|---|---|---|---|---|
| ENSMUSG00000042608 | Stk40 | serine/threonine kinase 40 [Source:MGI Symbol;Acc:MGI:1921428] | 118,0769613 | -0,8496561 | **-1,80207127** | 6,88193007 | 25,6537078 | 4,0851E-07 | 4,72037E-06 | protein_coding |
| ENSMUSG00000066677 | Pydc3 | pyrin domain containing 3 [Source:MGI Symbol;Acc:MGI:2442822] | 8,100218597 | -0,8509828 | **-1,8037292** | 3,02032775 | 10,3950776 | 0,001263517 | 0,006275886 | protein_coding |
| ENSMUSG00000030474 | Siglece | sialic acid binding Ig-like lectin E [Source:MGI Symbol;Acc:MGI:1932475] | 275,1176336 | -0,8514662 | **-1,80433371** | 8,10373601 | 19,8133974 | 8,53821E-06 | 7,53489E-05 | protein_coding |
| ENSMUSG00000030067 | Foxp1 | forkhead box P1 [Source:MGI Symbol;Acc:MGI:1914004] | 167,6585122 | -0,851474 | **-1,80434347** | 7,38891193 | 33,4028321 | 7,49148E-09 | 1,21221E-07 | protein_coding |
| ENSMUSG00000041040 | Fam117b | family with sequence similarity 117, member B [Source:MGI Symbol;Acc:MGI:1920000] | 61,50818256 | -0,8515617 | **-1,80445321** | 5,93679049 | 32,4636578 | 1,21442E-08 | 1,90613E-07 | protein_coding |
| ENSMUSG00000036501 | Fam13b | family with sequence similarity 13, member B [Source:MGI Symbol;Acc:MGI:2447834] | 66,16659749 | -0,8529727 | **-1,80621884** | 6,0419527 | 30,1050157 | 4,09271E-08 | 5,83225E-07 | protein_coding |
| ENSMUSG00000031390 | Avpr2 | arginine vasopressin receptor 2 [Source:MGI Symbol;Acc:MGI:88123] | 10,4451573 | -0,8530389 | **-1,80630173** | 3,36544442 | 12,3524246 | 0,000440414 | 0,002505867 | protein_coding |
| ENSMUSG00000041483 | Zfp281 | zinc finger protein 281 [Source:MGI Symbol;Acc:MGI:3029290] | 90,25342904 | -0,8531261 | **-1,8064109** | 6,49415075 | 35,6613938 | 2,34771E-09 | 4,18059E-08 | protein_coding |
| ENSMUSG00000076431 | Sox4 | SRY-box containing gene 4 [Source:MGI Symbol;Acc:MGI:98366] | 3,567541387 | -0,8539666 | **-1,80746364** | 1,83954464 | 6,87642537 | 0,008734007 | 0,03244102 | protein_coding |
| ENSMUSG00000026663 | Atf6 | activating transcription factor 6 [Source:MGI Symbol;Acc:MGI:1926157] | 179,9374224 | -0,8547094 | **-1,80839449** | 7,49082626 | 33,6010049 | 6,76578E-09 | 1,10127E-07 | protein_coding |
| ENSMUSG00000097077 | | | 10,355268 | -0,8556792 | **-1,80961044** | 3,36400225 | 11,1621904 | 0,000834813 | 0,00438856 | lincRNA |
| ENSMUSG00000037822 | 1110003E01Rik | RIKEN cDNA 1110003E01 gene [Source:MGI Symbol;Acc:MGI:1915802] | 155,161007 | -0,8563148 | **-1,81040792** | 7,27297754 | 31,2596207 | 2,25726E-08 | 3,36964E-07 | protein_coding |
| ENSMUSG00000097347 | | | 13,29353872 | -0,8572826 | **-1,81162278** | 3,71692286 | 17,2548062 | 3,2687E-05 | 0,00025079 | lincRNA |
| ENSMUSG00000017707 | Serinc3 | serine incorporator 3 [Source:MGI Symbol;Acc:MGI:1349457] | 1081,031198 | -0,8585408 | **-1,81320346** | 10,078071 | 18,3294004 | 1,85817E-05 | 0,000151791 | protein_coding |
| ENSMUSG00000038481 | Cdk19 | cyclin-dependent kinase 19 [Source:MGI Symbol;Acc:MGI:1925584] | 182,8720183 | -0,8604438 | **-1,81559678** | 7,51310123 | 34,022342 | 5,44829E-09 | 9,05603E-08 | protein_coding |
| ENSMUSG00000019876 | Pkib | protein kinase inhibitor beta, cAMP dependent, testis specific [Source:MGI Symbol;Acc:MGI:101937] | 38,69246896 | -0,8613527 | **-1,81674094** | 5,26628998 | 29,0282723 | 7,13297E-08 | 9,72331E-07 | protein_coding |
| ENSMUSG00000022292 | Rrm2b | ribonucleotide reductase M2 B (TP53 inducible) [Source:MGI Symbol;Acc:MGI:2155865] | 26,86583505 | -0,8613944 | **-1,81679344** | 4,74139839 | 32,5631637 | 1,15381E-08 | 1,81878E-07 | protein_coding |
| ENSMUSG00000035847 | Ids | iduronate 2-sulfatase [Source:MGI Symbol;Acc:MGI:96417] | 107,5466933 | -0,8628955 | **-1,81868471** | 6,74462972 | 38,8752732 | 4,51767E-10 | 9,15787E-09 | protein_coding |
| ENSMUSG00000058443 | Rpl10-ps3 | ribosomal protein L10, pseudogene 3 [Source:MGI Symbol;Acc:MGI:3704336] | 561,2135631 | -0,8676484 | **-1,82468625** | 9,13189867 | 21,2226064 | 4,08913E-06 | 3,91012E-05 | protein_coding |
| ENSMUSG00000039007 | Pgcp | plasma glutamate carboxypeptidase [Source:MGI Symbol;Acc:MGI:1889205] | 46,84625736 | -0,8677427 | **-1,82480553** | 5,54487658 | 38,7297905 | 4,86724E-10 | 9,74068E-09 | protein_coding |
| ENSMUSG00000036452 | Arhgap26 | Rho GTPase activating protein 26 [Source:MGI Symbol;Acc:MGI:1918552] | 61,66263072 | -0,8698825 | **-1,8275141** | 5,94567037 | 26,9526247 | 2,08504E-07 | 2,54254E-06 | protein_coding |
| ENSMUSG00000032018 | Sc5d | sterol-C5-desaturase (fungal ERG3, delta-5-desaturase) homolog (S. cerevisae) [Source:MGI Symbol;Acc:MGI:1353611 | 37,80850977 | -0,8707437 | **-1,82860534** | 5,23219488 | 15,9497157 | 6,50475E-05 | 0,000466195 | protein_coding |
| ENSMUSG00000074671 | Tspyl3 | TSPY-like 3 [Source:MGI Symbol;Acc:MGI:2139328] | 21,01111998 | -0,8714851 | **-1,82954525** | 4,380145 | 20,3806447 | 6,34685E-06 | 5,78216E-05 | protein_coding |
| ENSMUSG00000021203 | Otub2 | OTU domain, ubiquitin aldehyde binding 2 [Source:MGI Symbol;Acc:MGI:1915399] | 11,22728106 | -0,872107 | **-1,83033408** | 3,49757668 | 18,3327344 | 1,85492E-05 | 0,000151791 | protein_coding |
| ENSMUSG00000081604 | Gm11518 | predicted gene 11518 [Source:MGI Symbol;Acc:MGI:3650306] | 244,2079514 | -0,8732364 | **-1,83176745** | 7,93023792 | 25,2411941 | 5,059E-07 | 5,7839E-06 | pseudogene |
| ENSMUSG00000026786 | Apbb1ip | amyloid beta (A4) precursor protein-binding, family B, member 1 interacting protein [Source:MGI Symbol;Acc:MGI:18613 | 672,920734 | -0,8749738 | **-1,83397476** | 9,39408144 | 21,7032142 | 3,18258E-06 | 3,11094E-05 | protein_coding |
| ENSMUSG00000036587 | Fut7 | fucosyltransferase 7 [Source:MGI Symbol;Acc:MGI:107692] | 3,544335005 | -0,8750257 | **-1,83404079** | 1,8217625 | 7,45153838 | 0,006338225 | 0,024747009 | protein_coding |
| ENSMUSG00000078716 | Tmem8b | transmembrane protein 8B [Source:MGI Symbol;Acc:MGI:2441680] | 5,682023059 | -0,8755295 | **-1,83468129** | 2,4958879 | 8,31899858 | 0,003923254 | 0,016540597 | protein_coding |
| ENSMUSG00000021870 | Slmap | sarcolemma associated protein [Source:MGI Symbol;Acc:MGI:1933549] | 173,2952014 | -0,8761166 | **-1,83542814** | 7,43542625 | 41,1762234 | 1,39104E-10 | 3,02642E-09 | protein_coding |
| ENSMUSG00000036067 | Slc2a6 | solute carrier family 2 (facilitated glucose transporter), member 6 [Source:MGI Symbol;Acc:MGI:2443286] | 45,1029185 | -0,8764349 | **-1,83583315** | 5,48689187 | 15,9498604 | 6,50426E-05 | 0,000466195 | protein_coding |
| ENSMUSG00000032997 | Chpf | chondroitin polymerizing factor [Source:MGI Symbol;Acc:MGI:106576] | 29,75391756 | -0,8776387 | **-1,8373656** | 4,89627619 | 30,4132114 | 3,49142E-08 | 5,07411E-07 | protein_coding |
| ENSMUSG00000072653 | Zfp783 | zinc finger protein 783 [Source:MGI Symbol;Acc:MGI:3040704] | 7,565009317 | -0,8777666 | **-1,83752849** | 2,89798109 | 12,9852867 | 0,000313948 | 0,001860491 | pseudogene |
| ENSMUSG00000026004 | Kansl1l | KAT8 regulatory NSL complex subunit 1-like [Source:MGI Symbol;Acc:MGI:1915941] | 28,94121067 | -0,8782371 | **-1,83812786** | 4,85465531 | 33,8527911 | 5,94437E-09 | 9,80643E-08 | protein_coding |
| ENSMUSG00000030088 | Aldh1l1 | aldehyde dehydrogenase 1 family, member L1 [Source:MGI Symbol;Acc:MGI:1340024] | 16,75321892 | -0,8783431 | **-1,83826289** | 4,04438902 | 15,8178404 | 6,97418E-05 | 0,000495957 | protein_coding |
| ENSMUSG00000025823 | Pdia4 | protein disulfide isomerase associated 4 [Source:MGI Symbol;Acc:MGI:104864] | 139,1027438 | -0,878456 | **-1,83840675** | 7,11871544 | 31,3343306 | 2,17205E-08 | 3,26015E-07 | protein_coding |
| ENSMUSG00000034892 | Rps29 | ribosomal protein S29 [Source:MGI Symbol;Acc:MGI:107681] | 509,840781 | -0,8793017 | **-1,83948476** | 8,99298177 | 23,6125216 | 1,17818E-06 | 1,25799E-05 | protein_coding |
| ENSMUSG00000020340 | Cyfip2 | cytoplasmic FMR1 interacting protein 2 [Source:MGI Symbol;Acc:MGI:1924134] | 179,855158 | -0,8802182 | **-1,84065362** | 7,48922063 | 42,4257479 | 7,34171E-11 | 1,67005E-09 | protein_coding |
| ENSMUSG00000080848 | Gm9385 | predicted pseudogene 9385 [Source:MGI Symbol;Acc:MGI:3646182] | 11,02450549 | -0,8824176 | **-1,84346192** | 3,44013034 | 11,9796235 | 0,000537855 | 0,002992105 | pseudogene |
| ENSMUSG00000026764 | Kif5c | kinesin family member 5C [Source:MGI Symbol;Acc:MGI:1098269] | 4,481693251 | -0,8834213 | **-1,84474486** | 2,14195453 | 8,52329317 | 0,003506294 | 0,015094242 | protein_coding |
| ENSMUSG00000055184 | Fam72a | family with sequence similarity 72, member A [Source:MGI Symbol;Acc:MGI:1919669] | 9,56343076 | -0,8839242 | **-1,84538802** | 3,25512171 | 14,292769 | 0,000156465 | 0,001012414 | protein_coding |
| ENSMUSG00000019194 | Scn1b | sodium channel, voltage-gated, type I, beta [Source:MGI Symbol;Acc:MGI:98247] | 85,00465085 | -0,8839248 | **-1,84538875** | 6,40498021 | 38,4084564 | 5,73832E-10 | 1,12383E-08 | protein_coding |
| ENSMUSG00000052949 | Rnf157 | ring finger protein 157 [Source:MGI Symbol;Acc:MGI:2442484] | 44,67881962 | -0,8843998 | **-1,84599649** | 5,47613199 | 33,2690766 | 8,02486E-09 | 1,28902E-07 | protein_coding |
| ENSMUSG00000052160 | Pld4 | phospholipase D family, member 4 [Source:MGI Symbol;Acc:MGI:2144765] | 457,9130982 | -0,8865959 | **-1,84880862** | 8,83839889 | 22,1305156 | 2,54728E-06 | 2,54427E-05 | protein_coding |
| ENSMUSG00000020573 | Pik3cg | phosphoinositide-3-kinase, catalytic, gamma polypeptide [Source:MGI Symbol;Acc:MGI:1353576] | 313,7575212 | -0,8866351 | **-1,84885891** | 8,29320801 | 30,4375367 | 3,44791E-08 | 5,0274E-07 | protein_coding |
| ENSMUSG00000032643 | Fhl3 | four and a half LIM domains 3 [Source:MGI Symbol;Acc:MGI:1341092] | 165,5720875 | -0,8871167 | **-1,84947618** | 7,36907922 | 37,0432346 | 1,15539E-09 | 2,14793E-08 | protein_coding |
| ENSMUSG00000037235 | Mxd4 | Max dimerization protein 4 [Source:MGI Symbol;Acc:MGI:104991] | 46,98172537 | -0,8881383 | **-1,85078633** | 5,5432609 | 34,8175345 | 3,62098E-09 | 6,19208E-08 | protein_coding |
| ENSMUSG00000033220 | Rac2 | RAS-related C3 botulinum substrate 2 [Source:MGI Symbol;Acc:MGI:97846] | 1968,482575 | -0,8887274 | **-1,85154216** | 10,9428043 | 14,405139 | 0,0001474 | 0,000959407 | protein_coding |
| ENSMUSG00000043832 | Clec4a3 | C-type lectin domain family 4, member a3 [Source:MGI Symbol;Acc:MGI:1920399] | 146,3374594 | -0,8892781 | **-1,85224904** | 7,19187489 | 29,1710862 | 6,62607E-08 | 9,06608E-07 | protein_coding |
| ENSMUSG00000024241 | Sos1 | son of sevenless homolog 1 (Drosophila) [Source:MGI Symbol;Acc:MGI:98354] | 145,1684144 | -0,8909307 | **-1,85437202** | 7,18120348 | 27,9936277 | 1,21716E-07 | 1,57328E-06 | protein_coding |
| ENSMUSG00000056130 | Ticam2 | toll-like receptor adaptor molecule 2 [Source:MGI Symbol;Acc:MGI:3040056] | 70,48056436 | -0,8913437 | **-1,85490291** | 6,13423558 | 33,5855148 | 6,81987E-09 | 1,10679E-07 | protein_coding |
| ENSMUSG00000027993 | Trim2 | tripartite motif-containing 2 [Source:MGI Symbol;Acc:MGI:1933163] | 41,96599685 | -0,8917995 | **-1,85548907** | 5,37508423 | 38,1079856 | 6,69356E-10 | 1,29704E-08 | protein_coding |
| ENSMUSG00000020571 | Pdia6 | protein disulfide isomerase associated 6 [Source:MGI Symbol;Acc:MGI:1919103] | 277,9218673 | -0,8936756 | **-1,85790355** | 8,1181833 | 29,2829635 | 6,25432E-08 | 8,60027E-07 | protein_coding |
| ENSMUSG00000041939 | Mvk | mevalonate kinase [Source:MGI Symbol;Acc:MGI:107624] | 14,72405127 | -0,8937334 | **-1,85797802** | 3,86729899 | 10,3202002 | 0,00131582 | 0,006502836 | protein_coding |
| ENSMUSG00000090035 | Galnt4 | UDP-N-acetyl-alpha-D-galactosamine:polypeptide N-acetylgalactosaminyltransferase 4 [Source:MGI Symbol;Acc:MGI:8 | 26,07980075 | -0,89451 | **-1,8589784** | 4,69725595 | 25,1955144 | 5,18025E-07 | 5,91022E-06 | protein_coding |
| ENSMUSG00000025630 | Hprt | hypoxanthine guanine phosphoribosyl transferase [Source:MGI Symbol;Acc:MGI:96217] | 304,7180232 | -0,8947408 | **-1,85927583** | 8,25106171 | 34,5974549 | 4,05436E-09 | 6,89555E-08 | protein_coding |
| ENSMUSG00000021273 | Fdft1 | farnesyl diphosphate farnesyl transferase 1 [Source:MGI Symbol;Acc:MGI:102706] | 27,31861759 | -0,8952695 | **-1,85995735** | 4,76361254 | 9,38909332 | 0,002182801 | 0,010106379 | protein_coding |
| ENSMUSG00000028826 | Tmem57 | transmembrane protein 57 [Source:MGI Symbol;Acc:MGI:1913396] | 63,95384686 | -0,8954236 | **-1,86015597** | 5,99339314 | 36,4924831 | 1,53256E-09 | 2,80171E-08 | protein_coding |
| ENSMUSG00000036305 | Rpl39-ps | ribosomal protein L39, pseudogene [Source:MGI Symbol;Acc:MGI:3705781] | 48,98878116 | -0,8960327 | **-1,86094144** | 5,60934182 | 38,3350691 | 5,95822E-10 | 1,15864E-08 | pseudogene |
| ENSMUSG00000021880 | Rnase6 | ribonuclease, RNase A family, 6 [Source:MGI Symbol;Acc:MGI:1925666] | 2,793395233 | -0,8960362 | **-1,86094599** | 1,45625279 | 6,148133 | 0,01315509 | 0,045337195 | protein_coding |
| ENSMUSG00000038594 | Gm9766 | predicted gene 9766 [Source:MGI Symbol;Acc:MGI:3642684] | 11,4259212 | -0,8979701 | **-1,86344218** | 3,50490106 | 14,5589792 | 0,00013584 | 0,00089156 | protein_coding |
| ENSMUSG00000041729 | Coro2b | coronin, actin binding protein, 2B [Source:MGI Symbol;Acc:MGI:2444283] | 8,195849962 | -0,8980888 | **-1,86359556** | 3,02681018 | 10,7332486 | 0,001052276 | 0,005362408 | protein_coding |
| ENSMUSG00000004266 | Ptpn6 | protein tyrosine phosphatase, non-receptor type 6 [Source:MGI Symbol;Acc:MGI:96055] | 671,9564991 | -0,8983077 | **-1,86387837** | 9,39193967 | 22,0463389 | 2,66147E-06 | 2,64869E-05 | protein_coding |
| ENSMUSG00000061111 | Fam195b | family with sequence similarity 195, member B [Source:MGI Symbol;Acc:MGI:2384752] | 26,90963699 | -0,8993094 | **-1,86517296** | 4,72980311 | 29,5345154 | 5,49297E-08 | 7,66853E-07 | protein_coding |
| ENSMUSG00000024235 | Map3k8 | mitogen-activated protein kinase kinase kinase 8 [Source:MGI Symbol;Acc:MGI:1346878] | 62,12306449 | -0,9011831 | **-1,86759694** | 5,95398149 | 28,4561516 | 9,58447E-08 | 1,26416E-06 | protein_coding |
| ENSMUSG00000049091 | Sephs2 | selenophosphate synthetase 2 [Source:MGI Symbol;Acc:MGI:108388] | 127,1836731 | -0,9011971 | **-1,867615** | 6,98865768 | 38,5806209 | 5,2538E-10 | 1,04572E-08 | protein_coding |
| ENSMUSG00000028234 | Rps20 | ribosomal protein S20 [Source:MGI Symbol;Acc:MGI:1914677] | 510,2276473 | -0,9036779 | **-1,8708293** | 8,99428635 | 24,0601086 | 9,33748E-07 | 1,029E-05 | protein_coding |
| ENSMUSG00000003500 | Impdh1 | inosine 5'-phosphate dehydrogenase 1 [Source:MGI Symbol;Acc:MGI:96567] | 98,08906683 | -0,9043142 | **-1,87165458** | 6,6111801 | 42,4426878 | 7,2784E-11 | 1,65908E-09 | protein_coding |
| ENSMUSG00000091971 | Hspa1a | heat shock protein 1A [Source:MGI Symbol;Acc:MGI:96244] | 15,24585502 | -0,9067562 | **-1,87482529** | 3,96818127 | 24,2799919 | 8,32999E-07 | 9,25395E-06 | protein_coding |
| ENSMUSG00000053559 | Smagp | small cell adhesion glycoprotein [Source:MGI Symbol;Acc:MGI:2448476] | 18,21192455 | -0,9081349 | **-1,87661784** | 4,17680746 | 25,6740057 | 4,04236E-07 | 4,68002E-06 | protein_coding |
| ENSMUSG00000032067 | Pts | 6-pyruvoyl-tetrahydropterin synthase [Source:MGI Symbol;Acc:MGI:1338783] | 13,04448579 | -0,9083884 | **-1,87694762** | 3,69710818 | 19,4289733 | 1,04411E-05 | 9,0115E-05 | protein_coding |
| ENSMUSG00000073125 | Xlr3b | X-linked lymphocyte-regulated 3B [Source:MGI Symbol;Acc:MGI:109505] | 5,562001994 | -0,9099257 | **-1,87894873** | 2,43752644 | 9,05543563 | 0,00261915 | 0,011774386 | protein_coding |

| | | | | | | | | | | |
|---|---|---|---|---|---|---|---|---|---|---|
| ENSMUSG00000008496 | Pou2f2 | POU domain, class 2, transcription factor 2 [Source:MGI Symbol;Acc:MGI:101897] | 133,529302 | -0,9099768 | **-1,87901523** | 7,05820199 | 37,0923268 | 1,12666E-09 | 2,10521E-08 | protein_coding |
| ENSMUSG00000032527 | Pccb | propionyl Coenzyme A carboxylase, beta polypeptide [Source:MGI Symbol;Acc:MGI:1914154] | 37,79653494 | -0,9108404 | **-1,88014034** | 5,23032965 | 38,8153845 | 4,65842E-10 | 9,37151E-09 | protein_coding |
| ENSMUSG00000085786 | Gm15987 | predicted gene 15987 [Source:MGI Symbol;Acc:MGI:3801849] | 32,0858218 | -0,9125533 | **-1,88237395** | 4,99879756 | 33,1942378 | 8,33973E-09 | 1,33569E-07 | antisense |
| ENSMUSG00000002058 | Unc119 | unc-119 homolog (C. elegans) [Source:MGI Symbol;Acc:MGI:1328357] | 80,14027911 | -0,9146501 | **-1,88511182** | 6,31993528 | 42,0947272 | 8,69573E-11 | 1,95378E-09 | protein_coding |
| ENSMUSG00000097551 | | | 3,459892316 | -0,9190596 | **-1,8908823** | 1,76488702 | 6,95426791 | 0,008361951 | 0,031238611 | lincRNA |
| ENSMUSG00000000876 | Pxmp4 | peroxisomal membrane protein 4 [Source:MGI Symbol;Acc:MGI:1891701] | 15,22624755 | -0,9191586 | **-1,89101217** | 3,91739879 | 20,3545483 | 6,434E-06 | 5,84701E-05 | protein_coding |
| ENSMUSG00000022912 | Pros1 | protein S (alpha) [Source:MGI Symbol;Acc:MGI:1095733] | 52,27197985 | -0,9193408 | **-1,89125093** | 5,70416945 | 42,974161 | 5,54676E-11 | 1,29389E-09 | protein_coding |
| ENSMUSG00000043300 | B3galnt1 | UDP-GalNAc:betaGlcNAc beta 1,3-galactosaminyltransferase, polypeptide 1 [Source:MGI Symbol;Acc:MGI:1349405] | 136,3905077 | -0,921736 | **-1,89439341** | 7,08994777 | 35,2874546 | 2,84459E-09 | 4,93736E-08 | protein_coding |
| ENSMUSG00000026158 | Ogfrl1 | opioid growth factor receptor-like 1 [Source:MGI Symbol;Acc:MGI:1917405] | 42,64498767 | -0,9217414 | **-1,89440054** | 5,4157657 | 35,347652 | 2,75801E-09 | 4,80226E-08 | protein_coding |
| ENSMUSG00000019189 | Rnf145 | ring finger protein 145 [Source:MGI Symbol;Acc:MGI:1921565] | 103,1575364 | -0,9251567 | **-1,89889044** | 6,68659093 | 29,4031018 | 5,87835E-08 | 8,16503E-07 | protein_coding |
| ENSMUSG00000042349 | Ikbke | inhibitor of kappaB kinase epsilon [Source:MGI Symbol;Acc:MGI:1929612] | 559,3339831 | -0,9255928 | **-1,89946461** | 9,12727194 | 21,1258297 | 4,30092E-06 | 4,09127E-05 | protein_coding |
| ENSMUSG00000026807 | Ak8 | adenylate kinase 8 [Source:MGI Symbol;Acc:MGI:1916120] | 4,195587464 | -0,9275676 | **-1,90206636** | 2,03601412 | 8,15619382 | 0,004291434 | 0,017832824 | protein_coding |
| ENSMUSG00000042700 | Sipa1l1 | signal-induced proliferation-associated 1 like 1 [Source:MGI Symbol;Acc:MGI:2443679] | 227,5624252 | -0,9281426 | **-1,90282467** | 7,82945014 | 30,7021815 | 3,00827E-08 | 4,43649E-07 | protein_coding |
| ENSMUSG00000037447 | Arid5a | AT rich interactive domain 5A (MRF1-like) [Source:MGI Symbol;Acc:MGI:2443039] | 11,4630867 | -0,9286409 | **-1,90348196** | 3,5138264 | 10,7135583 | 0,001063533 | 0,005407236 | protein_coding |
| ENSMUSG00000084416 | Rpl10a-ps1 | ribosomal protein L10A, pseudogene 1 [Source:MGI Symbol;Acc:MGI:3705885] | 338,5004008 | -0,9292602 | **-1,9042993** | 8,4020877 | 27,8046158 | 1,34206E-07 | 1,71655E-06 | pseudogene |
| ENSMUSG00000050912 | Tmem123 | transmembrane protein 123 [Source:MGI Symbol;Acc:MGI:1919179] | 243,443291 | -0,9299788 | **-1,90524801** | 7,92652641 | 26,9644324 | 2,07234E-07 | 2,52987E-06 | protein_coding |
| ENSMUSG00000026000 | Lancl1 | LanC (bacterial lantibiotic synthetase component C)-like 1 [Source:MGI Symbol;Acc:MGI:1336997] | 42,91871011 | -0,9301309 | **-1,90544886** | 5,42425987 | 35,1525667 | 3,04859E-09 | 5,26649E-08 | protein_coding |
| ENSMUSG00000014837 | 4931428F04Rik | RIKEN cDNA 4931428F04 gene [Source:MGI Symbol;Acc:MGI:1921606] | 3,906173063 | -0,9302337 | **-1,90558463** | 1,95599282 | 8,90895371 | 0,002837758 | 0,012597353 | protein_coding |
| ENSMUSG00000032514 | Ttc21a | tetratricopeptide repeat domain 21A [Source:MGI Symbol;Acc:MGI:1921302] | 8,835731256 | -0,9326537 | **-1,90878384** | 3,12028219 | 13,4384438 | 0,00024652 | 0,001508047 | protein_coding |
| ENSMUSG00000018459 | Slc13a3 | solute carrier family 13 (sodium-dependent dicarboxylate transporter), member 3 [Source:MGI Symbol;Acc:MGI:214963 | 11817,41824 | -0,9352428 | **-1,91221236** | 13,5286226 | 9,91046623 | 0,001643415 | 0,007929819 | protein_coding |
| ENSMUSG00000026770 | Il2ra | interleukin 2 receptor, alpha chain [Source:MGI Symbol;Acc:MGI:96549] | 53,07962721 | -0,9353318 | **-1,91233045** | 5,7389944 | 29,8861607 | 4,58171E-08 | 6,48702E-07 | protein_coding |
| ENSMUSG00000050530 | Fam171a1 | family with sequence similarity 171, member A1 [Source:MGI Symbol;Acc:MGI:2442917] | 2,534961147 | -0,9377234 | **-1,91550321** | 1,34594409 | 6,18359118 | 0,012894032 | 0,044605393 | protein_coding |
| ENSMUSG00000028518 | Prkaa2 | protein kinase, AMP-activated, alpha 2 catalytic subunit [Source:MGI Symbol;Acc:MGI:1336173] | 4,075906662 | -0,9387202 | **-1,9168271** | 1,98279854 | 7,33901277 | 0,006747389 | 0,026103365 | protein_coding |
| ENSMUSG00000038437 | Mllt6 | myeloid/lymphoid or mixed-lineage leukemia (trithorax homolog, Drosophila); translocated to, 6 [Source:MGI Symbol;Acc | 247,4038941 | -0,9388919 | **-1,91705528** | 7,94940074 | 28,7980317 | 8,03327E-08 | 1,07636E-06 | protein_coding |
| ENSMUSG00000059089 | Fcgr4 | Fc receptor, IgG, low affinity IV [Source:MGI Symbol;Acc:MGI:2179523] | 219,2574606 | -0,9391786 | **-1,91743619** | 7,77597614 | 19,2635696 | 1,13858E-05 | 9,74268E-05 | protein_coding |
| ENSMUSG00000097723 | | | 2,823047758 | -0,9393791 | **-1,91770272** | 1,46934798 | 6,93089405 | 0,008471933 | 0,031606494 | lincRNA |
| ENSMUSG00000021238 | Aldh6a1 | aldehyde dehydrogenase family 6, subfamily A1 [Source:MGI Symbol;Acc:MGI:1915077] | 53,76356696 | -0,9402106 | **-1,91880827** | 5,74455476 | 39,313376 | 3,6096E-10 | 7,39901E-09 | protein_coding |
| ENSMUSG00000079488 | Cd47 | CD47 antigen (Rh-related antigen, integrin-associated signal transducer) [Source:MGI Symbol;Acc:MGI:96617] | 7,8884989 | -0,940213 | **-1,91881151** | 2,96922583 | 9,7399058 | 0,001803101 | 0,008616404 | ocessed_pseudoge |
| ENSMUSG00000030403 | Vasp | vasodilator-stimulated phosphoprotein [Source:MGI Symbol;Acc:MGI:109268] | 523,2084158 | -0,9402673 | **-1,91888372** | 9,03084548 | 23,2606994 | 1,41461E-06 | 1,48447E-05 | protein_coding |
| ENSMUSG00000028465 | Tln1 | talin 1 [Source:MGI Symbol;Acc:MGI:1099832] | 1697,376432 | -0,9406664 | **-1,91941468** | 10,728986 | 17,6759274 | 2,6192E-05 | 0,000205993 | protein_coding |
| ENSMUSG00000052760 | A630001G21Rik | RIKEN cDNA A630001G21 gene [Source:MGI Symbol;Acc:MGI:2443131] | 119,9885939 | -0,9407296 | **-1,91949875** | 6,90363702 | 35,7016277 | 2,29972E-09 | 4,10179E-08 | protein_coding |
| ENSMUSG00000031880 | Rrad | Ras-related associated with diabetes [Source:MGI Symbol;Acc:MGI:1930943] | 11,06886384 | -0,9414482 | **-1,92045504** | 3,45973341 | 21,1524782 | 4,24152E-06 | 4,03827E-05 | protein_coding |
| ENSMUSG00000009731 | Kcnd1 | potassium voltage-gated channel, Shal-related family, member 1 [Source:MGI Symbol;Acc:MGI:96671] | 13,81316213 | -0,9419317 | **-1,92109878** | 3,7712126 | 14,7560811 | 0,000122352 | 0,000815212 | protein_coding |
| ENSMUSG00000024238 | Zeb1 | zinc finger E-box binding homeobox 1 [Source:MGI Symbol;Acc:MGI:1344313] | 9,878312499 | -0,9422154 | **-1,92147664** | 3,2805103 | 17,9740657 | 2,23935E-05 | 0,000179984 | protein_coding |
| ENSMUSG00000024983 | Vti1a | vesicle transport through interaction with t-SNAREs homolog 1A (yeast) [Source:MGI Symbol;Acc:MGI:1855699] | 135,1346707 | -0,9424768 | **-1,92182481** | 7,07710414 | 34,480828 | 4,30471E-09 | 7,27628E-08 | protein_coding |
| ENSMUSG00000028159 | Dapp1 | dual adaptor for phosphotyrosine and 3-phosphoinositides 1 [Source:MGI Symbol;Acc:MGI:1347063] | 30,41663849 | -0,9424778 | **-1,92182604** | 4,91947975 | 38,7566846 | 4,80063E-10 | 9,62492E-09 | protein_coding |
| ENSMUSG00000093809 | Bcl2a1a | B cell leukemia/lymphoma 2 related protein A1a [Source:MGI Symbol;Acc:MGI:102687] | 55,8792289 | -0,9432324 | **-1,92283154** | 5,80471204 | 28,2769658 | 1,0514E-07 | 1,3752E-06 | protein_coding |
| ENSMUSG00000038990 | Cables2 | CDK5 and Abl enzyme substrate 2 [Source:MGI Symbol;Acc:MGI:2182335] | 41,23308934 | -0,9432635 | **-1,92287303** | 5,35802488 | 31,3681441 | 2,13455E-08 | 3,21264E-07 | protein_coding |
| ENSMUSG00000034751 | Mast4 | microtubule associated serine/threonine kinase family member 4 [Source:MGI Symbol;Acc:MGI:1918885] | 11,69393727 | -0,9445221 | **-1,9245513** | 3,52870075 | 21,1690752 | 4,20495E-06 | 4,0104E-05 | protein_coding |
| ENSMUSG00000079020 | Slc45a4 | solute carrier family 45, member 4 [Source:MGI Symbol;Acc:MGI:2146236] | 108,499364 | -0,9457902 | **-1,92624369** | 6,75686074 | 41,2262877 | 1,35587E-10 | 2,96751E-09 | protein_coding |
| ENSMUSG00000064367 | mt-Nd5 | mitochondrially encoded NADH dehydrogenase 5 [Source:MGI Symbol;Acc:MGI:102496] | 828,5598867 | -0,9473799 | **-1,92836732** | 9,69455661 | 26,238457 | 3,01753E-07 | 3,6115E-06 | protein_coding |
| ENSMUSG00000032035 | Ets1 | E26 avian leukemia oncogene 1, 5' domain [Source:MGI Symbol;Acc:MGI:95455] | 29,61977199 | -0,9488945 | **-1,93039285** | 4,88946548 | 32,6282894 | 1,11579E-08 | 1,76137E-07 | protein_coding |
| ENSMUSG00000026430 | Rassf5 | Ras association (RalGDS/AF-6) domain family member 5 [Source:MGI Symbol;Acc:MGI:1926375] | 48,73419398 | -0,9510692 | **-1,93330495** | 5,5969459 | 44,2186132 | 2,93676E-11 | 7,09146E-10 | protein_coding |
| ENSMUSG00000041112 | Elmo1 | engulfment and cell motility 1 [Source:MGI Symbol;Acc:MGI:2153044] | 390,7234713 | -0,9516577 | **-1,93409373** | 8,60951879 | 30,1225808 | 4,05581E-08 | 5,78717E-07 | protein_coding |
| ENSMUSG00000037270 | 4932438A13Rik | RIKEN cDNA 4932438A13 gene [Source:MGI Symbol;Acc:MGI:2444631] | 269,3646856 | -0,9517821 | **-1,93426054** | 8,07266889 | 42,7542279 | 6,20676E-11 | 1,43566E-09 | protein_coding |
| ENSMUSG00000021069 | Pygl | liver glycogen phosphorylase [Source:MGI Symbol;Acc:MGI:97829] | 177,5586558 | -0,9531813 | **-1,9361374** | 7,47093588 | 42,5363507 | 6,93807E-11 | 1,5881E-09 | protein_coding |
| ENSMUSG00000028538 | St3gal3 | ST3 beta-galactoside alpha-2,3-sialyltransferase 3 [Source:MGI Symbol;Acc:MGI:1316659] | 17,82962254 | -0,9541597 | **-1,93745082** | 4,14520809 | 32,2807733 | 1,33427E-08 | 2,08233E-07 | protein_coding |
| ENSMUSG00000026672 | Optn | optineurin [Source:MGI Symbol;Acc:MGI:1918898] | 62,73597762 | -0,954841 | **-1,93836601** | 5,96665703 | 30,6909598 | 3,02571E-08 | 4,45624E-07 | protein_coding |
| ENSMUSG00000001741 | Il16 | interleukin 16 [Source:MGI Symbol;Acc:MGI:1270855] | 55,91139773 | -0,9554956 | **-1,93924569** | 5,80020148 | 16,6226615 | 4,56029E-05 | 0,000338083 | protein_coding |
| ENSMUSG00000032946 | Rasgrp2 | RAS, guanyl releasing protein 2 [Source:MGI Symbol;Acc:MGI:1333849] | 17,00967148 | -0,955525 | **-1,93928516** | 4,07164955 | 26,0778938 | 3,27917E-07 | 3,88235E-06 | protein_coding |
| ENSMUSG00000035824 | Tk2 | thymidine kinase 2, mitochondrial [Source:MGI Symbol;Acc:MGI:1913266] | 46,17283936 | -0,956512 | **-1,9406124** | 5,51997271 | 35,0607292 | 3,19581E-09 | 5,50351E-08 | protein_coding |
| ENSMUSG00000029449 | Rhof | ras homolog gene family, member f [Source:MGI Symbol;Acc:MGI:1345629] | 16,3027118 | -0,9572008 | **-1,9415391** | 4,01792112 | 23,3404721 | 1,35714E-06 | 1,43099E-05 | protein_coding |
| ENSMUSG00000001348 | Acp5 | acid phosphatase 5, tartrate resistant [Source:MGI Symbol;Acc:MGI:87883] | 90,63496597 | -0,9574309 | **-1,94184886** | 6,49888971 | 31,7140948 | 1,78621E-08 | 2,7295E-07 | protein_coding |
| ENSMUSG00000036504 | Phpt1 | phosphohistidine phosphatase 1 [Source:MGI Symbol;Acc:MGI:1922704] | 9,344146186 | -0,957797 | **-1,9423417** | 3,20512528 | 17,9579132 | 2,25844E-05 | 0,000181385 | protein_coding |
| ENSMUSG00000049313 | Sorl1 | sortilin-related receptor, LDLR class A repeats-containing [Source:MGI Symbol;Acc:MGI:1202296] | 29,40186785 | -0,95868 | **-1,94353083** | 4,88487546 | 41,8581974 | 9,81385E-11 | 2,17828E-09 | protein_coding |
| ENSMUSG00000032609 | Klhdc8b | kelch domain containing 8B [Source:MGI Symbol;Acc:MGI:1925517] | 3,325299452 | -0,9589014 | **-1,94382917** | 1,69556565 | 7,91680807 | 0,004897761 | 0,019837671 | protein_coding |
| ENSMUSG00000063314 | Gm12657 | predicted gene 12657 [Source:MGI Symbol;Acc:MGI:3651714] | 4,136516918 | -0,9594725 | **-1,9445987** | 2,06355171 | 9,44651716 | 0,002115504 | 0,009840409 | pseudogene |
| ENSMUSG00000036469 | 41334 | membrane-associated ring finger (C3HC4) 1 [Source:MGI Symbol;Acc:MGI:1920175] | 114,8091942 | -0,9600425 | **-1,94536725** | 6,84175315 | 31,6045966 | 1,88982E-08 | 2,87184E-07 | protein_coding |
| ENSMUSG00000037410 | Tbc1d2b | TBC1 domain family, member 2B [Source:MGI Symbol;Acc:MGI:1914266] | 331,7312195 | -0,9610011 | **-1,94666019** | 8,37332422 | 34,9816244 | 3,32832E-09 | 5,70487E-08 | protein_coding |
| ENSMUSG00000074743 | Thbd | thrombomodulin [Source:MGI Symbol;Acc:MGI:98736] | 56,51908569 | -0,9616041 | **-1,94747406** | 5,82668274 | 38,9444141 | 4,36046E-10 | 8,87193E-09 | protein_coding |
| ENSMUSG00000031320 | Rps4x | ribosomal protein S4, X-linked [Source:MGI Symbol;Acc:MGI:98158] | 1033,46907 | -0,963579 | **-1,9501418** | 10,0130757 | 22,5031758 | 2,09796E-06 | 2,12446E-05 | protein_coding |
| ENSMUSG00000041688 | Amot | angiomotin [Source:MGI Symbol;Acc:MGI:108440] | 4,592142499 | -0,9639404 | **-1,9506304** | 2,20320546 | 10,3362943 | 0,001304396 | 0,006449776 | protein_coding |
| ENSMUSG00000008318 | Relt | RELT tumor necrosis factor receptor [Source:MGI Symbol;Acc:MGI:2443373] | 11,67120732 | -0,9643621 | **-1,95120062** | 3,52959593 | 12,7758306 | 0,000351127 | 0,002055316 | protein_coding |
| ENSMUSG00000034255 | Arhgap27 | Rho GTPase activating protein 27 [Source:MGI Symbol;Acc:MGI:1916903] | 232,1415735 | -0,9644042 | **-1,95125749** | 7,85731035 | 33,7633701 | 6,22398E-09 | 1,0176E-07 | protein_coding |
| ENSMUSG00000053291 | Rab4b | RAB4B, member RAS oncogene family [Source:MGI Symbol;Acc:MGI:105071] | 103,9534362 | -0,9666301 | **-1,95427046** | 6,69468893 | 41,2115497 | 1,36613E-10 | 2,98403E-09 | protein_coding |
| ENSMUSG00000028525 | Pde4b | phosphodiesterase 4B, cAMP specific [Source:MGI Symbol;Acc:MGI:99557] | 88,14311179 | -0,9673791 | **-1,95528531** | 6,46192897 | 25,232451 | 5,08199E-07 | 5,80414E-06 | protein_coding |
| ENSMUSG00000018654 | Ikzf1 | IKAROS family zinc finger 1 [Source:MGI Symbol;Acc:MGI:1342540] | 201,8358568 | -0,9673821 | **-1,95528928** | 7,65617019 | 35,021173 | 3,2614E-09 | 5,60766E-08 | protein_coding |
| ENSMUSG00000042992 | Loh12cr1 | loss of heterozygosity, 12, chromosomal region 1 homolog (human) [Source:MGI Symbol;Acc:MGI:1915024] | 17,04724076 | -0,9675308 | **-1,95549091** | 4,07949933 | 20,5053477 | 5,94649E-06 | 5,45359E-05 | protein_coding |
| ENSMUSG00000037151 | Lrrc20 | leucine rich repeat containing 20 [Source:MGI Symbol;Acc:MGI:2387182] | 43,31512576 | -0,9702839 | **-1,95922606** | 5,43143201 | 38,5011208 | 5,47223E-10 | 1,07748E-08 | protein_coding |
| ENSMUSG00000039158 | Akna | AT-hook transcription factor [Source:MGI Symbol;Acc:MGI:2140340] | 173,6689861 | -0,9714847 | **-1,96085744** | 7,4380097 | 31,9516943 | 1,58054E-08 | 2,42873E-07 | protein_coding |
| ENSMUSG00000064370 | mt-Cytb | mitochondrially encoded cytochrome b [Source:MGI Symbol;Acc:MGI:102501] | 1133,619556 | -0,9727436 | **-1,96256926** | 10,1467747 | 23,6555181 | 1,15215E-06 | 1,23499E-05 | protein_coding |

| | | | | | | | | | | |
|---|---|---|---|---|---|---|---|---|---|---|
| ENSMUSG00000090523 | Gypc | glycophorin C [Source:MGI Symbol;Acc:MGI:1098566] | 28,99845127 | -0,9771174 | **-1,96852825** | 4,84852377 | 33,8020796 | 6,10136E-09 | 1,00203E-07 | protein_coding |
| ENSMUSG00000020787 | P2rx1 | purinergic receptor P2X, ligand-gated ion channel, 1 [Source:MGI Symbol;Acc:MGI:1098235] | 3,174027542 | -0,9772752 | **-1,96874356** | 1,63936703 | 7,46100725 | 0,006304972 | 0,024652215 | protein_coding |
| ENSMUSG00000053329 | D10Jhu81e | DNA segment, Chr 10, Johns Hopkins University 81 expressed [Source:MGI Symbol;Acc:MGI:1351861] | 35,88586385 | -0,9789116 | **-1,97097784** | 5,15500973 | 34,4095315 | 4,46531E-09 | 7,53616E-08 | protein_coding |
| ENSMUSG00000053137 | Mapk11 | mitogen-activated protein kinase 11 [Source:MGI Symbol;Acc:MGI:1338024] | 6,654767446 | -0,9792311 | **-1,97141448** | 2,72994137 | 13,8664996 | 0,000196266 | 0,001233623 | protein_coding |
| ENSMUSG00000029178 | Klf3 | Kruppel-like factor 3 (basic) [Source:MGI Symbol;Acc:MGI:1342773] | 79,21394245 | -0,9793655 | **-1,97159805** | 6,30349843 | 34,3693252 | 4,55851E-09 | 7,66989E-08 | protein_coding |
| ENSMUSG00000028381 | Ugcg | UDP-glucose ceramide glucosyltransferase [Source:MGI Symbol;Acc:MGI:1332243] | 76,4816423 | -0,9800309 | **-1,97250765** | 6,25377546 | 33,637349 | 6,64054E-09 | 1,08249E-07 | protein_coding |
| ENSMUSG00000000244 | Tspan32 | tetraspanin 32 [Source:MGI Symbol;Acc:MGI:1350360] | 23,94217135 | -0,9824639 | **-1,97583692** | 4,57141477 | 31,8981876 | 1,62469E-08 | 2,48613E-07 | protein_coding |
| ENSMUSG00000025876 | Unc5a | unc-5 homolog A (C. elegans) [Source:MGI Symbol;Acc:MGI:894682] | 2,338338647 | -0,983827 | **-1,97770471** | 1,22977469 | 6,23880216 | 0,012498098 | 0,043468376 | protein_coding |
| ENSMUSG00000027763 | Mbnl1 | muscleblind-like 1 (Drosophila) [Source:MGI Symbol;Acc:MGI:1928482] | 676,7000579 | -0,9848846 | **-1,979155** | 9,40202025 | 28,2945725 | 1,04188E-07 | 1,36566E-06 | protein_coding |
| ENSMUSG00000038648 | Creb3l2 | cAMP responsive element binding protein 3-like 2 [Source:MGI Symbol;Acc:MGI:2442695] | 16,46767814 | -0,985986 | **-1,98066648** | 4,03330605 | 22,0086843 | 2,7142E-06 | 2,69385E-05 | protein_coding |
| ENSMUSG00000089722 | Gm11711 | predicted gene 11711 [Source:MGI Symbol;Acc:MGI:3702661] | 93,57778681 | -0,9872332 | **-1,98237957** | 6,55010376 | 34,5016391 | 4,25893E-09 | 7,20999E-08 | protein_coding |
| ENSMUSG00000040592 | Cd79b | CD79B antigen [Source:MGI Symbol;Acc:MGI:96431] | 30,30819589 | -0,9900176 | **-1,98620925** | 4,91857244 | 36,1603164 | 1,81734E-09 | 3,2949E-08 | protein_coding |
| ENSMUSG00000040699 | Limd2 | LIM domain containing 2 [Source:MGI Symbol;Acc:MGI:1915053] | 376,923227 | -0,9902443 | **-1,98652136** | 8,55744874 | 31,6081244 | 1,88639E-08 | 2,8706E-07 | protein_coding |
| ENSMUSG00000022540 | Rogdi | rogdi homolog (Drosophila) [Source:MGI Symbol;Acc:MGI:1913299] | 20,71757649 | -0,9908509 | **-1,98735683** | 4,3630904 | 29,9978186 | 4,32533E-08 | 6,13984E-07 | protein_coding |
| ENSMUSG00000032883 | Acsl3 | acyl-CoA synthetase long-chain family member 3 [Source:MGI Symbol;Acc:MGI:1921455] | 36,53792819 | -0,9911239 | **-1,98773282** | 5,18280307 | 22,7541633 | 1,84105E-06 | 1,89575E-05 | protein_coding |
| ENSMUSG00000060090 | Rp2h | retinitis pigmentosa 2 homolog (human) [Source:MGI Symbol;Acc:MGI:1277953] | 122,3634578 | -0,9915543 | **-1,98832593** | 6,93160028 | 44,8869567 | 2,08744E-11 | 5,1308E-10 | protein_coding |
| ENSMUSG00000044026 | Tmem20 | transmembrane protein 20 [Source:MGI Symbol;Acc:MGI:2444789] | 174,1233048 | -0,9931037 | **-1,9904625** | 7,44456114 | 40,2213216 | 2,2676E-10 | 4,81897E-09 | protein_coding |
| ENSMUSG00000024948 | Map4k2 | mitogen-activated protein kinase kinase kinase kinase 2 [Source:MGI Symbol;Acc:MGI:1346883] | 100,635299 | -0,9932511 | **-1,99066585** | 6,64815313 | 44,0633912 | 3,17911E-11 | 7,60979E-10 | protein_coding |
| ENSMUSG00000034066 | Farp2 | FERM, RhoGEF and pleckstrin domain protein 2 [Source:MGI Symbol;Acc:MGI:2385126] | 16,93106465 | -0,994372 | **-1,99221315** | 4,07309242 | 18,7988994 | 1,45251E-05 | 0,000121915 | protein_coding |
| ENSMUSG00000028464 | Tpm2 | tropomyosin 2, beta [Source:MGI Symbol;Acc:MGI:98810] | 6,961681637 | -0,9983237 | **-1,99767746** | 2,8413736 | 14,1188013 | 0,00017162 | 0,001094363 | protein_coding |
| ENSMUSG00000031367 | Ap1s2 | adaptor-related protein complex 1, sigma 2 subunit [Source:MGI Symbol;Acc:MGI:1889383] | 299,6188544 | -0,998476 | **-1,99788842** | 8,22597397 | 42,4143191 | 7,38474E-11 | 1,67637E-09 | protein_coding |
| ENSMUSG00000059994 | Fcrl1 | Fc receptor-like 1 [Source:MGI Symbol;Acc:MGI:2442862] | 24,59919118 | -0,9985323 | **-1,99796637** | 4,62598462 | 30,7488096 | 2,93684E-08 | 4,33697E-07 | protein_coding |
| ENSMUSG00000026489 | Adck3 | aarF domain containing kinase 3 [Source:MGI Symbol;Acc:MGI:1914676] | 14,69876844 | -0,9991549 | **-1,99882875** | 3,86220349 | 25,8228233 | 3,74237E-07 | 4,36954E-06 | protein_coding |
| ENSMUSG00000053080 | 2700081O15Rik | RIKEN cDNA 2700081O15 gene [Source:MGI Symbol;Acc:MGI:1919667] | 6,553173247 | -0,9993143 | **-1,9990496** | 2,67535809 | 12,8813346 | 0,000331875 | 0,001956178 | protein_coding |
| ENSMUSG00000040139 | 9430038I01Rik | RIKEN cDNA 9430038I01 gene [Source:MGI Symbol;Acc:MGI:1924502] | 10,88960215 | -1,0006552 | **-2,00090849** | 3,42686048 | 20,9761059 | 4,65047E-06 | 4,38958E-05 | protein_coding |
| ENSMUSG00000013033 | Lphn1 | latrophilin 1 [Source:MGI Symbol;Acc:MGI:1929461] | 5,126158947 | -1,0010659 | **-2,00147821** | 2,33891464 | 11,2822396 | 0,000782521 | 0,004155417 | protein_coding |
| ENSMUSG00000095597 | | MCG116065; Uncharacterized protein  [Source:UniProtKB/TrEMBL;Acc:J3QJZ3] | 93,10507025 | -1,0019494 | **-2,00270426** | 6,54321556 | 38,0819269 | 6,78356E-10 | 1,31216E-08 | protein_coding |
| ENSMUSG00000030774 | Pak1 | p21 protein (Cdc42/Rac)-activated kinase 1 [Source:MGI Symbol;Acc:MGI:1339975] | 121,4975106 | -1,0020381 | **-2,00282746** | 6,92199154 | 44,0683819 | 3,17102E-11 | 7,60698E-10 | protein_coding |
| ENSMUSG00000000248 | Clec2g | C-type lectin domain family 2, member g [Source:MGI Symbol;Acc:MGI:1918059] | 2,366930372 | -1,0036232 | **-2,0050291** | 1,20662931 | 6,55646941 | 0,010450351 | 0,037694682 | protein_coding |
| ENSMUSG00000097771 | | Coronin  [Source:UniProtKB/TrEMBL;Acc:A2RS22] | 359,0191888 | -1,0042118 | **-2,00584731** | 8,48728246 | 35,44727 | 2,62049E-09 | 4,59192E-08 | protein_coding |
| ENSMUSG00000024835 | Coro1b | coronin, actin binding protein 1B [Source:MGI Symbol;Acc:MGI:1345963] | 359,0191888 | -1,0042155 | **-2,00585245** | 8,48728416 | 35,352066 | 2,75177E-09 | 4,79899E-08 | protein_coding |
| ENSMUSG00000058558 | Rpl5 | ribosomal protein L5 [Source:MGI Symbol;Acc:MGI:102854] | 32,50332217 | -1,0045371 | **-2,00629964** | 5,01787183 | 28,3389373 | 1,01827E-07 | 1,33825E-06 | protein_coding |
| ENSMUSG00000000127 | Fer | fer (fms/fps related) protein kinase [Source:MGI Symbol;Acc:MGI:105917] | 64,2947048 | -1,0058307 | **-2,00809938** | 6,00070168 | 49,0462445 | 2,49998E-12 | 7,0975E-11 | protein_coding |
| ENSMUSG00000044667 | D3Bwg0562e | DNA segment, Chr 3, Brigham & Women's Genetics 0562 expressed [Source:MGI Symbol;Acc:MGI:106530] | 3,509071076 | -1,0091557 | **-2,01273291** | 1,84647373 | 7,55631212 | 0,005980024 | 0,023591571 | protein_coding |
| ENSMUSG00000039735 | Fnbp1l | formin binding protein 1-like [Source:MGI Symbol;Acc:MGI:1925642] | 526,5791793 | -1,0106445 | **-2,01481091** | 9,04017448 | 28,6078701 | 8,86213E-08 | 1,17453E-06 | protein_coding |
| ENSMUSG00000067577 | A430093F15Rik | RIKEN cDNA A430093F15 gene [Source:MGI Symbol;Acc:MGI:2685520] | 4,641010353 | -1,0124639 | **-2,01735346** | 2,19679419 | 10,3745113 | 0,00127767 | 0,006337588 | protein_coding |
| ENSMUSG00000042228 | Lyn | Yamaguchi sarcoma viral (v-yes-1) oncogene homolog [Source:MGI Symbol;Acc:MGI:96892] | 575,6829359 | -1,012547 | **-2,01746965** | 9,16907745 | 28,6843293 | 8,51903E-08 | 1,13178E-06 | protein_coding |
| ENSMUSG00000089929 | Bcl2a1b | B cell leukemia/lymphoma 2 related protein A1b [Source:MGI Symbol;Acc:MGI:1278326] | 220,9638244 | -1,0129457 | **-2,01802726** | 7,78742328 | 26,8015241 | 2,25459E-07 | 2,73715E-06 | protein_coding |
| ENSMUSG00000004113 | Cacna1b | calcium channel, voltage-dependent, N type, alpha 1B subunit [Source:MGI Symbol;Acc:MGI:88296] | 3,703842926 | -1,0137454 | **-2,01914629** | 1,86682915 | 7,40969753 | 0,006487323 | 0,025257341 | protein_coding |
| ENSMUSG00000025010 | Ccnj | cyclin J [Source:MGI Symbol;Acc:MGI:2443297] | 15,52743256 | -1,0150259 | **-2,02093915** | 3,93703809 | 27,637347 | 1,46326E-07 | 1,84728E-06 | protein_coding |
| ENSMUSG00000073758 | Sh3d21 | SH3 domain containing 21 [Source:MGI Symbol;Acc:MGI:1914188] | 6,82943889 | -1,0159214 | **-2,02219405** | 2,76833064 | 13,4024552 | 0,000251295 | 0,001533878 | protein_coding |
| ENSMUSG00000009248 | Ascl2 | achaete-scute complex homolog 2 (Drosophila) [Source:MGI Symbol;Acc:MGI:96920] | 10,63006858 | -1,0212099 | **-2,02962041** | 3,39688507 | 18,5413201 | 1,66261E-05 | 0,000138073 | protein_coding |
| ENSMUSG00000097227 | | | 3,500932215 | -1,0246125 | **-2,03441284** | 1,75897852 | 9,55668362 | 0,001992227 | 0,009338139 | lincRNA |
| ENSMUSG00000090862 | Rps13 | ribosomal protein S13 [Source:MGI Symbol;Acc:MGI:1915302] | 84,21891032 | -1,0246946 | **-2,03452866** | 6,38964909 | 43,2013608 | 4,93863E-11 | 1,1619E-09 | protein_coding |
| ENSMUSG00000032258 | Lca5 | Leber congenital amaurosis 5 (human) [Source:MGI Symbol;Acc:MGI:1923032] | 18,57214188 | -1,0253253 | **-2,03541834** | 4,20351839 | 34,5924632 | 4,06477E-09 | 6,90257E-08 | protein_coding |
| ENSMUSG00000064043 | Trerf1 | transcriptional regulating factor 1 [Source:MGI Symbol;Acc:MGI:2442086] | 110,1104755 | -1,0261031 | **-2,03651597** | 6,77812417 | 42,9764394 | 5,54031E-11 | 1,29389E-09 | protein_coding |
| ENSMUSG00000021423 | Ly86 | lymphocyte antigen 86 [Source:MGI Symbol;Acc:MGI:1321404] | 84,92052321 | -1,0275989 | **-2,03862849** | 6,40536335 | 38,3695351 | 5,85391E-10 | 1,14037E-08 | protein_coding |
| ENSMUSG00000054263 | Lifr | leukemia inhibitory factor receptor [Source:MGI Symbol;Acc:MGI:96788] | 10,7162955 | -1,0282233 | **-2,03951099** | 3,40415026 | 16,4046457 | 5,11597E-05 | 0,000376233 | protein_coding |
| ENSMUSG00000038127 | Ccdc50 | coiled-coil domain containing 50 [Source:MGI Symbol;Acc:MGI:1914751] | 760,0365573 | -1,0348875 | **-2,04895388** | 9,56975516 | 27,1112099 | 1,92081E-07 | 2,36326E-06 | protein_coding |
| ENSMUSG00000001424 | Snd1 | staphylococcal nuclease and tudor domain containing 1 [Source:MGI Symbol;Acc:MGI:1929266] | 275,3434469 | -1,0352316 | **-2,04944257** | 8,10398291 | 37,4014517 | 9,61505E-10 | 1,81825E-08 | protein_coding |
| ENSMUSG00000029552 | Tes | testis derived transcript [Source:MGI Symbol;Acc:MGI:105081] | 219,1096676 | -1,0363661 | **-2,05105487** | 7,77424404 | 34,3330817 | 4,6442E-09 | 7,7902E-08 | protein_coding |
| ENSMUSG00000008450 | Nutf2 | nuclear transport factor 2 [Source:MGI Symbol;Acc:MGI:1915301] | 13,17413601 | -1,0375747 | **-2,0527738** | 3,71428941 | 22,6933407 | 1,90025E-06 | 1,9494E-05 | protein_coding |
| ENSMUSG00000020492 | Ska2 | spindle and kinetochore associated complex subunit 2 [Source:MGI Symbol;Acc:MGI:1913390] | 5,492007889 | -1,0389018 | **-2,05466306** | 2,4349411 | 12,4640168 | 0,000414867 | 0,002377747 | protein_coding |
| ENSMUSG00000041351 | Rap1gap | Rap1 GTPase-activating protein [Source:MGI Symbol;Acc:MGI:109338] | 7,395063742 | -1,0404831 | **-2,05691629** | 2,88966435 | 14,4380622 | 0,000144845 | 0,000945581 | protein_coding |
| ENSMUSG00000024854 | Pold4 | polymerase (DNA-directed), delta 4 [Source:MGI Symbol;Acc:MGI:1916995] | 22,06125616 | -1,0491103 | **-2,06925334** | 4,44383941 | 26,1214617 | 3,20601E-07 | 3,82043E-06 | protein_coding |
| ENSMUSG00000097508 | | Polymerase (DNA-directed), delta 4, isoform CRA_b; Small subunit DNA polymerase delta p12  [Source:UniProtKB/TrEM | 22,06125616 | -1,0491133 | **-2,06925769** | 4,44384324 | 26,1164833 | 3,21428E-07 | 3,82615E-06 | protein_coding |
| ENSMUSG00000030465 | Psd3 | pleckstrin and Sec7 domain containing 3 [Source:MGI Symbol;Acc:MGI:1918215] | 57,09438052 | -1,0503851 | **-2,07108263** | 5,82975548 | 48,1652113 | 3,91778E-12 | 1,06811E-10 | protein_coding |
| ENSMUSG00000063873 | Slc24a3 | solute carrier family 24 (sodium/potassium/calcium exchanger), member 3 [Source:MGI Symbol;Acc:MGI:2137513] | 10,09164564 | -1,0505088 | **-2,07126017** | 3,31434477 | 23,5890876 | 1,19262E-06 | 1,26847E-05 | protein_coding |
| ENSMUSG00000051285 | Pcmtd1 | protein-L-isoaspartate (D-aspartate) O-methyltransferase domain containing 1 [Source:MGI Symbol;Acc:MGI:2441773] | 158,8717729 | -1,0508275 | **-2,07171778** | 7,30868171 | 58,3921562 | 2,14745E-14 | 8,10793E-13 | protein_coding |
| ENSMUSG00000006435 | Neurl1a | neuralized homolog 1A (Drosophila) [Source:MGI Symbol;Acc:MGI:1334263] | 9,783341537 | -1,0542302 | **-2,0766098** | 3,26913312 | 17,3298128 | 3,14218E-05 | 0,000242608 | protein_coding |
| ENSMUSG00000031381 | Piga | phosphatidylinositol glycan anchor biosynthesis, class A [Source:MGI Symbol;Acc:MGI:99461] | 23,42882041 | -1,0554828 | **-2,07841361** | 4,54613226 | 28,3093484 | 1,03395E-07 | 1,35724E-06 | protein_coding |
| ENSMUSG00000022769 | Sdf2l1 | stromal cell-derived factor 2-like 1 [Source:MGI Symbol;Acc:MGI:2149842] | 76,16775652 | -1,0585988 | **-2,08290754** | 6,24575199 | 42,9325383 | 5,66604E-11 | 1,31613E-09 | protein_coding |
| ENSMUSG00000067586 | S1pr3 | sphingosine-1-phosphate receptor 3 [Source:MGI Symbol;Acc:MGI:1339365] | 5,50156711 | -1,059648 | **-2,08442285** | 2,48710393 | 13,9353374 | 0,000189208 | 0,001195414 | protein_coding |
| ENSMUSG00000019853 | Hebp2 | heme binding protein 2 [Source:MGI Symbol;Acc:MGI:1860084] | 3,958155108 | -1,0637778 | **-2,09039827** | 1,96166207 | 11,7331987 | 0,00061395 | 0,003357624 | protein_coding |
| ENSMUSG00000078937 | Cpt1b | carnitine palmitoyltransferase 1b, muscle [Source:MGI Symbol;Acc:MGI:1098297] | 5,724000086 | -1,0657809 | **-2,09330264** | 2,48282913 | 12,552257 | 0,000395728 | 0,002285943 | protein_coding |
| ENSMUSG00000006517 | Mvd | mevalonate (diphospho) decarboxylase [Source:MGI Symbol;Acc:MGI:2179327] | 29,95633022 | -1,0668146 | **-2,09480308** | 4,89596477 | 13,7523055 | 0,000208565 | 0,001299407 | protein_coding |
| ENSMUSG00000031778 | Cx3cl1 | chemokine (C-X3-C motif) ligand 1 [Source:MGI Symbol;Acc:MGI:1097153] | 5,692416686 | -1,0673701 | **-2,0956098** | 2,47989172 | 14,1712053 | 0,000166905 | 0,001071138 | protein_coding |
| ENSMUSG00000000915 | Hip1r | huntingtin interacting protein 1 related [Source:MGI Symbol;Acc:MGI:1352504] | 11,72688425 | -1,067439 | **-2,09570988** | 3,5309359 | 16,8330473 | 4,08163E-05 | 0,000305201 | protein_coding |
| ENSMUSG00000032575 | Manf | mesencephalic astrocyte-derived neurotrophic factor [Source:MGI Symbol;Acc:MGI:1922090] | 116,2637575 | -1,0684913 | **-2,09723901** | 6,85938404 | 34,3885251 | 4,51377E-09 | 7,60625E-08 | protein_coding |
| ENSMUSG00000057440 | Mpp7 | membrane protein, palmitoylated 7 (MAGUK p55 subfamily member 7) [Source:MGI Symbol;Acc:MGI:1922989] | 13,54416887 | -1,0686274 | **-2,09743682** | 3,7476826 | 28,2433707 | 1,0698E-07 | 1,39595E-06 | protein_coding |
| ENSMUSG00000086825 | Gm15675 | predicted gene 15675 [Source:MGI Symbol;Acc:MGI:3783117] | 113,8896124 | -1,0705564 | **-2,1002432** | 6,8274481 | 44,4433332 | 2,61827E-11 | 6,39265E-10 | lincRNA |

| | | | | | | | | | | |
|---|---|---|---|---|---|---|---|---|---|---|
| ENSMUSG00000032216 | Nedd4 | neural precursor cell expressed, developmentally down-regulated 4 [Source:MGI Symbol;Acc:MGI:97297] | 11,83681239 | -1,070609 | **-2,10031975** | 3,58850792 | 18,0139887 | 2,19288E-05 | 0,000176765 | protein_coding |
| ENSMUSG00000022261 | Sdc2 | syndecan 2 [Source:MGI Symbol;Acc:MGI:1349165] | 4,265301781 | -1,0710442 | **-2,10095338** | 2,06789803 | 12,3907659 | 0,000431462 | 0,002462585 | protein_coding |
| ENSMUSG00000059895 | Ptp4a3 | protein tyrosine phosphatase 4a3 [Source:MGI Symbol;Acc:MGI:1277098] | 80,56139785 | -1,0749735 | **-2,10668334** | 6,3271101 | 50,9781442 | 9,34002E-13 | 2,766E-11 | protein_coding |
| ENSMUSG00000052539 | Magi3 | membrane associated guanylate kinase, WW and PDZ domain containing 3 [Source:MGI Symbol;Acc:MGI:1923484] | 12,16850534 | -1,0784451 | **-2,11175889** | 3,59785068 | 27,5609603 | 1,5222E-07 | 1,90267E-06 | protein_coding |
| ENSMUSG00000059824 | Dbp | D site albumin promoter binding protein [Source:MGI Symbol;Acc:MGI:94866] | 11,98950603 | -1,079698 | **-2,11359356** | 3,56180745 | 19,718952 | 8,97073E-06 | 7,88491E-05 | protein_coding |
| ENSMUSG00000074794 | Arrdc3 | arrestin domain containing 3 [Source:MGI Symbol;Acc:MGI:2145242] | 16,79657976 | -1,0812413 | **-2,11585586** | 4,04827639 | 32,8558109 | 9,92539E-09 | 1,58044E-07 | protein_coding |
| ENSMUSG00000036052 | Dnajb5 | DnaJ (Hsp40) homolog, subfamily B, member 5 [Source:MGI Symbol;Acc:MGI:1930018] | 21,66719958 | -1,0834055 | **-2,11903219** | 4,42369752 | 35,587922 | 2,43794E-09 | 4,31375E-08 | protein_coding |
| ENSMUSG00000058709 | Egln2 | EGL nine homolog 2 (C. elegans) [Source:MGI Symbol;Acc:MGI:1932287] | 165,8947232 | -1,0851608 | **-2,12161187** | 7,37125323 | 42,5791065 | 6,78806E-11 | 1,557E-09 | protein_coding |
| ENSMUSG00000024232 | Bambi | BMP and activin membrane-bound inhibitor, homolog (Xenopus laevis) [Source:MGI Symbol;Acc:MGI:1915260] | 16,36768585 | -1,0865097 | **-2,12359651** | 4,01286087 | 26,3754536 | 2,8109E-07 | 3,37523E-06 | protein_coding |
| ENSMUSG00000064360 | mt-Nd3 | mitochondrially encoded NADH dehydrogenase 3 [Source:MGI Symbol;Acc:MGI:102499] | 111,0259805 | -1,0865761 | **-2,12369434** | 6,79352398 | 53,5160763 | 2,56485E-13 | 8,3127E-12 | protein_coding |
| ENSMUSG00000068686 | Cd59b | CD59b antigen [Source:MGI Symbol;Acc:MGI:1888996] | 2,860396011 | -1,0918954 | **-2,13153894** | 1,49255928 | 8,50891836 | 0,003534101 | 0,015179501 | protein_coding |
| ENSMUSG00000024378 | Stard4 | StAR-related lipid transfer (START) domain containing 4 [Source:MGI Symbol;Acc:MGI:2156764] | 61,69797357 | -1,0940619 | **-2,13474227** | 5,94272371 | 19,4498515 | 1,03276E-05 | 8,92755E-05 | protein_coding |
| ENSMUSG00000097142 | | | 1652,119203 | -1,0968669 | **-2,13889688** | 10,6901164 | 22,2362866 | 2,41074E-06 | 2,41668E-05 | lincRNA |
| ENSMUSG00000015202 | Cnksr3 | Cnksr family member 3 [Source:MGI Symbol;Acc:MGI:2674130] | 134,3089055 | -1,0990274 | **-2,14210239** | 7,06866614 | 29,3597689 | 6,01127E-08 | 8,29722E-07 | protein_coding |
| ENSMUSG00000003283 | Hck | hemopoietic cell kinase [Source:MGI Symbol;Acc:MGI:96052] | 507,8651316 | -1,0994132 | **-2,14267527** | 8,9880917 | 29,6972516 | 5,05064E-08 | 7,08702E-07 | protein_coding |
| ENSMUSG00000038540 | Tmc3 | transmembrane channel-like gene family 3 [Source:MGI Symbol;Acc:MGI:2669033] | 39,5126993 | -1,1023759 | **-2,14707996** | 5,31016395 | 26,8968243 | 2,14611E-07 | 2,61412E-06 | protein_coding |
| ENSMUSG00000028555 | Ttc39a | tetratricopeptide repeat domain 39A [Source:MGI Symbol;Acc:MGI:2444350] | 2,904401203 | -1,1034163 | **-2,1486289** | 1,50886419 | 9,69920365 | 0,001843479 | 0,008756724 | protein_coding |
| ENSMUSG00000039096 | Rsad1 | radical S-adenosyl methionine domain containing 1 [Source:MGI Symbol;Acc:MGI:3039628] | 22,54713462 | -1,1061186 | **-2,15265721** | 4,48074391 | 37,2028689 | 1,06457E-09 | 2,00281E-08 | protein_coding |
| ENSMUSG00000079317 | Trappc2 | trafficking protein particle complex 2 [Source:MGI Symbol;Acc:MGI:1913476] | 77,49083362 | -1,1091927 | **-2,15724893** | 6,27499587 | 46,5967592 | 8,72044E-12 | 2,26505E-10 | protein_coding |
| ENSMUSG00000059277 | R74862 | expressed sequence R74862 [Source:MGI Symbol;Acc:MGI:2142382] | 2,059664723 | -1,1119106 | **-2,16131687** | 1,00575031 | 7,77670881 | 0,005292413 | 0,0212606 | lincRNA |
| ENSMUSG00000060512 | 0610040J01Rik | RIKEN cDNA 0610040J01 gene [Source:MGI Symbol;Acc:MGI:1923511] | 5,935489632 | -1,111929 | **-2,16134445** | 2,56105671 | 16,08888 | 6,04379E-05 | 0,00043715 | protein_coding |
| ENSMUSG00000035356 | Nfkbiz | nuclear factor of kappa light polypeptide gene enhancer in B cells inhibitor, zeta [Source:MGI Symbol;Acc:MGI:1931595] | 584,0145074 | -1,1143169 | **-2,16492476** | 9,18964746 | 31,3222872 | 2,18556E-08 | 3,27149E-07 | protein_coding |
| ENSMUSG00000018417 | Myo1b | myosin IB [Source:MGI Symbol;Acc:MGI:107752] | 83,29308017 | -1,1159866 | **-2,16743185** | 6,37695951 | 61,0972269 | 5,43246E-15 | 2,24385E-13 | protein_coding |
| ENSMUSG00000029810 | Tmem176b | transmembrane protein 176B [Source:MGI Symbol;Acc:MGI:1916348] | 1116,665097 | -1,1181625 | **-2,17070329** | 10,1248794 | 23,1019989 | 1,53631E-06 | 1,59995E-05 | protein_coding |
| ENSMUSG00000032890 | Rims3 | regulating synaptic membrane exocytosis 3 [Source:MGI Symbol;Acc:MGI:2443331] | 103,9648856 | -1,1220284 | **-2,17652773** | 6,69551827 | 55,9358351 | 7,48753E-14 | 2,6116E-12 | protein_coding |
| ENSMUSG00000030826 | Bcat2 | branched chain aminotransferase 2, mitochondrial [Source:MGI Symbol;Acc:MGI:1276534] | 162,6890929 | -1,1240426 | **-2,17956864** | 7,34448086 | 52,6421476 | 4,00208E-13 | 1,24563E-11 | protein_coding |
| ENSMUSG00000039157 | Fam102a | family with sequence similarity 102, member A [Source:MGI Symbol;Acc:MGI:2138935] | 47,79698897 | -1,1248388 | **-2,18077183** | 5,57215912 | 61,5060247 | 4,41391E-15 | 1,85807E-13 | protein_coding |
| ENSMUSG00000004880 | Lbr | lamin B receptor [Source:MGI Symbol;Acc:MGI:2138281] | 16,02893396 | -1,1248721 | **-2,18082208** | 3,9920147 | 30,4440093 | 3,43643E-08 | 5,02075E-07 | protein_coding |
| ENSMUSG00000058258 | Idi1 | isopentenyl-diphosphate delta isomerase [Source:MGI Symbol;Acc:MGI:2442264] | 44,53069896 | -1,1267894 | **-2,18372227** | 5,47200032 | 13,8712294 | 0,000195773 | 0,001231227 | protein_coding |
| ENSMUSG00000033105 | Lss | lanosterol synthase [Source:MGI Symbol;Acc:MGI:1336155] | 51,59701635 | -1,1296884 | **-2,1881147** | 5,68316674 | 20,0298508 | 7,62426E-06 | 6,79382E-05 | protein_coding |
| ENSMUSG00000030878 | Cdr2 | cerebellar degeneration-related 2 [Source:MGI Symbol;Acc:MGI:1100885] | 27,14108376 | -1,1354182 | **-2,19682232** | 4,75740761 | 29,1486746 | 6,70316E-08 | 9,16015E-07 | protein_coding |
| ENSMUSG00000029406 | Pitpnm2 | phosphatidylinositol transfer protein, membrane-associated 2 [Source:MGI Symbol;Acc:MGI:1336192] | 7,591417961 | -1,1374964 | **-2,19998915** | 2,90702316 | 20,1387169 | 7,20239E-06 | 6,47037E-05 | protein_coding |
| ENSMUSG00000031389 | Arhgap4 | Rho GTPase activating protein 4 [Source:MGI Symbol;Acc:MGI:2159577] | 413,8442257 | -1,1375522 | **-2,20007419** | 8,6922732 | 36,966657 | 1,20167E-09 | 2,22643E-08 | protein_coding |
| ENSMUSG00000033174 | Mgll | monoglyceride lipase [Source:MGI Symbol;Acc:MGI:1346042] | 2,787128417 | -1,1379089 | **-2,20061824** | 1,45862978 | 9,66397211 | 0,001879169 | 0,008893666 | protein_coding |
| ENSMUSG00000020589 | Fam49a | family with sequence similarity 49, member A [Source:MGI Symbol;Acc:MGI:1261783] | 290,5542119 | -1,1406696 | **-2,2048333** | 8,18246211 | 49,9762425 | 1,55619E-12 | 4,5113E-11 | protein_coding |
| ENSMUSG00000048647 | Exd1 | exonuclease 3'-5' domain containing 1 [Source:MGI Symbol;Acc:MGI:3045306] | 3,862512311 | -1,141074 | **-2,20545138** | 1,93283107 | 10,2045006 | 0,001400983 | 0,006864 | protein_coding |
| ENSMUSG00000030472 | Ceacam18 | carcinoembryonic antigen-related cell adhesion molecule 18 [Source:MGI Symbol;Acc:MGI:1919681] | 2,324970898 | -1,1452198 | **-2,21179824** | 1,20676027 | 7,75791007 | 0,005347781 | 0,021451648 | protein_coding |
| ENSMUSG00000095332 | Gm9821 | predicted gene 9821 [Source:MGI Symbol;Acc:MGI:3704208] | 9,723810642 | -1,1457707 | **-2,21264299** | 3,23764528 | 27,0918665 | 1,94013E-07 | 2,38436E-06 | protein_coding |
| ENSMUSG00000079419 | Ms4a6c | membrane-spanning 4-domains, subfamily A, member 6C [Source:MGI Symbol;Acc:MGI:2385644] | 526,1284061 | -1,1484766 | **-2,21679689** | 9,03907496 | 31,5539521 | 1,93975E-08 | 2,94365E-07 | protein_coding |
| ENSMUSG00000026357 | Rgs18 | regulator of G-protein signaling 18 [Source:MGI Symbol;Acc:MGI:1927498] | 28,6357541 | -1,1557783 | **-2,22804495** | 4,82705385 | 48,7989922 | 2,83585E-12 | 7,98911E-11 | protein_coding |
| ENSMUSG00000071637 | Cebpd | CCAAT/enhancer binding protein (C/EBP), delta [Source:MGI Symbol;Acc:MGI:103573] | 67,57197655 | -1,1621295 | **-2,23787502** | 6,07294104 | 33,3375337 | 7,74729E-09 | 1,24992E-07 | ocessed_pseudoge |
| ENSMUSG00000023832 | Acat2 | acetyl-Coenzyme A acetyltransferase 2 [Source:MGI Symbol;Acc:MGI:87871] | 31,40093337 | -1,162759 | **-2,23885181** | 4,96551203 | 14,800144 | 0,000119526 | 0,000798806 | protein_coding |
| ENSMUSG00000052889 | Prkcb | protein kinase C, beta [Source:MGI Symbol;Acc:MGI:97596] | 623,3423681 | -1,1681111 | **-2,24717293** | 9,28363993 | 37,6016289 | 8,6772E-10 | 1,65495E-08 | protein_coding |
| ENSMUSG00000069607 | Cd300lh | CD300 antigen like family member H [Source:MGI Symbol;Acc:MGI:2687214] | 361,9324513 | -1,1702875 | **-2,25056543** | 8,49967761 | 48,2903687 | 3,67553E-12 | 1,00706E-10 | protein_coding |
| ENSMUSG00000087691 | Gm15674 | predicted gene 15674 [Source:MGI Symbol;Acc:MGI:3783116] | 33,46134998 | -1,1706208 | **-2,25108541** | 5,05014107 | 48,3059963 | 3,64635E-12 | 1,00156E-10 | antisense |
| ENSMUSG00000034641 | Cd300ld | CD300 molecule-like family member d [Source:MGI Symbol;Acc:MGI:2442358] | 303,7920537 | -1,1737411 | **-2,2559594** | 8,24688421 | 42,8243375 | 5,98825E-11 | 1,38804E-09 | protein_coding |
| ENSMUSG00000030471 | Zdhhc13 | zinc finger, DHHC domain containing 13 [Source:MGI Symbol;Acc:MGI:1919227] | 72,83732128 | -1,1742047 | **-2,25668437** | 6,18380897 | 43,2493671 | 4,81894E-11 | 1,13617E-09 | protein_coding |
| ENSMUSG00000021087 | Rtn1 | reticulon 1 [Source:MGI Symbol;Acc:MGI:1933947] | 7,69955365 | -1,1748902 | **-2,2577569** | 2,9149488 | 17,8941264 | 2,33541E-05 | 0,000186748 | protein_coding |
| ENSMUSG00000042826 | Fgf11 | fibroblast growth factor 11 [Source:MGI Symbol;Acc:MGI:109167] | 76,60508414 | -1,1768407 | **-2,26081155** | 6,25615682 | 51,3899494 | 7,57252E-13 | 2,27321E-11 | protein_coding |
| ENSMUSG00000031486 | Gpr124 | G protein-coupled receptor 124 [Source:MGI Symbol;Acc:MGI:1925810] | 2,559267151 | -1,1769588 | **-2,26099656** | 1,37009936 | 8,719296 | 0,003148596 | 0,013765867 | protein_coding |
| ENSMUSG00000079038 | D130040H23Rik | RIKEN cDNA D130040H23 gene [Source:MGI Symbol;Acc:MGI:2444324] | 4,996327146 | -1,1775587 | **-2,261937** | 2,30061851 | 14,4452079 | 0,000144297 | 0,00094256 | protein_coding |
| ENSMUSG00000031385 | Plxnb3 | plexin B3 [Source:MGI Symbol;Acc:MGI:2154240] | 13,70958275 | -1,1791336 | **-2,26440753** | 3,76930623 | 37,039877 | 1,15738E-09 | 2,14799E-08 | protein_coding |
| ENSMUSG00000028709 | Mob3c | MOB kinase activator 3C [Source:MGI Symbol;Acc:MGI:2140623] | 38,89760163 | -1,1808183 | **-2,26705321** | 5,27423261 | 46,1125329 | 1,11652E-11 | 2,85284E-10 | protein_coding |
| ENSMUSG00000034266 | Batf | basic leucine zipper transcription factor, ATF-like [Source:MGI Symbol;Acc:MGI:1859147] | 58,72098512 | -1,1810881 | **-2,26747736** | 5,87388392 | 34,1513364 | 5,09882E-09 | 8,48799E-08 | protein_coding |
| ENSMUSG00000038332 | Sesn1 | sestrin 1 [Source:MGI Symbol;Acc:MGI:2155278] | 208,4350396 | -1,1825039 | **-2,26970357** | 7,70132118 | 46,9068336 | 7,44427E-12 | 1,95203E-10 | protein_coding |
| ENSMUSG00000044350 | Lacc1 | laccase (multicopper oxidoreductase) domain containing 1 [Source:MGI Symbol;Acc:MGI:2445077] | 236,7518668 | -1,1872794 | **-2,27722904** | 7,88640678 | 35,7660069 | 2,22496E-09 | 3,98788E-08 | protein_coding |
| ENSMUSG00000096255 | Dynlt1b | dynein light chain Tctex-type 1B [Source:MGI Symbol;Acc:MGI:98643] | 6,197472585 | -1,1934922 | **-2,28705678** | 2,66671041 | 14,5094242 | 0,00013946 | 0,00091314 | protein_coding |
| ENSMUSG00000078616 | Trim30c | tripartite motif-containing 30C [Source:MGI Symbol;Acc:MGI:4821257] | 4,826622278 | -1,1963456 | **-2,2915847** | 2,28571881 | 15,1297633 | 0,000100368 | 0,000682816 | protein_coding |
| ENSMUSG00000081855 | Rpl17-ps5 | ribosomal protein L17, pseudogene 5 [Source:MGI Symbol;Acc:MGI:3704246] | 2,326337071 | -1,1977375 | **-2,29379669** | 1,15907518 | 8,21043379 | 0,004165019 | 0,017386424 | pseudogene |
| ENSMUSG00000028044 | Cks1b | CDC28 protein kinase 1b [Source:MGI Symbol;Acc:MGI:1889208] | 18,78420407 | -1,1977673 | **-2,29384407** | 4,22317861 | 37,8187326 | 7,76327E-10 | 1,49117E-08 | protein_coding |
| ENSMUSG00000020142 | Slc1a4 | solute carrier family 1 (glutamate/neutral amino acid transporter), member 4 [Source:MGI Symbol;Acc:MGI:2135601] | 10,73597393 | -1,1985129 | **-2,29502976** | 3,4025025 | 15,1426971 | 9,96831E-05 | 0,000679416 | protein_coding |
| ENSMUSG00000063450 | Syne2 | synaptic nuclear envelope 2 [Source:MGI Symbol;Acc:MGI:2449316] | 8,30079823 | -1,1986261 | **-2,29520989** | 3,03695206 | 22,4050834 | 2,20789E-06 | 2,22144E-05 | protein_coding |
| ENSMUSG00000046207 | Pik3r6 | phosphoinositide-3-kinase, regulatory subunit 6 [Source:MGI Symbol;Acc:MGI:2144613] | 89,94599951 | -1,2013364 | **-2,29952576** | 6,48584566 | 38,0687804 | 6,82942E-10 | 1,31871E-08 | protein_coding |
| ENSMUSG00000071107 | 4732465J04Rik | RIKEN cDNA 4732465J04 gene [Source:MGI Symbol;Acc:MGI:3041208] | 1,801718074 | -1,2016038 | **-2,29995212** | 0,79473282 | 7,59366556 | 0,005857373 | 0,023174275 | protein_coding |
| ENSMUSG00000061751 | Kalrn | kalirin, RhoGEF kinase [Source:MGI Symbol;Acc:MGI:2685385] | 6,240458624 | -1,2117406 | **-2,31616916** | 2,64747961 | 18,8584246 | 1,40788E-05 | 0,000118805 | protein_coding |
| ENSMUSG00000074886 | Grk6 | G protein-coupled receptor kinase 6 [Source:MGI Symbol;Acc:MGI:1347078] | 121,5798382 | -1,2160175 | **-2,32304568** | 6,92270292 | 51,8713139 | 5,92599E-13 | 1,81362E-11 | protein_coding |
| ENSMUSG00000055862 | Izumo4 | IZUMO family member 4 [Source:MGI Symbol;Acc:MGI:1918814] | 2,470705289 | -1,2193823 | **-2,32846998** | 1,25658872 | 8,49839122 | 0,003554606 | 0,015243739 | protein_coding |
| ENSMUSG00000030930 | Chst15 | carbohydrate (N-acetylgalactosamine 4-sulfate 6-O) sulfotransferase 15 [Source:MGI Symbol;Acc:MGI:1924840] | 4,496065539 | -1,2195073 | **-2,32867173** | 2,16200674 | 13,8618719 | 0,00019675 | 0,001234546 | protein_coding |
| ENSMUSG00000045731 | Pnoc | prepronociceptin [Source:MGI Symbol;Acc:MGI:105308] | 2,196114679 | -1,2234933 | **-2,33511454** | 1,10743996 | 7,58362269 | 0,005890095 | 0,023270217 | protein_coding |
| ENSMUSG00000000682 | Cd52 | CD52 antigen [Source:MGI Symbol;Acc:MGI:1346088] | 651,0214106 | -1,2258904 | **-2,33899762** | 9,3462488 | 40,1491714 | 2,35291E-10 | 4,97143E-09 | protein_coding |
| ENSMUSG00000037405 | Icam1 | intercellular adhesion molecule 1 [Source:MGI Symbol;Acc:MGI:96392] | 311,1730771 | -1,226272 | **-2,33961641** | 8,28165845 | 45,2425659 | 1,7408E-11 | 4,36671E-10 | protein_coding |

| | | | | | | | | | | |
|---|---|---|---|---|---|---|---|---|---|---|
| ENSMUSG00000025792 | Slc25a10 | solute carrier family 25 (mitochondrial carrier, dicarboxylate transporter), member 10 [Source:MGI Symbol;Acc:MGI:1353 | 262,4902229 | -1,2317632 | **-2,34853844** | 8,03512619 | 47,1586093 | 6,54685E-12 | 1,72909E-10 | protein_coding |
| ENSMUSG00000059743 | Fdps | farnesyl diphosphate synthetase [Source:MGI Symbol;Acc:MGI:104888] | 66,03870724 | -1,2402431 | **-2,3623833** | 6,04149451 | 16,0303619 | 6,23348E-05 | 0,000450204 | protein_coding |
| ENSMUSG00000001508 | Sgca | sarcoglycan, alpha (dystrophin-associated glycoprotein) [Source:MGI Symbol;Acc:MGI:894698] | 3,895322761 | -1,2406737 | **-2,36308862** | 1,94531256 | 11,7078831 | 0,000622359 | 0,003396849 | protein_coding |
| ENSMUSG00000055202 | Zfp811 | zinc finger protein 811 [Source:MGI Symbol;Acc:MGI:2682944] | 31,09192966 | -1,2464185 | **-2,37251713** | 4,94873911 | 65,6059542 | 5,50721E-16 | 2,67733E-14 | protein_coding |
| ENSMUSG00000044827 | Tlr1 | toll-like receptor 1 [Source:MGI Symbol;Acc:MGI:1341295] | 359,2160426 | -1,259983 | **-2,39492919** | 8,48841319 | 35,7845803 | 2,20385E-09 | 3,95649E-08 | protein_coding |
| ENSMUSG00000017652 | Cd40 | CD40 antigen [Source:MGI Symbol;Acc:MGI:88336] | 24,96422247 | -1,2622229 | **-2,39865042** | 4,64419257 | 50,9432334 | 9,50762E-13 | 2,80807E-11 | protein_coding |
| ENSMUSG00000004665 | Cnn2 | calponin 2 [Source:MGI Symbol;Acc:MGI:105093] | 126,9071343 | -1,2670863 | **-2,40675007** | 6,9855819 | 61,8902916 | 3,63136E-15 | 1,55244E-13 | protein_coding |
| ENSMUSG00000052364 | B630019K06Rik | RIKEN cDNA B630019K06 gene [Source:MGI Symbol;Acc:MGI:2147918] | 4,351895422 | -1,2672491 | **-2,40702155** | 2,07265654 | 16,6778524 | 4,42951E-05 | 0,000328831 | protein_coding |
| ENSMUSG00000047786 | Lix1 | limb expression 1 homolog (chicken) [Source:MGI Symbol;Acc:MGI:1913893] | 1,667719046 | -1,2698127 | **-2,41130263** | 0,75468334 | 7,61882156 | 0,005776222 | 0,022894427 | protein_coding |
| ENSMUSG00000096991 | | | 2,366168721 | -1,275665 | **-2,42110388** | 1,19508229 | 10,9303603 | 0,000946011 | 0,004895724 | lincRNA |
| ENSMUSG00000015852 | Fcrls | Fc receptor-like S, scavenger receptor [Source:MGI Symbol;Acc:MGI:1933397] | 3710,161412 | -1,2796935 | **-2,42787394** | 11,8572332 | 21,9772294 | 2,75904E-06 | 2,73096E-05 | protein_coding |
| ENSMUSG00000026458 | Ppfia4 | protein tyrosine phosphatase, receptor type, f polypeptide (PTPRF), interacting protein (liprin), alpha 4 [Source:MGI Sym | 13,39566561 | -1,2817992 | **-2,43142021** | 3,72131152 | 20,9906248 | 4,61536E-06 | 4,36019E-05 | protein_coding |
| ENSMUSG00000087412 | Gm15501 | predicted pseudogene 15501 [Source:MGI Symbol;Acc:MGI:3704296] | 14,74107863 | -1,2824791 | **-2,43256618** | 3,84017996 | 17,2675156 | 3,2469E-05 | 0,000249359 | pseudogene |
| ENSMUSG00000009292 | Trpm2 | transient receptor potential cation channel, subfamily M, member 2 [Source:MGI Symbol;Acc:MGI:1351901] | 53,33265586 | -1,2859649 | **-2,43845088** | 5,72591603 | 54,5444112 | 1,51974E-13 | 5,09067E-12 | protein_coding |
| ENSMUSG00000040724 | Kcna2 | potassium voltage-gated channel, shaker-related subfamily, member 2 [Source:MGI Symbol;Acc:MGI:96659] | 3,50764416 | -1,2860275 | **-2,43855662** | 1,84295861 | 14,0613004 | 0,000176947 | 0,00112507 | protein_coding |
| ENSMUSG00000033768 | Nrxn2 | neurexin II [Source:MGI Symbol;Acc:MGI:1096362] | 38,09537487 | -1,2868113 | **-2,43988184** | 5,24365988 | 47,954409 | 4,36246E-12 | 1,18055E-10 | protein_coding |
| ENSMUSG00000043257 | Pigv | phosphatidylinositol glycan anchor biosynthesis, class V [Source:MGI Symbol;Acc:MGI:2442480] | 21,28494706 | -1,288525 | **-2,44278182** | 4,40025028 | 31,6322483 | 1,8631E-08 | 2,83909E-07 | protein_coding |
| ENSMUSG00000049744 | Arhgap15 | Rho GTPase activating protein 15 [Source:MGI Symbol;Acc:MGI:1923367] | 28,88829173 | -1,2931138 | **-2,45056397** | 4,83852736 | 61,7496445 | 3,90022E-15 | 1,65451E-13 | protein_coding |
| ENSMUSG00000073412 | Lst1 | leukocyte specific transcript 1 [Source:MGI Symbol;Acc:MGI:1096324] | 298,3069071 | -1,2957527 | **-2,45505045** | 8,21979187 | 57,0398444 | 4,27071E-14 | 1,56408E-12 | protein_coding |
| ENSMUSG00000040010 | Slc7a5 | solute carrier family 7 (cationic amino acid transporter, y+ system), member 5 [Source:MGI Symbol;Acc:MGI:1298205] | 40,09213284 | -1,2971519 | **-2,45743266** | 5,31633463 | 28,5110728 | 9,31642E-08 | 1,23028E-06 | protein_coding |
| ENSMUSG00000079481 | Nhsl2 | NHS-like 2 [Source:MGI Symbol;Acc:MGI:3645090] | 31,79768123 | -1,2996266 | **-2,46165161** | 4,98231626 | 41,9717185 | 9,26031E-11 | 2,06375E-09 | protein_coding |
| ENSMUSG00000057191 | AB124611 | cDNA sequence AB124611 [Source:MGI Symbol;Acc:MGI:3043001] | 180,5476644 | -1,3048923 | **-2,47065288** | 7,49388486 | 46,1137358 | 1,11583E-11 | 2,85284E-10 | protein_coding |
| ENSMUSG00000049612 | Omg | oligodendrocyte myelin glycoprotein [Source:MGI Symbol;Acc:MGI:106586] | 2,93021745 | -1,3083972 | **-2,47666243** | 1,52662433 | 12,7790635 | 0,00035052 | 0,002052861 | protein_coding |
| ENSMUSG00000024845 | Tmem134 | transmembrane protein 134 [Source:MGI Symbol;Acc:MGI:1914240] | 200,6796849 | -1,3103567 | **-2,48002858** | 7,64663239 | 59,3458889 | 1,32256E-14 | 5,15283E-13 | processed_transcrip |
| ENSMUSG00000048721 | Fndc9 | fibronectin type III domain containing 9 [Source:MGI Symbol;Acc:MGI:2443410] | 2,013300915 | -1,310849 | **-2,48087489** | 1,00478891 | 8,87069435 | 0,002897849 | 0,012838172 | protein_coding |
| ENSMUSG00000039304 | Tnfsf10 | tumor necrosis factor (ligand) superfamily, member 10 [Source:MGI Symbol;Acc:MGI:107414] | 10,05503667 | -1,3112865 | **-2,48162738** | 3,31669753 | 24,2001877 | 8,68238E-07 | 9,59691E-06 | protein_coding |
| ENSMUSG00000059742 | Kcnh7 | potassium voltage-gated channel, subfamily H (eag-related), member 7 [Source:MGI Symbol;Acc:MGI:2159566] | 2,022466588 | -1,3136874 | **-2,48576071** | 1,01494854 | 10,5927102 | 0,001135344 | 0,005716786 | protein_coding |
| ENSMUSG00000029185 | Fam114a1 | family with sequence similarity 114, member A1 [Source:MGI Symbol;Acc:MGI:1915553] | 69,39668383 | -1,3172553 | **-2,49191576** | 6,11497467 | 44,3289505 | 2,77581E-11 | 6,7324E-10 | protein_coding |
| ENSMUSG00000087362 | Gm13710 | predicted gene 13710 [Source:MGI Symbol;Acc:MGI:3650894] | 9,47178676 | -1,3175002 | **-2,49233878** | 3,1888768 | 33,1621138 | 8,47865E-09 | 1,35597E-07 | lincRNA |
| ENSMUSG00000040520 | Manea | mannosidase, endo-alpha [Source:MGI Symbol;Acc:MGI:2444484] | 124,0648985 | -1,3270525 | **-2,50889578** | 6,9521181 | 70,8756638 | 3,80462E-17 | 2,03909E-15 | protein_coding |
| ENSMUSG00000079363 | Gbp4 | guanylate binding protein 4 [Source:MGI Symbol;Acc:MGI:97072] | 1,887545786 | -1,3282718 | **-2,511017** | 0,94473022 | 9,1948283 | 0,002426999 | 0,011073685 | protein_coding |
| ENSMUSG00000036330 | Slc18a1 | solute carrier family 18 (vesicular monoamine), member 1 [Source:MGI Symbol;Acc:MGI:106684] | 8,728239897 | -1,3334776 | **-2,52009413** | 3,11472507 | 32,1008106 | 1,46376E-08 | 2,26356E-07 | protein_coding |
| ENSMUSG00000002699 | Lcp2 | lymphocyte cytosolic protein 2 [Source:MGI Symbol;Acc:MGI:1321402] | 415,6792528 | -1,3346934 | **-2,52221873** | 8,69912153 | 42,0139624 | 9,0624E-11 | 2,02787E-09 | protein_coding |
| ENSMUSG00000032356 | Rasgrf1 | RAS protein-specific guanine nucleotide-releasing factor 1 [Source:MGI Symbol;Acc:MGI:99694] | 6,021429449 | -1,3397294 | **-2,53103842** | 2,57326998 | 15,190347 | 9,7199E-05 | 0,000663308 | protein_coding |
| ENSMUSG00000002104 | Rapsn | receptor-associated protein of the synapse [Source:MGI Symbol;Acc:MGI:99422] | 3,688170498 | -1,3417635 | **-2,5346095** | 1,83491153 | 9,4040357 | 0,002165084 | 0,010043308 | protein_coding |
| ENSMUSG00000005125 | Ndrg1 | N-myc downstream regulated gene 1 [Source:MGI Symbol;Acc:MGI:1341799] | 2759,827188 | -1,3431881 | **-2,53711364** | 11,4303323 | 25,1116841 | 5,4104E-07 | 6,14092E-06 | protein_coding |
| ENSMUSG00000031434 | Morc4 | microrchidia 4 [Source:MGI Symbol;Acc:MGI:1922996] | 84,50457011 | -1,3456273 | **-2,5414068** | 6,3985154 | 88,6557636 | 4,69856E-21 | 4,12984E-19 | protein_coding |
| ENSMUSG00000016763 | Scube1 | signal peptide, CUB domain, EGF-like 1 [Source:MGI Symbol;Acc:MGI:1890616] | 1,995898546 | -1,3490865 | **-2,54750767** | 1,02132175 | 10,6720746 | 0,001087651 | 0,005512001 | protein_coding |
| ENSMUSG00000008305 | Tle1 | transducin-like enhancer of split 1, homolog of Drosophila E(spl) [Source:MGI Symbol;Acc:MGI:104636] | 30,65522632 | -1,3624813 | **-2,57127033** | 4,93197426 | 63,3242175 | 1,75335E-15 | 7,9934E-14 | protein_coding |
| ENSMUSG00000001249 | Hpn | hepsin [Source:MGI Symbol;Acc:MGI:1196620] | 42,16819248 | -1,3656106 | **-2,57685356** | 5,39236895 | 42,6188834 | 6,65142E-11 | 1,52885E-09 | protein_coding |
| ENSMUSG00000078670 | Fam174b | family with sequence similarity 174, member B [Source:MGI Symbol;Acc:MGI:3698178] | 15,33345619 | -1,3719174 | **-2,58814319** | 3,92851764 | 44,9193388 | 2,0532E-11 | 5,05796E-10 | protein_coding |
| ENSMUSG00000026815 | Gfi1b | growth factor independent 1B [Source:MGI Symbol;Acc:MGI:1276578] | 8,589244016 | -1,3790908 | **-2,60104407** | 3,0825821 | 30,3288458 | 3,64662E-08 | 5,27177E-07 | protein_coding |
| ENSMUSG00000015854 | Cd5l | CD5 antigen-like [Source:MGI Symbol;Acc:MGI:1334419] | 1973,355342 | -1,3812829 | **-2,60499913** | 10,9463244 | 32,1678644 | 1,4141E-08 | 2,19446E-07 | protein_coding |
| ENSMUSG00000037347 | Chst7 | carbohydrate (N-acetylglucosamino) sulfotransferase 7 [Source:MGI Symbol;Acc:MGI:1891767] | 19,05352789 | -1,3840209 | **-2,60994771** | 4,24126048 | 39,356354 | 3,53102E-10 | 7,26504E-09 | protein_coding |
| ENSMUSG00000097485 | | Cyclin-dependent kinase 2-associated protein 2 [Source:UniProtKB/TrEMBL;Acc:G3UX88] | 39,65563027 | -1,3880169 | **-2,61718679** | 5,29790519 | 45,1239082 | 1,84954E-11 | 4,6151E-10 | protein_coding |
| ENSMUSG00000024856 | Cdk2ap2 | CDK2-associated protein 2 [Source:MGI Symbol;Acc:MGI:1098779] | 39,65563027 | -1,3880182 | **-2,61718916** | 5,29790599 | 45,1208526 | 1,85242E-11 | 4,6151E-10 | protein_coding |
| ENSMUSG00000089753 | Gm11709 | predicted gene 11709 [Source:MGI Symbol;Acc:MGI:3649405] | 5,367595226 | -1,3916745 | **-2,62383051** | 2,41588922 | 17,0926345 | 3,56001E-05 | 0,000269937 | protein_coding |
| ENSMUSG00000048107 | Kctd12 | potassium channel tetramerisation domain containing 12 [Source:MGI Symbol;Acc:MGI:2145823] | 409,5762753 | -1,393401 | **-2,62697232** | 8,67483037 | 61,4639391 | 4,50927E-15 | 1,89097E-13 | processed_pseudoge |
| ENSMUSG00000045730 | Adrb2 | adrenergic receptor, beta 2 [Source:MGI Symbol;Acc:MGI:87938] | 24,00848838 | -1,4018515 | **-2,64240473** | 4,57121785 | 56,1861223 | 6,59252E-14 | 2,35169E-12 | protein_coding |
| ENSMUSG00000080875 | Gm7332 | predicted gene 7332 [Source:MGI Symbol;Acc:MGI:3643344] | 9,737954855 | -1,4029911 | **-2,64449289** | 3,28062989 | 16,8788681 | 3,98427E-05 | 0,000299326 | pseudogene |
| ENSMUSG00000022372 | Sla | src-like adaptor [Source:MGI Symbol;Acc:MGI:104295] | 274,8978275 | -1,4036955 | **-2,64578433** | 8,10201223 | 43,073617 | 5,27184E-11 | 1,235E-09 | protein_coding |
| ENSMUSG00000029153 | Ociad2 | OCIA domain containing 2 [Source:MGI Symbol;Acc:MGI:1916377] | 14,10227805 | -1,4210097 | **-2,67772849** | 3,81007444 | 52,8210968 | 3,65356E-13 | 1,15019E-11 | protein_coding |
| ENSMUSG00000075254 | Heg1 | HEG homolog 1 (zebrafish) [Source:MGI Symbol;Acc:MGI:1924696] | 93,01777871 | -1,424349 | **-2,68393362** | 6,54138293 | 68,2410213 | 1,44684E-16 | 7,42826E-15 | protein_coding |
| ENSMUSG00000056592 | Zfp658 | zinc finger protein 658 [Source:MGI Symbol;Acc:MGI:2652821] | 10,04647063 | -1,4251147 | **-2,68535849** | 3,31275328 | 29,77844 | 4,84349E-08 | 6,82248E-07 | protein_coding |
| ENSMUSG00000045886 | Gm9803 | predicted gene 9803 [Source:MGI Symbol;Acc:MGI:3704359] | 6,010867954 | -1,4341248 | **-2,70218198** | 2,58792274 | 7,93888172 | 0,004838373 | 0,019641921 | protein_coding |
| ENSMUSG00000090936 | Gm17705 | predicted gene, 17705 [Source:MGI Symbol;Acc:MGI:4937339] | 19,94645334 | -1,4374408 | **-2,70839992** | 4,30318505 | 47,009767 | 7,06338E-12 | 1,85659E-10 | lincRNA |
| ENSMUSG00000023367 | Tmem176a | transmembrane protein 176A [Source:MGI Symbol;Acc:MGI:1913308] | 632,4906275 | -1,4418514 | **-2,71669264** | 9,30457927 | 38,5552686 | 5,32249E-10 | 1,05747E-08 | protein_coding |
| ENSMUSG00000033577 | Myo6 | myosin VI [Source:MGI Symbol;Acc:MGI:104785] | 12,85521981 | -1,4457467 | **-2,72403769** | 3,6714972 | 44,1671376 | 3,01501E-11 | 7,26445E-10 | protein_coding |
| ENSMUSG00000096334 | Sh2d1b1 | SH2 domain protein 1B1 [Source:MGI Symbol;Acc:MGI:1349420] | 9,892031907 | -1,4488484 | **-2,72990047** | 3,28126683 | 33,9264669 | 5,72348E-09 | 9,48475E-08 | protein_coding |
| ENSMUSG00000037995 | Igsf9 | immunoglobulin superfamily, member 9 [Source:MGI Symbol;Acc:MGI:2135283] | 64,12500538 | -1,44904 | **-2,73026311** | 5,99448239 | 76,2439338 | 2,50702E-18 | 1,53025E-16 | protein_coding |
| ENSMUSG00000045294 | Insig1 | insulin induced gene 1 [Source:MGI Symbol;Acc:MGI:1916289] | 81,83158806 | -1,4592603 | **-2,74967347** | 6,35095456 | 30,2459387 | 3,80587E-08 | 5,48755E-07 | protein_coding |
| ENSMUSG00000024397 | Aif1 | allograft inflammatory factor 1 [Source:MGI Symbol;Acc:MGI:1343098] | 472,970096 | -1,4607047 | **-2,75242773** | 8,88529345 | 55,02285 | 1,19137E-13 | 4,06508E-12 | protein_coding |
| ENSMUSG00000044456 | Rin3 | Ras and Rab interactor 3 [Source:MGI Symbol;Acc:MGI:2385708] | 105,0769853 | -1,4645502 | **-2,7597741** | 6,71104669 | 44,9559361 | 2,01518E-11 | 4,97807E-10 | protein_coding |
| ENSMUSG00000022844 | Pdia5 | protein disulfide isomerase associated 5 [Source:MGI Symbol;Acc:MGI:1919849] | 7,70449666 | -1,4667715 | **-2,76402653** | 2,94201127 | 22,6092166 | 1,9853E-06 | 2,0253E-05 | protein_coding |
| ENSMUSG00000039081 | Zfp503 | zinc finger protein 503 [Source:MGI Symbol;Acc:MGI:1353644] | 14,52994129 | -1,4688411 | **-2,76799455** | 3,8314425 | 43,2865759 | 4,72816E-11 | 1,11958E-09 | protein_coding |
| ENSMUSG00000075514 | Gm13375 | predicted gene 13375 [Source:MGI Symbol;Acc:MGI:3649913] | 8,03847294 | -1,4734122 | **-2,77677877** | 2,97367129 | 27,8367812 | 1,31993E-07 | 1,69416E-06 | antisense |
| ENSMUSG00000036591 | Arhgap21 | Rho GTPase activating protein 21 [Source:MGI Symbol;Acc:MGI:1918685] | 273,820975 | -1,4748655 | **-2,77957736** | 8,09598006 | 58,7683848 | 1,77368E-14 | 6,74307E-13 | protein_coding |
| ENSMUSG00000012017 | Scarf2 | scavenger receptor class F, member 2 [Source:MGI Symbol;Acc:MGI:1858430] | 17,86241345 | -1,4835373 | **-2,79633511** | 4,1495885 | 39,4411763 | 3,38092E-10 | 6,98236E-09 | protein_coding |
| ENSMUSG00000018341 | Il12rb2 | interleukin 12 receptor, beta 2 [Source:MGI Symbol;Acc:MGI:1270861] | 3,007988565 | -1,4914934 | **-2,81179884** | 1,59051349 | 16,2727596 | 5,48468E-05 | 0,000400933 | protein_coding |
| ENSMUSG00000024042 | Sik1 | salt inducible kinase 1 [Source:MGI Symbol;Acc:MGI:104754] | 70,35992288 | -1,5015111 | **-2,83139117** | 6,13139541 | 62,9177853 | 2,15517E-15 | 9,66485E-14 | protein_coding |
| ENSMUSG00000039116 | Gpr126 | G protein-coupled receptor 126 [Source:MGI Symbol;Acc:MGI:1916151] | 208,1270099 | -1,5015383 | **-2,83144461** | 7,70045952 | 76,8295412 | 1,86369E-18 | 1,1768E-16 | protein_coding |

| | | | | | | | | | | |
|---|---|---|---|---|---|---|---|---|---|---|
| ENSMUSG00000037318 | Traf3ip3 | TRAF3 interacting protein 3 [Source:MGI Symbol;Acc:MGI:2441706] | 28,25468956 | -1,5028907 | **-2,83409999** | 4,80798623 | 40,9362597 | 1,57276E-10 | 3,38822E-09 | protein_coding |
| ENSMUSG00000024013 | Fgd2 | FYVE, RhoGEF and PH domain containing 2 [Source:MGI Symbol;Acc:MGI:1347084] | 225,0690629 | -1,5120562 | **-2,85216256** | 7,81211844 | 57,7090803 | 3,03897E-14 | 1,12421E-12 | protein_coding |
| ENSMUSG00000030621 | Me3 | malic enzyme 3, NADP(+)-dependent, mitochondrial [Source:MGI Symbol;Acc:MGI:1916679] | 2,367177548 | -1,5167075 | **-2,86137293** | 1,18562366 | 15,136619 | 0,000100005 | 0,000680897 | protein_coding |
| ENSMUSG00000030711 | Sult1a1 | sulfotransferase family 1A, phenol-preferring, member 1 [Source:MGI Symbol;Acc:MGI:102896] | 2,057816212 | -1,5289531 | **-2,88576351** | 1,01274323 | 12,6006852 | 0,000385605 | 0,002240426 | protein_coding |
| ENSMUSG00000050199 | Lgr4 | leucine-rich repeat-containing G protein-coupled receptor 4 [Source:MGI Symbol;Acc:MGI:1891468] | 6,665210807 | -1,5297365 | **-2,88733089** | 2,71136791 | 25,6533747 | 4,0858E-07 | 4,72037E-06 | protein_coding |
| ENSMUSG00000051236 | Msrb3 | methionine sulfoxide reductase B3 [Source:MGI Symbol;Acc:MGI:2443538] | 4,916338096 | -1,5305825 | **-2,88902467** | 2,29979286 | 25,7312486 | 3,92421E-07 | 4,56247E-06 | protein_coding |
| ENSMUSG00000079559 | Gm684 | predicted gene 684 [Source:MGI Symbol;Acc:MGI:2685530] | 4,468153617 | -1,5374765 | **-2,90286309** | 2,13647674 | 15,0044042 | 0,000107261 | 0,000724767 | protein_coding |
| ENSMUSG00000026655 | Fam107b | family with sequence similarity 107, member B [Source:MGI Symbol;Acc:MGI:1913790] | 138,8356265 | -1,5411656 | **-2,91029547** | 7,1144475 | 68,1826634 | 1,4903E-16 | 7,61579E-15 | protein_coding |
| ENSMUSG00000026630 | Batf3 | basic leucine zipper transcription factor, ATF-like 3 [Source:MGI Symbol;Acc:MGI:1925491] | 16,3479757 | -1,5431613 | **-2,91432412** | 4,01878739 | 41,8128052 | 1,00443E-10 | 2,22494E-09 | protein_coding |
| ENSMUSG00000009145 | Dqx1 | DEAQ RNA-dependent ATPase [Source:MGI Symbol;Acc:MGI:2136388] | 2,776841228 | -1,5432692 | **-2,91454206** | 1,44461411 | 14,1410853 | 0,000169599 | 0,001084297 | protein_coding |
| ENSMUSG00000027750 | Postn | periostin, osteoblast specific factor [Source:MGI Symbol;Acc:MGI:1926321] | 9,747932871 | -1,5441694 | **-2,91636114** | 3,31759068 | 44,9549132 | 2,01624E-11 | 4,97807E-10 | protein_coding |
| ENSMUSG00000035275 | Raver2 | ribonucleoprotein, PTB-binding 2 [Source:MGI Symbol;Acc:MGI:2443623] | 216,1992468 | -1,546011 | **-2,92008633** | 7,7551261 | 56,5981864 | 5,34611E-14 | 1,93216E-12 | protein_coding |
| ENSMUSG00000020717 | Pecam1 | platelet/endothelial cell adhesion molecule 1 [Source:MGI Symbol;Acc:MGI:97537] | 21,08276466 | -1,5554016 | **-2,93915523** | 4,38313997 | 39,1335161 | 3,95791E-10 | 8,06782E-09 | protein_coding |
| ENSMUSG00000023032 | Slc4a8 | solute carrier family 4 (anion exchanger), member 8 [Source:MGI Symbol;Acc:MGI:1928745] | 3,749458662 | -1,5625662 | **-2,9537879** | 1,86170035 | 20,1885888 | 7,01703E-06 | 6,32973E-05 | protein_coding |
| ENSMUSG00000004328 | Hif3a | hypoxia inducible factor 3, alpha subunit [Source:MGI Symbol;Acc:MGI:1859778] | 7,150145371 | -1,5628367 | **-2,95434164** | 2,82014599 | 24,6314119 | 6,9411E-07 | 7,75019E-06 | protein_coding |
| ENSMUSG00000034926 | Dhcr24 | 24-dehydrocholesterol reductase [Source:MGI Symbol;Acc:MGI:1922004] | 40,54635481 | -1,5703551 | **-2,96977801** | 5,33603161 | 15,4720454 | 8,37345E-05 | 0,000584493 | protein_coding |
| ENSMUSG00000045282 | Tmem86b | transmembrane protein 86B [Source:MGI Symbol;Acc:MGI:1915505] | 2,341448051 | -1,576185 | **-2,98180312** | 1,21179438 | 13,4617394 | 0,000243478 | 0,001491133 | protein_coding |
| ENSMUSG00000039899 | Fgl2 | fibrinogen-like protein 2 [Source:MGI Symbol;Acc:MGI:103266] | 2003,179043 | -1,5772992 | **-2,98410692** | 10,9679941 | 37,1360569 | 1,10168E-09 | 2,06203E-08 | protein_coding |
| ENSMUSG00000040297 | Suco | SUN domain containing ossification factor [Source:MGI Symbol;Acc:MGI:2138346] | 224,5064108 | -1,5867923 | **-3,00380744** | 7,80983002 | 86,9115527 | 1,13485E-20 | 9,10113E-19 | protein_coding |
| ENSMUSG00000034145 | Tmem63c | transmembrane protein 63c [Source:MGI Symbol;Acc:MGI:2444386] | 31,21520405 | -1,5887208 | **-3,00782536** | 4,95860641 | 56,6759831 | 5,13873E-14 | 1,86334E-12 | protein_coding |
| ENSMUSG00000031155 | Pim2 | proviral integration site 2 [Source:MGI Symbol;Acc:MGI:97587] | 144,2347977 | -1,5972442 | **-3,02564806** | 7,17058144 | 72,7299925 | 1,48658E-17 | 8,37591E-16 | protein_coding |
| ENSMUSG00000085389 | 1700003M07Rik | RIKEN cDNA 1700003M07 gene [Source:MGI Symbol;Acc:MGI:1919475] | 23,26411893 | -1,6022454 | **-3,03615487** | 4,52962799 | 49,2806351 | 2,21842E-12 | 6,33679E-11 | lincRNA |
| ENSMUSG00000006576 | Slc4a3 | solute carrier family 4 (anion exchanger), member 3 [Source:MGI Symbol;Acc:MGI:109350] | 31,55838171 | -1,606141 | **-3,0443642** | 4,96897884 | 60,8431483 | 6,18083E-15 | 2,53391E-13 | protein_coding |
| ENSMUSG00000034438 | Gbp8 | guanylate-binding protein 8 [Source:MGI Symbol;Acc:MGI:1923324] | 3,971092587 | -1,6069054 | **-3,04597772** | 1,94172689 | 17,4912328 | 2,88636E-05 | 0,000224602 | protein_coding |
| ENSMUSG00000020785 | Camkk1 | calcium/calmodulin-dependent protein kinase kinase 1, alpha [Source:MGI Symbol;Acc:MGI:1891766] | 10,17450631 | -1,6085868 | **-3,04952972** | 3,32620521 | 25,0869907 | 5,48013E-07 | 6,21364E-06 | protein_coding |
| ENSMUSG00000074342 | I830077J02Rik | RIKEN cDNA I830077J02 gene [Source:MGI Symbol;Acc:MGI:3588284] | 6,931197944 | -1,6236975 | **-3,08163839** | 2,76250175 | 23,5054158 | 1,24563E-06 | 1,3172E-05 | protein_coding |
| ENSMUSG00000039943 | Plcb4 | phospholipase C, beta 4 [Source:MGI Symbol;Acc:MGI:107464] | 5,072978127 | -1,64097 | **-3,11875457** | 2,29174549 | 28,7646639 | 8,17287E-08 | 1,08975E-06 | protein_coding |
| ENSMUSG00000041132 | N4bp2l1 | NEDD4 binding protein 2-like 1 [Source:MGI Symbol;Acc:MGI:2140872] | 31,70296399 | -1,6458278 | **-3,12927367** | 4,97415695 | 62,1104383 | 3,24726E-15 | 1,41578E-13 | protein_coding |
| ENSMUSG00000030830 | Itgal | integrin alpha L [Source:MGI Symbol;Acc:MGI:96606] | 1399,134373 | -1,6504672 | **-3,13935279** | 10,4502535 | 42,0248161 | 9,01224E-11 | 2,02077E-09 | protein_coding |
| ENSMUSG00000037922 | Bank1 | B cell scaffold protein with ankyrin repeats 1 [Source:MGI Symbol;Acc:MGI:2442120] | 4,2058836 | -1,6505566 | **-3,1395475** | 2,05255433 | 19,7221046 | 8,95594E-06 | 7,87822E-05 | protein_coding |
| ENSMUSG00000036526 | Card11 | caspase recruitment domain family, member 11 [Source:MGI Symbol;Acc:MGI:1916978] | 106,139399 | -1,6557901 | **-3,15095704** | 6,72393522 | 86,0791018 | 1,7288E-20 | 1,35674E-18 | protein_coding |
| ENSMUSG00000030683 | Sez6l2 | seizure related 6 homolog like 2 [Source:MGI Symbol;Acc:MGI:2385295] | 56,41254645 | -1,6672285 | **-3,17603877** | 5,81293119 | 99,0690258 | 2,43853E-23 | 2,55163E-21 | protein_coding |
| ENSMUSG00000092901 | Mir5130 | microRNA 5130 [Source:MGI Symbol;Acc:MGI:4950455] | 3,875320713 | -1,6748695 | **-3,19290465** | 1,88836267 | 21,5468557 | 3,45288E-06 | 3,34835E-05 | miRNA |
| ENSMUSG00000030263 | Lrmp | lymphoid-restricted membrane protein [Source:MGI Symbol;Acc:MGI:108424] | 297,2753957 | -1,6872524 | **-3,22042793** | 8,21434645 | 65,2492779 | 6,59984E-16 | 3,18037E-14 | protein_coding |
| ENSMUSG00000000732 | Icosl | icos ligand [Source:MGI Symbol;Acc:MGI:1354701] | 32,94154682 | -1,6923395 | **-3,23180343** | 5,03350525 | 65,9125466 | 4,71381E-16 | 2,32245E-14 | protein_coding |
| ENSMUSG00000024074 | Crim1 | cysteine rich transmembrane BMP regulator 1 (chordin like) [Source:MGI Symbol;Acc:MGI:1354756] | 88,23129157 | -1,6952581 | **-3,23834825** | 6,45885199 | 78,6909429 | 7,26278E-19 | 4,83613E-17 | protein_coding |
| ENSMUSG00000049191 | Rgag4 | retrotransposon gag domain containing 4 [Source:MGI Symbol;Acc:MGI:3045324] | 189,0032534 | -1,7211687 | **-3,29703396** | 7,56062731 | 75,2573208 | 4,13195E-18 | 2,45393E-16 | protein_coding |
| ENSMUSG00000039611 | Tmem246 | transmembrane protein 246 [Source:MGI Symbol;Acc:MGI:1914313] | 1,82127017 | -1,7288256 | **-3,31457888** | 0,85690694 | 15,2905337 | 9,21773E-05 | 0,000633762 | protein_coding |
| ENSMUSG00000086184 | Gm12764 | predicted gene 12764 [Source:MGI Symbol;Acc:MGI:3649980] | 5,400334025 | -1,7387881 | **-3,33754686** | 2,41311878 | 28,0331826 | 1,19253E-07 | 1,54327E-06 | antisense |
| ENSMUSG00000004633 | Chn2 | chimerin (chimaerin) 2 [Source:MGI Symbol;Acc:MGI:1917243] | 2,661515516 | -1,7422652 | **-3,34560049** | 1,36557254 | 19,6632099 | 9,23626E-06 | 8,08241E-05 | protein_coding |
| ENSMUSG00000048031 | Fcrl5 | Fc receptor-like 5 [Source:MGI Symbol;Acc:MGI:3053558] | 115,6481137 | -1,7506073 | **-3,36500181** | 6,85265601 | 92,1868506 | 7,88671E-22 | 7,40609E-20 | protein_coding |
| ENSMUSG00000031257 | Nox1 | NADPH oxidase 1 [Source:MGI Symbol;Acc:MGI:2450016] | 8,911652443 | -1,7655681 | **-3,40007851** | 3,14870229 | 45,187432 | 1,7905E-11 | 4,48116E-10 | protein_coding |
| ENSMUSG00000036432 | Siah2 | seven in absentia 2 [Source:MGI Symbol;Acc:MGI:108062] | 56,10265053 | -1,7901719 | **-3,45856099** | 5,80324755 | 66,9073684 | 2,84569E-16 | 1,4159E-14 | protein_coding |
| ENSMUSG00000030029 | Lrig1 | leucine-rich repeats and immunoglobulin-like domains 1 [Source:MGI Symbol;Acc:MGI:107935] | 3,145423919 | -1,8270736 | **-3,54816612** | 1,62538566 | 22,5903048 | 2,00494E-06 | 2,03965E-05 | protein_coding |
| ENSMUSG00000041594 | Tmtc4 | transmembrane and tetratricopeptide repeat containing 4 [Source:MGI Symbol;Acc:MGI:1921050] | 216,8780649 | -1,8331362 | **-3,56310799** | 7,75923799 | 87,0235949 | 1,07234E-20 | 8,6631E-19 | protein_coding |
| ENSMUSG00000031523 | Dlc1 | deleted in liver cancer 1 [Source:MGI Symbol;Acc:MGI:1354949] | 3,75969267 | -1,8336578 | **-3,56439647** | 1,8866636 | 28,8335401 | 7,88733E-08 | 1,0581E-06 | protein_coding |
| ENSMUSG00000050350 | Gpr18 | G protein-coupled receptor 18 [Source:MGI Symbol;Acc:MGI:107859] | 17,97335192 | -1,8352621 | **-3,56836232** | 4,15784417 | 59,6541599 | 1,1308E-14 | 4,45308E-13 | protein_coding |
| ENSMUSG00000038860 | Garnl3 | GTPase activating RANGAP domain-like 3 [Source:MGI Symbol;Acc:MGI:2139309] | 20,50731673 | -1,8421331 | **-3,58539746** | 4,33944048 | 51,8284151 | 6,05689E-13 | 1,84853E-11 | protein_coding |
| ENSMUSG00000004359 | Spic | Spi-C transcription factor (Spi-1/PU.1 related) [Source:MGI Symbol;Acc:MGI:1341168] | 25,99766297 | -1,8528893 | **-3,61222891** | 4,70230891 | 61,4416979 | 4,56049E-15 | 1,90518E-13 | protein_coding |
| ENSMUSG00000020256 | Aldh1l2 | aldehyde dehydrogenase 1 family, member L2 [Source:MGI Symbol;Acc:MGI:2444680] | 5,777446677 | -1,8849474 | **-3,69339463** | 2,4867276 | 22,5420561 | 2,05593E-06 | 2,08728E-05 | protein_coding |
| ENSMUSG00000016756 | Cmah | cytidine monophospho-N-acetylneuraminic acid hydroxylase [Source:MGI Symbol;Acc:MGI:103227] | 22,53240663 | -1,8849665 | **-3,69344345** | 4,46937762 | 114,526418 | 9,99261E-27 | 1,48363E-24 | protein_coding |
| ENSMUSG00000036006 | Fam65b | family with sequence similarity 65, member B [Source:MGI Symbol;Acc:MGI:2444879] | 14,29033354 | -1,9001359 | **-3,73248351** | 3,82176013 | 67,2590033 | 2,38081E-16 | 1,19991E-14 | protein_coding |
| ENSMUSG00000038807 | Rap1gap2 | RAP1 GTPase activating protein 2 [Source:MGI Symbol;Acc:MGI:3028623] | 51,91743363 | -1,9177253 | **-3,7782687** | 5,69596175 | 75,9319496 | 2,93611E-18 | 1,78227E-16 | protein_coding |
| ENSMUSG00000039221 | Rpl22l1 | ribosomal protein L22 like 1 [Source:MGI Symbol;Acc:MGI:1915278] | 54,81793473 | -1,920267 | **-3,78493099** | 5,7711332 | 73,9743262 | 7,91397E-18 | 4,60057E-16 | protein_coding |
| ENSMUSG00000034427 | Myo15b | myosin XVB [Source:MGI Symbol;Acc:MGI:2685534] | 16,09077307 | -1,9528927 | **-3,87150015** | 3,98305933 | 62,5126554 | 2,64738E-15 | 1,1776E-13 | protein_coding |
| ENSMUSG00000025432 | Avil | advillin [Source:MGI Symbol;Acc:MGI:1333798] | 4,445459897 | -1,9584558 | **-3,88645763** | 2,12421278 | 25,6206687 | 4,15564E-07 | 4,79601E-06 | protein_coding |
| ENSMUSG00000095334 | | | 6,545451647 | -1,986089 | **-3,96161584** | 2,68661541 | 40,312165 | 2,16457E-10 | 4,61789E-09 | protein_coding |
| ENSMUSG00000027347 | Rasgrp1 | RAS guanyl releasing protein 1 [Source:MGI Symbol;Acc:MGI:1314635] | 72,59897696 | -2,040841 | **-4,11485319** | 6,17826878 | 51,5553096 | 6,96082E-13 | 2,10106E-11 | protein_coding |
| ENSMUSG00000075463 | 4930594M22Rik | RIKEN cDNA 4930594M22 gene [Source:MGI Symbol;Acc:MGI:1925478] | 4,008462458 | -2,0559506 | **-4,15817538** | 1,98747637 | 27,6741698 | 1,43567E-07 | 1,82091E-06 | lincRNA |
| ENSMUSG00000031770 | Herpud1 | homocysteine-inducible, endoplasmic reticulum stress-inducible, ubiquitin-like domain member 1 [Source:MGI Symbol;A | 264,3486892 | -2,0668463 | **-4,18969803** | 8,04517559 | 64,5403991 | 9,45732E-16 | 4,4405E-14 | protein_coding |
| ENSMUSG00000048498 | Cd300e | CD300e antigen [Source:MGI Symbol;Acc:MGI:2387602] | 96,14390299 | -2,0990268 | **-4,28420274** | 6,58865324 | 86,2593055 | 1,57823E-20 | 1,24748E-18 | protein_coding |
| ENSMUSG00000086076 | Ncr3-ps | natural cytotoxicity triggering receptor 3, pseudogene [Source:MGI Symbol;Acc:MGI:2149332] | 2,23762866 | -2,1067428 | **-4,3071775** | 1,09207147 | 24,7648218 | 6,47688E-07 | 7,27766E-06 | pseudogene |
| ENSMUSG00000091421 | Gm4202 | predicted gene 4202 [Source:MGI Symbol;Acc:MGI:3782379] | 8,870827688 | -2,1205558 | **-4,34861446** | 3,14921152 | 20,8645358 | 4,92937E-06 | 4,62502E-05 | ocessed_pseudoge |
| ENSMUSG00000061353 | Cxcl12 | chemokine (C-X-C motif) ligand 12 [Source:MGI Symbol;Acc:MGI:103556] | 5,764072857 | -2,1616719 | **-4,47433077** | 2,57748909 | 33,9162687 | 5,75356E-09 | 9,52023E-08 | protein_coding |
| ENSMUSG00000050138 | Kcnk12 | potassium channel, subfamily K, member 12 [Source:MGI Symbol;Acc:MGI:2684043] | 4,424542653 | -2,2065329 | **-4,6156469** | 2,10305557 | 32,4037428 | 1,25245E-08 | 1,96021E-07 | protein_coding |
| ENSMUSG00000000861 | Bcl11a | B cell CLL/lymphoma 11A (zinc finger protein) [Source:MGI Symbol;Acc:MGI:106190] | 10,44167768 | -2,2154512 | **-4,64426776** | 3,35860797 | 53,2916556 | 2,87525E-13 | 9,26405E-12 | protein_coding |
| ENSMUSG00000041481 | Serpina3g | serine (or cysteine) peptidase inhibitor, clade A, member 3G [Source:MGI Symbol;Acc:MGI:105046] | 15,19809896 | -2,2299994 | **-4,68808706** | 3,9260798 | 53,9536421 | 2,05276E-13 | 6,7526E-12 | protein_coding |
| ENSMUSG00000027254 | Mtap1a | microtubule-associated protein 1 A [Source:MGI Symbol;Acc:MGI:1306776] | 65,88801484 | -2,2298818 | **-4,69095546** | 6,037191 | 50,4627414 | 1,21449E-12 | 3,55829E-11 | protein_coding |
| ENSMUSG00000030147 | Clec4b1 | C-type lectin domain family 4, member b1 [Source:MGI Symbol;Acc:MGI:1917060] | 6,352451546 | -2,2466055 | **-4,74564919** | 2,64304698 | 50,1627293 | 1,4151E-12 | 4,13503E-11 | protein_coding |
| ENSMUSG00000090164 | BC035044 | cDNA sequence BC035044 [Source:MGI Symbol;Acc:MGI:2448540] | 2,424763798 | -2,2630051 | **-4,79990245** | 1,22682834 | 21,5836145 | 3,38733E-06 | 3,29351E-05 | protein_coding |
| ENSMUSG00000059336 | Slc14a1 | solute carrier family 14 (urea transporter), member 1 [Source:MGI Symbol;Acc:MGI:1351654] | 12,19492791 | -2,2750736 | **-4,84022312** | 3,58843983 | 63,5373848 | 1,57352E-15 | 7,23359E-14 | protein_coding |

| | | | | | | | | | | |
|---|---|---|---|---|---|---|---|---|---|---|
| ENSMUSG00000090185 | Gm15523 | predicted gene 15523 [Source:MGI Symbol;Acc:MGI:3782970] | 2,231510036 | -2,3217867 | **-4,9995101** | 1,12746715 | 30,0058027 | 4,30755E-08 | 6,12252E-07 | antisense |
| ENSMUSG00000038264 | Sema7a | sema domain, immunoglobulin domain (Ig), and GPI membrane anchor, (semaphorin) 7A [Source:MGI Symbol;Acc:MGI: | 50,15200228 | -2,3268982 | **-5,0172549** | 5,6413175 | 74,0249876 | 7,71344E-18 | 4,50785E-16 | protein_coding |
| ENSMUSG00000022747 | St3gal6 | ST3 beta-galactoside alpha-2,3-sialyltransferase 6 [Source:MGI Symbol;Acc:MGI:1888707] | 8,283443403 | -2,3275234 | **-5,01942947** | 3,03581354 | 48,4098384 | 3,45829E-12 | 9,54679E-11 | protein_coding |
| ENSMUSG00000034258 | Mfsd7c | major facilitator superfamily domain containing 7C [Source:MGI Symbol;Acc:MGI:2384974] | 34,84080047 | -2,3623674 | **-5,14213475** | 5,12256655 | 121,732811 | 2,64116E-28 | 4,39673E-26 | protein_coding |
| ENSMUSG00000051159 | Cited1 | Cbp/p300-interacting transactivator with Glu/Asp-rich carboxy-terminal domain 1 [Source:MGI Symbol;Acc:MGI:108023] | 2,731476344 | -2,3766232 | **-5,19319789** | 1,51727689 | 29,5191761 | 5,53662E-08 | 7,71965E-07 | protein_coding |
| ENSMUSG00000002007 | Srpk3 | serine/arginine-rich protein specific kinase 3 [Source:MGI Symbol;Acc:MGI:1891338] | 19,10151401 | -2,4234642 | **-5,36457621** | 4,23787956 | 108,281667 | 2,33171E-25 | 3,01395E-23 | protein_coding |
| ENSMUSG00000021200 | Asb2 | ankyrin repeat and SOCS box-containing 2 [Source:MGI Symbol;Acc:MGI:1929743] | 136,6106136 | -2,5170688 | **-5,72417891** | 7,0901956 | 118,420758 | 1,40247E-27 | 2,20127E-25 | protein_coding |
| ENSMUSG00000009092 | Derl3 | Der1-like domain family, member 3 [Source:MGI Symbol;Acc:MGI:1917627] | 4,090866181 | -2,5269749 | **-5,76361857** | 2,00014008 | 24,5563425 | 7,21684E-07 | 8,04989E-06 | protein_coding |
| ENSMUSG00000076617 | Ighm | immunoglobulin heavy constant mu [Source:MGI Symbol;Acc:MGI:96448] | 119,5538928 | -2,5893475 | **-6,01826464** | 6,89903972 | 48,4805167 | 3,33587E-12 | 9,25534E-11 | IG_C_gene |
| ENSMUSG00000004709 | Cd244 | CD244 natural killer cell receptor 2B4 [Source:MGI Symbol;Acc:MGI:109294] | 66,64149383 | -2,6915279 | **-6,45997193** | 6,05380934 | 67,2891997 | 2,34462E-16 | 1,18711E-14 | protein_coding |
| ENSMUSG00000024399 | Ltb | lymphotoxin B [Source:MGI Symbol;Acc:MGI:104796] | 23,43888494 | -2,7952188 | **-6,94136217** | 4,53875037 | 76,5671695 | 2,12849E-18 | 1,32873E-16 | protein_coding |
| ENSMUSG00000008193 | Spib | Spi-B transcription factor (Spi-1/PU.1 related) [Source:MGI Symbol;Acc:MGI:892986] | 8,453335055 | -2,917058 | **-7,55304324** | 3,02308314 | 82,0679894 | 1,31485E-19 | 9,26042E-18 | protein_coding |
| ENSMUSG00000028782 | Bai2 | brain-specific angiogenesis inhibitor 2 [Source:MGI Symbol;Acc:MGI:2451244] | 18,91189004 | -3,0812807 | **-8,46365449** | 4,22742811 | 73,8927143 | 8,24802E-18 | 4,76952E-16 | protein_coding |
| ENSMUSG00000073940 | Hbb-b2 | hemoglobin, beta adult minor chain [Source:MGI Symbol;Acc:MGI:96022] | 8,235784846 | -3,3496883 | **-10,1942822** | 3,014136 | 53,6768066 | 2,36337E-13 | 7,72808E-12 | protein_coding |
